# *De novo* transcriptome of the cosmopolitan dinoflagellate *Amphidinium carterae* to identify enzymes with biotechnological potential

**DOI:** 10.1038/s41598-017-12092-1

**Published:** 2017-09-15

**Authors:** Chiara Lauritano, Daniele De Luca, Alberto Ferrarini, Carla Avanzato, Andrea Minio, Francesco Esposito, Adrianna Ianora

**Affiliations:** 1 Integrative Marine Ecology Department, Stazione Zoologica Anton Dohrn, Villa Comunale, 80121 Napoli, Italy; 2 Università degli Studi di Verona, Ca’ Vignal 1, Strada Le Grazie 15, 37134 Verona, Italy

## Abstract

Dinoflagellates are phytoplanktonic organisms found in both freshwater and marine habitats. They are often studied because related to harmful algal blooms but they are also known to produce bioactive compounds for the treatment of human pathologies. The aim of this study was to sequence the full transcriptome of the dinoflagellate *Amphidinium carterae* in both nitrogen-starved and -replete culturing conditions (1) to evaluate the response to nitrogen starvation at the transcriptional level, (2) to look for possible polyketide synthases (PKSs) in the studied clone (genes that may be involved in the synthesis of bioactive compounds), (3) if present, to evaluate if nutrient starvation can influence PKS expression, (4) to look for other possible enzymes of biotechnological interest and (5) to test strain cytotoxicity on human cell lines. Results showed an increase in nitrogen metabolism and stress response in nitrogen-starved cells and confirmed the presence of a type I β-ketosynthase. In addition, L-asparaginase (used for the treatment of Leukemia and for acrylamide reduction in food industries) and cellulase (useful for biofuel production and other industrial applications) have been identified for the first time in this species, giving new insights into possible biotechnological applications of dinoflagellates.

## Introduction

Marine phytoplankton generates about half of the global primary productivity, regulating global biogeochemical cycles and supporting valuable fisheries^1,2^. A subset of these species produces a series of compounds/toxins involved in harmful algal blooms with serious economic consequences for the aquaculture and fishing industries and/or deleterious impacts on human health^3–5^. Microalgae are known to produce not only toxins, but also a series of compounds derived from primary or secondary metabolism with applications in several market sectors: cosmetics, nutrition, bioremediation, aquaculture and treatment of human pathologies^3,6–11^. Recently, new insights have been gained into both the ecology and biotechnology of these important marine species thanks to genome and transcriptome sequencing projects. Not many microalgal genomes have been sequenced to date. Genomes are available for the rhodophyte *Cyanidio schyzonmerolae*, the green-algae *Chlamydomonas reinhardtii*, the chlorophytes *Ostreococcus* and *Micromonas*, the diatoms *Thalassiosira pseudonana* and *Phaeodactylum tricornutum*, and the coccolithophore *Emiliania huxleyi* (as reviewed in ref.^12^). Other genomes are in progress, such as the diatoms *Skeletonema marinoi* (http://cemeb.science.gu.se/research/target-species-imago%20/skeletonema-marinoi) and *Pseudo-nitzschia multistriata* ^13^. Dinoflagellates are known to produce a wide spectrum of bioactive molecules^6,10,11^, but molecular resources are still poor because they have very large genomes, ranging from 1.85 to 112 Gbp^14,15^. However, several transcriptomes have been sequenced and many of these are included in the Marine Microbial Eukaryote Transcriptome Sequencing Project (MMETSP) (http://marinemicroeukaryotes.org/)^16^.

In this study, we present the full-transcriptome of the dinoflagellate *Amphidinium carterae*. Considering that several studies have shown that different environmental conditions (e.g. nutrient starvation, UV radiation and ocean acidification) may influence microalgal growth and induce a reorientation of their metabolism and the production of bioactive compounds^17–22^, we performed RNA sequencing in both control- (Keller medium) and nitrogen-starvation culturing conditions. The aims of this study were (1) to evaluate the response of the dinoflagellate to nitrogen starvation, (2) to look for possible polyketide synthases (PKSs) in this *A. carterae* clone, (3) if PKS is present, to evaluate if nutrient starvation can influence PKS expression, (4) look for other possible enzymes of biotechnological interest and (5) test strain cytotoxicity on human cells. Indeed, a negative response for cytotoxicity would indicate that this strain likely does not produce cytotoxic compounds.

*Amphidinium* sp. is an athecate dinoflagellate^23^ and a widely distributed species, found in both temperate^24^ and tropical waters^25^. The genome is not available, but there are several EST sequences in GenBank and a transcriptome in MMETSP available for *Amphidinium* sp. Until now several compounds have been isolated from different *Amphidinium* strains, such as haemolysins^26^, amphirionin‑4^27^, karatungiols A and B^28^, amphidinols and more than 45 cytotoxic macrolides, known as amphidinolides^27,29–32^. Not all *Amphidinium* clones produce the same compounds, are toxic and/or show the same activities^26–32^. In addition, not all have the enzymatic machinery responsible for polyketide synthesis, the polyketide synthases (PKS)^4,33^.

PKS have been suggested to be responsible for the synthesis of toxins and other polyketides with interesting ecological and biotechnological functions (e.g. antipredator, allelopathic, anticancer, antifungal activity and/or beneficial effects for the treatment of Alzheimer’s disease^4,12,14,29,33,34^. There are three major functional groups of PKSs. Type I PKS are large multifunctional proteins, comprising several essential domains: acyltransferase domain (AT), β-ketosynthase domain (KS) and acyl carrier protein (ACP); they can also include β-ketoacyl reductase (KR), enoyl reductase (ER), methyl transferases (MT), thioesterases (TE) and dehydrogenase (DH) domains^35,36^. Type II PKS consist of mono-functional proteins with each catalytic domain on a separate peptide that form complexes for polyketide composition^36,37^. Type III PKSs are self-contained homodimeric enzymes where each monomer performs a specific function^35,36,38^. PKS enzymes have been found in many microalgal species and, in particular, Type I modular PKS genes have been identified, for example, in the microalgae *Karenia brevis*, *Heterocapsa circularisquama*, *Heterocapsa triquetra*, *Alexandrium ostenfeldii*, *Azadinium* sp. and various *Amphidinium* sp.^14,15,39,40^.

Other enzymes of biotechnological interest have been isolated from several marine organisms, mainly bacteria, but very few studies have focused on microalgae^4,41^. Enzyme studies in dinoflagellates have mainly focused on PKSs (e.g. refs^4,14,39,40^). In this study, we also looked for sequences coding for L-asparaginase and cellulase in the transcriptome of the dinoflagellate *A. carterae*. L-asparaginase (EC 3.5.1.1) is an enzyme that catalyzes the hydrolysis of L-asparagine to L-aspartic acid^42^. It is used to treat acute lymphoblastic leukemia^43^, acute myeloid leukemia^44^, and non-Hodgkin’s lymphoma^45^, since malignant cells have a reduced capacity to produce asparagine synthetase and rely on asparagine supplied directly from the blood. By limiting the supply of asparagine, the growth of cancer cells is inhibited^46,47^. This enzyme also has applications in the food industry (i.e. to reduce the carcinogenic acrylamide in several foods since asparagine is one of the precursors of acrylamide)^48–50^. For instance, L-asparaginase from *Aspergillus oryzae* added to French fries, biscuits, crisp bread, and fabricated chips, efficiently reduces formation of acrylamide^51^. L-asparaginases are produced by a large number of microorganisms that include bacteria, fungi, and yeast^50,52^. Asparaginase activity was found in the green algae *Chlorella vulgaris* by Ebrahiminezhad *et al*.^47^, but, to our knowledge there are no reports on the presence of this enzyme in dinoflagellates. However, sequences for asparaginase are available in GenBank (https://www.ncbi.nlm.nih.gov/genbank/) for the diatoms *Phaeodactylum tricornutum*, *Fragilariopsis cylindrus* and *Thalassiosira pseudonana*, and for the flagellate *Nannochloropsis gaditana*.

Cellulase is an enzyme complex exhibiting several activities involved in cellulose hydrolysis: endoglucanases (EC 3.2.1.4), exoglucanases, including cellobiohydrolases (EC 3.2.1.91) and d-cellodextrinases (EC 3.2.1.74), and β-glucosidases (EC 3.2.1.21)^53^. It is traditionally used in food, detergent, brewing, textile, paper manufacturing, and animal feed industries^54,55^. In addition, cellulase has attracted increasing attention in recent years due to its great future applications in biofuel production through biodegradation of lignocellulosic materials^56^. Recently, it was shown that eukaryotic microalgae can use cellulose as an alternative carbon source for growth^57^ and, hence, they may have the enzymatic machinery to use it. Kwok and Wong (ref.^58^) examined the effects of cellulase inhibitors on cell cycle progression in the dinoflagellate *Crypthecodinium cohnii* demonstrating that cellulase activity may play a role during cell cycle progression. However, cellulases are unfortunately scarcely studied in microalgae and cellulase sequences are available in GenBank only for the dinoflagellate *Lingulodinium polyedrum* and for the diatom *Thalassiosira oceanica*.

## Methods

### Cell culturing and harvesting

*Amphidinium carterae* (clone FE102) was cultured in Keller medium (K)^59^. Experimental culturing for both control and nitrogen starvation conditions was performed in 2 litre polycarbonate bottles (each experiment was performed in triplicate) constantly bubbled with air filtered through 0.2 µm membrane filters. For the control condition, normal K medium was used, while for the nitrogen-starvation experiment K medium was prepared with low concentrations of nitrogen (30 µmol L^−1^ of NO_3_^−^; N-starvation condition). Cultures were kept in a climate chamber at 19 °C on a 12:12 h light:dark cycle at 100 µmol photons m^−2^ s^−1^. Initial cell concentrations were about 5000 cells/mL for each experiment and culture growth rate was monitored, using the equation for net growth estimates^60^. Culture aliquots were sampled during the stationary phase (on the same day and at the same time of day for each replicate to avoid possible interference by intrinsic circadian rhythms), and centrifuged for 15 minutes at 4 °C at 1900 g (Eppendorf, 5810 R). For RNA extractions, for both RNA sequencing (RNAseq) and reverse transcription-quantitative PCR (RT-qPCR), pellets (triplicates for each condition and for each technique) were re-suspended in 500 µL of TRIZOL© (Invitrogen, Carlsbad, CA), incubated for 2–3 minutes at 60 °C until completely dissolved and kept at −80 °C. For chemical extractions (for the cytotoxicity assay), pellets (triplicates for each condition) were frozen directly at −80 °C.

### RNA extraction and cDNA synthesis

For RNA extraction, cells previously frozen in TRIZOL® were lysed using half a spatula of glass beads (about 200 mg; Sigma-Aldrich, Milan, Italy) for each 2 mL tube, incubating and mixing tubes for 10 min at 60 °C and maximum speed in the Thermo Shaker BS100 (Biosan). RNA was then extracted using the Direct-zol^TM^ RNA MiniPrep (Zymo Research), following the manufacturer’s instructions. RNA quantity and purity were assessed by Nano-Drop (ND-1000 UV-Vis spectrophotometer; NanoDrop Technologies) monitoring the absorbance at 260 nm and the 260/280 nm and 260/230 nm ratios (Both ratios were about 2.0). RNA quality was evaluated by gel electrophoresis that showed intact RNA, with sharp ribosomal bands. Total RNA quality was evaluated by measuring the RNA Integrity Number (RIN) with Agilent 2100 Bioanalyzer (Agilent Technologies, Inc.). High quality (RIN > 8) RNA was used for both RNAseq and RT-qPCR. For RT-qPCR, 500 ng/replicate were retrotranscribed into cDNA with the iScriptTM cDNA Synthesis Kit (BIORAD, Hercules, CA) following the manufacturer’s instructions.

### Library preparation and sequencing

RNA-Seq libraries, prepared for both control and nitrogen starvation conditions, were constructed from 2.5 µg of total RNA using the Illumina TruSeq® Stranded mRNA kit (Illumina Inc., San Diego, CA, USA) according to the manufacturer’s instructions. The libraries were size selected using the Pippin Prep automated gel electrophoresis system (Sage Science, Beverly, MA, USA) for 350 to 550 bp. Paired-end sequencing (2 × 100 bp) was performed with the HiSeq. 1000 Illumina platform using the TruSeq SBS v3-HS kit (200 cycles) and TruSeq PE Cluster v3-cBot-HS kit (Illumina).

### Assembly, functional annotation and differential expression analysis

Raw reads with more than 10% of undetermined bases or more than 50 bases with a quality score <7 were discarded. Reads were then clipped from adapter sequences using Scythe software (https://github.com/vsbuffalo/scythe), and low quality ends (Q score < 20 on a 10 nt window) were trimmed with Sickle (https://github.com/vsbuffalo/sickle). To maximize the sensitivity of transcripts reconstruction *de novo* assembly was performed with both the Oases/Velvet assembler (v. 0.2.08) with multiple k-mers (21; 25; 27; 29) and with Trinity (v. 1.8.26) using a minimum contig length of 200 bp^61,62^. Contigs from different assemblies were pooled in a single dataset and were then processed with CD-HIT-EST (minimum identity clustering threshold = 0.95) and TGICL pipeline to remove sequence redundancy^63,64^. Functional annotation of non-redundant contigs was performed using Blast2GO software with SwissProt database and default parameters^65^. To assess the expression profiles, reads from each sample were mapped against the contigs and expression abundances were quantified using RSEM (version 1.1.21) with default settings^66^. Only contigs with more than 100 aligned reads in at least one condition were considered further. The R package DESeq.^67^ was used to identify differentially expressed genes (FDR ≤ 0.05; |Log2(FC)| ≥ 1). Raw read counts were transformed to FPKM (fragments per kilobase of exon per million fragments mapped). In addition, functional categories were also deeply investigated for both the full transcriptome and the differentially expressed genes (DEGs) by using the Kyoto Encyclopedia of Genes and Genomes (KEGG) annotation^68^. Raw reads and assembled transcripts have been deposited in GenBank (GEO database)^69^ and are freely available under the series entry GSE94355.

### Identification of β-ketosynthase, asparaginase and cellulase

The nucleotide sequences corresponding to KS, L-asparaginase, and cellulase genes from our transcriptome were translated into the relative amino acid sequence using the Translate tool service available at the server ExPASy^70^. To retrieve orthologs of these proteins, we used the generated protein sequences for each gene as queries against two different protein databases: UniProtKB in the BLASTP server at EBI (http://www.ebi.ac.uk/Tools/sss/ncbiblast/), and EggNOG v. 4.5 (available at http://eggnogdb.embl.de/#/app/home)^71^. The first is a comprehensive and annotated database including all that is known about a particular protein sequence, whereas the latter includes only orthologous sequences, which are more likely to conserve their function than paralogs and are therefore of crucial importance for pharmacological and phylogenetic studies^71^. In UniProtKB, we searched both the entire database and its subsection “Swiss-Prot”, the latter including only manually curated and non-redundant sequences, and therefore were more accurate but less taxonomically representative. For this search, we used “BLOSUM62” as substitution matrix and retained only the sequences with a percentage of identity >25% in order to avoid selection of non-homologous sequences^72^. The list of sequences retrieved from these databases is available as Supplementary Tables S1, S2 and S3 for β-ketosynthase, L-asparaginase and cellulase, respectively. Functional prediction and identification of conserved amino acid residues in the active site of the selected transcripts were obtained using the InterProScan application (available at https://www.ebi.ac.uk/interpro/search/sequence-search)^73^.

In order to assess if KS, L-asparaginase and cellulase were secretory or non-secretory proteins, we predicted the presence of a signal peptide in the transcripts using SignalP-4.1^74^. This software uses three indices called S-score, C-score and Y-score to evaluate the presence or absence of signal peptides^74^. In detail, a high S-score in a particular amino acid position indicates that the corresponding amino acid is part of a signal peptide, while low values indicate that the amino acid is part of a mature protein. The C-score is the “cleavage site” score and should only be significantly high at the position immediately after the cleavage site (the first residue in the mature protein). The Y-score is a derivative of the two indices that provides a better cleavage site prediction than the raw C-score alone. Indeed, multiple high-peaking C-scores can be found in one sequence, but only one is the true cleavage site. The cleavage site is assigned from the Y-score where the slope of the S-score is steep and a significant C-score is found^75^.

### Phylogenetic analysis

The amino acid sequences obtained from different datasets were aligned in MEGA7^76^ using the ClustalW algorithm^77^. Maximum likelihood phylogenetic analyses were carried out in RaxML^78^, with 1000 bootstrap replicates and using the best fitting evolutionary model for each gene (LG + G for KS and L-asparaginase; and WAG + G for cellulase) using the Bayesian Information Criterion (BIC) as implemented in MEGA7. The resulting trees (Supplementary Figs S1, S2 and S3) were visualised and graphically edited in FigTree v1.4.3 (http://tree.bio.ed.ac.uk/software/figtree/).

### Oligo design

Primers for reference genes (RGs) and genes of interest (GOI) were designed using the software Primer3 v. 0.4.0 (http://frodo.wi.mit.edu/primer3/). Supplementary Table S4 lists selected RGs and GOI, their functions, primers’ sequences and efficiencies. To determine the specificity of the amplification, designed primer pairs were first tested in PCR, optimized in a GeneAmp PCR System 9700 (Perkin Elmer) according to the reaction conditions detailed in ref.^79^. Amplified PCR products were then analyzed by 1.5% agarose gel electrophoresis in TBE buffer. Only PCR products that showed a single band on agarose gel were further considered for this study. The resulting single bands for each gene were excised from the gel and extracted with the GenElute^TM^ Gel Extraction Kit (SIGMA). Sequence reactions were obtained with the BigDye Terminator Cycle Sequencing technology (Applied Biosystems, Foster City, CA), purified in automation using the Agencourt CleanSEQ Dye terminator removal Kit (Agencourt Bioscience Corporation, 500 Cummins Center, Suite 2450, Beverly MA 01915 - USA) and a robotic station Biomek FX (Beckman Coulter, Fullerton, CA). Products were analyzed on an Automated Capillary Electrophoresis Sequencer 3730 DNA Analyzer (Applied Biosystems). The identity of each sequence was confirmed using the blastn function.

### Reverse transcription-quantitative polymerase chain reaction (RT-qPCR)

In order to normalize expression levels of specific genes of interest (GOI), a panel of putative reference genes (i.e. alpha and beta tubulins, ubiquitin, cyclin-dependent kinase 3, glyceraldehyde 3-phosphate dehydrogenase; Supplementary Table S4) was first screened in the two experimental conditions: control and N-starvation conditions. The three different software BestKeeper^80^, geNorm^81^ and NormFinder^82^ were utilized to identify the best RGs. Serial dilutions of cDNA were used to determine primer reaction efficiency (E) and correlation factor (R^2^) (Supplementary Table S4). Standard curves were generated with five dilution points by using the cycle threshold (Ct) value versus the logarithm of each dilution factor using the equation E = 10^−1/slope^. The program was set to reveal the melting curve of each amplicon from 60 °C to 95 °C, and read every 0.5 °C, in order to ensure that a single product was amplified for each primer pair. Selected GOI were: heat-shock proteins 70 and 90 (HSP70 and HSP90), nitrate transporter, nitrate reductase and nitrite reductase (NATETR, NATE and NITE, respectively), histidase (HAL), 3-hydroxyacyl-CoA dehydrogenase (HADH), malate dehydrogenase (MDH), Polypeptide n-acetylgalactosaminyltransferase 1 (GALNT1), phosphoglycerate kinase (PGK), Phospholipase D1 (PLD1), light-indipendent protochlorophyllide reductase (LIPOR), permease (PER), β-ketosynthase (KS). RT-qPCR was performed as in ref.^17^ in a Viia7 real-time PCR system (Applied Biosystem). All RT-qPCR reactions were carried out in triplicate to capture intra-assay variability and included three no-template negative controls (NTC) for each primer pair. To study expression levels for each GOI relative to the most stable RGs, we used the REST tool (Relative Expression Software Tool)^83^. Control condition was represented by microalgae cultured in normal medium. Statistical analysis was performed using GraphPad Prim statistic software, V4.00 (GraphPad Software; http://www.graphpad.com/quickcalcs/). Normality of data was tested by using the Anderson-Darling test^84^ with the PAST software^85^ (v.3.15; Data were normally distributed as reported in Supplementary Table S5).

### Chemical extraction

Fifty mL of distilled water was added and samples were sonicated for 1 min at maximum speed. The same volume of acetone was added and, after 50 min mixing at room temperature, samples were evaporated under nitrogen stream down to half of their volume. About 1 g of Amberlite XAD16N resin (Sigma-Aldrich) was added to each sample. After 50 min of mixing at room temperature, 18 mL of water was added for a resin washing step. A centrifugation step (15 min at 3500 g at room temperature) allowed the elimination of water and the resin was incubated with 10 ml acetone for 50 min. Centrifugation for 15 min at room temperature allowed cells to settle and removal of the resin, while the supernatants, that were the final extracts, were freeze-dried and stored at −20 °C until testing. Before performing the cytotoxicity assay, extracts were first diluted at 1 mg/mL with sterilized MilliQ water and 2.5% DMSO.

### Cytotoxicity assay

Cytotoxicity was evaluated to check that microalgal extracts did not affect the survival of human hepatocellular liver carcinoma (HepG2 ATCC HB-8065™) cells after 24 h of exposure. The assay was performed as in ref.^86^. Briefly, 20,000 HepG2 cells were seeded per well and grown overnight. They were incubated with 50 μg/mL microalgal extract diluted in MEM Earle’s, without fetal bovine serum (total volume was 100 µl). Ten μL of CellTiter 96® AQueous One Solution Reagent (Promega, Madison, WI, USA) was added and plates were then further incubated for 1 h. Absorbance was measured at 485 nm in a DTX 880 Multimode Detector. Results were calculated as % survival compared to negative (assay media) and positive (Triton X-100; Sigma-Aldrich) control.

### Data availability statement

Data are available and sequences are deposited in GenBank.

## Results and Discussion

### Transcriptome sequencing, *de novo* assembly and functional annotation

The RNA sequencing from samples cultured in normal culturing condition and in nitrogen-starvation (3 biological replicates each) yielded 232,474,767 paired-end reads (2 × 100 bp) corresponding to 46.5 Gbp of sequence data. A combined *de novo* assembly strategy, involving different transcriptome assemblers, was employed to maximize the comprehensiveness of the reconstructed transcriptome. Actually, even when starting from the same sequences and similar parameters, the output of different assemblers can be quite different, and it is well recognized that different assemblers are more efficient at reconstructing different sets of sequences^87,88^. In particular, Trinity and Velvet/Oases assemblers were used implementing a range of k-mer sizes to generate many sequences that were then pooled into a super-set of 883,121 putative transcripts (Supplementary Table S6 and Supplementary Fig. S4). After clustering with an identity threshold of 95% and removal of redundant sequences a total of 112,530 putative transcripts were obtained. Of the initial reads 98% could be mapped to the assembled putative transcripts. To increase the confidence of the assembly only putative transcripts covered by at least 100 paired-end reads in at least one of the biological replicates were considered in the following analyses. A total of 70,787 putative transcripts with an average length of 2055 bp were obtained (Table 1).

**Table 1:** Summary statistics of putative transcripts assembly.

Number of putative transcripts	70,787
Average length of putative transcripts	1490
Maximum length of putative transcripts	21769
N50	2055
N90	793

**Figure 1:**
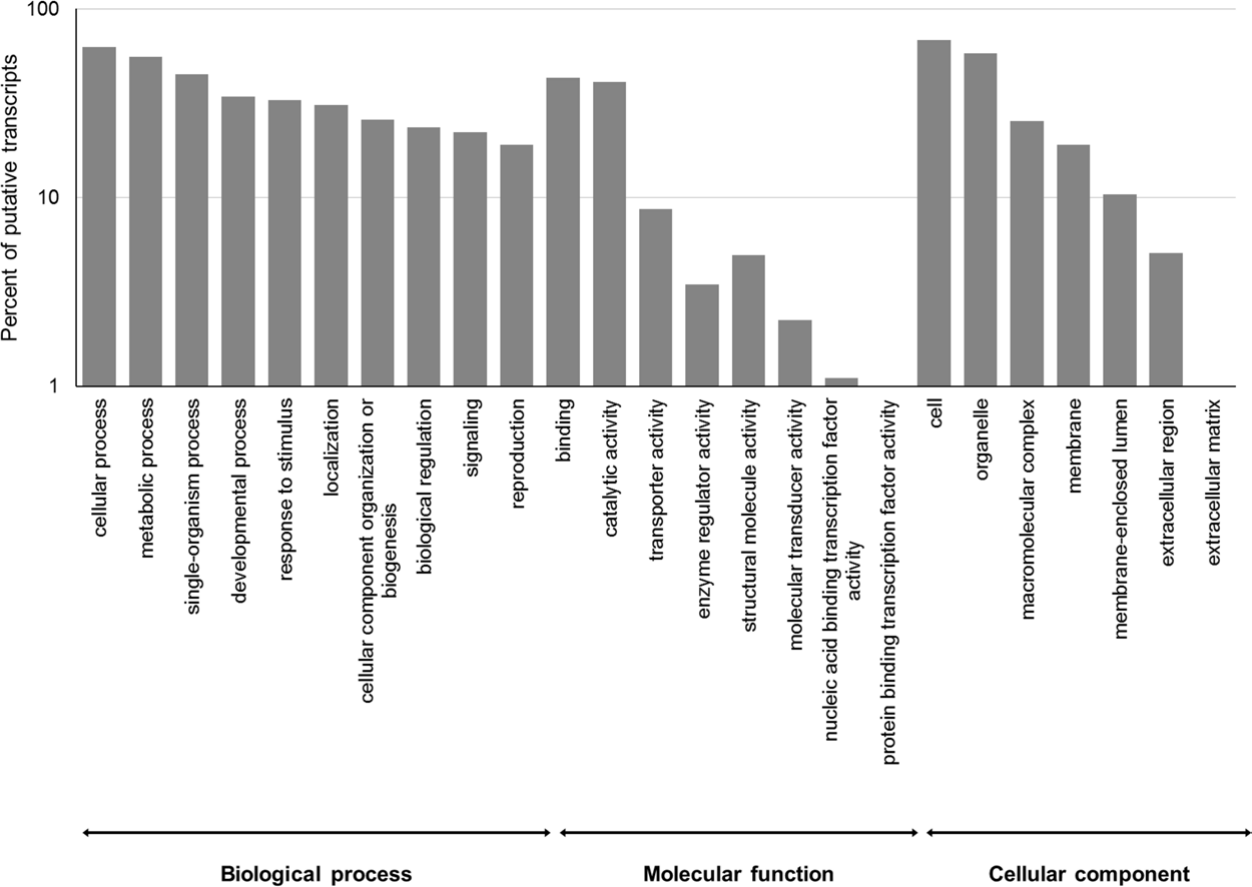
Histogram of GO classifications of *Amphidinium carterae* putative transcripts. Results are summarized for the three main GO categories: biological processes, cellular component and molecular function. The y-axis indicates the percentage of sequences for each category.

A sequence similarity search was performed against the Swiss-Prot database (E-value < 1e^−05^) using the BLASTx algorithm and 23,076 (33%) putative transcripts showed significant sequence similarity. Moreover, to provide a functional classification putative transcripts were mapped to Gene Ontology (GO) terms (Fig. 1). GO terms were assigned to 25,361 (35.8%) putative transcripts. Cellular process (15,861 putative transcripts) and metabolic process (14,154 putative transcripts) were the most highly represented groups. Genes involved in other important biological processes such as response to stimulus (8336 putative transcripts) and signaling (5606 putative transcripts) were also highly represented. Functional categories were also deeply investigated by using the Kyoto Encyclopedia of Genes and Genomes (KEGG) annotation. Using the KEGG pathway classification, 135 metabolic pathways were found (Supplementary Table S7). Of these, the biosynthesis of antibiotics pathway (Pathway ID map01130), was the one with the highest number of enzymes associated to it (135). Other highly represented pathways were purine metabolism (map00230), amino sugar and nucleotide sugar metabolism (map00520), cysteine and methionine metabolism (map00270), starch and sucrose metabolism (map00500) and pyrimidine metabolism (map00240).

Benchmarking Universal Single-Copy Orthologs (BUSCOs) are ideal for quantification of completeness of a genome, annotation or transcriptome^89^. Analysis with BUSCO software (v3.0.0) performed using eukaryote dataset (odb9) identified 72.4% of 303 BUSCOs (68.4% complete; 4% fragmented) suggesting a large portion of the transcriptome was represented with mostly full length transcripts (Supplementary Table S8).

### Differential expression analysis

**Figure 2:**
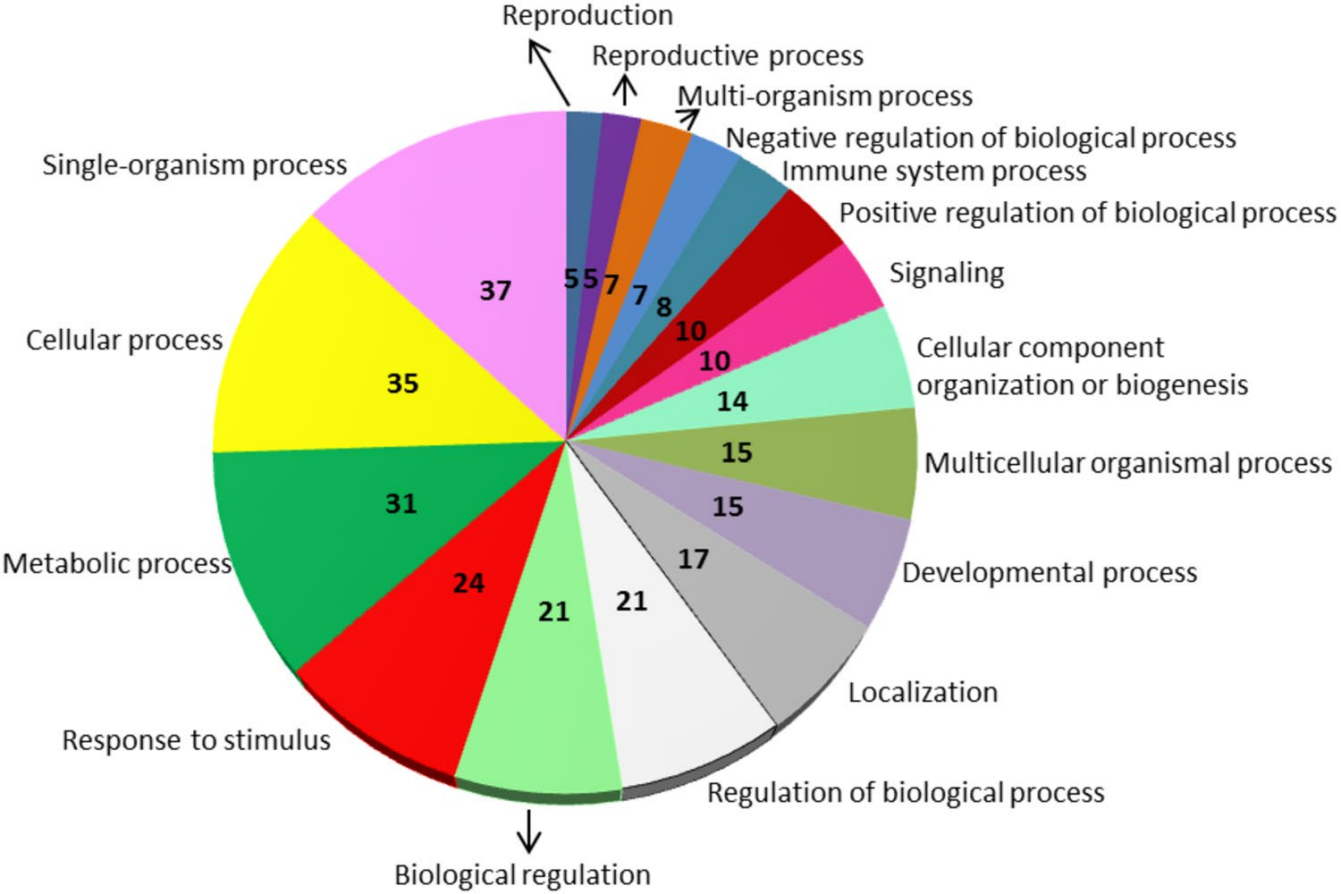
Pie chart showing sequence distribution of the differentially expressed genes within biological processes.

Differential expression analysis identified 220 genes with significant expression variations (|LogFC| > 0.5; P value adjusted ≤ 0.05) in nitrogen-starvation condition relative to control (i.e. *A. carterae* cultured in complete K medium). Among the 220 DEGs, 31 had no GO assignment, while the remaining 189 included 87 up-regulated and 102 down-regulated genes. Functional classification analysis showed that the top represented classes among DEGs were single-organism process, cellular process, metabolic process and response to stimulus, while the least represented class was reproduction (Fig. 2). The full list of DEGs, log2 fold change, adjusted P value (padj), and their GO annotation can be retrieved from Supplementary Table S9. Among the DEGs, the ones showing the highest expression in nitrogen starvation condition were the thylakoid lumenal 15 kda protein (padj = 9,90E-13), the enzyme malate dehydrogenase (MDH; EC 1.1.1.37), involved in the citrate cycle^90^ (TCA cycle) (padj = 7.24E-26), the protein mei2-like 6, involved in meiosis^91^ (padj = 2,14E-11), a peroxisomal multifunctional enzyme type 2 (padj = 2,14E-11), involved in lipid metabolism (http://www.uniprot.org/uniprot/P51659), and a Permease (PER) with putative purine nucleobase transmembrane transporter activity (http://www.ebi.ac.uk/interpro/entry/IPR006043; padj = 1.22E-09). Conversely, nitrogen starvation induced a strong down-regulation of the Light-independent protochlorophyllide reductase (LIPOR) involved in chlorophyll biosynthetic process^92^ (padj = 3.60E-23). KEGG metabolic pathways were also identified for both up- and down-regulated transcripts with GO and enzyme code assignment (Supplementary Tables S10 and S11). For the up-regulated transcripts, there were 30 pathways, but only for 8 pathways there were at least 2 enzymes involved (i.e. nitrogen metabolism, carbon fixation pathways in prokaryotes and carbon fixation in photosynthetic organisms, pyruvate metabolism, biosynthesis of antibiotics, fatty acid degradation and fatty acid elongation, and glycerolipid metabolism; Supplementary Table S10). By contrast, the down-regulated transcript set had no prominent pathways and only 13 were assigned with only 1 transcript each (except for biosynthesis of antibiotics pathway; Supplementary Table S11). Among the 31 transcripts without GO assignment, there were 18 up-regulated and 13 down-regulated but the functions of their products remain unknown. To validate differential expression analysis between the control and nitrogen-starvation conditions, reverse transcription-quantitative PCR (RT-qPCR) was performed. Eighteen transcripts were chosen for RT-qPCR analysis and the primers employed are listed in Supplementary Table S4. A significant positive correlation was established between RNAseq and RT-qPCR (R = 0.8679, p value < 0.00001), supporting the results obtained by RNA-Seq.

### Effects of nitrogen starvation on gene expression changes

In order to normalize expression levels of specific GOI upon nitrogen starvation stress, putative reference genes were analyzed by using the three different software BestKeeper^80^, geNorm^81^ and NormFinder^82^. All three concorded that the two most- stable RGs were UB and CDK (Supplementary Fig. S5 for details). Hence, these two genes were used to normalize expression levels of selected genes of interest in RT-qPCR analyses.

**Figure 3:**
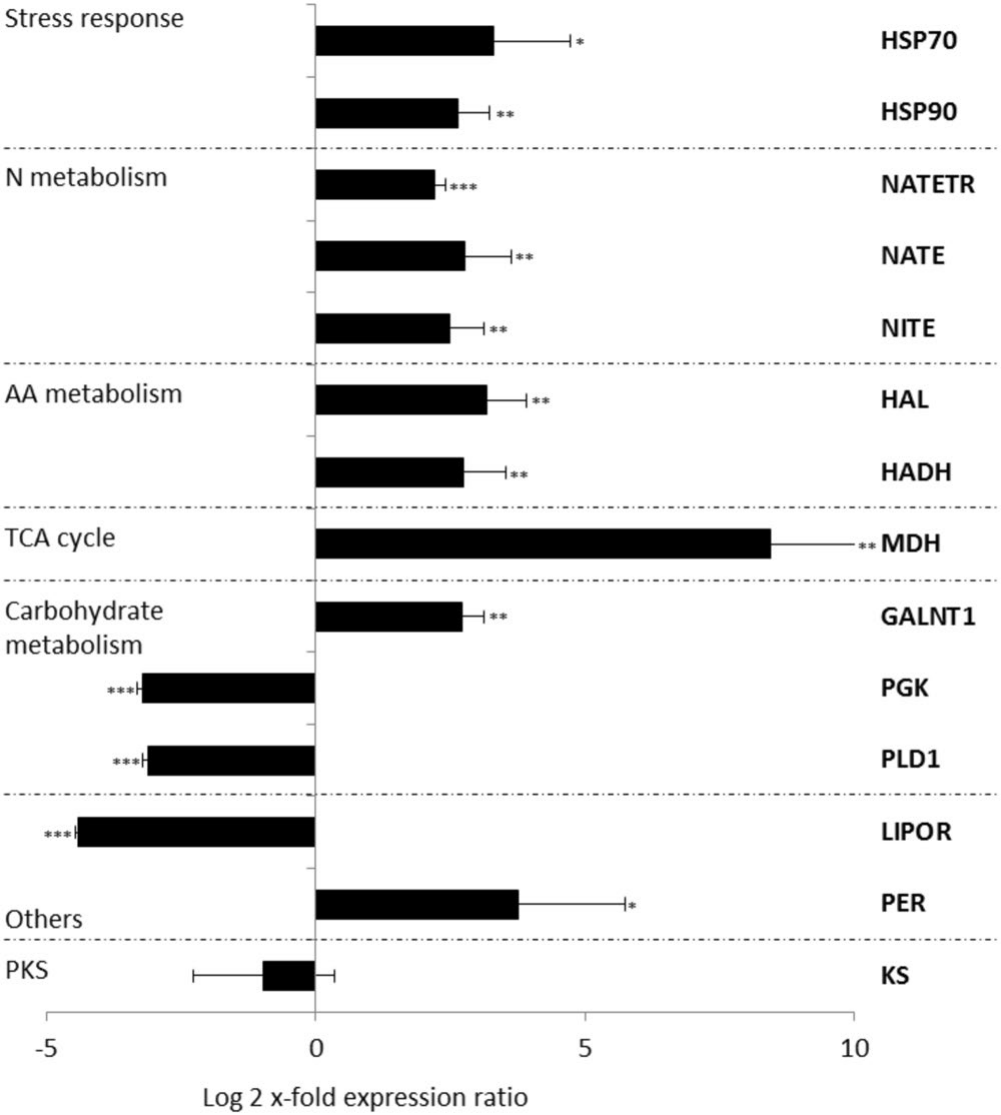
Expression levels of selected genes in *Amphidinium carterae* cells cultured in nitrogen starvation compared to the control condition (i.e. culturing in complete medium; represented in the figure by the x-axis). Data are represented as log2 x-fold expression ratio ± SD (n = 3). Gene abbreviations are: heat-shock proteins 70 and 90 (HSP70 and HSP90), nitrate transporter, nitrate reductase and nitrite reductase (NATETR, NATE and NITE, respectively), histidase (HAL), 3-hydroxyacyl-CoA dehydrogenase (HADH), malate dehydrogenase (MDH), Polypeptide n-acetylgalactosaminyltransferase 1 (GALNT1), phosphoglycerate kinase (PGK), Phospholipase D1 (PLD1), light-indipendent protochlorophyllide reductase (LIPOR), permease (PER), β-ketosynthase (KS). N is for nitrogen and AA for amino acids.

Nitrogen is required for the synthesis of amino acids, nucleic acids, chlorophylls, and toxins^93^. Hence, changes in the concentrations of various nitrogenous compounds can strongly affect both primary and secondary metabolism. Microalgae need to cope with various concentrations of nitrogen, particularly in the open ocean where it is often limiting. As often happens during stressful conditions, *A. carterae* cultured in nitrogen-starvation showed increased expression of heat shock proteins 70 and 90 (HSP70 and HSP90, respectively). RT-qPCR confirmed the up-regulation of 3.3 log2 x-fold for heat shock protein 70 (HSP70; Student’s t-test, p < 0.05) and 3.8 log2 x-fold for heat shock protein 90 (HSP90; Student’s t-test, p < 0.01) (Fig. 3). HSPs are molecular chaperones that can be involved in protein folding and unfolding, and degradation of mis-folded or aggregated proteins^94^. HSPs may be activated in response to various environmental stress factors, including nutrient starvation^17,95,96^. In addition, considering the deficiency of nitrogen, cells showed an increase in nitrate transporter, nitrate reductase and nitrite reductase (NATETR, NATE and NITE, respectively) that were 2.2, 2.8 and 2.5 log2 x-fold up-regulated, indicating an increase in nitrogen metabolism (Student’s t-test, p < 0.001 for NATETR and p < 0.01 for NATE and NITE, Fig. 3). Similar results were observed in other dinoflagellates and diatoms exposed to nitrogen starvation^97,98^. Microarray analysis of N-depleted *Karenia brevis* (dinoflagellate) cultures revealed an increase in the expression of transcripts involved in N-assimilation (including nitrate and ammonium transporters) compared to nutrient replete cells^98^. Similarly, the diatoms *Thalassiosira pseudonana*, *Fragilariopsis cylindrus* and *Pseudo-nitzschia multiseries* increased nitrate transporter and nitrite reductase transcripts^97^. Nitrate reductase was up-regulated in both *T. pseudonana* and *P. multiseries*, whereas no differences were observed in nitrogen starvation for *F. cylindrus* ^97^.

Nitrogen starvation determines a series of physiological, behavioural and transcriptomic modifications to ensure survival. These include changes in amino acid and carbohydrate metabolism, and TCA cycle. In this study, histidase (HAL, involved in histidine catabolism) and 3-hydroxyacyl-CoA dehydrogenase (HADH, involved in valine, leucine and isoleucine degradation) showed 3 log2 x-fold up-regulation (Student’s t-test, p < 0.01 for both, Fig. 3). This was in line with the expected metabolic changes upon nitrogen starvation^97^. Intracellular levels of nitrogenous molecules, such as free amino acids, are expected to be reduced when the growth of the low-nitrate cultures became yield limited, as found for the diatom diatom *T. pseudonana* ^99^. Regarding carbohydrate metabolism, there was the decrease of phosphoglycerate kinase (PGK) involved in Glycolysis/Gluconeogenesis and Phospholipase D1 (PLD1) involved in glycerophospholipid metabolism and the increase of Polypeptide n-acetylgalactosaminyltransferase 1 (GALNT1) involved in the synthesis of oligosaccharides (Student’s t-test, p < 0.01 for GALNT1, p < 0.001 for PGK and PLD1; Fig. 3). In the diatoms *T. pseudonana*, *F. cylindrus* and *P. multiseries* there was also a general reduction in carbohydrate metabolism upon nitrogen starvation^97^. In the freshwater microalgae *Haematococcus pluvialis* the first response to nitrogen starvation was an intensive production of carbohydrates, up to 63% of dry weight, but after the second experimental day there was a reduction to 41% of dry weight^9,100^. Up-regulation of the citric acid cycle (tricarboxylic acid, TCA) was expected, as found in N-starvation in *Chlamydomonas reinhardtii* for the production of carbon backbones for restoration of amino acid concentrations^101^. In this study, malate dehydrogenase (MDH, involved in the TCA cycle) was also 8.4 log2 x-fold up-regulated (Student’s t-test, p < 0.01; Fig. 3).

Other two transcripts analysed by RT-qPCR were the light-independent protochlorophyllide reductase (LIPOR) and a permease (PER). Strong down-regulation of LIPOR in nitrogen starvation condition detected by RNAseq was confirmed by RT-qPCR (4.4 log2 x-fold down-regulation; Student’s t-test, p < 0.001; Fig. 3). LIPOR catalyzes the reductive formation of chlorophyllide from protochlorophyllide during the biosynthesis of chlorophylls^92^. Chlorophyll is a nitrogenous macromolecule, and reducing its synthesis reduces the nitrogen demand of the cells. Therefore, it is not surprising that LIPOR decreased. N starvation led to a modification of the photosynthetic apparatus also in the flagellates *Nannochloropsis gaditana* ^102^ and *Dunaliella tertiolecta* ^103^, and in the diatoms *Thalassiosira weissflogii* ^103^ and *T. pseudonana* ^99^. PER belongs to the highly expressed transcripts among the DEGs in nitrogen starvation RNAseq and also had 3.7 log2 x-fold up-regulation in RT-qPCR experiments (PER; Student’s t-test, p < 0.05; Fig. 3). However, the precise function of this permease is not known.

Regarding the type I PKS β-ketosynthase (KS) transcript, nitrogen starvation did not induce significant gene expression changes (Student’s t-test, p > 0.05; Fig. 3) in *A. carterae* indicating no changes in the possible synthesis of bioactives in nutrient starvation conditions. On the contrary, nutrient starvation increased bioactive compound production by other dinoflagellates. In particular, Hardison *et al*. (ref.^22^) found an increase in brevetoxin production by *Karenia brevis* in N-starvation culturing, and Frangópulos *et al*. (ref.^21^) showed different paralytic shellfish poisoning (PSP) toxin production (i.e. gonyautoxins, neosaxitoxins, decarbamoyl and sulphocarbamoyl toxins) by *Alexandrium minutum, Alexandrium andersoni*, and two clones of *Alexandrium tamarense* under both nitrogen- and phosphate- starvation conditions. A better understanding of nitrogen metabolism in dinoflagellates may help to fully utilize dinoflagellate biotechnological potential, predict blooms and limit the impact of harmful algal blooms, especially in a future anthropogenically altered aquatic environment.

### Phylogenesis of β-ketosynthase, L-asparaginase and cellulase and identification of conserved amino acids residues and signal peptides

The phylogenetic trees obtained from the analysis of orthologous sequences of each gene in different taxa were consistent with the expectation of their similarity with sequences from other related organisms (Supplementary Figs S1, S2 and S3). Indeed, in all the three phylogenetic trees, the sequences of *A. carterae* fell within the taxonomic group named SAR (Stramenopiles-Alveolates-Rhizaria). For KS, except for the uncertainties about the positions of *Vitrella brassicaformis* (SAR) and the two chlorophytes *Ostreococcus lucimarinus* and *Chlamydomonas reinhardtii* (bootstrap value lower than 50%, Supplementary Fig. S1), the sequence of *A. carterae* clustered with the other SAR members into a highly supported clade (bootstrap value > 85%, Supplementary Fig. S1). The lack of a clear separation of Chlorophyta from some SAR members was probably due to the low percentage of similarity among sequences (around 38%). However, our analysis confirmed the occurrence of KS domains in *A. carterae* and other dinoflagellates, as recently summarized by Kohli *et al*.^4^.

**Figure 4:**
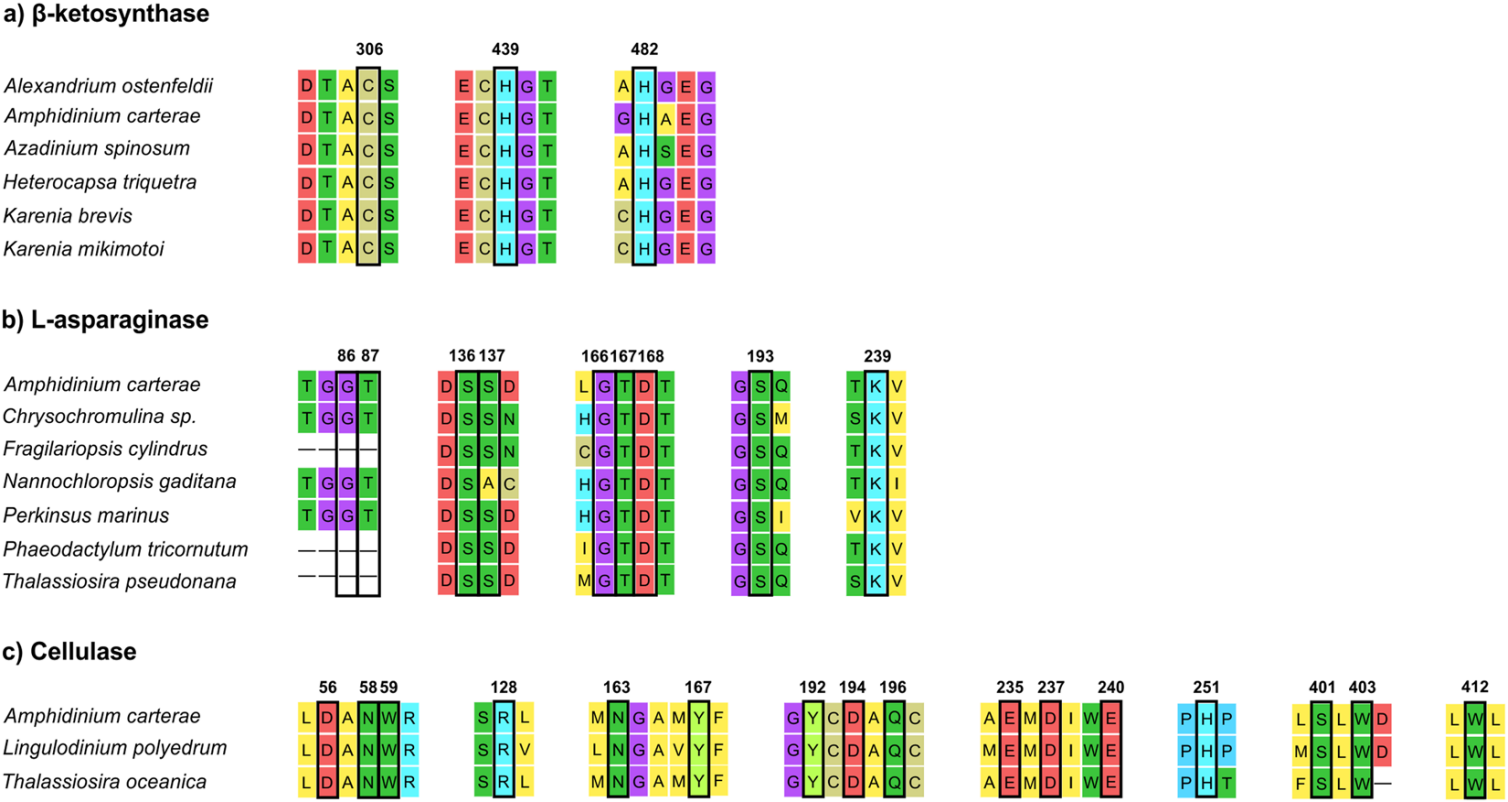
Conserved active sites of PKS β-ketosynthase (**a**), L-asparaginase (**b**) and cellulase (**c**) enzymes. Active site residues are highlighted in black boxes, and numbers above them refer to the *Amphidinium carterae* sequences of the current study. The accession numbers of the sequences used for this figures are reported in Supplementary Tables S1, S2 and S3 for β-ketosynthase, L-asparaginase and cellulase, respectively.

In the L-asparaginase tree (Supplementary Fig. S2) the sequence of *A. carterae* clustered together with other SAR members, with Stramenopiles as sister group (bootstrap value > 85%). This position in the phylogenetic tree reflected the high similarity among protein sequences (around 62%). Monophyly was also confirmed for other taxonomic groups as Bacteria, Animalia, Chlorophyta, Amebozoa, and Fungi (the latter with some exceptions, Supplementary Fig. S2). The cellulase phylogenetic tree showed a close similarity of our sequence from *A. carterae* with *Thalassiosira oceanica* (diatom, 54.1% of protein similarity) and *Lingulodinium polyedrum* (dinoflagellate, 57.7% of protein similarity) (Supplementary Fig. S3). We did not find any other cellulase sequence from microalgae. Since each of the genes of interest in *A. carterae* clustered together with the ones from other dinoflagellates or phylogenetically close taxa (e.g. diatoms and haptophytes) more than others (and these branches are highly supported) we looked deeply for conserved amino acids residues (Fig. 4).

For KS, the translated contig resulted in a 750 amino acids protein containing a β-ketosynthase domain (residues 135–555), with three conserved active sites having amino acids residues in the positions 306 (C), 439 (H), and 482 (H) (Fig. 4a). For L-asparaginase, we obtained a 444 amino acid sequence, containing both the N-terminal (76–285) and the C-terminal (286–417) portions. Nine conserved residues were found within the active site in the N-terminal half, with the two residues coding for threonine (positions 87 and 167, Fig. 4b) known to be involved in catalytic activity^104^.

The transcript coding for cellulase was translated in a 581 amino acid sequence, whose residues from 22 to 467 correspond to glycoside hydrolase family 7 and, in particular, to the concanavalin A-like lectin/glucanase domain. Sixteen conserved residues were found in the active site (Fig. 4c). The occurrence of a gene involved in the degradation of cellulose and related polysaccharides seems obvious in decomposers such as fungi and bacteria but not in dinoflagellates or diatoms. However, a recent study conducted in the dinoflagellate *Crypthecodinium cohnii* ^58^ showed that the coupling of cellulase expression and cell cycle progression regulate cell size in organisms with cell walls, and this may explain not only its occurrence in dinoflagellates, but also the high number of conserved amino acid residues in the active site of this enzyme. Blifernez-Klassen *et al*. (ref.^57^) also showed that the unicellular green microalga *Chlamydomonas reinhardtii* was able to use cellulose as an organic carbon source for heterotrophic growth, opening new perspectives for using cellulosic waste material for algal cultivation.

No signal peptides were found for KS and L-asparaginase transcripts. On the contrary, the analysis predicted a cleavage site for cellulase between amino acid positions 21 and 22 (Supplementary Fig. S6). C- and Y-scores in position 22 were 0.824 and 0.831, respectively. S-score in position 11 was 0.901 and mean S for positions 1–21 was 0.839. The analysis also yielded a D-score value (discrimination score, i.e. a weighted average of the mean S-score and the maximum Y-score) of 0.836. These findings imply that this protein can be secreted.

### Cytotoxicity

Cytotoxicity was analysed on human hepatocellular liver carcinoma (HepG2) cells to evaluate the potential toxic effect of the extracts. Hepatocytes are good models for studying toxicity since the liver is the primary site for drug metabolism and biotransformation^105,106^. *A. carterae* extracts did not show cytotoxicity on HepG2 cells after 24 h of 50 μg/mL extract exposure. Percentage of survival was still 100% after 24 h. *Amphidinium carterae* and/or *Amphidinium* spp. have been often associated to antimicrobial and haemolytic activities^28,30–32^. Cytotoxicity has not always been found. For instance, Shah *et al*. (ref.^26^) reported that RAW264.7 cell (Abelson murine leukemia virus-induced tumor cells) viability was still high (80%) after incubation for 24 h with 50 μg/mL of *A. carterae* methanol extracts. On the contrary, Samarakoon *et al*. (ref.^107^) found a reduction in cell viability of 60% after incubation for 24 h of 50 μg/mL chloroform extracts of *A. carterae*.

## Conclusion

In this study, we sequenced the full-transcriptome of the cosmopolitan dinoflagellate *A. carterae*, in both control and stressful (i.e. nitrogen starvation) conditions, in order to find transcripts for biotechnologically interesting enzymes. We identified for the first time in this species L-asparaginase and cellulase. L-asparaginase has applications for the treatment of acute lymphoblastic leukemia^43^, acute myeloid leukemia^44^, and non-Hodgkin’s lymphoma^45^, and for reduction of acrylamide in food industries^48–50^, while cellulase has applications for biofuel production and other industrial sectors^54–56^. Both enzymes have not been studied before in this species and L-asparaginase has not been studied before also in other dinoflagellates. In addition, we identified the transcript for the β-ketosynthase, an enzyme commonly found in dinoflagellates but not found in all *Amphidinium* clones^4^. This enzyme can be involved in the synthesis of bioactive compounds with antipredator and allelopathic activities, as well as anticancer, antifungal and/or beneficial effects for the treatment of Alzheimer’s disease^4,12,14,29,33,34^. We also tried to identify and annotate all the other possible PKS domains. In fact, we found 270 contigs blasted for acyltransferase, 37 for acyl carrier protein, 5 for β-ketoacyl reductase, 3 for enoyl reductase, 3304 for methyl transferases, 236 for thioesterases and 1247 for dehydrogenase domains. Considering the multiple reactions in which these enzymes may be involved, unfortunately we were not able to relate these contigs to PKS activity. In addition, the negative response for cytotoxicity indicated that this strain probably does not produce cytotoxic compounds. Production of interesting enzymes from microalgae is receiving increasing attention due to their low cost of production, easy cultivation and harvesting at large scales, cheaper and easier extraction, and higher yields and purification of protein and enzymes by simple methods^10,50,52^. These new findings open other possible applications for dinoflagellates in the blue biotechnology sector. Recent advances in genomics, metagenomics, proteomics, screening methods, expression systems, bioinformatics, and the ever-increasing availability of sequenced genomes and transcriptomes will further advance the microalgal biotechnology field by providing new opportunities for the discovery of novel enzymes and bioactive compounds.

## Electronic supplementary material

Supplementary Information
