# Supplementary material for: De novo transcriptome of the cosmopolitan dinoflagellate Amphidinium carterae to identify enzymes with biotechnological potential
Source: Sci Rep. 2017 Sep 15;7:11701. doi: 10.1038/s41598-017-12092-1 (PMC5601461; doi:10.1038/s41598-017-12092-1)
Supplement: Supplementary file 1 — Supplementary Information [file 41598_2017_12092_MOESM1_ESM.pdf]

Supplementary Information

De novo transcriptome of the cosmopolitan dinoflagellate *Amphidinium carterae* to identify enzymes with biotechnological potential

Lauritano Chiara, De Luca Daniele, Ferrarini Alberto, Avanzato Carla, Minio Andrea, Esposito Francesco, Ianora Adrianna

Supplementary Figure S1. Maximum likelihood tree for β-ketosynthase gene.
Colours on the branches refer to the taxonomic group as follows:
Amebozoa, Animalia, Chlorophyta, Fungi, and SAR (Stramenopiles-Alveolates-Rhizaria).
Colours in the circles indicate the bootstrap support to each node (< 60% in red, < 82% in green, and > 87% in blue), with colour intensity increasing with bootstrap value.

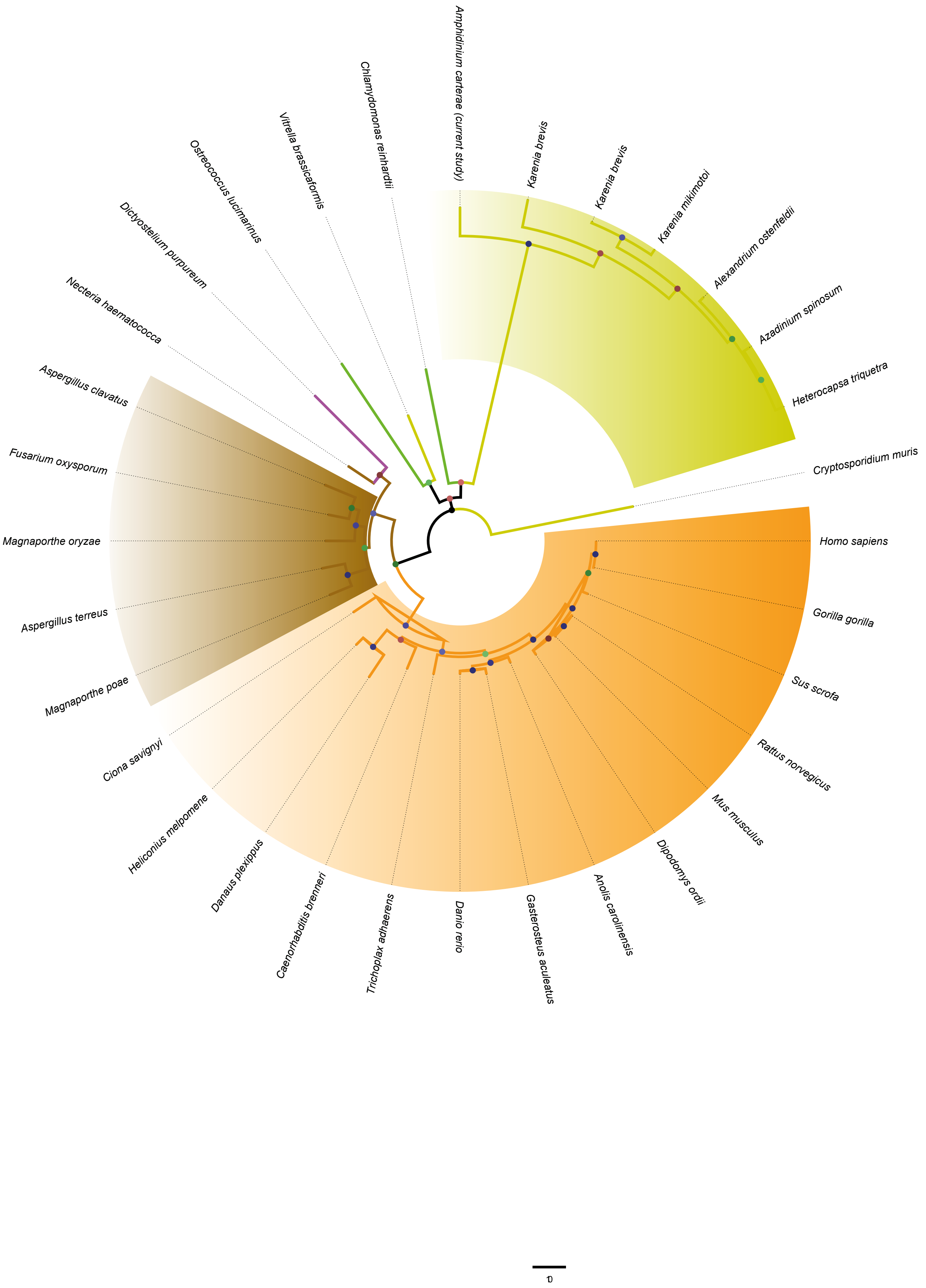

Supplementary Figure S2. Maximum likelihood tree for L-asparaginase gene. Colours on the branches refer to the taxonomic group as follows: Amebozoa, Animalia, Bacteria, Chlorophyta, Fungi, and SAR (Stramenopiles-Alveolates-Rhizaria).
Colours in the circles indicate the bootstrap support to each node (< 51% in red, < 83% in green, and > 85% in blue), with colour intensity increasing with bootstrap value.

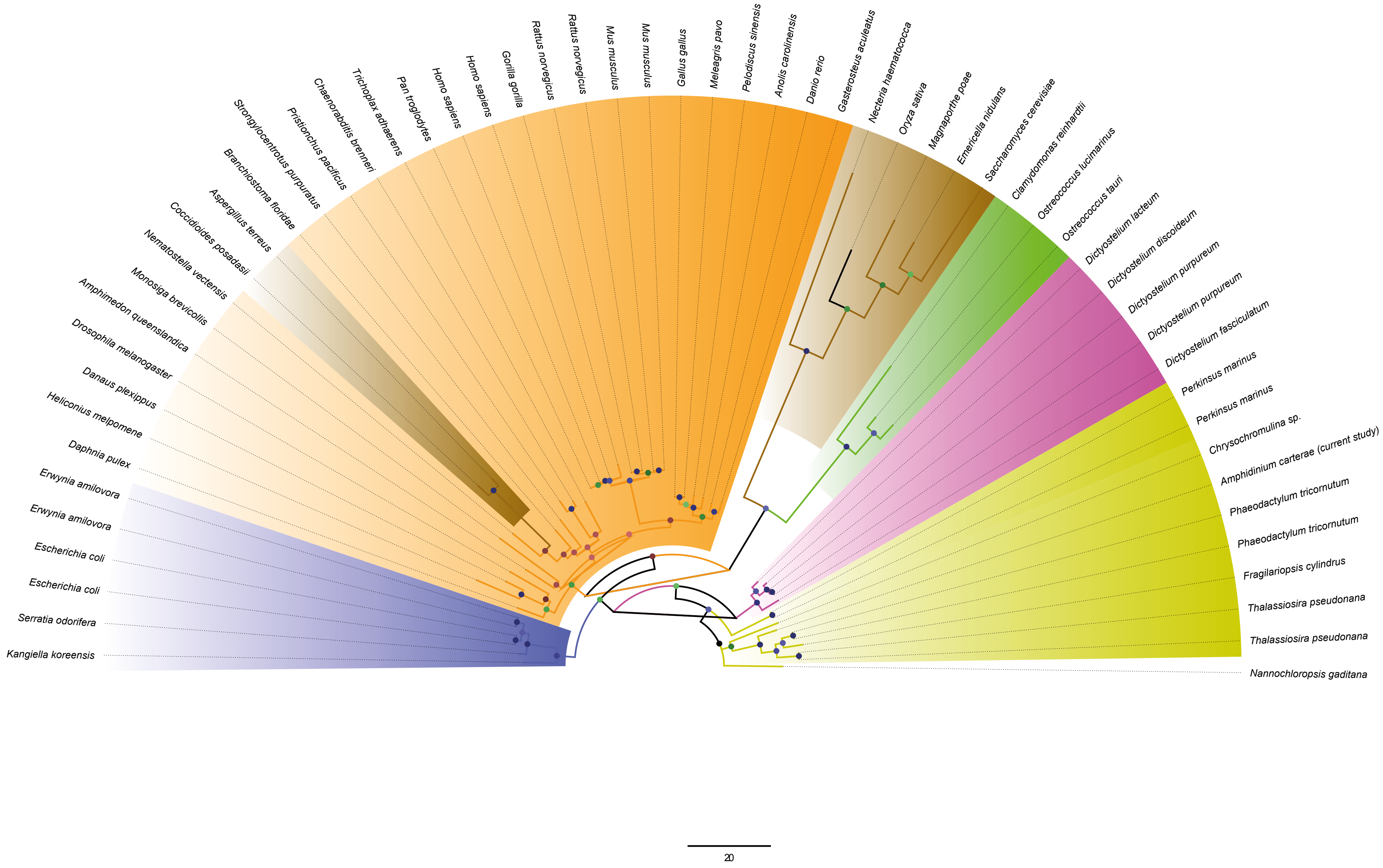

Supplementary Figure S3. Maximum likelihood tree for cellulase gene. Colours on the branches refer to the taxonomic group as follows: Amebozoa, Animalia, Fungi, and SAR (Stramenopiles-Alveolates-Rhizaria).
Colours in the circles indicate the bootstrap support to each node (< 54% in red, < 84% in green, and > 89% in blue), with colour intensity increasing with bootstrap value.

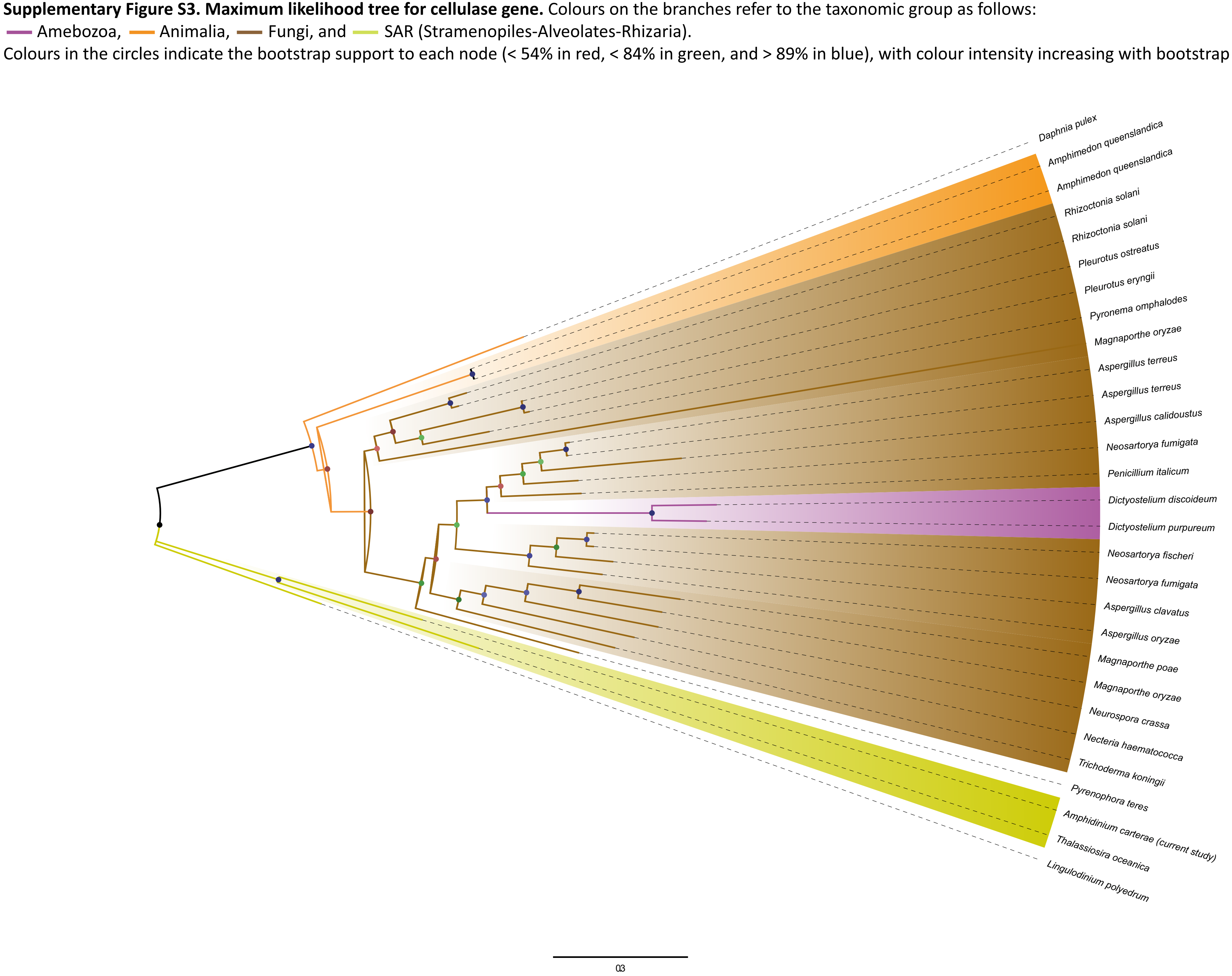

Supplementary Figure S4. Combined *de novo* transcriptome assembly workflow. The number of sequences produced by each step is reported in the boxes .

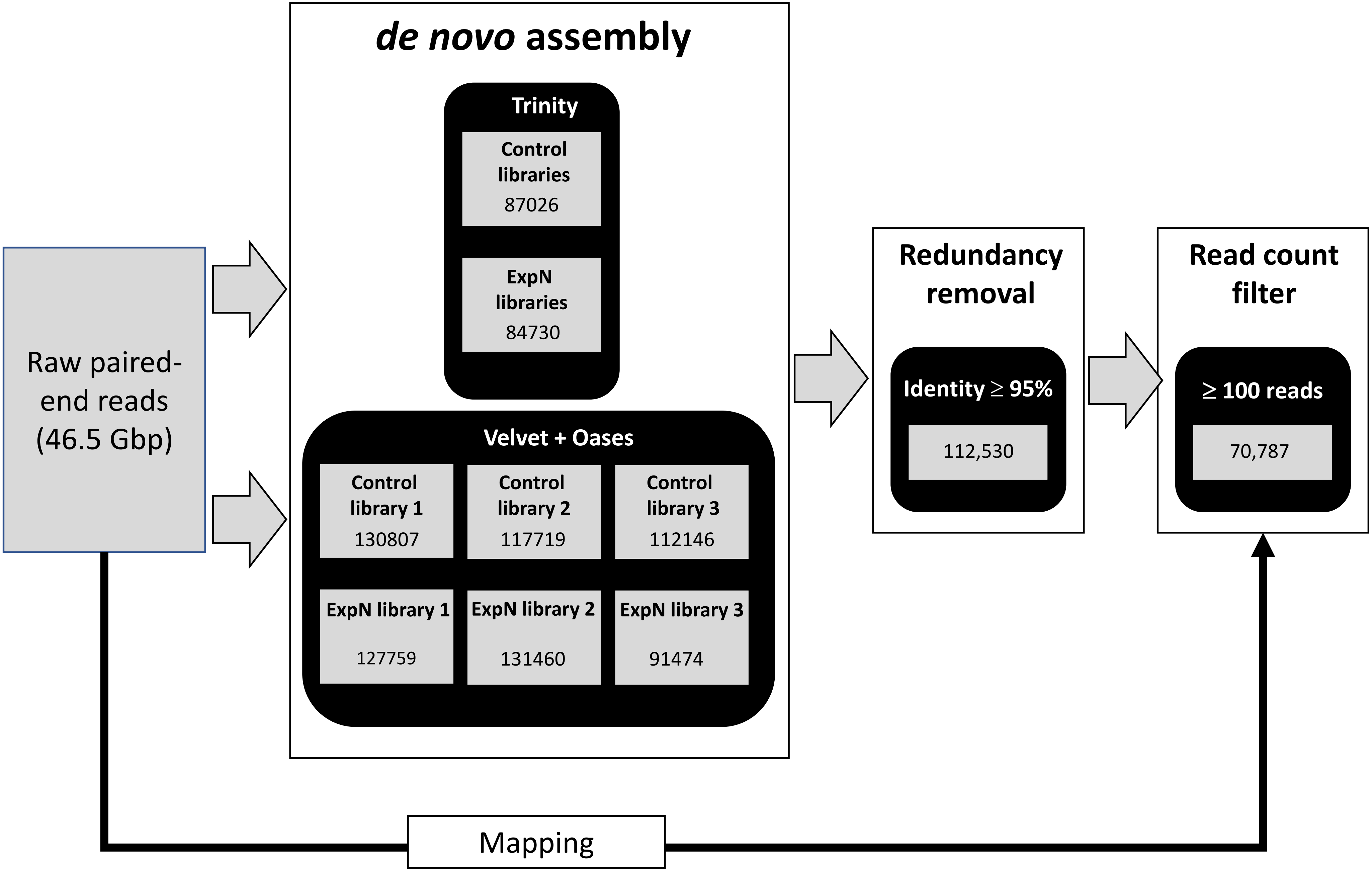

Supplementary Figure S5. Reference gene assessment. Ranking of the best reference genes (RGs). a) The NormFinder algorithm ranks the candidate RGs according to their expression stability. Lower stability values indicate more-stable genes. b) The stepwise exclusion of more-variable genes using the geNorm program. More stable genes with the lowest average expression stability value are indicated by the arrow (Vandesompele et al., 2002). c) Ranking of the best RGs based on standard deviation of the Ct values given by BestKeeper. Abbreviations used are: Ubiquitin (UB), cyclin-dependent kinase a-1 (CDK), alpha- and beta-tubulins (ATUB and BTUB, respectively) and glyceraldehyde-3-phosphate dehydrogenase (GAPDH).

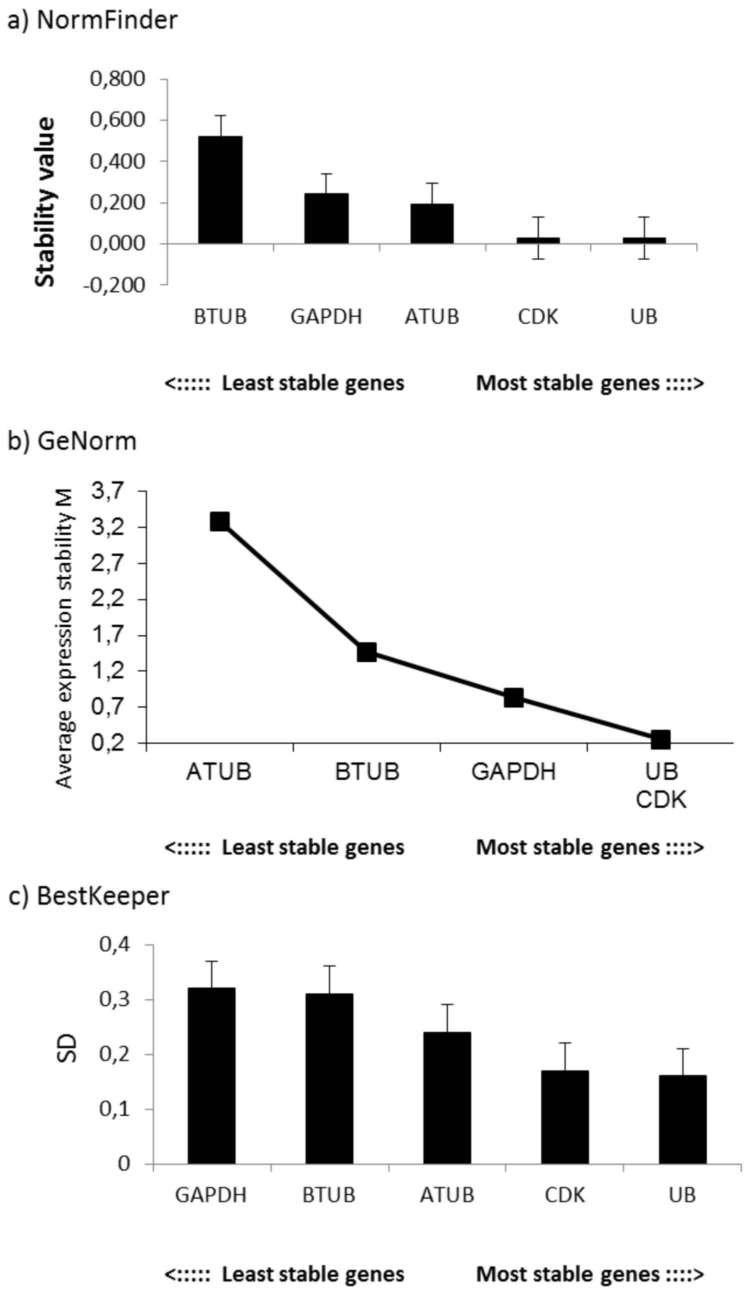

Supplementary Figure S6. SignalP V4.1 graphic output for cellulase. The values of the C-score (raw cleavage site score), S-score (signal peptide score) and Y-score (combination of the C- and S-scores, resulting in a better cleavage site prediction) are shown for each position in the sequence. The C-score is defined to be high for the position immediately after the cleavage site, i.e. the first position in the mature protein.

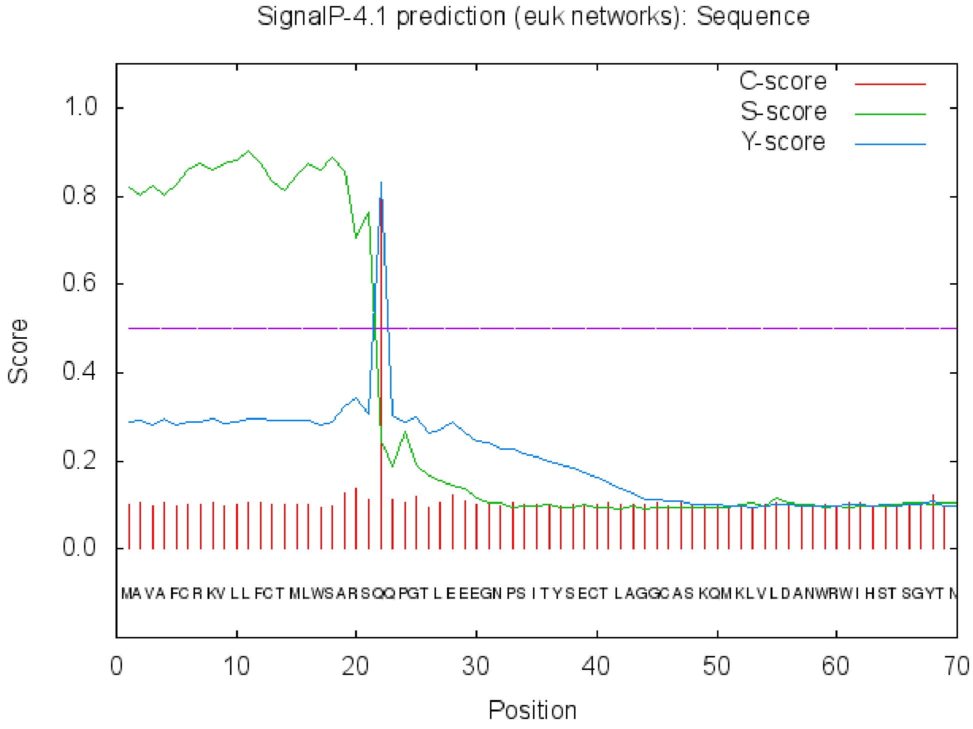

Supplementary Table S1. List of the sequences retrieved from public repositories for β-ketosynthase

Species	Database	Accession number
Alexandrium ostenfeldii	UniProtKB	K7WCI2
Anolis carolinensis	EggNOG 4.5.1	ENSACAP00000009733
Aspergillus clavatus	EggNOG 4.5.1	CADACLAP00000068
Aspergillus terreus	EggNOG 4.5.1	CADATEAP00000507
Azadinium spinosum	UniProtKB	A0A0A0V936
Caenorhabditis brenneri	EggNOG 4.5.1	CBN05956
Chlamydomonas reinhardtii	EggNOG 4.5.1	EDP07994
Ciona savignyi	EggNOG 4.5.1	ENSCSAVP00000011720
Cryptosporidium muris	EggNOG 4.5.1	XP_002140396.1
Danaus plexippus	EggNOG 4.5.1	EHJ69181
Danio rerio	EggNOG 4.5.1	ENSDARP00000098122
Dictyostelium purpureum	EggNOG 4.5.1	XP_003282946.1
Dipodomys ordii	EggNOG 4.5.1	ENSDORP00000004029
Fusarium oxysporum	EggNOG 4.5.1	FOXG_02741P0
Gasterosteus aculeatus	EggNOG 4.5.1	ENSGACP00000002882
Gorilla gorilla	EggNOG 4.5.1	ENSGGOP00000011384
Heliconius melpomene	EggNOG 4.5.1	HMEL004144-PA
Heterocapsa triquetra	UniProtKB	K7W9M8
Homo sapiens	EggNOG 4.5.1	ENSP00000304592
Karenia brevis	UniProtKB	A7KH55
Karenia brevis	UniProtKB	A7KH58
Karenia mikimotoi	UniProtKB	A0A0P0YL85
Magnaporthe oryzae	EggNOG 4.5.1	MGG_00233T0
Magnaporthe poae	EggNOG 4.5.1	MAPG_01087T0
Mus musculus	EggNOG 4.5.1	ENSMUSP00000052872
Necteria haematococca	EggNOG 4.5.1	NechaP106233
Ostreococcus lucimarinus	EggNOG 4.5.1	A4RSV8
Rattus norvegicus	EggNOG 4.5.1	ENSRNOP00000064445
Sus scrofa	EggNOG 4.5.1	ENSSSCP00000021647
Trichoplax adhaerens	EggNOG 4.5.1	TriadP31339
Vitrella brassicaformis	UniProtKB	A0A0G4GSR5

Supplementary Table S2. List of the sequences retrieved from public repositories for L-asparaginase

**Species**	**Database**	**Accession number**
Amphimedon queenslandica	EggNOG 4.5.1	PAC_15705820
Anolis carolinensis	EggNOG 4.5.1	ENSACAP00000010777
Aspergillus terreus	EggNOG 4.5.1	CADATEAP00002702
Branchiostoma floridae	EggNOG 4.5.1	JGI278040
Caenorhabditis brenneri	EggNOG 4.5.1	CBN28810
Chlamydomonas reinhardtii	EggNOG 4.5.1	EDP09027
Chrysochromulina sp.	UniProtKB	A0A0M0LS84
Coccidioides posadasii	EggNOG 4.5.1	XP_003069781.1
Danaus plexippus	EggNOG 4.5.1	EHJ67811
Danio rerio	EggNOG 4.5.1	ENSDARP00000103166
Daphnia pulex	EggNOG 4.5.1	DappuP300337
Dictyostelium discoideum	EggNOG 4.5.1	DDB0201640
Dictyostelium fasciculatum	UniProtKB	F4PK88
Dictyostelium lacteum	UniProtKB	A0A151Z2Z6
Dictyostelium purpureum	UniProtKB	F0ZKN3
Dictyostelium purpureum	EggNOG 4.5.1	XP_003287968.1
Drosophila melanogaster	EggNOG 4.5.1	FBpp0081620
Emericella nidulans	EggNOG 4.5.1	CADANIAP00002415
Erwinia amylovora	UniProtKB	D4I3Y5
Erwinia amylovora	UniProtKB	E5B626
Escherichia coli	UniProtKB	P0A962
Escherichia coli	UniProtKB	P0A963
Fragilariopsis cylindrus	UniProtKB	A0A1E7F9C6
Gallus gallus	EggNOG 4.5.1	ENSGALP00000018854
Gasterosteus aculeatus	EggNOG 4.5.1	ENSGACP00000010569
Gorilla gorilla	EggNOG 4.5.1	ENSGGOP00000003182
Heliconius melpomene	EggNOG 4.5.1	HMEL010500-PA
Homo sapiens	UniProtKB	Q86U10
Homo sapiens	EggNOG 4.5.1	ENSP00000450040
Kangiella koreensis	UniProtKB	C7RBC6
Magnaporthe poae	EggNOG 4.5.1	MAPG_02287T0
Meleagris gallopavo	EggNOG 4.5.1	ENSMGAP00000014197
Monosiga brevicollis	EggNOG 4.5.1	XP_001747607.1
Mus musculus	UniProtKB	A0JNU3
Mus musculus	EggNOG 4.5.1	ENSMUSP00000078369
Nannochloropsis gaditana	UniProtKB	W7U8P8
Necteria haematococca	EggNOG 4.5.1	NechaP55158
Nematostella vectensis	EggNOG 4.5.1	NEMVEDRAFT_v1g10699-PA
Oryza sativa	EggNOG 4.5.1	BGIOSGA039015-PA
Ostreococcus lucimarinus	EggNOG 4.5.1	A4S6P8
Ostreococcus tauri	EggNOG 4.5.1	Q00WU7
Pan troglodytes	EggNOG 4.5.1	ENSPTRP00000011549
Pelodiscus sinensis	EggNOG 4.5.1	ENSPSIP00000014276

Perkinsus marinus	UniProtKB	C5LA22
Perkinsus marinus	EggNOG 4.5.1	XP_002765048.1
Phaeodactylum tricornutum	UniProtKB	B7G1S2
Phaeodactylum tricornutum	EggNOG 4.5.1	Phatr20905
Pristionchus pacificus	EggNOG 4.5.1	PPA26441
Rattus norvegicus	UniProtKB	O88202
Rattus norvegicus	EggNOG 4.5.1	ENSRNOP00000017454
Saccharomyces cerevisiae	EggNOG 4.5.1	YDR321W
Serratia odorifera	UniProtKB	D4E5P7
Strongylocentrotus purpuratus	EggNOG 4.5.1	SPU_007448tr
Thalassiosira pseudonana	UniProtKB	B5YNE5
Thalassiosira pseudonana	EggNOG 4.5.1	Thaps7120
Trichoplax adhaerens	EggNOG 4.5.1	TriadP30040

Supplementary Table S3. List of the sequences retrieved from public repositories for cellulase

**Species**	**Database**	**Accession number**
Amphimedon queenslandica	EggNOG 4.5.1	PAC_15719652
Amphimedon queenslandica	UniProtKB	I1FU52
Aspergillus calidoustus	UniProtKB	A0A0U5G5A8
Aspergillus clavatus	UniProtKB	A1CE97
Aspergillus oryzae	UniProtKB	Q2UBM3
Aspergillus terreus	EggNOG 4.5.1	CADATEAP00005220
Aspergillus terreus	UniProtKB	Q5G1M0
Daphnia pulex	EggNOG 4.5.1	DappuP300366
Dictyostelium discoideum	EggNOG 4.5.1	DDB0302429
Dictyostelium purpureum	EggNOG 4.5.1	XP_003287745.1
Lingulodinium polyedrum	UniProtKB	D8UXL7
Magnaporthe oryzae	EggNOG 4.5.1	MGG_06834T0
Magnaporthe oryzae	UniProtKB	G4NK46
Magnaporthe poae	EggNOG 4.5.1	MAPG_04437T0
Necteria haematococca	EggNOG 4.5.1	NechaP52498
Neosartorya fischeri	UniProtKB	A1DMA5
Neosartorya fumigata	UniProtKB	B0Y793
Neosartorya fumigata	UniProtKB	B0Y8K2
Neurospora crassa	UniProtKB	Q7SA23
Penicillium italicum	UniProtKB	A0A0A2L6R7
Pleurotus eryngii	UniProtKB	A0A0D6A8W8
Pleurotus ostreatus	UniProtKB	A0A067NLE5
Pyrenophora teres f. teres	EggNOG 4.5.1	XP_003301114.1
Pyronema omphalodes	UniProtKB	U4L139
Rhizoctonia solani	UniProtKB	A0A0K6GD52
Rhizoctonia solani	UniProtKB	A0A0A1UL53
Thalassiosira oceanica	UniProtKB	K0T5R6
Trichoderma koningii	UniProtKB	P62695

Supplementary Table S4. List of selected reference genes (RG) and genes of interest (GOI) with their abbreviations (Abbr), functions, primer's sequences and efficiencies.

Gene	Abbr	Function	Primer F	Primer R	E
Reference genes (RG)					
Alpha tubulin	ATUB	Microtubule subunit	AACTCATCACCGTAGCCCTC	ATGTACTCCAAGCGTGCCTT	1.99
Beta tubulin	BTUB	Microtubule subunit	TTCCTCATCGAACGCACCTT	CAATGATGATGCGCCACTCC	2
Ubiquitin	UB	Post-translational modification	GGAATTCCCTCTTTGTCCTGGA	GCTCAAGGTCGGTAGGGTC	2
Cyclin-dependent kinase 3	CDK3	Cell cycle regulation	TCCCAATGACCACACATCGA	ATTGAAGCTGGCGGACTTTG	2
Glyceraldehyde 3-phosphate dehydrogenase	GAPDH	Glycolysis	GGAGAAGGGGCAAGAGACAA	GTGCCTTCCAGTGCTCAATG	1.97
Genes of interest (GOI)					
Heat shock protein 70	HSP70	Stress response	ATGCGATCGATCTCTGCCTG	CGCGCAATGACAAAGGACAA	2
Heat shock protein 90	HSP90	Stress response	GATCTCGAGCCCTTCCTTGG	GCCCAGGTGTCATCTTCTCC	2
Nitrate transporter	NATETR	Transporter	GGTAGCACCCACTGTTCCAA	TTGACAGATGGAGGTGTGGC	2
Nitrate reductase	NATE	Reduces nitrate in nitrite	CTCCGAGGCTTGTTGTGGAT	AAGATCGACGGCCAAGTTGT	2
Nitrite reductase	NITE	Reduces nitrite in different products	CCAAGCCAGCATCAACCAAC	TGGCCAACATCCAGAAGACC	1.99
Histidine ammonia-lyase, histidase	HAL	Histidine catabolism	ACCTAGTCTGCCACCAAGAC	TCCCAACTCCCAAGGTCATC	2
3-hydroxyacyl-CoA dehydrogenase	HADH	Valine, leucine and isoleucine degradation	AACGGGGTTGCTATCCATCA	AGGGAAACATGATGCTTGCG	2
Malate dehydrogenase	MDH	Citrate cycle (TCA cycle)	GCTGTGCTCGTTCTGTGAAA	ATGCAGTTGTGGCGTTGATT	2
Polypeptide n-acetylgalactosaminyltransferase 1	GALNT1	Oligosaccharide biosynthesis	ATGTCCCAAGAGACGAAGCA	GCGCTAAGAATACTGGTGGC	2
Phosphoglycerate kinase	PGK	Glycolysis / Gluconeogenesis	CTGATCGCCAACATCGCTAC	ATCGGTGGTGGTGATTCAGT	2
Phospholipase D1	PLD1	Glycerophospholipid metabolism	TTTCTGTTGGGCTGTGCTTC	TCCAACAAAGAATGCCCAGC	2
Light-independent protochlorophyllide reductase	LIPOR	Chlorophyll biosynthetic process	TCCAAGAGACCGCAAGCTTA	TGAGTTGCGCACAGAAGTTT	2
Permease	PER	Membrane transport protein	CTTGAAGACGAATCGCCTGG	TTTGAGGGCTTGGTCTTTGC	2
β-ketosynthase (Type I PKS β-ketoacyl synthase)	KS	Polyketide synthesis	GGTATCGCTTGCCAGGATCA	AGGTTGAATGTCTGCTGGCA	2

Supplementary Table S5. Anderson-Darling normality test results and p values, both normal and Monte Carlo.

	HSP90	HSP70	NATE	NITE	NATETR	KS	LIPOR	PER	MDH	PGK	PLD1	HAL
Anderson-Darling A	0,512	0,5714	0,287	0,2881	0,363	0,4114	0,2278	0,4305	0,5523	0,2552	0,3435	0,3021
p (normal)	0,09793	0,06444	0,4584	0,4552	0,2759	0,197	0,5754	0,1721	0,0559	0,5405	0,315	0,36
p (Monte Carlo)	0,1012	0,0603	0,5238	0,5214	0,3113	0,2233	0,7305	0,1905	0,0526	0,6418	0,3558	0,4501

	GALNT1	HADH
Anderson-Darling A	0,171	0,3349
p (normal)	0,8561	0,284
p (Monte Carlo)	0,9317	0,3474

Supplementary Table S6: Statistics of assemblies produced by trinity and oases assemblers

Replicates used	all	all	1	2	3	1	2	3	
Condition	control	expN	control	control	control	expN	expN	expN	
Assembler	trinity	trinity	oases	oases	oases	oases	oases	oases	trinity + oases + cd-hit
Number of contigs	87026	84730	130807	117719	112146	127759	131460	91474	112530
Total assembly length	101129093	99099585	168493401	154063973	147864097	162921637	169418069	120536365	160504075
Min length (bp)	201	201	200	200	200	200	200	200	121
Mean length (bp)	1162	1169	1288	1308	1318	1275	1288	1317	1426
Max length (bp)	21702	21725	21769	17175	18612	19077	21257	15252	21769
N50	1813	1805	1883	1894	1896	1885	1872	1865	1979
N90	530	547	632	649	666	616	633	677	750

Supplementary Table S7. List of KEGG pathways associated to contigs with assigned enzyme codes in the full transcriptome. The table contains pathway names, pathway ID, number of enzymes found for each pathway, enzyme codes and sequence names.

Pathway	Pathway ID	#Enzs in Pathway	Enzyme	Seqs per Enzyme	Seqs ID
Biosynthesis of antibiotics	map01130	135	ec:2.6.1.11 - transaminase, ec:6.3.3.1 - cyclo-ligase, ec:6.4.1.2 - carboxylase, ec:2.6.1.16 - transaminase (isomerizing), ec:2.7.1.90 - 1-phosphotransferase, ec:2.7.4.6 - kinase, ec:2.7.4.3 - kinase, ec:4.1.1.48 - synthase, ec:2.7.4.2 - kinase, ec:4.1.1.49 - carboxykinase (ATP), ec:4.1.1.39 - carboxylase, ec:1.3.5.1 - dehydrogenase, ec:1.4.3.3 - oxidase, ec:4.1.1.32 - carboxykinase (GTP), ec:4.1.1.20 - decarboxylase, ec:1.17.7.1 - synthase (ferredoxin), ec:2.6.1.52 - transaminase, ec:2.2.1.1 - glycolaldehydetransferase, ec:2.2.1.6 - synthase, ec:2.2.1.7 - synthase, ec:2.7.2.4 - kinase, ec:2.7.2.3 - kinase, ec:4.3.2.1 - lyase, ec:4.3.2.2 - lyase, ec:1.2.1.38 - reductase, ec:2.7.1.40 - kinase, ec:3.5.4.6 - deaminase, ec:2.6.1.42 - transaminase, ec:1.2.1.59 - dehydrogenase (NAD(P)+) (phosphorylating), ec:2.7.1.11 - phosphohexokinase, ec:4.2.1.51 - dehydratase, ec:2.5.1.10 - diphosphate synthase, ec:6.2.1.5 - ligase (ADP-forming), ec:2.5.1.19 - 1-carboxyvinyltransferase, ec:6.2.1.4 - ligase (GDP-forming), ec:6.2.1.1 - ligase, ec:2.5.1.21 - synthase, ec:2.5.1.29 - diphosphate synthase, ec:1.1.1.133 - reductase, ec:1.2.1.11 - dehydrogenase, ec:1.2.1.12 - dehydrogenase	4, 4, 16, 1, 1, 20, 10, 1, 1, 5, 95, 11, 1, 5, 2, 2, 4, 24, 1, 1, 7, 41, 1, 1, 4, 25, 3, 6, 16, 5, 1, 1, 28, 1, 6, 1, 1, 1, 2, 4, 45, 1, 1, 1, 4, 3, 2, 4, 1, 9, 2, 2, 2, 2, 1, 2, 10, 1, 4, 1, 2, 2, 1, 1, 5, 2, 2, 1, 7, 3, 1, 4, 1, 31, 1, 1, 11, 6, 6, 1, 4, 8, 1, 9, 3, 38, 1, 2, 8, 2, 1, 1, 5, 2, 5, 3, 4, 8, 7, 5, 10, 4, 4, 2, 5, 1, 2, 1, 1, 2, 8, 2, 5, 23, 2, 1, 1, 7, 15, 10, 11, 2, 1, 24, 16, 10, 10, 6, 7, 1, 2, 1, 8, 1, 1	vo_c3_Locus_29526_Transcript_7/10_Confidence_0.268_Length_874, vo_n2_Locus_6432_Transcript_1/2_Confidence_0.667_Length_1653, vo_n2_Locus_3256_Transcript_3/4_Confidence_0.250_Length_4070, vo_c1_Locus_5351_Transcript_2/2_Confidence_0.333_Length_1693, vo_c2_Locus_14099_Transcript_8/21_Confidence_0.413_Length_6648, vo_c1_Locus_1785_Transcript_2/4_Confidence_0.667_Length_6496, vo_c2_Locus_14099_Transcript_2/21_Confidence_0.444_Length_6577, vo_c3_Locus_4727_Transcript_17/25_Confidence_0.456_Length_6641, vo_n3_Locus_484_Transcript_6/12_Confidence_0.250_Length_1533, vo_c3_Locus_17332_Transcript_1/1_Confidence_0.333_Length_6493, vo_n3_Locus_696_Transcript_20/35_Confidence_0.473_Length_5396, vo_c3_Locus_2164_Transcript_38/39_Confidence_0.292_Length_6879, tri_n_comp41552_c0_seq1, vo_c2_Locus_1509_Transcript_1/47_Confidence_0.149_Length_2731, vo_c2_Locus_2193_Transcript_1/3_Confidence_0.667_Length_1218, vo_c1_Locus_860_Transcript_10/29_Confidence_0.571_Length_6744, vo_c2_Locus_1509_Transcript_11/47_Confidence_0.164_Length_2686, vo_n2_Locus_1751_Transcript_51/59_Confidence_0.207_Length_3566, vo_n2_Locus_15097_Transcript_1/1_Confidence_0.000_Length_3935, vo_n3_Locus_484_Transcript_5/12_Confidence_0.250_Length_1451, vo_c1_Locus_860_Transcript_11/29_Confidence_0.571_Length_6741, tri_c_comp19913_c0_seq3, vo_n2_Locus_1751_Transcript_37/59_Confidence_0.195_Length_3437, vo_c2_Locus_1509_Transcript_6/47_Confidence_0.149_Length_2592, vo_n2_Locus_1408_Transcript_4/5_Confidence_0.222_Length_11768, vo_c2_Locus_6348_Transcript_1/2_Confidence_1.000_Length_3755, vo_c1_Locus_13409_Transcript_2/6_Confidence_0.286_Length_871, vo_c3_Locus_2169_Transcript_1/3_Confidence_0.167_Length_848, vo_n2_Locus_739_Transcript_8/8_Confidence_0.167_Length_1338, vo_n3_Locus_4095_Transcript_2/3_Confidence_0.444_Length_3401, vo_c1_Locus_12423_Transcript_2/2_Confidence_0.333_Length_980, vo_c1_Locus_25242_Transcript_1/1_Confidence_0.000_Length_1465, vo_c1_Locus_4920_Transcript_1/2_Confidence_0.714_Length_1520, tri_c_comp49412_c0_seq1, vo_n2_Locus_5279_Transcript_1/1_Confidence_0.333_Length_2493, vo_n2_Locus_20723_Transcript_1/1_Confidence_0.000_Length_1179, vo_n3_Locus_24634_Transcript_1/1_Confidence_0.000_Length_445, vo_n1_Locus_5513_Transcript_4/4_Confidence_0.286_Length_1053, tri_c_comp24673_c0_seq1, tri_c_comp5012_c0_seq2, vo_c3_Locus_3080_Transcript_1/28_Confidence_0.009_Length_1584, vo_n1_Locus_9563_Transcript_1/1_Confidence_0.000_Length_1437, vo_n2_Locus_26604_Transcript_1/2_Confidence_0.750_Length_1361, vo_n2_Locus_2689_Transcript_2/2_Confidence_0.000_Length_2612, vo_c2_Locus_4541_Transcript_2/6_Confidence_0.571_Length_909, vo_n2_Locus_26604_Transcript_2/2_Confidence_0.000_Length_1685, vo_c2_Locus_10361_Transcript_6/7_Confidence_0.250_Length_1220, tri_c_comp24673_c0_seq1, vo_n1_Locus_2451_Transcript_1/3_Confidence_0.600_Length_2348, vo_c3_Locus_4102_Transcript_1/1_Confidence_0.000_Length_1034, vo_n2_Locus_22654_Transcript_1/1_Confidence_0.000_Length_691, vo_n1_Locus_2897_Transcript_2/14_Confidence_0.160_Length_971, vo_n2_Locus_14542_Transcript_1/3_Confidence_0.200_Length_924, vo_n3_Locus_12191_Transcript_1/1_Confidence_0.000_Length_1671, vo_n2_Locus_5279_Transcript_1/1_Confidence_0.333_Length_2493, vo_n3_Locus_24634_Transcript_1/1_Confidence_0.000_Length_445, vo_c3_Locus_25814_Transcript_1/1_Confidence_0.000_Length_1912, vo_n2_Locus_13158_Transcript_1/2_Confidence_1.000_Length_1003, vo_c1_Locus_2295_Transcript_9/10_Confidence_0.238_Length_2213, vo_c2_Locus_2862_Transcript_9/10_Confidence_0.250_Length_2209, vo_c1_Locus_2295_Transcript_3/10_Confidence_0.524_Length_2330, vo_n2_Locus_1690_Transcript_8/11_Confidence_0.429_Length_2234, vo_n2_Locus_1690_Transcript_9/11_Confidence_0.429_Length_2277, vo_c1_Locus_1636_Transcript_17/122_Confidence_0.157_Length_2013, vo_n3_Locus_37_Transcript_16/46_Confidence_0.281_Length_1685, vo_n2_Locus_3_Transcript_22527/24291_Confidence_0.000_Length_1609, vo_n3_Locus_37_Transcript_10/46_Confidence_0.207_Length_1727, vo_n3_Locus_37_Transcript_9/46_Confidence_0.215_Length_1812, vo_c1_Locus_1636_Transcript_122/122_Confidence_0.108_Length_2335, vo_n2_Locus_3_Transcript_22541/24291_Confidence_0.000_Length_288, vo_n2_Locus_3_Transcript_22550/24291_Confidence_0.000_Length_1791, vo_n3_Locus_37_Transcript_33/46_Confidence_0.163_Length_732, vo_n2_Locus_3_Transcript_22526/24291_Confidence_0.000_Length_620, vo_n2_Locus_3_Transcript_22511/24291_Confidence_0.000_Length_798, vo_c1_Locus_1636_Transcript_15/122_Confidence_0.065_Length_512, vo_c2_Locus_52_Transcript_144/466_Confidence_0.025_Length_2131, tri_n_comp20144_c0_seq14, vo_c2_Locus_52_Transcript_133/466_Confidence_0.023_Length_2364, tri_n_comp20144_c0_seq13, vo_n3_Locus_41731_Transcript_1/1_Confidence_0.500_Length_459, vo_c2_Locus_52_Transcript_132/466_Confidence_0.016_Length_1024, vo_n1_Locus_55_Transcript_52/66_Confidence_0.069_Length_313, tri_c_comp20343_c0_seq9, tri_n_comp20144_c0_seq10, tri_n_comp20144_c0_seq7, tri_n_comp20144_c0_seq6, vo_c3_Locus_31_Transcript_6/79_Confidence_0.152_Length_709, tri_n_comp20144_c0_seq3, tri_n_comp20144_c0_seq4, tri_n_comp20144_c0_seq1, vo_c1_Locus_1636_Transcript_86/122_Confidence_0.182_Length_2244, vo_c1_Locus_1636_Transcript_73/122_Confidence_0.222_Length_1747, vo_c2_Locus_52_Transcript_164/466_Confidence_0.011_Length_2810, vo_n2_Locus_3_Transcript_22535/24291_Confidence_0.000_Length_656, vo_n2_Locus_3_Transcript_22546/24291_Confidence_0.000_Length_1498, vo_c3_Locus_31_Transcript_74/79_Confidence_0.124_Length_2769, vo_c3_Locus_31_Transcript_68/79_Confidence_0.124_Length_1224, vo_n3_Locus_37_Transcript_21/46_Confidence_0.148_Length_1139, vo_c3_Locus_31_Transcript_67/79_Confidence_0.152_Length_2229, vo_n3_Locus_37_Transcript_14/46_Confidence_0.259_Length_5942, vo_n1_Locus_55_Transcript_3/66_Confidence_0.063_Length_450,

			(phosphorylating), ec:4.1.1.17 - decarboxylase, ec:2.7.1.148 - 5'-diphospho)-2-C-methyl-D-erythritol kinase, ec:4.2.1.91 - dehydratase, ec:2.7.6.1 - diphosphokinase, ec:1.17.1.8 - reductase, ec:1.8.1.4 - dehydrogenase, ec:2.5.1.1 - geranyl-diphosphate synthase, ec:4.6.1.12 - 2,4-cyclodiphosphate synthase, ec:5.3.1.1 - isomerase, ec:5.3.1.8 - isomerase, ec:5.3.1.6 - isomerase, ec:5.3.1.9 - isomerase, ec:2.1.1.41 - 24-C-methyltransferase, ec:2.7.7.60 - 4-phosphate cytidylyltransferase, ec:3.1.3.3 - phosphatase, ec:1.1.1.2 - dehydrogenase (NADP+), ec:1.1.1.3 - dehydrogenase, ec:2.6.1.1 - transaminase, ec:5.3.3.2 - Delta-isomerase, ec:4.3.1.17 - ammonia-lyase, ec:4.3.1.19 - ammonia-lyase, ec:1.3.1.13 - dehydrogenase (NADP+), ec:1.3.1.12 - dehydrogenase, ec:1.2.1.3 - dehydrogenase (NAD+), ec:3.5.1.14 - acid amidohydrolase, ec:2.3.1.9 - C-acetyltransferase, ec:2.3.1.1 - N-acetyltransferase, ec:4.4.1.1 - gamma-lyase, ec:1.1.5.4 - dehydrogenase (quinone), ec:4.3.3.7 - synthase, ec:6.3.5.3 - synthase, ec:1.14.13.72 - monooxygenase, ec:2.3.3.8 - citrate synthase, ec:1.5.1.2 - reductase, ec:5.4.99.18 - ribonucleotide mutase, ec:2.3.3.1 - (Si)-synthase, ec:1.5.1.8 - dehydrogenase (NADP+, L-lysine-forming), ec:1.5.1.9 - dehydrogenase (NAD+, L-glutamate-forming), ec:5.1.3.13 - 3,5-epimerase, ec:2.3.3.10 - synthase, ec:2.1.2.3 -		tri_c_comp20343_c0_seq4, tri_c_comp20343_c0_seq3, vo_n2_Locus_3_Transcript_22566/24291_Confidence_0.000_Length_2168, vo_n2_Locus_3452_Transcript_2/4_Confidence_0.636_Length_263, vo_n2_Locus_3_Transcript_22486/24291_Confidence_0.000_Length_1883, vo_c1_Locus_1636_Transcript_120/122_Confidence_0.135_Length_3625, vo_c2_Locus_52_Transcript_120/466_Confidence_0.009_Length_610, vo_c3_Locus_31_Transcript_13/79_Confidence_0.099_Length_748, vo_c1_Locus_1636_Transcript_69/122_Confidence_0.074_Length_844, vo_n2_Locus_3_Transcript_22520/24291_Confidence_0.000_Length_1452, vo_n2_Locus_3_Transcript_22533/24291_Confidence_0.000_Length_679, vo_n2_Locus_3_Transcript_22537/24291_Confidence_0.000_Length_840, vo_c3_Locus_31_Transcript_63/79_Confidence_0.148_Length_3613, vo_c1_Locus_1636_Transcript_71/122_Confidence_0.055_Length_846, vo_c3_Locus_31_Transcript_77/79_Confidence_0.297_Length_1736, vo_c1_Locus_1636_Transcript_83/122_Confidence_0.191_Length_2430, tri_n_comp20833_c0_seq3, vo_c3_Locus_31_Transcript_75/79_Confidence_0.212_Length_8463, vo_c2_Locus_52_Transcript_153/466_Confidence_0.026_Length_1551, vo_c3_Locus_31_Transcript_3/79_Confidence_0.035_Length_301, vo_n2_Locus_3_Transcript_22489/24291_Confidence_0.000_Length_274, vo_n2_Locus_3_Transcript_22509/24291_Confidence_0.000_Length_800, vo_n3_Locus_37_Transcript_38/46_Confidence_0.163_Length_922, tri_c_comp21288_c0_seq1, vo_c1_Locus_1636_Transcript_88/122_Confidence_0.074_Length_1384, vo_c1_Locus_1636_Transcript_121/122_Confidence_0.018_Length_960, vo_n3_Locus_37_Transcript_5/46_Confidence_0.289_Length_1505, vo_n1_Locus_55_Transcript_33/66_Confidence_0.040_Length_1095, vo_c3_Locus_31_Transcript_65/79_Confidence_0.166_Length_2369, vo_c3_Locus_31_Transcript_73/79_Confidence_0.208_Length_2557, vo_c3_Locus_31_Transcript_9/79_Confidence_0.152_Length_714, vo_c2_Locus_52_Transcript_113/466_Confidence_0.004_Length_590, vo_n2_Locus_3_Transcript_22492/24291_Confidence_0.000_Length_1595, vo_n1_Locus_55_Transcript_54/66_Confidence_0.183_Length_2185, vo_c3_Locus_33_Transcript_2/2_Confidence_0.714_Length_611, vo_c2_Locus_52_Transcript_125/466_Confidence_0.007_Length_559, vo_c2_Locus_52_Transcript_135/466_Confidence_0.025_Length_2711, vo_n1_Locus_55_Transcript_12/66_Confidence_0.331_Length_2232, vo_c1_Locus_1636_Transcript_100/122_Confidence_0.031_Length_685, vo_c2_Locus_52_Transcript_131/466_Confidence_0.023_Length_1964, vo_c3_Locus_31_Transcript_7/79_Confidence_0.184_Length_710, vo_n1_Locus_55_Transcript_39/66_Confidence_0.194_Length_1391, vo_n3_Locus_37_Transcript_28/46_Confidence_0.193_Length_1043, vo_n1_Locus_55_Transcript_41/66_Confidence_0.263_Length_1742, vo_n1_Locus_55_Transcript_38/66_Confidence_0.171_Length_1387, vo_n2_Locus_3_Transcript_22491/24291_Confidence_0.000_Length_1174, vo_n3_Locus_37_Transcript_30/46_Confidence_0.178_Length_1043, vo_n2_Locus_3_Transcript_22503/24291_Confidence_0.000_Length_721, vo_n2_Locus_3_Transcript_22540/24291_Confidence_0.000_Length_271, vo_c2_Locus_52_Transcript_128/466_Confidence_0.003_Length_564, vo_c2_Locus_52_Transcript_162/466_Confidence_0.019_Length_1668, vo_n2_Locus_3_Transcript_22534/24291_Confidence_0.000_Length_449, vo_c1_Locus_1636_Transcript_116/122_Confidence_0.129_Length_3526, vo_c3_Locus_31_Transcript_70/79_Confidence_0.208_Length_1586, vo_n1_Locus_55_Transcript_9/66_Confidence_0.349_Length_2172, vo_c1_Locus_8362_Transcript_4/9_Confidence_0.254_Length_482, vo_n1_Locus_55_Transcript_50/66_Confidence_0.166_Length_2379, vo_n2_Locus_5500_Transcript_5/5_Confidence_0.308_Length_1268, vo_n2_Locus_5500_Transcript_1/5_Confidence_0.308_Length_1100, tri_n_comp12494_c0_seq1, vo_n2_Locus_5500_Transcript_2/5_Confidence_0.538_Length_1312, vo_n2_Locus_2182_Transcript_14/15_Confidence_0.458_Length_3129, vo_c2_Locus_642_Transcript_3/10_Confidence_0.571_Length_2141, vo_n1_Locus_1508_Transcript_4/6_Confidence_0.429_Length_1979, vo_n1_Locus_2259_Transcript_6/13_Confidence_0.429_Length_1275, vo_n1_Locus_2259_Transcript_9/13_Confidence_0.429_Length_1314, vo_c1_Locus_1462_Transcript_1/46_Confidence_0.182_Length_2129, vo_c1_Locus_1462_Transcript_8/46_Confidence_0.530_Length_9104, vo_n2_Locus_26350_Transcript_7/9_Confidence_0.231_Length_1309, vo_c1_Locus_2295_Transcript_9/10_Confidence_0.238_Length_2213, vo_c2_Locus_2862_Transcript_9/10_Confidence_0.250_Length_2209, vo_c1_Locus_2295_Transcript_3/10_Confidence_0.524_Length_2330, vo_n2_Locus_1690_Transcript_8/11_Confidence_0.429_Length_2234, vo_n2_Locus_1690_Transcript_9/11_Confidence_0.429_Length_2277, tri_c_comp30246_c0_seq1, vo_n1_Locus_454_Transcript_6/7_Confidence_0.364_Length_3522, tri_n_comp20539_c0_seq1, vo_n2_Locus_2098_Transcript_8/8_Confidence_0.130_Length_2485, vo_n2_Locus_2440_Transcript_3/6_Confidence_0.182_Length_1751, tri_c_comp24934_c0_seq1, vo_c3_Locus_9067_Transcript_6/7_Confidence_0.125_Length_1757, vo_c2_Locus_1886_Transcript_3/5_Confidence_0.444_Length_2764, vo_c3_Locus_1314_Transcript_17/46_Confidence_0.365_Length_2530, vo_n3_Locus_387_Transcript_34/39_Confidence_0.386_Length_2514, vo_c2_Locus_924_Transcript_20/40_Confidence_0.452_Length_2520, vo_c2_Locus_924_Transcript_13/40_Confidence_0.310_Length_2559, vo_c3_Locus_1314_Transcript_33/46_Confidence_0.292_Length_2524, vo_c1_Locus_1204_Transcript_14/17_Confidence_0.394_Length_4476, vo_c2_Locus_924_Transcript_19/40_Confidence_0.333_Length_2562, vo_n1_Locus_327_Transcript_13/41_Confidence_0.275_Length_2480, vo_c3_Locus_1314_Transcript_6/46_Confidence_0.208_Length_1518, vo_n1_Locus_327_Transcript_40/41_Confidence_0.176_Length_2442, vo_c3_Locus_1314_Transcript_38/46_Confidence_0.302_Length_2522, vo_c2_Locus_924_Transcript_34/40_Confidence_0.369_Length_2618, vo_c3_Locus_1314_Transcript_14/46_Confidence_0.229_Length_3828, vo_c2_Locus_924_Transcript_16/40_Confidence_0.321_Length_2559, vo_c2_Locus_924_Transcript_23/40_Confidence_0.488_Length_2850, vo_c3_Locus_1314_Transcript_21/46_Confidence_0.365_Length_2524, vo_c1_Locus_1204_Transcript_13/17_Confidence_0.364_Length_4497, vo_n1_Locus_327_Transcript_24/41_Confidence_0.385_Length_2547, vo_c2_Locus_924_Transcript_17/40_Confidence_0.333_Length_2453, tri_c_comp20608_c0_seq4, tri_c_comp20608_c0_seq5, vo_c3_Locus_1314_Transcript_43/46_Confidence_0.198_Length_2519, vo_n2_Locus_2054_Transcript_26/37_Confidence_0.448_Length_2522, tri_c_comp20608_c0_seq7, tri_n_comp26150_c0_seq1, vo_c1_Locus_12331_Transcript_1/1_Confidence_0.000_Length_2548, vo_c1_Locus_1636_Transcript_120/122_Confidence_0.135_Length_3625, tri_n_comp29741_c0_seq1, vo_c3_Locus_31_Transcript_74/79_Confidence_0.124_Length_2769, vo_c2_Locus_10862_Transcript_1/1_Confidence_0.000_Length_1564, vo_c3_Locus_31_Transcript_63/79_Confidence_0.148_Length_3613, vo_c1_Locus_1636_Transcript_116/122_Confidence_0.129_Length_3526, vo_c2_Locus_52_Transcript_164/466_Confidence_0.011_Length_2810,

			formyltransferase, ec:2.1.2.2 - formyltransferase, ec:2.1.2.1 - hydroxymethyltransferase, ec:2.3.1.61 - succinyltransferase, ec:4.1.2.13 - aldolase, ec:6.3.4.5 - synthase, ec:4.2.1.9 - dehydratase, ec:4.2.1.3 - hydratase, ec:4.2.1.2 - hydratase, ec:3.5.3.1 - arginine amidinase, ec:2.7.2.11 - 5-kinase, ec:6.3.2.6 - synthase, ec:2.7.1.2 - glucokinase (phosphorylating), ec:4.2.3.4 - synthase, ec:5.1.3.1 - 3-epimerase, ec:3.1.3.18 - phosphatase, ec:3.1.3.11 - hexose diphosphatase, ec:2.7.7.4 - adenylyltransferase, ec:3.1.3.25 - phosphatase, ec:2.7.7.9 - uridylyltransferase, ec:2.3.1.31 - O-acetyltransferase, ec:2.3.1.30 - O-acetyltransferase, ec:2.3.1.35 - N-acetyltransferase, ec:6.3.4.13 - ligase, ec:1.1.1.267 - reductoisomerase, ec:3.1.1.31 - phosphogluconolactonase, ec:5.4.99.7 - synthase, ec:5.4.99.5 - mutase, ec:2.1.1.100 - O-methyltransferase, ec:4.2.1.22 - beta-synthase, ec:4.2.1.20 - synthase, ec:2.5.1.47 - synthase, ec:4.2.1.11 - hydratase, ec:4.2.1.10 - dehydratase, ec:2.5.1.54 - synthase, ec:2.5.1.58 - farnesyltransferase, ec:4.2.1.17 - hydratase, ec:1.4.1.14 - synthase (NADH), ec:1.4.1.13 - synthase (NADPH), ec:1.1.3.15 - oxidase, ec:4.2.1.46 - 4,6-		vo_n3_Locus_2623_Transcript_14/17_Confidence_0.556_Length_1555, vo_n3_Locus_74_Transcript_17/25_Confidence_0.436_Length_1764, vo_c1_Locus_788_Transcript_17/34_Confidence_0.404_Length_1861, vo_c1_Locus_788_Transcript_30/34_Confidence_0.255_Length_1712, tri_c_comp19022_c0_seq2, vo_c3_Locus_239_Transcript_24/29_Confidence_0.409_Length_3108, vo_c2_Locus_298_Transcript_10/24_Confidence_0.500_Length_1796, tri_n_comp18813_c0_seq15, vo_n2_Locus_106_Transcript_17/30_Confidence_0.344_Length_2068, tri_n_comp18813_c0_seq11, vo_n1_Locus_2248_Transcript_15/40_Confidence_0.275_Length_1844, tri_n_comp18813_c0_seq10, tri_n_comp20156_c0_seq1, vo_c2_Locus_3634_Transcript_2/8_Confidence_0.467_Length_2376, vo_c1_Locus_788_Transcript_5/34_Confidence_0.340_Length_1423, vo_n2_Locus_2252_Transcript_14/26_Confidence_0.410_Length_2227, vo_c3_Locus_239_Transcript_16/29_Confidence_0.364_Length_1433, vo_c3_Locus_2347_Transcript_16/16_Confidence_0.270_Length_1737, vo_c2_Locus_298_Transcript_21/24_Confidence_0.368_Length_1678, vo_c3_Locus_3927_Transcript_2/9_Confidence_0.250_Length_2629, vo_c2_Locus_3634_Transcript_8/8_Confidence_0.133_Length_2428, vo_c2_Locus_2437_Transcript_14/25_Confidence_0.455_Length_1511, vo_c1_Locus_3033_Transcript_3/5_Confidence_0.400_Length_2348, vo_c3_Locus_239_Transcript_29/29_Confidence_0.182_Length_2208, vo_n3_Locus_2623_Transcript_15/17_Confidence_0.361_Length_1551, vo_c3_Locus_2347_Transcript_5/16_Confidence_0.514_Length_1554, vo_c3_Locus_239_Transcript_19/29_Confidence_0.295_Length_1502, vo_c2_Locus_2437_Transcript_25/25_Confidence_0.159_Length_1517, vo_n2_Locus_2252_Transcript_22/26_Confidence_0.256_Length_2337, vo_n2_Locus_2252_Transcript_23/26_Confidence_0.359_Length_1959, vo_c3_Locus_239_Transcript_10/29_Confidence_0.386_Length_1727, vo_c1_Locus_788_Transcript_6/34_Confidence_0.362_Length_1766, vo_c1_Locus_788_Transcript_22/34_Confidence_0.277_Length_1358, vo_n1_Locus_2248_Transcript_26/40_Confidence_0.333_Length_1991, tri_c_comp20571_c0_seq1, vo_n2_Locus_2252_Transcript_18/26_Confidence_0.256_Length_1506, vo_c1_Locus_788_Transcript_11/34_Confidence_0.213_Length_2514, vo_c2_Locus_907_Transcript_3/3_Confidence_0.333_Length_630, vo_n2_Locus_7087_Transcript_1/2_Confidence_0.500_Length_4087, vo_n1_Locus_2248_Transcript_2/40_Confidence_0.373_Length_1784, vo_n2_Locus_2980_Transcript_1/1_Confidence_0.333_Length_1623, vo_c2_Locus_18300_Transcript_2/3_Confidence_0.200_Length_1671, vo_n2_Locus_9167_Transcript_1/4_Confidence_0.286_Length_6748, vo_n2_Locus_3068_Transcript_2/3_Confidence_0.250_Length_1991, vo_n1_Locus_4025_Transcript_3/5_Confidence_0.286_Length_3475, vo_c1_Locus_9004_Transcript_2/4_Confidence_0.400_Length_3537, vo_c1_Locus_10440_Transcript_1/2_Confidence_0.667_Length_1569, vo_n2_Locus_9037_Transcript_9/15_Confidence_0.486_Length_4553, vo_c2_Locus_684_Transcript_7/16_Confidence_0.194_Length_2803, vo_n3_Locus_2293_Transcript_1/2_Confidence_0.750_Length_1794, vo_n3_Locus_1756_Transcript_10/15_Confidence_0.393_Length_2226, vo_c3_Locus_725_Transcript_6/15_Confidence_0.548_Length_5086, vo_n3_Locus_1756_Transcript_7/15_Confidence_0.250_Length_2226, vo_n2_Locus_16147_Transcript_1/1_Confidence_0.000_Length_1452, vo_n2_Locus_9037_Transcript_2/15_Confidence_0.257_Length_3951, vo_n2_Locus_9037_Transcript_13/15_Confidence_0.143_Length_1729, vo_n2_Locus_9037_Transcript_5/15_Confidence_0.286_Length_2279, vo_n2_Locus_9037_Transcript_6/15_Confidence_0.229_Length_2279, tri_c_comp19644_c0_seq1, vo_n2_Locus_171_Transcript_17/42_Confidence_0.229_Length_1803, tri_c_comp19644_c0_seq4, vo_c3_Locus_12256_Transcript_3/3_Confidence_0.000_Length_508, vo_c3_Locus_725_Transcript_12/15_Confidence_0.290_Length_3194, vo_c1_Locus_6543_Transcript_5/23_Confidence_0.220_Length_2294, vo_n2_Locus_9037_Transcript_12/15_Confidence_0.257_Length_2318, vo_c3_Locus_725_Transcript_13/15_Confidence_0.452_Length_4249, tri_n_comp19211_c0_seq1, vo_c3_Locus_883_Transcript_1/2_Confidence_1.000_Length_1783, vo_n2_Locus_9037_Transcript_1/15_Confidence_0.143_Length_1677, vo_c3_Locus_307_Transcript_2/21_Confidence_0.576_Length_1809, vo_n1_Locus_41_Transcript_20/90_Confidence_0.068_Length_2175, vo_c2_Locus_1667_Transcript_21/21_Confidence_0.195_Length_1788, tri_c_comp80018_c0_seq1, vo_c1_Locus_37084_Transcript_1/2_Confidence_0.667_Length_2193, vo_c3_Locus_20892_Transcript_1/1_Confidence_0.000_Length_2501, vo_c2_Locus_384_Transcript_2/3_Confidence_0.400_Length_1725, tri_c_comp32679_c0_seq1, vo_c1_Locus_10788_Transcript_1/1_Confidence_0.000_Length_1721, vo_c2_Locus_14969_Transcript_4/8_Confidence_0.750_Length_1231, vo_n2_Locus_3786_Transcript_2/4_Confidence_0.778_Length_1466, vo_n2_Locus_3786_Transcript_3/4_Confidence_0.667_Length_1442, vo_c3_Locus_550_Transcript_25/30_Confidence_0.321_Length_1640, vo_c3_Locus_550_Transcript_10/30_Confidence_0.420_Length_1515, vo_n1_Locus_133_Transcript_12/30_Confidence_0.033_Length_580, vo_c2_Locus_320_Transcript_7/32_Confidence_0.299_Length_1642, vo_c3_Locus_550_Transcript_27/30_Confidence_0.395_Length_1758, vo_c2_Locus_320_Transcript_9/32_Confidence_0.338_Length_1673, vo_c2_Locus_320_Transcript_22/32_Confidence_0.545_Length_1648, vo_c3_Locus_550_Transcript_30/30_Confidence_0.210_Length_1413, vo_c3_Locus_550_Transcript_8/30_Confidence_0.148_Length_1492, vo_n3_Locus_587_Transcript_13/22_Confidence_0.444_Length_1456, vo_n2_Locus_105_Transcript_30/33_Confidence_0.449_Length_1990, vo_c3_Locus_550_Transcript_28/30_Confidence_0.309_Length_1831, vo_c2_Locus_320_Transcript_20/32_Confidence_0.325_Length_1586, vo_n1_Locus_133_Transcript_30/30_Confidence_0.033_Length_2622, vo_c2_Locus_320_Transcript_13/32_Confidence_0.351_Length_2604, vo_c2_Locus_320_Transcript_15/32_Confidence_0.390_Length_1557, vo_c3_Locus_1613_Transcript_6/17_Confidence_0.158_Length_5809, vo_n1_Locus_10193_Transcript_1/1_Confidence_0.000_Length_1647, vo_c2_Locus_6348_Transcript_1/2_Confidence_1.000_Length_3755, vo_n3_Locus_23556_Transcript_2/4_Confidence_0.500_Length_4210, tri_c_comp43537_c0_seq1, vo_c2_Locus_15226_Transcript_2/2_Confidence_0.500_Length_2141, tri_c_comp29205_c0_seq1, vo_c1_Locus_2207_Transcript_20/51_Confidence_0.393_Length_3556, vo_c3_Locus_2283_Transcript_28/38_Confidence_0.331_Length_3501, vo_n3_Locus_1164_Transcript_30/46_Confidence_0.400_Length_3745, tri_c_comp20184_c0_seq2, vo_c2_Locus_7270_Transcript_1/1_Confidence_0.500_Length_1488, vo_c1_Locus_31_Transcript_859/1155_Confidence_0.002_Length_1257, vo_n3_Locus_1164_Transcript_32/46_Confidence_0.456_Length_3745, vo_n1_Locus_3448_Transcript_46/64_Confidence_0.085_Length_783, vo_c2_Locus_644_Transcript_12/40_Confidence_0.459_Length_3665, vo_n3_Locus_1164_Transcript_35/46_Confidence_0.511_Length_3745,

			dehydratase, ec:1.1.1.86 - reductoisomerase (NADP+), ec:1.1.1.37 - dehydrogenase, ec:1.1.1.35 - dehydrogenase, ec:1.1.1.44 - dehydrogenase (NADP+-dependent, decarboxylating), ec:1.1.1.42 - dehydrogenase (NADP+), ec:1.1.1.49 - dehydrogenase (NADP+), ec:5.4.2.2 - (alpha-D-glucose-1,6-bisphosphate-dependent), ec:5.4.2.3 - mutase, ec:1.2.4.2 - dehydrogenase (succinyl-transferring), ec:1.2.4.4 - dehydrogenase (2-methylpropanoyl-transferring), ec:3.5.4.10 - cyclohydrolase, ec:5.3.1.24 - isomerase, ec:2.7.4.10 - kinase		vo_c3_Locus_5_Transcript_229/353_Confidence_0.004_Length_952, vo_c2_Locus_644_Transcript_3/40_Confidence_0.071_Length_957, vo_n1_Locus_3448_Transcript_53/64_Confidence_0.225_Length_3567, vo_n2_Locus_386_Transcript_32/33_Confidence_0.208_Length_3664, vo_n1_Locus_3448_Transcript_63/64_Confidence_0.318_Length_3637, vo_n3_Locus_1164_Transcript_33/46_Confidence_0.456_Length_3748, vo_c3_Locus_2283_Transcript_26/38_Confidence_0.285_Length_3501, vo_n2_Locus_386_Transcript_22/33_Confidence_0.376_Length_3568, vo_c3_Locus_2283_Transcript_29/38_Confidence_0.292_Length_3501, vo_n2_Locus_386_Transcript_1/33_Confidence_0.139_Length_3600, vo_c2_Locus_644_Transcript_24/40_Confidence_0.398_Length_3511, vo_n3_Locus_1164_Transcript_43/46_Confidence_0.389_Length_3338, vo_c1_Locus_2207_Transcript_39/51_Confidence_0.250_Length_3586, vo_c3_Locus_79_Transcript_1/3_Confidence_0.200_Length_5222, vo_c2_Locus_52_Transcript_234/466_Confidence_0.003_Length_1462, vo_n2_Locus_386_Transcript_10/33_Confidence_0.376_Length_3676, vo_n1_Locus_1394_Transcript_2/3_Confidence_0.400_Length_2232, tri_n_comp19507_c0_seq6, tri_c_comp32056_c0_seq1, vo_c3_Locus_79_Transcript_1/3_Confidence_0.200_Length_5222, vo_n1_Locus_1394_Transcript_2/3_Confidence_0.400_Length_2232, vo_n2_Locus_2182_Transcript_14/15_Confidence_0.458_Length_3129, vo_c1_Locus_1462_Transcript_1/46_Confidence_0.182_Length_2129, vo_c1_Locus_1462_Transcript_8/46_Confidence_0.530_Length_9104, vo_c2_Locus_642_Transcript_3/10_Confidence_0.571_Length_2141, vo_n3_Locus_9269_Transcript_1/1_Confidence_0.000_Length_2238, vo_n2_Locus_19315_Transcript_2/2_Confidence_0.000_Length_1802, tri_c_comp29205_c0_seq1, vo_c2_Locus_1643_Transcript_1/1_Confidence_0.000_Length_1756, vo_c1_Locus_3806_Transcript_1/1_Confidence_0.000_Length_1482, vo_n2_Locus_3068_Transcript_2/3_Confidence_0.250_Length_1991, vo_n1_Locus_4025_Transcript_3/5_Confidence_0.286_Length_3475, vo_c1_Locus_9004_Transcript_2/4_Confidence_0.400_Length_3537, vo_c1_Locus_10440_Transcript_1/2_Confidence_0.667_Length_1569, vo_c3_Locus_550_Transcript_25/30_Confidence_0.321_Length_1640, vo_c3_Locus_550_Transcript_10/30_Confidence_0.420_Length_1515, vo_n1_Locus_133_Transcript_12/30_Confidence_0.033_Length_580, vo_n2_Locus_13049_Transcript_20/20_Confidence_0.289_Length_1233, vo_c3_Locus_1015_Transcript_30/32_Confidence_0.181_Length_1172, vo_n2_Locus_13049_Transcript_19/20_Confidence_0.368_Length_1268, vo_c3_Locus_550_Transcript_27/30_Confidence_0.395_Length_1758, vo_n1_Locus_296_Transcript_24/31_Confidence_0.275_Length_1182, vo_n2_Locus_13049_Transcript_17/20_Confidence_0.342_Length_1329, vo_c3_Locus_1015_Transcript_13/32_Confidence_0.222_Length_1363, vo_c2_Locus_1551_Transcript_19/27_Confidence_0.222_Length_1248, vo_n1_Locus_296_Transcript_27/31_Confidence_0.078_Length_2886, vo_c3_Locus_1015_Transcript_3/32_Confidence_0.181_Length_1206, vo_n2_Locus_105_Transcript_30/33_Confidence_0.449_Length_1990, vo_c3_Locus_550_Transcript_28/30_Confidence_0.309_Length_1831, vo_n3_Locus_150_Transcript_17/25_Confidence_0.183_Length_1185, vo_n3_Locus_150_Transcript_6/25_Confidence_0.232_Length_1192, vo_n1_Locus_133_Transcript_30/30_Confidence_0.033_Length_2622, tri_c_comp21115_c0_seq1, vo_n3_Locus_587_Transcript_2/22_Confidence_0.111_Length_206, vo_c2_Locus_320_Transcript_13/32_Confidence_0.351_Length_2604, vo_n1_Locus_296_Transcript_25/31_Confidence_0.157_Length_1180, vo_n2_Locus_13049_Transcript_7/20_Confidence_0.421_Length_1354, vo_c1_Locus_223_Transcript_13/65_Confidence_0.197_Length_1636, vo_c1_Locus_223_Transcript_47/65_Confidence_0.099_Length_1259, vo_c2_Locus_320_Transcript_7/32_Confidence_0.299_Length_1642, vo_n1_Locus_133_Transcript_14/30_Confidence_0.456_Length_2738, vo_c3_Locus_1015_Transcript_12/32_Confidence_0.222_Length_1270, vo_c2_Locus_320_Transcript_9/32_Confidence_0.338_Length_1673, vo_c2_Locus_320_Transcript_22/32_Confidence_0.545_Length_1648, vo_n1_Locus_296_Transcript_23/31_Confidence_0.294_Length_1182, vo_n2_Locus_13049_Transcript_9/20_Confidence_0.316_Length_1224, vo_c1_Locus_223_Transcript_52/65_Confidence_0.042_Length_799, vo_c3_Locus_550_Transcript_30/30_Confidence_0.210_Length_1413, vo_n1_Locus_133_Transcript_13/30_Confidence_0.456_Length_2508, vo_n1_Locus_133_Transcript_24/30_Confidence_0.311_Length_1984, vo_n1_Locus_296_Transcript_30/31_Confidence_0.078_Length_1319, vo_n1_Locus_133_Transcript_15/30_Confidence_0.456_Length_2671, vo_c2_Locus_320_Transcript_20/32_Confidence_0.325_Length_1586, vo_c1_Locus_223_Transcript_19/65_Confidence_0.092_Length_1454, vo_n1_Locus_296_Transcript_10/31_Confidence_0.118_Length_1164, vo_c1_Locus_223_Transcript_54/65_Confidence_0.134_Length_1458, vo_c2_Locus_320_Transcript_15/32_Confidence_0.390_Length_1557, vo_c3_Locus_1015_Transcript_9/32_Confidence_0.236_Length_1272, vo_n1_Locus_133_Transcript_25/30_Confidence_0.278_Length_2179, vo_n1_Locus_24_Transcript_2/4_Confidence_0.667_Length_1878, vo_c2_Locus_14475_Transcript_1/1_Confidence_0.000_Length_1444, vo_c2_Locus_15226_Transcript_2/2_Confidence_0.500_Length_2141, vo_c3_Locus_18398_Transcript_2/2_Confidence_0.000_Length_715, vo_n2_Locus_18825_Transcript_1/3_Confidence_0.500_Length_1632, tri_c_comp33638_c0_seq1, vo_n2_Locus_8943_Transcript_1/2_Confidence_0.750_Length_1351, vo_n1_Locus_32462_Transcript_1/1_Confidence_0.000_Length_1047, vo_n2_Locus_24260_Transcript_2/3_Confidence_0.250_Length_976, vo_c1_Locus_22485_Transcript_1/2_Confidence_0.500_Length_1035, vo_n3_Locus_6810_Transcript_2/3_Confidence_0.913_Length_1699, vo_n1_Locus_6460_Transcript_1/1_Confidence_0.000_Length_1912, tri_c_comp29205_c0_seq1, vo_n2_Locus_26426_Transcript_1/1_Confidence_0.000_Length_3133, tri_c_comp37590_c0_seq1, vo_n2_Locus_15100_Transcript_1/9_Confidence_0.417_Length_1002, tri_n_comp46466_c0_seq1, vo_c2_Locus_3634_Transcript_8/8_Confidence_0.133_Length_2428, vo_c2_Locus_324_Transcript_5/12_Confidence_0.333_Length_1009, vo_n1_Locus_5721_Transcript_6/9_Confidence_0.389_Length_989, vo_c2_Locus_324_Transcript_11/12_Confidence_0.333_Length_4393, vo_n2_Locus_475_Transcript_7/13_Confidence_0.312_Length_1141, vo_n1_Locus_5721_Transcript_1/9_Confidence_0.278_Length_986, vo_n2_Locus_475_Transcript_2/13_Confidence_0.312_Length_1187, vo_c2_Locus_324_Transcript_2/12_Confidence_0.400_Length_1106, vo_c3_Locus_3927_Transcript_2/9_Confidence_0.250_Length_2629, vo_c2_Locus_1828_Transcript_1/2_Confidence_0.963_Length_1886, vo_n2_Locus_19533_Transcript_1/1_Confidence_0.000_Length_1783, vo_c1_Locus_34898_Transcript_1/1_Confidence_0.000_Length_1083, vo_n2_Locus_8097_Transcript_1/6_Confidence_0.500_Length_1196, vo_n2_Locus_11241_Transcript_1/3_Confidence_0.600_Length_3773, vo_c2_Locus_479_Transcript_1/2_Confidence_1.000_Length_2154, vo_c2_Locus_11041_Transcript_1/2_Confidence_0.750_Length_1378, vo_c2_Locus_11041_Transcript_2/2_Confidence_0.000_Length_1411, vo_c3_Locus_22219_Transcript_1/1_Confidence_0.000_Length_1123,

					vo_c3_Locus_3840_Transcript_2/5_Confidence_0.200_Length_1699, vo_c2_Locus_27691_Transcript_1/2_Confidence_0.750_Length_923, tri_c_comp11242_c0_seq2, tri_n_comp64914_c0_seq1, vo_c2_Locus_9062_Transcript_10/11_Confidence_0.312_Length_4945, vo_n2_Locus_22224_Transcript_1/1_Confidence_0.000_Length_1370, tri_n_comp14915_c0_seq1, vo_n3_Locus_15713_Transcript_4/7_Confidence_0.429_Length_2557, tri_c_comp37399_c0_seq1, tri_n_comp58061_c0_seq1, vo_n1_Locus_22736_Transcript_1/2_Confidence_0.333_Length_1213, vo_n2_Locus_19677_Transcript_1/1_Confidence_0.000_Length_2823, vo_c2_Locus_10862_Transcript_1/1_Confidence_0.000_Length_1564, vo_n1_Locus_3423_Transcript_2/4_Confidence_0.250_Length_1347, tri_n_comp27683_c0_seq1, vo_c1_Locus_3127_Transcript_1/1_Confidence_0.000_Length_1478, vo_n1_Locus_3423_Transcript_3/4_Confidence_0.250_Length_1416, tri_c_comp43842_c0_seq1, vo_c2_Locus_30746_Transcript_1/1_Confidence_0.000_Length_1279, vo_n1_Locus_2779_Transcript_1/2_Confidence_0.333_Length_2962, vo_c2_Locus_30746_Transcript_1/1_Confidence_0.000_Length_1279, tri_c_comp34117_c0_seq1, vo_c2_Locus_15226_Transcript_2/2_Confidence_0.500_Length_2141, vo_c2_Locus_15226_Transcript_2/2_Confidence_0.500_Length_2141, tri_c_comp55340_c0_seq1, vo_n1_Locus_19864_Transcript_2/2_Confidence_0.000_Length_2707, vo_c3_Locus_21741_Transcript_5/9_Confidence_0.583_Length_1757, vo_n3_Locus_25600_Transcript_5/6_Confidence_0.375_Length_1777, tri_n_comp58061_c0_seq1, tri_c_comp31460_c0_seq1, vo_n2_Locus_4794_Transcript_3/3_Confidence_0.000_Length_966, vo_c1_Locus_2657_Transcript_1/1_Confidence_0.000_Length_1513, vo_n2_Locus_23058_Transcript_1/1_Confidence_0.000_Length_1342, vo_n2_Locus_4233_Transcript_1/2_Confidence_1.000_Length_2049, tri_c_comp42317_c0_seq1, vo_n2_Locus_7374_Transcript_2/3_Confidence_0.750_Length_1746, vo_n2_Locus_8711_Transcript_5/5_Confidence_0.067_Length_1558, vo_c2_Locus_11984_Transcript_2/6_Confidence_0.385_Length_1557, vo_c2_Locus_31468_Transcript_4/5_Confidence_0.667_Length_1579, vo_n1_Locus_3498_Transcript_1/3_Confidence_0.600_Length_1462, tri_c_comp30049_c0_seq1, vo_n1_Locus_833_Transcript_2/6_Confidence_0.333_Length_2200, vo_n2_Locus_3042_Transcript_2/3_Confidence_0.750_Length_2030, vo_c1_Locus_6244_Transcript_1/2_Confidence_0.750_Length_1731, vo_c3_Locus_7185_Transcript_2/3_Confidence_0.333_Length_1661, vo_c3_Locus_7565_Transcript_10/13_Confidence_0.400_Length_4317, tri_c_comp16509_c0_seq1, vo_c3_Locus_7565_Transcript_1/13_Confidence_0.375_Length_4301, vo_n1_Locus_4052_Transcript_3/24_Confidence_0.524_Length_4370, vo_c1_Locus_12613_Transcript_1/1_Confidence_0.000_Length_2362, vo_c1_Locus_2207_Transcript_20/51_Confidence_0.393_Length_3556, vo_c3_Locus_2283_Transcript_28/38_Confidence_0.331_Length_3501, vo_n3_Locus_1164_Transcript_30/46_Confidence_0.400_Length_3745, tri_c_comp20184_c0_seq2, vo_n3_Locus_1164_Transcript_32/46_Confidence_0.456_Length_3745, vo_n1_Locus_3448_Transcript_1/64_Confidence_0.062_Length_478, vo_n1_Locus_3448_Transcript_46/64_Confidence_0.085_Length_783, vo_c2_Locus_644_Transcript_12/40_Confidence_0.459_Length_3665, vo_n3_Locus_1164_Transcript_35/46_Confidence_0.511_Length_3745, vo_c2_Locus_644_Transcript_3/40_Confidence_0.071_Length_957, vo_n1_Locus_3448_Transcript_53/64_Confidence_0.225_Length_3567, vo_n2_Locus_386_Transcript_32/33_Confidence_0.208_Length_3664, vo_n1_Locus_3448_Transcript_63/64_Confidence_0.318_Length_3637, vo_n3_Locus_1164_Transcript_33/46_Confidence_0.456_Length_3748, vo_c3_Locus_2283_Transcript_26/38_Confidence_0.285_Length_3501, vo_n2_Locus_386_Transcript_22/33_Confidence_0.376_Length_3568, vo_n1_Locus_3448_Transcript_2/64_Confidence_0.054_Length_402, vo_c3_Locus_2283_Transcript_29/38_Confidence_0.292_Length_3501, vo_c3_Locus_2283_Transcript_35/38_Confidence_0.400_Length_3582, vo_n2_Locus_386_Transcript_1/33_Confidence_0.139_Length_3600, vo_c2_Locus_644_Transcript_24/40_Confidence_0.398_Length_3511, vo_n3_Locus_1164_Transcript_43/46_Confidence_0.389_Length_3338, vo_c1_Locus_2207_Transcript_39/51_Confidence_0.250_Length_3586, vo_c3_Locus_79_Transcript_1/3_Confidence_0.200_Length_5222, vo_n2_Locus_386_Transcript_10/33_Confidence_0.376_Length_3676, vo_n1_Locus_1394_Transcript_2/3_Confidence_0.400_Length_2232, vo_c2_Locus_644_Transcript_6/40_Confidence_0.204_Length_3616, vo_c1_Locus_2207_Transcript_36/51_Confidence_0.402_Length_3407, vo_n2_Locus_386_Transcript_5/33_Confidence_0.040_Length_566, tri_n_comp19507_c0_seq6, vo_n1_Locus_3448_Transcript_55/64_Confidence_0.233_Length_3564, vo_c3_Locus_7050_Transcript_5/6_Confidence_0.667_Length_1047, tri_c_comp23673_c0_seq1, vo_c3_Locus_4005_Transcript_5/6_Confidence_0.167_Length_1517, vo_c1_Locus_6811_Transcript_7/8_Confidence_0.364_Length_589, vo_n2_Locus_297_Transcript_2/5_Confidence_0.636_Length_1679, vo_c2_Locus_1080_Transcript_4/21_Confidence_0.241_Length_1741, vo_c2_Locus_1080_Transcript_3/21_Confidence_0.207_Length_1702, vo_n3_Locus_1253_Transcript_1/2_Confidence_0.500_Length_1626, vo_c2_Locus_1080_Transcript_8/21_Confidence_0.138_Length_1077, vo_c2_Locus_4581_Transcript_3/8_Confidence_0.684_Length_1578, vo_n2_Locus_901_Transcript_1/95_Confidence_0.023_Length_1512, vo_n2_Locus_20307_Transcript_6/7_Confidence_0.062_Length_2030, vo_n3_Locus_1253_Transcript_2/2_Confidence_0.000_Length_1587, vo_n2_Locus_1937_Transcript_10/11_Confidence_0.615_Length_3407, vo_n1_Locus_1386_Transcript_4/9_Confidence_0.688_Length_3621, vo_n1_Locus_1386_Transcript_9/9_Confidence_0.250_Length_2403, vo_n1_Locus_1386_Transcript_5/9_Confidence_0.625_Length_3599, vo_n1_Locus_1386_Transcript_7/9_Confidence_0.438_Length_3591, vo_n3_Locus_12585_Transcript_4/4_Confidence_0.000_Length_3579, vo_n2_Locus_1937_Transcript_10/11_Confidence_0.615_Length_3407, vo_n1_Locus_1386_Transcript_4/9_Confidence_0.688_Length_3621, vo_n1_Locus_1386_Transcript_9/9_Confidence_0.250_Length_2403, vo_n1_Locus_1386_Transcript_5/9_Confidence_0.625_Length_3599, vo_n1_Locus_1386_Transcript_7/9_Confidence_0.438_Length_3591, vo_n3_Locus_12585_Transcript_4/4_Confidence_0.000_Length_3579, vo_c2_Locus_1643_Transcript_1/1_Confidence_0.000_Length_1756, vo_n2_Locus_634_Transcript_4/6_Confidence_0.357_Length_8557, tri_c_comp57035_c0_seq1, vo_c1_Locus_1809_Transcript_1/1_Confidence_0.000_Length_3087, vo_c3_Locus_18718_Transcript_1/1_Confidence_0.000_Length_2686, vo_c1_Locus_1055_Transcript_9/11_Confidence_0.357_Length_2035, vo_n1_Locus_561_Transcript_9/10_Confidence_0.462_Length_2182, vo_n2_Locus_3954_Transcript_2/8_Confidence_0.478_Length_2452, vo_c3_Locus_289_Transcript_1/7_Confidence_0.381_Length_2114,

					vo_n2_Locus_3954_Transcript_4/8_Confidence_0.435_Length_2452, vo_n1_Locus_561_Transcript_7/10_Confidence_0.385_Length_2182, vo_c1_Locus_1055_Transcript_7/11_Confidence_0.357_Len

Purine metabolism	map00230	49	ec:6.3.3.1 - cyclo-ligase, ec:2.7.4.8 - kinase, ec:2.7.4.6 - kinase, ec:2.7.4.3 - kinase, ec:3.5.2.5 - ec:3.5.2.5 allantoinase, ec:4.1.1.21 - carboxylase, ec:3.1.4.17 - phosphodiesterase, ec:3.5.4.4 - deaminase, ec:4.3.2.2 - lyase, ec:3.5.4.2 - deaminase, ec:3.5.4.3 - deaminase, ec:2.7.1.40 - kinase, ec:3.5.4.6 - deaminase, ec:3.1.4.35 - phosphodiesterase, ec:2.7.1.25 - kinase, ec:2.7.1.20 - kinase, ec:2.7.6.1 - diphosphokinase, ec:4.6.1.1 - cyclase, ec:4.6.1.2 - cyclase, ec:3.1.3.5 - uridine 5'-nucleotidase, ec:6.3.5.2 - synthase (glutamine-hydrolysing), ec:6.3.5.3 - synthase, ec:5.4.99.18 - ribonucleotide mutase, ec:3.5.1.5 - ec:3.5.1.5 urease, ec:2.1.2.3 - formyltransferase, ec:2.1.2.2 - formyltransferase, ec:6.3.4.4 - synthase, ec:3.6.1.3 - adenylpyrophosphatase, ec:3.6.1.6 - diphosphate phosphatase, ec:3.6.1.8 - diphosphatase, ec:3.6.1.9 - diphosphatase, ec:1.17.4.1 - reductase, ec:6.3.2.6 - synthase, ec:3.6.1.13 - diphosphatase, ec:3.6.1.15 - phosphatase, ec:2.7.7.4 - adenylyltransferase, ec:3.6.1.21 - diphosphatase, ec:3.6.1.29 - dinucleosidetriphosphatase, ec:2.7.7.6 - RNA polymerase, ec:2.7.7.7 - DNA polymerase, ec:6.3.4.13 - ligase, ec:3.5.2.17 - hydrolase, ec:3.2.2.1 - nucleosidase, ec:1.1.1.205 - dehydrogenase, ec:5.4.2.2 - (alpha-D-glucose-1,6-bisphosphate-dependent),	4, 2, 20, 10, 1, 1, 26, 1, 1, 1, 1, 25, 3, 28, 2, 3, 4, 6, 10, 11, 11, 4, 1, 1, 8, 1, 1, 334, 7, 1, 7, 32, 5, 3, 54, 7, 5, 1, 24, 30, 5, 2, 1, 12, 7, 1, 8, 1, 1	vo_c2_Locus_14099_Transcript_8/21_Confidence_0.413_Length_6648, vo_c1_Locus_1785_Transcript_2/4_Confidence_0.667_Length_6496, vo_c2_Locus_14099_Transcript_2/21_Confidence_0.444_Length_6577, vo_c3_Locus_4727_Transcript_17/25_Confidence_0.456_Length_6641, vo_n2_Locus_25289_Transcript_2/2_Confidence_0.000_Length_331, vo_c1_Locus_9429_Transcript_1/1_Confidence_0.000_Length_2416, vo_c1_Locus_13409_Transcript_2/6_Confidence_0.286_Length_871, vo_c3_Locus_2169_Transcript_1/3_Confidence_0.167_Length_848, vo_n2_Locus_739_Transcript_8/8_Confidence_0.167_Length_1338, vo_n3_Locus_4095_Transcript_2/3_Confidence_0.444_Length_3401, vo_c1_Locus_12423_Transcript_2/2_Confidence_0.333_Length_980, vo_c1_Locus_25242_Transcript_1/1_Confidence_0.000_Length_1465, vo_c1_Locus_4920_Transcript_1/2_Confidence_0.714_Length_1520, tri_c_comp49412_c0_seq1, vo_n2_Locus_5279_Transcript_1/1_Confidence_0.333_Length_2493, vo_n2_Locus_20723_Transcript_1/1_Confidence_0.000_Length_1179, vo_n3_Locus_24634_Transcript_1/1_Confidence_0.000_Length_445, vo_n1_Locus_5513_Transcript_4/4_Confidence_0.286_Length_1053, tri_c_comp24673_c0_seq1, tri_c_comp5012_c0_seq2, vo_c3_Locus_3080_Transcript_1/28_Confidence_0.009_Length_1584, vo_n1_Locus_9563_Transcript_1/1_Confidence_0.000_Length_1437, vo_n2_Locus_26604_Transcript_1/2_Confidence_0.750_Length_1361, vo_n2_Locus_2689_Transcript_2/2_Confidence_0.000_Length_2612, vo_c2_Locus_4541_Transcript_2/6_Confidence_0.571_Length_909, vo_n2_Locus_26604_Transcript_2/2_Confidence_0.000_Length_1685, vo_c2_Locus_10361_Transcript_6/7_Confidence_0.250_Length_1220, tri_c_comp24673_c0_seq1, vo_n1_Locus_2451_Transcript_1/3_Confidence_0.600_Length_2348, vo_c3_Locus_4102_Transcript_1/1_Confidence_0.000_Length_1034, vo_n2_Locus_22654_Transcript_1/1_Confidence_0.000_Length_691, vo_n1_Locus_2897_Transcript_2/14_Confidence_0.160_Length_971, vo_n2_Locus_14542_Transcript_1/3_Confidence_0.200_Length_924, vo_n3_Locus_12191_Transcript_1/1_Confidence_0.000_Length_1671, vo_n2_Locus_5279_Transcript_1/1_Confidence_0.333_Length_2493, vo_n3_Locus_24634_Transcript_1/1_Confidence_0.000_Length_445, vo_c1_Locus_16752_Transcript_1/1_Confidence_0.000_Length_1631, tri_c_comp23673_c0_seq1, vo_c1_Locus_11234_Transcript_1/1_Confidence_0.000_Length_3551, vo_n2_Locus_25869_Transcript_2/4_Confidence_0.667_Length_3997, vo_n3_Locus_2145_Transcript_70/88_Confidence_0.066_Length_3701, vo_c1_Locus_1289_Transcript_271/281_Confidence_0.010_Length_799, vo_n2_Locus_13038_Transcript_3/3_Confidence_0.000_Length_657, tri_n_comp20612_c0_seq41, tri_n_comp20612_c0_seq20, tri_c_comp41695_c0_seq1, vo_n2_Locus_8329_Transcript_2/4_Confidence_0.429_Length_5800, vo_n2_Locus_486_Transcript_5/19_Confidence_0.109_Length_1729, tri_n_comp20612_c0_seq23, vo_c1_Locus_1289_Transcript_276/281_Confidence_0.007_Length_682, vo_n1_Locus_2298_Transcript_8/8_Confidence_0.000_Length_405, vo_n2_Locus_17431_Transcript_1/1_Confidence_0.000_Length_3247, vo_n1_Locus_16872_Transcript_5/10_Confidence_0.467_Length_541, vo_c1_Locus_10943_Transcript_3/3_Confidence_0.167_Length_2079, tri_n_comp20612_c0_seq32, vo_c2_Locus_16226_Transcript_1/1_Confidence_0.500_Length_2623, vo_c3_Locus_71_Transcript_42/86_Confidence_0.049_Length_3035, vo_n2_Locus_16680_Transcript_1/2_Confidence_1.000_Length_1706, vo_c3_Locus_71_Transcript_44/86_Confidence_0.034_Length_2679, tri_n_comp20612_c0_seq19, vo_n3_Locus_14014_Transcript_3/7_Confidence_0.400_Length_1850, vo_c1_Locus_5298_Transcript_2/3_Confidence_0.400_Length_2630, vo_c2_Locus_28848_Transcript_1/1_Confidence_0.750_Length_2971, vo_n3_Locus_589_Transcript_1/1_Confidence_0.000_Length_2005, tri_n_comp92826_c0_seq1, vo_n2_Locus_9167_Transcript_1/4_Confidence_0.286_Length_6748, vo_n2_Locus_13025_Transcript_1/1_Confidence_0.000_Length_1235, tri_c_comp58344_c0_seq1, vo_n2_Locus_9037_Transcript_9/15_Confidence_0.486_Length_4553, vo_c2_Locus_684_Transcript_7/16_Confidence_0.194_Length_2803, vo_n3_Locus_2293_Transcript_1/2_Confidence_0.750_Length_1794, vo_n3_Locus_1756_Transcript_10/15_Confidence_0.393_Length_2226, vo_c3_Locus_725_Transcript_6/15_Confidence_0.548_Length_5086, vo_n3_Locus_1756_Transcript_7/15_Confidence_0.250_Length_2226, vo_n2_Locus_16147_Transcript_1/1_Confidence_0.000_Length_1452, vo_n2_Locus_9037_Transcript_2/15_Confidence_0.257_Length_3951, vo_n2_Locus_9037_Transcript_13/15_Confidence_0.143_Length_1729, vo_n2_Locus_9037_Transcript_5/15_Confidence_0.286_Length_2279, vo_n2_Locus_9037_Transcript_6/15_Confidence_0.229_Length_2279, tri_c_comp19644_c0_seq1, vo_n2_Locus_171_Transcript_17/42_Confidence_0.229_Length_1803, tri_c_comp19644_c0_seq4, vo_c3_Locus_12256_Transcript_3/3_Confidence_0.000_Length_508, vo_c3_Locus_725_Transcript_12/15_Confidence_0.290_Length_3194, vo_c1_Locus_6543_Transcript_5/23_Confidence_0.220_Length_2294, vo_n2_Locus_9037_Transcript_12/15_Confidence_0.257_Length_2318, vo_c3_Locus_725_Transcript_13/15_Confidence_0.452_Length_4249, tri_n_comp19211_c0_seq1, vo_c3_Locus_883_Transcript_1/2_Confidence_1.000_Length_1783, vo_n2_Locus_9037_Transcript_1/15_Confidence_0.143_Length_1677, vo_c3_Locus_307_Transcript_2/21_Confidence_0.576_Length_1809, vo_n1_Locus_41_Transcript_20/90_Confidence_0.068_Length_2175, vo_c2_Locus_1667_Transcript_21/21_Confidence_0.195_Length_1788, tri_c_comp80018_c0_seq1, vo_c1_Locus_37084_Transcript_1/2_Confidence_0.667_Length_2193, vo_c3_Locus_20892_Transcript_1/1_Confidence_0.000_Length_2501, vo_c1_Locus_11369_Transcript_2/3_Confidence_0.944_Length_3455, vo_c1_Locus_15657_Transcript_1/1_Confidence_0.000_Length_3265, vo_c3_Locus_8108_Transcript_1/1_Confidence_0.000_Length_1594, vo_n1_Locus_1533_Transcript_3/6_Confidence_0.167_Length_1394, vo_n2_Locus_3406_Transcript_3/4_Confidence_0.571_Length_827, vo_c2_Locus_17150_Transcript_26/102_Confidence_0.048_Length_1638, tri_n_comp50886_c0_seq1, tri_n_comp20612_c0_seq3, tri_c_comp38877_c0_seq1, vo_n1_Locus_2298_Transcript_3/8_Confidence_0.211_Length_3234, tri_n_comp20612_c0_seq5, tri_n_comp45457_c0_seq1, vo_n1_Locus_10882_Transcript_3/3_Confidence_0.200_Length_793, vo_n2_Locus_18247_Transcript_2/4_Confidence_0.333_Length_4047, vo_n1_Locus_3276_Transcript_1/2_Confidence_0.964_Length_3472, vo_n2_Locus_21555_Transcript_1/1_Confidence_0.000_Length_2269, vo_n2_Locus_1392_Transcript_1/1_Confidence_0.000_Length_3210, vo_n2_Locus_3406_Transcript_4/4_Confidence_0.286_Length_731, vo_n2_Locus_4835_Transcript_1/1_Confidence_0.000_Length_3087, vo_c2_Locus_18667_Transcript_1/2_Confidence_0.947_Length_3406, vo_n3_Locus_19488_Transcript_2/3_Confidence_0.958_Length_3497,

			ec:5.4.2.7 - phosphodeoxyribomutase, ec:3.5.4.10 - cyclohydrolase, ec:2.4.2.7 - phosphoribosyltransferase, ec:2.7.4.10 - kinase		tri_c_comp20307_c0_seq9, vo_c3_Locus_8654_Transcript_8/9_Confidence_0.133_Length_2413, tri_c_comp20307_c0_seq3, vo_n1_Locus_2298_Transcript_1/8_Confidence_0.368_Length_3186, vo_c2_Locus_18670_Transcript_1/1_Confidence_0.000_Length_6956, vo_c2_Locus_4106_Transcript_3/4_Confidence_0.429_Length_3178, vo_c3_Locus_8439_Transcript_2/2_Confidence_0.000_Length_3521, vo_c1_Locus_18424_Transcript_2/2_Confidence_0.000_Length_956, vo_c1_Locus_18424_Transcript_1/2_Confidence_0.750_Length_906, vo_n3_Locus_1405_Transcript_3/5_Confidence_0.500_Length_2519, vo_c1_Locus_11931_Transcript_1/1_Confidence_0.000_Length_1187, vo_n3_Locus_1405_Transcript_2/5_Confidence_0.667_Length_2492, vo_c3_Locus_18398_Transcript_2/2_Confidence_0.000_Length_715, vo_n2_Locus_18825_Transcript_1/3_Confidence_0.500_Length_1632, tri_c_comp33638_c0_seq1, vo_n2_Locus_8943_Transcript_1/2_Confidence_0.750_Length_1351, vo_n2_Locus_22854_Transcript_4/5_Confidence_0.333_Length_897, vo_c3_Locus_12814_Transcript_1/1_Confidence_0.000_Length_3781, tri_n_comp43454_c0_seq1, vo_c3_Locus_18559_Transcript_2/5_Confidence_0.400_Length_3906, vo_n1_Locus_7807_Transcript_1/1_Confidence_0.000_Length_2655, tri_c_comp38249_c0_seq1, vo_c1_Locus_2643_Transcript_1/1_Confidence_0.000_Length_3649, tri_c_comp15408_c0_seq1, tri_n_comp18351_c0_seq4, vo_c1_Locus_2409_Transcript_4/7_Confidence_0.444_Length_1597, vo_c3_Locus_37877_Transcript_1/1_Confidence_0.000_Length_862, tri_c_comp21242_c0_seq12, vo_c1_Locus_1548_Transcript_3/4_Confidence_0.429_Length_6787, tri_n_comp83886_c0_seq1, vo_c1_Locus_14933_Transcript_2/4_Confidence_0.429_Length_4839, vo_c3_Locus_31_Transcript_59/79_Confidence_0.018_Length_4351, vo_c1_Locus_21072_Transcript_1/1_Confidence_0.000_Length_965, vo_c2_Locus_25862_Transcript_4/4_Confidence_0.000_Length_988, vo_c2_Locus_26482_Transcript_5/6_Confidence_0.286_Length_1654, vo_c2_Locus_26482_Transcript_4/6_Confidence_0.571_Length_1609, vo_n2_Locus_21191_Transcript_4/6_Confidence_0.300_Length_1691, vo_c1_Locus_18480_Transcript_2/2_Confidence_0.000_Length_1013, vo_c2_Locus_27118_Transcript_12/49_Confidence_0.025_Length_3122, vo_c2_Locus_6520_Transcript_5/15_Confidence_0.455_Length_1419, vo_c3_Locus_7168_Transcript_5/8_Confidence_0.538_Length_1343, vo_n1_Locus_7057_Transcript_3/15_Confidence_0.611_Length_1294, vo_n2_Locus_21191_Transcript_3/6_Confidence_0.500_Length_1646, vo_n1_Locus_9681_Transcript_4/11_Confidence_0.529_Length_1748, vo_n2_Locus_215_Transcript_14/28_Confidence_0.182_Length_1973, vo_n3_Locus_2366_Transcript_13/24_Confidence_0.236_Length_1948, vo_n1_Locus_9681_Transcript_6/11_Confidence_0.353_Length_1721, vo_n2_Locus_215_Transcript_12/28_Confidence_0.212_Length_2644, vo_n1_Locus_9681_Transcript_11/11_Confidence_0.000_Length_529, vo_n1_Locus_9681_Transcript_5/11_Confidence_0.529_Length_1790, vo_n2_Locus_215_Transcript_22/28_Confidence_0.197_Length_2480, vo_n2_Locus_215_Transcript_8/28_Confidence_0.227_Length_2670, vo_c1_Locus_1789_Transcript_29/32_Confidence_0.300_Length_3220, vo_n1_Locus_6239_Transcript_3/19_Confidence_0.425_Length_1997, vo_c3_Locus_7565_Transcript_10/13_Confidence_0.400_Length_4317, tri_c_comp16509_c0_seq1, vo_c3_Locus_7565_Transcript_1/13_Confidence_0.375_Length_4301, vo_n1_Locus_4052_Transcript_3/24_Confidence_0.524_Length_4370, tri_c_comp23673_c0_seq1, tri_c_comp36137_c0_seq1, vo_c1_Locus_1055_Transcript_9/11_Confidence_0.357_Length_2035, vo_n1_Locus_561_Transcript_9/10_Confidence_0.462_Length_2182, vo_n2_Locus_3954_Transcript_2/8_Confidence_0.478_Length_2452, vo_c3_Locus_289_Transcript_1/7_Confidence_0.381_Length_2114, vo_n2_Locus_3954_Transcript_4/8_Confidence_0.435_Length_2452, vo_n1_Locus_561_Transcript_7/10_Confidence_0.385_Length_2182, vo_c1_Locus_1055_Transcript_7/11_Confidence_0.357_Length_2063, vo_n1_Locus_561_Transcript_1/10_Confidence_0.615_Length_2142, vo_n2_Locus_3094_Transcript_1/1_Confidence_0.000_Length_2179, tri_n_comp32103_c0_seq1, vo_c1_Locus_31_Transcript_975/1155_Confidence_0.009_Length_2875, vo_n2_Locus_5517_Transcript_2/6_Confidence_0.500_Length_15215, vo_n3_Locus_3296_Transcript_2/3_Confidence_0.105_Length_2093, vo_n1_Locus_31401_Transcript_1/1_Confidence_0.000_Length_3290, vo_n2_Locus_3_Transcript_135/24291_Confidence_0.000_Length_1423, tri_c_comp22421_c0_seq1, vo_n2_Locus_25738_Transcript_1/2_Confidence_0.750_Length_2303, vo_n3_Locus_12111_Transcript_1/1_Confidence_0.000_Length_3744, vo_c2_Locus_555_Transcript_1/1_Confidence_0.500_Length_2228, vo_c3_Locus_5_Transcript_149/353_Confidence_0.048_Length_4156, vo_c3_Locus_5_Transcript_329/353_Confidence_0.010_Length_1383, vo_n3_Locus_4760_Transcript_4/5_Confidence_0.067_Length_1112, vo_c3_Locus_7645_Transcript_5/8_Confidence_0.526_Length_2659, vo_n3_Locus_427_Transcript_145/175_Confidence_0.110_Length_3547, vo_n3_Locus_81_Transcript_40/54_Confidence_0.354_Length_2989, tri_c_comp39238_c0_seq1, vo_c3_Locus_15013_Transcript_2/4_Confidence_0.800_Length_2706, vo_n1_Locus_19766_Transcript_2/4_Confidence_0.500_Length_3906, vo_n2_Locus_7964_Transcript_6/11_Confidence_0.650_Length_3693, vo_n2_Locus_3_Transcript_6590/24291_Confidence_0.000_Length_2496, tri_n_comp19803_c0_seq4, tri_n_comp20613_c0_seq2, vo_c2_Locus_3932_Transcript_1/1_Confidence_0.000_Length_1456, vo_n2_Locus_88_Transcript_3/3_Confidence_0.250_Length_876, vo_n3_Locus_81_Transcript_8/54_Confidence_0.415_Length_3794, vo_n3_Locus_11156_Transcript_9/11_Confidence_0.500_Length_6082, vo_c1_Locus_4849_Transcript_14/16_Confidence_0.259_Length_2445, vo_c1_Locus_8562_Transcript_1/1_Confidence_0.000_Length_2414, vo_c1_Locus_5480_Transcript_2/9_Confidence_0.312_Length_693, vo_c2_Locus_579_Transcript_160/548_Confidence_0.016_Length_1203, vo_n3_Locus_7390_Transcript_2/3_Confidence_0.400_Length_4559, vo_n3_Locus_87_Transcript_4/8_Confidence_0.400_Length_2639, vo_n2_Locus_3_Transcript_20035/24291_Confidence_0.000_Length_2188, tri_c_comp27724_c0_seq1, vo_n2_Locus_3_Transcript_6586/24291_Confidence_0.000_Length_257, vo_n2_Locus_3_Transcript_20537/24291_Confidence_0.000_Length_3437, vo_n3_Locus_427_Transcript_118/175_Confidence_0.158_Length_3483, vo_c3_Locus_5_Transcript_27/353_Confidence_0.024_Length_1533, vo_c1_Locus_247_Transcript_53/92_Confidence_0.147_Length_2668, vo_n2_Locus_3_Transcript_20499/24291_Confidence_0.000_Length_5167, tri_n_comp20573_c1_seq9, tri_n_comp20573_c1_seq7, vo_n3_Locus_23289_Transcript_1/5_Confidence_0.429_Length_3154, tri_n_comp20573_c1_seq5, vo_c1_Locus_4_Transcript_358/366_Confidence_0.039_Length_986, vo_n3_Locus_427_Transcript_113/175_Confidence_0.081_Length_2714, tri_c_comp75769_c0_seq1, vo_c1_Locus_1558_Transcript_10/10_Confidence_0.225_Length_6627, vo_n2_Locus_3_Transcript_894/24291_Confidence_0.000_Length_542,

					tri_n_comp47775_c0_seq1, vo_n2_Locus_2545_Transcript_2/2_Confidence_0.000_Length_3134, vo_c3_Locus_32274_Transcript_3/4_Confidence_0.333_Length_1761, vo_n1_Locus_68_Transcript_33/39_Confidence_0.217_Length_1672, vo_c1_Locus_31_Transcript_986/1155_Confidence_0.003_Length_1716, vo_n2_Locus_3_Transcript_6579/24291_Confidence_0.000_Length_1740, vo_c2_Locus_52_Transcript_314/466_Confidence_0.012_Length_1560, vo_n2_Locus_2078_Transcript_1/1_Confidence_0.000_Length_2771, tri_c_comp20681_c1_seq2, vo_n1_Locus_7216_Transcript_3/5_Confidence_0.410_Length_13054, vo_n2_Locus_3423_Transcript_2/10_Confidence_0.667_Length_2090, tri_n_comp23721_c0_seq1, vo_n2_Locus_14944_Transcript_3/6_Confidence_0.625_Length_1989, tri_n_comp61222_c0_seq1, vo_c3_Locus_1299_Transcript_4/15_Confidence_0.556_Length_2256, vo_c1_Locus_18050_Transcript_18/18_Confidence_0.000_Length_2637, vo_c3_Locus_16970_Transcript_1/1_Confidence_0.000_Length_1358, vo_n2_Locus_25592_Transcript_1/2_Confidence_0.333_Length_931, vo_n2_Locus_3_Transcript_6490/24291_Confidence_0.000_Length_1110, tri_c_comp2918_c0_seq1, vo_c3_Locus_7131_Transcript_3/18_Confidence_0.333_Length_7767, tri_c_comp62474_c0_seq1, vo_c1_Locus_2349_Transcript_14/17_Confidence_0.588_Length_4132, vo_n2_Locus_3_Transcript_198/24291_Confidence_0.000_Length_734, vo_c3_Locus_2745_Transcript_3/8_Confidence_0.545_Length_2262, vo_n2_Locus_3_Transcript_2146/24291_Confidence_0.000_Length_8136, vo_c2_Locus_16170_Transcript_2/2_Confidence_0.000_Length_417, vo_n2_Locus_336_Transcript_5/9_Confidence_0.318_Length_1829, vo_c2_Locus_579_Transcript_125/548_Confidence_0.024_Length_1059, vo_n1_Locus_1858_Transcript_419/440_Confidence_0.022_Length_537, vo_c2_Locus_1543_Transcript_18/29_Confidence_0.425_Length_3326, vo_c2_Locus_1543_Transcript_26/29_Confidence_0.575_Length_3718, vo_c1_Locus_9448_Transcript_1/1_Confidence_0.000_Length_1462, vo_n1_Locus_7662_Transcript_1/2_Confidence_0.967_Length_3451, vo_n2_Locus_7754_Transcript_1/3_Confidence_0.167_Length_1808, vo_c1_Locus_31_Transcript_974/1155_Confidence_0.009_Length_2112, vo_n1_Locus_16407_Transcript_1/11_Confidence_0.400_Length_2360, tri_n_comp20616_c0_seq4, vo_c2_Locus_3834_Transcript_23/50_Confidence_0.062_Length_3121, vo_n1_Locus_68_Transcript_34/39_Confidence_0.075_Length_2855, vo_n1_Locus_16_Transcript_107/225_Confidence_0.016_Length_2544, vo_c3_Locus_7216_Transcript_3/5_Confidence_0.333_Length_2942, vo_n1_Locus_586_Transcript_15/171_Confidence_0.017_Length_4099, vo_n2_Locus_3_Transcript_6527/24291_Confidence_0.000_Length_14582, tri_c_comp43067_c0_seq1, vo_c1_Locus_26083_Transcript_4/7_Confidence_0.545_Length_2333, vo_c3_Locus_17678_Transcript_1/2_Confidence_0.667_Length_1546, vo_n2_Locus_3_Transcript_195/24291_Confidence_0.000_Length_447, tri_c_comp6033_c0_seq1, vo_n2_Locus_15403_Transcript_1/2_Confidence_0.667_Length_1439, tri_n_comp12113_c0_seq1, vo_n1_Locus_693_Transcript_1/16_Confidence_0.200_Length_1520, vo_c3_Locus_11591_Transcript_2/3_Confidence_0.571_Length_11254, vo_c2_Locus_10236_Transcript_3/20_Confidence_0.150_Length_1941, vo_n2_Locus_3_Transcript_280/24291_Confidence_0.000_Length_1356, vo_c3_Locus_1766_Transcript_1/1_Confidence_0.000_Length_1152, vo_n1_Locus_1055_Transcript_1/31_Confidence_0.419_Length_3579, vo_c3_Locus_13234_Transcript_1/4_Confidence_0.600_Length_4487, vo_c1_Locus_4_Transcript_269/366_Confidence_0.017_Length_689, vo_n1_Locus_1858_Transcript_417/440_Confidence_0.011_Length_549, vo_c3_Locus_5_Transcript_311/353_Confidence_0.038_Length_3204, vo_n1_Locus_3_Transcript_42/53_Confidence_0.250_Length_15193, vo_c1_Locus_44080_Transcript_1/1_Confidence_0.500_Length_170, vo_c2_Locus_947_Transcript_3/5_Confidence_0.667_Length_3262, vo_c2_Locus_13950_Transcript_1/1_Confidence_0.500_Length_2942, vo_c3_Locus_363_Transcript_3/24_Confidence_0.238_Length_3160, vo_n1_Locus_6445_Transcript_1/17_Confidence_0.143_Length_1951, vo_c3_Locus_33664_Transcript_2/2_Confidence_0.000_Length_2232, vo_n1_Locus_7700_Transcript_1/1_Confidence_0.000_Length_2536, tri_n_comp37528_c0_seq1, vo_n3_Locus_81_Transcript_42/54_Confidence_0.245_Length_2948, vo_c2_Locus_52_Transcript_346/466_Confidence_0.005_Length_2463, vo_n2_Locus_3_Transcript_6537/24291_Confidence_0.000_Length_2637, tri_n_comp20775_c0_seq2, tri_n_comp94388_c0_seq1, vo_c2_Locus_5794_Transcript_2/2_Confidence_0.000_Length_2377, tri_n_comp20775_c0_seq8, vo_c2_Locus_52_Transcript_305/466_Confidence_0.047_Length_2671, vo_c1_Locus_31_Transcript_981/1155_Confidence_0.012_Length_2636, vo_c2_Locus_1956_Transcript_2/3_Confidence_0.200_Length_861, vo_n3_Locus_81_Transcript_15/54_Confidence_0.340_Length_3575, vo_n1_Locus_429_Transcript_17/19_Confidence_0.483_Length_2215, vo_n2_Locus_3_Transcript_6543/24291_Confidence_0.000_Length_3424, vo_c3_Locus_3164_Transcript_96/111_Confidence_0.009_Length_11338, vo_n2_Locus_10873_Transcript_1/2_Confidence_0.333_Length_1429, vo_n2_Locus_3748_Transcript_2/7_Confidence_0.500_Length_14045, vo_n3_Locus_427_Transcript_117/175_Confidence_0.093_Length_2715, vo_c1_Locus_247_Transcript_67/92_Confidence_0.147_Length_2373, vo_n1_Locus_21852_Transcript_2/2_Confidence_0.167_Length_2557, tri_n_comp14883_c0_seq1, tri_n_comp18105_c0_seq3, vo_c1_Locus_4_Transcript_289/366_Confidence_0.028_Length_1095, vo_c1_Locus_31_Transcript_949/1155_Confidence_0.002_Length_2152, vo_c3_Locus_5_Transcript_126/353_Confidence_0.019_Length_4217, tri_c_comp38221_c0_seq1, vo_c3_Locus_24981_Transcript_15/16_Confidence_0.015_Length_2313, vo_n1_Locus_7307_Transcript_14/44_Confidence_0.145_Length_1234, vo_c3_Locus_1323_Transcript_7/23_Confidence_0.455_Length_3889, vo_c3_Locus_30043_Transcript_1/3_Confidence_0.444_Length_2397, vo_c3_Locus_4508_Transcript_1/7_Confidence_0.438_Length_2333, vo_c1_Locus_5803_Transcript_20/21_Confidence_0.417_Length_2594, vo_n1_Locus_5525_Transcript_6/11_Confidence_0.133_Length_4294, tri_c_comp41265_c0_seq1, vo_n2_Locus_14056_Transcript_2/3_Confidence_0.333_Length_2513, vo_c1_Locus_4993_Transcript_4/4_Confidence_0.143_Length_5162, vo_n3_Locus_427_Transcript_54/175_Confidence_0.090_Length_2679, vo_n3_Locus_81_Transcript_41/54_Confidence_0.320_Length_3232, vo_n2_Locus_17878_Transcript_2/2_Confidence_0.800_Length_3026, vo_n1_Locus_2635_Transcript_18/21_Confidence_0.156_Length_2310, vo_c2_Locus_1611_Transcript_19/22_Confidence_0.057_Length_1197, vo_n2_Locus_3_Transcript_6536/24291_Confidence_0.000_Length_2542, vo_c2_Locus_37_Transcript_72/305_Confidence_0.025_Length_3316, vo_n1_Locus_16_Transcript_57/225_Confidence_0.055_Length_2636, tri_c_comp18389_c0_seq7, vo_c2_Locus_52_Transcript_300/466_Confidence_0.033_Length_2317, tri_c_comp18389_c0_seq2,

					vo_c2_Locus_579_Transcript_123/548_Confidence_0.012_Length_999, vo_n2_Locus_6563_Transcript_1/1_Confidence_0.000_Length_2370, vo_c2_Locus_37_Transcript_51/305_Confidence_0.012_Length_2747, tri_n_comp64176_c0_seq1, tri_n_comp46870_c0_seq1, vo_c2_Locus_52_Transcript_309/466_Confidence_0.036_Length_2654, vo_n1_Locus_782_Transcript_2/2_Confidence_0.000_Length_2820, tri_c_comp55454_c0_seq1, vo_c2_Locus_4906_Transcript_1/6_Confidence_0.320_Length_10739, vo_n1_Locus_13939_Transcript_2/2_Confidence_0.000_Length_2279, tri_n_comp52479_c0_seq1, vo_n1_Locus_1404_Transcript_8/51_Confidence_0.273_Length_9488, vo_c2_Locus_579_Transcript_203/548_Confidence_0.006_Length_4691, vo_c1_Locus_13654_Transcript_2/4_Confidence_0.846_Length_2821, vo_c3_Locus_363_Transcript_22/24_Confidence_0.095_Length_1049, vo_c2_Locus_21966_Transcript_2/3_Confidence_0.333_Length_4811, vo_c3_Locus_5_Transcript_118/353_Confidence_0.045_Length_3310, vo_n2_Locus_3748_Transcript_1/7_Confidence_0.500_Length_14018, vo_c1_Locus_4_Transcript_278/366_Confidence_0.036_Length_1569, vo_c3_Locus_150_Transcript_4/4_Confidence_0.429_Length_2271, tri_n_comp46298_c0_seq1, vo_n1_Locus_68_Transcript_10/39_Confidence_0.325_Length_2921, vo_n3_Locus_11413_Transcript_1/1_Confidence_0.000_Length_2938, vo_c3_Locus_5_Transcript_140/353_Confidence_0.035_Length_3043, vo_c3_Locus_7131_Transcript_13/18_Confidence_0.500_Length_1394, vo_n2_Locus_3_Transcript_6585/24291_Confidence_0.001_Length_4455, vo_n1_Locus_14212_Transcript_1/3_Confidence_0.750_Length_12890, vo_n2_Locus_14045_Transcript_1/1_Confidence_0.000_Length_2252, vo_c3_Locus_5_Transcript_308/353_Confidence_0.047_Length_3138, vo_c2_Locus_1787_Transcript_5/9_Confidence_0.450_Length_3889, vo_n3_Locus_8653_Transcript_4/4_Confidence_0.125_Length_2635, vo_n2_Locus_8606_Transcript_2/2_Confidence_0.333_Length_4357, vo_c3_Locus_5_Transcript_120/353_Confidence_0.032_Length_2041, vo_n1_Locus_3552_Transcript_1/1_Confidence_0.000_Length_2088, vo_n3_Locus_4760_Transcript_3/5_Confidence_0.778_Length_2904, vo_n2_Locus_1515_Transcript_18/24_Confidence_0.026_Length_2573, vo_c2_Locus_9860_Transcript_3/3_Confidence_0.167_Length_3453, vo_n1_Locus_68_Transcript_25/39_Confidence_0.283_Length_2926, vo_n1_Locus_26316_Transcript_1/1_Confidence_0.000_Length_4173, vo_c1_Locus_19255_Transcript_1/1_Confidence_0.000_Length_7213, tri_c_comp24491_c0_seq1, vo_c1_Locus_25755_Transcript_1/1_Confidence_0.000_Length_1907, vo_c3_Locus_5_Transcript_153/353_Confidence_0.018_Length_3233, vo_n2_Locus_986_Transcript_4/33_Confidence_0.366_Length_2850, vo_n1_Locus_6445_Transcript_16/17_Confidence_0.114_Length_1048, vo_c3_Locus_6617_Transcript_2/3_Confidence_0.400_Length_2184, vo_n2_Locus_3_Transcript_20530/24291_Confidence_0.000_Length_3785, tri_n_comp6725_c0_seq1, vo_c1_Locus_26083_Transcript_7/7_Confidence_0.000_Length_484, vo_n1_Locus_34601_Transcript_1/1_Confidence_0.000_Length_2147, vo_c2_Locus_17150_Transcript_4/102_Confidence_0.039_Length_466, vo_n1_Locus_68_Transcript_13/39_Confidence_0.300_Length_2992, tri_n_comp51573_c0_seq1, vo_c3_Locus_713_Transcript_6/49_Confidence_0.139_Length_4520, vo_n3_Locus_427_Transcript_114/175_Confidence_0.081_Length_2387, vo_c2_Locus_5066_Transcript_1/2_Confidence_0.968_Length_2042, vo_c2_Locus_324_Transcript_11/12_Confidence_0.333_Length_4393, vo_c2_Locus_52_Transcript_310/466_Confidence_0.036_Length_2651, vo_n3_Locus_427_Transcript_136/175_Confidence_0.027_Length_886, vo_c2_Locus_579_Transcript_120/548_Confidence_0.013_Length_902, vo_c3_Locus_1299_Transcript_7/15_Confidence_0.444_Length_2262, vo_n1_Locus_4600_Transcript_4/22_Confidence_0.250_Length_5922, tri_n_comp5665_c0_seq1, vo_c1_Locus_4849_Transcript_10/16_Confidence_0.556_Length_2123, vo_c2_Locus_52_Transcript_336/466_Confidence_0.005_Length_4799, vo_n1_Locus_37234_Transcript_1/3_Confidence_1.000_Length_627, vo_n3_Locus_4760_Transcript_2/5_Confidence_0.044_Length_2212, tri_c_comp20278_c1_seq8, vo_c2_Locus_5809_Transcript_2/2_Confidence_0.250_Length_1859, vo_n1_Locus_869_Transcript_7/10_Confidence_0.605_Length_13092, vo_n3_Locus_18313_Transcript_1/1_Confidence_0.000_Length_1710, vo_n2_Locus_23536_Transcript_1/1_Confidence_0.000_Length_1634, vo_c2_Locus_579_Transcript_216/548_Confidence_0.006_Length_7997, vo_n3_Locus_81_Transcript_51/54_Confidence_0.197_Length_2262, tri_n_comp46526_c0_seq1, vo_c2_Locus_52_Transcript_318/466_Confidence_0.005_Length_3440, vo_n2_Locus_3_Transcript_20542/24291_Confidence_0.000_Length_3884, vo_n2_Locus_5350_Transcript_5/6_Confidence_0.375_Length_3890, vo_c2_Locus_40983_Transcript_3/3_Confidence_0.467_Length_271, vo_n2_Locus_25738_Transcript_2/2_Confidence_0.000_Length_2365, vo_c1_Locus_64_Transcript_5/17_Confidence_0.500_Length_2619, vo_n1_Locus_586_Transcript_20/171_Confidence_0.047_Length_1325, vo_c1_Locus_4849_Transcript_16/16_Confidence_0.222_Length_2335, vo_n1_Locus_5081_Transcript_2/2_Confidence_0.000_Length_1015, vo_c2_Locus_7918_Transcript_3/7_Confidence_0.333_Length_4982, vo_n3_Locus_81_Transcript_43/54_Confidence_0.347_Length_2521, tri_c_comp20278_c1_seq3, vo_n2_Locus_576_Transcript_1/11_Confidence_0.500_Length_2179, vo_c2_Locus_11169_Transcript_1/1_Confidence_0.000_Length_3106, vo_c1_Locus_247_Transcript_83/92_Confidence_0.091_Length_2168, vo_n1_Locus_331_Transcript_1/3_Confidence_0.864_Length_13100, tri_n_comp7525_c0_seq1, vo_n2_Locus_3_Transcript_8818/24291_Confidence_0.000_Length_11199, vo_c3_Locus_5_Transcript_122/353_Confidence_0.031_Length_2266, vo_n3_Locus_81_Transcript_3/54_Confidence_0.095_Length_356, tri_c_comp42419_c0_seq1, vo_n2_Locus_1836_Transcript_11/26_Confidence_0.553_Length_4022, vo_n2_Locus_1836_Transcript_9/26_Confidence_0.421_Length_3319, vo_c1_Locus_21246_Transcript_3/3_Confidence_0.000_Length_1335, vo_c3_Locus_5_Transcript_314/353_Confidence_0.041_Length_5162, vo_n3_Locus_81_Transcript_53/54_Confidence_0.122_Length_3108, vo_c3_Locus_11720_Transcript_1/2_Confidence_0.333_Length_4343, vo_c3_Locus_5_Transcript_139/353_Confidence_0.032_Length_2633, vo_n3_Locus_81_Transcript_38/54_Confidence_0.388_Length_3107, vo_n3_Locus_18043_Transcript_2/3_Confidence_0.667_Length_2705, vo_n2_Locus_3_Transcript_6549/24291_Confidence_0.000_Length_3661, vo_c1_Locus_4_Transcript_267/366_Confidence_0.015_Length_1391, vo_n3_Locus_9081_Transcript_1/2_Confidence_0.909_Length_2859, vo_c2_Locus_6435_Transcript_5/6_Confidence_0.032_Length_7385, vo_c2_Locus_12150_Transcript_7/14_Confidence_0.200_Length_5692, vo_n1_Locus_68_Transcript_23/39_Confidence_0.150_Length_2727, vo_c1_Locus_21235_Transcript_2/4_Confidence_0.625_Length_1588, vo_n3_Locus_427_Transcript_112/175_Confidence_0.081_Length_2772,

					tri_n_comp55121_c0_seq1, vo_c2_Locus_6519_Transcript_1/1_Confidence_0.000_Length_1138, vo_c1_Locus_31507_Transcript_2/2_Confidence_0.000_Length_3719, vo_n2_Locus_3_Transcript_6520/24291_Confidence_0.000_Length_2555, vo_c1_Locus_247_Transcript_89/92_Confidence_0.134_Length_2677, vo_c1_Locus_26083_Transcript_6/7_Confidence_0.091_Length_760, vo_c1_Locus_31191_Transcript_2/4_Confidence_0.810_Length_5131, vo_c2_Locus_7918_Transcript_1/7_Confidence_0.111_Length_5075, vo_n3_Locus_11624_Transcript_2/6_Confidence_0.500_Length_713, vo_c1_Locus_24254_Transcript_1/2_Confidence_0.667_Length_2432, vo_n1_Locus_7897_Transcript_10/12_Confidence_0.400_Length_12811, vo_n2_Locus_2647_Transcript_12/14_Confidence_0.579_Length_10184, vo_c2_Locus_703_Transcript_1/2_Confidence_0.333_Length_2334, tri_c_comp12118_c0_seq2, vo_c1_Locus_247_Transcript_71/92_Confidence_0.039_Length_2524, vo_c3_Locus_1323_Transcript_1/23_Confidence_0.045_Length_621, vo_n2_Locus_2314_Transcript_1/1_Confidence_0.667_Length_1996, vo_n1_Locus_68_Transcript_36/39_Confidence_0.267_Length_1533, vo_c1_Locus_31_Transcript_982/1155_Confidence_0.007_Length_2463, vo_n2_Locus_20021_Transcript_3/5_Confidence_0.688_Length_3229, vo_n2_Locus_3_Transcript_20541/24291_Confidence_0.000_Length_3818, vo_c3_Locus_5_Transcript_321/353_Confidence_0.051_Length_2976, vo_c3_Locus_324_Transcript_24/40_Confidence_0.286_Length_4286, vo_n2_Locus_12320_Transcript_1/3_Confidence_0.600_Length_1445, vo_n1_Locus_2635_Transcript_1/21_Confidence_0.406_Length_3170, tri_c_comp13353_c0_seq1, tri_n_comp20201_c0_seq3, vo_n2_Locus_7754_Transcript_3/3_Confidence_0.167_Length_2874, tri_c_comp13353_c0_seq2, tri_c_comp50556_c0_seq1, vo_c2_Locus_37_Transcript_76/305_Confidence_0.039_Length_3490, vo_c2_Locus_9988_Transcript_6/11_Confidence_0.611_Length_2701, vo_n3_Locus_3787_Transcript_3/6_Confidence_0.462_Length_2514, vo_n1_Locus_4600_Transcript_20/22_Confidence_0.250_Length_12970, vo_c2_Locus_579_Transcript_233/548_Confidence_0.006_Length_12924, vo_n1_Locus_26480_Transcript_1/1_Confidence_0.000_Length_2601, vo_n2_Locus_3_Transcript_20538/24291_Confidence_0.001_Length_4570, vo_n1_Locus_869_Transcript_5/10_Confidence_0.224_Length_12948, vo_n3_Locus_81_Transcript_44/54_Confidence_0.354_Length_2429, vo_n1_Locus_3_Transcript_53/53_Confidence_0.045_Length_1965, vo_c1_Locus_31_Transcript_944/1155_Confidence_0.006_Length_1733, vo_n1_Locus_68_Transcript_21/39_Confidence_0.192_Length_6030, vo_n2_Locus_18661_Transcript_4/4_Confidence_0.000_Length_7078, vo_n3_Locus_81_Transcript_46/54_Confidence_0.340_Length_2532, tri_c_comp40807_c0_seq1, tri_n_comp50453_c0_seq1, vo_c3_Locus_7422_Transcript_1/2_Confidence_1.000_Length_5077, vo_n2_Locus_3_Transcript_6587/24291_Confidence_0.001_Length_4002, vo_c3_Locus_5_Transcript_148/353_Confidence_0.052_Length_4150, vo_n2_Locus_3_Transcript_2135/24291_Confidence_0.000_Length_5848, tri_c_comp9981_c0_seq1, vo_c2_Locus_37_Transcript_71/305_Confidence_0.030_Length_7470, vo_c3_Locus_5_Transcript_151/353_Confidence_0.013_Length_2255, vo_n2_Locus_1836_Transcript_3/26_Confidence_0.579_Length_3826, vo_n2_Locus_12166_Transcript_1/1_Confidence_0.000_Length_2506, vo_n2_Locus_5350_Transcript_3/6_Confidence_0.375_Length_4163, vo_c3_Locus_32274_Transcript_3/4_Confidence_0.333_Length_1761, vo_c3_Locus_32274_Transcript_4/4_Confidence_0.111_Length_782, tri_c_comp8863_c0_seq2, tri_c_comp31434_c0_seq1, vo_n1_Locus_11021_Transcript_1/1_Confidence_0.000_Length_1895, vo_c1_Locus_16918_Transcript_2/2_Confidence_0.000_Length_1637, vo_n2_Locus_15575_Transcript_2/4_Confidence_0.714_Length_1610, tri_c_comp4030_c0_seq1, vo_c2_Locus_35825_Transcript_1/2_Confidence_0.500_Length_995, vo_c2_Locus_35825_Transcript_2/2_Confidence_0.000_Length_972, tri_n_comp34093_c0_seq1, vo_c2_Locus_11939_Transcript_1/4_Confidence_0.273_Length_1336, vo_c1_Locus_18906_Transcript_3/4_Confidence_0.400_Length_1298, vo_c2_Locus_26280_Transcript_1/1_Confidence_0.000_Length_1316, vo_c1_Locus_31652_Transcript_2/3_Confidence_0.429_Length_964, vo_c3_Locus_2584_Transcript_3/5_Confidence_0.727_Length_2680, vo_c3_Locus_2584_Transcript_5/5_Confidence_0.500_Length_3685, vo_c1_Locus_2715_Transcript_9/14_Confidence_0.429_Length_1359, vo_c1_Locus_2066_Transcript_3/17_Confidence_0.415_Length_3989, vo_c3_Locus_4226_Transcript_1/2_Confidence_1.000_Length_2714, vo_c1_Locus_10489_Transcript_1/1_Confidence_0.000_Length_1447, vo_c2_Locus_80_Transcript_8/10_Confidence_0.310_Length_2635, vo_c1_Locus_2066_Transcript_16/17_Confidence_0.293_Length_2774, vo_n1_Locus_4390_Transcript_2/5_Confidence_0.333_Length_2677, vo_n1_Locus_4390_Transcript_3/5_Confidence_0.600_Length_2659, vo_n2_Locus_2860_Transcript_3/6_Confidence_0.250_Length_3111, vo_n1_Locus_2992_Transcript_5/14_Confidence_0.476_Length_1500, vo_n2_Locus_294_Transcript_20/20_Confidence_0.167_Length_2119, vo_c3_Locus_3000_Transcript_12/15_Confidence_0.407_Length_1288, vo_c2_Locus_5247_Transcript_3/7_Confidence_0.500_Length_1443, vo_n1_Locus_357_Transcript_3/6_Confidence_0.429_Length_2710, vo_c2_Locus_5247_Transcript_6/7_Confidence_0.600_Length_1453, vo_c2_Locus_80_Transcript_10/10_Confidence_0.207_Length_2759, vo_c3_Locus_3000_Transcript_6/15_Confidence_0.556_Length_1355, vo_n1_Locus_1856_Transcript_4/7_Confidence_0.462_Length_1396, vo_c2_Locus_5247_Transcript_5/7_Confidence_0.300_Length_1303, vo_c2_Locus_5247_Transcript_4/7_Confidence_0.400_Length_1501, vo_c1_Locus_2715_Transcript_6/14_Confidence_0.429_Length_1383, vo_c3_Locus_3737_Transcript_1/1_Confidence_0.500_Length_2762, vo_c2_Locus_5247_Transcript_7/7_Confidence_0.100_Length_1511, tri_c_comp13375_c0_seq4, vo_c1_Locus_1353_Transcript_2/5_Confidence_0.600_Length_1352, vo_c3_Locus_3000_Transcript_7/15_Confidence_0.259_Length_1522, vo_c2_Locus_6282_Transcript_1/2_Confidence_0.857_Length_2758, vo_n2_Locus_2860_Transcript_4/6_Confidence_0.583_Length_4084, tri_c_comp13375_c0_seq3, vo_n1_Locus_2992_Transcript_6/14_Confidence_0.476_Length_1522, vo_c2_Locus_1093_Transcript_1/3_Confidence_0.714_Length_1748, vo_c2_Locus_1093_Transcript_3/3_Confidence_0.286_Length_1727, tri_n_comp22909_c0_seq1, tri_c_comp21907_c0_seq1, vo_c2_Locus_5036_Transcript_1/2_Confidence_1.000_Length_1776, vo_c2_Locus_35825_Transcript_1/2_Confidence_0.500_Length_995, vo_c2_Locus_35825_Transcript_2/2_Confidence_0.000_Length_972, tri_n_comp14890_c0_seq3, vo_c1_Locus_103_Transcript_20/42_Confidence_0.312_Length_4488, vo_n2_Locus_12749_Transcript_4/14_Confidence_0.387_Length_2426, vo_c3_Locus_9603_Transcr

Amino sugar and nucleotide sugar metabolism	map00520	29	ec:1.1.1.187 - reductase, ec:2.6.1.16 - transaminase (isomerizing), ec:2.7.1.44 - galacturonokinase (phosphorylating) D-galacturonic acid kinase, ec:2.7.1.46 - L-arabinokinase (phosphorylating), ec:2.7.1.43 - glucuronokinase (phosphorylating), ec:4.2.1.76 - 4,6-dehydratase, ec:5.3.1.8 - isomerase, ec:5.3.1.9 - isomerase, ec:3.2.1.52 - hexosaminidase, ec:2.7.7.44 - uridylyltransferase, ec:3.2.1.55 - end alpha-L-arabinofuranosidase, ec:2.7.7.10 - uridylyltransferase, ec:2.7.7.12 - uridylyltransferase, ec:2.3.1.4 - N-acetyltransferase, ec:3.2.1.14 - ChiC, ec:2.7.1.2 - glucokinase (phosphorylating), ec:2.7.1.6 - galactokinase (phosphorylating), ec:5.1.3.2 - 4-epimerase, ec:5.1.3.5 - 4-epimerase, ec:5.1.3.6 - 4-epimerase, ec:2.7.7.9 - uridylyltransferase, ec:1.1.1.271 - synthase, ec:5.4.99.9 - mutase, ec:3.5.99.6 - deaminase, ec:4.2.1.47 - 4,6-dehydratase, ec:1.6.2.2 - reductase, ec:5.4.2.2 - (alpha-D-glucose-1,6-bisphosphate-dependent), ec:5.4.2.3 - mutase, ec:1.1.1.22 - 6-dehydrogenase	1, 1, 2, 3, 2, 2, 2, 2, 13, 5, 1, 5, 1, 1, 1, 2, 4, 9, 3, 1, 10, 1, 3, 1, 3, 1, 7, 1, 1	vo_c2_Locus_4832_Transcript_1/1_Confidence_0.000_Length_1285, vo_n2_Locus_1408_Transcript_4/5_Confidence_0.222_Length_11768, tri_c_comp10536_c0_seq1, vo_c2_Locus_32726_Transcript_2/2_Confidence_0.000_Length_696, tri_c_comp10536_c0_seq1, tri_c_comp28455_c0_seq1, vo_c2_Locus_32726_Transcript_2/2_Confidence_0.000_Length_696, tri_c_comp10536_c0_seq1, vo_c2_Locus_32726_Transcript_2/2_Confidence_0.000_Length_696, vo_c2_Locus_5377_Transcript_5/9_Confidence_0.150_Length_2142, vo_n1_Locus_5366_Transcript_4/4_Confidence_0.556_Length_1623, vo_c2_Locus_1828_Transcript_1/2_Confidence_0.963_Length_1886, vo_n2_Locus_19533_Transcript_1/1_Confidence_0.000_Length_1783, vo_n2_Locus_11241_Transcript_1/3_Confidence_0.600_Length_3773, vo_c2_Locus_479_Transcript_1/2_Confidence_1.000_Length_2154, vo_c3_Locus_25279_Transcript_1/3_Confidence_0.500_Length_3460, tri_c_comp15706_c0_seq2, vo_n2_Locus_4554_Transcript_3/4_Confidence_0.444_Length_2253, vo_n2_Locus_3_Transcript_18735/24291_Confidence_0.000_Length_2121, vo_n2_Locus_4395_Transcript_3/3_Confidence_0.167_Length_2032, vo_c2_Locus_6406_Transcript_1/4_Confidence_0.333_Length_1666, vo_n1_Locus_14758_Transcript_1/2_Confidence_0.667_Length_1933, tri_n_comp35667_c0_seq1, vo_n2_Locus_4395_Transcript_1/3_Confidence_0.667_Length_1925, tri_c_comp32936_c0_seq1, tri_n_comp92955_c0_seq1, tri_n_comp30287_c0_seq1, vo_n2_Locus_4554_Transcript_4/4_Confidence_0.111_Length_2049, vo_n2_Locus_12563_Transcript_1/1_Confidence_0.000_Length_1886, vo_c1_Locus_21075_Transcript_1/3_Confidence_0.500_Length_1034, vo_n1_Locus_3509_Transcript_2/2_Confidence_0.000_Length_1894, tri_c_comp13085_c0_seq3, tri_c_comp54583_c0_seq1, vo_n1_Locus_20887_Transcript_1/1_Confidence_0.000_Length_886, vo_n2_Locus_12563_Transcript_1/1_Confidence_0.000_Length_1886, vo_c1_Locus_21075_Transcript_1/3_Confidence_0.500_Length_1034, vo_n1_Locus_3509_Transcript_2/2_Confidence_0.000_Length_1894, tri_c_comp13085_c0_seq3, tri_c_comp54583_c0_seq1, vo_n2_Locus_26011_Transcript_1/2_Confidence_0.333_Length_2712, vo_n2_Locus_23544_Transcript_2/2_Confidence_0.333_Length_704, vo_n1_Locus_19422_Transcript_1/1_Confidence_0.000_Length_1575, tri_c_comp10536_c0_seq1, vo_c2_Locus_32726_Transcript_2/2_Confidence_0.000_Length_696, vo_n1_Locus_7597_Transcript_1/2_Confidence_0.333_Length_2494, tri_c_comp10536_c0_seq1, vo_c1_Locus_21386_Transcript_3/8_Confidence_0.538_Length_942, vo_c2_Locus_32726_Transcript_2/2_Confidence_0.000_Length_696, vo_c2_Locus_13936_Transcript_11/19_Confidence_0.261_Length_294, tri_n_comp13578_c0_seq1, vo_n1_Locus_7847_Transcript_10/14_Confidence_0.579_Length_1000, vo_n2_Locus_711_Transcript_4/13_Confidence_0.474_Length_608, vo_c3_Locus_20156_Transcript_3/3_Confidence_0.000_Length_1297, vo_n2_Locus_711_Transcript_10/13_Confidence_0.316_Length_1285, vo_n2_Locus_711_Transcript_7/13_Confidence_0.474_Length_690, vo_c2_Locus_13936_Transcript_3/19_Confidence_0.435_Length_599, vo_n1_Locus_7847_Transcript_3/14_Confidence_0.105_Length_511, tri_n_comp13578_c0_seq1, vo_c3_Locus_20156_Transcript_3/3_Confidence_0.000_Length_1297, vo_n2_Locus_711_Transcript_10/13_Confidence_0.316_Length_1285, vo_n1_Locus_4249_Transcript_3/6_Confidence_0.438_Length_1199, vo_n1_Locus_14248_Transcript_2/11_Confidence_0.333_Length_688, vo_c1_Locus_21075_Transcript_1/3_Confidence_0.500_Length_1034, vo_c2_Locus_2206_Transcript_1/2_Confidence_1.000_Length_1671, tri_c_comp13085_c0_seq3, tri_c_comp54583_c0_seq1, vo_c2_Locus_2427_Transcript_3/6_Confidence_0.455_Length_1530, vo_n2_Locus_12563_Transcript_1/1_Confidence_0.000_Length_1886, vo_n2_Locus_6619_Transcript_2/3_Confidence_0.571_Length_1613, vo_n1_Locus_3509_Transcript_2/2_Confidence_0.000_Length_1894, vo_n1_Locus_14248_Transcript_5/11_Confidence_0.222_Length_1040, vo_c2_Locus_4832_Transcript_1/1_Confidence_0.000_Length_1285, vo_c2_Locus_3850_Transcript_1/4_Confidence_0.500_Length_2735, vo_c2_Locus_3850_Transcript_4/4_Confidence_0.250_Length_3514, vo_n2_Locus_1476_Transcript_2/3_Confidence_0.750_Length_1867, vo_c3_Locus_13309_Transcript_1/1_Confidence_0.000_Length_2744, tri_c_comp20306_c0_seq1, vo_c1_Locus_3665_Transcript_26/28_Confidence_0.462_Length_1222, vo_c2_Locus_1898_Transcript_1/15_Confidence_0.462_Length_1289, vo_n2_Locus_24042_Transcript_14/18_Confidence_0.340_Length_1703, tri_n_comp18813_c0_seq13, vo_c2_Locus_23037_Transcript_2/2_Confidence_0.000_Length_2109, vo_n3_Locus_362_Transcript_3/4_Confidence_0.364_Length_3895, vo_n3_Locus_362_Transcript_1/4_Confidence_0.727_Length_3306, vo_n1_Locus_5208_Transcript_2/2_Confidence_0.000_Length_2077, vo_c2_Locus_23037_Transcript_1/2_Confidence_0.750_Length_1965, vo_n3_Locus_15336_Transcript_1/1_Confidence_0.000_Length_1810, vo_n3_Locus_15336_Transcript_1/1_Confidence_0.000_Length_1810, vo_c1_Locus_11739_Transcript_1/3_Confidence_0.929_Length_1730

Cysteine and methionine metabolism	map00270	28	ec:2.7.2.4 - kinase, ec:2.6.1.42 - transaminase, ec:2.6.1.44 - transaminase, ec:2.5.1.16 - synthase, ec:2.5.1.22 - synthase, ec:1.2.1.11 - dehydrogenase, ec:2.5.1.6 - adenosyltransferase, ec:1.1.1.3 - dehydrogenase, ec:2.1.1.10 - S-methyltransferase, ec:2.1.1.13 - synthase, ec:3.3.1.1 - S-adenosylhomocysteine synthase, ec:2.6.1.1 - transaminase, ec:4.3.1.17 - ammonia-lyase, ec:4.4.1.8 - beta-lyase, ec:4.4.1.1 - gamma-lyase, ec:4.4.1.15 - desulfhydrase, ec:2.8.1.2 - sulfurtransferase, ec:6.3.2.3 - synthase, ec:6.3.2.2 - ligase, ec:1.13.11.54 - dioxygenase [iron(II)-requiring], ec:2.3.1.31 - O-acetyltransferase, ec:2.3.1.30 - O-acetyltransferase, ec:1.13.11.20 - dioxygenase, ec:2.3.1.46 - O-succinyltransferase, ec:3.5.99.7 - deaminase, ec:4.2.1.22 - beta-synthase, ec:2.5.1.47 - synthase, ec:1.1.1.37 - dehydrogenase	7, 6, 2, 1, 2, 4, 18, 1, 4, 4, 39, 4, 2, 3, 7, 2, 1, 1, 5, 1, 4, 4, 2, 1, 2, 8, 5, 24	vo_c1_Locus_1636_Transcript_120/122_Confidence_0.135_Length_3625, tri_n_comp29741_c0_seq1, vo_c3_Locus_31_Transcript_74/79_Confidence_0.124_Length_2769, vo_c2_Locus_10862_Transcript_1/1_Confidence_0.000_Length_1564, vo_c3_Locus_31_Transcript_63/79_Confidence_0.148_Length_3613, vo_c1_Locus_1636_Transcript_116/122_Confidence_0.129_Length_3526, vo_c2_Locus_52_Transcript_164/466_Confidence_0.011_Length_2810, vo_c2_Locus_384_Transcript_2/3_Confidence_0.400_Length_1725, tri_c_comp32679_c0_seq1, vo_c1_Locus_10788_Transcript_1/1_Confidence_0.000_Length_1721, vo_c2_Locus_14969_Transcript_4/8_Confidence_0.750_Length_1231, vo_n2_Locus_3786_Transcript_2/4_Confidence_0.778_Length_1466, vo_n2_Locus_3786_Transcript_3/4_Confidence_0.667_Length_1442, vo_c3_Locus_33872_Transcript_1/1_Confidence_0.000_Length_1446, vo_c1_Locus_7220_Transcript_2/3_Confidence_0.400_Length_2898, vo_c2_Locus_25147_Transcript_1/2_Confidence_0.333_Length_1097, vo_c2_Locus_25147_Transcript_1/2_Confidence_0.333_Length_1097, tri_c_comp23701_c0_seq1, vo_n2_Locus_3068_Transcript_2/3_Confidence_0.250_Length_1991, vo_n1_Locus_4025_Transcript_3/5_Confidence_0.286_Length_3475, vo_c1_Locus_9004_Transcript_2/4_Confidence_0.400_Length_3537, vo_c1_Locus_10440_Transcript_1/2_Confidence_0.667_Length_1569, vo_c1_Locus_209_Transcript_22/41_Confidence_0.253_Length_2098, vo_n1_Locus_818_Transcript_23/28_Confidence_0.300_Length_1700, vo_n1_Locus_818_Transcript_22/28_Confidence_0.320_Length_1697, vo_c1_Locus_209_Transcript_25/41_Confidence_0.253_Length_2035, vo_n3_Locus_2574_Transcript_13/21_Confidence_0.419_Length_1731, vo_c1_Locus_209_Transcript_21/41_Confidence_0.207_Length_2111, vo_c1_Locus_209_Transcript_24/41_Confidence_0.241_Length_2150, vo_n3_Locus_2574_Transcript_6/21_Confidence_0.387_Length_1729, vo_n3_Locus_2574_Transcript_4/21_Confidence_0.323_Length_1795, vo_c2_Locus_1176_Transcript_15/36_Confidence_0.126_Length_2169, vo_n2_Locus_206_Transcript_73/103_Confidence_0.088_Length_2363, vo_c3_Locus_479_Transcript_94/98_Confidence_0.030_Length_2004, vo_c1_Locus_209_Transcript_23/41_Confidence_0.207_Length_2096, vo_c1_Locus_209_Transcript_38/41_Confidence_0.069_Length_1149, vo_c2_Locus_1176_Transcript_10/36_Confidence_0.150_Length_2209, vo_c3_Locus_479_Transcript_93/98_Confidence_0.072_Length_1825, vo_c2_Locus_1176_Transcript_2/36_Confidence_0.150_Length_1923, vo_c2_Locus_1176_Transcript_22/36_Confidence_0.087_Length_1396, vo_c2_Locus_10862_Transcript_1/1_Confidence_0.000_Length_1564, vo_n3_Locus_12898_Transcript_5/14_Confidence_0.429_Length_3637, vo_n2_Locus_4083_Transcript_7/15_Confidence_0.571_Length_4447, vo_c3_Locus_876_Transcript_5/9_Confidence_0.526_Length_3245, vo_n2_Locus_4083_Transcript_2/15_Confidence_0.619_Length_4830, vo_n3_Locus_12898_Transcript_5/14_Confidence_0.429_Length_3637, vo_n2_Locus_4083_Transcript_7/15_Confidence_0.571_Length_4447, vo_c3_Locus_876_Transcript_5/9_Confidence_0.526_Length_3245, vo_n2_Locus_4083_Transcript_2/15_Confidence_0.619_Length_4830, vo_c2_Locus_307_Transcript_26/35_Confidence_0.431_Length_2318, vo_c1_Locus_245_Transcript_19/37_Confidence_0.467_Length_1908, vo_n1_Locus_332_Transcript_22/32_Confidence_0.140_Length_1176, vo_n2_Locus_3_Transcript_3356/24291_Confidence_0.000_Length_1953, vo_c2_Locus_307_Transcript_11/35_Confidence_0.403_Length_2123, vo_c3_Locus_829_Transcript_29/29_Confidence_0.233_Length_1857, vo_c2_Locus_307_Transcript_20/35_Confidence_0.333_Length_1795, vo_n2_Locus_3_Transcript_3359/24291_Confidence_0.000_Length_2133, vo_n3_Locus_66_Transcript_20/33_Confidence_0.187_Length_1893, vo_n2_Locus_3_Transcript_3344/24291_Confidence_0.000_Length_2398, vo_c2_Locus_307_Transcript_13/35_Confidence_0.389_Length_2048, vo_c2_Locus_307_Transcript_19/35_Confidence_0.375_Length_1796, vo_c2_Locus_41824_Transcript_1/1_Confidence_0.750_Length_325, vo_n3_Locus_66_Transcript_32/33_Confidence_0.106_Length_2329, vo_c1_Locus_245_Transcript_8/37_Confidence_0.083_Length_2442, vo_c1_Locus_245_Transcript_9/37_Confidence_0.450_Length_2027, vo_c3_Locus_829_Transcript_5/29_Confidence_0.558_Length_1940, vo_n1_Locus_332_Transcript_14/32_Confidence_0.340_Length_2438, vo_n3_Locus_66_Transcript_13/33_Confidence_0.203_Length_1680, vo_n3_Locus_66_Transcript_21/33_Confidence_0.211_Length_2011, vo_c2_Locus_307_Transcript_17/35_Confidence_0.097_Length_2301, vo_n3_Locus_66_Transcript_23/33_Confidence_0.252_Length_2080, vo_n2_Locus_3_Transcript_3352/24291_Confidence_0.000_Length_1867, vo_c2_Locus_307_Transcript_7/35_Confidence_0.181_Length_3468, vo_c3_Locus_829_Transcript_8/29_Confidence_0.488_Length_1870, vo_n2_Locus_3_Transcript_3340/24291_Confidence_0.000_Length_2561, vo_n3_Locus_66_Transcript_18/33_Confidence_0.650_Length_2193, vo_c3_Locus_829_Transcript_3/29_Confidence_0.442_Length_1977, vo_n1_Locus_332_Transcript_21/32_Confidence_0.520_Length_3273, vo_n3_Locus_66_Transcript_28/33_Confidence_0.195_Length_1816, tri_n_comp20010_c0_seq1, vo_c1_Locus_245_Transcript_11/37_Confidence_0.350_Length_1730, vo_n2_Locus_3_Transcript_3339/24291_Confidence_0.000_Length_1719, vo_n1_Locus_332_Transcript_16/32_Confidence_0.330_Length_2427, tri_c_comp22471_c0_seq1, vo_n3_Locus_66_Transcript_22/33_Confidence_0.211_Length_1962, vo_n2_Locus_3_Transcript_3353/24291_Confidence_0.000_Length_1105, vo_n3_Locus_66_Transcript_26/33_Confidence_0.122_Length_3344, vo_n2_Locus_3_Transcript_3338/24291_Confidence_0.000_Length_1541, vo_n1_Locus_3423_Transcript_2/4_Confidence_0.250_Length_1347, tri_n_comp27683_c0_seq1, vo_c1_Locus_3127_Transcript_1/1_Confidence_0.000_Length_1478, vo_n1_Locus_3423_Transcript_3/4_Confidence_0.250_Length_1416, vo_c2_Locus_30746_Transcript_1/1_Confidence_0.000_Length_1279, vo_n1_Locus_2779_Transcript_1/2_Confidence_0.333_Length_2962, vo_n2_Locus_7374_Transcript_2/3_Confidence_0.750_Length_1746, vo_c2_Locus_31468_Transcript_4/5_Confidence_0.667_Length_1579, vo_n1_Locus_3498_Transcript_1/3_Confidence_0.600_Length_1462, tri_c_comp42317_c0_seq1, vo_n2_Locus_7374_Transcript_2/3_Confidence_0.750_Length_1746, vo_n2_Locus_8711_Transcript_5/5_Confidence_0.067_Length_1558, vo_c2_Locus_11984_Transcript_2/6_Confidence_0.385_Length_1557, vo_c2_Locus_31468_Transcript_4/5_Confidence_0.667_Length_1579, vo_n1_Locus_3498_Transcript_1/3_Confidence_0.600_Length_1462, tri_c_comp30049_c0_seq1, vo_c3_Locus_1071_Transcript_2/6_Confidence_0.571_Length_1547, vo_c1_Locus_1555_Transcript_3/3_Confidence_0.200_Length_1281, vo_n1_Locus_21114_Transcript_3/5_Confidence_0.444_Length_1255, vo_c1_Locus_8043_Transcript_1/1_Confidence_0.000_Length_1747, vo_n2_Locus_20981_Transcript_1/1_Confidence_0.000_Length_2435, vo_n2_Locus_5430_Transcript_2/3_Confidence_0.400_Length_1394, tri_n_comp34788_c0_seq1, vo_c1_Locus_1493_Transcript_3/4_Confidence_0.286_Length_2721,

					vo_n3_Locus_29456_Transcript_1/1_Confidence_0.000_Length_1949, tri_n_comp41228_c0_seq1, vo_n1_Locus_9946_Transcript_1/3_Confidence_0.714_Length_1615, vo_c1_Locus_2886_Transcript_6/19_Confidence_0.481_Length_4535, vo_c2_Locus_4812_Transcript_2/3_Confidence_0.625_Length_1578, vo_c2_Locus_4812_Transcript_1/3_Confidence_0.625_Length_1831, vo_n1_Locus_9946_Transcript_1/3_Confidence_0.714_Length_1615, vo_c1_Locus_2886_Transcript_6/19_Confidence_0.481_Length_4535, vo_c2_Locus_4812_Transcript_2/3_Confidence_0.625_Length_1578, vo_c2_Locus_4812_Transcript_1/3_Confidence_0.625_Length_1831, vo_c2_Locus_20699_Transcript_22/34_Confidence_0.180_Length_2604, vo_c2_Locus_20699_Transcript_12/34_Confidence_0.165_Length_2453, vo_n1_Locus_9681_Transcript_11/11_Confidence_0.000_Length_529, vo_c3_Locus_1071_Transcript_2/6_Confidence_0.571_Length_1547, vo_c1_Locus_1555_Transcript_3/3_Confidence_0.200_Length_1281, vo_n1_Locus_664_Transcript_3/4_Confidence_0.429_Length_1448, tri_n_comp16677_c0_seq4, vo_c1_Locus_1695_Transcript_1/2_Confidence_0.974_Length_1764, tri_n_comp16677_c0_seq1, tri_c_comp17806_c0_seq2, vo_c3_Locus_4013_Transcript_1/4_Confidence_0.625_Length_2457, vo_n2_Locus_211_Transcript_4/8_Confidence_0.400_Length_1517, vo_c2_Locus_615_Transcript_1/2_Confidence_0.400_Length_2717, vo_c2_Locus_2964_Transcript_1/2_Confidence_0.333_Length_1319, tri_c_comp42317_c0_seq1, vo_n2_Locus_211_Transcript_4/8_Confidence_0.400_Length_1517, vo_c1_Locus_1695_Transcript_1/2_Confidence_0.974_Length_1764, tri_n_comp16677_c0_seq1, vo_c3_Locus_1723_Transcript_29/37_Confidence_0.411_Length_1695, vo_n2_Locus_330_Transcript_25/38_Confidence_0.361_Length_1516, vo_c3_Locus_1723_Transcript_35/37_Confidence_0.464_Length_1702, vo_c1_Locus_15206_Transcript_3/3_Confidence_0.000_Length_1312, vo_c1_Locus_7371_Transcript_4/4_Confidence_0.600_Length_1352, vo_c3_Locus_1723_Transcript_37/37_Confidence_0.214_Length_1530, vo_n1_Locus_769_Transcript_28/41_Confidence_0.400_Length_1609, vo_n1_Locus_769_Transcript_9/41_Confidence_0.460_Length_1494, vo_c3_Locus_2965_Transcript_2/2_Confidence_0.250_Length_1327, vo_n2_Locus_330_Transcript_5/38_Confidence_0.328_Length_11715, vo_n1_Locus_9772_Transcript_1/2_Confidence_0.333_Length_1224, vo_c1_Locus_3603_Transcript_44/48_Confidence_0.492_Length_1696, vo_c2_Locus_4242_Transcript_2/3_Confidence_0.400_Length_1168, vo_n1_Locus_6649_Transcript_2/3_Confidence_0.500_Length_2124, vo_c1_Locus_7371_Transcript_1/4_Confidence_0.500_Length_1352, vo_n3_Locus_2381_Transcript_2/3_Confidence_0.500_Length_1330, vo_c3_Locus_1723_Transcript_16/37_Confidence_0.286_Length_1491, vo_n1_Locus_769_Transcript_36/41_Confidence_0.420_Length_1536, vo_n1_Locus_769_Transcript_40/41_Confidence_0.320_Length_1479, vo_n1_Locus_6649_Transcript_1/3_Confidence_0.750_Length_1883, vo_c1_Locus_3603_Transcript_26/48_Confidence_0.400_Length_1432, vo_n1_Locus_5348_Transcript_2/2_Confidence_0.200_Length_1608, vo_n3_Locus_3383_Transcript_35/37_Confidence_0.389_Length_1512, tri_c_comp20802_c0_seq1

Starch and sucrose metabolism	map00500	25	ec:2.7.1.106 - synthase, ec:3.2.1.4 - endo-1,4-beta-D-glucanase, ec:3.2.1.3 - 1,4-alpha-glucosidase, ec:3.2.1.2 - saccharogen amylase, ec:3.2.1.1 - glycogenase, ec:3.2.1.68 - debranching enzyme, ec:5.3.1.9 - isomerase, ec:3.2.1.58 - 1,3-beta-glucosidase, ec:3.2.1.91 - 1,4-beta-cellobiosidase (non-reducing end), ec:3.2.1.20 - maltase, ec:3.2.1.21 - gentiobiase, ec:2.4.1.34 - synthase, ec:3.2.1.28 - trehalase, ec:3.2.1.39 - endo-1,3-beta-D-glucosidase, ec:2.4.1.1 - phosphorylase, ec:2.4.1.18 - branching enzyme, ec:2.4.1.15 - synthase (UDP-forming), ec:2.4.1.21 - synthase (glycosyl-transferring), ec:3.6.1.9 - diphosphatase, ec:2.7.1.2 - glucokinase (phosphorylating), ec:3.1.3.12 - trehalose 6-phosphatase, ec:3.6.1.21 - diphosphatase, ec:2.7.7.9 - uridylyltransferase, ec:5.4.2.2 - (alpha-D-glucose-1,6-bisphosphate-dependent), ec:5.4.2.6 - beta-pgm (gene name)	1, 11, 1, 4, 7, 2, 2, 4, 47, 1, 1, 2, 2, 3, 3, 7, 2, 12, 7, 2, 2, 5, 10, 7, 2	vo_c2_Locus_23037_Transcript_1/2_Confidence_0.750_Length_1965, vo_n1_Locus_3997_Transcript_24/86_Confidence_0.037_Length_866, tri_c_comp21238_c0_seq2, vo_n2_Locus_3_Transcript_20185/24291_Confidence_0.000_Length_2632, vo_n2_Locus_3_Transcript_20192/24291_Confidence_0.000_Length_2788, vo_n1_Locus_3997_Transcript_14/86_Confidence_0.162_Length_2014, vo_n1_Locus_3997_Transcript_82/86_Confidence_0.143_Length_9458, vo_c2_Locus_1527_Transcript_36/36_Confidence_0.072_Length_913, vo_c2_Locus_1527_Transcript_18/36_Confidence_0.234_Length_2293, vo_n1_Locus_3997_Transcript_29/86_Confidence_0.092_Length_2830, vo_c1_Locus_276_Transcript_20/22_Confidence_0.068_Length_2129, vo_n1_Locus_3997_Transcript_22/86_Confidence_0.007_Length_1222, vo_c2_Locus_14395_Transcript_1/1_Confidence_0.000_Length_3374, vo_n1_Locus_14414_Transcript_1/2_Confidence_0.833_Length_1154, vo_n2_Locus_37776_Transcript_1/1_Confidence_0.000_Length_675, vo_n3_Locus_23299_Transcript_1/2_Confidence_0.667_Length_2577, tri_c_comp21076_c0_seq5, vo_c1_Locus_4524_Transcript_1/3_Confidence_0.600_Length_3012, vo_n1_Locus_6549_Transcript_2/6_Confidence_0.643_Length_4768, vo_n1_Locus_6549_Transcript_6/6_Confidence_0.214_Length_4881, vo_n1_Locus_3507_Transcript_2/3_Confidence_0.400_Length_3947, vo_c1_Locus_16465_Transcript_1/1_Confidence_0.000_Length_2200, tri_n_comp5289_c0_seq1, vo_n1_Locus_986_Transcript_27/36_Confidence_0.155_Length_9490, tri_c_comp28455_c0_seq1, tri_n_comp30681_c0_seq1, vo_n2_Locus_11241_Transcript_1/3_Confidence_0.600_Length_3773, vo_c2_Locus_479_Transcript_1/2_Confidence_1.000_Length_2154, tri_c_comp12867_c0_seq1, tri_n_comp70995_c0_seq1, vo_c3_Locus_4852_Transcript_3/3_Confidence_0.000_Length_806, vo_c1_Locus_788_Transcript_9/34_Confidence_0.021_Length_747, vo_c3_Locus_147_Transcript_27/36_Confidence_0.385_Length_3809, vo_n1_Locus_11315_Transcript_44/46_Confidence_0.376_Length_3978, vo_n2_Locus_174_Transcript_26/35_Confidence_0.385_Length_3142, vo_c3_Locus_147_Transcript_15/36_Confidence_0.429_Length_3820, vo_n2_Locus_174_Transcript_9/35_Confidence_0.319_Length_3595, vo_c3_Locus_147_Transcript_24/36_Confidence_0.407_Length_4120, vo_n3_Locus_223_Transcript_35/39_Confidence_0.222_Length_2437, tri_c_comp21238_c0_seq2, vo_n2_Locus_3_Transcript_20185/24291_Confidence_0.000_Length_2632, vo_c3_Locus_10908_Transcript_16/18_Confidence_0.333_Length_2326, vo_n1_Locus_1202_Transcript_20/44_Confidence_0.398_Length_3502, vo_c3_Locus_147_Transcript_13/36_Confidence_0.429_Length_3782, vo_c3_Locus_147_Transcript_23/36_Confidence_0.363_Length_3964, vo_c1_Locus_1050_Transcript_45/49_Confidence_0.390_Length_3244, vo_c3_Locus_147_Transcript_11/36_Confidence_0.440_Length_3299, vo_n3_Locus_223_Transcript_18/39_Confidence_0.489_Length_2944, vo_n1_Locus_3997_Transcript_24/86_Confidence_0.037_Length_866, vo_n3_Locus_14056_Transcript_23/30_Confidence_0.484_Length_3994, vo_c2_Locus_3916_Transcript_32/38_Confidence_0.391_Length_3924, vo_n1_Locus_11315_Transcript_40/46_Confidence_0.387_Length_3974, vo_n1_Locus_3997_Transcript_14/86_Confidence_0.162_Length_2014, vo_n1_Locus_3997_Transcript_82/86_Confidence_0.143_Length_9458, vo_c2_Locus_3916_Transcript_35/38_Confidence_0.379_Length_3853, vo_n3_Locus_223_Transcript_11/39_Confidence_0.333_Length_2328, vo_n1_Locus_1202_Transcript_40/44_Confidence_0.482_Length_3789, vo_c2_Locus_3916_Transcript_25/38_Confidence_0.460_Length_4104, vo_n1_Locus_1202_Transcript_4/44_Confidence_0.301_Length_2245, vo_n1_Locus_3997_Transcript_29/86_Confidence_0.092_Length_2830, vo_n1_Locus_3997_Transcript_22/86_Confidence_0.007_Length_1222, vo_n2_Locus_174_Transcript_3/35_Confidence_0.099_Length_2501, vo_n1_Locus_1202_Transcript_41/44_Confidence_0.482_Length_3746, vo_n2_Locus_3_Transcript_20192/24291_Confidence_0.000_Length_2788, vo_c1_Locus_1050_Transcript_34/49_Confidence_0.390_Length_3322, vo_c1_Locus_1050_Transcript_4/49_Confidence_0.317_Length_2768, vo_n1_Locus_1202_Transcript_34/44_Confidence_0.386_Length_3186, vo_n2_Locus_174_Transcript_25/35_Confidence_0.363_Length_3321, vo_c2_Locus_1527_Transcript_36/36_Confidence_0.072_Length_913, vo_c2_Locus_3916_Transcript_23/38_Confidence_0.460_Length_4069, vo_n1_Locus_1202_Transcript_21/44_Confidence_0.301_Length_2911, vo_c1_Locus_1050_Transcript_29/49_Confidence_0.329_Length_3265, vo_c1_Locus_21512_Transcript_13/13_Confidence_0.170_Length_812, vo_c2_Locus_1527_Transcript_18/36_Confidence_0.234_Length_2293, vo_c3_Locus_147_Transcript_5/36_Confidence_0.275_Length_2545, vo_n1_Locus_11315_Transcript_31/46_Confidence_0.398_Length_3986, vo_n3_Locus_223_Transcript_29/39_Confidence_0.256_Length_2902, vo_c1_Locus_276_Transcript_20/22_Confidence_0.068_Length_2129, vo_c2_Locus_3916_Transcript_38/38_Confidence_0.184_Length_4071, vo_n2_Locus_3872_Transcript_1/1_Confidence_0.000_Length_2389, vo_c2_Locus_13105_Transcript_1/1_Confidence_0.000_Length_2797, vo_n2_Locus_11990_Transcript_1/1_Confidence_0.000_Length_7576, vo_n2_Locus_1515_Transcript_20/24_Confidence_0.103_Length_4140, vo_c1_Locus_7595_Transcript_2/2_Confidence_0.000_Length_2004, vo_n1_Locus_29249_Transcript_2/2_Confidence_0.250_Length_2011, tri_c_comp12867_c0_seq1, tri_n_comp70995_c0_seq1, vo_c3_Locus_32079_Transcript_2/2_Confidence_0.250_Length_2113, vo_n1_Locus_20275_Transcript_1/1_Confidence_0.000_Length_2621, vo_n2_Locus_1397_Transcript_7/15_Confidence_0.091_Length_5840, vo_n2_Locus_618_Transcript_2/13_Confidence_0.167_Length_2515, vo_c1_Locus_5279_Transcript_4/8_Confidence_0.600_Length_6117, vo_c3_Locus_6753_Transcript_5/9_Confidence_0.467_Length_6108, vo_n3_Locus_734_Transcript_1/5_Confidence_0.375_Length_3606, tri_c_comp42932_c0_seq1, vo_c3_Locus_6753_Transcript_8/9_Confidence_0.200_Length_3716, vo_c1_Locus_7002_Transcript_5/7_Confidence_0.462_Length_4257, vo_c3_Locus_7490_Transcript_1/4_Confidence_0.667_Length_2130, vo_c1_Locus_18692_Transcript_18/20_Confidence_0.455_Length_4000, vo_n2_Locus_17428_Transcript_1/1_Confidence_0.000_Length_3307, vo_c2_Locus_262_Transcript_3/36_Confidence_0.143_Length_2398, vo_c2_Locus_262_Transcript_5/36_Confidence_0.365_Length_2514, vo_n3_Locus_734_Transcript_1/5_Confidence_0.375_Length_3606, vo_c2_Locus_12549_Transcript_1/1_Confidence_0.000_Length_2428, vo_c2_Locus_6067_Transcript_7/10_Confidence_0.235_Length_1527, vo_c3_Locus_6753_Transcript_8/9_Confidence_0.200_Length_3716, vo_c2_Locus_6067_Transcript_9/10_Confidence_0.176_Length_1493, vo_n2_Locus_3675_Transcript_3/4_Confidence_0.167_Length_2615, tri_c_comp21252_c0_seq24, vo_c2_Locus_5836_Transcript_6/6_Confidence_0.400_Length_2597, vo_n3_Locus_600_Transcript_20/32_Confidence_0.426_Length_2952, vo_c2_Locus_262_Transcript_4/36_Confidence_0.302_Length_2651, vo_c2_Locus_35825_Transcript_1/2_Confidence_0.500_Length_995, vo_c2_Locus_35825_Transcript_2/2_Confidence_0.000_Length_972, tri_n_comp34093_c0_seq1, vo_c2_Locus_11939_Transcript_1/4_Confidence_0.273_Length_1336,

					vo_c1_Locus_18906_Transcript_3/4_Confidence_0.400_Length_1298, vo_c2_Locus_26280_Transcript_1/1_Confidence_0.000_Length_1316, vo_c1_Locus_31652_Transcript_2/3_Confidence_0.429_Length_964, tri_c_comp10536_c0_seq1, vo_c2_Locus_32726_Transcript_2/2_Confidence_0.000_Length_696, vo_c1_Locus_18692_Transcript_18/20_Confidence_0.455_Length_4000, vo_n2_Locus_17428_Transcript_1/1_Confidence_0.000_Length_3307, vo_c2_Locus_35825_Transcript_1/2_Confidence_0.500_Length_995, vo_c2_Locus_35825_Transcript_2/2_Confidence_0.000_Length_972, vo_c1_Locus_18906_Transcript_3/4_Confidence_0.400_Length_1298, tri_n_comp34093_c0_seq1, vo_c2_Locus_11939_Transcript_1/4_Confidence_0.273_Length_1336, vo_n1_Locus_14248_Transcript_2/11_Confidence_0.333_Length_688, vo_c1_Locus_21075_Transcript_1/3_Confidence_0.500_Length_1034, vo_c2_Locus_2206_Transcript_1/2_Confidence_1.000_Length_1671, tri_c_comp13085_c0_seq3, tri_c_comp54583_c0_seq1, vo_c2_Locus_2427_Transcript_3/6_Confidence_0.455_Length_1530, vo_n2_Locus_12563_Transcript_1/1_Confidence_0.000_Length_1886, vo_n2_Locus_6619_Transcript_2/3_Confidence_0.571_Length_1613, vo_n1_Locus_3509_Transcript_2/2_Confidence_0.000_Length_1894, vo_n1_Locus_14248_Transcript_5/11_Confidence_0.222_Length_1040, tri_n_comp18813_c0_seq13, vo_c2_Locus_23037_Transcript_2/2_Confidence_0.000_Length_2109, vo_n3_Locus_362_Transcript_3/4_Confidence_0.364_Length_3895, vo_n3_Locus_362_Transcript_1/4_Confidence_0.727_Length_3306, vo_n1_Locus_5208_Transcript_2/2_Confidence_0.000_Length_2077, vo_c2_Locus_23037_Transcript_1/2_Confidence_0.750_Length_1965, vo_n3_Locus_15336_Transcript_1/1_Confidence_0.000_Length_1810, vo_n2_Locus_19354_Transcript_2/2_Confidence_0.000_Length_489, tri_c_comp14768_c0_seq1

Glycine, serine and threonine metabolism	map00260	24	ec:1.4.3.3 - oxidase, ec:2.6.1.52 - transaminase, ec:2.7.2.4 - kinase, ec:2.6.1.44 - transaminase, ec:1.1.1.103 - 3-dehydrogenase, ec:1.2.1.11 - dehydrogenase, ec:1.5.8.4 - dehydrogenase, ec:1.5.8.3 - dehydrogenase, ec:1.8.1.4 - dehydrogenase, ec:3.1.3.3 - phosphatase, ec:1.1.1.3 - dehydrogenase, ec:4.3.1.17 - ammonia-lyase, ec:4.3.1.18 - ammonia-lyase, ec:4.3.1.19 - ammonia-lyase, ec:1.2.1.8 - dehydrogenase, ec:4.4.1.1 - gamma-lyase, ec:1.1.99.1 - dehydrogenase, ec:2.1.2.1 - hydroxymethyltransferase, ec:1.4.4.2 - dehydrogenase (aminomethyl-transferring), ec:2.3.1.29 - C-acetyltransferase, ec:2.1.2.10 - S-aminomethyldihydrolipoylprotein:(6S)-tetrahydrofolate aminomethyltransferase (ammonia-forming), ec:4.2.1.22 - beta-synthase, ec:4.2.1.20 - synthase, ec:1.4.3.21 - oxidase	1, 4, 7, 2, 10, 4, 1, 1, 2, 2, 1, 2, 2, 2, 1, 7, 2, 9, 3, 1, 9, 8, 2, 1	vo_n2_Locus_26350_Transcript_7/9_Confidence_0.231_Length_1309, vo_n2_Locus_2440_Transcript_3/6_Confidence_0.182_Length_1751, tri_c_comp24934_c0_seq1, vo_c3_Locus_9067_Transcript_6/7_Confidence_0.125_Length_1757, vo_c2_Locus_1886_Transcript_3/5_Confidence_0.444_Length_2764, vo_c1_Locus_1636_Transcript_120/122_Confidence_0.135_Length_3625, tri_n_comp29741_c0_seq1, vo_c3_Locus_31_Transcript_74/79_Confidence_0.124_Length_2769, vo_c2_Locus_10862_Transcript_1/1_Confidence_0.000_Length_1564, vo_c3_Locus_31_Transcript_63/79_Confidence_0.148_Length_3613, vo_c1_Locus_1636_Transcript_116/122_Confidence_0.129_Length_3526, vo_c2_Locus_52_Transcript_164/466_Confidence_0.011_Length_2810, vo_c3_Locus_33872_Transcript_1/1_Confidence_0.000_Length_1446, vo_c1_Locus_7220_Transcript_2/3_Confidence_0.400_Length_2898, vo_c2_Locus_7588_Transcript_12/16_Confidence_0.364_Length_3463, vo_n1_Locus_2728_Transcript_5/7_Confidence_0.143_Length_3965, vo_c3_Locus_217_Transcript_3/16_Confidence_0.360_Length_1567, vo_c2_Locus_7588_Transcript_10/16_Confidence_0.455_Length_4063, vo_c2_Locus_7588_Transcript_11/16_Confidence_0.318_Length_4206, vo_c2_Locus_7588_Transcript_6/16_Confidence_0.455_Length_4082, vo_c3_Locus_217_Transcript_4/16_Confidence_0.160_Length_468, vo_c2_Locus_7588_Transcript_14/16_Confidence_0.227_Length_3444, vo_c2_Locus_7588_Transcript_7/16_Confidence_0.455_Length_4225, vo_n3_Locus_18141_Transcript_3/4_Confidence_0.400_Length_4159, vo_n2_Locus_3068_Transcript_2/3_Confidence_0.250_Length_1991, vo_n1_Locus_4025_Transcript_3/5_Confidence_0.286_Length_3475, vo_c1_Locus_9004_Transcript_2/4_Confidence_0.400_Length_3537, vo_c1_Locus_10440_Transcript_1/2_Confidence_0.667_Length_1569, vo_c3_Locus_7605_Transcript_3/4_Confidence_0.250_Length_2875, tri_n_comp13806_c0_seq3, vo_n3_Locus_6810_Transcript_2/3_Confidence_0.913_Length_1699, vo_n1_Locus_6460_Transcript_1/1_Confidence_0.000_Length_1912, vo_c3_Locus_3840_Transcript_2/5_Confidence_0.200_Length_1699, vo_c2_Locus_27691_Transcript_1/2_Confidence_0.750_Length_923, vo_c2_Locus_10862_Transcript_1/1_Confidence_0.000_Length_1564, vo_c2_Locus_30746_Transcript_1/1_Confidence_0.000_Length_1279, vo_n1_Locus_2779_Transcript_1/2_Confidence_0.333_Length_2962, vo_c2_Locus_30746_Transcript_1/1_Confidence_0.000_Length_1279, vo_n1_Locus_2779_Transcript_1/2_Confidence_0.333_Length_2962, vo_c2_Locus_30746_Transcript_1/1_Confidence_0.000_Length_1279, tri_c_comp34117_c0_seq1, vo_c3_Locus_7280_Transcript_1/2_Confidence_0.667_Length_1614, tri_c_comp42317_c0_seq1, vo_n2_Locus_7374_Transcript_2/3_Confidence_0.750_Length_1746, vo_n2_Locus_8711_Transcript_5/5_Confidence_0.067_Length_1558, vo_c2_Locus_11984_Transcript_2/6_Confidence_0.385_Length_1557, vo_c2_Locus_31468_Transcript_4/5_Confidence_0.667_Length_1579, vo_n1_Locus_3498_Transcript_1/3_Confidence_0.600_Length_1462, tri_c_comp30049_c0_seq1, tri_n_comp26575_c0_seq1, tri_n_comp24797_c0_seq1, vo_c2_Locus_1465_Transcript_1/1_Confidence_0.000_Length_2684, vo_n2_Locus_668_Transcript_12/17_Confidence_0.465_Length_3343, vo_n3_Locus_1281_Transcript_13/14_Confidence_0.467_Length_2089, vo_n2_Locus_668_Transcript_10/17_Confidence_0.419_Length_1865, vo_c3_Locus_6604_Transcript_15/15_Confidence_0.590_Length_1792, vo_n3_Locus_1281_Transcript_3/14_Confidence_0.533_Length_1904, vo_c3_Locus_6604_Transcript_12/15_Confidence_0.230_Length_1729, tri_n_comp22443_c0_seq1, vo_c2_Locus_2191_Transcript_14/17_Confidence_0.464_Length_1841, vo_c3_Locus_6331_Transcript_3/4_Confidence_0.200_Length_905, tri_n_comp24631_c0_seq1, vo_c2_Locus_52_Transcript_237/466_Confidence_0.002_Length_590, tri_n_comp5199_c0_seq1, tri_c_comp25586_c0_seq1, vo_c2_Locus_23277_Transcript_1/1_Confidence_0.000_Length_1058, vo_n1_Locus_803_Transcript_2/31_Confidence_0.118_Length_2529, vo_n1_Locus_27713_Transcript_1/1_Confidence_0.000_Length_1034, tri_n_comp13806_c0_seq3, tri_c_comp13451_c0_seq2, vo_n1_Locus_803_Transcript_9/31_Confidence_0.324_Length_2360, vo_n2_Locus_3952_Transcript_1/2_Confidence_0.750_Length_1244, vo_n2_Locus_514_Transcript_37/44_Confidence_0.155_Length_1950, vo_n1_Locus_664_Transcript_3/4_Confidence_0.429_Length_1448, tri_n_comp16677_c0_seq4, vo_c1_Locus_1695_Transcript_1/2_Confidence_0.974_Length_1764, tri_n_comp16677_c0_seq1, tri_c_comp17806_c0_seq2, vo_c3_Locus_4013_Transcript_1/4_Confidence_0.625_Length_2457, vo_n2_Locus_211_Transcript_4/8_Confidence_0.400_Length_1517, vo_c2_Locus_615_Transcript_1/2_Confidence_0.400_Length_2717, tri_c_comp44055_c0_seq1, vo_c1_Locus_8760_Transcript_1/1_Confidence_0.000_Length_2415, tri_n_comp48625_c0_seq1

Pyrimidine metabolism	map00240	22	ec:2.7.4.6 - kinase, ec:3.5.2.3 - carbamoylaspartic dehydrase, ec:2.7.4.9 - kinase, ec:2.7.1.48 - kinase, ec:2.1.3.2 - carbamoyltransferase, ec:4.2.1.70 - synthase, ec:2.1.1.45 - synthase, ec:3.1.3.5 - uridine 5'-nucleotidase, ec:6.3.5.5 - synthase (glutamine-hydrolysing), ec:6.3.4.2 - synthase (glutamine hydrolysing), ec:3.6.1.6 - diphosphate phosphatase, ec:3.6.1.8 - diphosphatase, ec:3.6.1.9 - diphosphatase, ec:1.17.4.1 - reductase, ec:3.6.1.23 - diphosphatase, ec:2.7.7.6 - RNA polymerase, ec:2.7.7.7 - DNA polymerase, ec:2.4.2.10 - phosphoribosyltransferase, ec:3.5.4.12 - deaminase, ec:2.7.4.22 - kinase, ec:2.4.2.9 - phosphoribosyltransferase, ec:2.7.4.14 - kinase	20, 4, 4, 10, 3, 1, 5, 11, 2, 1, 7, 1, 7, 32, 1, 24, 30, 1, 1, 8, 2, 16	vo_c1_Locus_13409_Transcript_2/6_Confidence_0.286_Length_871, vo_c3_Locus_2169_Transcript_1/3_Confidence_0.167_Length_848, vo_n2_Locus_739_Transcript_8/8_Confidence_0.167_Length_1338, vo_n3_Locus_4095_Transcript_2/3_Confidence_0.444_Length_3401, vo_c1_Locus_12423_Transcript_2/2_Confidence_0.333_Length_980, vo_c1_Locus_25242_Transcript_1/1_Confidence_0.000_Length_1465, vo_c1_Locus_4920_Transcript_1/2_Confidence_0.714_Length_1520, tri_c_comp49412_c0_seq1, vo_n2_Locus_5279_Transcript_1/1_Confidence_0.333_Length_2493, vo_n2_Locus_20723_Transcript_1/1_Confidence_0.000_Length_1179, vo_n3_Locus_24634_Transcript_1/1_Confidence_0.000_Length_445, vo_n1_Locus_5513_Transcript_4/4_Confidence_0.286_Length_1053, tri_c_comp24673_c0_seq1, tri_c_comp5012_c0_seq2, vo_c3_Locus_3080_Transcript_1/28_Confidence_0.009_Length_1584, vo_n1_Locus_9563_Transcript_1/1_Confidence_0.000_Length_1437, vo_n2_Locus_26604_Transcript_1/2_Confidence_0.750_Length_1361, vo_n2_Locus_2689_Transcript_2/2_Confidence_0.000_Length_2612, vo_c2_Locus_4541_Transcript_2/6_Confidence_0.571_Length_909, vo_n2_Locus_26604_Transcript_2/2_Confidence_0.000_Length_1685, vo_c2_Locus_11816_Transcript_2/2_Confidence_0.250_Length_1509, vo_n1_Locus_6422_Transcript_1/1_Confidence_0.000_Length_1398, tri_c_comp33901_c0_seq1, vo_n2_Locus_5805_Transcript_1/2_Confidence_0.333_Length_5227, vo_n2_Locus_7391_Transcript_1/1_Confidence_0.000_Length_1253, vo_n1_Locus_18221_Transcript_2/3_Confidence_0.333_Length_2330, tri_n_comp29271_c0_seq1, tri_c_comp39328_c0_seq1, vo_n1_Locus_27053_Transcript_1/1_Confidence_0.333_Length_1866, vo_n2_Locus_1685_Transcript_1/2_Confidence_0.667_Length_854, vo_n1_Locus_1829_Transcript_14/31_Confidence_0.635_Length_1771, vo_n1_Locus_1829_Transcript_24/31_Confidence_0.558_Length_1469, vo_c3_Locus_527_Transcript_60/87_Confidence_0.092_Length_2707, vo_n1_Locus_1829_Transcript_21/31_Confidence_0.519_Length_1458, vo_c1_Locus_12423_Transcript_2/2_Confidence_0.333_Length_980, vo_n2_Locus_26604_Transcript_1/2_Confidence_0.750_Length_1361, vo_n1_Locus_1829_Transcript_31/31_Confidence_0.269_Length_1123, vo_n2_Locus_26604_Transcript_2/2_Confidence_0.000_Length_1685, tri_n_comp27462_c0_seq1, tri_c_comp33901_c0_seq1, vo_n2_Locus_5805_Transcript_1/2_Confidence_0.333_Length_5227, tri_c_comp27551_c0_seq1, tri_c_comp11590_c0_seq1, vo_c3_Locus_15578_Transcript_2/4_Confidence_0.722_Length_967, vo_c1_Locus_6843_Transcript_1/2_Confidence_0.833_Length_813, vo_c2_Locus_1969_Transcript_1/2_Confidence_0.200_Length_1807, vo_c1_Locus_6843_Transcript_2/2_Confidence_0.167_Length_995, vo_c1_Locus_21072_Transcript_1/1_Confidence_0.000_Length_965, vo_c2_Locus_25862_Transcript_4/4_Confidence_0.000_Length_988, vo_c2_Locus_26482_Transcript_5/6_Confidence_0.286_Length_1654, vo_c2_Locus_26482_Transcript_4/6_Confidence_0.571_Length_1609, vo_n2_Locus_21191_Transcript_4/6_Confidence_0.300_Length_1691, vo_c1_Locus_18480_Transcript_2/2_Confidence_0.000_Length_1013, vo_c2_Locus_27118_Transcript_12/49_Confidence_0.025_Length_3122, vo_c2_Locus_6520_Transcript_5/15_Confidence_0.455_Length_1419, vo_c3_Locus_7168_Transcript_5/8_Confidence_0.538_Length_1343, vo_n1_Locus_7057_Transcript_3/15_Confidence_0.611_Length_1294, vo_n2_Locus_21191_Transcript_3/6_Confidence_0.500_Length_1646, tri_c_comp33901_c0_seq1, vo_n2_Locus_5805_Transcript_1/2_Confidence_0.333_Length_5227, vo_c3_Locus_13321_Transcript_1/2_Confidence_0.500_Length_4253, vo_c3_Locus_32274_Transcript_3/4_Confidence_0.333_Length_1761, vo_c3_Locus_32274_Transcript_4/4_Confidence_0.111_Length_782, tri_c_comp8863_c0_seq2, tri_c_comp31434_c0_seq1, vo_n1_Locus_11021_Transcript_1/1_Confidence_0.000_Length_1895, vo_c1_Locus_16918_Transcript_2/2_Confidence_0.000_Length_1637, vo_n2_Locus_15575_Transcript_2/4_Confidence_0.714_Length_1610, tri_c_comp4030_c0_seq1, vo_c2_Locus_35825_Transcript_1/2_Confidence_0.500_Length_995, vo_c2_Locus_35825_Transcript_2/2_Confidence_0.000_Length_972, tri_n_comp34093_c0_seq1, vo_c2_Locus_11939_Transcript_1/4_Confidence_0.273_Length_1336, vo_c1_Locus_18906_Transcript_3/4_Confidence_0.400_Length_1298, vo_c2_Locus_26280_Transcript_1/1_Confidence_0.000_Length_1316, vo_c1_Locus_31652_Transcript_2/3_Confidence_0.429_Length_964, vo_c3_Locus_2584_Transcript_3/5_Confidence_0.727_Length_2680, vo_c3_Locus_2584_Transcript_5/5_Confidence_0.500_Length_3685, vo_c1_Locus_2715_Transcript_9/14_Confidence_0.429_Length_1359, vo_c1_Locus_2066_Transcript_3/17_Confidence_0.415_Length_3989, vo_c3_Locus_4226_Transcript_1/2_Confidence_1.000_Length_2714, vo_c1_Locus_10489_Transcript_1/1_Confidence_0.000_Length_1447, vo_c2_Locus_80_Transcript_8/10_Confidence_0.310_Length_2635, vo_c1_Locus_2066_Transcript_16/17_Confidence_0.293_Length_2774, vo_n1_Locus_4390_Transcript_2/5_Confidence_0.333_Length_2677, vo_n1_Locus_4390_Transcript_3/5_Confidence_0.600_Length_2659, vo_n2_Locus_2860_Transcript_3/6_Confidence_0.250_Length_3111, vo_n1_Locus_2992_Transcript_5/14_Confidence_0.476_Length_1500, vo_n2_Locus_294_Transcript_20/20_Confidence_0.167_Length_2119, vo_c3_Locus_3000_Transcript_12/15_Confidence_0.407_Length_1288, vo_c2_Locus_5247_Transcript_3/7_Confidence_0.500_Length_1443, vo_n1_Locus_357_Transcript_3/6_Confidence_0.429_Length_2710, vo_c2_Locus_5247_Transcript_6/7_Confidence_0.600_Length_1453, vo_c2_Locus_80_Transcript_10/10_Confidence_0.207_Length_2759, vo_c3_Locus_3000_Transcript_6/15_Confidence_0.556_Length_1355, vo_n1_Locus_1856_Transcript_4/7_Confidence_0.462_Length_1396, vo_c2_Locus_5247_Transcript_5/7_Confidence_0.300_Length_1303, vo_c2_Locus_5247_Transcript_4/7_Confidence_0.400_Length_1501, vo_c1_Locus_2715_Transcript_6/14_Confidence_0.429_Length_1383, vo_c3_Locus_3737_Transcript_1/1_Confidence_0.500_Length_2762, vo_c2_Locus_5247_Transcript_7/7_Confidence_0.100_Length_1511, tri_c_comp13375_c0_seq4, vo_c1_Locus_1353_Transcript_2/5_Confidence_0.600_Length_1352, vo_c3_Locus_3000_Transcript_7/15_Confidence_0.259_Length_1522, vo_c2_Locus_6282_Transcript_1/2_Confidence_0.857_Length_2758, vo_n2_Locus_2860_Transcript_4/6_Confidence_0.583_Length_4084, tri_c_comp13375_c0_seq3, vo_n1_Locus_2992_Transcript_6/14_Confidence_0.476_Length_1522, vo_c3_Locus_13948_Transcript_1/1_Confidence_0.000_Length_1677, vo_c2_Locus_21951_Transcript_1/2_Confidence_0.667_Length_522, vo_c3_Locus_8549_Transcript_1/1_Confidence_0.000_Length_686, tri_n_comp6678_c0_seq2, vo_n1_Locus_29096_Transcript_1/1_Confidence_0.000_Length_4601, vo_n1_Locus_26419_Transcript_1/1_Confidence_0.000_Length_1111, vo_n1_Locus_24077_Transcript_1/2_Confidence_0.333_Length_525, vo_c2_Locus_3665_Transcript_1/2_Confidence_1.000_Length_637, tri_c_comp46174_c0_seq1, tri_n_comp45393_c0_seq1, tri_n_comp46803_c0_seq1,

					vo_n2_Locus_1565_Transcript_2/3_Confidence_0.400_Length_2971, vo_c1_Locus_2941_Transcript_1/5_Confidence_0.625_Length_2451, vo_c2_Locus_3489_Transcript_4/4_Confidence_0.286_Length_6498, vo_n2_Locus_12191_Transcript_8/8_Confidence_0.077_Length_5136, vo_c3_Locus_29721_Transcript_4/9_Confidence_0.357_Length_544, tri_n_comp54991_c0_seq1, vo_n2_Locus_212_Transcript_1/2_Confidence_0.667_Length_4253, tri_n_comp45898_c0_seq1, vo_c2_Locus_25849_Transcript_3/3_Confidence_0.000_Length_380, vo_n1_Locus_24077_Transcript_2/2_Confidence_0.000_Length_636, vo_c3_Locus_19591_Transcript_3/4_Confidence_0.429_Length_1562, vo_n2_Locus_14100_Transcript_2/6_Confidence_0.429_Length_978, tri_n_comp34592_c0_seq1, vo_n2_Locus_18092_Transcript_1/2_Confidence_1.000_Length_590, vo_c2_Locus_5970_Transcript_2/3_Confidence_0.400_Length_1948, vo_c1_Locus_12052_Transcript_1/1_Confidence_0.000_Length_2738, vo_c2_Locus_23845_Transcript_1/1_Confidence_0.500_Length_1993, vo_c3_Locus_11479_Transcript_1/2_Confidence_1.000_Length_1915, vo_c2_Locus_10542_Transcript_7/18_Confidence_0.290_Length_2762, vo_n1_Locus_9968_Transcript_1/1_Confidence_0.000_Length_2754, vo_c3_Locus_882_Transcript_5/5_Confidence_0.125_Length_2804, tri_c_comp17035_c0_seq1, tri_n_comp28983_c0_seq1, vo_n2_Locus_1838_Transcript_6/10_Confidence_0.524_Length_5262, tri_c_comp17035_c0_seq4, vo_c1_Locus_4956_Transcript_1/7_Confidence_0.600_Length_2026, vo_c2_Locus_21629_Transcript_1/2_Confidence_0.600_Length_5843, vo_c2_Locus_1147_Transcript_1/2_Confidence_0.750_Length_1828, vo_c3_Locus_4611_Transcript_2/3_Confidence_0.333_Length_1813, vo_c2_Locus_15210_Transcript_1/1_Confidence_0.000_Length_935, vo_n2_Locus_1536_Transcript_2/3_Confidence_0.400_Length_9999, vo_n2_Locus_1838_Transcript_3/10_Confidence_0.190_Length_2641, vo_n2_Locus_19943_Transcript_1/1_Confidence_0.333_Length_5399, vo_n2_Locus_6649_Transcript_25/30_Confidence_0.093_Length_2933, vo_c2_Locus_5289_Transcript_1/4_Confidence_0.556_Length_1812, vo_c2_Locus_10542_Transcript_1/18_Confidence_0.290_Length_2706, vo_c3_Locus_4899_Transcript_2/2_Confidence_0.000_Length_2213, vo_c2_Locus_29692_Transcript_1/1_Confidence_0.000_Length_3737, tri_c_comp9488_c0_seq2, vo_n1_Locus_2112_Transcript_1/3_Confidence_0.600_Length_3970, vo_n2_Locus_17002_Transcript_2/3_Confidence_0.333_Length_3454, vo_c2_Locus_35701_Transcript_2/2_Confidence_0.000_Length_939, vo_c2_Locus_27257_Transcript_1/2_Confidence_0.333_Length_3415, vo_n1_Locus_9358_Transcript_1/2_Confidence_0.333_Length_2793, vo_c1_Locus_6671_Transcript_2/2_Confidence_0.000_Length_1815, tri_n_comp28286_c0_seq1, vo_n1_Locus_5513_Transcript_4/4_Confidence_0.286_Length_1053, vo_c1_Locus_13409_Transcript_2/6_Confidence_0.286_Length_871, vo_c3_Locus_2169_Transcript_1/3_Confidence_0.167_Length_848, vo_n2_Locus_739_Transcript_8/8_Confidence_0.167_Length_1338, vo_c1_Locus_12423_Transcript_2/2_Confidence_0.333_Length_980, vo_n2_Locus_26604_Transcript_1/2_Confidence_0.750_Length_1361, vo_c2_Locus_4541_Transcript_2/6_Confidence_0.571_Length_909, vo_n2_Locus_26604_Transcript_2/2_Confidence_0.000_Length_1685, vo_n1_Locus_27053_Transcript_1/1_Confidence_0.333_Length_1866, vo_n2_Locus_1685_Transcript_1/2_Confidence_0.667_Length_854, vo_n2_Locus_2594_Transcript_4/9_Confidence_0.538_Length_5946, vo_c1_Locus_13409_Transcript_2/6_Confidence_0.286_Length_871, vo_c3_Locus_2169_Transcript_1/3_Confidence_0.167_Length_848, vo_n2_Locus_739_Transcript_8/8_Confidence_0.167_Length_1338, vo_c2_Locus_289_Transcript_2/6_Confidence_0.667_Length_4931, vo_c1_Locus_12423_Transcript_2/2_Confidence_0.333_Length_980, vo_n2_Locus_5279_Transcript_1/1_Confidence_0.333_Length_2493, vo_n3_Locus_24634_Transcript_1/1_Confidence_0.000_Length_445, vo_c2_Locus_289_Transcript_5/6_Confidence_0.667_Length_4996, vo_n1_Locus_5513_Transcript_4/4_Confidence_0.286_Length_1053, tri_c_comp24673_c0_seq1, vo_c3_Locus_167_Transcript_14/17_Confidence_0.041_Length_5034, vo_n2_Locus_26604_Transcript_1/2_Confidence_0.750_Length_1361, vo_n2_Locus_2594_Transcript_6/9_Confidence_0.538_Length_6223, vo_c2_Locus_4541_Transcript_2/6_Confidence_0.571_Length_909, vo_n2_Locus_26604_Transcript_2/2_Confidence_0.000_Length_1685

Pyruvate metabolism	map00620	21	ec:6.4.1.1 - carboxylase, ec:6.4.1.2 - carboxylase, ec:4.1.1.49 - carboxykinase (ATP), ec:4.1.1.32 - carboxykinase (GTP), ec:2.7.1.40 - kinase, ec:6.2.1.1 - ligase, ec:1.8.1.4 - dehydrogenase, ec:1.2.1.3 - dehydrogenase (NAD+), ec:2.3.1.9 - C-acetyltransferase, ec:4.4.1.5 - lyase, ec:1.1.5.4 - dehydrogenase (quinone), ec:2.3.3.13 - synthase, ec:4.2.1.2 - hydratase, ec:2.3.1.54 - C-acetyltransferase, ec:3.1.2.6 - hydrolase, ec:2.7.9.2 - water dikinase, ec:1.1.1.82 - dehydrogenase (NADP+), ec:1.1.1.37 - dehydrogenase, ec:1.1.1.39 - dehydrogenase (decarboxylating), ec:1.1.1.40 - dehydrogenase (oxaloacetate-decarboxylating) (NADP+), ec:1.1.1.28 - dehydrogenase	1, 16, 5, 5, 25, 1, 2, 5, 2, 2, 3, 1, 2, 9, 7, 15, 23, 24, 11, 7, 9	vo_n2_Locus_15097_Transcript_1/1_Confidence_0.000_Length_3935, vo_n3_Locus_484_Transcript_6/12_Confidence_0.250_Length_1533, vo_c3_Locus_17332_Transcript_1/1_Confidence_0.333_Length_6493, vo_n3_Locus_696_Transcript_20/35_Confidence_0.473_Length_5396, vo_c3_Locus_2164_Transcript_38/39_Confidence_0.292_Length_6879, tri_n_comp41552_c0_seq1, vo_c2_Locus_1509_Transcript_1/47_Confidence_0.149_Length_2731, vo_c2_Locus_2193_Transcript_1/3_Confidence_0.667_Length_1218, vo_c1_Locus_860_Transcript_10/29_Confidence_0.571_Length_6744, vo_c2_Locus_1509_Transcript_11/47_Confidence_0.164_Length_2686, vo_n2_Locus_1751_Transcript_51/59_Confidence_0.207_Length_3566, vo_n2_Locus_15097_Transcript_1/1_Confidence_0.000_Length_3935, vo_n3_Locus_484_Transcript_5/12_Confidence_0.250_Length_1451, vo_c1_Locus_860_Transcript_11/29_Confidence_0.571_Length_6741, tri_c_comp19913_c0_seq3, vo_n2_Locus_1751_Transcript_37/59_Confidence_0.195_Length_3437, vo_c2_Locus_1509_Transcript_6/47_Confidence_0.149_Length_2592, vo_c1_Locus_2295_Transcript_9/10_Confidence_0.238_Length_2213, vo_c2_Locus_2862_Transcript_9/10_Confidence_0.250_Length_2209, vo_c1_Locus_2295_Transcript_3/10_Confidence_0.524_Length_2330, vo_n2_Locus_1690_Transcript_8/11_Confidence_0.429_Length_2234, vo_n2_Locus_1690_Transcript_9/11_Confidence_0.429_Length_2277, vo_c1_Locus_2295_Transcript_9/10_Confidence_0.238_Length_2213, vo_c2_Locus_2862_Transcript_9/10_Confidence_0.250_Length_2209, vo_c1_Locus_2295_Transcript_3/10_Confidence_0.524_Length_2330, vo_n2_Locus_1690_Transcript_8/11_Confidence_0.429_Length_2234, vo_n2_Locus_1690_Transcript_9/11_Confidence_0.429_Length_2277, vo_n2_Locus_9037_Transcript_9/15_Confidence_0.486_Length_4553, vo_c2_Locus_684_Transcript_7/16_Confidence_0.194_Length_2803, vo_n3_Locus_2293_Transcript_1/2_Confidence_0.750_Length_1794, vo_n3_Locus_1756_Transcript_10/15_Confidence_0.393_Length_2226, vo_c3_Locus_725_Transcript_6/15_Confidence_0.548_Length_5086, vo_n3_Locus_1756_Transcript_7/15_Confidence_0.250_Length_2226, vo_n2_Locus_16147_Transcript_1/1_Confidence_0.000_Length_1452, vo_n2_Locus_9037_Transcript_2/15_Confidence_0.257_Length_3951, vo_n2_Locus_9037_Transcript_13/15_Confidence_0.143_Length_1729, vo_n2_Locus_9037_Transcript_5/15_Confidence_0.286_Length_2279, vo_n2_Locus_9037_Transcript_6/15_Confidence_0.229_Length_2279, tri_c_comp19644_c0_seq1, vo_n2_Locus_171_Transcript_17/42_Confidence_0.229_Length_1803, tri_c_comp19644_c0_seq4, vo_c3_Locus_12256_Transcript_3/3_Confidence_0.000_Length_508, vo_c3_Locus_725_Transcript_12/15_Confidence_0.290_Length_3194, vo_c1_Locus_6543_Transcript_5/23_Confidence_0.220_Length_2294, vo_n2_Locus_9037_Transcript_12/15_Confidence_0.257_Length_2318, vo_c3_Locus_725_Transcript_13/15_Confidence_0.452_Length_4249, tri_n_comp19211_c0_seq1, vo_c3_Locus_883_Transcript_1/2_Confidence_1.000_Length_1783, vo_n2_Locus_9037_Transcript_1/15_Confidence_0.143_Length_1677, vo_c3_Locus_307_Transcript_2/21_Confidence_0.576_Length_1809, vo_n1_Locus_41_Transcript_20/90_Confidence_0.068_Length_2175, vo_c2_Locus_1667_Transcript_21/21_Confidence_0.195_Length_1788, vo_n3_Locus_9269_Transcript_1/1_Confidence_0.000_Length_2238, vo_n3_Locus_6810_Transcript_2/3_Confidence_0.913_Length_1699, vo_n1_Locus_6460_Transcript_1/1_Confidence_0.000_Length_1912, tri_c_comp55340_c0_seq1, vo_n1_Locus_19864_Transcript_2/2_Confidence_0.000_Length_2707, vo_c3_Locus_21741_Transcript_5/9_Confidence_0.583_Length_1757, vo_n3_Locus_25600_Transcript_5/6_Confidence_0.375_Length_1777, tri_n_comp58061_c0_seq1, vo_c1_Locus_2657_Transcript_1/1_Confidence_0.000_Length_1513, vo_n2_Locus_23058_Transcript_1/1_Confidence_0.000_Length_1342, vo_c1_Locus_17818_Transcript_2/2_Confidence_0.000_Length_800, vo_n2_Locus_4321_Transcript_1/2_Confidence_0.750_Length_948, vo_n1_Locus_833_Transcript_2/6_Confidence_0.333_Length_2200, vo_n2_Locus_3042_Transcript_2/3_Confidence_0.750_Length_2030, vo_c1_Locus_6244_Transcript_1/2_Confidence_0.750_Length_1731, vo_n1_Locus_2267_Transcript_1/1_Confidence_0.000_Length_2010, tri_n_comp8247_c0_seq2, vo_c3_Locus_1133_Transcript_4/9_Confidence_0.455_Length_1666, vo_c3_Locus_1564_Transcript_1/27_Confidence_0.474_Length_3491, vo_n2_Locus_4549_Transcript_18/24_Confidence_0.395_Length_3486, vo_c1_Locus_1748_Transcript_10/39_Confidence_0.197_Length_3498, vo_c1_Locus_1748_Transcript_19/39_Confidence_0.211_Length_3788, vo_c1_Locus_1748_Transcript_30/39_Confidence_0.282_Length_4645, vo_n2_Locus_4549_Transcript_11/24_Confidence_0.474_Length_3796, vo_n1_Locus_103_Transcript_17/28_Confidence_0.483_Length_3469, vo_n3_Locus_2375_Transcript_13/19_Confidence_0.467_Length_3465, vo_c3_Locus_1564_Transcript_22/27_Confidence_0.526_Length_3527, vo_c1_Locus_31307_Transcript_5/10_Confidence_0.467_Length_800, vo_n1_Locus_29308_Transcript_2/2_Confidence_0.000_Length_535, vo_n2_Locus_8305_Transcript_5/8_Confidence_0.143_Length_2284, vo_c1_Locus_31307_Transcript_7/10_Confidence_0.467_Length_896, vo_c3_Locus_32598_Transcript_4/10_Confidence_0.385_Length_1319, vo_c2_Locus_15787_Transcript_2/5_Confidence_0.400_Length_917, vo_c2_Locus_15787_Transcript_4/5_Confidence_0.300_Length_1396, vo_c1_Locus_1662_Transcript_19/44_Confidence_0.278_Length_3148, vo_n2_Locus_1146_Transcript_9/32_Confidence_0.492_Length_2897, vo_n3_Locus_3213_Transcript_1/29_Confidence_0.022_Length_515, vo_n1_Locus_1032_Transcript_1/39_Confidence_0.018_Length_458, tri_n_comp20108_c0_seq2, vo_c1_Locus_1662_Transcript_28/44_Confidence_0.200_Length_3087, vo_c2_Locus_49_Transcript_21/157_Confidence_0.007_Length_465, vo_c2_Locus_49_Transcript_31/157_Confidence_0.027_Length_1554, vo_c2_Locus_49_Transcript_20/157_Confidence_0.037_Length_3535, vo_c1_Locus_1662_Transcript_6/44_Confidence_0.211_Length_3160, vo_c2_Locus_49_Transcript_28/157_Confidence_0.071_Length_2983, vo_n1_Locus_1032_Transcript_5/39_Confidence_0.404_Length_3016, vo_n1_Locus_1032_Transcript_38/39_Confidence_0.228_Length_2924, vo_n1_Locus_1032_Transcript_8/39_Confidence_0.386_Length_2954, vo_c3_Locus_1031_Transcript_12/33_Confidence_0.385_Length_3145, vo_c3_Locus_1723_Transcript_29/37_Confidence_0.411_Length_1695, vo_n2_Locus_330_Transcript_25/38_Confidence_0.361_Length_1516, vo_c3_Locus_1723_Transcript_35/37_Confidence_0.464_Length_1702, vo_c1_Locus_7371_Transcript_4/4_Confidence_0.600_Length_1352, vo_c3_Locus_1723_Transcript_37/37_Confidence_0.214_Length_1530, vo_n1_Locus_769_Transcript_28/41_Confidence_0.400_Length_1609, vo_n1_Locus_769_Transcript_9/41_Confidence_0.460_Length_1494, vo_c3_Locus_2965_Transcript_2/2_Confidence_0.250_Length_1327, vo_n2_Locus_330_Transcript_5/38_Confidence_0.328_Length_11715, vo_n1_Locus_9772_Transcript_1/2_Confidence_0.333_Length_1224, vo_c1_Locus_3603_Transcript_44/48_Confidence_0.492_Length_1696, vo_c2_Locus_4242_Transcript_2/3_Confidence_0.400_Length_1168,

					vo_n1_Locus_6649_Transcript_2/3_Confidence_0.500_Length_2124, vo_c1_Locus_7371_Transcript_1/4_Confidence_0.500_Length_1352, vo_n3_Locus_2381_Transcript_2/3_Confidence_0.500_Length_1330, vo_c3_Locus_1723_Transcript_16/37_Confidence_0.286_Length_1491, vo_n1_Locus_769_Transcript_36/41_Confidence_0.420_Length_1536, vo_n1_Locus_769_Transcript_40/41_Confidence_0.320_Length_1479, vo_n1_Locus_6649_Transcript_1/3_Confidence_0.750_Length_1883, vo_c1_Locus_3603_Transcript_26/48_Confidence_0.400_Length_1432, vo_n1_Locus_5348_Transcript_2/2_Confidence_0.200_Length_1608, vo_n3_Locus_3383_Transcript_35/37_Confidence_0.389_Length_1512, tri_c_comp20802_c0_seq1, vo_c3_Locus_1723_Transcript_29/37_Confidence_0.411_Length_1695, vo_n2_Locus_330_Transcript_25/38_Confidence_0.361_Length_1516, vo_c3_Locus_1723_Transcript_35/37_Confidence_0.464_Length_1702, vo_c1_Locus_15206_Transcript_3/3_Confidence_0.000_Length_1312, vo_c1_Locus_7371_Transcript_4/4_Confidence_0.600_Length_1352, vo_c3_Locus_1723_Transcript_37/37_Confidence_0.214_Length_1530, vo_n1_Locus_769_Transcript_28/41_Confidence_0.400_Length_1609, vo_n1_Locus_769_Transcript_9/41_Confidence_0.460_Length_1494, vo_c3_Locus_2965_Transcript_2/2_Confidence_0.250_Length_1327, vo_n2_Locus_330_Transcript_5/38_Confidence_0.328_Length_11715, vo_n1_Locus_9772_Transcript_1/2_Confidence_0.333_Length_1224, vo_c1_Locus_3603_Transcript_44/48_Confidence_0.492_Length_1696, vo_c2_Locus_4242_Transcript_2/3_Confidence_0.400_Length_1168, vo_n1_Locus_6649_Transcript_2/3_Confidence_0.500_Length_2124, vo_c1_Locus_7371_Transcript_1/4_Confidence_0.500_Length_1352, vo_n3_Locus_2381_Transcript_2/3_Confidence_0.500_Length_1330, vo_c3_Locus_1723_Transcript_16/37_Confidence_0.286_Length_1491, vo_n1_Locus_769_Transcript_36/41_Confidence_0.420_Length_1536, vo_n1_Locus_769_Transcript_40/41_Confidence_0.320_Length_1479, vo_n1_Locus_6649_Transcript_1/3_Confidence_0.750_Length_1883, vo_c1_Locus_3603_Transcript_26/48_Confidence_0.400_Length_1432, vo_n1_Locus_5348_Transcript_2/2_Confidence_0.200_Length_1608, vo_n3_Locus_3383_Transcript_35/37_Confidence_0.389_Length_1512, tri_c_comp20802_c0_seq1, vo_c1_Locus_8302_Transcript_51/55_Confidence_0.100_Length_500, vo_n2_Locus_2969_Transcript_26/36_Confidence_0.373_Length_1726, vo_n3_Locus_8916_Transcript_29/29_Confidence_0.191_Length_2143, vo_c1_Locus_8302_Transcript_52/55_Confidence_0.100_Length_554, vo_c1_Locus_8302_Transcript_26/55_Confidence_0.129_Length_1144, vo_n3_Locus_8916_Transcript_11/29_Confidence_0.277_Length_861, vo_n2_Locus_2969_Transcript_30/36_Confidence_0.441_Length_2103, vo_c1_Locus_8302_Transcript_55/55_Confidence_0.043_Length_1090, vo_c3_Locus_20712_Transcript_20/28_Confidence_0.550_Length_2132, tri_c_comp20385_c0_seq1, vo_c1_Locus_8302_Transcript_54/55_Confidence_0.114_Length_1036, vo_n3_Locus_8916_Transcript_29/29_Confidence_0.191_Length_2143, vo_c1_Locus_8302_Transcript_26/55_Confidence_0.129_Length_1144, vo_n3_Locus_8916_Transcript_11/29_Confidence_0.277_Length_861, vo_n2_Locus_2969_Transcript_30/36_Confidence_0.441_Length_2103, vo_c3_Locus_20712_Transcript_20/28_Confidence_0.550_Length_2132, tri_c_comp20385_c0_seq1, tri_c_comp20385_c0_seq4, vo_n3_Locus_19185_Transcript_2/2_Confidence_0.000_Length_1260, vo_c2_Locus_2981_Transcript_2/13_Confidence_0.389_Length_2102, vo_c3_Locus_11593_Transcript_2/4_Confidence_0.286_Length_1967, tri_c_comp25619_c0_seq1, vo_c3_Locus_3313_Transcript_3/12_Confidence_0.182_Length_3522, vo_n2_Locus_8997_Transcript_3/3_Confidence_0.167_Length_2401, vo_n2_Locus_9550_Transcript_1/2_Confidence_0.333_Length_1798, vo_c2_Locus_6699_Transcript_1/2_Confidence_0.667_Length_1590, vo_n2_Locus_20141_Transcript_2/18_Confidence_0.143_Length_2044

Glycolysis / Gluconeogenesis	map00010	21	ec:2.7.1.90 - 1-phosphotransferase, ec:4.1.1.49 - carboxykinase (ATP), ec:4.1.1.32 - carboxykinase (GTP), ec:2.7.2.3 - kinase, ec:2.7.1.40 - kinase, ec:1.2.1.59 - dehydrogenase (NAD(P)+) (phosphorylating), ec:2.7.1.11 - phosphohexokinase, ec:6.2.1.1 - ligase, ec:1.2.1.12 - dehydrogenase (phosphorylating), ec:1.8.1.4 - dehydrogenase, ec:5.3.1.1 - isomerase, ec:5.3.1.9 - isomerase, ec:1.1.1.2 - dehydrogenase (NADP+), ec:1.2.1.5 - dehydrogenase [NAD(P)+], ec:1.2.1.3 - dehydrogenase (NAD+), ec:1.2.1.9 - dehydrogenase (NADP+), ec:4.1.2.13 - aldolase, ec:2.7.1.2 - glucokinase (phosphorylating), ec:3.1.3.11 - hexose diphosphatase, ec:4.2.1.11 - hydratase, ec:5.4.2.2 - (alpha-D-glucose-1,6-bisphosphate-dependent)	1, 5, 5, 41, 25, 16, 5, 1, 45, 2, 9, 2, 10, 2, 5, 9, 38, 2, 8, 23, 7	vo_c2_Locus_6348_Transcript_1/2_Confidence_1.000_Length_3755, vo_c1_Locus_2295_Transcript_9/10_Confidence_0.238_Length_2213, vo_c2_Locus_2862_Transcript_9/10_Confidence_0.250_Length_2209, vo_c1_Locus_2295_Transcript_3/10_Confidence_0.524_Length_2330, vo_n2_Locus_1690_Transcript_8/11_Confidence_0.429_Length_2234, vo_n2_Locus_1690_Transcript_9/11_Confidence_0.429_Length_2277, vo_c1_Locus_2295_Transcript_9/10_Confidence_0.238_Length_2213, vo_c2_Locus_2862_Transcript_9/10_Confidence_0.250_Length_2209, vo_c1_Locus_2295_Transcript_3/10_Confidence_0.524_Length_2330, vo_n2_Locus_1690_Transcript_8/11_Confidence_0.429_Length_2234, vo_n2_Locus_1690_Transcript_9/11_Confidence_0.429_Length_2277, vo_n3_Locus_2623_Transcript_14/17_Confidence_0.556_Length_1555, vo_n3_Locus_74_Transcript_17/25_Confidence_0.436_Length_1764, vo_c1_Locus_788_Transcript_17/34_Confidence_0.404_Length_1861, vo_c1_Locus_788_Transcript_30/34_Confidence_0.255_Length_1712, tri_c_comp19022_c0_seq2, vo_c3_Locus_239_Transcript_24/29_Confidence_0.409_Length_3108, vo_c2_Locus_298_Transcript_10/24_Confidence_0.500_Length_1796, tri_n_comp18813_c0_seq15, vo_n2_Locus_106_Transcript_17/30_Confidence_0.344_Length_2068, tri_n_comp18813_c0_seq11, vo_n1_Locus_2248_Transcript_15/40_Confidence_0.275_Length_1844, tri_n_comp18813_c0_seq10, tri_n_comp20156_c0_seq1, vo_c2_Locus_3634_Transcript_2/8_Confidence_0.467_Length_2376, vo_c1_Locus_788_Transcript_5/34_Confidence_0.340_Length_1423, vo_n2_Locus_2252_Transcript_14/26_Confidence_0.410_Length_2227, vo_c3_Locus_239_Transcript_16/29_Confidence_0.364_Length_1433, vo_c3_Locus_2347_Transcript_16/16_Confidence_0.270_Length_1737, vo_c2_Locus_298_Transcript_21/24_Confidence_0.368_Length_1678, vo_c3_Locus_3927_Transcript_2/9_Confidence_0.250_Length_2629, vo_c2_Locus_3634_Transcript_8/8_Confidence_0.133_Length_2428, vo_c2_Locus_2437_Transcript_14/25_Confidence_0.455_Length_1511, vo_c1_Locus_3033_Transcript_3/5_Confidence_0.400_Length_2348, vo_c3_Locus_239_Transcript_29/29_Confidence_0.182_Length_2208, vo_n3_Locus_2623_Transcript_15/17_Confidence_0.361_Length_1551, vo_c3_Locus_2347_Transcript_5/16_Confidence_0.514_Length_1554, vo_c3_Locus_239_Transcript_19/29_Confidence_0.295_Length_1502, vo_c2_Locus_2437_Transcript_25/25_Confidence_0.159_Length_1517, vo_n2_Locus_2252_Transcript_22/26_Confidence_0.256_Length_2337, vo_n2_Locus_2252_Transcript_23/26_Confidence_0.359_Length_1959, vo_c3_Locus_239_Transcript_10/29_Confidence_0.386_Length_1727, vo_c1_Locus_788_Transcript_6/34_Confidence_0.362_Length_1766, vo_c1_Locus_788_Transcript_22/34_Confidence_0.277_Length_1358, vo_n1_Locus_2248_Transcript_26/40_Confidence_0.333_Length_1991, tri_c_comp20571_c0_seq1, vo_n2_Locus_2252_Transcript_18/26_Confidence_0.256_Length_1506, vo_c1_Locus_788_Transcript_11/34_Confidence_0.213_Length_2514, vo_c2_Locus_907_Transcript_3/3_Confidence_0.333_Length_630, vo_n2_Locus_7087_Transcript_1/2_Confidence_0.500_Length_4087, vo_n1_Locus_2248_Transcript_2/40_Confidence_0.373_Length_1784, vo_n2_Locus_2980_Transcript_1/1_Confidence_0.333_Length_1623, vo_n2_Locus_9037_Transcript_9/15_Confidence_0.486_Length_4553, vo_c2_Locus_684_Transcript_7/16_Confidence_0.194_Length_2803, vo_n3_Locus_2293_Transcript_1/2_Confidence_0.750_Length_1794, vo_n3_Locus_1756_Transcript_10/15_Confidence_0.393_Length_2226, vo_c3_Locus_725_Transcript_6/15_Confidence_0.548_Length_5086, vo_n3_Locus_1756_Transcript_7/15_Confidence_0.250_Length_2226, vo_n2_Locus_16147_Transcript_1/1_Confidence_0.000_Length_1452, vo_n2_Locus_9037_Transcript_2/15_Confidence_0.257_Length_3951, vo_n2_Locus_9037_Transcript_13/15_Confidence_0.143_Length_1729, vo_n2_Locus_9037_Transcript_5/15_Confidence_0.286_Length_2279, vo_n2_Locus_9037_Transcript_6/15_Confidence_0.229_Length_2279, tri_c_comp19644_c0_seq1, vo_n2_Locus_171_Transcript_17/42_Confidence_0.229_Length_1803, tri_c_comp19644_c0_seq4, vo_c3_Locus_12256_Transcript_3/3_Confidence_0.000_Length_508, vo_c3_Locus_725_Transcript_12/15_Confidence_0.290_Length_3194, vo_c1_Locus_6543_Transcript_5/23_Confidence_0.220_Length_2294, vo_n2_Locus_9037_Transcript_12/15_Confidence_0.257_Length_2318, vo_c3_Locus_725_Transcript_13/15_Confidence_0.452_Length_4249, tri_n_comp19211_c0_seq1, vo_c3_Locus_883_Transcript_1/2_Confidence_1.000_Length_1783, vo_n2_Locus_9037_Transcript_1/15_Confidence_0.143_Length_1677, vo_c3_Locus_307_Transcript_2/21_Confidence_0.576_Length_1809, vo_n1_Locus_41_Transcript_20/90_Confidence_0.068_Length_2175, vo_c2_Locus_1667_Transcript_21/21_Confidence_0.195_Length_1788, vo_c3_Locus_550_Transcript_25/30_Confidence_0.321_Length_1640, vo_c3_Locus_550_Transcript_10/30_Confidence_0.420_Length_1515, vo_n1_Locus_133_Transcript_12/30_Confidence_0.033_Length_580, vo_c2_Locus_320_Transcript_7/32_Confidence_0.299_Length_1642, vo_c3_Locus_550_Transcript_27/30_Confidence_0.395_Length_1758, vo_c2_Locus_320_Transcript_9/32_Confidence_0.338_Length_1673, vo_c2_Locus_320_Transcript_22/32_Confidence_0.545_Length_1648, vo_c3_Locus_550_Transcript_30/30_Confidence_0.210_Length_1413, vo_c3_Locus_550_Transcript_8/30_Confidence_0.148_Length_1492, vo_n3_Locus_587_Transcript_13/22_Confidence_0.444_Length_1456, vo_n2_Locus_105_Transcript_30/33_Confidence_0.449_Length_1990, vo_c3_Locus_550_Transcript_28/30_Confidence_0.309_Length_1831, vo_c2_Locus_320_Transcript_20/32_Confidence_0.325_Length_1586, vo_n1_Locus_133_Transcript_30/30_Confidence_0.033_Length_2622, vo_c2_Locus_320_Transcript_13/32_Confidence_0.351_Length_2604, vo_c2_Locus_320_Transcript_15/32_Confidence_0.390_Length_1557, vo_c3_Locus_1613_Transcript_6/17_Confidence_0.158_Length_5809, vo_n1_Locus_10193_Transcript_1/1_Confidence_0.000_Length_1647, vo_c2_Locus_6348_Transcript_1/2_Confidence_1.000_Length_3755, vo_n3_Locus_23556_Transcript_2/4_Confidence_0.500_Length_4210, tri_c_comp43537_c0_seq1, vo_n3_Locus_9269_Transcript_1/1_Confidence_0.000_Length_2238, vo_c3_Locus_550_Transcript_25/30_Confidence_0.321_Length_1640, vo_c3_Locus_550_Transcript_10/30_Confidence_0.420_Length_1515, vo_n1_Locus_133_Transcript_12/30_Confidence_0.033_Length_580, vo_n2_Locus_13049_Transcript_20/20_Confidence_0.289_Length_1233, vo_c3_Locus_1015_Transcript_30/32_Confidence_0.181_Length_1172, vo_n2_Locus_13049_Transcript_19/20_Confidence_0.368_Length_1268, vo_c3_Locus_550_Transcript_27/30_Confidence_0.395_Length_1758, vo_n1_Locus_296_Transcript_24/31_Confidence_0.275_Length_1182, vo_n2_Locus_13049_Transcript_17/20_Confidence_0.342_Length_1329, vo_c3_Locus_1015_Transcript_13/32_Confidence_0.222_Length_1363, vo_c2_Locus_1551_Transcript_19/27_Confidence_0.222_Length_1248, vo_n1_Locus_296_Transcript_27/31_Confidence_0.078_Length_2886, vo_c3_Locus_1015_Transcript_3/32_Confidence_0.181_Length_1206, vo_n2_Locus_105_Transcript_30/33_Confidence_0.449_Length_1990, vo_c3_Locus_550_Transcript_28/30_Confidence_0.309_Length_1831,

					vo_n3_Locus_150_Transcript_17/25_Confidence_0.183_Length_1185, vo_n3_Locus_150_Transcript_6/25_Confidence_0.232_Length_1192, vo_n1_Locus_133_Transcript_30/30_Confidence_0.033_Length_2622, tri_c_comp21115_c0_seq1, vo_n3_Locus_587_Transcript_2/22_Confidence_0.111_Length_206, vo_c2_Locus_320_Transcript_13/32_Confidence_0.351_Length_2604, vo_n1_Locus_296_Transcript_25/31_Confidence_0.157_Length_1180, vo_n2_Locus_13049_Transcript_7/20_Confidence_0.421_Length_1354, vo_c1_Locus_223_Transcript_13/65_Confidence_0.197_Length_1636, vo_c1_Locus_223_Transcript_47/65_Confidence_0.099_Length_1259, vo_c2_Locus_320_Transcript_7/32_Confidence_0.299_Length_1642, vo_n1_Locus_133_Transcript_14/30_Confidence_0.456_Length_2738, vo_c3_Locus_1015_Transcript_12/32_Confidence_0.222_Length_1270, vo_c2_Locus_320_Transcript_9/32_Confidence_0.338_Length_1673, vo_c2_Locus_320_Transcript_22/32_Confidence_0.545_Length_1648, vo_n1_Locus_296_Transcript_23/31_Confidence_0.294_Length_1182, vo_n2_Locus_13049_Transcript_9/20_Confidence_0.316_Length_1224, vo_c1_Locus_223_Transcript_52/65_Confidence_0.042_Length_799, vo_c3_Locus_550_Transcript_30/30_Confidence_0.210_Length_1413, vo_n1_Locus_133_Transcript_13/30_Confidence_0.456_Length_2508, vo_n1_Locus_133_Transcript_24/30_Confidence_0.311_Length_1984, vo_n1_Locus_296_Transcript_30/31_Confidence_0.078_Length_1319, vo_n1_Locus_133_Transcript_15/30_Confidence_0.456_Length_2671, vo_c2_Locus_320_Transcript_20/32_Confidence_0.325_Length_1586, vo_c1_Locus_223_Transcript_19/65_Confidence_0.092_Length_1454, vo_n1_Locus_296_Transcript_10/31_Confidence_0.118_Length_1164, vo_c1_Locus_223_Transcript_54/65_Confidence_0.134_Length_1458, vo_c2_Locus_320_Transcript_15/32_Confidence_0.390_Length_1557, vo_c3_Locus_1015_Transcript_9/32_Confidence_0.236_Length_1272, vo_n1_Locus_133_Transcript_25/30_Confidence_0.278_Length_2179, vo_n3_Locus_6810_Transcript_2/3_Confidence_0.913_Length_1699, vo_n1_Locus_6460_Transcript_1/1_Confidence_0.000_Length_1912, vo_c2_Locus_3634_Transcript_8/8_Confidence_0.133_Length_2428, vo_c2_Locus_324_Transcript_5/12_Confidence_0.333_Length_1009, vo_n1_Locus_5721_Transcript_6/9_Confidence_0.389_Length_989, vo_c2_Locus_324_Transcript_11/12_Confidence_0.333_Length_4393, vo_n2_Locus_475_Transcript_7/13_Confidence_0.312_Length_1141, vo_n1_Locus_5721_Transcript_1/9_Confidence_0.278_Length_986, vo_n2_Locus_475_Transcript_2/13_Confidence_0.312_Length_1187, vo_c2_Locus_324_Transcript_2/12_Confidence_0.400_Length_1106, vo_c3_Locus_3927_Transcript_2/9_Confidence_0.250_Length_2629, vo_n2_Locus_11241_Transcript_1/3_Confidence_0.600_Length_3773, vo_c2_Locus_479_Transcript_1/2_Confidence_1.000_Length_2154, tri_c_comp11242_c0_seq2, tri_n_comp64914_c0_seq1, vo_c2_Locus_9062_Transcript_10/11_Confidence_0.312_Length_4945, vo_n2_Locus_22224_Transcript_1/1_Confidence_0.000_Length_1370, tri_n_comp14915_c0_seq1, vo_n3_Locus_15713_Transcript_4/7_Confidence_0.429_Length_2557, tri_c_comp37399_c0_seq1, tri_n_comp58061_c0_seq1, vo_n1_Locus_22736_Transcript_1/2_Confidence_0.333_Length_1213, vo_n2_Locus_19677_Transcript_1/1_Confidence_0.000_Length_2823, tri_c_comp55340_c0_seq1, tri_n_comp58061_c0_seq1, tri_c_comp55340_c0_seq1, vo_n1_Locus_19864_Transcript_2/2_Confidence_0.000_Length_2707, vo_c3_Locus_21741_Transcript_5/9_Confidence_0.583_Length_1757, vo_n3_Locus_25600_Transcript_5/6_Confidence_0.375_Length_1777, tri_n_comp58061_c0_seq1, vo_c1_Locus_223_Transcript_52/65_Confidence_0.042_Length_799, vo_n2_Locus_13049_Transcript_20/20_Confidence_0.289_Length_1233, vo_c3_Locus_1015_Transcript_30/32_Confidence_0.181_Length_1172, vo_n2_Locus_13049_Transcript_19/20_Confidence_0.368_Length_1268, vo_n2_Locus_13049_Transcript_17/20_Confidence_0.342_Length_1329, vo_c3_Locus_1015_Transcript_9/32_Confidence_0.236_Length_1272, vo_n2_Locus_13049_Transcript_9/20_Confidence_0.316_Length_1224, vo_c3_Locus_1015_Transcript_13/32_Confidence_0.222_Length_1363, vo_n2_Locus_13049_Transcript_7/20_Confidence_0.421_Length_1354, vo_n1_Locus_565_Transcript_31/31_Confidence_0.178_Length_3922, vo_c3_Locus_2800_Transcript_3/23_Confidence_0.566_Length_1690, vo_c3_Locus_2800_Transcript_9/23_Confidence_0.585_Length_1396, tri_n_comp21653_c0_seq1, vo_n1_Locus_565_Transcript_5/31_Confidence_0.244_Length_1533, vo_n2_Locus_4661_Transcript_5/12_Confidence_0.208_Length_1240, vo_n1_Locus_565_Transcript_17/31_Confidence_0.422_Length_1454, vo_c3_Locus_2800_Transcript_5/23_Confidence_0.566_Length_2133, vo_c1_Locus_1757_Transcript_17/19_Confidence_0.375_Length_1367, vo_c1_Locus_298_Transcript_4/32_Confidence_0.255_Length_1585, vo_n1_Locus_8402_Transcript_3/4_Confidence_0.286_Length_1464, vo_c3_Locus_2800_Transcript_23/23_Confidence_0.151_Length_1466, vo_n2_Locus_903_Transcript_38/40_Confidence_0.222_Length_6612, vo_n1_Locus_565_Transcript_20/31_Confidence_0.444_Length_1622, vo_n2_Locus_2046_Transcript_7/18_Confidence_0.345_Length_1296, vo_c3_Locus_2800_Transcript_8/23_Confidence_0.585_Length_1344, vo_n3_Locus_1119_Transcript_19/50_Confidence_0.050_Length_498, vo_c2_Locus_5742_Transcript_10/21_Confidence_0.244_Length_1454, vo_n3_Locus_1119_Transcript_11/50_Confidence_0.124_Length_2839, vo_c2_Locus_5742_Transcript_18/21_Confidence_0.317_Length_2233, vo_n1_Locus_565_Transcript_13/31_Confidence_0.378_Length_1400, vo_c3_Locus_2800_Transcript_14/23_Confidence_0.321_Length_1426, vo_c3_Locus_614_Transcript_19/24_Confidence_0.394_Length_3443, vo_c2_Locus_358_Transcript_1/41_Confidence_0.080_Length_1708, vo_c2_Locus_5742_Transcript_14/21_Confidence_0.439_Length_2413, vo_c1_Locus_1757_Transcript_8/19_Confidence_0.458_Length_1460, vo_n1_Locus_986_Transcript_16/36_Confidence_0.211_Length_1811, vo_n1_Locus_565_Transcript_16/31_Confidence_0.400_Length_1499, vo_n2_Locus_903_Transcript_37/40_Confidence_0.346_Length_3926, tri_c_comp20312_c0_seq4, vo_c3_Locus_614_Transcript_13/24_Confidence_0.061_Length_4970, vo_n2_Locus_2046_Transcript_11/18_Confidence_0.310_Length_1666, vo_n2_Locus_903_Transcript_31/40_Confidence_0.321_Length_2251, vo_n1_Locus_565_Transcript_30/31_Confidence_0.422_Length_3814, vo_c3_Locus_614_Transcript_2/24_Confidence_0.485_Length_1777, vo_n1_Locus_5148_Transcript_1/4_Confidence_0.667_Length_1983, vo_c1_Locus_10454_Transcript_1/2_Confidence_0.750_Length_1441, vo_n3_Locus_1119_Transcript_18/50_Confidence_0.149_Length_1959, tri_c_comp10536_c0_seq1, vo_c2_Locus_32726_Transcript_2/2_Confidence_0.000_Length_696, vo_c2_Locus_40594_Transcript_2/3_Confidence_0.500_Length_1728, vo_n2_Locus_39295_Transcript_2/2_Confidence_0.500_Length_1939, vo_c2_Locus_6460_Transcript_1/2_Confidence_1.000_Length_1563, vo_n1_Locus_3261_Transcript_3/4_Confidence_0.667_Length_1633, vo_n3_Locus_27_Transcript_1/1_Confidence_0.000_Length_1278, tri_c_comp23875_c0_seq1, tri_c_comp24147_c0_seq1, vo_c1_Locus_4576_Transcript_2/2_Confidence_0.000_Length_1644, tri_n_comp18813_c0_seq8,

					tri_c_comp19022_c0_seq2, vo_n2_Locus_106_Transcript_27/30_Confidence_0.078_Length_2735, vo_c2_Locus_2699_Transcript_2/7_Confidence_0.471_Length_1721, vo_n2_Locus_106_Transcript_24/30_Confidence_0.219_Length_3708, vo_n3_Locus_362_Transcript_1/4_Confidence_0.727_Length_3306, vo_n3_Locus_3996_Transcript_1/4_Confidence_0.417_Length_1642, vo_n1_Locus_3710_Transcript_9/10_Confidence_0.500_Length_1616, vo_n2_Locus_106_Transcript_25/30_Confidence_0.188_Length_2659, vo_n2_Locus_1384_Transcript_2/5_Confidence_0.308_Length_2123, vo_n3_Locus_3996_Transcript_3/4_Confidence_0.500_Length_3581, tri_n_comp18813_c0_seq3, vo_c1_Locus_6488_Transcript_4/4_Confidence_0.250_Length_8239, vo_c2_Locus_1551_Transcript_20/27_Confidence_0.238_Length_2634, tri_n_comp18813_c0_seq13, vo_n3_Locus_685_Transcript_7/8_Confidence_0.353_Length_1680, vo_n2_Locus_1324_Transcript_10/14_Confidence_0.296_Length_1766, vo_c3_Locus_1613_Transcript_6/17_Confidence_0.158_Length_5809, vo_n3_Locus_685_Transcript_3/8_Confidence_0.471_Length_1755, vo_c2_Locus_16827_Transcript_1/1_Confidence_0.000_Length_1783, vo_n3_Locus_362_Transcript_3/4_Confidence_0.364_Length_3895, vo_c3_Locus_1613_Transcript_11/17_Confidence_0.316_Length_2455, tri_c_comp13285_c0_seq1, tri_n_comp18813_c0_seq13, vo_c2_Locus_23037_Transcript_2/2_Confidence_0.000_Length_2109, vo_n3_Locus_362_Transcript_3/4_Confidence_0.364_Length_3895, vo_n3_Locus_362_Transcript_1/4_Confidence_0.727_Length_3306, vo_n1_Locus_5208_Transcript_2/2_Confidence_0.000_Length_2077, vo_c2_Locus_23037_Transcript_1/2_Confidence_0.750_Length_1965, vo_n3_Locus_15336_Transcript_1/1_Confidence_0.000_Length_1810

Alanine, aspartate and glutamate metabolism	map00250	20	ec:2.6.1.16 - transaminase (isomerizing), ec:2.6.1.19 - transaminase, ec:6.3.1.1 - ligase, ec:6.3.1.2 - synthetase, ec:1.2.1.24 - dehydrogenase (NAD+), ec:4.3.2.1 - lyase, ec:4.3.2.2 - lyase, ec:2.1.3.2 - carbamoyltransferase, ec:2.6.1.44 - transaminase, ec:1.2.1.16 - dehydrogenase [NAD(P)+], ec:2.6.1.1 - transaminase, ec:6.3.5.4 - synthase (glutamine-hydrolysing), ec:6.3.5.5 - synthase (glutamine-hydrolysing), ec:1.4.1.2 - dehydrogenase, ec:3.5.1.1 - asparaginase II, ec:6.3.4.4 - synthase, ec:6.3.4.5 - synthase, ec:6.3.4.16 - synthase (ammonia), ec:1.4.1.14 - synthase (NADH), ec:1.4.1.13 - synthase (NADPH)	1, 2, 1, 15, 1, 1, 1, 3, 2, 1, 4, 3, 2, 1, 3, 1, 1, 2, 15, 10	vo_n2_Locus_1408_Transcript_4/5_Confidence_0.222_Length_11768, vo_c3_Locus_29526_Transcript_7/10_Confidence_0.268_Length_874, vo_c2_Locus_844_Transcript_5/40_Confidence_0.162_Length_2081, vo_n1_Locus_210_Transcript_2/3_Confidence_0.571_Length_2158, vo_c1_Locus_1340_Transcript_12/17_Confidence_0.109_Length_2389, vo_c1_Locus_1340_Transcript_9/17_Confidence_0.172_Length_2429, tri_n_comp11684_c0_seq1, vo_c1_Locus_4136_Transcript_1/2_Confidence_0.967_Length_2259, vo_c2_Locus_6408_Transcript_2/14_Confidence_0.268_Length_1478, vo_n1_Locus_335_Transcript_2/3_Confidence_0.909_Length_2286, vo_c2_Locus_3512_Transcript_3/4_Confidence_0.500_Length_2501, vo_n2_Locus_4820_Transcript_2/3_Confidence_0.545_Length_2376, vo_n3_Locus_2644_Transcript_11/11_Confidence_0.323_Length_2390, vo_n2_Locus_515_Transcript_8/9_Confidence_0.471_Length_3194, vo_n2_Locus_515_Transcript_7/9_Confidence_0.176_Length_2355, vo_c2_Locus_3512_Transcript_2/4_Confidence_0.750_Length_2320, vo_c3_Locus_1501_Transcript_6/9_Confidence_0.203_Length_2420, vo_n2_Locus_5127_Transcript_1/3_Confidence_0.200_Length_1636, vo_n2_Locus_22912_Transcript_1/2_Confidence_0.333_Length_2481, vo_c1_Locus_24159_Transcript_1/2_Confidence_0.333_Length_1573, vo_c2_Locus_18300_Transcript_2/3_Confidence_0.200_Length_1671, vo_n2_Locus_9167_Transcript_1/4_Confidence_0.286_Length_6748, tri_n_comp27462_c0_seq1, tri_c_comp33901_c0_seq1, vo_n2_Locus_5805_Transcript_1/2_Confidence_0.333_Length_5227, vo_c3_Locus_33872_Transcript_1/1_Confidence_0.000_Length_1446, vo_c1_Locus_7220_Transcript_2/3_Confidence_0.400_Length_2898, vo_c1_Locus_24159_Transcript_1/2_Confidence_0.333_Length_1573, vo_n1_Locus_3423_Transcript_2/4_Confidence_0.250_Length_1347, tri_n_comp27683_c0_seq1, vo_c1_Locus_3127_Transcript_1/1_Confidence_0.000_Length_1478, vo_n1_Locus_3423_Transcript_3/4_Confidence_0.250_Length_1416, vo_c3_Locus_29578_Transcript_2/4_Confidence_0.800_Length_3844, vo_c1_Locus_12300_Transcript_2/4_Confidence_0.375_Length_3857, vo_n1_Locus_210_Transcript_2/3_Confidence_0.571_Length_2158, tri_c_comp33901_c0_seq1, vo_n2_Locus_5805_Transcript_1/2_Confidence_0.333_Length_5227, tri_c_comp28026_c0_seq1, tri_c_comp3336_c0_seq2, tri_n_comp8685_c0_seq2, tri_c_comp3336_c0_seq1, tri_n_comp32103_c0_seq1, vo_c2_Locus_2824_Transcript_1/6_Confidence_0.533_Length_1586, tri_c_comp33901_c0_seq1, vo_n2_Locus_5805_Transcript_1/2_Confidence_0.333_Length_5227, vo_c1_Locus_13166_Transcript_27/32_Confidence_0.394_Length_6627, vo_c3_Locus_3082_Transcript_7/7_Confidence_0.167_Length_5439, vo_n1_Locus_991_Transcript_1/21_Confidence_0.113_Length_1473, tri_c_comp19198_c0_seq1, vo_n3_Locus_4402_Transcript_3/18_Confidence_0.269_Length_6798, vo_c2_Locus_4368_Transcript_2/25_Confidence_0.375_Length_6775, vo_n2_Locus_3_Transcript_20516/24291_Confidence_0.000_Length_6564, vo_n3_Locus_4402_Transcript_4/18_Confidence_0.077_Length_491, vo_c1_Locus_13166_Transcript_10/32_Confidence_0.076_Length_628, vo_c1_Locus_13166_Transcript_28/32_Confidence_0.409_Length_6630, vo_n1_Locus_7772_Transcript_1/1_Confidence_0.000_Length_6986, tri_c_comp19198_c0_seq4, tri_c_comp19198_c0_seq3, vo_c3_Locus_3082_Transcript_5/7_Confidence_0.333_Length_2051, vo_c1_Locus_1115_Transcript_3/3_Confidence_0.000_Length_916, vo_c2_Locus_4368_Transcript_2/25_Confidence_0.375_Length_6775, vo_c1_Locus_13166_Transcript_27/32_Confidence_0.394_Length_6627, vo_n2_Locus_3_Transcript_20516/24291_Confidence_0.000_Length_6564, vo_c3_Locus_3082_Transcript_7/7_Confidence_0.167_Length_5439, vo_n1_Locus_991_Transcript_1/21_Confidence_0.113_Length_1473, tri_c_comp19198_c0_seq1, vo_c1_Locus_13166_Transcript_28/32_Confidence_0.409_Length_6630, vo_n1_Locus_7772_Transcript_1/1_Confidence_0.000_Length_6986, vo_n3_Locus_4402_Transcript_3/18_Confidence_0.269_Length_6798, vo_c3_Locus_3082_Transcript_5/7_Confidence_0.333_Length_2051

Propanoate metabolism	map00640	20	ec:6.4.1.2 - carboxylase, ec:2.6.1.18 - transaminase, ec:2.6.1.19 - transaminase, ec:4.1.3.30 - lyase, ec:1.2.1.27 - dehydrogenase (CoA-acylating), ec:6.2.1.5 - ligase (ADP-forming), ec:6.2.1.4 - ligase (GDP-forming), ec:6.2.1.1 - ligase, ec:1.2.1.18 - dehydrogenase (acetylating), ec:4.2.1.79 - dehydratase, ec:4.2.1.99 - dehydratase, ec:1.8.1.4 - dehydrogenase, ec:2.3.1.9 - C-acetyltransferase, ec:4.1.1.9 - decarboxylase, ec:2.3.1.54 - C-acetyltransferase, ec:2.8.3.8 - CoA-transferase, ec:3.1.2.4 - hydrolase, ec:4.2.1.17 - hydratase, ec:1.1.1.202 - dehydrogenase, ec:1.2.4.4 - dehydrogenase (2-methylpropanoyl-transferring)	16, 1, 2, 3, 3, 28, 6, 1, 3, 1, 7, 2, 2, 1, 9, 1, 1, 7, 1, 1	vo_n3_Locus_484_Transcript_6/12_Confidence_0.250_Length_1533, vo_c3_Locus_17332_Transcript_1/1_Confidence_0.333_Length_6493, vo_n3_Locus_696_Transcript_20/35_Confidence_0.473_Length_5396, vo_c3_Locus_2164_Transcript_38/39_Confidence_0.292_Length_6879, tri_n_comp41552_c0_seq1, vo_c2_Locus_1509_Transcript_1/47_Confidence_0.149_Length_2731, vo_c2_Locus_2193_Transcript_1/3_Confidence_0.667_Length_1218, vo_c1_Locus_860_Transcript_10/29_Confidence_0.571_Length_6744, vo_c2_Locus_1509_Transcript_11/47_Confidence_0.164_Length_2686, vo_n2_Locus_1751_Transcript_51/59_Confidence_0.207_Length_3566, vo_n2_Locus_15097_Transcript_1/1_Confidence_0.000_Length_3935, vo_n3_Locus_484_Transcript_5/12_Confidence_0.250_Length_1451, vo_c1_Locus_860_Transcript_11/29_Confidence_0.571_Length_6741, tri_c_comp19913_c0_seq3, vo_n2_Locus_1751_Transcript_37/59_Confidence_0.195_Length_3437, vo_c2_Locus_1509_Transcript_6/47_Confidence_0.149_Length_2592, vo_c1_Locus_7220_Transcript_2/3_Confidence_0.400_Length_2898, vo_c3_Locus_29526_Transcript_7/10_Confidence_0.268_Length_874, vo_c2_Locus_844_Transcript_5/40_Confidence_0.162_Length_2081, vo_c3_Locus_145_Transcript_31/32_Confidence_0.014_Length_533, vo_n1_Locus_1163_Transcript_28/49_Confidence_0.319_Length_2335, vo_n2_Locus_29491_Transcript_1/2_Confidence_0.333_Length_1144, vo_c3_Locus_1581_Transcript_3/3_Confidence_0.000_Length_1736, vo_c1_Locus_1298_Transcript_3/6_Confidence_0.625_Length_1775, vo_n2_Locus_157_Transcript_2/4_Confidence_0.286_Length_2059, vo_c1_Locus_2207_Transcript_20/51_Confidence_0.393_Length_3556, vo_c3_Locus_2283_Transcript_28/38_Confidence_0.331_Length_3501, vo_n3_Locus_1164_Transcript_30/46_Confidence_0.400_Length_3745, tri_c_comp20184_c0_seq2, vo_c2_Locus_7270_Transcript_1/1_Confidence_0.500_Length_1488, vo_c1_Locus_31_Transcript_859/1155_Confidence_0.002_Length_1257, vo_n3_Locus_1164_Transcript_32/46_Confidence_0.456_Length_3745, vo_n1_Locus_3448_Transcript_46/64_Confidence_0.085_Length_783, vo_c2_Locus_644_Transcript_12/40_Confidence_0.459_Length_3665, vo_n3_Locus_1164_Transcript_35/46_Confidence_0.511_Length_3745, vo_c3_Locus_5_Transcript_229/353_Confidence_0.004_Length_952, vo_c2_Locus_644_Transcript_3/40_Confidence_0.071_Length_957, vo_n1_Locus_3448_Transcript_53/64_Confidence_0.225_Length_3567, vo_n2_Locus_386_Transcript_32/33_Confidence_0.208_Length_3664, vo_n1_Locus_3448_Transcript_63/64_Confidence_0.318_Length_3637, vo_n3_Locus_1164_Transcript_33/46_Confidence_0.456_Length_3748, vo_c3_Locus_2283_Transcript_26/38_Confidence_0.285_Length_3501, vo_n2_Locus_386_Transcript_22/33_Confidence_0.376_Length_3568, vo_c3_Locus_2283_Transcript_29/38_Confidence_0.292_Length_3501, vo_n2_Locus_386_Transcript_1/33_Confidence_0.139_Length_3600, vo_c2_Locus_644_Transcript_24/40_Confidence_0.398_Length_3511, vo_n3_Locus_1164_Transcript_43/46_Confidence_0.389_Length_3338, vo_c1_Locus_2207_Transcript_39/51_Confidence_0.250_Length_3586, vo_c3_Locus_79_Transcript_1/3_Confidence_0.200_Length_5222, vo_c2_Locus_52_Transcript_234/466_Confidence_0.003_Length_1462, vo_n2_Locus_386_Transcript_10/33_Confidence_0.376_Length_3676, vo_n1_Locus_1394_Transcript_2/3_Confidence_0.400_Length_2232, tri_n_comp19507_c0_seq6, vo_c3_Locus_79_Transcript_1/3_Confidence_0.200_Length_5222, vo_n1_Locus_1394_Transcript_2/3_Confidence_0.400_Length_2232, vo_n2_Locus_2182_Transcript_14/15_Confidence_0.458_Length_3129, vo_c1_Locus_1462_Transcript_1/46_Confidence_0.182_Length_2129, vo_c1_Locus_1462_Transcript_8/46_Confidence_0.530_Length_9104, vo_c2_Locus_642_Transcript_3/10_Confidence_0.571_Length_2141, vo_n3_Locus_9269_Transcript_1/1_Confidence_0.000_Length_2238, vo_c3_Locus_1581_Transcript_3/3_Confidence_0.000_Length_1736, vo_c1_Locus_1298_Transcript_3/6_Confidence_0.625_Length_1775, vo_n2_Locus_157_Transcript_2/4_Confidence_0.286_Length_2059, vo_n2_Locus_3116_Transcript_1/3_Confidence_0.750_Length_2885, tri_n_comp15079_c0_seq3, vo_c1_Locus_2380_Transcript_17/17_Confidence_0.086_Length_3951, vo_c2_Locus_191_Transcript_2/2_Confidence_0.167_Length_3106, tri_c_comp21028_c0_seq2, tri_c_comp21028_c0_seq3, vo_c2_Locus_354_Transcript_3/17_Confidence_0.667_Length_4966, vo_n1_Locus_30469_Transcript_2/2_Confidence_0.000_Length_979, vo_n3_Locus_6810_Transcript_2/3_Confidence_0.913_Length_1699, vo_n1_Locus_6460_Transcript_1/1_Confidence_0.000_Length_1912, vo_c1_Locus_2657_Transcript_1/1_Confidence_0.000_Length_1513, vo_n2_Locus_23058_Transcript_1/1_Confidence_0.000_Length_1342, vo_c2_Locus_22623_Transcript_1/1_Confidence_0.000_Length_1765, vo_c3_Locus_1564_Transcript_1/27_Confidence_0.474_Length_3491, vo_n2_Locus_4549_Transcript_18/24_Confidence_0.395_Length_3486, vo_c1_Locus_1748_Transcript_10/39_Confidence_0.197_Length_3498, vo_c1_Locus_1748_Transcript_19/39_Confidence_0.211_Length_3788, vo_c1_Locus_1748_Transcript_30/39_Confidence_0.282_Length_4645, vo_n2_Locus_4549_Transcript_11/24_Confidence_0.474_Length_3796, vo_n1_Locus_103_Transcript_17/28_Confidence_0.483_Length_3469, vo_n3_Locus_2375_Transcript_13/19_Confidence_0.467_Length_3465, vo_c3_Locus_1564_Transcript_22/27_Confidence_0.526_Length_3527, vo_c2_Locus_6864_Transcript_3/5_Confidence_0.667_Length_1701, vo_c3_Locus_6234_Transcript_1/1_Confidence_0.000_Length_1326, vo_c2_Locus_2261_Transcript_1/2_Confidence_1.000_Length_2564, vo_c3_Locus_5948_Transcript_1/1_Confidence_0.000_Length_1027, vo_n2_Locus_16945_Transcript_1/3_Confidence_0.500_Length_1084, vo_c3_Locus_9925_Transcript_8/11_Confidence_0.263_Length_3550, vo_n1_Locus_11137_Transcript_5/5_Confidence_0.143_Length_2829, vo_n2_Locus_16945_Transcript_3/3_Confidence_0.000_Length_746, vo_n2_Locus_30747_Transcript_1/1_Confidence_0.000_Length_951, vo_n2_Locus_7435_Transcript_1/1_Confidence_0.000_Length_1552, vo_c1_Locus_5714_Transcript_2/3_Confidence_0.714_Length_2588

Aminoacyl-tRNA biosynthesis	map00970	19	ec:6.1.1.6 - ligase, ec:6.1.1.7 - ligase, ec:6.1.1.9 - ligase, ec:6.1.1.2 - ligase, ec:6.1.1.3 - ligase, ec:6.1.1.4 - ligase, ec:6.1.1.5 - ligase, ec:6.1.1.18 - ligase, ec:6.1.1.17 - ligase, ec:6.1.1.19 - ligase, ec:6.1.1.14 - ligase, ec:6.1.1.16 - ligase, ec:6.1.1.15 - ligase, ec:6.1.1.10 - ligase, ec:6.1.1.12 - ligase, ec:6.1.1.11 - ligase, ec:6.1.1.21 - ligase, ec:6.1.1.20 - ligase, ec:6.1.1.22 - ligase	4, 2, 1, 4, 3, 1, 2, 1, 5, 1, 3, 4, 3, 1, 3, 6, 2, 4, 2	vo_c2_Locus_16703_Transcript_2/2_Confidence_0.000_Length_2117, tri_n_comp27405_c0_seq1, vo_c1_Locus_5594_Transcript_1/2_Confidence_0.500_Length_3744, vo_c2_Locus_17209_Transcript_1/2_Confidence_0.750_Length_2003, vo_c1_Locus_5547_Transcript_4/5_Confidence_0.300_Length_3117, vo_n2_Locus_16525_Transcript_2/3_Confidence_0.500_Length_3475, vo_c3_Locus_3849_Transcript_3/4_Confidence_0.250_Length_3311, tri_n_comp86678_c0_seq1, vo_n2_Locus_13581_Transcript_1/1_Confidence_0.000_Length_1796, tri_c_comp39733_c0_seq1, vo_n1_Locus_3026_Transcript_2/3_Confidence_0.400_Length_2292, vo_c3_Locus_928_Transcript_4/4_Confidence_0.000_Length_2840, vo_c3_Locus_928_Transcript_3/4_Confidence_0.667_Length_2754, tri_n_comp27258_c0_seq1, tri_c_comp46022_c0_seq1, vo_n2_Locus_4121_Transcript_4/4_Confidence_0.167_Length_4300, vo_c2_Locus_17767_Transcript_1/1_Confidence_0.000_Length_3269, vo_c1_Locus_2898_Transcript_1/1_Confidence_0.000_Length_2284, tri_n_comp17763_c0_seq2, tri_n_comp33248_c0_seq1, vo_n1_Locus_24951_Transcript_1/2_Confidence_0.750_Length_1980, vo_n1_Locus_24951_Transcript_2/2_Confidence_0.000_Length_2025, vo_n3_Locus_8238_Transcript_3/4_Confidence_0.750_Length_2102, vo_c3_Locus_22675_Transcript_1/4_Confidence_0.625_Length_2149, vo_c3_Locus_8275_Transcript_2/3_Confidence_0.750_Length_2028, vo_c3_Locus_19664_Transcript_2/6_Confidence_0.583_Length_2143, tri_c_comp34188_c0_seq1, vo_c2_Locus_32225_Transcript_1/1_Confidence_0.000_Length_2315, tri_n_comp7418_c0_seq1, vo_n2_Locus_11287_Transcript_1/1_Confidence_0.000_Length_2751, vo_n2_Locus_12683_Transcript_2/2_Confidence_0.000_Length_965, vo_n3_Locus_8238_Transcript_3/4_Confidence_0.750_Length_2102, tri_n_comp17763_c0_seq2, tri_n_comp33248_c0_seq1, vo_c1_Locus_27004_Transcript_1/1_Confidence_0.000_Length_2081, tri_c_comp31460_c0_seq1, tri_n_comp11954_c0_seq2, vo_n2_Locus_4794_Transcript_3/3_Confidence_0.000_Length_966, vo_c2_Locus_6681_Transcript_4/4_Confidence_0.200_Length_4226, vo_c2_Locus_29740_Transcript_1/1_Confidence_0.000_Length_1633, tri_n_comp34210_c0_seq1, tri_n_comp27198_c0_seq1, vo_n2_Locus_20560_Transcript_1/1_Confidence_0.000_Length_1758, tri_n_comp7628_c0_seq1, vo_c3_Locus_8136_Transcript_1/1_Confidence_0.333_Length_1822, vo_n2_Locus_18588_Transcript_1/2_Confidence_0.429_Length_1692, vo_n2_Locus_276_Transcript_1/2_Confidence_1.000_Length_1715, vo_c2_Locus_2909_Transcript_2/3_Confidence_0.333_Length_2506, vo_c3_Locus_7748_Transcript_2/2_Confidence_0.333_Length_2296, vo_n2_Locus_14099_Transcript_1/1_Confidence_0.000_Length_2060, vo_c3_Locus_23099_Transcript_1/1_Confidence_0.000_Length_1800, vo_c2_Locus_7737_Transcript_1/1_Confidence_0.000_Length_1822

Carbon fixation pathways in prokaryotes	map00720	19	ec:6.4.1.1 - carboxylase, ec:6.4.1.2 - carboxylase, ec:1.3.5.1 - dehydrogenase, ec:4.1.3.24 - lyase, ec:3.5.4.9 - cyclohydrolase, ec:6.2.1.5 - ligase (ADP-forming), ec:6.2.1.1 - ligase, ec:2.3.1.9 - C-acetyltransferase, ec:1.5.1.5 - dehydrogenase (NADP+), ec:2.3.3.8 - citrate synthase, ec:6.3.4.3 - ligase, ec:1.5.1.20 - reductase [NAD(P)H], ec:4.2.1.3 - hydratase, ec:4.2.1.2 - hydratase, ec:4.2.1.17 - hydratase, ec:2.7.9.2 - water dikinase, ec:1.1.1.37 - dehydrogenase, ec:1.1.1.35 - dehydrogenase, ec:1.1.1.42 - dehydrogenase (NADP+)	1, 16, 11, 15, 2, 28, 1, 2, 2, 31, 2, 1, 8, 2, 7, 15, 24, 16, 10	vo_n2_Locus_15097_Transcript_1/1_Confidence_0.000_Length_3935, vo_n3_Locus_484_Transcript_6/12_Confidence_0.250_Length_1533, vo_c3_Locus_17332_Transcript_1/1_Confidence_0.333_Length_6493, vo_n3_Locus_696_Transcript_20/35_Confidence_0.473_Length_5396, vo_c3_Locus_2164_Transcript_38/39_Confidence_0.292_Length_6879, tri_n_comp41552_c0_seq1, vo_c2_Locus_1509_Transcript_1/47_Confidence_0.149_Length_2731, vo_c2_Locus_2193_Transcript_1/3_Confidence_0.667_Length_1218, vo_c1_Locus_860_Transcript_10/29_Confidence_0.571_Length_6744, vo_c2_Locus_1509_Transcript_11/47_Confidence_0.164_Length_2686, vo_n2_Locus_1751_Transcript_51/59_Confidence_0.207_Length_3566, vo_n2_Locus_15097_Transcript_1/1_Confidence_0.000_Length_3935, vo_n3_Locus_484_Transcript_5/12_Confidence_0.250_Length_1451, vo_c1_Locus_860_Transcript_11/29_Confidence_0.571_Length_6741, tri_c_comp19913_c0_seq3, vo_n2_Locus_1751_Transcript_37/59_Confidence_0.195_Length_3437, vo_c2_Locus_1509_Transcript_6/47_Confidence_0.149_Length_2592, vo_n2_Locus_5500_Transcript_5/5_Confidence_0.308_Length_1268, vo_n2_Locus_5500_Transcript_1/5_Confidence_0.308_Length_1100, tri_n_comp12494_c0_seq1, vo_n2_Locus_5500_Transcript_2/5_Confidence_0.538_Length_1312, vo_n2_Locus_2182_Transcript_14/15_Confidence_0.458_Length_3129, vo_c2_Locus_642_Transcript_3/10_Confidence_0.571_Length_2141, vo_n1_Locus_1508_Transcript_4/6_Confidence_0.429_Length_1979, vo_n1_Locus_2259_Transcript_6/13_Confidence_0.429_Length_1275, vo_n1_Locus_2259_Transcript_9/13_Confidence_0.429_Length_1314, vo_c1_Locus_1462_Transcript_1/46_Confidence_0.182_Length_2129, vo_c1_Locus_1462_Transcript_8/46_Confidence_0.530_Length_9104, vo_c1_Locus_9668_Transcript_5/11_Confidence_0.391_Length_1245, vo_c2_Locus_3988_Transcript_2/10_Confidence_0.500_Length_1233, vo_n3_Locus_4554_Transcript_5/8_Confidence_0.600_Length_1377, vo_c2_Locus_3988_Transcript_1/10_Confidence_0.607_Length_1361, vo_n1_Locus_2626_Transcript_19/26_Confidence_0.238_Length_3851, vo_c1_Locus_9668_Transcript_4/11_Confidence_0.435_Length_1360, vo_n1_Locus_2626_Transcript_15/26_Confidence_0.262_Length_1311, vo_c1_Locus_9668_Transcript_6/11_Confidence_0.348_Length_1630, vo_n2_Locus_3_Transcript_5064/24291_Confidence_0.000_Length_1233, vo_n3_Locus_4554_Transcript_6/8_Confidence_0.533_Length_1337, vo_c2_Locus_3988_Transcript_5/10_Confidence_0.536_Length_2785, vo_n1_Locus_2626_Transcript_18/26_Confidence_0.310_Length_2079, vo_c2_Locus_3988_Transcript_7/10_Confidence_0.500_Length_1273, vo_n2_Locus_3_Transcript_5069/24291_Confidence_0.000_Length_1240, vo_c2_Locus_3988_Transcript_9/10_Confidence_0.429_Length_1401, tri_n_comp31819_c0_seq1, vo_n2_Locus_10494_Transcript_3/4_Confidence_0.333_Length_2314, vo_c1_Locus_2207_Transcript_20/51_Confidence_0.393_Length_3556, vo_c3_Locus_2283_Transcript_28/38_Confidence_0.331_Length_3501, vo_n3_Locus_1164_Transcript_30/46_Confidence_0.400_Length_3745, tri_c_comp20184_c0_seq2, vo_c2_Locus_7270_Transcript_1/1_Confidence_0.500_Length_1488, vo_c1_Locus_31_Transcript_859/1155_Confidence_0.002_Length_1257, vo_n3_Locus_1164_Transcript_32/46_Confidence_0.456_Length_3745, vo_n1_Locus_3448_Transcript_46/64_Confidence_0.085_Length_783, vo_c2_Locus_644_Transcript_12/40_Confidence_0.459_Length_3665, vo_n3_Locus_1164_Transcript_35/46_Confidence_0.511_Length_3745, vo_c3_Locus_5_Transcript_229/353_Confidence_0.004_Length_952, vo_c2_Locus_644_Transcript_3/40_Confidence_0.071_Length_957, vo_n1_Locus_3448_Transcript_53/64_Confidence_0.225_Length_3567, vo_n2_Locus_386_Transcript_32/33_Confidence_0.208_Length_3664, vo_n1_Locus_3448_Transcript_63/64_Confidence_0.318_Length_3637, vo_n3_Locus_1164_Transcript_33/46_Confidence_0.456_Length_3748, vo_c3_Locus_2283_Transcript_26/38_Confidence_0.285_Length_3501, vo_n2_Locus_386_Transcript_22/33_Confidence_0.376_Length_3568, vo_c3_Locus_2283_Transcript_29/38_Confidence_0.292_Length_3501, vo_n2_Locus_386_Transcript_1/33_Confidence_0.139_Length_3600, vo_c2_Locus_644_Transcript_24/40_Confidence_0.398_Length_3511, vo_n3_Locus_1164_Transcript_43/46_Confidence_0.389_Length_3338, vo_c1_Locus_2207_Transcript_39/51_Confidence_0.250_Length_3586, vo_c3_Locus_79_Transcript_1/3_Confidence_0.200_Length_5222, vo_c2_Locus_52_Transcript_234/466_Confidence_0.003_Length_1462, vo_n2_Locus_386_Transcript_10/33_Confidence_0.376_Length_3676, vo_n1_Locus_1394_Transcript_2/3_Confidence_0.400_Length_2232, tri_n_comp19507_c0_seq6, vo_n3_Locus_9269_Transcript_1/1_Confidence_0.000_Length_2238, vo_c1_Locus_2657_Transcript_1/1_Confidence_0.000_Length_1513, vo_n2_Locus_23058_Transcript_1/1_Confidence_0.000_Length_1342, vo_n2_Locus_2550_Transcript_2/3_Confidence_0.636_Length_1302, tri_n_comp31819_c0_seq1, vo_c1_Locus_2207_Transcript_20/51_Confidence_0.393_Length_3556, vo_c3_Locus_2283_Transcript_28/38_Confidence_0.331_Length_3501, vo_n3_Locus_1164_Transcript_30/46_Confidence_0.400_Length_3745, tri_c_comp20184_c0_seq2, vo_n3_Locus_1164_Transcript_32/46_Confidence_0.456_Length_3745, vo_n1_Locus_3448_Transcript_1/64_Confidence_0.062_Length_478, vo_n1_Locus_3448_Transcript_46/64_Confidence_0.085_Length_783, vo_c2_Locus_644_Transcript_12/40_Confidence_0.459_Length_3665, vo_n3_Locus_1164_Transcript_35/46_Confidence_0.511_Length_3745, vo_c2_Locus_644_Transcript_3/40_Confidence_0.071_Length_957, vo_n1_Locus_3448_Transcript_53/64_Confidence_0.225_Length_3567, vo_n2_Locus_386_Transcript_32/33_Confidence_0.208_Length_3664, vo_n1_Locus_3448_Transcript_63/64_Confidence_0.318_Length_3637, vo_n3_Locus_1164_Transcript_33/46_Confidence_0.456_Length_3748, vo_c3_Locus_2283_Transcript_26/38_Confidence_0.285_Length_3501, vo_n2_Locus_386_Transcript_22/33_Confidence_0.376_Length_3568, vo_n1_Locus_3448_Transcript_2/64_Confidence_0.054_Length_402, vo_c3_Locus_2283_Transcript_29/38_Confidence_0.292_Length_3501, vo_c3_Locus_2283_Transcript_35/38_Confidence_0.400_Length_3582, vo_n2_Locus_386_Transcript_1/33_Confidence_0.139_Length_3600, vo_c2_Locus_644_Transcript_24/40_Confidence_0.398_Length_3511, vo_n3_Locus_1164_Transcript_43/46_Confidence_0.389_Length_3338, vo_c1_Locus_2207_Transcript_39/51_Confidence_0.250_Length_3586, vo_c3_Locus_79_Transcript_1/3_Confidence_0.200_Length_5222, vo_n2_Locus_386_Transcript_10/33_Confidence_0.376_Length_3676, vo_n1_Locus_1394_Transcript_2/3_Confidence_0.400_Length_2232, vo_c2_Locus_644_Transcript_6/40_Confidence_0.204_Length_3616, vo_c1_Locus_2207_Transcript_36/51_Confidence_0.402_Length_3407, vo_n2_Locus_386_Transcript_5/33_Confidence_0.040_Length_566, tri_n_comp19507_c0_seq6, vo_n1_Locus_3448_Transcript_55/64_Confidence_0.233_Length_3564, tri_n_comp31819_c0_seq1, vo_n2_Locus_10494_Transcript_3/4_Confidence_0.333_Length_2314, vo_c2_Locus_1465_Transcript_1/1_Confidence_0.000_Length_2684,

					tri_n_comp15079_c0_seq3, vo_c1_Locus_2380_Transcript_17/17_Confidence_0.086_Length_3951, vo_c2_Locus_191_Transcript_2/2_Confidence_0.167_Length_3106, vo_c1_Locus_2380_Transcript_3/17_Confidence_0.371_Length_12857, tri_c_comp21028_c0_seq2, tri_c_comp21028_c0_seq3, vo_c2_Locus_354_Transcript_3/17_Confidence_0.667_Length_4966, vo_n1_Locus_30469_Transcript_2/2_Confidence_0.000_Length_979, tri_n_comp8247_c0_seq2, vo_c3_Locus_1133_Transcript_4/9_Confidence_0.455_Length_1666, vo_c2_Locus_2261_Transcript_1/2_Confidence_1.000_Length_2564, vo_c3_Locus_5948_Transcript_1/1_Confidence_0.000_Length_1027, vo_n2_Locus_16945_Transcript_1/3_Confidence_0.500_Length_1084, vo_c3_Locus_9925_Transcript_8/11_Confidence_0.263_Length_3550, vo_n1_Locus_11137_Transcript_5/5_Confidence_0.143_Length_2829, vo_n2_Locus_16945_Transcript_3/3_Confidence_0.000_Length_746, vo_n2_Locus_30747_Transcript_1/1_Confidence_0.000_Length_951, vo_c1_Locus_1662_Transcript_19/44_Confidence_0.278_Length_3148, vo_n2_Locus_1146_Transcript_9/32_Confidence_0.492_Length_2897, vo_n3_Locus_3213_Transcript_1/29_Confidence_0.022_Length_515, vo_n1_Locus_1032_Transcript_1/39_Confidence_0.018_Length_458, tri_n_comp20108_c0_seq2, vo_c1_Locus_1662_Transcript_28/44_Confidence_0.200_Length_3087, vo_c2_Locus_49_Transcript_21/157_Confidence_0.007_Length_465, vo_c2_Locus_49_Transcript_31/157_Confidence_0.027_Length_1554, vo_c2_Locus_49_Transcript_20/157_Confidence_0.037_Length_3535, vo_c1_Locus_1662_Transcript_6/44_Confidence_0.211_Length_3160, vo_c2_Locus_49_Transcript_28/157_Confidence_0.071_Length_2983, vo_n1_Locus_1032_Transcript_5/39_Confidence_0.404_Length_3016, vo_n1_Locus_1032_Transcript_38/39_Confidence_0.228_Length_2924, vo_n1_Locus_1032_Transcript_8/39_Confidence_0.386_Length_2954, vo_c3_Locus_1031_Transcript_12/33_Confidence_0.385_Length_3145, vo_c3_Locus_1723_Transcript_29/37_Confidence_0.411_Length_1695, vo_n2_Locus_330_Transcript_25/38_Confidence_0.361_Length_1516, vo_c3_Locus_1723_Transcript_35/37_Confidence_0.464_Length_1702, vo_c1_Locus_15206_Transcript_3/3_Confidence_0.000_Length_1312, vo_c1_Locus_7371_Transcript_4/4_Confidence_0.600_Length_1352, vo_c3_Locus_1723_Transcript_37/37_Confidence_0.214_Length_1530, vo_n1_Locus_769_Transcript_28/41_Confidence_0.400_Length_1609, vo_n1_Locus_769_Transcript_9/41_Confidence_0.460_Length_1494, vo_c3_Locus_2965_Transcript_2/2_Confidence_0.250_Length_1327, vo_n2_Locus_330_Transcript_5/38_Confidence_0.328_Length_11715, vo_n1_Locus_9772_Transcript_1/2_Confidence_0.333_Length_1224, vo_c1_Locus_3603_Transcript_44/48_Confidence_0.492_Length_1696, vo_c2_Locus_4242_Transcript_2/3_Confidence_0.400_Length_1168, vo_n1_Locus_6649_Transcript_2/3_Confidence_0.500_Length_2124, vo_c1_Locus_7371_Transcript_1/4_Confidence_0.500_Length_1352, vo_n3_Locus_2381_Transcript_2/3_Confidence_0.500_Length_1330, vo_c3_Locus_1723_Transcript_16/37_Confidence_0.286_Length_1491, vo_n1_Locus_769_Transcript_36/41_Confidence_0.420_Length_1536, vo_n1_Locus_769_Transcript_40/41_Confidence_0.320_Length_1479, vo_n1_Locus_6649_Transcript_1/3_Confidence_0.750_Length_1883, vo_c1_Locus_3603_Transcript_26/48_Confidence_0.400_Length_1432, vo_n1_Locus_5348_Transcript_2/2_Confidence_0.200_Length_1608, vo_n3_Locus_3383_Transcript_35/37_Confidence_0.389_Length_1512, tri_c_comp20802_c0_seq1, vo_c2_Locus_2261_Transcript_1/2_Confidence_1.000_Length_2564, vo_n1_Locus_9414_Transcript_8/13_Confidence_0.167_Length_606, vo_c1_Locus_40339_Transcript_1/1_Confidence_0.000_Length_1318, vo_c3_Locus_2031_Transcript_8/8_Confidence_0.111_Length_1389, vo_n1_Locus_11137_Transcript_5/5_Confidence_0.143_Length_2829, vo_c1_Locus_4675_Transcript_3/3_Confidence_0.200_Length_1348, vo_n2_Locus_30747_Transcript_1/1_Confidence_0.000_Length_951, vo_n2_Locus_636_Transcript_13/16_Confidence_0.222_Length_768, vo_c1_Locus_2815_Transcript_3/4_Confidence_0.200_Length_2244, vo_c3_Locus_9925_Transcript_8/11_Confidence_0.263_Length_3550, tri_n_comp42565_c0_seq1, vo_c2_Locus_189_Transcript_29/60_Confidence_0.015_Length_692, vo_n2_Locus_636_Transcript_16/16_Confidence_0.139_Length_616, vo_n2_Locus_636_Transcript_14/16_Confidence_0.222_Length_622, vo_n2_Locus_636_Transcript_5/16_Confidence_0.194_Length_591, vo_n2_Locus_636_Transcript_3/16_Confidence_0.306_Length_685, vo_c3_Locus_6304_Transcript_1/17_Confidence_0.159_Length_2915, vo_n2_Locus_353_Transcript_7/18_Confidence_0.420_Length_2960, vo_c1_Locus_5705_Transcript_18/19_Confidence_0.191_Length_3822, vo_n2_Locus_353_Transcript_14/18_Confidence_0.280_Length_2879, vo_n1_Locus_2903_Transcript_2/3_Confidence_0.400_Length_1386, vo_n1_Locus_464_Transcript_11/22_Confidence_0.362_Length_3670, vo_c2_Locus_11026_Transcript_2/2_Confidence_0.000_Length_1965, vo_c3_Locus_6304_Transcript_5/17_Confidence_0.477_Length_2995, vo_c1_Locus_5705_Transcript_16/19_Confidence_0.489_Length_4193, vo_c3_Locus_6304_Transcript_2/17_Confidence_0.523_Length_3712

Carbon fixation in photosynthetic organisms	map00710	18	ec:4.1.1.49 - carboxykinase (ATP), ec:4.1.1.39 - carboxylase, ec:2.2.1.1 - glycolaldehydetransferase, ec:2.7.2.3 - kinase, ec:1.2.1.59 - dehydrogenase (NAD(P)+) (phosphorylating), ec:2.7.1.19 - phosphopentokinase, ec:1.2.1.12 - dehydrogenase (phosphorylating), ec:5.3.1.1 - isomerase, ec:5.3.1.6 - isomerase, ec:2.6.1.1 - transaminase, ec:4.1.2.13 - aldolase, ec:3.1.3.37 - SBPase, ec:5.1.3.1 - 3-epimerase, ec:3.1.3.11 - hexose diphosphatase, ec:1.1.1.82 - dehydrogenase (NADP+), ec:1.1.1.37 - dehydrogenase, ec:1.1.1.39 - dehydrogenase (decarboxylating), ec:1.1.1.40 - dehydrogenase (oxaloacetate-decarboxylating) (NADP+)	5, 95, 24, 41, 16, 25, 45, 9, 2, 4, 38, 3, 3, 8, 23, 24, 11, 7	vo_c1_Locus_2295_Transcript_9/10_Confidence_0.238_Length_2213, vo_c2_Locus_2862_Transcript_9/10_Confidence_0.250_Length_2209, vo_c1_Locus_2295_Transcript_3/10_Confidence_0.524_Length_2330, vo_n2_Locus_1690_Transcript_8/11_Confidence_0.429_Length_2234, vo_n2_Locus_1690_Transcript_9/11_Confidence_0.429_Length_2277, vo_c1_Locus_1636_Transcript_17/122_Confidence_0.157_Length_2013, vo_n3_Locus_37_Transcript_16/46_Confidence_0.281_Length_1685, vo_n2_Locus_3_Transcript_22527/24291_Confidence_0.000_Length_1609, vo_n3_Locus_37_Transcript_10/46_Confidence_0.207_Length_1727, vo_n3_Locus_37_Transcript_9/46_Confidence_0.215_Length_1812, vo_c1_Locus_1636_Transcript_122/122_Confidence_0.108_Length_2335, vo_n2_Locus_3_Transcript_22541/24291_Confidence_0.000_Length_288, vo_n2_Locus_3_Transcript_22550/24291_Confidence_0.000_Length_1791, vo_n3_Locus_37_Transcript_33/46_Confidence_0.163_Length_732, vo_n2_Locus_3_Transcript_22526/24291_Confidence_0.000_Length_620, vo_n2_Locus_3_Transcript_22511/24291_Confidence_0.000_Length_798, vo_c1_Locus_1636_Transcript_15/122_Confidence_0.065_Length_512, vo_c2_Locus_52_Transcript_144/466_Confidence_0.025_Length_2131, tri_n_comp20144_c0_seq14, vo_c2_Locus_52_Transcript_133/466_Confidence_0.023_Length_2364, tri_n_comp20144_c0_seq13, vo_n3_Locus_41731_Transcript_1/1_Confidence_0.500_Length_459, vo_c2_Locus_52_Transcript_132/466_Confidence_0.016_Length_1024, vo_n1_Locus_55_Transcript_52/66_Confidence_0.069_Length_313, tri_c_comp20343_c0_seq9, tri_n_comp20144_c0_seq10, tri_n_comp20144_c0_seq7, tri_n_comp20144_c0_seq6, vo_c3_Locus_31_Transcript_6/79_Confidence_0.152_Length_709, tri_n_comp20144_c0_seq3, tri_n_comp20144_c0_seq4, tri_n_comp20144_c0_seq1, vo_c1_Locus_1636_Transcript_86/122_Confidence_0.182_Length_2244, vo_c1_Locus_1636_Transcript_73/122_Confidence_0.222_Length_1747, vo_c2_Locus_52_Transcript_164/466_Confidence_0.011_Length_2810, vo_n2_Locus_3_Transcript_22535/24291_Confidence_0.000_Length_656, vo_n2_Locus_3_Transcript_22546/24291_Confidence_0.000_Length_1498, vo_c3_Locus_31_Transcript_74/79_Confidence_0.124_Length_2769, vo_c3_Locus_31_Transcript_68/79_Confidence_0.124_Length_1224, vo_n3_Locus_37_Transcript_21/46_Confidence_0.148_Length_1139, vo_c3_Locus_31_Transcript_67/79_Confidence_0.152_Length_2229, vo_n3_Locus_37_Transcript_14/46_Confidence_0.259_Length_5942, vo_n1_Locus_55_Transcript_3/66_Confidence_0.063_Length_450, tri_c_comp20343_c0_seq4, tri_c_comp20343_c0_seq3, vo_n2_Locus_3_Transcript_22566/24291_Confidence_0.000_Length_2168, vo_n2_Locus_3452_Transcript_2/4_Confidence_0.636_Length_263, vo_n2_Locus_3_Transcript_22486/24291_Confidence_0.000_Length_1883, vo_c1_Locus_1636_Transcript_120/122_Confidence_0.135_Length_3625, vo_c2_Locus_52_Transcript_120/466_Confidence_0.009_Length_610, vo_c3_Locus_31_Transcript_13/79_Confidence_0.099_Length_748, vo_c1_Locus_1636_Transcript_69/122_Confidence_0.074_Length_844, vo_n2_Locus_3_Transcript_22520/24291_Confidence_0.000_Length_1452, vo_n2_Locus_3_Transcript_22533/24291_Confidence_0.000_Length_679, vo_n2_Locus_3_Transcript_22537/24291_Confidence_0.000_Length_840, vo_c3_Locus_31_Transcript_63/79_Confidence_0.148_Length_3613, vo_c1_Locus_1636_Transcript_71/122_Confidence_0.055_Length_846, vo_c3_Locus_31_Transcript_77/79_Confidence_0.297_Length_1736, vo_c1_Locus_1636_Transcript_83/122_Confidence_0.191_Length_2430, tri_n_comp20833_c0_seq3, vo_c3_Locus_31_Transcript_75/79_Confidence_0.212_Length_8463, vo_c2_Locus_52_Transcript_153/466_Confidence_0.026_Length_1551, vo_c3_Locus_31_Transcript_3/79_Confidence_0.035_Length_301, vo_n2_Locus_3_Transcript_22489/24291_Confidence_0.000_Length_274, vo_n2_Locus_3_Transcript_22509/24291_Confidence_0.000_Length_800, vo_n3_Locus_37_Transcript_38/46_Confidence_0.163_Length_922, tri_c_comp21288_c0_seq1, vo_c1_Locus_1636_Transcript_88/122_Confidence_0.074_Length_1384, vo_c1_Locus_1636_Transcript_121/122_Confidence_0.018_Length_960, vo_n3_Locus_37_Transcript_5/46_Confidence_0.289_Length_1505, vo_n1_Locus_55_Transcript_33/66_Confidence_0.040_Length_1095, vo_c3_Locus_31_Transcript_65/79_Confidence_0.166_Length_2369, vo_c3_Locus_31_Transcript_73/79_Confidence_0.208_Length_2557, vo_c3_Locus_31_Transcript_9/79_Confidence_0.152_Length_714, vo_c2_Locus_52_Transcript_113/466_Confidence_0.004_Length_590, vo_n2_Locus_3_Transcript_22492/24291_Confidence_0.000_Length_1595, vo_n1_Locus_55_Transcript_54/66_Confidence_0.183_Length_2185, vo_c3_Locus_33_Transcript_2/2_Confidence_0.714_Length_611, vo_c2_Locus_52_Transcript_125/466_Confidence_0.007_Length_559, vo_c2_Locus_52_Transcript_135/466_Confidence_0.025_Length_2711, vo_n1_Locus_55_Transcript_12/66_Confidence_0.331_Length_2232, vo_c1_Locus_1636_Transcript_100/122_Confidence_0.031_Length_685, vo_c2_Locus_52_Transcript_131/466_Confidence_0.023_Length_1964, vo_c3_Locus_31_Transcript_7/79_Confidence_0.184_Length_710, vo_n1_Locus_55_Transcript_39/66_Confidence_0.194_Length_1391, vo_n3_Locus_37_Transcript_28/46_Confidence_0.193_Length_1043, vo_n1_Locus_55_Transcript_41/66_Confidence_0.263_Length_1742, vo_n1_Locus_55_Transcript_38/66_Confidence_0.171_Length_1387, vo_n2_Locus_3_Transcript_22491/24291_Confidence_0.000_Length_1174, vo_n3_Locus_37_Transcript_30/46_Confidence_0.178_Length_1043, vo_n2_Locus_3_Transcript_22503/24291_Confidence_0.000_Length_721, vo_n2_Locus_3_Transcript_22540/24291_Confidence_0.000_Length_271, vo_c2_Locus_52_Transcript_128/466_Confidence_0.003_Length_564, vo_c2_Locus_52_Transcript_162/466_Confidence_0.019_Length_1668, vo_n2_Locus_3_Transcript_22534/24291_Confidence_0.000_Length_449, vo_c1_Locus_1636_Transcript_116/122_Confidence_0.129_Length_3526, vo_c3_Locus_31_Transcript_70/79_Confidence_0.208_Length_1586, vo_n1_Locus_55_Transcript_9/66_Confidence_0.349_Length_2172, vo_c1_Locus_8362_Transcript_4/9_Confidence_0.254_Length_482, vo_n1_Locus_55_Transcript_50/66_Confidence_0.166_Length_2379, vo_c3_Locus_1314_Transcript_17/46_Confidence_0.365_Length_2530, vo_n3_Locus_387_Transcript_34/39_Confidence_0.386_Length_2514, vo_c2_Locus_924_Transcript_20/40_Confidence_0.452_Length_2520, vo_c2_Locus_924_Transcript_13/40_Confidence_0.310_Length_2559, vo_c3_Locus_1314_Transcript_33/46_Confidence_0.292_Length_2524, vo_c1_Locus_1204_Transcript_14/17_Confidence_0.394_Length_4476, vo_c2_Locus_924_Transcript_19/40_Confidence_0.333_Length_2562, vo_n1_Locus_327_Transcript_13/41_Confidence_0.275_Length_2480, vo_c3_Locus_1314_Transcript_6/46_Confidence_0.208_Length_1518, vo_n1_Locus_327_Transcript_40/41_Confidence_0.176_Length_2442, vo_c3_Locus_1314_Transcript_38/46_Confidence_0.302_Length_2522, vo_c2_Locus_924_Transcript_34/40_Confidence_0.369_Length_2618, vo_c3_Locus_1314_Transcript_14/46_Confidence_0.229_Length_3828, vo_c2_Locus_924_Transcript_16/40_Confidence_0.321_Length_2559, vo_c2_Locus_924_Transcript_23/40_Confidence_0.488_Length_2850, vo_c3_Locus_1314_Transcript_21/46_Confidence_0.365_Length_2524, vo_c1_Locus_1204_Transcript_13/17_Confidence_0.364_Length_4497, vo_n1_Locus_327_Transcript_24/41_Confidence_0.385_Length_2547,

					vo_c2_Locus_924_Transcript_17/40_Confidence_0.333_Length_2453, tri_c_comp20608_c0_seq4, tri_c_comp20608_c0_seq5, vo_c3_Locus_1314_Transcript_43/46_Confidence_0.198_Length_2519, vo_n2_Locus_2054_Transcript_26/37_Confidence_0.448_Length_2522, tri_c_comp20608_c0_seq7, vo_n3_Locus_2623_Transcript_14/17_Confidence_0.556_Length_1555, vo_n3_Locus_74_Transcript_17/25_Confidence_0.436_Length_1764, vo_c1_Locus_788_Transcript_17/34_Confidence_0.404_Length_1861, vo_c1_Locus_788_Transcript_30/34_Confidence_0.255_Length_1712, tri_c_comp19022_c0_seq2, vo_c3_Locus_239_Transcript_24/29_Confidence_0.409_Length_3108, vo_c2_Locus_298_Transcript_10/24_Confidence_0.500_Length_1796, tri_n_comp18813_c0_seq15, vo_n2_Locus_106_Transcript_17/30_Confidence_0.344_Length_2068, tri_n_comp18813_c0_seq11, vo_n1_Locus_2248_Transcript_15/40_Confidence_0.275_Length_1844, tri_n_comp18813_c0_seq10, tri_n_comp20156_c0_seq1, vo_c2_Locus_3634_Transcript_2/8_Confidence_0.467_Length_2376, vo_c1_Locus_788_Transcript_5/34_Confidence_0.340_Length_1423, vo_n2_Locus_2252_Transcript_14/26_Confidence_0.410_Length_2227, vo_c3_Locus_239_Transcript_16/29_Confidence_0.364_Length_1433, vo_c3_Locus_2347_Transcript_16/16_Confidence_0.270_Length_1737, vo_c2_Locus_298_Transcript_21/24_Confidence_0.368_Length_1678, vo_c3_Locus_3927_Transcript_2/9_Confidence_0.250_Length_2629, vo_c2_Locus_3634_Transcript_8/8_Confidence_0.133_Length_2428, vo_c2_Locus_2437_Transcript_14/25_Confidence_0.455_Length_1511, vo_c1_Locus_3033_Transcript_3/5_Confidence_0.400_Length_2348, vo_c3_Locus_239_Transcript_29/29_Confidence_0.182_Length_2208, vo_n3_Locus_2623_Transcript_15/17_Confidence_0.361_Length_1551, vo_c3_Locus_2347_Transcript_5/16_Confidence_0.514_Length_1554, vo_c3_Locus_239_Transcript_19/29_Confidence_0.295_Length_1502, vo_c2_Locus_2437_Transcript_25/25_Confidence_0.159_Length_1517, vo_n2_Locus_2252_Transcript_22/26_Confidence_0.256_Length_2337, vo_n2_Locus_2252_Transcript_23/26_Confidence_0.359_Length_1959, vo_c3_Locus_239_Transcript_10/29_Confidence_0.386_Length_1727, vo_c1_Locus_788_Transcript_6/34_Confidence_0.362_Length_1766, vo_c1_Locus_788_Transcript_22/34_Confidence_0.277_Length_1358, vo_n1_Locus_2248_Transcript_26/40_Confidence_0.333_Length_1991, tri_c_comp20571_c0_seq1, vo_n2_Locus_2252_Transcript_18/26_Confidence_0.256_Length_1506, vo_c1_Locus_788_Transcript_11/34_Confidence_0.213_Length_2514, vo_c2_Locus_907_Transcript_3/3_Confidence_0.333_Length_630, vo_n2_Locus_7087_Transcript_1/2_Confidence_0.500_Length_4087, vo_n1_Locus_2248_Transcript_2/40_Confidence_0.373_Length_1784, vo_n2_Locus_2980_Transcript_1/1_Confidence_0.333_Length_1623, vo_c3_Locus_550_Transcript_25/30_Confidence_0.321_Length_1640, vo_c3_Locus_550_Transcript_10/30_Confidence_0.420_Length_1515, vo_n1_Locus_133_Transcript_12/30_Confidence_0.033_Length_580, vo_c2_Locus_320_Transcript_7/32_Confidence_0.299_Length_1642, vo_c3_Locus_550_Transcript_27/30_Confidence_0.395_Length_1758, vo_c2_Locus_320_Transcript_9/32_Confidence_0.338_Length_1673, vo_c2_Locus_320_Transcript_22/32_Confidence_0.545_Length_1648, vo_c3_Locus_550_Transcript_30/30_Confidence_0.210_Length_1413, vo_c3_Locus_550_Transcript_8/30_Confidence_0.148_Length_1492, vo_n3_Locus_587_Transcript_13/22_Confidence_0.444_Length_1456, vo_n2_Locus_105_Transcript_30/33_Confidence_0.449_Length_1990, vo_c3_Locus_550_Transcript_28/30_Confidence_0.309_Length_1831, vo_c2_Locus_320_Transcript_20/32_Confidence_0.325_Length_1586, vo_n1_Locus_133_Transcript_30/30_Confidence_0.033_Length_2622, vo_c2_Locus_320_Transcript_13/32_Confidence_0.351_Length_2604, vo_c2_Locus_320_Transcript_15/32_Confidence_0.390_Length_1557, vo_c2_Locus_500_Transcript_3/29_Confidence_0.344_Length_1522, vo_c3_Locus_527_Transcript_60/87_Confidence_0.092_Length_2707, vo_n2_Locus_560_Transcript_46/53_Confidence_0.118_Length_1515, vo_n2_Locus_560_Transcript_41/53_Confidence_0.300_Length_1572, vo_c2_Locus_500_Transcript_4/29_Confidence_0.377_Length_1516, vo_c2_Locus_500_Transcript_13/29_Confidence_0.328_Length_1409, vo_c2_Locus_500_Transcript_15/29_Confidence_0.393_Length_1699, vo_n3_Locus_874_Transcript_21/35_Confidence_0.236_Length_1484, vo_c2_Locus_500_Transcript_28/29_Confidence_0.279_Length_1509, vo_n3_Locus_874_Transcript_32/35_Confidence_0.270_Length_1521, vo_n1_Locus_1829_Transcript_21/31_Confidence_0.519_Length_1458, vo_n3_Locus_874_Transcript_27/35_Confidence_0.303_Length_1523, vo_n2_Locus_560_Transcript_47/53_Confidence_0.173_Length_1554, vo_n3_Locus_874_Transcript_28/35_Confidence_0.213_Length_1767, vo_c1_Locus_962_Transcript_32/36_Confidence_0.383_Length_1689, vo_n2_Locus_560_Transcript_43/53_Confidence_0.273_Length_2234, vo_c3_Locus_527_Transcript_66/87_Confidence_0.220_Length_3076, vo_c2_Locus_500_Transcript_2/29_Confidence_0.328_Length_1849, vo_n1_Locus_1829_Transcript_31/31_Confidence_0.269_Length_1123, vo_n2_Locus_560_Transcript_50/53_Confidence_0.227_Length_1564, vo_c1_Locus_962_Transcript_33/36_Confidence_0.383_Length_1437, vo_c1_Locus_962_Transcript_29/36_Confidence_0.417_Length_1683, vo_c2_Locus_500_Transcript_27/29_Confidence_0.262_Length_1458, vo_n3_Locus_874_Transcript_8/35_Confidence_0.315_Length_1484, vo_n2_Locus_560_Transcript_36/53_Confidence_0.245_Length_1639, vo_c3_Locus_550_Transcript_25/30_Confidence_0.321_Length_1640, vo_c3_Locus_550_Transcript_10/30_Confidence_0.420_Length_1515, vo_n1_Locus_133_Transcript_12/30_Confidence_0.033_Length_580, vo_n2_Locus_13049_Transcript_20/20_Confidence_0.289_Length_1233, vo_c3_Locus_1015_Transcript_30/32_Confidence_0.181_Length_1172, vo_n2_Locus_13049_Transcript_19/20_Confidence_0.368_Length_1268, vo_c3_Locus_550_Transcript_27/30_Confidence_0.395_Length_1758, vo_n1_Locus_296_Transcript_24/31_Confidence_0.275_Length_1182, vo_n2_Locus_13049_Transcript_17/20_Confidence_0.342_Length_1329, vo_c3_Locus_1015_Transcript_13/32_Confidence_0.222_Length_1363, vo_c2_Locus_1551_Transcript_19/27_Confidence_0.222_Length_1248, vo_n1_Locus_296_Transcript_27/31_Confidence_0.078_Length_2886, vo_c3_Locus_1015_Transcript_3/32_Confidence_0.181_Length_1206, vo_n2_Locus_105_Transcript_30/33_Confidence_0.449_Length_1990, vo_c3_Locus_550_Transcript_28/30_Confidence_0.309_Length_1831, vo_n3_Locus_150_Transcript_17/25_Confidence_0.183_Length_1185, vo_n3_Locus_150_Transcript_6/25_Confidence_0.232_Length_1192, vo_n1_Locus_133_Transcript_30/30_Confidence_0.033_Length_2622, tri_c_comp21115_c0_seq1, vo_n3_Locus_587_Transcript_2/22_Confidence_0.111_Length_206, vo_c2_Locus_320_Transcript_13/32_Confidence_0.351_Length_2604, vo_n1_Locus_296_Transcript_25/31_Confidence_0.157_Length_1180, vo_n2_Locus_13049_Transcript_7/20_Confidence_0.421_Length_1354, vo_c1_Locus_223_Transcript_13/65_Confidence_0.197_Length_1636, vo_c1_Locus_223_Transcript_47/65_Confidence_0.099_Length_1259, vo_c2_Locus_320_Transcript_7/32_Confidence_0.299_Length_1642, vo_n1_Locus_133_Transcript_14/30_Confidence_0.456_Length_2738,

					vo_c3_Locus_1015_Transcript_12/32_Confidence_0.222_Length_1270, vo_c2_Locus_320_Transcript_9/32_Confidence_0.338_Length_1673, vo_c2_Locus_320_Transcript_22/32_Confidence_0.545_Length_1648, vo_n1_Locus_296_Transcript_23/31_Confidence_0.294_Length_1182, vo_n2_Locus_13049_Transcript_9/20_Confidence_0.316_Length_1224, vo_c1_Locus_223_Transcript_52/65_Confidence_0.042_Length_799, vo_c3_Locus_550_Transcript_30/30_Confidence_0.210_Length_1413, vo_n1_Locus_133_Transcript_13/30_Confidence_0.456_Length_2508, vo_n1_Locus_133_Transcript_24/30_Confidence_0.311_Length_1984, vo_n1_Locus_296_Transcript_30/31_Confidence_0.078_Length_1319, vo_n1_Locus_133_Transcript_15/30_Confidence_0.456_Length_2671, vo_c2_Locus_320_Transcript_20/32_Confidence_0.325_Length_1586, vo_c1_Locus_223_Transcript_19/65_Confidence_0.092_Length_1454, vo_n1_Locus_296_Transcript_10/31_Confidence_0.118_Length_1164, vo_c1_Locus_223_Transcript_54/65_Confidence_0.134_Length_1458, vo_c2_Locus_320_Transcript_15/32_Confidence_0.390_Length_1557, vo_c3_Locus_1015_Transcript_9/32_Confidence_0.236_Length_1272, vo_n1_Locus_133_Transcript_25/30_Confidence_0.278_Length_2179, vo_c2_Locus_3634_Transcript_8/8_Confidence_0.133_Length_2428, vo_c2_Locus_324_Transcript_5/12_Confidence_0.333_Length_1009, vo_n1_Locus_5721_Transcript_6/9_Confidence_0.389_Length_989, vo_c2_Locus_324_Transcript_11/12_Confidence_0.333_Length_4393, vo_n2_Locus_475_Transcript_7/13_Confidence_0.312_Length_1141, vo_n1_Locus_5721_Transcript_1/9_Confidence_0.278_Length_986, vo_n2_Locus_475_Transcript_2/13_Confidence_0.312_Length_1187, vo_c2_Locus_324_Transcript_2/12_Confidence_0.400_Length_1106, vo_c3_Locus_3927_Transcript_2/9_Confidence_0.250_Length_2629, vo_c1_Locus_34898_Transcript_1/1_Confidence_0.000_Length_1083, vo_n2_Locus_8097_Transcript_1/6_Confidence_0.500_Length_1196, vo_n1_Locus_3423_Transcript_2/4_Confidence_0.250_Length_1347, tri_n_comp27683_c0_seq1, vo_c1_Locus_3127_Transcript_1/1_Confidence_0.000_Length_1478, vo_n1_Locus_3423_Transcript_3/4_Confidence_0.250_Length_1416, vo_n1_Locus_565_Transcript_31/31_Confidence_0.178_Length_3922, vo_c3_Locus_2800_Transcript_3/23_Confidence_0.566_Length_1690, vo_c3_Locus_2800_Transcript_9/23_Confidence_0.585_Length_1396, tri_n_comp21653_c0_seq1, vo_n1_Locus_565_Transcript_5/31_Confidence_0.244_Length_1533, vo_n2_Locus_4661_Transcript_5/12_Confidence_0.208_Length_1240, vo_n1_Locus_565_Transcript_17/31_Confidence_0.422_Length_1454, vo_c3_Locus_2800_Transcript_5/23_Confidence_0.566_Length_2133, vo_c1_Locus_1757_Transcript_17/19_Confidence_0.375_Length_1367, vo_c1_Locus_298_Transcript_4/32_Confidence_0.255_Length_1585, vo_n1_Locus_8402_Transcript_3/4_Confidence_0.286_Length_1464, vo_c3_Locus_2800_Transcript_23/23_Confidence_0.151_Length_1466, vo_n2_Locus_903_Transcript_38/40_Confidence_0.222_Length_6612, vo_n1_Locus_565_Transcript_20/31_Confidence_0.444_Length_1622, vo_n2_Locus_2046_Transcript_7/18_Confidence_0.345_Length_1296, vo_c3_Locus_2800_Transcript_8/23_Confidence_0.585_Length_1344, vo_n3_Locus_1119_Transcript_19/50_Confidence_0.050_Length_498, vo_c2_Locus_5742_Transcript_10/21_Confidence_0.244_Length_1454, vo_n3_Locus_1119_Transcript_11/50_Confidence_0.124_Length_2839, vo_c2_Locus_5742_Transcript_18/21_Confidence_0.317_Length_2233, vo_n1_Locus_565_Transcript_13/31_Confidence_0.378_Length_1400, vo_c3_Locus_2800_Transcript_14/23_Confidence_0.321_Length_1426, vo_c3_Locus_614_Transcript_19/24_Confidence_0.394_Length_3443, vo_c2_Locus_358_Transcript_1/41_Confidence_0.080_Length_1708, vo_c2_Locus_5742_Transcript_14/21_Confidence_0.439_Length_2413, vo_c1_Locus_1757_Transcript_8/19_Confidence_0.458_Length_1460, vo_n1_Locus_986_Transcript_16/36_Confidence_0.211_Length_1811, vo_n1_Locus_565_Transcript_16/31_Confidence_0.400_Length_1499, vo_n2_Locus_903_Transcript_37/40_Confidence_0.346_Length_3926, tri_c_comp20312_c0_seq4, vo_c3_Locus_614_Transcript_13/24_Confidence_0.061_Length_4970, vo_n2_Locus_2046_Transcript_11/18_Confidence_0.310_Length_1666, vo_n2_Locus_903_Transcript_31/40_Confidence_0.321_Length_2251, vo_n1_Locus_565_Transcript_30/31_Confidence_0.422_Length_3814, vo_c3_Locus_614_Transcript_2/24_Confidence_0.485_Length_1777, vo_n1_Locus_5148_Transcript_1/4_Confidence_0.667_Length_1983, vo_c1_Locus_10454_Transcript_1/2_Confidence_0.750_Length_1441, vo_n3_Locus_1119_Transcript_18/50_Confidence_0.149_Length_1959, vo_c3_Locus_6221_Transcript_1/1_Confidence_0.000_Length_1565, vo_c2_Locus_6460_Transcript_1/2_Confidence_1.000_Length_1563, vo_n2_Locus_3068_Transcript_1/3_Confidence_0.250_Length_1485, vo_n3_Locus_1573_Transcript_4/9_Confidence_0.364_Length_1074, vo_n2_Locus_3298_Transcript_6/11_Confidence_0.333_Length_1072, vo_n1_Locus_7783_Transcript_1/2_Confidence_0.667_Length_900, vo_c2_Locus_40594_Transcript_2/3_Confidence_0.500_Length_1728, vo_n2_Locus_39295_Transcript_2/2_Confidence_0.500_Length_1939, vo_c2_Locus_6460_Transcript_1/2_Confidence_1.000_Length_1563, vo_n1_Locus_3261_Transcript_3/4_Confidence_0.667_Length_1633, vo_n3_Locus_27_Transcript_1/1_Confidence_0.000_Length_1278, tri_c_comp23875_c0_seq1, tri_c_comp24147_c0_seq1, vo_c1_Locus_4576_Transcript_2/2_Confidence_0.000_Length_1644, vo_c3_Locus_1723_Transcript_29/37_Confidence_0.411_Length_1695, vo_n2_Locus_330_Transcript_25/38_Confidence_0.361_Length_1516, vo_c3_Locus_1723_Transcript_35/37_Confidence_0.464_Length_1702, vo_c1_Locus_7371_Transcript_4/4_Confidence_0.600_Length_1352, vo_c3_Locus_1723_Transcript_37/37_Confidence_0.214_Length_1530, vo_n1_Locus_769_Transcript_28/41_Confidence_0.400_Length_1609, vo_n1_Locus_769_Transcript_9/41_Confidence_0.460_Length_1494, vo_c3_Locus_2965_Transcript_2/2_Confidence_0.250_Length_1327, vo_n2_Locus_330_Transcript_5/38_Confidence_0.328_Length_11715, vo_n1_Locus_9772_Transcript_1/2_Confidence_0.333_Length_1224, vo_c1_Locus_3603_Transcript_44/48_Confidence_0.492_Length_1696, vo_c2_Locus_4242_Transcript_2/3_Confidence_0.400_Length_1168, vo_n1_Locus_6649_Transcript_2/3_Confidence_0.500_Length_2124, vo_c1_Locus_7371_Transcript_1/4_Confidence_0.500_Length_1352, vo_n3_Locus_2381_Transcript_2/3_Confidence_0.500_Length_1330, vo_c3_Locus_1723_Transcript_16/37_Confidence_0.286_Length_1491, vo_n1_Locus_769_Transcript_36/41_Confidence_0.420_Length_1536, vo_n1_Locus_769_Transcript_40/41_Confidence_0.320_Length_1479, vo_n1_Locus_6649_Transcript_1/3_Confidence_0.750_Length_1883, vo_c1_Locus_3603_Transcript_26/48_Confidence_0.400_Length_1432, vo_n1_Locus_5348_Transcript_2/2_Confidence_0.200_Length_1608, vo_n3_Locus_3383_Transcript_35/37_Confidence_0.389_Length_1512, tri_c_comp20802_c0_seq1, vo_c3_Locus_1723_Transcript_29/37_Confidence_0.411_Length_1695, vo_n2_Locus_330_Transcript_25/38_Confidence_0.361_Length_1516, vo_c3_Locus_1723_Transcript_35/37_Confidence_0.464_Length_1702, vo_c1_Locus_15206_Transcript_3/3_Confidence_0.000_Length_1312, vo_c1_Locus_7371_Transcript_4/4_Confidence_0.600_Length_1352,

					vo_c3_Locus_1723_Transcript_37/37_Confidence_0.214_Length_1530, vo_n1_Locus_769_Transcript_28/41_Confidence_0.400_Length_1609, vo_n1_Locus_769_Transcript_9/41_Confidence_0.460_Length_1494, vo_c3_Locus_2965_Transcript_2/2_Confidence_0.250_Length_1327, vo_n2_Locus_330_Transcript_5/38_Confidence_0.328_Length_11715, vo_n1_Locus_9772_Transcript_1/2_Confidence_0.333_Length_1224, vo_c1_Locus_3603_Transcript_44/48_Confidence_0.492_Length_1696, vo_c2_Locus_4242_Transcript_2/3_Confidence_0.400_Length_1168, vo_n1_Locus_6649_Transcript_2/3_Confidence_0.500_Length_2124, vo_c1_Locus_7371_Transcript_1/4_Confidence_0.500_Length_1352, vo_n3_Locus_2381_Transcript_2/3_Confidence_0.500_Length_1330, vo_c3_Locus_1723_Transcript_16/37_Confidence_0.286_Length_1491, vo_n1_Locus_769_Transcript_36/41_Confidence_0.420_Length_1536, vo_n1_Locus_769_Transcript_40/41_Confidence_0.320_Length_1479, vo_n1_Locus_6649_Transcript_1/3_Confidence_0.750_Length_1883, vo_c1_Locus_3603_Transcript_26/48_Confidence_0.400_Length_1432, vo_n1_Locus_5348_Transcript_2/2_Confidence_0.200_Length_1608, vo_n3_Locus_3383_Transcript_35/37_Confidence_0.389_Length_1512, tri_c_comp20802_c0_seq1, vo_c1_Locus_8302_Transcript_51/55_Confidence_0.100_Length_500, vo_n2_Locus_2969_Transcript_26/36_Confidence_0.373_Length_1726, vo_n3_Locus_8916_Transcript_29/29_Confidence_0.191_Length_2143, vo_c1_Locus_8302_Transcript_52/55_Confidence_0.100_Length_554, vo_c1_Locus_8302_Transcript_26/55_Confidence_0.129_Length_1144, vo_n3_Locus_8916_Transcript_11/29_Confidence_0.277_Length_861, vo_n2_Locus_2969_Transcript_30/36_Confidence_0.441_Length_2103, vo_c1_Locus_8302_Transcript_55/55_Confidence_0.043_Length_1090, vo_c3_Locus_20712_Transcript_20/28_Confidence_0.550_Length_2132, tri_c_comp20385_c0_seq1, vo_c1_Locus_8302_Transcript_54/55_Confidence_0.114_Length_1036, vo_n3_Locus_8916_Transcript_29/29_Confidence_0.191_Length_2143, vo_c1_Locus_8302_Transcript_26/55_Confidence_0.129_Length_1144, vo_n3_Locus_8916_Transcript_11/29_Confidence_0.277_Length_861, vo_n2_Locus_2969_Transcript_30/36_Confidence_0.441_Length_2103, vo_c3_Locus_20712_Transcript_20/28_Confidence_0.550_Length_2132, tri_c_comp20385_c0_seq1, tri_c_comp20385_c0_seq4

Porphyrin and chlorophyll metabolism	map00860	17	ec:1.2.1.70 - reductase, ec:4.1.1.37 - decarboxylase, ec:1.3.1.76 - dehydrogenase, ec:4.99.1.1 - ferrochelatase, ec:2.1.1.11 - protoporphyrin IX methyltransferase, ec:1.3.1.33 - reductase, ec:5.4.3.8 - 2,1-aminomutase, ec:2.4.1.17 - 1-naphthol glucuronyltransferase, ec:1.3.3.4 - oxidase, ec:1.3.3.3 - oxidase, ec:6.6.1.1 - chelatase, ec:4.4.1.17 - synthase, ec:1.1.1.294 - b reductase, ec:4.2.1.24 - synthase, ec:2.5.1.61 - synthase, ec:1.14.13.122 - oxygenase, ec:6.1.1.17 - ligase	1, 1, 1, 7, 2, 1, 11, 13, 1, 4, 11, 2, 1, 1, 2, 3, 5	vo_c2_Locus_6351_Transcript_1/1_Confidence_0.000_Length_2446, vo_c2_Locus_14123_Transcript_1/1_Confidence_0.000_Length_2014, vo_c1_Locus_10296_Transcript_1/2_Confidence_0.333_Length_535, vo_c1_Locus_19524_Transcript_9/12_Confidence_0.615_Length_3975, vo_c1_Locus_9488_Transcript_3/3_Confidence_0.143_Length_1651, vo_c1_Locus_19524_Transcript_11/12_Confidence_0.231_Length_3997, vo_c2_Locus_859_Transcript_3/4_Confidence_0.125_Length_2424, tri_n_comp33679_c0_seq1, vo_n1_Locus_16962_Transcript_2/2_Confidence_0.000_Length_1641, vo_c2_Locus_9907_Transcript_3/4_Confidence_0.300_Length_2286, vo_n2_Locus_15987_Transcript_6/14_Confidence_0.633_Length_2476, vo_n1_Locus_18944_Transcript_5/7_Confidence_0.308_Length_1755, vo_n2_Locus_2037_Transcript_3/9_Confidence_0.545_Length_1901, vo_c3_Locus_2906_Transcript_6/11_Confidence_0.600_Length_1624, vo_n2_Locus_3814_Transcript_11/17_Confidence_0.455_Length_1871, vo_c2_Locus_1591_Transcript_4/11_Confidence_0.529_Length_1593, vo_c1_Locus_4196_Transcript_6/13_Confidence_0.333_Length_1733, vo_n2_Locus_3814_Transcript_8/17_Confidence_0.485_Length_1854, tri_n_comp11497_c0_seq1, vo_c1_Locus_4196_Transcript_11/13_Confidence_0.167_Length_3524, vo_c3_Locus_2906_Transcript_3/11_Confidence_0.600_Length_1609, vo_n2_Locus_3814_Transcript_12/17_Confidence_0.424_Length_3305, vo_c2_Locus_1591_Transcript_3/11_Confidence_0.588_Length_1821, vo_c2_Locus_1591_Transcript_11/11_Confidence_0.176_Length_1763, tri_c_comp6209_c0_seq1, vo_c1_Locus_26340_Transcript_5/9_Confidence_0.600_Length_1699, tri_n_comp65990_c0_seq1, vo_n1_Locus_13372_Transcript_1/2_Confidence_0.333_Length_1687, vo_n2_Locus_34154_Transcript_3/3_Confidence_0.000_Length_2043, vo_n2_Locus_668_Transcript_17/17_Confidence_0.070_Length_2403, tri_c_comp58135_c0_seq1, vo_c3_Locus_16093_Transcript_1/1_Confidence_0.000_Length_1750, vo_n1_Locus_13372_Transcript_2/2_Confidence_0.000_Length_1314, vo_c2_Locus_24841_Transcript_3/3_Confidence_0.167_Length_3241, vo_n2_Locus_4774_Transcript_1/1_Confidence_0.000_Length_884, vo_c1_Locus_35615_Transcript_1/1_Confidence_0.000_Length_2075, vo_n1_Locus_15639_Transcript_1/1_Confidence_0.000_Length_1086, vo_c1_Locus_11209_Transcript_1/1_Confidence_0.000_Length_1909, vo_c1_Locus_31905_Transcript_2/2_Confidence_0.000_Length_1664, vo_n2_Locus_30478_Transcript_3/5_Confidence_0.455_Length_1623, tri_n_comp21857_c0_seq1, vo_n3_Locus_12576_Transcript_2/2_Confidence_0.250_Length_1541, tri_c_comp22421_c0_seq1, vo_n1_Locus_5081_Transcript_2/2_Confidence_0.000_Length_1015, tri_n_comp19825_c0_seq2, vo_c1_Locus_2134_Transcript_19/32_Confidence_0.509_Length_4654, vo_c2_Locus_1956_Transcript_2/3_Confidence_0.200_Length_861, vo_n1_Locus_5020_Transcript_1/33_Confidence_0.018_Length_238, tri_c_comp59880_c0_seq1, vo_c3_Locus_17678_Transcript_1/2_Confidence_0.667_Length_1546, vo_n3_Locus_3787_Transcript_3/6_Confidence_0.462_Length_2514, vo_c1_Locus_21235_Transcript_2/4_Confidence_0.625_Length_1588, vo_c1_Locus_2134_Transcript_1/32_Confidence_0.019_Length_215, vo_n2_Locus_3_Transcript_126/24291_Confidence_0.000_Length_867, vo_n2_Locus_3_Transcript_123/24291_Confidence_0.000_Length_923, vo_c3_Locus_371_Transcript_5/5_Confidence_0.000_Length_569, vo_c3_Locus_14534_Transcript_1/1_Confidence_0.000_Length_1548, vo_n2_Locus_5339_Transcript_1/2_Confidence_0.800_Length_1469, tri_c_comp65020_c0_seq1, tri_c_comp23477_c0_seq1, vo_c2_Locus_19685_Transcript_3/3_Confidence_0.000_Length_1955, vo_n1_Locus_28213_Transcript_1/1_Confidence_0.000_Length_2019, tri_n_comp17763_c0_seq2, tri_n_comp33248_c0_seq1, vo_n1_Locus_24951_Transcript_1/2_Confidence_0.750_Length_1980, vo_n1_Locus_24951_Transcript_2/2_Confidence_0.000_Length_2025, vo_n3_Locus_8238_Transcript_3/4_Confidence_0.750_Length_2102

Terpenoid backbone biosynthesis	map00900	17	ec:2.7.4.2 - kinase, ec:1.17.7.1 - synthase (ferredoxin), ec:2.2.1.7 - synthase, ec:2.5.1.10 - diphosphate synthase, ec:2.5.1.29 - diphosphate synthase, ec:2.7.1.148 - 5'-diphospho)-2-C-methyl-D-erythritol kinase, ec:2.5.1.1 - geranyl-diphosphate synthase, ec:4.6.1.12 - 2,4-cyclodiphosphate synthase, ec:2.7.7.60 - 4-phosphate cytidylyltransferase, ec:5.3.3.2 - Delta-isomerase, ec:2.3.1.9 - C-acetyltransferase, ec:2.3.3.10 - synthase, ec:1.1.1.267 - reductoisomerase, ec:2.5.1.85 - diphosphate synthase [geranylgeranyl-diphosphate specific], ec:2.5.1.84 - diphosphate synthase [geranyl-diphosphate specific], ec:2.1.1.100 - O-methyltransferase, ec:2.5.1.58 - farnesyltransferase	1, 2, 1, 1, 1, 1, 4, 1, 1, 1, 2, 4, 1, 3, 3, 2, 1	vo_n2_Locus_13158_Transcript_1/2_Confidence_1.000_Length_1003, tri_n_comp20539_c0_seq1, vo_n2_Locus_2098_Transcript_8/8_Confidence_0.130_Length_2485, vo_c1_Locus_12331_Transcript_1/1_Confidence_0.000_Length_2548, tri_c_comp29205_c0_seq1, tri_c_comp29205_c0_seq1, vo_c2_Locus_14475_Transcript_1/1_Confidence_0.000_Length_1444, tri_c_comp29205_c0_seq1, vo_n2_Locus_26426_Transcript_1/1_Confidence_0.000_Length_3133, tri_c_comp37590_c0_seq1, vo_n2_Locus_15100_Transcript_1/9_Confidence_0.417_Length_1002, tri_n_comp46466_c0_seq1, vo_c3_Locus_22219_Transcript_1/1_Confidence_0.000_Length_1123, tri_c_comp43842_c0_seq1, vo_c1_Locus_2657_Transcript_1/1_Confidence_0.000_Length_1513, vo_n2_Locus_23058_Transcript_1/1_Confidence_0.000_Length_1342, vo_n2_Locus_634_Transcript_4/6_Confidence_0.357_Length_8557, tri_c_comp57035_c0_seq1, vo_c1_Locus_1809_Transcript_1/1_Confidence_0.000_Length_3087, vo_c3_Locus_18718_Transcript_1/1_Confidence_0.000_Length_2686, vo_n3_Locus_12427_Transcript_1/2_Confidence_1.000_Length_1739, vo_n2_Locus_15100_Transcript_1/9_Confidence_0.417_Length_1002, vo_n2_Locus_15100_Transcript_8/9_Confidence_0.500_Length_2909, vo_n2_Locus_26426_Transcript_1/1_Confidence_0.000_Length_3133, vo_n2_Locus_15100_Transcript_1/9_Confidence_0.417_Length_1002, vo_n2_Locus_15100_Transcript_8/9_Confidence_0.500_Length_2909, vo_n2_Locus_26426_Transcript_1/1_Confidence_0.000_Length_3133, vo_n3_Locus_17871_Transcript_5/5_Confidence_0.417_Length_1328, vo_c2_Locus_15058_Transcript_3/14_Confidence_0.343_Length_2024, vo_c1_Locus_13009_Transcript_1/1_Confidence_0.000_Length_1250

Glyoxylate and dicarboxylate metabolism	map00630	16	ec:4.1.1.39 - carboxylase, ec:6.3.1.2 - synthetase, ec:4.1.3.24 - lyase, ec:6.2.1.8 - ligase, ec:1.4.7.1 - synthase (ferredoxin), ec:2.3.1.9 - C-acetyltransferase, ec:1.2.3.4 - oxidase, ec:2.3.3.1 - (Si)-synthase, ec:3.5.1.9 - kynurenine formamidase, ec:2.1.2.1 - hydroxymethyltransferase, ec:4.2.1.3 - hydratase, ec:4.1.3.1 - lyase, ec:3.1.3.18 - phosphatase, ec:1.1.3.15 - oxidase, ec:1.1.1.37 - dehydrogenase, ec:1.1.1.36 - reductase	95, 15, 15, 1, 12, 2, 1, 11, 2, 9, 8, 30, 4, 11, 24, 1	vo_c1_Locus_1636_Transcript_17/122_Confidence_0.157_Length_2013, vo_n3_Locus_37_Transcript_16/46_Confidence_0.281_Length_1685, vo_n2_Locus_3_Transcript_22527/24291_Confidence_0.000_Length_1609, vo_n3_Locus_37_Transcript_10/46_Confidence_0.207_Length_1727, vo_n3_Locus_37_Transcript_9/46_Confidence_0.215_Length_1812, vo_c1_Locus_1636_Transcript_122/122_Confidence_0.108_Length_2335, vo_n2_Locus_3_Transcript_22541/24291_Confidence_0.000_Length_288, vo_n2_Locus_3_Transcript_22550/24291_Confidence_0.000_Length_1791, vo_n3_Locus_37_Transcript_33/46_Confidence_0.163_Length_732, vo_n2_Locus_3_Transcript_22526/24291_Confidence_0.000_Length_620, vo_n2_Locus_3_Transcript_22511/24291_Confidence_0.000_Length_798, vo_c1_Locus_1636_Transcript_15/122_Confidence_0.065_Length_512, vo_c2_Locus_52_Transcript_144/466_Confidence_0.025_Length_2131, tri_n_comp20144_c0_seq14, vo_c2_Locus_52_Transcript_133/466_Confidence_0.023_Length_2364, tri_n_comp20144_c0_seq13, vo_n3_Locus_41731_Transcript_1/1_Confidence_0.500_Length_459, vo_c2_Locus_52_Transcript_132/466_Confidence_0.016_Length_1024, vo_n1_Locus_55_Transcript_52/66_Confidence_0.069_Length_313, tri_c_comp20343_c0_seq9, tri_n_comp20144_c0_seq10, tri_n_comp20144_c0_seq7, tri_n_comp20144_c0_seq6, vo_c3_Locus_31_Transcript_6/79_Confidence_0.152_Length_709, tri_n_comp20144_c0_seq3, tri_n_comp20144_c0_seq4, tri_n_comp20144_c0_seq1, vo_c1_Locus_1636_Transcript_86/122_Confidence_0.182_Length_2244, vo_c1_Locus_1636_Transcript_73/122_Confidence_0.222_Length_1747, vo_c2_Locus_52_Transcript_164/466_Confidence_0.011_Length_2810, vo_n2_Locus_3_Transcript_22535/24291_Confidence_0.000_Length_656, vo_n2_Locus_3_Transcript_22546/24291_Confidence_0.000_Length_1498, vo_c3_Locus_31_Transcript_74/79_Confidence_0.124_Length_2769, vo_c3_Locus_31_Transcript_68/79_Confidence_0.124_Length_1224, vo_n3_Locus_37_Transcript_21/46_Confidence_0.148_Length_1139, vo_c3_Locus_31_Transcript_67/79_Confidence_0.152_Length_2229, vo_n3_Locus_37_Transcript_14/46_Confidence_0.259_Length_5942, vo_n1_Locus_55_Transcript_3/66_Confidence_0.063_Length_450, tri_c_comp20343_c0_seq4, tri_c_comp20343_c0_seq3, vo_n2_Locus_3_Transcript_22566/24291_Confidence_0.000_Length_2168, vo_n2_Locus_3452_Transcript_2/4_Confidence_0.636_Length_263, vo_n2_Locus_3_Transcript_22486/24291_Confidence_0.000_Length_1883, vo_c1_Locus_1636_Transcript_120/122_Confidence_0.135_Length_3625, vo_c2_Locus_52_Transcript_120/466_Confidence_0.009_Length_610, vo_c3_Locus_31_Transcript_13/79_Confidence_0.099_Length_748, vo_c1_Locus_1636_Transcript_69/122_Confidence_0.074_Length_844, vo_n2_Locus_3_Transcript_22520/24291_Confidence_0.000_Length_1452, vo_n2_Locus_3_Transcript_22533/24291_Confidence_0.000_Length_679, vo_n2_Locus_3_Transcript_22537/24291_Confidence_0.000_Length_840, vo_c3_Locus_31_Transcript_63/79_Confidence_0.148_Length_3613, vo_c1_Locus_1636_Transcript_71/122_Confidence_0.055_Length_846, vo_c3_Locus_31_Transcript_77/79_Confidence_0.297_Length_1736, vo_c1_Locus_1636_Transcript_83/122_Confidence_0.191_Length_2430, tri_n_comp20833_c0_seq3, vo_c3_Locus_31_Transcript_75/79_Confidence_0.212_Length_8463, vo_c2_Locus_52_Transcript_153/466_Confidence_0.026_Length_1551, vo_c3_Locus_31_Transcript_3/79_Confidence_0.035_Length_301, vo_n2_Locus_3_Transcript_22489/24291_Confidence_0.000_Length_274, vo_n2_Locus_3_Transcript_22509/24291_Confidence_0.000_Length_800, vo_n3_Locus_37_Transcript_38/46_Confidence_0.163_Length_922, tri_c_comp21288_c0_seq1, vo_c1_Locus_1636_Transcript_88/122_Confidence_0.074_Length_1384, vo_c1_Locus_1636_Transcript_121/122_Confidence_0.018_Length_960, vo_n3_Locus_37_Transcript_5/46_Confidence_0.289_Length_1505, vo_n1_Locus_55_Transcript_33/66_Confidence_0.040_Length_1095, vo_c3_Locus_31_Transcript_65/79_Confidence_0.166_Length_2369, vo_c3_Locus_31_Transcript_73/79_Confidence_0.208_Length_2557, vo_c3_Locus_31_Transcript_9/79_Confidence_0.152_Length_714, vo_c2_Locus_52_Transcript_113/466_Confidence_0.004_Length_590, vo_n2_Locus_3_Transcript_22492/24291_Confidence_0.000_Length_1595, vo_n1_Locus_55_Transcript_54/66_Confidence_0.183_Length_2185, vo_c3_Locus_33_Transcript_2/2_Confidence_0.714_Length_611, vo_c2_Locus_52_Transcript_125/466_Confidence_0.007_Length_559, vo_c2_Locus_52_Transcript_135/466_Confidence_0.025_Length_2711, vo_n1_Locus_55_Transcript_12/66_Confidence_0.331_Length_2232, vo_c1_Locus_1636_Transcript_100/122_Confidence_0.031_Length_685, vo_c2_Locus_52_Transcript_131/466_Confidence_0.023_Length_1964, vo_c3_Locus_31_Transcript_7/79_Confidence_0.184_Length_710, vo_n1_Locus_55_Transcript_39/66_Confidence_0.194_Length_1391, vo_n3_Locus_37_Transcript_28/46_Confidence_0.193_Length_1043, vo_n1_Locus_55_Transcript_41/66_Confidence_0.263_Length_1742, vo_n1_Locus_55_Transcript_38/66_Confidence_0.171_Length_1387, vo_n2_Locus_3_Transcript_22491/24291_Confidence_0.000_Length_1174, vo_n3_Locus_37_Transcript_30/46_Confidence_0.178_Length_1043, vo_n2_Locus_3_Transcript_22503/24291_Confidence_0.000_Length_721, vo_n2_Locus_3_Transcript_22540/24291_Confidence_0.000_Length_271, vo_c2_Locus_52_Transcript_128/466_Confidence_0.003_Length_564, vo_c2_Locus_52_Transcript_162/466_Confidence_0.019_Length_1668, vo_n2_Locus_3_Transcript_22534/24291_Confidence_0.000_Length_449, vo_c1_Locus_1636_Transcript_116/122_Confidence_0.129_Length_3526, vo_c3_Locus_31_Transcript_70/79_Confidence_0.208_Length_1586, vo_n1_Locus_55_Transcript_9/66_Confidence_0.349_Length_2172, vo_c1_Locus_8362_Transcript_4/9_Confidence_0.254_Length_482, vo_n1_Locus_55_Transcript_50/66_Confidence_0.166_Length_2379, vo_c1_Locus_1340_Transcript_12/17_Confidence_0.109_Length_2389, vo_c1_Locus_1340_Transcript_9/17_Confidence_0.172_Length_2429, tri_n_comp11684_c0_seq1, vo_c1_Locus_4136_Transcript_1/2_Confidence_0.967_Length_2259, vo_c2_Locus_6408_Transcript_2/14_Confidence_0.268_Length_1478, vo_n1_Locus_335_Transcript_2/3_Confidence_0.909_Length_2286, vo_c2_Locus_3512_Transcript_3/4_Confidence_0.500_Length_2501, vo_n2_Locus_4820_Transcript_2/3_Confidence_0.545_Length_2376, vo_n3_Locus_2644_Transcript_11/11_Confidence_0.323_Length_2390, vo_n2_Locus_515_Transcript_8/9_Confidence_0.471_Length_3194, vo_n2_Locus_515_Transcript_7/9_Confidence_0.176_Length_2355, vo_c2_Locus_3512_Transcript_2/4_Confidence_0.750_Length_2320, vo_c3_Locus_1501_Transcript_6/9_Confidence_0.203_Length_2420, vo_n2_Locus_5127_Transcript_1/3_Confidence_0.200_Length_1636, vo_n2_Locus_22912_Transcript_1/2_Confidence_0.333_Length_2481, vo_c1_Locus_9668_Transcript_5/11_Confidence_0.391_Length_1245, vo_c2_Locus_3988_Transcript_2/10_Confidence_0.500_Length_1233, vo_n3_Locus_4554_Transcript_5/8_Confidence_0.600_Length_1377, vo_c2_Locus_3988_Transcript_1/10_Confidence_0.607_Length_1361, vo_n1_Locus_2626_Transcript_19/26_Confidence_0.238_Length_3851, vo_c1_Locus_9668_Transcript_4/11_Confidence_0.435_Length_1360, vo_n1_Locus_2626_Transcript_15/26_Confidence_0.262_Length_1311,

					vo_c1_Locus_9668_Transcript_6/11_Confidence_0.348_Length_1630, vo_n2_Locus_3_Transcript_5064/24291_Confidence_0.000_Length_1233, vo_n3_Locus_4554_Transcript_6/8_Confidence_0.533_Length_1337, vo_c2_Locus_3988_Transcript_5/10_Confidence_0.536_Length_2785, vo_n1_Locus_2626_Transcript_18/26_Confidence_0.310_Length_2079, vo_c2_Locus_3988_Transcript_7/10_Confidence_0.500_Length_1273, vo_n2_Locus_3_Transcript_5069/24291_Confidence_0.000_Length_1240, vo_c2_Locus_3988_Transcript_9/10_Confidence_0.429_Length_1401, vo_n2_Locus_854_Transcript_1/1_Confidence_0.000_Length_1753, vo_c2_Locus_4368_Transcript_2/25_Confidence_0.375_Length_6775, vo_c1_Locus_13166_Transcript_27/32_Confidence_0.394_Length_6627, vo_n2_Locus_3_Transcript_20516/24291_Confidence_0.000_Length_6564, vo_c3_Locus_3082_Transcript_7/7_Confidence_0.167_Length_5439, vo_n1_Locus_991_Transcript_1/21_Confidence_0.113_Length_1473, tri_c_comp19198_c0_seq1, vo_c1_Locus_13166_Transcript_28/32_Confidence_0.409_Length_6630, vo_n1_Locus_7772_Transcript_1/1_Confidence_0.000_Length_6986, tri_c_comp19198_c0_seq4, tri_c_comp19198_c0_seq3, vo_n3_Locus_4402_Transcript_3/18_Confidence_0.269_Length_6798, vo_c3_Locus_3082_Transcript_5/7_Confidence_0.333_Length_2051, vo_c1_Locus_2657_Transcript_1/1_Confidence_0.000_Length_1513, vo_n2_Locus_23058_Transcript_1/1_Confidence_0.000_Length_1342, vo_c2_Locus_4832_Transcript_1/1_Confidence_0.000_Length_1285, vo_c3_Locus_4005_Transcript_5/6_Confidence_0.167_Length_1517, vo_c1_Locus_6811_Transcript_7/8_Confidence_0.364_Length_589, vo_n2_Locus_297_Transcript_2/5_Confidence_0.636_Length_1679, vo_c2_Locus_1080_Transcript_4/21_Confidence_0.241_Length_1741, vo_c2_Locus_1080_Transcript_3/21_Confidence_0.207_Length_1702, vo_n3_Locus_1253_Transcript_1/2_Confidence_0.500_Length_1626, vo_c2_Locus_1080_Transcript_8/21_Confidence_0.138_Length_1077, vo_c2_Locus_4581_Transcript_3/8_Confidence_0.684_Length_1578, vo_n2_Locus_901_Transcript_1/95_Confidence_0.023_Length_1512, vo_n2_Locus_20307_Transcript_6/7_Confidence_0.062_Length_2030, vo_n3_Locus_1253_Transcript_2/2_Confidence_0.000_Length_1587, vo_n2_Locus_10311_Transcript_5/6_Confidence_0.357_Length_778, vo_n2_Locus_10311_Transcript_2/6_Confidence_0.500_Length_1843, vo_c2_Locus_1465_Transcript_1/1_Confidence_0.000_Length_2684, vo_n2_Locus_668_Transcript_12/17_Confidence_0.465_Length_3343, vo_n3_Locus_1281_Transcript_13/14_Confidence_0.467_Length_2089, vo_n2_Locus_668_Transcript_10/17_Confidence_0.419_Length_1865, vo_c3_Locus_6604_Transcript_15/15_Confidence_0.590_Length_1792, vo_n3_Locus_1281_Transcript_3/14_Confidence_0.533_Length_1904, vo_c3_Locus_6604_Transcript_12/15_Confidence_0.230_Length_1729, tri_n_comp22443_c0_seq1, vo_c2_Locus_2191_Transcript_14/17_Confidence_0.464_Length_1841, tri_n_comp15079_c0_seq3, vo_c1_Locus_2380_Transcript_17/17_Confidence_0.086_Length_3951, vo_c2_Locus_191_Transcript_2/2_Confidence_0.167_Length_3106, vo_c1_Locus_2380_Transcript_3/17_Confidence_0.371_Length_12857, tri_c_comp21028_c0_seq2, tri_c_comp21028_c0_seq3, vo_c2_Locus_354_Transcript_3/17_Confidence_0.667_Length_4966, vo_n1_Locus_30469_Transcript_2/2_Confidence_0.000_Length_979, vo_n3_Locus_4489_Transcript_2/3_Confidence_0.444_Length_2229, vo_n1_Locus_1163_Transcript_25/49_Confidence_0.232_Length_2084, vo_n1_Locus_1163_Transcript_5/49_Confidence_0.087_Length_1841, vo_n1_Locus_1163_Transcript_28/49_Confidence_0.319_Length_2335, vo_c2_Locus_352_Transcript_3/4_Confidence_0.889_Length_1920, vo_n1_Locus_1163_Transcript_13/49_Confidence_0.203_Length_2083, vo_n1_Locus_1163_Transcript_18/49_Confidence_0.319_Length_2098, vo_n3_Locus_527_Transcript_24/31_Confidence_0.194_Length_2036, vo_n3_Locus_527_Transcript_17/31_Confidence_0.343_Length_2078, vo_c2_Locus_6197_Transcript_2/4_Confidence_0.143_Length_1400, vo_n2_Locus_3_Transcript_20033/24291_Confidence_0.000_Length_1861, vo_c2_Locus_364_Transcript_15/30_Confidence_0.278_Length_2035, vo_n2_Locus_3_Transcript_20001/24291_Confidence_0.000_Length_2052, vo_c2_Locus_364_Transcript_11/30_Confidence_0.306_Length_2035, vo_c2_Locus_364_Transcript_5/30_Confidence_0.292_Length_2051, vo_c2_Locus_364_Transcript_9/30_Confidence_0.194_Length_2033, vo_c3_Locus_145_Transcript_31/32_Confidence_0.014_Length_533, vo_n1_Locus_1163_Transcript_23/49_Confidence_0.290_Length_2037, vo_n2_Locus_3_Transcript_19989/24291_Confidence_0.000_Length_1977, vo_n2_Locus_3_Transcript_20027/24291_Confidence_0.000_Length_2905, vo_n3_Locus_527_Transcript_18/31_Confidence_0.388_Length_2047, tri_c_comp17405_c0_seq2, tri_c_comp20952_c0_seq1, vo_n1_Locus_1163_Transcript_44/49_Confidence_0.101_Length_1863, vo_n3_Locus_527_Transcript_29/31_Confidence_0.328_Length_2078, vo_c2_Locus_364_Transcript_24/30_Confidence_0.250_Length_1867, vo_n1_Locus_1163_Transcript_10/49_Confidence_0.246_Length_2083, vo_c2_Locus_364_Transcript_17/30_Confidence_0.306_Length_2052, vo_n3_Locus_527_Transcript_6/31_Confidence_0.313_Length_2013, vo_n2_Locus_3_Transcript_20024/24291_Confidence_0.000_Length_2008, vo_c3_Locus_4822_Transcript_1/2_Confidence_0.714_Length_1522, vo_c1_Locus_1081_Transcript_12/28_Confidence_0.279_Length_1129, vo_n1_Locus_10488_Transcript_5/10_Confidence_0.438_Length_1440, tri_n_comp11728_c0_seq1, vo_c2_Locus_2688_Transcript_17/23_Confidence_0.279_Length_1682, vo_c1_Locus_4014_Transcript_24/28_Confidence_0.138_Length_2429, vo_c1_Locus_4014_Transcript_20/28_Confidence_0.117_Length_1747, vo_c1_Locus_4014_Transcript_28/28_Confidence_0.085_Length_1888, vo_c1_Locus_4014_Transcript_6/28_Confidence_0.404_Length_1911, tri_n_comp77429_c0_seq1, vo_n1_Locus_14_Transcript_1/27_Confidence_0.261_Length_1848, tri_n_comp20167_c0_seq2, vo_n3_Locus_4343_Transcript_9/16_Confidence_0.200_Length_1729, vo_c3_Locus_10348_Transcript_1/2_Confidence_0.875_Length_1734, vo_c1_Locus_4014_Transcript_12/28_Confidence_0.245_Length_1582, vo_c3_Locus_1723_Transcript_29/37_Confidence_0.411_Length_1695, vo_n2_Locus_330_Transcript_25/38_Confidence_0.361_Length_1516, vo_c3_Locus_1723_Transcript_35/37_Confidence_0.464_Length_1702, vo_c1_Locus_15206_Transcript_3/3_Confidence_0.000_Length_1312, vo_c1_Locus_7371_Transcript_4/4_Confidence_0.600_Length_1352, vo_c3_Locus_1723_Transcript_37/37_Confidence_0.214_Length_1530, vo_n1_Locus_769_Transcript_28/41_Confidence_0.400_Length_1609, vo_n1_Locus_769_Transcript_9/41_Confidence_0.460_Length_1494, vo_c3_Locus_2965_Transcript_2/2_Confidence_0.250_Length_1327, vo_n2_Locus_330_Transcript_5/38_Confidence_0.328_Length_11715, vo_n1_Locus_9772_Transcript_1/2_Confidence_0.333_Length_1224, vo_c1_Locus_3603_Transcript_44/48_Confidence_0.492_Length_1696, vo_c2_Locus_4242_Transcript_2/3_Confidence_0.400_Length_1168, vo_n1_Locus_6649_Transcript_2/3_Confidence_0.500_Length_2124, vo_c1_Locus_7371_Transcript_1/4_Confidence_0.500_Length_1352, vo_n3_Locus_2381_Transcript_2/3_Confidence_0.500_Length_1330, vo_c3_Locus_1723_Transcript_16/37_Confidence_0.286_Length_1491,

					vo_n1_Locus_769_Transcript_36/41_Confidence_0.420_Length_1536, vo_n1_Locus_769_Transcript_40/41_Confidence_0.320_Length_1479, vo_n1_Locus_6649_Transcript_1/3_Confidence_0.750_Length_1883, vo_c1_Locus_3603_Transcript_26/48_Confidence_0.400_Length_1432, vo_n1_Locus_5348_Transcript_2/2_Confidence_0.200_Length_1608, vo_n3_Locus_3383_Transcript_35/37_Confidence_0.389_Length_1512, tri_c_comp20802_c0_seq1, vo_c1_Locus_2815_Transcript_3/4_Confidence_0.200_Length_2244

Valine, leucine and isoleucine degradation	map00280	16	ec:2.6.1.18 - transaminase, ec:1.2.1.27 - dehydrogenase (CoA-acylating), ec:2.6.1.40 - transaminase, ec:2.6.1.42 - transaminase, ec:1.8.1.4 - dehydrogenase, ec:1.2.1.3 - dehydrogenase (NAD+), ec:2.3.1.9 - C-acetyltransferase, ec:2.3.3.10 - synthase, ec:1.3.99.12 - dehydrogenase, ec:1.3.8.1 - acyl-CoA dehydrogenase, ec:2.8.3.5 - CoA-transferase, ec:3.1.2.4 - hydrolase, ec:4.2.1.17 - hydratase, ec:1.1.1.35 - dehydrogenase, ec:1.1.1.31 - dehydrogenase, ec:1.2.4.4 - dehydrogenase (2-methylpropanoyl-transferring)	1, 3, 1, 6, 2, 5, 2, 4, 1, 2, 1, 1, 7, 16, 5, 1	vo_c1_Locus_7220_Transcript_2/3_Confidence_0.400_Length_2898, vo_c3_Locus_1581_Transcript_3/3_Confidence_0.000_Length_1736, vo_c1_Locus_1298_Transcript_3/6_Confidence_0.625_Length_1775, vo_n2_Locus_157_Transcript_2/4_Confidence_0.286_Length_2059, vo_c1_Locus_7220_Transcript_2/3_Confidence_0.400_Length_2898, vo_c2_Locus_384_Transcript_2/3_Confidence_0.400_Length_1725, tri_c_comp32679_c0_seq1, vo_c1_Locus_10788_Transcript_1/1_Confidence_0.000_Length_1721, vo_c2_Locus_14969_Transcript_4/8_Confidence_0.750_Length_1231, vo_n2_Locus_3786_Transcript_2/4_Confidence_0.778_Length_1466, vo_n2_Locus_3786_Transcript_3/4_Confidence_0.667_Length_1442, vo_n3_Locus_6810_Transcript_2/3_Confidence_0.913_Length_1699, vo_n1_Locus_6460_Transcript_1/1_Confidence_0.000_Length_1912, tri_c_comp55340_c0_seq1, vo_n1_Locus_19864_Transcript_2/2_Confidence_0.000_Length_2707, vo_c3_Locus_21741_Transcript_5/9_Confidence_0.583_Length_1757, vo_n3_Locus_25600_Transcript_5/6_Confidence_0.375_Length_1777, tri_n_comp58061_c0_seq1, vo_c1_Locus_2657_Transcript_1/1_Confidence_0.000_Length_1513, vo_n2_Locus_23058_Transcript_1/1_Confidence_0.000_Length_1342, vo_n2_Locus_634_Transcript_4/6_Confidence_0.357_Length_8557, tri_c_comp57035_c0_seq1, vo_c1_Locus_1809_Transcript_1/1_Confidence_0.000_Length_3087, vo_c3_Locus_18718_Transcript_1/1_Confidence_0.000_Length_2686, vo_c3_Locus_18248_Transcript_1/2_Confidence_0.667_Length_1596, tri_c_comp7217_c0_seq2, vo_c3_Locus_18248_Transcript_1/2_Confidence_0.667_Length_1596, vo_c2_Locus_6864_Transcript_3/5_Confidence_0.667_Length_1701, vo_c3_Locus_6234_Transcript_1/1_Confidence_0.000_Length_1326, vo_c2_Locus_2261_Transcript_1/2_Confidence_1.000_Length_2564, vo_c3_Locus_5948_Transcript_1/1_Confidence_0.000_Length_1027, vo_n2_Locus_16945_Transcript_1/3_Confidence_0.500_Length_1084, vo_c3_Locus_9925_Transcript_8/11_Confidence_0.263_Length_3550, vo_n1_Locus_11137_Transcript_5/5_Confidence_0.143_Length_2829, vo_n2_Locus_16945_Transcript_3/3_Confidence_0.000_Length_746, vo_n2_Locus_30747_Transcript_1/1_Confidence_0.000_Length_951, vo_c2_Locus_2261_Transcript_1/2_Confidence_1.000_Length_2564, vo_n1_Locus_9414_Transcript_8/13_Confidence_0.167_Length_606, vo_c1_Locus_40339_Transcript_1/1_Confidence_0.000_Length_1318, vo_c3_Locus_2031_Transcript_8/8_Confidence_0.111_Length_1389, vo_n1_Locus_11137_Transcript_5/5_Confidence_0.143_Length_2829, vo_c1_Locus_4675_Transcript_3/3_Confidence_0.200_Length_1348, vo_n2_Locus_30747_Transcript_1/1_Confidence_0.000_Length_951, vo_n2_Locus_636_Transcript_13/16_Confidence_0.222_Length_768, vo_c1_Locus_2815_Transcript_3/4_Confidence_0.200_Length_2244, vo_c3_Locus_9925_Transcript_8/11_Confidence_0.263_Length_3550, tri_n_comp42565_c0_seq1, vo_c2_Locus_189_Transcript_29/60_Confidence_0.015_Length_692, vo_n2_Locus_636_Transcript_16/16_Confidence_0.139_Length_616, vo_n2_Locus_636_Transcript_14/16_Confidence_0.222_Length_622, vo_n2_Locus_636_Transcript_5/16_Confidence_0.194_Length_591, vo_n2_Locus_636_Transcript_3/16_Confidence_0.306_Length_685, tri_c_comp25671_c0_seq1, tri_n_comp18543_c0_seq8, vo_c2_Locus_14292_Transcript_1/3_Confidence_0.857_Length_1370, vo_c2_Locus_14292_Transcript_3/3_Confidence_0.429_Length_1343, vo_c2_Locus_13495_Transcript_4/17_Confidence_0.346_Length_1808, vo_c1_Locus_5714_Transcript_2/3_Confidence_0.714_Length_2588

Fructose and mannose metabolism	map00051	16	ec:1.1.1.187 - reductase, ec:2.7.1.105 - phosphofructokinase 2, ec:2.7.1.90 - 1-phosphotransferase, ec:2.7.1.11 - phosphohexokinase, ec:5.3.1.1 - isomerase, ec:5.3.1.8 - isomerase, ec:5.3.1.6 - isomerase, ec:4.1.2.13 - aldolase, ec:3.1.3.46 - 2-phosphatase, ec:3.1.3.11 - hexose diphosphatase, ec:3.6.1.21 - diphosphatase, ec:1.1.1.271 - synthase, ec:4.2.1.47 - 4,6-dehydratase, ec:1.1.1.67 - 2-dehydrogenase, ec:1.1.1.14 - 2-dehydrogenase, ec:1.1.1.21 - reductase	1, 5, 1, 5, 9, 2, 2, 38, 6, 8, 5, 1, 3, 1, 3, 4	vo_c2_Locus_4832_Transcript_1/1_Confidence_0.000_Length_1285, vo_c3_Locus_21070_Transcript_1/1_Confidence_0.000_Length_1698, vo_c3_Locus_6327_Transcript_1/2_Confidence_0.750_Length_2258, tri_c_comp28824_c0_seq1, vo_n1_Locus_11871_Transcript_1/1_Confidence_0.000_Length_1701, vo_c3_Locus_6327_Transcript_2/2_Confidence_0.250_Length_2279, vo_c2_Locus_6348_Transcript_1/2_Confidence_1.000_Length_3755, vo_c3_Locus_1613_Transcript_6/17_Confidence_0.158_Length_5809, vo_n1_Locus_10193_Transcript_1/1_Confidence_0.000_Length_1647, vo_c2_Locus_6348_Transcript_1/2_Confidence_1.000_Length_3755, vo_n3_Locus_23556_Transcript_2/4_Confidence_0.500_Length_4210, tri_c_comp43537_c0_seq1, vo_c2_Locus_3634_Transcript_8/8_Confidence_0.133_Length_2428, vo_c2_Locus_324_Transcript_5/12_Confidence_0.333_Length_1009, vo_n1_Locus_5721_Transcript_6/9_Confidence_0.389_Length_989, vo_c2_Locus_324_Transcript_11/12_Confidence_0.333_Length_4393, vo_n2_Locus_475_Transcript_7/13_Confidence_0.312_Length_1141, vo_n1_Locus_5721_Transcript_1/9_Confidence_0.278_Length_986, vo_n2_Locus_475_Transcript_2/13_Confidence_0.312_Length_1187, vo_c2_Locus_324_Transcript_2/12_Confidence_0.400_Length_1106, vo_c3_Locus_3927_Transcript_2/9_Confidence_0.250_Length_2629, vo_c2_Locus_1828_Transcript_1/2_Confidence_0.963_Length_1886, vo_n2_Locus_19533_Transcript_1/1_Confidence_0.000_Length_1783, vo_c1_Locus_34898_Transcript_1/1_Confidence_0.000_Length_1083, vo_n2_Locus_8097_Transcript_1/6_Confidence_0.500_Length_1196, vo_n1_Locus_565_Transcript_31/31_Confidence_0.178_Length_3922, vo_c3_Locus_2800_Transcript_3/23_Confidence_0.566_Length_1690, vo_c3_Locus_2800_Transcript_9/23_Confidence_0.585_Length_1396, tri_n_comp21653_c0_seq1, vo_n1_Locus_565_Transcript_5/31_Confidence_0.244_Length_1533, vo_n2_Locus_4661_Transcript_5/12_Confidence_0.208_Length_1240, vo_n1_Locus_565_Transcript_17/31_Confidence_0.422_Length_1454, vo_c3_Locus_2800_Transcript_5/23_Confidence_0.566_Length_2133, vo_c1_Locus_1757_Transcript_17/19_Confidence_0.375_Length_1367, vo_c1_Locus_298_Transcript_4/32_Confidence_0.255_Length_1585, vo_n1_Locus_8402_Transcript_3/4_Confidence_0.286_Length_1464, vo_c3_Locus_2800_Transcript_23/23_Confidence_0.151_Length_1466, vo_n2_Locus_903_Transcript_38/40_Confidence_0.222_Length_6612, vo_n1_Locus_565_Transcript_20/31_Confidence_0.444_Length_1622, vo_n2_Locus_2046_Transcript_7/18_Confidence_0.345_Length_1296, vo_c3_Locus_2800_Transcript_8/23_Confidence_0.585_Length_1344, vo_n3_Locus_1119_Transcript_19/50_Confidence_0.050_Length_498, vo_c2_Locus_5742_Transcript_10/21_Confidence_0.244_Length_1454, vo_n3_Locus_1119_Transcript_11/50_Confidence_0.124_Length_2839, vo_c2_Locus_5742_Transcript_18/21_Confidence_0.317_Length_2233, vo_n1_Locus_565_Transcript_13/31_Confidence_0.378_Length_1400, vo_c3_Locus_2800_Transcript_14/23_Confidence_0.321_Length_1426, vo_c3_Locus_614_Transcript_19/24_Confidence_0.394_Length_3443, vo_c2_Locus_358_Transcript_1/41_Confidence_0.080_Length_1708, vo_c2_Locus_5742_Transcript_14/21_Confidence_0.439_Length_2413, vo_c1_Locus_1757_Transcript_8/19_Confidence_0.458_Length_1460, vo_n1_Locus_986_Transcript_16/36_Confidence_0.211_Length_1811, vo_n1_Locus_565_Transcript_16/31_Confidence_0.400_Length_1499, vo_n2_Locus_903_Transcript_37/40_Confidence_0.346_Length_3926, tri_c_comp20312_c0_seq4, vo_c3_Locus_614_Transcript_13/24_Confidence_0.061_Length_4970, vo_n2_Locus_2046_Transcript_11/18_Confidence_0.310_Length_1666, vo_n2_Locus_903_Transcript_31/40_Confidence_0.321_Length_2251, vo_n1_Locus_565_Transcript_30/31_Confidence_0.422_Length_3814, vo_c3_Locus_614_Transcript_2/24_Confidence_0.485_Length_1777, vo_n1_Locus_5148_Transcript_1/4_Confidence_0.667_Length_1983, vo_c1_Locus_10454_Transcript_1/2_Confidence_0.750_Length_1441, vo_n3_Locus_1119_Transcript_18/50_Confidence_0.149_Length_1959, vo_c3_Locus_21070_Transcript_1/1_Confidence_0.000_Length_1698, vo_c3_Locus_6327_Transcript_1/2_Confidence_0.750_Length_2258, tri_c_comp28824_c0_seq1, vo_n1_Locus_11871_Transcript_1/1_Confidence_0.000_Length_1701, vo_n3_Locus_27_Transcript_1/1_Confidence_0.000_Length_1278, vo_c3_Locus_6327_Transcript_2/2_Confidence_0.250_Length_2279, vo_c2_Locus_40594_Transcript_2/3_Confidence_0.500_Length_1728, vo_n2_Locus_39295_Transcript_2/2_Confidence_0.500_Length_1939, vo_c2_Locus_6460_Transcript_1/2_Confidence_1.000_Length_1563, vo_n1_Locus_3261_Transcript_3/4_Confidence_0.667_Length_1633, vo_n3_Locus_27_Transcript_1/1_Confidence_0.000_Length_1278, tri_c_comp23875_c0_seq1, tri_c_comp24147_c0_seq1, vo_c1_Locus_4576_Transcript_2/2_Confidence_0.000_Length_1644, vo_c2_Locus_35825_Transcript_1/2_Confidence_0.500_Length_995, vo_c2_Locus_35825_Transcript_2/2_Confidence_0.000_Length_972, vo_c1_Locus_18906_Transcript_3/4_Confidence_0.400_Length_1298, tri_n_comp34093_c0_seq1, vo_c2_Locus_11939_Transcript_1/4_Confidence_0.273_Length_1336, vo_c2_Locus_4832_Transcript_1/1_Confidence_0.000_Length_1285, tri_c_comp20306_c0_seq1, vo_c1_Locus_3665_Transcript_26/28_Confidence_0.462_Length_1222, vo_c2_Locus_1898_Transcript_1/15_Confidence_0.462_Length_1289, vo_n1_Locus_13598_Transcript_3/3_Confidence_0.143_Length_1862, tri_n_comp61454_c0_seq1, vo_c3_Locus_27324_Transcript_1/1_Confidence_0.000_Length_1468, vo_n3_Locus_17668_Transcript_1/2_Confidence_1.000_Length_1468, vo_c1_Locus_13556_Transcript_2/2_Confidence_0.000_Length_1297, tri_c_comp22527_c0_seq1, vo_n1_Locus_10504_Transcript_1/2_Confidence_0.750_Length_1211, vo_c2_Locus_9062_Transcript_1/11_Confidence_0.312_Length_4797

Methane metabolism	map00680	16	ec:2.6.1.52 - transaminase, ec:4.1.3.24 - lyase, ec:1.2.1.46 - dehydrogenase, ec:2.7.1.29 - kinase, ec:2.7.1.11 - phosphohexokinase, ec:6.2.1.1 - ligase, ec:3.1.2.12 - hydrolase, ec:3.1.3.3 - phosphatase, ec:2.1.2.1 - hydroxymethyltransferase, ec:4.1.2.13 - aldolase, ec:3.1.3.11 - hexose diphosphatase, ec:1.1.1.284 - dehydrogenase, ec:4.2.1.11 - hydratase, ec:2.5.1.77 - synthase, ec:2.7.9.2 - water dikinase, ec:1.1.1.37 - dehydrogenase	4, 15, 2, 2, 5, 1, 2, 2, 9, 38, 8, 2, 23, 1, 15, 24	vo_n2_Locus_2440_Transcript_3/6_Confidence_0.182_Length_1751, tri_c_comp24934_c0_seq1, vo_c3_Locus_9067_Transcript_6/7_Confidence_0.125_Length_1757, vo_c2_Locus_1886_Transcript_3/5_Confidence_0.444_Length_2764, vo_c1_Locus_9668_Transcript_5/11_Confidence_0.391_Length_1245, vo_c2_Locus_3988_Transcript_2/10_Confidence_0.500_Length_1233, vo_n3_Locus_4554_Transcript_5/8_Confidence_0.600_Length_1377, vo_c2_Locus_3988_Transcript_1/10_Confidence_0.607_Length_1361, vo_n1_Locus_2626_Transcript_19/26_Confidence_0.238_Length_3851, vo_c1_Locus_9668_Transcript_4/11_Confidence_0.435_Length_1360, vo_n1_Locus_2626_Transcript_15/26_Confidence_0.262_Length_1311, vo_c1_Locus_9668_Transcript_6/11_Confidence_0.348_Length_1630, vo_n2_Locus_3_Transcript_5064/24291_Confidence_0.000_Length_1233, vo_n3_Locus_4554_Transcript_6/8_Confidence_0.533_Length_1337, vo_c2_Locus_3988_Transcript_5/10_Confidence_0.536_Length_2785, vo_n1_Locus_2626_Transcript_18/26_Confidence_0.310_Length_2079, vo_c2_Locus_3988_Transcript_7/10_Confidence_0.500_Length_1273, vo_n2_Locus_3_Transcript_5069/24291_Confidence_0.000_Length_1240, vo_c2_Locus_3988_Transcript_9/10_Confidence_0.429_Length_1401, vo_c3_Locus_11405_Transcript_4/5_Confidence_0.429_Length_4324, vo_c3_Locus_15849_Transcript_1/1_Confidence_0.000_Length_1425, vo_n1_Locus_15871_Transcript_1/1_Confidence_0.000_Length_1944, vo_c2_Locus_10095_Transcript_1/4_Confidence_0.286_Length_4859, vo_c3_Locus_1613_Transcript_6/17_Confidence_0.158_Length_5809, vo_n1_Locus_10193_Transcript_1/1_Confidence_0.000_Length_1647, vo_c2_Locus_6348_Transcript_1/2_Confidence_1.000_Length_3755, vo_n3_Locus_23556_Transcript_2/4_Confidence_0.500_Length_4210, tri_c_comp43537_c0_seq1, vo_n3_Locus_9269_Transcript_1/1_Confidence_0.000_Length_2238, vo_n2_Locus_8305_Transcript_5/8_Confidence_0.143_Length_2284, vo_n1_Locus_29308_Transcript_2/2_Confidence_0.000_Length_535, vo_c3_Locus_3840_Transcript_2/5_Confidence_0.200_Length_1699, vo_c2_Locus_27691_Transcript_1/2_Confidence_0.750_Length_923, vo_c2_Locus_1465_Transcript_1/1_Confidence_0.000_Length_2684, vo_n2_Locus_668_Transcript_12/17_Confidence_0.465_Length_3343, vo_n3_Locus_1281_Transcript_13/14_Confidence_0.467_Length_2089, vo_n2_Locus_668_Transcript_10/17_Confidence_0.419_Length_1865, vo_c3_Locus_6604_Transcript_15/15_Confidence_0.590_Length_1792, vo_n3_Locus_1281_Transcript_3/14_Confidence_0.533_Length_1904, vo_c3_Locus_6604_Transcript_12/15_Confidence_0.230_Length_1729, tri_n_comp22443_c0_seq1, vo_c2_Locus_2191_Transcript_14/17_Confidence_0.464_Length_1841, vo_n1_Locus_565_Transcript_31/31_Confidence_0.178_Length_3922, vo_c3_Locus_2800_Transcript_3/23_Confidence_0.566_Length_1690, vo_c3_Locus_2800_Transcript_9/23_Confidence_0.585_Length_1396, tri_n_comp21653_c0_seq1, vo_n1_Locus_565_Transcript_5/31_Confidence_0.244_Length_1533, vo_n2_Locus_4661_Transcript_5/12_Confidence_0.208_Length_1240, vo_n1_Locus_565_Transcript_17/31_Confidence_0.422_Length_1454, vo_c3_Locus_2800_Transcript_5/23_Confidence_0.566_Length_2133, vo_c1_Locus_1757_Transcript_17/19_Confidence_0.375_Length_1367, vo_c1_Locus_298_Transcript_4/32_Confidence_0.255_Length_1585, vo_n1_Locus_8402_Transcript_3/4_Confidence_0.286_Length_1464, vo_c3_Locus_2800_Transcript_23/23_Confidence_0.151_Length_1466, vo_n2_Locus_903_Transcript_38/40_Confidence_0.222_Length_6612, vo_n1_Locus_565_Transcript_20/31_Confidence_0.444_Length_1622, vo_n2_Locus_2046_Transcript_7/18_Confidence_0.345_Length_1296, vo_c3_Locus_2800_Transcript_8/23_Confidence_0.585_Length_1344, vo_n3_Locus_1119_Transcript_19/50_Confidence_0.050_Length_498, vo_c2_Locus_5742_Transcript_10/21_Confidence_0.244_Length_1454, vo_n3_Locus_1119_Transcript_11/50_Confidence_0.124_Length_2839, vo_c2_Locus_5742_Transcript_18/21_Confidence_0.317_Length_2233, vo_n1_Locus_565_Transcript_13/31_Confidence_0.378_Length_1400, vo_c3_Locus_2800_Transcript_14/23_Confidence_0.321_Length_1426, vo_c3_Locus_614_Transcript_19/24_Confidence_0.394_Length_3443, vo_c2_Locus_358_Transcript_1/41_Confidence_0.080_Length_1708, vo_c2_Locus_5742_Transcript_14/21_Confidence_0.439_Length_2413, vo_c1_Locus_1757_Transcript_8/19_Confidence_0.458_Length_1460, vo_n1_Locus_986_Transcript_16/36_Confidence_0.211_Length_1811, vo_n1_Locus_565_Transcript_16/31_Confidence_0.400_Length_1499, vo_n2_Locus_903_Transcript_37/40_Confidence_0.346_Length_3926, tri_c_comp20312_c0_seq4, vo_c3_Locus_614_Transcript_13/24_Confidence_0.061_Length_4970, vo_n2_Locus_2046_Transcript_11/18_Confidence_0.310_Length_1666, vo_n2_Locus_903_Transcript_31/40_Confidence_0.321_Length_2251, vo_n1_Locus_565_Transcript_30/31_Confidence_0.422_Length_3814, vo_c3_Locus_614_Transcript_2/24_Confidence_0.485_Length_1777, vo_n1_Locus_5148_Transcript_1/4_Confidence_0.667_Length_1983, vo_c1_Locus_10454_Transcript_1/2_Confidence_0.750_Length_1441, vo_n3_Locus_1119_Transcript_18/50_Confidence_0.149_Length_1959, vo_c2_Locus_40594_Transcript_2/3_Confidence_0.500_Length_1728, vo_n2_Locus_39295_Transcript_2/2_Confidence_0.500_Length_1939, vo_c2_Locus_6460_Transcript_1/2_Confidence_1.000_Length_1563, vo_n1_Locus_3261_Transcript_3/4_Confidence_0.667_Length_1633, vo_n3_Locus_27_Transcript_1/1_Confidence_0.000_Length_1278, tri_c_comp23875_c0_seq1, tri_c_comp24147_c0_seq1, vo_c1_Locus_4576_Transcript_2/2_Confidence_0.000_Length_1644, vo_c3_Locus_11405_Transcript_4/5_Confidence_0.429_Length_4324, vo_c3_Locus_15849_Transcript_1/1_Confidence_0.000_Length_1425, tri_n_comp18813_c0_seq8, tri_c_comp19022_c0_seq2, vo_n2_Locus_106_Transcript_27/30_Confidence_0.078_Length_2735, vo_c2_Locus_2699_Transcript_2/7_Confidence_0.471_Length_1721, vo_n2_Locus_106_Transcript_24/30_Confidence_0.219_Length_3708, vo_n3_Locus_362_Transcript_1/4_Confidence_0.727_Length_3306, vo_n3_Locus_3996_Transcript_1/4_Confidence_0.417_Length_1642, vo_n1_Locus_3710_Transcript_9/10_Confidence_0.500_Length_1616, vo_n2_Locus_106_Transcript_25/30_Confidence_0.188_Length_2659, vo_n2_Locus_1384_Transcript_2/5_Confidence_0.308_Length_2123, vo_n3_Locus_3996_Transcript_3/4_Confidence_0.500_Length_3581, tri_n_comp18813_c0_seq3, vo_c1_Locus_6488_Transcript_4/4_Confidence_0.250_Length_8239, vo_c2_Locus_1551_Transcript_20/27_Confidence_0.238_Length_2634, tri_n_comp18813_c0_seq13, vo_n3_Locus_685_Transcript_7/8_Confidence_0.353_Length_1680, vo_n2_Locus_1324_Transcript_10/14_Confidence_0.296_Length_1766, vo_c3_Locus_1613_Transcript_6/17_Confidence_0.158_Length_5809, vo_n3_Locus_685_Transcript_3/8_Confidence_0.471_Length_1755, vo_c2_Locus_16827_Transcript_1/1_Confidence_0.000_Length_1783, vo_n3_Locus_362_Transcript_3/4_Confidence_0.364_Length_3895, vo_c3_Locus_1613_Transcript_11/17_Confidence_0.316_Length_2455, tri_c_comp13285_c0_seq1, tri_n_comp12586_c0_seq4, vo_c1_Locus_1662_Transcript_19/44_Confidence_0.278_Length_3148,

					vo_n2_Locus_1146_Transcript_9/32_Confidence_0.492_Length_2897, vo_n3_Locus_3213_Transcript_1/29_Confidence_0.022_Length_515, vo_n1_Locus_1032_Transcript_1/39_Confidence_0.018_Length_458, tri_n_comp20108_c0_seq2, vo_c1_Locus_1662_Transcript_28/44_Confidence_0.200_Length_3087, vo_c2_Locus_49_Transcript_21/157_Confidence_0.007_Length_465, vo_c2_Locus_49_Transcript_31/157_Confidence_0.027_Length_1554, vo_c2_Locus_49_Transcript_20/157_Confidence_0.037_Length_3535, vo_c1_Locus_1662_Transcript_6/44_Confidence_0.211_Length_3160, vo_c2_Locus_49_Transcript_28/157_Confidence_0.071_Length_2983, vo_n1_Locus_1032_Transcript_5/39_Confidence_0.404_Length_3016, vo_n1_Locus_1032_Transcript_38/39_Confidence_0.228_Length_2924, vo_n1_Locus_1032_Transcript_8/39_Confidence_0.386_Length_2954, vo_c3_Locus_1031_Transcript_12/33_Confidence_0.385_Length_3145, vo_c3_Locus_1723_Transcript_29/37_Confidence_0.411_Length_1695, vo_n2_Locus_330_Transcript_25/38_Confidence_0.361_Length_1516, vo_c3_Locus_1723_Transcript_35/37_Confidence_0.464_Length_1702, vo_c1_Locus_15206_Transcript_3/3_Confidence_0.000_Length_1312, vo_c1_Locus_7371_Transcript_4/4_Confidence_0.600_Length_1352, vo_c3_Locus_1723_Transcript_37/37_Confidence_0.214_Length_1530, vo_n1_Locus_769_Transcript_28/41_Confidence_0.400_Length_1609, vo_n1_Locus_769_Transcript_9/41_Confidence_0.460_Length_1494, vo_c3_Locus_2965_Transcript_2/2_Confidence_0.250_Length_1327, vo_n2_Locus_330_Transcript_5/38_Confidence_0.328_Length_11715, vo_n1_Locus_9772_Transcript_1/2_Confidence_0.333_Length_1224, vo_c1_Locus_3603_Transcript_44/48_Confidence_0.492_Length_1696, vo_c2_Locus_4242_Transcript_2/3_Confidence_0.400_Length_1168, vo_n1_Locus_6649_Transcript_2/3_Confidence_0.500_Length_2124, vo_c1_Locus_7371_Transcript_1/4_Confidence_0.500_Length_1352, vo_n3_Locus_2381_Transcript_2/3_Confidence_0.500_Length_1330, vo_c3_Locus_1723_Transcript_16/37_Confidence_0.286_Length_1491, vo_n1_Locus_769_Transcript_36/41_Confidence_0.420_Length_1536, vo_n1_Locus_769_Transcript_40/41_Confidence_0.320_Length_1479, vo_n1_Locus_6649_Transcript_1/3_Confidence_0.750_Length_1883, vo_c1_Locus_3603_Transcript_26/48_Confidence_0.400_Length_1432, vo_n1_Locus_5348_Transcript_2/2_Confidence_0.200_Length_1608, vo_n3_Locus_3383_Transcript_35/37_Confidence_0.389_Length_1512, tri_c_comp20802_c0_seq1

Butanoate metabolism	map00650	16	ec:2.6.1.19 - transaminase, ec:1.3.5.1 - dehydrogenase, ec:1.2.1.24 - dehydrogenase (NAD+), ec:2.2.1.6 - synthase, ec:5.1.2.3 - epimerase, ec:1.2.1.16 - dehydrogenase [NAD(P)+], ec:2.3.1.9 - C-acetyltransferase, ec:2.3.3.10 - synthase, ec:1.3.8.1 - acyl-CoA dehydrogenase, ec:2.3.1.54 - C-acetyltransferase, ec:2.8.3.8 - CoA-transferase, ec:2.8.3.5 - CoA-transferase, ec:4.2.1.17 - hydratase, ec:1.1.1.36 - reductase, ec:1.1.1.35 - dehydrogenase, ec:1.1.1.30 - dehydrogenase	2, 11, 1, 1, 2, 1, 2, 4, 2, 9, 1, 1, 7, 1, 16, 6	vo_c3_Locus_29526_Transcript_7/10_Confidence_0.268_Length_874, vo_c2_Locus_844_Transcript_5/40_Confidence_0.162_Length_2081, vo_n2_Locus_5500_Transcript_5/5_Confidence_0.308_Length_1268, vo_n2_Locus_5500_Transcript_1/5_Confidence_0.308_Length_1100, tri_n_comp12494_c0_seq1, vo_n2_Locus_5500_Transcript_2/5_Confidence_0.538_Length_1312, vo_n2_Locus_2182_Transcript_14/15_Confidence_0.458_Length_3129, vo_c2_Locus_642_Transcript_3/10_Confidence_0.571_Length_2141, vo_n1_Locus_1508_Transcript_4/6_Confidence_0.429_Length_1979, vo_n1_Locus_2259_Transcript_6/13_Confidence_0.429_Length_1275, vo_n1_Locus_2259_Transcript_9/13_Confidence_0.429_Length_1314, vo_c1_Locus_1462_Transcript_1/46_Confidence_0.182_Length_2129, vo_c1_Locus_1462_Transcript_8/46_Confidence_0.530_Length_9104, vo_c1_Locus_24159_Transcript_1/2_Confidence_0.333_Length_1573, tri_n_comp26150_c0_seq1, vo_n1_Locus_11137_Transcript_5/5_Confidence_0.143_Length_2829, vo_c3_Locus_9925_Transcript_8/11_Confidence_0.263_Length_3550, vo_c1_Locus_24159_Transcript_1/2_Confidence_0.333_Length_1573, vo_c1_Locus_2657_Transcript_1/1_Confidence_0.000_Length_1513, vo_n2_Locus_23058_Transcript_1/1_Confidence_0.000_Length_1342, vo_n2_Locus_634_Transcript_4/6_Confidence_0.357_Length_8557, tri_c_comp57035_c0_seq1, vo_c1_Locus_1809_Transcript_1/1_Confidence_0.000_Length_3087, vo_c3_Locus_18718_Transcript_1/1_Confidence_0.000_Length_2686, tri_c_comp7217_c0_seq2, vo_c3_Locus_18248_Transcript_1/2_Confidence_0.667_Length_1596, vo_c3_Locus_1564_Transcript_1/27_Confidence_0.474_Length_3491, vo_n2_Locus_4549_Transcript_18/24_Confidence_0.395_Length_3486, vo_c1_Locus_1748_Transcript_10/39_Confidence_0.197_Length_3498, vo_c1_Locus_1748_Transcript_19/39_Confidence_0.211_Length_3788, vo_c1_Locus_1748_Transcript_30/39_Confidence_0.282_Length_4645, vo_n2_Locus_4549_Transcript_11/24_Confidence_0.474_Length_3796, vo_n1_Locus_103_Transcript_17/28_Confidence_0.483_Length_3469, vo_n3_Locus_2375_Transcript_13/19_Confidence_0.467_Length_3465, vo_c3_Locus_1564_Transcript_22/27_Confidence_0.526_Length_3527, vo_c2_Locus_6864_Transcript_3/5_Confidence_0.667_Length_1701, vo_c2_Locus_6864_Transcript_3/5_Confidence_0.667_Length_1701, vo_c2_Locus_2261_Transcript_1/2_Confidence_1.000_Length_2564, vo_c3_Locus_5948_Transcript_1/1_Confidence_0.000_Length_1027, vo_n2_Locus_16945_Transcript_1/3_Confidence_0.500_Length_1084, vo_c3_Locus_9925_Transcript_8/11_Confidence_0.263_Length_3550, vo_n1_Locus_11137_Transcript_5/5_Confidence_0.143_Length_2829, vo_n2_Locus_16945_Transcript_3/3_Confidence_0.000_Length_746, vo_n2_Locus_30747_Transcript_1/1_Confidence_0.000_Length_951, vo_c1_Locus_2815_Transcript_3/4_Confidence_0.200_Length_2244, vo_c2_Locus_2261_Transcript_1/2_Confidence_1.000_Length_2564, vo_n1_Locus_9414_Transcript_8/13_Confidence_0.167_Length_606, vo_c1_Locus_40339_Transcript_1/1_Confidence_0.000_Length_1318, vo_c3_Locus_2031_Transcript_8/8_Confidence_0.111_Length_1389, vo_n1_Locus_11137_Transcript_5/5_Confidence_0.143_Length_2829, vo_c1_Locus_4675_Transcript_3/3_Confidence_0.200_Length_1348, vo_n2_Locus_30747_Transcript_1/1_Confidence_0.000_Length_951, vo_n2_Locus_636_Transcript_13/16_Confidence_0.222_Length_768, vo_c1_Locus_2815_Transcript_3/4_Confidence_0.200_Length_2244, vo_c3_Locus_9925_Transcript_8/11_Confidence_0.263_Length_3550, tri_n_comp42565_c0_seq1, vo_c2_Locus_189_Transcript_29/60_Confidence_0.015_Length_692, vo_n2_Locus_636_Transcript_16/16_Confidence_0.139_Length_616, vo_n2_Locus_636_Transcript_14/16_Confidence_0.222_Length_622, vo_n2_Locus_636_Transcript_5/16_Confidence_0.194_Length_591, vo_n2_Locus_636_Transcript_3/16_Confidence_0.306_Length_685, vo_c3_Locus_1735_Transcript_2/3_Confidence_0.333_Length_8270, vo_c2_Locus_9268_Transcript_2/5_Confidence_0.500_Length_8293, vo_n2_Locus_10545_Transcript_1/1_Confidence_0.000_Length_1731, vo_n2_Locus_12693_Transcript_1/1_Confidence_0.000_Length_2082, vo_n2_Locus_19729_Transcript_5/6_Confidence_0.500_Length_1106, vo_c1_Locus_1033_Transcript_1/1_Confidence_0.000_Length_1983

Pentose phosphate pathway	map00030	16	ec:2.7.1.90 - 1-phosphotransferase, ec:2.2.1.1 - glycolaldehydetransferase, ec:2.7.1.15 - deoxyribokinase, ec:2.7.1.11 - phosphohexokinase, ec:2.7.6.1 - diphosphokinase, ec:5.3.1.6 - isomerase, ec:5.3.1.9 - isomerase, ec:1.2.1.9 - dehydrogenase (NADP+), ec:4.1.2.13 - aldolase, ec:5.1.3.1 - 3-epimerase, ec:3.1.3.11 - hexose diphosphatase, ec:3.1.1.31 - phosphogluconolactonase, ec:1.1.1.44 - dehydrogenase (NADP+-dependent, decarboxylating), ec:1.1.1.49 - dehydrogenase (NADP+), ec:5.4.2.2 - (alpha-D-glucose-1,6-bisphosphate-dependent), ec:5.4.2.7 - phosphodeoxyribomutase	1, 24, 2, 5, 4, 2, 2, 9, 38, 3, 8, 2, 10, 6, 7, 1	vo_c2_Locus_6348_Transcript_1/2_Confidence_1.000_Length_3755, vo_c3_Locus_1314_Transcript_17/46_Confidence_0.365_Length_2530, vo_n3_Locus_387_Transcript_34/39_Confidence_0.386_Length_2514, vo_c2_Locus_924_Transcript_20/40_Confidence_0.452_Length_2520, vo_c2_Locus_924_Transcript_13/40_Confidence_0.310_Length_2559, vo_c3_Locus_1314_Transcript_33/46_Confidence_0.292_Length_2524, vo_c1_Locus_1204_Transcript_14/17_Confidence_0.394_Length_4476, vo_c2_Locus_924_Transcript_19/40_Confidence_0.333_Length_2562, vo_n1_Locus_327_Transcript_13/41_Confidence_0.275_Length_2480, vo_c3_Locus_1314_Transcript_6/46_Confidence_0.208_Length_1518, vo_n1_Locus_327_Transcript_40/41_Confidence_0.176_Length_2442, vo_c3_Locus_1314_Transcript_38/46_Confidence_0.302_Length_2522, vo_c2_Locus_924_Transcript_34/40_Confidence_0.369_Length_2618, vo_c3_Locus_1314_Transcript_14/46_Confidence_0.229_Length_3828, vo_c2_Locus_924_Transcript_16/40_Confidence_0.321_Length_2559, vo_c2_Locus_924_Transcript_23/40_Confidence_0.488_Length_2850, vo_c3_Locus_1314_Transcript_21/46_Confidence_0.365_Length_2524, vo_c1_Locus_1204_Transcript_13/17_Confidence_0.364_Length_4497, vo_n1_Locus_327_Transcript_24/41_Confidence_0.385_Length_2547, vo_c2_Locus_924_Transcript_17/40_Confidence_0.333_Length_2453, tri_c_comp20608_c0_seq4, tri_c_comp20608_c0_seq5, vo_c3_Locus_1314_Transcript_43/46_Confidence_0.198_Length_2519, vo_n2_Locus_2054_Transcript_26/37_Confidence_0.448_Length_2522, tri_c_comp20608_c0_seq7, vo_c1_Locus_36426_Transcript_3/5_Confidence_0.429_Length_2307, vo_c2_Locus_27773_Transcript_4/7_Confidence_0.545_Length_2350, vo_c3_Locus_1613_Transcript_6/17_Confidence_0.158_Length_5809, vo_n1_Locus_10193_Transcript_1/1_Confidence_0.000_Length_1647, vo_c2_Locus_6348_Transcript_1/2_Confidence_1.000_Length_3755, vo_n3_Locus_23556_Transcript_2/4_Confidence_0.500_Length_4210, tri_c_comp43537_c0_seq1, vo_c3_Locus_18398_Transcript_2/2_Confidence_0.000_Length_715, vo_n2_Locus_18825_Transcript_1/3_Confidence_0.500_Length_1632, tri_c_comp33638_c0_seq1, vo_n2_Locus_8943_Transcript_1/2_Confidence_0.750_Length_1351, vo_c1_Locus_34898_Transcript_1/1_Confidence_0.000_Length_1083, vo_n2_Locus_8097_Transcript_1/6_Confidence_0.500_Length_1196, vo_n2_Locus_11241_Transcript_1/3_Confidence_0.600_Length_3773, vo_c2_Locus_479_Transcript_1/2_Confidence_1.000_Length_2154, vo_c1_Locus_223_Transcript_52/65_Confidence_0.042_Length_799, vo_n2_Locus_13049_Transcript_20/20_Confidence_0.289_Length_1233, vo_c3_Locus_1015_Transcript_30/32_Confidence_0.181_Length_1172, vo_n2_Locus_13049_Transcript_19/20_Confidence_0.368_Length_1268, vo_n2_Locus_13049_Transcript_17/20_Confidence_0.342_Length_1329, vo_c3_Locus_1015_Transcript_9/32_Confidence_0.236_Length_1272, vo_n2_Locus_13049_Transcript_9/20_Confidence_0.316_Length_1224, vo_c3_Locus_1015_Transcript_13/32_Confidence_0.222_Length_1363, vo_n2_Locus_13049_Transcript_7/20_Confidence_0.421_Length_1354, vo_n1_Locus_565_Transcript_31/31_Confidence_0.178_Length_3922, vo_c3_Locus_2800_Transcript_3/23_Confidence_0.566_Length_1690, vo_c3_Locus_2800_Transcript_9/23_Confidence_0.585_Length_1396, tri_n_comp21653_c0_seq1, vo_n1_Locus_565_Transcript_5/31_Confidence_0.244_Length_1533, vo_n2_Locus_4661_Transcript_5/12_Confidence_0.208_Length_1240, vo_n1_Locus_565_Transcript_17/31_Confidence_0.422_Length_1454, vo_c3_Locus_2800_Transcript_5/23_Confidence_0.566_Length_2133, vo_c1_Locus_1757_Transcript_17/19_Confidence_0.375_Length_1367, vo_c1_Locus_298_Transcript_4/32_Confidence_0.255_Length_1585, vo_n1_Locus_8402_Transcript_3/4_Confidence_0.286_Length_1464, vo_c3_Locus_2800_Transcript_23/23_Confidence_0.151_Length_1466, vo_n2_Locus_903_Transcript_38/40_Confidence_0.222_Length_6612, vo_n1_Locus_565_Transcript_20/31_Confidence_0.444_Length_1622, vo_n2_Locus_2046_Transcript_7/18_Confidence_0.345_Length_1296, vo_c3_Locus_2800_Transcript_8/23_Confidence_0.585_Length_1344, vo_n3_Locus_1119_Transcript_19/50_Confidence_0.050_Length_498, vo_c2_Locus_5742_Transcript_10/21_Confidence_0.244_Length_1454, vo_n3_Locus_1119_Transcript_11/50_Confidence_0.124_Length_2839, vo_c2_Locus_5742_Transcript_18/21_Confidence_0.317_Length_2233, vo_n1_Locus_565_Transcript_13/31_Confidence_0.378_Length_1400, vo_c3_Locus_2800_Transcript_14/23_Confidence_0.321_Length_1426, vo_c3_Locus_614_Transcript_19/24_Confidence_0.394_Length_3443, vo_c2_Locus_358_Transcript_1/41_Confidence_0.080_Length_1708, vo_c2_Locus_5742_Transcript_14/21_Confidence_0.439_Length_2413, vo_c1_Locus_1757_Transcript_8/19_Confidence_0.458_Length_1460, vo_n1_Locus_986_Transcript_16/36_Confidence_0.211_Length_1811, vo_n1_Locus_565_Transcript_16/31_Confidence_0.400_Length_1499, vo_n2_Locus_903_Transcript_37/40_Confidence_0.346_Length_3926, tri_c_comp20312_c0_seq4, vo_c3_Locus_614_Transcript_13/24_Confidence_0.061_Length_4970, vo_n2_Locus_2046_Transcript_11/18_Confidence_0.310_Length_1666, vo_n2_Locus_903_Transcript_31/40_Confidence_0.321_Length_2251, vo_n1_Locus_565_Transcript_30/31_Confidence_0.422_Length_3814, vo_c3_Locus_614_Transcript_2/24_Confidence_0.485_Length_1777, vo_n1_Locus_5148_Transcript_1/4_Confidence_0.667_Length_1983, vo_c1_Locus_10454_Transcript_1/2_Confidence_0.750_Length_1441, vo_n3_Locus_1119_Transcript_18/50_Confidence_0.149_Length_1959, vo_n3_Locus_1573_Transcript_4/9_Confidence_0.364_Length_1074, vo_n2_Locus_3298_Transcript_6/11_Confidence_0.333_Length_1072, vo_n1_Locus_7783_Transcript_1/2_Confidence_0.667_Length_900, vo_c2_Locus_40594_Transcript_2/3_Confidence_0.500_Length_1728, vo_n2_Locus_39295_Transcript_2/2_Confidence_0.500_Length_1939, vo_c2_Locus_6460_Transcript_1/2_Confidence_1.000_Length_1563, vo_n1_Locus_3261_Transcript_3/4_Confidence_0.667_Length_1633, vo_n3_Locus_27_Transcript_1/1_Confidence_0.000_Length_1278, tri_c_comp23875_c0_seq1, tri_c_comp24147_c0_seq1, vo_c1_Locus_4576_Transcript_2/2_Confidence_0.000_Length_1644, vo_n2_Locus_1545_Transcript_1/1_Confidence_0.000_Length_1374, vo_c1_Locus_37991_Transcript_1/1_Confidence_0.000_Length_924, tri_n_comp18543_c0_seq8, vo_c2_Locus_14292_Transcript_1/3_Confidence_0.857_Length_1370, vo_n2_Locus_25571_Transcript_1/1_Confidence_0.000_Length_1103, tri_n_comp18543_c0_seq4, vo_c2_Locus_13495_Transcript_4/17_Confidence_0.346_Length_1808, tri_c_comp25671_c0_seq1, tri_n_comp49842_c0_seq1, vo_n2_Locus_6054_Transcript_2/13_Confidence_0.556_Length_2470, vo_c2_Locus_14292_Transcript_3/3_Confidence_0.429_Length_1343, vo_c3_Locus_5223_Transcript_5/7_Confidence_0.444_Length_5924, vo_c1_Locus_22469_Transcript_1/1_Confidence_0.000_Length_2691, vo_c1_Locus_2369_Transcript_1/3_Confidence_0.667_Length_1916, vo_n2_Locus_1545_Transcript_1/1_Confidence_0.000_Length_1374, vo_c1_Locus_2369_Transcript_2/3_Confidence_0.333_Length_2032, vo_c3_Locus_3736_Transcript_2/6_Confidence_0.571_Length_1920,

					vo_c3_Locus_3736_Transcript_4/6_Confidence_0.286_Length_917, tri_n_comp18813_c0_seq13, vo_c2_Locus_23037_Transcript_2/2_Confidence_0.000_Length_2109, vo_n3_Locus_362_Transcript_3/4_Confidence_0.364_Length_3895, vo_n3_Locus_362_Transcript_1/4_Confidence_0.727_Length_3306, vo_n1_Locus_5208_Transcript_2/2_Confidence_0.000_Length_2077, vo_c2_Locus_23037_Transcript_1/2_Confidence_0.750_Length_1965, vo_n3_Locus_15336_Transcript_1/1_Confidence_0.000_Length_1810, vo_c2_Locus_23037_Transcript_1/2_Confidence_0.750_Length_1965

Citrate cycle (TCA cycle)	map00020	16	ec:6.4.1.1 - carboxylase, ec:4.1.1.49 - carboxykinase (ATP), ec:1.3.5.1 - dehydrogenase, ec:4.1.1.32 - carboxykinase (GTP), ec:6.2.1.5 - ligase (ADP-forming), ec:6.2.1.4 - ligase (GDP-forming), ec:1.8.1.4 - dehydrogenase, ec:1.1.5.4 - dehydrogenase (quinone), ec:2.3.3.8 - citrate synthase, ec:2.3.3.1 - (Si)-synthase, ec:2.3.1.61 - succinyltransferase, ec:4.2.1.3 - hydratase, ec:4.2.1.2 - hydratase, ec:1.1.1.37 - dehydrogenase, ec:1.1.1.42 - dehydrogenase (NADP+), ec:1.2.4.2 - dehydrogenase (succinyl-transferring)	1, 5, 11, 5, 28, 6, 2, 3, 31, 11, 3, 8, 2, 24, 10, 2	vo_n2_Locus_15097_Transcript_1/1_Confidence_0.000_Length_3935, vo_c1_Locus_2295_Transcript_9/10_Confidence_0.238_Length_2213, vo_c2_Locus_2862_Transcript_9/10_Confidence_0.250_Length_2209, vo_c1_Locus_2295_Transcript_3/10_Confidence_0.524_Length_2330, vo_n2_Locus_1690_Transcript_8/11_Confidence_0.429_Length_2234, vo_n2_Locus_1690_Transcript_9/11_Confidence_0.429_Length_2277, vo_n2_Locus_5500_Transcript_5/5_Confidence_0.308_Length_1268, vo_n2_Locus_5500_Transcript_1/5_Confidence_0.308_Length_1100, tri_n_comp12494_c0_seq1, vo_n2_Locus_5500_Transcript_2/5_Confidence_0.538_Length_1312, vo_n2_Locus_2182_Transcript_14/15_Confidence_0.458_Length_3129, vo_c2_Locus_642_Transcript_3/10_Confidence_0.571_Length_2141, vo_n1_Locus_1508_Transcript_4/6_Confidence_0.429_Length_1979, vo_n1_Locus_2259_Transcript_6/13_Confidence_0.429_Length_1275, vo_n1_Locus_2259_Transcript_9/13_Confidence_0.429_Length_1314, vo_c1_Locus_1462_Transcript_1/46_Confidence_0.182_Length_2129, vo_c1_Locus_1462_Transcript_8/46_Confidence_0.530_Length_9104, vo_c1_Locus_2295_Transcript_9/10_Confidence_0.238_Length_2213, vo_c2_Locus_2862_Transcript_9/10_Confidence_0.250_Length_2209, vo_c1_Locus_2295_Transcript_3/10_Confidence_0.524_Length_2330, vo_n2_Locus_1690_Transcript_8/11_Confidence_0.429_Length_2234, vo_n2_Locus_1690_Transcript_9/11_Confidence_0.429_Length_2277, vo_c1_Locus_2207_Transcript_20/51_Confidence_0.393_Length_3556, vo_c3_Locus_2283_Transcript_28/38_Confidence_0.331_Length_3501, vo_n3_Locus_1164_Transcript_30/46_Confidence_0.400_Length_3745, tri_c_comp20184_c0_seq2, vo_c2_Locus_7270_Transcript_1/1_Confidence_0.500_Length_1488, vo_c1_Locus_31_Transcript_859/1155_Confidence_0.002_Length_1257, vo_n3_Locus_1164_Transcript_32/46_Confidence_0.456_Length_3745, vo_n1_Locus_3448_Transcript_46/64_Confidence_0.085_Length_783, vo_c2_Locus_644_Transcript_12/40_Confidence_0.459_Length_3665, vo_n3_Locus_1164_Transcript_35/46_Confidence_0.511_Length_3745, vo_c3_Locus_5_Transcript_229/353_Confidence_0.004_Length_952, vo_c2_Locus_644_Transcript_3/40_Confidence_0.071_Length_957, vo_n1_Locus_3448_Transcript_53/64_Confidence_0.225_Length_3567, vo_n2_Locus_386_Transcript_32/33_Confidence_0.208_Length_3664, vo_n1_Locus_3448_Transcript_63/64_Confidence_0.318_Length_3637, vo_n3_Locus_1164_Transcript_33/46_Confidence_0.456_Length_3748, vo_c3_Locus_2283_Transcript_26/38_Confidence_0.285_Length_3501, vo_n2_Locus_386_Transcript_22/33_Confidence_0.376_Length_3568, vo_c3_Locus_2283_Transcript_29/38_Confidence_0.292_Length_3501, vo_n2_Locus_386_Transcript_1/33_Confidence_0.139_Length_3600, vo_c2_Locus_644_Transcript_24/40_Confidence_0.398_Length_3511, vo_n3_Locus_1164_Transcript_43/46_Confidence_0.389_Length_3338, vo_c1_Locus_2207_Transcript_39/51_Confidence_0.250_Length_3586, vo_c3_Locus_79_Transcript_1/3_Confidence_0.200_Length_5222, vo_c2_Locus_52_Transcript_234/466_Confidence_0.003_Length_1462, vo_n2_Locus_386_Transcript_10/33_Confidence_0.376_Length_3676, vo_n1_Locus_1394_Transcript_2/3_Confidence_0.400_Length_2232, tri_n_comp19507_c0_seq6, vo_c3_Locus_79_Transcript_1/3_Confidence_0.200_Length_5222, vo_n1_Locus_1394_Transcript_2/3_Confidence_0.400_Length_2232, vo_n2_Locus_2182_Transcript_14/15_Confidence_0.458_Length_3129, vo_c1_Locus_1462_Transcript_1/46_Confidence_0.182_Length_2129, vo_c1_Locus_1462_Transcript_8/46_Confidence_0.530_Length_9104, vo_c2_Locus_642_Transcript_3/10_Confidence_0.571_Length_2141, vo_n3_Locus_6810_Transcript_2/3_Confidence_0.913_Length_1699, vo_n1_Locus_6460_Transcript_1/1_Confidence_0.000_Length_1912, vo_n1_Locus_833_Transcript_2/6_Confidence_0.333_Length_2200, vo_n2_Locus_3042_Transcript_2/3_Confidence_0.750_Length_2030, vo_c1_Locus_6244_Transcript_1/2_Confidence_0.750_Length_1731, vo_c1_Locus_2207_Transcript_20/51_Confidence_0.393_Length_3556, vo_c3_Locus_2283_Transcript_28/38_Confidence_0.331_Length_3501, vo_n3_Locus_1164_Transcript_30/46_Confidence_0.400_Length_3745, tri_c_comp20184_c0_seq2, vo_n3_Locus_1164_Transcript_32/46_Confidence_0.456_Length_3745, vo_n1_Locus_3448_Transcript_1/64_Confidence_0.062_Length_478, vo_n1_Locus_3448_Transcript_46/64_Confidence_0.085_Length_783, vo_c2_Locus_644_Transcript_12/40_Confidence_0.459_Length_3665, vo_n3_Locus_1164_Transcript_35/46_Confidence_0.511_Length_3745, vo_c2_Locus_644_Transcript_3/40_Confidence_0.071_Length_957, vo_n1_Locus_3448_Transcript_53/64_Confidence_0.225_Length_3567, vo_n2_Locus_386_Transcript_32/33_Confidence_0.208_Length_3664, vo_n1_Locus_3448_Transcript_63/64_Confidence_0.318_Length_3637, vo_n3_Locus_1164_Transcript_33/46_Confidence_0.456_Length_3748, vo_c3_Locus_2283_Transcript_26/38_Confidence_0.285_Length_3501, vo_n2_Locus_386_Transcript_22/33_Confidence_0.376_Length_3568, vo_n1_Locus_3448_Transcript_2/64_Confidence_0.054_Length_402, vo_c3_Locus_2283_Transcript_29/38_Confidence_0.292_Length_3501, vo_c3_Locus_2283_Transcript_35/38_Confidence_0.400_Length_3582, vo_n2_Locus_386_Transcript_1/33_Confidence_0.139_Length_3600, vo_c2_Locus_644_Transcript_24/40_Confidence_0.398_Length_3511, vo_n3_Locus_1164_Transcript_43/46_Confidence_0.389_Length_3338, vo_c1_Locus_2207_Transcript_39/51_Confidence_0.250_Length_3586, vo_c3_Locus_79_Transcript_1/3_Confidence_0.200_Length_5222, vo_n2_Locus_386_Transcript_10/33_Confidence_0.376_Length_3676, vo_n1_Locus_1394_Transcript_2/3_Confidence_0.400_Length_2232, vo_c2_Locus_644_Transcript_6/40_Confidence_0.204_Length_3616, vo_c1_Locus_2207_Transcript_36/51_Confidence_0.402_Length_3407, vo_n2_Locus_386_Transcript_5/33_Confidence_0.040_Length_566, tri_n_comp19507_c0_seq6, vo_n1_Locus_3448_Transcript_55/64_Confidence_0.233_Length_3564, vo_c3_Locus_4005_Transcript_5/6_Confidence_0.167_Length_1517, vo_c1_Locus_6811_Transcript_7/8_Confidence_0.364_Length_589, vo_n2_Locus_297_Transcript_2/5_Confidence_0.636_Length_1679, vo_c2_Locus_1080_Transcript_4/21_Confidence_0.241_Length_1741, vo_c2_Locus_1080_Transcript_3/21_Confidence_0.207_Length_1702, vo_n3_Locus_1253_Transcript_1/2_Confidence_0.500_Length_1626, vo_c2_Locus_1080_Transcript_8/21_Confidence_0.138_Length_1077, vo_c2_Locus_4581_Transcript_3/8_Confidence_0.684_Length_1578, vo_n2_Locus_901_Transcript_1/95_Confidence_0.023_Length_1512, vo_n2_Locus_20307_Transcript_6/7_Confidence_0.062_Length_2030, vo_n3_Locus_1253_Transcript_2/2_Confidence_0.000_Length_1587, vo_n2_Locus_3821_Transcript_2/4_Confidence_0.750_Length_1813, tri_c_comp16609_c0_seq3, vo_c3_Locus_278_Transcript_151/322_Confidence_0.010_Length_2531, tri_n_comp15079_c0_seq3, vo_c1_Locus_2380_Transcript_17/17_Confidence_0.086_Length_3951, vo_c2_Locus_191_Transcript_2/2_Confidence_0.167_Length_3106, vo_c1_Locus_2380_Transcript_3/17_Confidence_0.371_Length_12857, tri_c_comp21028_c0_seq2, tri_c_comp21028_c0_seq3, vo_c2_Locus_354_Transcript_3/17_Confidence_0.667_Length_4966, vo_n1_Locus_30469_Transcript_2/2_Confidence_0.000_Length_979,

					tri_n_comp8247_c0_seq2, vo_c3_Locus_1133_Transcript_4/9_Confidence_0.455_Length_1666, vo_c3_Locus_1723_Transcript_29/37_Confidence_0.411_Length_1695, vo_n2_Locus_330_Transcript_25/38_Confidence_0.361_Length_1516, vo_c3_Locus_1723_Transcript_35/37_Confidence_0.464_Length_1702, vo_c1_Locus_15206_Transcript_3/3_Confidence_0.000_Length_1312, vo_c1_Locus_7371_Transcript_4/4_Confidence_0.600_Length_1352, vo_c3_Locus_1723_Transcript_37/37_Confidence_0.214_Length_1530, vo_n1_Locus_769_Transcript_28/41_Confidence_0.400_Length_1609, vo_n1_Locus_769_Transcript_9/41_Confidence_0.460_Length_1494, vo_c3_Locus_2965_Transcript_2/2_Confidence_0.250_Length_1327, vo_n2_Locus_330_Transcript_5/38_Confidence_0.328_Length_11715, vo_n1_Locus_9772_Transcript_1/2_Confidence_0.333_Length_1224, vo_c1_Locus_3603_Transcript_44/48_Confidence_0.492_Length_1696, vo_c2_Locus_4242_Transcript_2/3_Confidence_0.400_Length_1168, vo_n1_Locus_6649_Transcript_2/3_Confidence_0.500_Length_2124, vo_c1_Locus_7371_Transcript_1/4_Confidence_0.500_Length_1352, vo_n3_Locus_2381_Transcript_2/3_Confidence_0.500_Length_1330, vo_c3_Locus_1723_Transcript_16/37_Confidence_0.286_Length_1491, vo_n1_Locus_769_Transcript_36/41_Confidence_0.420_Length_1536, vo_n1_Locus_769_Transcript_40/41_Confidence_0.320_Length_1479, vo_n1_Locus_6649_Transcript_1/3_Confidence_0.750_Length_1883, vo_c1_Locus_3603_Transcript_26/48_Confidence_0.400_Length_1432, vo_n1_Locus_5348_Transcript_2/2_Confidence_0.200_Length_1608, vo_n3_Locus_3383_Transcript_35/37_Confidence_0.389_Length_1512, tri_c_comp20802_c0_seq1, vo_c3_Locus_6304_Transcript_1/17_Confidence_0.159_Length_2915, vo_n2_Locus_353_Transcript_7/18_Confidence_0.420_Length_2960, vo_c1_Locus_5705_Transcript_18/19_Confidence_0.191_Length_3822, vo_n2_Locus_353_Transcript_14/18_Confidence_0.280_Length_2879, vo_n1_Locus_2903_Transcript_2/3_Confidence_0.400_Length_1386, vo_n1_Locus_464_Transcript_11/22_Confidence_0.362_Length_3670, vo_c2_Locus_11026_Transcript_2/2_Confidence_0.000_Length_1965, vo_c3_Locus_6304_Transcript_5/17_Confidence_0.477_Length_2995, vo_c1_Locus_5705_Transcript_16/19_Confidence_0.489_Length_4193, vo_c3_Locus_6304_Transcript_2/17_Confidence_0.523_Length_3712, vo_n2_Locus_3831_Transcript_3/5_Confidence_0.286_Length_3559, vo_c2_Locus_13441_Transcript_1/2_Confidence_0.333_Length_3397

Glutathione metabolism	map00480	16	ec:2.5.1.16 - synthase, ec:2.5.1.18 - transferase, ec:2.5.1.22 - synthase, ec:4.1.1.17 - decarboxylase, ec:1.8.1.7 - reductase, ec:1.11.1.9 - peroxidase, ec:1.17.4.1 - reductase, ec:6.3.2.3 - synthase, ec:6.3.2.2 - ligase, ec:1.8.5.1 - dehydrogenase (ascorbate), ec:1.1.1.44 - dehydrogenase (NADP+-dependent, decarboxylating), ec:1.1.1.42 - dehydrogenase (NADP+), ec:2.3.2.2 - glutamyl transpeptidase, ec:1.1.1.49 - dehydrogenase (NADP+), ec:1.11.1.12 - glutathione peroxidase, ec:1.11.1.15 - thioredoxin peroxidase	1, 11, 2, 1, 18, 13, 32, 1, 5, 1, 10, 10, 1, 6, 1, 14	vo_c2_Locus_25147_Transcript_1/2_Confidence_0.333_Length_1097, vo_c3_Locus_7919_Transcript_7/13_Confidence_0.524_Length_1057, vo_c3_Locus_483_Transcript_11/12_Confidence_0.217_Length_759, vo_c2_Locus_8227_Transcript_3/3_Confidence_0.143_Length_1106, vo_c2_Locus_8227_Transcript_2/3_Confidence_0.571_Length_1060, vo_n2_Locus_34681_Transcript_5/6_Confidence_0.077_Length_2256, vo_c1_Locus_15275_Transcript_1/2_Confidence_0.333_Length_968, vo_n2_Locus_8358_Transcript_1/8_Confidence_0.444_Length_1513, vo_n2_Locus_2232_Transcript_6/7_Confidence_0.455_Length_2038, vo_c2_Locus_1790_Transcript_5/10_Confidence_0.474_Length_1047, vo_n2_Locus_8358_Transcript_6/8_Confidence_0.389_Length_1051, tri_n_comp38105_c0_seq1, vo_c2_Locus_25147_Transcript_1/2_Confidence_0.333_Length_1097, tri_c_comp23701_c0_seq1, vo_n1_Locus_24_Transcript_2/4_Confidence_0.667_Length_1878, vo_n2_Locus_10545_Transcript_1/1_Confidence_0.000_Length_1731, vo_c2_Locus_5797_Transcript_1/2_Confidence_0.400_Length_1712, vo_n1_Locus_23402_Transcript_3/3_Confidence_0.000_Length_1071, vo_c2_Locus_21654_Transcript_2/4_Confidence_0.500_Length_1715, vo_n3_Locus_1380_Transcript_5/6_Confidence_0.273_Length_1217, vo_n3_Locus_4780_Transcript_1/1_Confidence_0.000_Length_1276, vo_c2_Locus_6794_Transcript_18/19_Confidence_0.031_Length_2437, vo_n2_Locus_12693_Transcript_1/1_Confidence_0.000_Length_2082, vo_c1_Locus_8243_Transcript_1/1_Confidence_0.000_Length_849, vo_n1_Locus_471_Transcript_16/17_Confidence_0.065_Length_2412, tri_n_comp41310_c0_seq1, vo_c3_Locus_1735_Transcript_2/3_Confidence_0.333_Length_8270, vo_n2_Locus_3_Transcript_23780/24291_Confidence_0.000_Length_2094, vo_c2_Locus_9268_Transcript_2/5_Confidence_0.500_Length_8293, vo_n2_Locus_6286_Transcript_1/1_Confidence_0.000_Length_1634, vo_c2_Locus_2829_Transcript_4/21_Confidence_0.026_Length_813, vo_n1_Locus_471_Transcript_9/17_Confidence_0.387_Length_2658, vo_c1_Locus_1033_Transcript_1/1_Confidence_0.000_Length_1983, vo_n2_Locus_1609_Transcript_3/3_Confidence_0.000_Length_489, vo_n3_Locus_21278_Transcript_2/2_Confidence_0.250_Length_899, vo_c3_Locus_7729_Transcript_4/5_Confidence_0.167_Length_5654, vo_c1_Locus_15275_Transcript_1/2_Confidence_0.333_Length_968, vo_n2_Locus_23060_Transcript_2/2_Confidence_0.000_Length_889, vo_n1_Locus_4880_Transcript_4/6_Confidence_0.333_Length_1109, vo_n1_Locus_4880_Transcript_2/6_Confidence_0.333_Length_883, vo_n2_Locus_3013_Transcript_1/2_Confidence_0.750_Length_964, vo_n2_Locus_34681_Transcript_5/6_Confidence_0.077_Length_2256, vo_c3_Locus_26080_Transcript_2/2_Confidence_0.250_Length_928, vo_c2_Locus_5609_Transcript_4/4_Confidence_0.250_Length_1105, vo_c3_Locus_17900_Transcript_1/1_Confidence_0.000_Length_966, tri_c_comp22021_c0_seq1, vo_c3_Locus_2584_Transcript_3/5_Confidence_0.727_Length_2680, vo_c3_Locus_2584_Transcript_5/5_Confidence_0.500_Length_3685, vo_c1_Locus_2715_Transcript_9/14_Confidence_0.429_Length_1359, vo_c1_Locus_2066_Transcript_3/17_Confidence_0.415_Length_3989, vo_c3_Locus_4226_Transcript_1/2_Confidence_1.000_Length_2714, vo_c1_Locus_10489_Transcript_1/1_Confidence_0.000_Length_1447, vo_c2_Locus_80_Transcript_8/10_Confidence_0.310_Length_2635, vo_c1_Locus_2066_Transcript_16/17_Confidence_0.293_Length_2774, vo_n1_Locus_4390_Transcript_2/5_Confidence_0.333_Length_2677, vo_n1_Locus_4390_Transcript_3/5_Confidence_0.600_Length_2659, vo_n2_Locus_2860_Transcript_3/6_Confidence_0.250_Length_3111, vo_n1_Locus_2992_Transcript_5/14_Confidence_0.476_Length_1500, vo_n2_Locus_294_Transcript_20/20_Confidence_0.167_Length_2119, vo_c3_Locus_3000_Transcript_12/15_Confidence_0.407_Length_1288, vo_c2_Locus_5247_Transcript_3/7_Confidence_0.500_Length_1443, vo_n1_Locus_357_Transcript_3/6_Confidence_0.429_Length_2710, vo_c2_Locus_5247_Transcript_6/7_Confidence_0.600_Length_1453, vo_c2_Locus_80_Transcript_10/10_Confidence_0.207_Length_2759, vo_c3_Locus_3000_Transcript_6/15_Confidence_0.556_Length_1355, vo_n1_Locus_1856_Transcript_4/7_Confidence_0.462_Length_1396, vo_c2_Locus_5247_Transcript_5/7_Confidence_0.300_Length_1303, vo_c2_Locus_5247_Transcript_4/7_Confidence_0.400_Length_1501, vo_c1_Locus_2715_Transcript_6/14_Confidence_0.429_Length_1383, vo_c3_Locus_3737_Transcript_1/1_Confidence_0.500_Length_2762, vo_c2_Locus_5247_Transcript_7/7_Confidence_0.100_Length_1511, tri_c_comp13375_c0_seq4, vo_c1_Locus_1353_Transcript_2/5_Confidence_0.600_Length_1352, vo_c3_Locus_3000_Transcript_7/15_Confidence_0.259_Length_1522, vo_c2_Locus_6282_Transcript_1/2_Confidence_0.857_Length_2758, vo_n2_Locus_2860_Transcript_4/6_Confidence_0.583_Length_4084, tri_c_comp13375_c0_seq3, vo_n1_Locus_2992_Transcript_6/14_Confidence_0.476_Length_1522, vo_c1_Locus_8043_Transcript_1/1_Confidence_0.000_Length_1747, vo_n2_Locus_20981_Transcript_1/1_Confidence_0.000_Length_2435, vo_n2_Locus_5430_Transcript_2/3_Confidence_0.400_Length_1394, tri_n_comp34788_c0_seq1, vo_c1_Locus_1493_Transcript_3/4_Confidence_0.286_Length_2721, vo_n3_Locus_29456_Transcript_1/1_Confidence_0.000_Length_1949, tri_n_comp19474_c0_seq4, tri_n_comp18543_c0_seq8, vo_c2_Locus_14292_Transcript_1/3_Confidence_0.857_Length_1370, vo_n2_Locus_25571_Transcript_1/1_Confidence_0.000_Length_1103, tri_n_comp18543_c0_seq4, vo_c2_Locus_13495_Transcript_4/17_Confidence_0.346_Length_1808, tri_c_comp25671_c0_seq1, tri_n_comp49842_c0_seq1, vo_n2_Locus_6054_Transcript_2/13_Confidence_0.556_Length_2470, vo_c2_Locus_14292_Transcript_3/3_Confidence_0.429_Length_1343, vo_c3_Locus_5223_Transcript_5/7_Confidence_0.444_Length_5924, vo_c3_Locus_6304_Transcript_1/17_Confidence_0.159_Length_2915, vo_n2_Locus_353_Transcript_7/18_Confidence_0.420_Length_2960, vo_c1_Locus_5705_Transcript_18/19_Confidence_0.191_Length_3822, vo_n2_Locus_353_Transcript_14/18_Confidence_0.280_Length_2879, vo_n1_Locus_2903_Transcript_2/3_Confidence_0.400_Length_1386, vo_n1_Locus_464_Transcript_11/22_Confidence_0.362_Length_3670, vo_c2_Locus_11026_Transcript_2/2_Confidence_0.000_Length_1965, vo_c3_Locus_6304_Transcript_5/17_Confidence_0.477_Length_2995, vo_c1_Locus_5705_Transcript_16/19_Confidence_0.489_Length_4193, vo_c3_Locus_6304_Transcript_2/17_Confidence_0.523_Length_3712, vo_n2_Locus_15602_Transcript_2/2_Confidence_0.250_Length_2162, vo_c1_Locus_22469_Transcript_1/1_Confidence_0.000_Length_2691, vo_c1_Locus_2369_Transcript_1/3_Confidence_0.667_Length_1916, vo_n2_Locus_1545_Transcript_1/1_Confidence_0.000_Length_1374, vo_c1_Locus_2369_Transcript_2/3_Confidence_0.333_Length_2032, vo_c3_Locus_3736_Transcript_2/6_Confidence_0.571_Length_1920, vo_c3_Locus_3736_Transcript_4/6_Confidence_0.286_Length_917, vo_c3_Locus_26080_Transcript_2/2_Confidence_0.250_Length_928, vo_n1_Locus_30628_Transcript_1/2_Confidence_0.667_Length_554,

					vo_c3_Locus_7729_Transcript_4/5_Confidence_0.167_Length_5654, vo_n2_Locus_23060_Transcript_2/2_Confidence_0.000_Length_889, vo_n1_Locus_4880_Transcript_4/6_Confidence_0.333_Length_1109, vo_n1_Locus_4880_Transcript_2/6_Confidence_0.333_Length_883, vo_n2_Locus_3013_Transcript_1/2_Confidence_0.750_Length_964, vo_n3_Locus_2602_Transcript_1/2_Confidence_0.750_Length_664, tri_c_comp52750_c0_seq1, vo_n2_Locus_3_Transcript_19366/24291_Confidence_0.000_Length_2154, vo_c2_Locus_5609_Transcript_4/4_Confidence_0.250_Length_1105, vo_c3_Locus_17900_Transcript_1/1_Confidence_0.000_Length_966, vo_n3_Locus_15275_Transcript_1/5_Confidence_0.300_Length_3763, vo_c3_Locus_11858_Transcript_3/3_Confidence_0.000_Length_503, tri_c_comp22021_c0_seq1

Phenylalanine, tyrosine and tryptophan biosynthesis	map00400	16	ec:4.1.1.48 - synthase, ec:4.2.1.51 - dehydratase, ec:2.5.1.19 - 1-carboxyvinyltransferase, ec:2.6.1.78 - aminotransferase, ec:2.6.1.79 - aminotransferase, ec:4.2.1.91 - dehydratase, ec:2.6.1.1 - transaminase, ec:1.3.1.13 - dehydrogenase (NADP+), ec:1.3.1.12 - dehydrogenase, ec:1.14.16.1 - 4-monooxygenase, ec:4.2.3.4 - synthase, ec:5.4.99.5 - mutase, ec:4.2.1.20 - synthase, ec:4.2.1.10 - dehydratase, ec:2.5.1.54 - synthase, ec:5.3.1.24 - isomerase	1, 1, 1, 2, 2, 1, 4, 1, 1, 8, 5, 1, 2, 2, 1, 1	vo_c3_Locus_25814_Transcript_1/1_Confidence_0.000_Length_1912, vo_c2_Locus_15226_Transcript_2/2_Confidence_0.500_Length_2141, tri_c_comp32056_c0_seq1, tri_n_comp27683_c0_seq1, vo_n1_Locus_3423_Transcript_3/4_Confidence_0.250_Length_1416, tri_n_comp27683_c0_seq1, vo_n1_Locus_3423_Transcript_3/4_Confidence_0.250_Length_1416, vo_c2_Locus_15226_Transcript_2/2_Confidence_0.500_Length_2141, vo_n1_Locus_3423_Transcript_2/4_Confidence_0.250_Length_1347, tri_n_comp27683_c0_seq1, vo_c1_Locus_3127_Transcript_1/1_Confidence_0.000_Length_1478, vo_n1_Locus_3423_Transcript_3/4_Confidence_0.250_Length_1416, vo_c2_Locus_15226_Transcript_2/2_Confidence_0.500_Length_2141, vo_c2_Locus_15226_Transcript_2/2_Confidence_0.500_Length_2141, vo_c2_Locus_16025_Transcript_2/3_Confidence_0.400_Length_2217, vo_c3_Locus_4625_Transcript_6/11_Confidence_0.650_Length_2945, vo_c2_Locus_12196_Transcript_8/8_Confidence_0.133_Length_3092, vo_n1_Locus_3143_Transcript_9/11_Confidence_0.350_Length_2900, vo_c3_Locus_4625_Transcript_2/11_Confidence_0.350_Length_974, vo_c3_Locus_4625_Transcript_1/11_Confidence_0.350_Length_912, vo_c3_Locus_4625_Transcript_4/11_Confidence_0.650_Length_3056, vo_c3_Locus_4625_Transcript_3/11_Confidence_0.300_Length_1085, vo_n1_Locus_17511_Transcript_1/1_Confidence_0.000_Length_1342, vo_c1_Locus_219_Transcript_18/20_Confidence_0.373_Length_2065, vo_c3_Locus_2261_Transcript_20/33_Confidence_0.517_Length_1984, vo_c3_Locus_2261_Transcript_15/33_Confidence_0.500_Length_2011, vo_n1_Locus_14156_Transcript_1/1_Confidence_0.000_Length_1313, vo_n2_Locus_10254_Transcript_1/1_Confidence_0.000_Length_1125, tri_c_comp44055_c0_seq1, vo_c1_Locus_8760_Transcript_1/1_Confidence_0.000_Length_2415, vo_n1_Locus_29816_Transcript_1/1_Confidence_0.000_Length_650, vo_n1_Locus_5622_Transcript_1/3_Confidence_0.600_Length_1895, vo_n2_Locus_2223_Transcript_2/2_Confidence_0.250_Length_1508, vo_c3_Locus_25814_Transcript_1/1_Confidence_0.000_Length_1912

Glycerophospholipid metabolism	map00564	16	ec:2.7.1.107 - kinase (ATP), ec:2.7.1.32 - kinase, ec:4.1.1.65 - decarboxylase, ec:2.7.8.2 - cholinephosphotransferase, ec:2.7.8.5 - 1-phosphatidyltransferase, ec:3.1.1.4 - A2, ec:3.1.1.5 - lecithinase B, ec:1.1.1.8 - dehydrogenase (NAD+), ec:2.7.7.41 - cytidylyltransferase, ec:2.7.7.14 - cytidylyltransferase, ec:2.7.7.15 - cytidylyltransferase, ec:1.1.5.3 - dehydrogenase, ec:3.1.3.75 - phosphatase, ec:2.3.1.51 - O-acyltransferase, ec:2.1.1.103 - N-methyltransferase, ec:3.1.4.4 - D	9, 7, 2, 1, 3, 59, 5, 6, 1, 3, 4, 6, 1, 2, 1, 13	tri_n_comp47452_c0_seq1, vo_c3_Locus_6073_Transcript_2/9_Confidence_0.467_Length_1975, vo_c2_Locus_6531_Transcript_1/1_Confidence_0.000_Length_1860, tri_c_comp37156_c0_seq1, vo_c1_Locus_17180_Transcript_1/1_Confidence_0.000_Length_4157, vo_c2_Locus_11596_Transcript_2/3_Confidence_0.167_Length_1499, vo_n2_Locus_6369_Transcript_1/1_Confidence_0.000_Length_1901, vo_n2_Locus_8637_Transcript_3/3_Confidence_0.000_Length_1561, vo_c3_Locus_6073_Transcript_3/9_Confidence_0.400_Length_2122, vo_c1_Locus_10562_Transcript_1/3_Confidence_0.600_Length_1690, vo_n2_Locus_10323_Transcript_2/2_Confidence_0.000_Length_2787, vo_n2_Locus_7555_Transcript_15/18_Confidence_0.436_Length_3023, vo_n3_Locus_18_Transcript_1/1_Confidence_0.000_Length_1596, vo_n2_Locus_6874_Transcript_1/1_Confidence_0.000_Length_1254, vo_n3_Locus_17698_Transcript_1/1_Confidence_0.000_Length_1625, vo_c1_Locus_10067_Transcript_5/6_Confidence_0.417_Length_1561, vo_n2_Locus_17534_Transcript_2/2_Confidence_0.000_Length_1917, vo_n1_Locus_3423_Transcript_3/4_Confidence_0.250_Length_1416, vo_n2_Locus_32954_Transcript_1/2_Confidence_0.333_Length_1192, vo_c3_Locus_505_Transcript_1/2_Confidence_0.333_Length_960, vo_n2_Locus_3_Transcript_18744/24291_Confidence_0.000_Length_992, vo_n2_Locus_3_Transcript_18745/24291_Confidence_0.000_Length_1071, vo_n2_Locus_311_Transcript_71/114_Confidence_0.150_Length_1045, vo_c3_Locus_7729_Transcript_4/5_Confidence_0.167_Length_5654, vo_n1_Locus_443_Transcript_30/53_Confidence_0.193_Length_928, vo_c1_Locus_430_Transcript_20/51_Confidence_0.291_Length_976, vo_c2_Locus_601_Transcript_31/36_Confidence_0.229_Length_1002, vo_c2_Locus_5092_Transcript_6/14_Confidence_0.476_Length_1090, vo_c3_Locus_1328_Transcript_24/26_Confidence_0.245_Length_1398, vo_n2_Locus_311_Transcript_78/114_Confidence_0.093_Length_844, vo_c3_Locus_1273_Transcript_51/57_Confidence_0.108_Length_895, vo_c1_Locus_21671_Transcript_6/49_Confidence_0.021_Length_1919, vo_c3_Locus_1273_Transcript_50/57_Confidence_0.144_Length_1471, vo_n1_Locus_443_Transcript_24/53_Confidence_0.109_Length_905, vo_n2_Locus_311_Transcript_87/114_Confidence_0.061_Length_2919, vo_n3_Locus_407_Transcript_22/43_Confidence_0.292_Length_1222, tri_n_comp10070_c0_seq2, tri_c_comp22021_c0_seq1, vo_n1_Locus_443_Transcript_32/53_Confidence_0.252_Length_1164, vo_n3_Locus_407_Transcript_32/43_Confidence_0.283_Length_1271, vo_n1_Locus_443_Transcript_10/53_Confidence_0.193_Length_1076, vo_n3_Locus_407_Transcript_7/43_Confidence_0.132_Length_1778, vo_n2_Locus_311_Transcript_109/114_Confidence_0.117_Length_1018, vo_n3_Locus_407_Transcript_39/43_Confidence_0.236_Length_1222, vo_c3_Locus_15467_Transcript_2/2_Confidence_0.000_Length_1100, vo_n1_Locus_4880_Transcript_2/6_Confidence_0.333_Length_883, vo_n2_Locus_3013_Transcript_1/2_Confidence_0.750_Length_964, vo_n2_Locus_311_Transcript_112/114_Confidence_0.146_Length_1678, vo_c2_Locus_601_Transcript_27/36_Confidence_0.337_Length_991, vo_c2_Locus_601_Transcript_2/36_Confidence_0.133_Length_1744, vo_n1_Locus_443_Transcript_39/53_Confidence_0.210_Length_1938, vo_c3_Locus_15467_Transcript_1/2_Confidence_0.750_Length_988, vo_c1_Locus_430_Transcript_11/51_Confidence_0.145_Length_955, vo_c2_Locus_5092_Transcript_10/14_Confidence_0.429_Length_1495, vo_n2_Locus_21867_Transcript_2/5_Confidence_0.200_Length_5139, tri_n_comp20432_c0_seq6, vo_n2_Locus_311_Transcript_84/114_Confidence_0.117_Length_1124, vo_n2_Locus_311_Transcript_77/114_Confidence_0.134_Length_997, vo_n2_Locus_311_Transcript_90/114_Confidence_0.061_Length_1016, vo_c2_Locus_601_Transcript_11/36_Confidence_0.289_Length_1016, tri_n_comp20432_c0_seq2, vo_c1_Locus_430_Transcript_48/51_Confidence_0.227_Length_968, vo_n1_Locus_443_Transcript_17/53_Confidence_0.168_Length_1009, tri_c_comp8925_c1_seq1, vo_c2_Locus_601_Transcript_18/36_Confidence_0.265_Length_801, vo_c3_Locus_1273_Transcript_17/57_Confidence_0.050_Length_1126, vo_n3_Locus_407_Transcript_40/43_Confidence_0.217_Length_1200, vo_c2_Locus_601_Transcript_36/36_Confidence_0.217_Length_953, vo_n3_Locus_407_Transcript_8/43_Confidence_0.189_Length_1800, vo_c3_Locus_1273_Transcript_16/57_Confidence_0.079_Length_1101, tri_n_comp20662_c0_seq7, vo_n2_Locus_23060_Transcript_2/2_Confidence_0.000_Length_889, vo_c2_Locus_601_Transcript_15/36_Confidence_0.337_Length_815, vo_n1_Locus_4880_Transcript_4/6_Confidence_0.333_Length_1109, vo_n2_Locus_311_Transcript_3/114_Confidence_0.142_Length_866, tri_c_comp20860_c0_seq2, vo_n1_Locus_443_Transcript_8/53_Confidence_0.261_Length_1031, vo_n1_Locus_443_Transcript_49/53_Confidence_0.134_Length_862, vo_n3_Locus_407_Transcript_14/43_Confidence_0.142_Length_872, vo_c2_Locus_5609_Transcript_4/4_Confidence_0.250_Length_1105, vo_c3_Locus_17900_Transcript_1/1_Confidence_0.000_Length_966, tri_c_comp3336_c0_seq2, tri_c_comp14506_c0_seq1, vo_c2_Locus_8559_Transcript_12/12_Confidence_0.130_Length_2414, tri_c_comp3336_c0_seq1, vo_n2_Locus_16855_Transcript_8/9_Confidence_0.636_Length_2285, vo_c2_Locus_3936_Transcript_7/12_Confidence_0.412_Length_660, vo_c2_Locus_3936_Transcript_4/12_Confidence_0.353_Length_588, vo_c2_Locus_3936_Transcript_9/12_Confidence_0.235_Length_3618, vo_n2_Locus_22608_Transcript_1/1_Confidence_0.000_Length_1353, tri_c_comp15460_c1_seq4, vo_n2_Locus_1545_Transcript_1/1_Confidence_0.000_Length_1374, vo_c3_Locus_4113_Transcript_2/4_Confidence_0.500_Length_1659, vo_c3_Locus_19662_Transcript_1/2_Confidence_0.833_Length_5009, vo_c3_Locus_1617_Transcript_6/6_Confidence_0.222_Length_1645, vo_n1_Locus_16053_Transcript_1/1_Confidence_0.000_Length_1438, vo_c3_Locus_19662_Transcript_1/2_Confidence_0.833_Length_5009, vo_c3_Locus_1617_Transcript_6/6_Confidence_0.222_Length_1645, vo_n1_Locus_16053_Transcript_1/1_Confidence_0.000_Length_1438, vo_n1_Locus_29872_Transcript_1/1_Confidence_0.000_Length_1764, vo_c2_Locus_3936_Transcript_7/12_Confidence_0.412_Length_660, vo_c2_Locus_3936_Transcript_4/12_Confidence_0.353_Length_588, vo_c2_Locus_3936_Transcript_9/12_Confidence_0.235_Length_3618, vo_c1_Locus_11476_Transcript_1/4_Confidence_0.667_Length_1434, tri_c_comp15460_c1_seq4, vo_n2_Locus_1545_Transcript_1/1_Confidence_0.000_Length_1374, tri_c_comp14949_c0_seq1, vo_n2_Locus_4057_Transcript_1/1_Confidence_0.000_Length_1221, vo_n1_Locus_7235_Transcript_3/6_Confidence_0.625_Length_1162, vo_n2_Locus_7309_Transcript_1/2_Confidence_1.000_Length_1642, vo_c1_Locus_947_Transcript_1/7_Confidence_0.286_Length_2009, vo_c3_Locus_6042_Transcript_5/5_Confidence_0.300_Length_3385, vo_c3_Locus_4758_Transcript_1/1_Confidence_0.000_Length_1881, tri_n_comp25890_c0_seq1, tri_c_comp20356_c0_seq35, tri_c_comp20356_c0_seq10, tri_c_comp20356_c0_seq21,

					vo_n2_Locus_6470_Transcript_5/10_Confidence_0.333_Length_2772, tri_c_comp20356_c0_seq29, tri_c_comp20356_c0_seq27, tri_c_comp20356_c0_seq25, tri_c_comp20356_c0_seq36, tri_c_comp20356_c0_seq47

Arginine and proline metabolism	map00330	15	ec:1.4.3.3 - oxidase, ec:1.14.11.2 - dioxygenase, ec:2.5.1.16 - synthase, ec:2.5.1.22 - synthase, ec:2.6.1.81 - transaminase, ec:4.1.1.17 - decarboxylase, ec:2.6.1.1 - transaminase, ec:1.2.1.3 - dehydrogenase (NAD+), ec:3.5.3.11 - agmatine ureohydrolase, ec:1.5.1.2 - reductase, ec:3.5.1.4 - acylamidase, ec:2.7.3.2 - kinase, ec:3.5.3.1 - arginine amidinase, ec:2.7.2.11 - 5-kinase, ec:5.1.1.4 - racemase	1, 20, 1, 2, 1, 1, 4, 5, 1, 1, 3, 2, 1, 1, 1	vo_n2_Locus_26350_Transcript_7/9_Confidence_0.231_Length_1309, vo_c1_Locus_1460_Transcript_4/35_Confidence_0.155_Length_1407, vo_c2_Locus_16106_Transcript_1/1_Confidence_0.000_Length_993, vo_c3_Locus_1745_Transcript_21/21_Confidence_0.302_Length_1239, vo_n1_Locus_7577_Transcript_4/4_Confidence_0.429_Length_967, vo_c1_Locus_1460_Transcript_3/35_Confidence_0.172_Length_836, vo_c1_Locus_8740_Transcript_2/3_Confidence_0.333_Length_2420, tri_c_comp17414_c0_seq2, vo_c1_Locus_1460_Transcript_1/35_Confidence_0.155_Length_884, vo_n2_Locus_845_Transcript_1/1_Confidence_0.000_Length_944, vo_c2_Locus_4878_Transcript_2/4_Confidence_0.500_Length_1100, vo_c1_Locus_1460_Transcript_28/35_Confidence_0.207_Length_884, vo_n1_Locus_3958_Transcript_9/10_Confidence_0.211_Length_1244, vo_c1_Locus_1460_Transcript_17/35_Confidence_0.155_Length_1027, vo_n2_Locus_1182_Transcript_13/16_Confidence_0.488_Length_1489, vo_n1_Locus_3958_Transcript_2/10_Confidence_0.763_Length_1031, tri_n_comp19826_c0_seq2, vo_c3_Locus_16783_Transcript_1/1_Confidence_0.600_Length_988, tri_c_comp20282_c0_seq4, vo_c1_Locus_1460_Transcript_24/35_Confidence_0.241_Length_982, tri_c_comp35917_c0_seq1, vo_c2_Locus_25147_Transcript_1/2_Confidence_0.333_Length_1097, vo_c2_Locus_25147_Transcript_1/2_Confidence_0.333_Length_1097, tri_c_comp23701_c0_seq1, vo_c1_Locus_5351_Transcript_2/2_Confidence_0.333_Length_1693, vo_n1_Locus_24_Transcript_2/4_Confidence_0.667_Length_1878, vo_n1_Locus_3423_Transcript_2/4_Confidence_0.250_Length_1347, tri_n_comp27683_c0_seq1, vo_c1_Locus_3127_Transcript_1/1_Confidence_0.000_Length_1478, vo_n1_Locus_3423_Transcript_3/4_Confidence_0.250_Length_1416, tri_c_comp55340_c0_seq1, vo_n1_Locus_19864_Transcript_2/2_Confidence_0.000_Length_2707, vo_c3_Locus_21741_Transcript_5/9_Confidence_0.583_Length_1757, vo_n3_Locus_25600_Transcript_5/6_Confidence_0.375_Length_1777, tri_n_comp58061_c0_seq1, vo_c3_Locus_13257_Transcript_3/4_Confidence_0.375_Length_1594, vo_c3_Locus_7050_Transcript_5/6_Confidence_0.667_Length_1047, vo_c3_Locus_1581_Transcript_3/3_Confidence_0.000_Length_1736, vo_c1_Locus_1298_Transcript_3/6_Confidence_0.625_Length_1775, vo_n2_Locus_157_Transcript_2/4_Confidence_0.286_Length_2059, vo_c3_Locus_2801_Transcript_665/665_Confidence_0.000_Length_5508, vo_c3_Locus_2801_Transcript_664/665_Confidence_0.002_Length_5469, vo_c2_Locus_4334_Transcript_2/31_Confidence_0.064_Length_1982, vo_c2_Locus_12865_Transcript_1/3_Confidence_0.667_Length_1839, vo_n2_Locus_19368_Transcript_1/1_Confidence_0.000_Length_1215

Galactose metabolism	map00052	14	ec:2.7.1.11 - phosphohexokinase, ec:2.7.7.10 - uridylyltransferase, ec:2.7.7.12 - uridylyltransferase, ec:3.2.1.20 - maltase, ec:3.2.1.22 - melibiase, ec:3.2.1.23 - lactase (ambiguous), ec:2.7.1.2 - glucokinase (phosphorylating), ec:2.7.1.6 - galactokinase (phosphorylating), ec:2.4.1.123 - 3-alpha-galactosyltransferase, ec:5.1.3.2 - 4-epimerase, ec:2.7.7.9 - uridylyltransferase, ec:5.4.99.9 - mutase, ec:5.4.2.2 - (alpha-D-glucose-1,6-bisphosphate-dependent), ec:1.1.1.21 - reductase	5, 5, 1, 1, 5, 6, 2, 4, 5, 9, 10, 3, 7, 4	vo_c3_Locus_1613_Transcript_6/17_Confidence_0.158_Length_5809, vo_n1_Locus_10193_Transcript_1/1_Confidence_0.000_Length_1647, vo_c2_Locus_6348_Transcript_1/2_Confidence_1.000_Length_3755, vo_n3_Locus_23556_Transcript_2/4_Confidence_0.500_Length_4210, tri_c_comp43537_c0_seq1, vo_n2_Locus_12563_Transcript_1/1_Confidence_0.000_Length_1886, vo_c1_Locus_21075_Transcript_1/3_Confidence_0.500_Length_1034, vo_n1_Locus_3509_Transcript_2/2_Confidence_0.000_Length_1894, tri_c_comp13085_c0_seq3, tri_c_comp54583_c0_seq1, vo_n2_Locus_26011_Transcript_1/2_Confidence_0.333_Length_2712, vo_n2_Locus_3872_Transcript_1/1_Confidence_0.000_Length_2389, vo_c2_Locus_17150_Transcript_58/102_Confidence_0.019_Length_2519, vo_n2_Locus_11259_Transcript_1/2_Confidence_0.333_Length_2880, tri_c_comp44396_c0_seq1, vo_n2_Locus_31986_Transcript_2/2_Confidence_0.333_Length_2185, vo_n2_Locus_20375_Transcript_1/2_Confidence_0.333_Length_1577, tri_c_comp12867_c0_seq1, vo_n2_Locus_1748_Transcript_2/3_Confidence_0.333_Length_4221, tri_n_comp70995_c0_seq1, vo_c2_Locus_26351_Transcript_1/2_Confidence_0.500_Length_4013, vo_c3_Locus_8047_Transcript_1/2_Confidence_0.333_Length_4021, vo_c3_Locus_32079_Transcript_2/2_Confidence_0.250_Length_2113, tri_c_comp10536_c0_seq1, vo_c2_Locus_32726_Transcript_2/2_Confidence_0.000_Length_696, vo_n1_Locus_7597_Transcript_1/2_Confidence_0.333_Length_2494, tri_c_comp10536_c0_seq1, vo_c1_Locus_21386_Transcript_3/8_Confidence_0.538_Length_942, vo_c2_Locus_32726_Transcript_2/2_Confidence_0.000_Length_696, vo_c1_Locus_22386_Transcript_1/2_Confidence_0.967_Length_2287, vo_c2_Locus_27372_Transcript_1/2_Confidence_0.969_Length_2448, vo_c2_Locus_6506_Transcript_2/2_Confidence_0.000_Length_692, vo_c3_Locus_24139_Transcript_1/2_Confidence_0.971_Length_2271, vo_n3_Locus_28820_Transcript_1/2_Confidence_0.950_Length_2237, vo_c2_Locus_13936_Transcript_11/19_Confidence_0.261_Length_294, tri_n_comp13578_c0_seq1, vo_n1_Locus_7847_Transcript_10/14_Confidence_0.579_Length_1000, vo_n2_Locus_711_Transcript_4/13_Confidence_0.474_Length_608, vo_c3_Locus_20156_Transcript_3/3_Confidence_0.000_Length_1297, vo_n2_Locus_711_Transcript_10/13_Confidence_0.316_Length_1285, vo_n2_Locus_711_Transcript_7/13_Confidence_0.474_Length_690, vo_c2_Locus_13936_Transcript_3/19_Confidence_0.435_Length_599, vo_n1_Locus_7847_Transcript_3/14_Confidence_0.105_Length_511, vo_n1_Locus_14248_Transcript_2/11_Confidence_0.333_Length_688, vo_c1_Locus_21075_Transcript_1/3_Confidence_0.500_Length_1034, vo_c2_Locus_2206_Transcript_1/2_Confidence_1.000_Length_1671, tri_c_comp13085_c0_seq3, tri_c_comp54583_c0_seq1, vo_c2_Locus_2427_Transcript_3/6_Confidence_0.455_Length_1530, vo_n2_Locus_12563_Transcript_1/1_Confidence_0.000_Length_1886, vo_n2_Locus_6619_Transcript_2/3_Confidence_0.571_Length_1613, vo_n1_Locus_3509_Transcript_2/2_Confidence_0.000_Length_1894, vo_n1_Locus_14248_Transcript_5/11_Confidence_0.222_Length_1040, vo_c2_Locus_3850_Transcript_1/4_Confidence_0.500_Length_2735, vo_c2_Locus_3850_Transcript_4/4_Confidence_0.250_Length_3514, vo_n2_Locus_1476_Transcript_2/3_Confidence_0.750_Length_1867, tri_n_comp18813_c0_seq13, vo_c2_Locus_23037_Transcript_2/2_Confidence_0.000_Length_2109, vo_n3_Locus_362_Transcript_3/4_Confidence_0.364_Length_3895, vo_n3_Locus_362_Transcript_1/4_Confidence_0.727_Length_3306, vo_n1_Locus_5208_Transcript_2/2_Confidence_0.000_Length_2077, vo_c2_Locus_23037_Transcript_1/2_Confidence_0.750_Length_1965, vo_n3_Locus_15336_Transcript_1/1_Confidence_0.000_Length_1810, vo_c1_Locus_13556_Transcript_2/2_Confidence_0.000_Length_1297, tri_c_comp22527_c0_seq1, vo_n1_Locus_10504_Transcript_1/2_Confidence_0.750_Length_1211, vo_c2_Locus_9062_Transcript_1/11_Confidence_0.312_Length_4797

Pentose and glucuronate interconversions	map00040	14	ec:2.7.1.43 - glucuronokinase (phosphorylating), ec:1.1.1.2 - dehydrogenase (NADP+), ec:1.1.1.9 - reductase, ec:2.7.7.44 - uridylyltransferase, ec:2.4.1.17 - 1-naphthol glucuronyltransferase, ec:3.2.1.15 - pectin depolymerase, ec:5.1.3.1 - 3-epimerase, ec:2.7.7.9 - uridylyltransferase, ec:1.1.1.57 - reductase, ec:1.1.1.14 - 2-dehydrogenase, ec:1.1.1.12 - 4-dehydrogenase, ec:1.1.1.19 - reductase, ec:1.1.1.22 - 6-dehydrogenase, ec:1.1.1.21 - reductase	2, 10, 2, 5, 13, 8, 3, 10, 3, 3, 1, 2, 1, 4	tri_c_comp10536_c0_seq1, vo_c2_Locus_32726_Transcript_2/2_Confidence_0.000_Length_696, tri_c_comp11242_c0_seq2, tri_n_comp64914_c0_seq1, vo_c2_Locus_9062_Transcript_10/11_Confidence_0.312_Length_4945, vo_n2_Locus_22224_Transcript_1/1_Confidence_0.000_Length_1370, tri_n_comp14915_c0_seq1, vo_n3_Locus_15713_Transcript_4/7_Confidence_0.429_Length_2557, tri_c_comp37399_c0_seq1, tri_n_comp58061_c0_seq1, vo_n1_Locus_22736_Transcript_1/2_Confidence_0.333_Length_1213, vo_n2_Locus_19677_Transcript_1/1_Confidence_0.000_Length_2823, vo_c3_Locus_27324_Transcript_1/1_Confidence_0.000_Length_1468, vo_n3_Locus_17668_Transcript_1/2_Confidence_1.000_Length_1468, vo_n2_Locus_12563_Transcript_1/1_Confidence_0.000_Length_1886, vo_c1_Locus_21075_Transcript_1/3_Confidence_0.500_Length_1034, vo_n1_Locus_3509_Transcript_2/2_Confidence_0.000_Length_1894, tri_c_comp13085_c0_seq3, tri_c_comp54583_c0_seq1, tri_c_comp6209_c0_seq1, vo_c1_Locus_26340_Transcript_5/9_Confidence_0.600_Length_1699, tri_n_comp65990_c0_seq1, vo_n1_Locus_13372_Transcript_1/2_Confidence_0.333_Length_1687, vo_n2_Locus_34154_Transcript_3/3_Confidence_0.000_Length_2043, vo_n2_Locus_668_Transcript_17/17_Confidence_0.070_Length_2403, tri_c_comp58135_c0_seq1, vo_c3_Locus_16093_Transcript_1/1_Confidence_0.000_Length_1750, vo_n1_Locus_13372_Transcript_2/2_Confidence_0.000_Length_1314, vo_c2_Locus_24841_Transcript_3/3_Confidence_0.167_Length_3241, vo_n2_Locus_4774_Transcript_1/1_Confidence_0.000_Length_884, vo_c1_Locus_35615_Transcript_1/1_Confidence_0.000_Length_2075, vo_n1_Locus_15639_Transcript_1/1_Confidence_0.000_Length_1086, tri_c_comp19835_c0_seq11, tri_c_comp19835_c0_seq13, tri_c_comp19835_c0_seq5, vo_n1_Locus_29870_Transcript_1/5_Confidence_0.636_Length_1692, vo_c3_Locus_20087_Transcript_1/3_Confidence_0.714_Length_1659, vo_n3_Locus_5942_Transcript_1/1_Confidence_0.000_Length_2020, vo_c1_Locus_10150_Transcript_1/10_Confidence_0.429_Length_1713, tri_c_comp19835_c0_seq3, vo_n3_Locus_1573_Transcript_4/9_Confidence_0.364_Length_1074, vo_n2_Locus_3298_Transcript_6/11_Confidence_0.333_Length_1072, vo_n1_Locus_7783_Transcript_1/2_Confidence_0.667_Length_900, vo_n1_Locus_14248_Transcript_2/11_Confidence_0.333_Length_688, vo_c1_Locus_21075_Transcript_1/3_Confidence_0.500_Length_1034, vo_c2_Locus_2206_Transcript_1/2_Confidence_1.000_Length_1671, tri_c_comp13085_c0_seq3, tri_c_comp54583_c0_seq1, vo_c2_Locus_2427_Transcript_3/6_Confidence_0.455_Length_1530, vo_n2_Locus_12563_Transcript_1/1_Confidence_0.000_Length_1886, vo_n2_Locus_6619_Transcript_2/3_Confidence_0.571_Length_1613, vo_n1_Locus_3509_Transcript_2/2_Confidence_0.000_Length_1894, vo_n1_Locus_14248_Transcript_5/11_Confidence_0.222_Length_1040, vo_n1_Locus_13598_Transcript_3/3_Confidence_0.143_Length_1862, vo_c1_Locus_7476_Transcript_4/9_Confidence_0.667_Length_1739, vo_n1_Locus_13598_Transcript_1/3_Confidence_0.714_Length_1803, tri_n_comp61454_c0_seq1, vo_c3_Locus_27324_Transcript_1/1_Confidence_0.000_Length_1468, vo_n3_Locus_17668_Transcript_1/2_Confidence_1.000_Length_1468, tri_n_comp61454_c0_seq1, vo_c1_Locus_13556_Transcript_2/2_Confidence_0.000_Length_1297, vo_n1_Locus_10504_Transcript_1/2_Confidence_0.750_Length_1211, vo_c1_Locus_11739_Transcript_1/3_Confidence_0.929_Length_1730, vo_c1_Locus_13556_Transcript_2/2_Confidence_0.000_Length_1297, tri_c_comp22527_c0_seq1, vo_n1_Locus_10504_Transcript_1/2_Confidence_0.750_Length_1211, vo_c2_Locus_9062_Transcript_1/11_Confidence_0.312_Length_4797

Phosphatidylinositol signaling system	map04070	13	ec:2.7.1.107 - kinase (ATP), ec:2.7.1.127 - 3-kinase, ec:2.7.1.67 - 4-kinase, ec:2.7.1.68 - 5-kinase, ec:3.1.4.11 - phospholipase C, ec:2.7.1.137 - 3-kinase, ec:2.7.1.158 - 2-kinase, ec:2.7.11.13 - kinase C, ec:2.7.7.41 - cytidylyltransferase, ec:3.1.3.56 - 5-phosphatase, ec:3.1.3.67 - 3-phosphatase, ec:3.1.3.64 - inositol-1,3-bisphosphate 3-phosphatase, ec:3.1.3.25 - phosphatase	9, 4, 1, 69, 2, 3, 1, 36, 1, 1, 4, 3, 5	tri_n_comp47452_c0_seq1, vo_c3_Locus_6073_Transcript_2/9_Confidence_0.467_Length_1975, vo_c2_Locus_6531_Transcript_1/1_Confidence_0.000_Length_1860, tri_c_comp37156_c0_seq1, vo_c1_Locus_17180_Transcript_1/1_Confidence_0.000_Length_4157, vo_c2_Locus_11596_Transcript_2/3_Confidence_0.167_Length_1499, vo_n2_Locus_6369_Transcript_1/1_Confidence_0.000_Length_1901, vo_n2_Locus_8637_Transcript_3/3_Confidence_0.000_Length_1561, vo_c3_Locus_6073_Transcript_3/9_Confidence_0.400_Length_2122, vo_c1_Locus_11471_Transcript_1/1_Confidence_0.000_Length_1552, vo_n1_Locus_10442_Transcript_2/3_Confidence_0.400_Length_4947, tri_n_comp57149_c0_seq1, vo_n3_Locus_25151_Transcript_2/2_Confidence_0.000_Length_2321, vo_c1_Locus_8439_Transcript_1/1_Confidence_0.000_Length_2856, vo_n2_Locus_10900_Transcript_7/8_Confidence_0.182_Length_1541, vo_c2_Locus_1347_Transcript_3/5_Confidence_0.500_Length_2327, vo_n2_Locus_22259_Transcript_6/9_Confidence_0.300_Length_1350, tri_n_comp34533_c0_seq1, vo_c2_Locus_16394_Transcript_1/3_Confidence_0.600_Length_1523, tri_n_comp47088_c0_seq1, vo_n2_Locus_5776_Transcript_2/5_Confidence_0.400_Length_1068, tri_n_comp20588_c0_seq18, tri_n_comp20588_c0_seq19, tri_n_comp20588_c0_seq17, tri_n_comp20588_c0_seq14, vo_c1_Locus_42275_Transcript_3/3_Confidence_0.556_Length_279, vo_c1_Locus_7030_Transcript_2/2_Confidence_0.000_Length_1330, tri_n_comp20588_c0_seq12, vo_n2_Locus_8440_Transcript_2/5_Confidence_0.375_Length_590, tri_c_comp22370_c0_seq1, vo_c2_Locus_8945_Transcript_5/8_Confidence_0.500_Length_1223, vo_n1_Locus_5753_Transcript_1/1_Confidence_0.000_Length_1679, vo_c3_Locus_7815_Transcript_4/6_Confidence_0.214_Length_985, tri_n_comp36241_c0_seq1, vo_c2_Locus_2278_Transcript_2/4_Confidence_0.556_Length_2279, tri_n_comp58246_c0_seq1, vo_n2_Locus_2705_Transcript_42/102_Confidence_0.021_Length_886, tri_n_comp20588_c0_seq8, vo_c1_Locus_1892_Transcript_3/5_Confidence_0.500_Length_2124, vo_c1_Locus_8772_Transcript_7/10_Confidence_0.235_Length_1211, vo_n2_Locus_6471_Transcript_2/29_Confidence_0.525_Length_5179, tri_c_comp32581_c0_seq1, vo_n3_Locus_32114_Transcript_2/2_Confidence_0.000_Length_539, vo_n2_Locus_20439_Transcript_1/1_Confidence_0.667_Length_1183, vo_c2_Locus_16479_Transcript_4/5_Confidence_0.500_Length_1223, vo_n2_Locus_6607_Transcript_2/3_Confidence_0.714_Length_1081, tri_c_comp33632_c0_seq1, vo_n2_Locus_29144_Transcript_1/1_Confidence_0.000_Length_1255, tri_c_comp34002_c0_seq1, vo_c2_Locus_13720_Transcript_3/4_Confidence_0.667_Length_997, vo_n2_Locus_6471_Transcript_7/29_Confidence_0.525_Length_5327, vo_n1_Locus_12692_Transcript_1/5_Confidence_0.286_Length_2909, vo_n3_Locus_22093_Transcript_2/2_Confidence_0.000_Length_372, vo_c2_Locus_5202_Transcript_1/2_Confidence_0.333_Length_647, tri_c_comp45302_c0_seq1, vo_n1_Locus_5719_Transcript_37/41_Confidence_0.500_Length_5524, tri_n_comp38041_c0_seq1, vo_c2_Locus_3016_Transcript_1/2_Confidence_1.000_Length_1686, vo_c3_Locus_7815_Transcript_5/6_Confidence_0.143_Length_1013, vo_c1_Locus_1170_Transcript_1/1_Confidence_0.000_Length_813, vo_n2_Locus_13406_Transcript_3/4_Confidence_0.667_Length_2171, vo_c3_Locus_3411_Transcript_3/5_Confidence_0.500_Length_2118, vo_c3_Locus_15968_Transcript_1/8_Confidence_0.019_Length_1026, tri_c_comp53370_c0_seq1, vo_c2_Locus_11572_Transcript_1/1_Confidence_0.000_Length_970, tri_n_comp52311_c0_seq1, vo_n2_Locus_13406_Transcript_4/4_Confidence_0.333_Length_2198, vo_c1_Locus_7030_Transcript_1/2_Confidence_0.750_Length_1255, vo_n1_Locus_16464_Transcript_1/2_Confidence_0.333_Length_1172, vo_n2_Locus_5776_Transcript_4/5_Confidence_0.200_Length_888, vo_c1_Locus_2471_Transcript_1/1_Confidence_0.000_Length_834, vo_n1_Locus_6857_Transcript_5/11_Confidence_0.556_Length_1705, vo_n2_Locus_9915_Transcript_1/1_Confidence_0.000_Length_1411, vo_c1_Locus_14433_Transcript_11/14_Confidence_0.294_Length_4956, vo_c2_Locus_502_Transcript_1/1_Confidence_0.000_Length_903, vo_c1_Locus_23583_Transcript_1/2_Confidence_0.333_Length_1107, vo_n2_Locus_1276_Transcript_1/2_Confidence_0.333_Length_497, tri_n_comp36227_c0_seq1, tri_n_comp39413_c0_seq1, tri_n_comp27385_c0_seq1, vo_c1_Locus_16332_Transcript_1/2_Confidence_0.333_Length_1029, vo_n1_Locus_11487_Transcript_3/3_Confidence_0.000_Length_568, vo_c2_Locus_2278_Transcript_4/4_Confidence_0.111_Length_2395, tri_n_comp9871_c0_seq1, tri_c_comp55877_c0_seq1, vo_n1_Locus_5560_Transcript_2/10_Confidence_0.136_Length_787, tri_n_comp56781_c0_seq1, vo_c1_Locus_8439_Transcript_1/1_Confidence_0.000_Length_2856, vo_n1_Locus_10442_Transcript_2/3_Confidence_0.400_Length_4947, vo_c1_Locus_22333_Transcript_11/14_Confidence_0.391_Length_2288, vo_n2_Locus_3054_Transcript_2/3_Confidence_0.333_Length_3045, tri_c_comp18638_c0_seq3, vo_c3_Locus_19291_Transcript_4/6_Confidence_0.250_Length_3644, vo_n1_Locus_5027_Transcript_2/4_Confidence_0.800_Length_3074, tri_c_comp12452_c0_seq2, vo_c3_Locus_4531_Transcript_1/2_Confidence_0.333_Length_3256, vo_c3_Locus_12823_Transcript_7/11_Confidence_0.150_Length_1099, vo_c1_Locus_6709_Transcript_2/10_Confidence_0.444_Length_1193, vo_c3_Locus_17253_Transcript_3/4_Confidence_0.375_Length_1433, tri_n_comp12148_c0_seq1, vo_c1_Locus_6477_Transcript_5/9_Confidence_0.235_Length_1167, vo_n2_Locus_1515_Transcript_15/24_Confidence_0.077_Length_1136, vo_c3_Locus_10023_Transcript_2/6_Confidence_0.125_Length_1290, vo_n2_Locus_1426_Transcript_22/28_Confidence_0.304_Length_1443, vo_c1_Locus_3372_Transcript_11/12_Confidence_0.350_Length_1415, vo_c1_Locus_6709_Transcript_7/10_Confidence_0.500_Length_1389, vo_c3_Locus_1810_Transcript_8/22_Confidence_0.262_Length_1483, vo_n2_Locus_19434_Transcript_1/2_Confidence_0.333_Length_3274, vo_n2_Locus_1426_Transcript_24/28_Confidence_0.304_Length_1547, vo_n1_Locus_3614_Transcript_4/8_Confidence_0.231_Length_1789, vo_n1_Locus_842_Transcript_34/37_Confidence_0.203_Length_1893, vo_n3_Locus_877_Transcript_3/5_Confidence_0.400_Length_1624, vo_c3_Locus_1810_Transcript_9/22_Confidence_0.262_Length_1447, vo_c1_Locus_2046_Transcript_1/1_Confidence_0.000_Length_2599, vo_c2_Locus_10357_Transcript_15/15_Confidence_0.045_Length_698, vo_c3_Locus_12823_Transcript_5/11_Confidence_0.200_Length_1070, vo_n1_Locus_3614_Transcript_2/8_Confidence_0.462_Length_2211, vo_c1_Locus_6709_Transcript_9/10_Confidence_0.278_Length_1468, vo_c2_Locus_16186_Transcript_7/8_Confidence_0.143_Length_2140,

					vo_n1_Locus_842_Transcript_11/37_Confidence_0.188_Length_2091, vo_c3_Locus_1810_Transcript_17/22_Confidence_0.214_Length_1421, vo_n2_Locus_1426_Transcript_12/28_Confidence_0.370_Length_1887, vo_n2_Locus_1426_Transcript_16/28_Confidence_0.326_Length_1443, vo_n1_Locus_14687_Transcript_3/3_Confidence_0.200_Length_1029, vo_c3_Locus_1810_Transcript_5/22_Confidence_0.310_Length_1343, vo_c3_Locus_4113_Transcript_2/4_Confidence_0.500_Length_1659, vo_n3_Locus_9268_Transcript_2/3_Confidence_0.400_Length_3937, vo_n3_Locus_22682_Transcript_1/1_Confidence_0.000_Length_2879, tri_n_comp6659_c0_seq1, vo_n2_Locus_183_Transcript_39/101_Confidence_0.031_Length_2986, vo_n3_Locus_1224_Transcript_33/39_Confidence_0.173_Length_3279, vo_c1_Locus_25427_Transcript_1/1_Confidence_0.000_Length_2216, vo_n2_Locus_183_Transcript_39/101_Confidence_0.031_Length_2986, vo_n3_Locus_22682_Transcript_1/1_Confidence_0.000_Length_2879, vo_n2_Locus_24510_Transcript_1/1_Confidence_0.000_Length_1024, vo_c2_Locus_24561_Transcript_2/2_Confidence_0.000_Length_1596, vo_c2_Locus_24561_Transcript_1/2_Confidence_0.750_Length_1539, vo_c1_Locus_21354_Transcript_1/6_Confidence_0.600_Length_1404, vo_c2_Locus_13210_Transcript_4/4_Confidence_0.125_Length_1454

Inositol phosphate metabolism	map00562	13	ec:2.7.1.127 - 3-kinase, ec:2.7.1.67 - 4-kinase, ec:2.7.1.68 - 5-kinase, ec:3.1.4.11 - phospholipase C, ec:1.2.1.18 - dehydrogenase (acetylating), ec:2.7.1.137 - 3-kinase, ec:2.7.1.158 - 2-kinase, ec:5.3.1.1 - isomerase, ec:3.1.3.56 - 5-phosphatase, ec:3.1.3.67 - 3-phosphatase, ec:3.1.3.64 - inositol-1,3-bisphosphate 3-phosphatase, ec:3.1.3.25 - phosphatase, ec:1.13.99.1 - oxygenase	4, 1, 69, 2, 3, 3, 1, 9, 1, 4, 3, 5, 7	vo_c1_Locus_11471_Transcript_1/1_Confidence_0.000_Length_1552, vo_n1_Locus_10442_Transcript_2/3_Confidence_0.400_Length_4947, tri_n_comp57149_c0_seq1, vo_n3_Locus_25151_Transcript_2/2_Confidence_0.000_Length_2321, vo_c1_Locus_8439_Transcript_1/1_Confidence_0.000_Length_2856, vo_n2_Locus_10900_Transcript_7/8_Confidence_0.182_Length_1541, vo_c2_Locus_1347_Transcript_3/5_Confidence_0.500_Length_2327, vo_n2_Locus_22259_Transcript_6/9_Confidence_0.300_Length_1350, tri_n_comp34533_c0_seq1, vo_c2_Locus_16394_Transcript_1/3_Confidence_0.600_Length_1523, tri_n_comp47088_c0_seq1, vo_n2_Locus_5776_Transcript_2/5_Confidence_0.400_Length_1068, tri_n_comp20588_c0_seq18, tri_n_comp20588_c0_seq19, tri_n_comp20588_c0_seq17, tri_n_comp20588_c0_seq14, vo_c1_Locus_42275_Transcript_3/3_Confidence_0.556_Length_279, vo_c1_Locus_7030_Transcript_2/2_Confidence_0.000_Length_1330, tri_n_comp20588_c0_seq12, vo_n2_Locus_8440_Transcript_2/5_Confidence_0.375_Length_590, tri_c_comp22370_c0_seq1, vo_c2_Locus_8945_Transcript_5/8_Confidence_0.500_Length_1223, vo_n1_Locus_5753_Transcript_1/1_Confidence_0.000_Length_1679, vo_c3_Locus_7815_Transcript_4/6_Confidence_0.214_Length_985, tri_n_comp36241_c0_seq1, vo_c2_Locus_2278_Transcript_2/4_Confidence_0.556_Length_2279, tri_n_comp58246_c0_seq1, vo_n2_Locus_2705_Transcript_42/102_Confidence_0.021_Length_886, tri_n_comp20588_c0_seq8, vo_c1_Locus_1892_Transcript_3/5_Confidence_0.500_Length_2124, vo_c1_Locus_8772_Transcript_7/10_Confidence_0.235_Length_1211, vo_n2_Locus_6471_Transcript_2/29_Confidence_0.525_Length_5179, tri_c_comp32581_c0_seq1, vo_n3_Locus_32114_Transcript_2/2_Confidence_0.000_Length_539, vo_n2_Locus_20439_Transcript_1/1_Confidence_0.667_Length_1183, vo_c2_Locus_16479_Transcript_4/5_Confidence_0.500_Length_1223, vo_n2_Locus_6607_Transcript_2/3_Confidence_0.714_Length_1081, tri_c_comp33632_c0_seq1, vo_n2_Locus_29144_Transcript_1/1_Confidence_0.000_Length_1255, tri_c_comp34002_c0_seq1, vo_c2_Locus_13720_Transcript_3/4_Confidence_0.667_Length_997, vo_n2_Locus_6471_Transcript_7/29_Confidence_0.525_Length_5327, vo_n1_Locus_12692_Transcript_1/5_Confidence_0.286_Length_2909, vo_n3_Locus_22093_Transcript_2/2_Confidence_0.000_Length_372, vo_c2_Locus_5202_Transcript_1/2_Confidence_0.333_Length_647, tri_c_comp45302_c0_seq1, vo_n1_Locus_5719_Transcript_37/41_Confidence_0.500_Length_5524, tri_n_comp38041_c0_seq1, vo_c2_Locus_3016_Transcript_1/2_Confidence_1.000_Length_1686, vo_c3_Locus_7815_Transcript_5/6_Confidence_0.143_Length_1013, vo_c1_Locus_1170_Transcript_1/1_Confidence_0.000_Length_813, vo_n2_Locus_13406_Transcript_3/4_Confidence_0.667_Length_2171, vo_c3_Locus_3411_Transcript_3/5_Confidence_0.500_Length_2118, vo_c3_Locus_15968_Transcript_1/8_Confidence_0.019_Length_1026, tri_c_comp53370_c0_seq1, vo_c2_Locus_11572_Transcript_1/1_Confidence_0.000_Length_970, tri_n_comp52311_c0_seq1, vo_n2_Locus_13406_Transcript_4/4_Confidence_0.333_Length_2198, vo_c1_Locus_7030_Transcript_1/2_Confidence_0.750_Length_1255, vo_n1_Locus_16464_Transcript_1/2_Confidence_0.333_Length_1172, vo_n2_Locus_5776_Transcript_4/5_Confidence_0.200_Length_888, vo_c1_Locus_2471_Transcript_1/1_Confidence_0.000_Length_834, vo_n1_Locus_6857_Transcript_5/11_Confidence_0.556_Length_1705, vo_n2_Locus_9915_Transcript_1/1_Confidence_0.000_Length_1411, vo_c1_Locus_14433_Transcript_11/14_Confidence_0.294_Length_4956, vo_c2_Locus_502_Transcript_1/1_Confidence_0.000_Length_903, vo_c1_Locus_23583_Transcript_1/2_Confidence_0.333_Length_1107, vo_n2_Locus_1276_Transcript_1/2_Confidence_0.333_Length_497, tri_n_comp36227_c0_seq1, tri_n_comp39413_c0_seq1, tri_n_comp27385_c0_seq1, vo_c1_Locus_16332_Transcript_1/2_Confidence_0.333_Length_1029, vo_n1_Locus_11487_Transcript_3/3_Confidence_0.000_Length_568, vo_c2_Locus_2278_Transcript_4/4_Confidence_0.111_Length_2395, tri_n_comp9871_c0_seq1, tri_c_comp55877_c0_seq1, vo_c3_Locus_1581_Transcript_3/3_Confidence_0.000_Length_1736, vo_c1_Locus_1298_Transcript_3/6_Confidence_0.625_Length_1775, vo_n2_Locus_157_Transcript_2/4_Confidence_0.286_Length_2059, vo_n1_Locus_5560_Transcript_2/10_Confidence_0.136_Length_787, tri_n_comp56781_c0_seq1, vo_c1_Locus_8439_Transcript_1/1_Confidence_0.000_Length_2856, vo_n1_Locus_10442_Transcript_2/3_Confidence_0.400_Length_4947, vo_c2_Locus_3634_Transcript_8/8_Confidence_0.133_Length_2428, vo_c2_Locus_324_Transcript_5/12_Confidence_0.333_Length_1009, vo_n1_Locus_5721_Transcript_6/9_Confidence_0.389_Length_989, vo_c2_Locus_324_Transcript_11/12_Confidence_0.333_Length_4393, vo_n2_Locus_475_Transcript_7/13_Confidence_0.312_Length_1141, vo_n1_Locus_5721_Transcript_1/9_Confidence_0.278_Length_986, vo_n2_Locus_475_Transcript_2/13_Confidence_0.312_Length_1187, vo_c2_Locus_324_Transcript_2/12_Confidence_0.400_Length_1106, vo_c3_Locus_3927_Transcript_2/9_Confidence_0.250_Length_2629, vo_n3_Locus_9268_Transcript_2/3_Confidence_0.400_Length_3937, vo_n3_Locus_22682_Transcript_1/1_Confidence_0.000_Length_2879, tri_n_comp6659_c0_seq1, vo_n2_Locus_183_Transcript_39/101_Confidence_0.031_Length_2986, vo_n3_Locus_1224_Transcript_33/39_Confidence_0.173_Length_3279, vo_c1_Locus_25427_Transcript_1/1_Confidence_0.000_Length_2216, vo_n2_Locus_183_Transcript_39/101_Confidence_0.031_Length_2986, vo_n3_Locus_22682_Transcript_1/1_Confidence_0.000_Length_2879, vo_n2_Locus_24510_Transcript_1/1_Confidence_0.000_Length_1024, vo_c2_Locus_24561_Transcript_2/2_Confidence_0.000_Length_1596, vo_c2_Locus_24561_Transcript_1/2_Confidence_0.750_Length_1539, vo_c1_Locus_21354_Transcript_1/6_Confidence_0.600_Length_1404, vo_c2_Locus_13210_Transcript_4/4_Confidence_0.125_Length_1454, vo_c2_Locus_7343_Transcript_5/6_Confidence_0.333_Length_1107, vo_c1_Locus_16134_Transcript_10/12_Confidence_0.200_Length_544, vo_c3_Locus_9233_Transcript_4/5_Confidence_0.375_Length_1432, vo_c2_Locus_7343_Transcript_6/6_Confidence_0.083_Length_1098, vo_c1_Locus_16134_Transcript_6/12_Confidence_0.250_Length_448, vo_c1_Locus_16134_Transcript_8/12_Confidence_0.150_Length_721, vo_c1_Locus_16134_Transcript_7/12_Confidence_0.200_Length_748

Pantothenate and CoA biosynthesis	map00770	13	ec:4.1.1.36 - decarboxylase, ec:2.2.1.6 - synthase, ec:2.7.1.33 - kinase, ec:2.6.1.42 - transaminase, ec:2.7.1.24 - kinase, ec:2.7.8.7 - synthase, ec:1.1.1.169 - 2-reductase, ec:3.6.1.9 - diphosphatase, ec:4.2.1.9 - dehydratase, ec:6.3.2.5 - ligase, ec:2.7.7.3 - adenylyltransferase, ec:2.1.2.11 - hydroxymethyltransferase, ec:1.1.1.86 - reductoisomerase (NADP+)	1, 1, 2, 6, 1, 4, 1, 7, 2, 1, 1, 3, 1	vo_n1_Locus_30711_Transcript_1/1_Confidence_0.000_Length_904, tri_n_comp26150_c0_seq1, vo_c2_Locus_25467_Transcript_3/4_Confidence_0.500_Length_2039, tri_c_comp48007_c0_seq1, vo_c2_Locus_384_Transcript_2/3_Confidence_0.400_Length_1725, tri_c_comp32679_c0_seq1, vo_c1_Locus_10788_Transcript_1/1_Confidence_0.000_Length_1721, vo_c2_Locus_14969_Transcript_4/8_Confidence_0.750_Length_1231, vo_n2_Locus_3786_Transcript_2/4_Confidence_0.778_Length_1466, vo_n2_Locus_3786_Transcript_3/4_Confidence_0.667_Length_1442, tri_n_comp6551_c0_seq1, vo_c3_Locus_3787_Transcript_1/1_Confidence_0.000_Length_1203, vo_c1_Locus_5370_Transcript_6/6_Confidence_0.333_Length_1849, vo_c1_Locus_22861_Transcript_1/1_Confidence_0.000_Length_1500, vo_c1_Locus_5370_Transcript_4/6_Confidence_0.267_Length_1370, vo_c3_Locus_23517_Transcript_1/1_Confidence_0.000_Length_1261, vo_c2_Locus_35825_Transcript_1/2_Confidence_0.500_Length_995, vo_c2_Locus_35825_Transcript_2/2_Confidence_0.000_Length_972, tri_n_comp34093_c0_seq1, vo_c2_Locus_11939_Transcript_1/4_Confidence_0.273_Length_1336, vo_c1_Locus_18906_Transcript_3/4_Confidence_0.400_Length_1298, vo_c2_Locus_26280_Transcript_1/1_Confidence_0.000_Length_1316, vo_c1_Locus_31652_Transcript_2/3_Confidence_0.429_Length_964, vo_n1_Locus_12155_Transcript_1/2_Confidence_0.500_Length_2024, vo_n2_Locus_484_Transcript_2/3_Confidence_0.333_Length_2007, vo_n2_Locus_16404_Transcript_1/2_Confidence_1.000_Length_1226, tri_c_comp39996_c0_seq1, vo_c1_Locus_6318_Transcript_9/9_Confidence_0.182_Length_1477, vo_c3_Locus_17424_Transcript_2/4_Confidence_0.400_Length_1582, vo_n2_Locus_3835_Transcript_3/4_Confidence_0.600_Length_1293, vo_n2_Locus_4139_Transcript_1/1_Confidence_0.000_Length_1669

Nicotinate and nicotinamide metabolism	map00760	13	ec:1.2.1.24 - dehydrogenase (NAD+), ec:2.7.1.23 - kinase, ec:2.7.1.22 - kinase, ec:1.2.1.16 - dehydrogenase [NAD(P)+], ec:3.1.3.5 - uridine 5'-nucleotidase, ec:5.3.3.6 - Delta-isomerase, ec:1.6.1.1 - transhydrogenase (Si-specific), ec:1.6.1.2 - transhydrogenase (Re/Si-specific), ec:3.6.1.9 - diphosphatase, ec:1.4.1.21 - dehydrogenase, ec:3.2.2.1 - nucleosidase, ec:2.4.2.19 - diphosphorylase (carboxylating), ec:2.4.2.12 - phosphoribosyltransferase	1, 10, 2, 1, 11, 1, 3, 3, 7, 1, 1, 2, 2	vo_c1_Locus_24159_Transcript_1/2_Confidence_0.333_Length_1573, tri_n_comp47452_c0_seq1, vo_c3_Locus_6073_Transcript_2/9_Confidence_0.467_Length_1975, vo_c2_Locus_6531_Transcript_1/1_Confidence_0.000_Length_1860, tri_c_comp37156_c0_seq1, vo_c1_Locus_17180_Transcript_1/1_Confidence_0.000_Length_4157, vo_c2_Locus_11596_Transcript_2/3_Confidence_0.167_Length_1499, vo_n2_Locus_6369_Transcript_1/1_Confidence_0.000_Length_1901, vo_n2_Locus_8637_Transcript_3/3_Confidence_0.000_Length_1561, vo_n2_Locus_4220_Transcript_2/4_Confidence_0.400_Length_1659, vo_c3_Locus_6073_Transcript_3/9_Confidence_0.400_Length_2122, vo_c3_Locus_8424_Transcript_2/2_Confidence_0.000_Length_1147, tri_n_comp69389_c0_seq1, vo_c1_Locus_24159_Transcript_1/2_Confidence_0.333_Length_1573, vo_c1_Locus_21072_Transcript_1/1_Confidence_0.000_Length_965, vo_c2_Locus_25862_Transcript_4/4_Confidence_0.000_Length_988, vo_c2_Locus_26482_Transcript_5/6_Confidence_0.286_Length_1654, vo_c2_Locus_26482_Transcript_4/6_Confidence_0.571_Length_1609, vo_n2_Locus_21191_Transcript_4/6_Confidence_0.300_Length_1691, vo_c1_Locus_18480_Transcript_2/2_Confidence_0.000_Length_1013, vo_c2_Locus_27118_Transcript_12/49_Confidence_0.025_Length_3122, vo_c2_Locus_6520_Transcript_5/15_Confidence_0.455_Length_1419, vo_c3_Locus_7168_Transcript_5/8_Confidence_0.538_Length_1343, vo_n1_Locus_7057_Transcript_3/15_Confidence_0.611_Length_1294, vo_n2_Locus_21191_Transcript_3/6_Confidence_0.500_Length_1646, vo_c1_Locus_22176_Transcript_1/1_Confidence_0.000_Length_1558, vo_n2_Locus_10341_Transcript_2/3_Confidence_0.333_Length_4122, tri_n_comp26117_c0_seq1, vo_n3_Locus_586_Transcript_15/30_Confidence_0.217_Length_1490, vo_n2_Locus_10341_Transcript_2/3_Confidence_0.333_Length_4122, tri_n_comp26117_c0_seq1, vo_n3_Locus_586_Transcript_15/30_Confidence_0.217_Length_1490, vo_c2_Locus_35825_Transcript_1/2_Confidence_0.500_Length_995, vo_c2_Locus_35825_Transcript_2/2_Confidence_0.000_Length_972, tri_n_comp34093_c0_seq1, vo_c2_Locus_11939_Transcript_1/4_Confidence_0.273_Length_1336, vo_c1_Locus_18906_Transcript_3/4_Confidence_0.400_Length_1298, vo_c2_Locus_26280_Transcript_1/1_Confidence_0.000_Length_1316, vo_c1_Locus_31652_Transcript_2/3_Confidence_0.429_Length_964, vo_c1_Locus_38689_Transcript_2/2_Confidence_0.000_Length_1212, vo_n1_Locus_31937_Transcript_5/5_Confidence_0.125_Length_1141, vo_c2_Locus_20360_Transcript_1/1_Confidence_0.000_Length_1892, tri_c_comp24450_c0_seq1, vo_c2_Locus_20360_Transcript_1/1_Confidence_0.000_Length_1892, tri_c_comp24450_c0_seq1

Arginine biosynthesis	map00220	13	ec:2.6.1.11 - transaminase, ec:6.3.1.2 - synthetase, ec:4.3.2.1 - lyase, ec:1.2.1.38 - reductase, ec:2.6.1.1 - transaminase, ec:3.5.1.14 - acid amidohydrolase, ec:2.3.1.1 - N-acetyltransferase, ec:1.4.1.2 - dehydrogenase, ec:3.5.1.5 - ec:3.5.1.5 urease, ec:6.3.4.5 - synthase, ec:3.5.3.1 - arginine amidinase, ec:2.3.1.35 - N-acetyltransferase, ec:6.3.4.16 - synthase (ammonia)	4, 15, 1, 4, 4, 2, 1, 1, 1, 1, 1, 2, 2	vo_c3_Locus_29526_Transcript_7/10_Confidence_0.268_Length_874, vo_n2_Locus_6432_Transcript_1/2_Confidence_0.667_Length_1653, vo_n2_Locus_3256_Transcript_3/4_Confidence_0.250_Length_4070, vo_c1_Locus_5351_Transcript_2/2_Confidence_0.333_Length_1693, vo_c1_Locus_1340_Transcript_12/17_Confidence_0.109_Length_2389, vo_c1_Locus_1340_Transcript_9/17_Confidence_0.172_Length_2429, tri_n_comp11684_c0_seq1, vo_c1_Locus_4136_Transcript_1/2_Confidence_0.967_Length_2259, vo_c2_Locus_6408_Transcript_2/14_Confidence_0.268_Length_1478, vo_n1_Locus_335_Transcript_2/3_Confidence_0.909_Length_2286, vo_c2_Locus_3512_Transcript_3/4_Confidence_0.500_Length_2501, vo_n2_Locus_4820_Transcript_2/3_Confidence_0.545_Length_2376, vo_n3_Locus_2644_Transcript_11/11_Confidence_0.323_Length_2390, vo_n2_Locus_515_Transcript_8/9_Confidence_0.471_Length_3194, vo_n2_Locus_515_Transcript_7/9_Confidence_0.176_Length_2355, vo_c2_Locus_3512_Transcript_2/4_Confidence_0.750_Length_2320, vo_c3_Locus_1501_Transcript_6/9_Confidence_0.203_Length_2420, vo_n2_Locus_5127_Transcript_1/3_Confidence_0.200_Length_1636, vo_n2_Locus_22912_Transcript_1/2_Confidence_0.333_Length_2481, vo_c2_Locus_18300_Transcript_2/3_Confidence_0.200_Length_1671, vo_n2_Locus_3068_Transcript_2/3_Confidence_0.250_Length_1991, vo_n1_Locus_4025_Transcript_3/5_Confidence_0.286_Length_3475, vo_c1_Locus_9004_Transcript_2/4_Confidence_0.400_Length_3537, vo_c1_Locus_10440_Transcript_1/2_Confidence_0.667_Length_1569, vo_n1_Locus_3423_Transcript_2/4_Confidence_0.250_Length_1347, tri_n_comp27683_c0_seq1, vo_c1_Locus_3127_Transcript_1/1_Confidence_0.000_Length_1478, vo_n1_Locus_3423_Transcript_3/4_Confidence_0.250_Length_1416, tri_c_comp31460_c0_seq1, vo_n2_Locus_4794_Transcript_3/3_Confidence_0.000_Length_966, vo_n2_Locus_4233_Transcript_1/2_Confidence_1.000_Length_2049, tri_c_comp28026_c0_seq1, tri_c_comp36137_c0_seq1, vo_c2_Locus_2824_Transcript_1/6_Confidence_0.533_Length_1586, vo_c2_Locus_4334_Transcript_2/31_Confidence_0.064_Length_1982, tri_c_comp38766_c0_seq1, vo_c3_Locus_3449_Transcript_3/3_Confidence_0.000_Length_1945, tri_c_comp33901_c0_seq1, vo_n2_Locus_5805_Transcript_1/2_Confidence_0.333_Length_5227

Glycerolipid metabolism	map00561	13	ec:2.7.1.107 - kinase (ATP), ec:2.7.1.29 - kinase, ec:2.3.1.158 - acyltransferase, ec:3.1.1.3 - lipase, ec:1.1.1.2 - dehydrogenase (NADP+), ec:1.2.1.3 - dehydrogenase (NAD+), ec:3.2.1.22 - melibiase, ec:2.3.1.20 - O-acyltransferase, ec:2.3.1.22 - O-acyltransferase, ec:2.3.1.51 - O-acyltransferase, ec:2.4.1.184 - galactosyltransferase, ec:1.1.1.202 - dehydrogenase, ec:1.1.1.21 - reductase	9, 2, 2, 2, 10, 5, 5, 8, 6, 2, 2, 1, 4	tri_n_comp47452_c0_seq1, vo_c3_Locus_6073_Transcript_2/9_Confidence_0.467_Length_1975, vo_c2_Locus_6531_Transcript_1/1_Confidence_0.000_Length_1860, tri_c_comp37156_c0_seq1, vo_c1_Locus_17180_Transcript_1/1_Confidence_0.000_Length_4157, vo_c2_Locus_11596_Transcript_2/3_Confidence_0.167_Length_1499, vo_n2_Locus_6369_Transcript_1/1_Confidence_0.000_Length_1901, vo_n2_Locus_8637_Transcript_3/3_Confidence_0.000_Length_1561, vo_c3_Locus_6073_Transcript_3/9_Confidence_0.400_Length_2122, vo_n1_Locus_15871_Transcript_1/1_Confidence_0.000_Length_1944, vo_c2_Locus_10095_Transcript_1/4_Confidence_0.286_Length_4859, vo_n3_Locus_25849_Transcript_1/7_Confidence_0.421_Length_1317, vo_c1_Locus_2758_Transcript_9/10_Confidence_0.267_Length_1451, tri_c_comp14506_c0_seq1, vo_n2_Locus_16855_Transcript_8/9_Confidence_0.636_Length_2285, tri_c_comp11242_c0_seq2, tri_n_comp64914_c0_seq1, vo_c2_Locus_9062_Transcript_10/11_Confidence_0.312_Length_4945, vo_n2_Locus_22224_Transcript_1/1_Confidence_0.000_Length_1370, tri_n_comp14915_c0_seq1, vo_n3_Locus_15713_Transcript_4/7_Confidence_0.429_Length_2557, tri_c_comp37399_c0_seq1, tri_n_comp58061_c0_seq1, vo_n1_Locus_22736_Transcript_1/2_Confidence_0.333_Length_1213, vo_n2_Locus_19677_Transcript_1/1_Confidence_0.000_Length_2823, tri_c_comp55340_c0_seq1, vo_n1_Locus_19864_Transcript_2/2_Confidence_0.000_Length_2707, vo_c3_Locus_21741_Transcript_5/9_Confidence_0.583_Length_1757, vo_n3_Locus_25600_Transcript_5/6_Confidence_0.375_Length_1777, tri_n_comp58061_c0_seq1, vo_c2_Locus_17150_Transcript_58/102_Confidence_0.019_Length_2519, vo_n2_Locus_11259_Transcript_1/2_Confidence_0.333_Length_2880, tri_c_comp44396_c0_seq1, vo_n2_Locus_31986_Transcript_2/2_Confidence_0.333_Length_2185, vo_n2_Locus_20375_Transcript_1/2_Confidence_0.333_Length_1577, vo_c2_Locus_1239_Transcript_4/4_Confidence_0.000_Length_1343, vo_n1_Locus_12743_Transcript_2/2_Confidence_0.000_Length_633, tri_c_comp9226_c0_seq1, tri_n_comp38992_c0_seq1, vo_n3_Locus_16690_Transcript_3/4_Confidence_0.667_Length_408, vo_n2_Locus_19192_Transcript_2/2_Confidence_0.250_Length_3053, vo_c2_Locus_16114_Transcript_1/1_Confidence_0.000_Length_1890, vo_n1_Locus_1454_Transcript_3/3_Confidence_0.000_Length_612, vo_c2_Locus_1239_Transcript_4/4_Confidence_0.000_Length_1343, vo_n2_Locus_19192_Transcript_2/2_Confidence_0.250_Length_3053, vo_n1_Locus_12743_Transcript_2/2_Confidence_0.000_Length_633, vo_n2_Locus_24861_Transcript_1/3_Confidence_0.714_Length_1018, tri_c_comp9226_c0_seq1, vo_c2_Locus_16114_Transcript_1/1_Confidence_0.000_Length_1890, vo_n2_Locus_4057_Transcript_1/1_Confidence_0.000_Length_1221, vo_n1_Locus_7235_Transcript_3/6_Confidence_0.625_Length_1162, vo_n3_Locus_14008_Transcript_1/1_Confidence_0.000_Length_2199, vo_n1_Locus_16218_Transcript_1/1_Confidence_0.000_Length_2119, vo_n2_Locus_7435_Transcript_1/1_Confidence_0.000_Length_1552, vo_c1_Locus_13556_Transcript_2/2_Confidence_0.000_Length_1297, tri_c_comp22527_c0_seq1, vo_n1_Locus_10504_Transcript_1/2_Confidence_0.750_Length_1211, vo_c2_Locus_9062_Transcript_1/11_Confidence_0.312_Length_4797

One carbon pool by folate	map00670	12	ec:6.3.3.2 - cyclo-ligase, ec:3.5.4.9 - cyclohydrolase, ec:2.1.1.45 - synthase, ec:2.1.1.13 - synthase, ec:1.5.1.3 - reductase, ec:1.5.1.5 - dehydrogenase (NADP+), ec:2.1.2.3 - formyltransferase, ec:2.1.2.2 - formyltransferase, ec:2.1.2.1 - hydroxymethyltransferase, ec:6.3.4.3 - ligase, ec:1.5.1.20 - reductase [NAD(P)H], ec:2.1.2.10 - S-aminomethyldihydrolipoylprotein:(6S)-tetrahydrofolate aminomethyltransferase (ammonia-forming)	9, 2, 5, 4, 1, 2, 8, 1, 9, 2, 1, 9	tri_n_comp18448_c0_seq7, vo_c3_Locus_3582_Transcript_8/9_Confidence_0.071_Length_1176, vo_c2_Locus_1080_Transcript_8/21_Confidence_0.138_Length_1077, tri_n_comp18448_c0_seq1, tri_n_comp18448_c0_seq2, tri_n_comp18448_c0_seq3, tri_n_comp18448_c0_seq4, vo_n2_Locus_26338_Transcript_1/2_Confidence_0.333_Length_1245, tri_n_comp18448_c0_seq6, tri_n_comp31819_c0_seq1, vo_n2_Locus_10494_Transcript_3/4_Confidence_0.333_Length_2314, tri_c_comp11590_c0_seq1, vo_c3_Locus_15578_Transcript_2/4_Confidence_0.722_Length_967, vo_c1_Locus_6843_Transcript_1/2_Confidence_0.833_Length_813, vo_c2_Locus_1969_Transcript_1/2_Confidence_0.200_Length_1807, vo_c1_Locus_6843_Transcript_2/2_Confidence_0.167_Length_995, vo_n3_Locus_12898_Transcript_5/14_Confidence_0.429_Length_3637, vo_n2_Locus_4083_Transcript_7/15_Confidence_0.571_Length_4447, vo_c3_Locus_876_Transcript_5/9_Confidence_0.526_Length_3245, vo_n2_Locus_4083_Transcript_2/15_Confidence_0.619_Length_4830, vo_c2_Locus_1969_Transcript_1/2_Confidence_0.200_Length_1807, vo_n2_Locus_2550_Transcript_2/3_Confidence_0.636_Length_1302, tri_n_comp31819_c0_seq1, vo_c1_Locus_1055_Transcript_9/11_Confidence_0.357_Length_2035, vo_n1_Locus_561_Transcript_9/10_Confidence_0.462_Length_2182, vo_n2_Locus_3954_Transcript_2/8_Confidence_0.478_Length_2452, vo_c3_Locus_289_Transcript_1/7_Confidence_0.381_Length_2114, vo_n2_Locus_3954_Transcript_4/8_Confidence_0.435_Length_2452, vo_n1_Locus_561_Transcript_7/10_Confidence_0.385_Length_2182, vo_c1_Locus_1055_Transcript_7/11_Confidence_0.357_Length_2063, vo_n1_Locus_561_Transcript_1/10_Confidence_0.615_Length_2142, vo_n2_Locus_3094_Transcript_1/1_Confidence_0.000_Length_2179, vo_c2_Locus_1465_Transcript_1/1_Confidence_0.000_Length_2684, vo_n2_Locus_668_Transcript_12/17_Confidence_0.465_Length_3343, vo_n3_Locus_1281_Transcript_13/14_Confidence_0.467_Length_2089, vo_n2_Locus_668_Transcript_10/17_Confidence_0.419_Length_1865, vo_c3_Locus_6604_Transcript_15/15_Confidence_0.590_Length_1792, vo_n3_Locus_1281_Transcript_3/14_Confidence_0.533_Length_1904, vo_c3_Locus_6604_Transcript_12/15_Confidence_0.230_Length_1729, tri_n_comp22443_c0_seq1, vo_c2_Locus_2191_Transcript_14/17_Confidence_0.464_Length_1841, tri_n_comp31819_c0_seq1, vo_n2_Locus_10494_Transcript_3/4_Confidence_0.333_Length_2314, vo_c2_Locus_1465_Transcript_1/1_Confidence_0.000_Length_2684, tri_c_comp25586_c0_seq1, vo_c2_Locus_23277_Transcript_1/1_Confidence_0.000_Length_1058, vo_n1_Locus_803_Transcript_2/31_Confidence_0.118_Length_2529, vo_n1_Locus_27713_Transcript_1/1_Confidence_0.000_Length_1034, tri_n_comp13806_c0_seq3, tri_c_comp13451_c0_seq2, vo_n1_Locus_803_Transcript_9/31_Confidence_0.324_Length_2360, vo_n2_Locus_3952_Transcript_1/2_Confidence_0.750_Length_1244, vo_n2_Locus_514_Transcript_37/44_Confidence_0.155_Length_1950

Nitrogen metabolism	map00910	12	ec:6.3.1.2 - synthetase, ec:1.4.7.1 - synthase (ferredoxin), ec:1.13.12.16 - monooxygenase, ec:1.7.1.1 - reductase (NADH), ec:1.7.1.4 - reductase [NAD(P)H], ec:1.4.1.2 - dehydrogenase, ec:4.2.1.1 - anhydrase, ec:3.5.5.1 - acetonitrilase, ec:6.3.4.16 - synthase (ammonia), ec:1.7.99.4 - reductase, ec:1.4.1.14 - synthase (NADH), ec:1.4.1.13 - synthase (NADPH)	15, 12, 2, 21, 3, 1, 5, 1, 2, 7, 15, 10	vo_c1_Locus_1340_Transcript_12/17_Confidence_0.109_Length_2389, vo_c1_Locus_1340_Transcript_9/17_Confidence_0.172_Length_2429, tri_n_comp11684_c0_seq1, vo_c1_Locus_4136_Transcript_1/2_Confidence_0.967_Length_2259, vo_c2_Locus_6408_Transcript_2/14_Confidence_0.268_Length_1478, vo_n1_Locus_335_Transcript_2/3_Confidence_0.909_Length_2286, vo_c2_Locus_3512_Transcript_3/4_Confidence_0.500_Length_2501, vo_n2_Locus_4820_Transcript_2/3_Confidence_0.545_Length_2376, vo_n3_Locus_2644_Transcript_11/11_Confidence_0.323_Length_2390, vo_n2_Locus_515_Transcript_8/9_Confidence_0.471_Length_3194, vo_n2_Locus_515_Transcript_7/9_Confidence_0.176_Length_2355, vo_c2_Locus_3512_Transcript_2/4_Confidence_0.750_Length_2320, vo_c3_Locus_1501_Transcript_6/9_Confidence_0.203_Length_2420, vo_n2_Locus_5127_Transcript_1/3_Confidence_0.200_Length_1636, vo_n2_Locus_22912_Transcript_1/2_Confidence_0.333_Length_2481, vo_c2_Locus_4368_Transcript_2/25_Confidence_0.375_Length_6775, vo_c1_Locus_13166_Transcript_27/32_Confidence_0.394_Length_6627, vo_n2_Locus_3_Transcript_20516/24291_Confidence_0.000_Length_6564, vo_c3_Locus_3082_Transcript_7/7_Confidence_0.167_Length_5439, vo_n1_Locus_991_Transcript_1/21_Confidence_0.113_Length_1473, tri_c_comp19198_c0_seq1, vo_c1_Locus_13166_Transcript_28/32_Confidence_0.409_Length_6630, vo_n1_Locus_7772_Transcript_1/1_Confidence_0.000_Length_6986, tri_c_comp19198_c0_seq4, tri_c_comp19198_c0_seq3, vo_n3_Locus_4402_Transcript_3/18_Confidence_0.269_Length_6798, vo_c3_Locus_3082_Transcript_5/7_Confidence_0.333_Length_2051, vo_c1_Locus_4368_Transcript_2/3_Confidence_0.333_Length_1374, vo_n2_Locus_5728_Transcript_1/2_Confidence_0.333_Length_1242, vo_n3_Locus_5844_Transcript_3/4_Confidence_0.091_Length_2683, vo_n2_Locus_9904_Transcript_14/17_Confidence_0.179_Length_436, vo_n3_Locus_1465_Transcript_5/6_Confidence_0.308_Length_662, vo_c1_Locus_4987_Transcript_9/13_Confidence_0.192_Length_474, vo_n1_Locus_3296_Transcript_15/19_Confidence_0.267_Length_1918, vo_c2_Locus_1186_Transcript_52/68_Confidence_0.072_Length_1585, vo_c3_Locus_1280_Transcript_27/33_Confidence_0.200_Length_480, vo_n1_Locus_3296_Transcript_18/19_Confidence_0.200_Length_528, tri_c_comp15887_c0_seq2, vo_n3_Locus_1465_Transcript_4/6_Confidence_0.692_Length_819, vo_c3_Locus_481_Transcript_40/75_Confidence_0.052_Length_1602, tri_n_comp21114_c0_seq1, vo_c2_Locus_1414_Transcript_6/9_Confidence_0.118_Length_478, tri_n_comp24956_c0_seq1, vo_n2_Locus_9904_Transcript_11/17_Confidence_0.205_Length_579, vo_c3_Locus_1280_Transcript_10/33_Confidence_0.273_Length_2453, vo_n2_Locus_9450_Transcript_1/2_Confidence_1.000_Length_2867, vo_n2_Locus_9904_Transcript_16/17_Confidence_0.231_Length_575, vo_c3_Locus_1280_Transcript_30/33_Confidence_0.109_Length_1151, vo_n1_Locus_3296_Transcript_8/19_Confidence_0.267_Length_743, vo_n2_Locus_9904_Transcript_8/17_Confidence_0.462_Length_876, vo_n2_Locus_128_Transcript_8/33_Confidence_0.085_Length_4154, vo_c3_Locus_2901_Transcript_35/41_Confidence_0.153_Length_4192, tri_c_comp58470_c0_seq1, tri_c_comp28026_c0_seq1, tri_c_comp25113_c0_seq1, vo_n3_Locus_15566_Transcript_4/4_Confidence_0.143_Length_781, vo_n2_Locus_9558_Transcript_1/1_Confidence_0.000_Length_2494, vo_n3_Locus_10160_Transcript_1/2_Confidence_0.958_Length_2574, tri_n_comp53048_c0_seq1, vo_c2_Locus_31357_Transcript_6/7_Confidence_0.700_Length_1464, tri_c_comp33901_c0_seq1, vo_n2_Locus_5805_Transcript_1/2_Confidence_0.333_Length_5227, vo_c2_Locus_13354_Transcript_6/9_Confidence_0.231_Length_2604, vo_c1_Locus_5503_Transcript_5/9_Confidence_0.400_Length_1997, tri_c_comp6632_c0_seq1, vo_c1_Locus_4987_Transcript_13/13_Confidence_0.115_Length_625, vo_c3_Locus_8831_Transcript_2/3_Confidence_0.333_Length_1264, vo_n1_Locus_5428_Transcript_1/1_Confidence_0.000_Length_885, vo_c3_Locus_1280_Transcript_24/33_Confidence_0.127_Length_2411, vo_c1_Locus_13166_Transcript_27/32_Confidence_0.394_Length_6627, vo_c3_Locus_3082_Transcript_7/7_Confidence_0.167_Length_5439, vo_n1_Locus_991_Transcript_1/21_Confidence_0.113_Length_1473, tri_c_comp19198_c0_seq1, vo_n3_Locus_4402_Transcript_3/18_Confidence_0.269_Length_6798, vo_c2_Locus_4368_Transcript_2/25_Confidence_0.375_Length_6775, vo_n2_Locus_3_Transcript_20516/24291_Confidence_0.000_Length_6564, vo_n3_Locus_4402_Transcript_4/18_Confidence_0.077_Length_491, vo_c1_Locus_13166_Transcript_10/32_Confidence_0.076_Length_628, vo_c1_Locus_13166_Transcript_28/32_Confidence_0.409_Length_6630, vo_n1_Locus_7772_Transcript_1/1_Confidence_0.000_Length_6986, tri_c_comp19198_c0_seq4, tri_c_comp19198_c0_seq3, vo_c3_Locus_3082_Transcript_5/7_Confidence_0.333_Length_2051, vo_c1_Locus_1115_Transcript_3/3_Confidence_0.000_Length_916, vo_c2_Locus_4368_Transcript_2/25_Confidence_0.375_Length_6775, vo_c1_Locus_13166_Transcript_27/32_Confidence_0.394_Length_6627, vo_n2_Locus_3_Transcript_20516/24291_Confidence_0.000_Length_6564, vo_c3_Locus_3082_Transcript_7/7_Confidence_0.167_Length_5439, vo_n1_Locus_991_Transcript_1/21_Confidence_0.113_Length_1473, tri_c_comp19198_c0_seq1, vo_c1_Locus_13166_Transcript_28/32_Confidence_0.409_Length_6630, vo_n1_Locus_7772_Transcript_1/1_Confidence_0.000_Length_6986, vo_n3_Locus_4402_Transcript_3/18_Confidence_0.269_Length_6798, vo_c3_Locus_3082_Transcript_5/7_Confidence_0.333_Length_2051

Tryptophan metabolism	map00380	11	ec:1.13.11.6 - 3,4-dioxygenase, ec:1.2.1.3 - dehydrogenase (NAD+), ec:2.3.1.9 - C-acetyltransferase, ec:1.14.16.4 - 5-monooxygenase, ec:3.5.1.4 - acylamidase, ec:3.5.1.9 - kynurenine formamidase, ec:3.7.1.3 - ec:3.7.1.3 kynureninase, ec:3.5.5.1 - acetonitrilase, ec:4.2.1.17 - hydratase, ec:1.1.1.35 - dehydrogenase, ec:1.2.4.2 - dehydrogenase (succinyl-transferring)	1, 5, 2, 1, 3, 2, 1, 1, 7, 16, 2	vo_n2_Locus_11170_Transcript_1/1_Confidence_0.000_Length_1117, tri_c_comp55340_c0_seq1, vo_n1_Locus_19864_Transcript_2/2_Confidence_0.000_Length_2707, vo_c3_Locus_21741_Transcript_5/9_Confidence_0.583_Length_1757, vo_n3_Locus_25600_Transcript_5/6_Confidence_0.375_Length_1777, tri_n_comp58061_c0_seq1, vo_c1_Locus_2657_Transcript_1/1_Confidence_0.000_Length_1513, vo_n2_Locus_23058_Transcript_1/1_Confidence_0.000_Length_1342, vo_c2_Locus_16025_Transcript_2/3_Confidence_0.400_Length_2217, vo_c3_Locus_1581_Transcript_3/3_Confidence_0.000_Length_1736, vo_c1_Locus_1298_Transcript_3/6_Confidence_0.625_Length_1775, vo_n2_Locus_157_Transcript_2/4_Confidence_0.286_Length_2059, vo_n2_Locus_10311_Transcript_5/6_Confidence_0.357_Length_778, vo_n2_Locus_10311_Transcript_2/6_Confidence_0.500_Length_1843, vo_c3_Locus_23248_Transcript_1/1_Confidence_0.000_Length_1653, vo_c2_Locus_31357_Transcript_6/7_Confidence_0.700_Length_1464, vo_c2_Locus_2261_Transcript_1/2_Confidence_1.000_Length_2564, vo_c3_Locus_5948_Transcript_1/1_Confidence_0.000_Length_1027, vo_n2_Locus_16945_Transcript_1/3_Confidence_0.500_Length_1084, vo_c3_Locus_9925_Transcript_8/11_Confidence_0.263_Length_3550, vo_n1_Locus_11137_Transcript_5/5_Confidence_0.143_Length_2829, vo_n2_Locus_16945_Transcript_3/3_Confidence_0.000_Length_746, vo_n2_Locus_30747_Transcript_1/1_Confidence_0.000_Length_951, vo_c2_Locus_2261_Transcript_1/2_Confidence_1.000_Length_2564, vo_n1_Locus_9414_Transcript_8/13_Confidence_0.167_Length_606, vo_c1_Locus_40339_Transcript_1/1_Confidence_0.000_Length_1318, vo_c3_Locus_2031_Transcript_8/8_Confidence_0.111_Length_1389, vo_n1_Locus_11137_Transcript_5/5_Confidence_0.143_Length_2829, vo_c1_Locus_4675_Transcript_3/3_Confidence_0.200_Length_1348, vo_n2_Locus_30747_Transcript_1/1_Confidence_0.000_Length_951, vo_n2_Locus_636_Transcript_13/16_Confidence_0.222_Length_768, vo_c1_Locus_2815_Transcript_3/4_Confidence_0.200_Length_2244, vo_c3_Locus_9925_Transcript_8/11_Confidence_0.263_Length_3550, tri_n_comp42565_c0_seq1, vo_c2_Locus_189_Transcript_29/60_Confidence_0.015_Length_692, vo_n2_Locus_636_Transcript_16/16_Confidence_0.139_Length_616, vo_n2_Locus_636_Transcript_14/16_Confidence_0.222_Length_622, vo_n2_Locus_636_Transcript_5/16_Confidence_0.194_Length_591, vo_n2_Locus_636_Transcript_3/16_Confidence_0.306_Length_685, vo_n2_Locus_3831_Transcript_3/5_Confidence_0.286_Length_3559, vo_c2_Locus_13441_Transcript_1/2_Confidence_0.333_Length_3397

beta-Alanine metabolism	map00410	11	ec:2.6.1.18 - transaminase, ec:2.6.1.19 - transaminase, ec:2.5.1.16 - synthase, ec:2.5.1.22 - synthase, ec:1.2.1.18 - dehydrogenase (acetylating), ec:1.2.1.5 - dehydrogenase [NAD(P)+], ec:1.2.1.3 - dehydrogenase (NAD+), ec:4.1.1.9 - decarboxylase, ec:3.1.2.4 - hydrolase, ec:4.2.1.17 - hydratase, ec:1.4.3.21 - oxidase	1, 2, 1, 2, 3, 2, 5, 1, 1, 7, 1	vo_c1_Locus_7220_Transcript_2/3_Confidence_0.400_Length_2898, vo_c3_Locus_29526_Transcript_7/10_Confidence_0.268_Length_874, vo_c2_Locus_844_Transcript_5/40_Confidence_0.162_Length_2081, vo_c2_Locus_25147_Transcript_1/2_Confidence_0.333_Length_1097, vo_c2_Locus_25147_Transcript_1/2_Confidence_0.333_Length_1097, tri_c_comp23701_c0_seq1, vo_c3_Locus_1581_Transcript_3/3_Confidence_0.000_Length_1736, vo_c1_Locus_1298_Transcript_3/6_Confidence_0.625_Length_1775, vo_n2_Locus_157_Transcript_2/4_Confidence_0.286_Length_2059, tri_c_comp55340_c0_seq1, tri_n_comp58061_c0_seq1, tri_c_comp55340_c0_seq1, vo_n1_Locus_19864_Transcript_2/2_Confidence_0.000_Length_2707, vo_c3_Locus_21741_Transcript_5/9_Confidence_0.583_Length_1757, vo_n3_Locus_25600_Transcript_5/6_Confidence_0.375_Length_1777, tri_n_comp58061_c0_seq1, vo_c2_Locus_22623_Transcript_1/1_Confidence_0.000_Length_1765, vo_c3_Locus_6234_Transcript_1/1_Confidence_0.000_Length_1326, vo_c2_Locus_2261_Transcript_1/2_Confidence_1.000_Length_2564, vo_c3_Locus_5948_Transcript_1/1_Confidence_0.000_Length_1027, vo_n2_Locus_16945_Transcript_1/3_Confidence_0.500_Length_1084, vo_c3_Locus_9925_Transcript_8/11_Confidence_0.263_Length_3550, vo_n1_Locus_11137_Transcript_5/5_Confidence_0.143_Length_2829, vo_n2_Locus_16945_Transcript_3/3_Confidence_0.000_Length_746, vo_n2_Locus_30747_Transcript_1/1_Confidence_0.000_Length_951, tri_n_comp48625_c0_seq1

Fatty acid degradation	map00071	11	ec:5.1.2.3 - epimerase, ec:6.2.1.3 - ligase, ec:4.2.1.74 - hydratase, ec:5.3.3.8 - isomerase, ec:1.2.1.3 - dehydrogenase (NAD+), ec:2.3.1.9 - C-acetyltransferase, ec:1.3.3.6 - oxidase, ec:1.3.8.1 - acyl-CoA dehydrogenase, ec:1.14.15.3 - 1-monooxygenase, ec:4.2.1.17 - hydratase, ec:1.1.1.35 - dehydrogenase	2, 6, 11, 2, 5, 2, 2, 2, 1, 7, 16	vo_n1_Locus_11137_Transcript_5/5_Confidence_0.143_Length_2829, vo_c3_Locus_9925_Transcript_8/11_Confidence_0.263_Length_3550, vo_c2_Locus_5191_Transcript_21/21_Confidence_0.029_Length_509, vo_c3_Locus_1178_Transcript_1/1_Confidence_0.667_Length_2367, vo_c2_Locus_3586_Transcript_1/4_Confidence_0.667_Length_2244, vo_c2_Locus_5191_Transcript_16/21_Confidence_0.457_Length_3699, tri_c_comp29321_c0_seq1, tri_c_comp48942_c0_seq1, vo_c2_Locus_2261_Transcript_1/2_Confidence_1.000_Length_2564, vo_n1_Locus_9414_Transcript_8/13_Confidence_0.167_Length_606, vo_c3_Locus_9925_Transcript_8/11_Confidence_0.263_Length_3550, vo_c2_Locus_189_Transcript_29/60_Confidence_0.015_Length_692, vo_c3_Locus_2031_Transcript_8/8_Confidence_0.111_Length_1389, vo_n2_Locus_636_Transcript_16/16_Confidence_0.139_Length_616, vo_n2_Locus_636_Transcript_14/16_Confidence_0.222_Length_622, vo_n2_Locus_636_Transcript_5/16_Confidence_0.194_Length_591, vo_n2_Locus_636_Transcript_3/16_Confidence_0.306_Length_685, vo_n2_Locus_30747_Transcript_1/1_Confidence_0.000_Length_951, vo_n2_Locus_636_Transcript_13/16_Confidence_0.222_Length_768, vo_n1_Locus_11137_Transcript_5/5_Confidence_0.143_Length_2829, vo_c3_Locus_9925_Transcript_8/11_Confidence_0.263_Length_3550, tri_c_comp55340_c0_seq1, vo_n1_Locus_19864_Transcript_2/2_Confidence_0.000_Length_2707, vo_c3_Locus_21741_Transcript_5/9_Confidence_0.583_Length_1757, vo_n3_Locus_25600_Transcript_5/6_Confidence_0.375_Length_1777, tri_n_comp58061_c0_seq1, vo_c1_Locus_2657_Transcript_1/1_Confidence_0.000_Length_1513, vo_n2_Locus_23058_Transcript_1/1_Confidence_0.000_Length_1342, tri_n_comp36670_c0_seq1, vo_n1_Locus_23625_Transcript_1/1_Confidence_0.000_Length_2286, tri_c_comp7217_c0_seq2, vo_c3_Locus_18248_Transcript_1/2_Confidence_0.667_Length_1596, vo_n2_Locus_2042_Transcript_5/7_Confidence_0.231_Length_2650, vo_c2_Locus_2261_Transcript_1/2_Confidence_1.000_Length_2564, vo_c3_Locus_5948_Transcript_1/1_Confidence_0.000_Length_1027, vo_n2_Locus_16945_Transcript_1/3_Confidence_0.500_Length_1084, vo_c3_Locus_9925_Transcript_8/11_Confidence_0.263_Length_3550, vo_n1_Locus_11137_Transcript_5/5_Confidence_0.143_Length_2829, vo_n2_Locus_16945_Transcript_3/3_Confidence_0.000_Length_746, vo_n2_Locus_30747_Transcript_1/1_Confidence_0.000_Length_951, vo_c2_Locus_2261_Transcript_1/2_Confidence_1.000_Length_2564, vo_n1_Locus_9414_Transcript_8/13_Confidence_0.167_Length_606, vo_c1_Locus_40339_Transcript_1/1_Confidence_0.000_Length_1318, vo_c3_Locus_2031_Transcript_8/8_Confidence_0.111_Length_1389, vo_n1_Locus_11137_Transcript_5/5_Confidence_0.143_Length_2829, vo_c1_Locus_4675_Transcript_3/3_Confidence_0.200_Length_1348, vo_n2_Locus_30747_Transcript_1/1_Confidence_0.000_Length_951, vo_n2_Locus_636_Transcript_13/16_Confidence_0.222_Length_768, vo_c1_Locus_2815_Transcript_3/4_Confidence_0.200_Length_2244, vo_c3_Locus_9925_Transcript_8/11_Confidence_0.263_Length_3550, tri_n_comp42565_c0_seq1, vo_c2_Locus_189_Transcript_29/60_Confidence_0.015_Length_692, vo_n2_Locus_636_Transcript_16/16_Confidence_0.139_Length_616, vo_n2_Locus_636_Transcript_14/16_Confidence_0.222_Length_622, vo_n2_Locus_636_Transcript_5/16_Confidence_0.194_Length_591, vo_n2_Locus_636_Transcript_3/16_Confidence_0.306_Length_685

Lysine degradation	map00310	11	ec:2.6.1.48 - transaminase, ec:2.1.1.43 - N-methyltransferase, ec:1.2.1.3 - dehydrogenase (NAD+), ec:2.3.1.9 - C-acetyltransferase, ec:1.5.1.8 - dehydrogenase (NADP+, L-lysine-forming), ec:1.5.1.9 - dehydrogenase (NAD+, L-glutamate-forming), ec:2.3.1.61 - succinyltransferase, ec:2.3.1.32 - N-acetyltransferase, ec:4.2.1.17 - hydratase, ec:1.1.1.35 - dehydrogenase, ec:1.2.4.2 - dehydrogenase (succinyl-transferring)	3, 77, 5, 2, 6, 6, 3, 21, 7, 16, 2	vo_c3_Locus_29526_Transcript_7/10_Confidence_0.268_Length_874, vo_c2_Locus_844_Transcript_5/40_Confidence_0.162_Length_2081, vo_c2_Locus_844_Transcript_6/40_Confidence_0.010_Length_1778, vo_n3_Locus_18095_Transcript_1/2_Confidence_0.333_Length_2906, vo_n2_Locus_470_Transcript_6/22_Confidence_0.514_Length_1171, tri_c_comp17599_c0_seq1, vo_n3_Locus_270_Transcript_17/29_Confidence_0.365_Length_3911, tri_c_comp17599_c0_seq3, tri_n_comp11833_c0_seq2, vo_c2_Locus_1219_Transcript_20/32_Confidence_0.384_Length_1442, vo_n3_Locus_3645_Transcript_1/4_Confidence_0.667_Length_2197, vo_c3_Locus_24936_Transcript_7/10_Confidence_0.400_Length_1560, vo_n1_Locus_10100_Transcript_3/3_Confidence_0.200_Length_3547, vo_c3_Locus_9140_Transcript_1/1_Confidence_0.000_Length_1391, vo_c1_Locus_5654_Transcript_1/3_Confidence_0.200_Length_1034, vo_n2_Locus_8440_Transcript_2/5_Confidence_0.375_Length_590, vo_n3_Locus_270_Transcript_18/29_Confidence_0.333_Length_1131, tri_c_comp16127_c0_seq2, tri_n_comp47054_c0_seq1, vo_c1_Locus_8919_Transcript_1/7_Confidence_0.667_Length_853, vo_n2_Locus_470_Transcript_17/22_Confidence_0.343_Length_1148, tri_n_comp19581_c0_seq4, vo_n2_Locus_470_Transcript_22/22_Confidence_0.143_Length_1080, vo_c2_Locus_108_Transcript_22/41_Confidence_0.124_Length_2147, vo_c2_Locus_1219_Transcript_30/32_Confidence_0.123_Length_997, vo_n1_Locus_2341_Transcript_4/10_Confidence_0.071_Length_978, tri_n_comp13606_c0_seq4, tri_n_comp17862_c0_seq6, vo_n1_Locus_5991_Transcript_1/3_Confidence_0.400_Length_1552, vo_c1_Locus_509_Transcript_5/34_Confidence_0.145_Length_1149, vo_n3_Locus_270_Transcript_3/29_Confidence_0.270_Length_1101, vo_c2_Locus_1219_Transcript_22/32_Confidence_0.260_Length_1159, vo_c2_Locus_19809_Transcript_1/6_Confidence_0.684_Length_1615, tri_c_comp34836_c0_seq1, vo_n1_Locus_98_Transcript_8/32_Confidence_0.226_Length_924, tri_c_comp29972_c0_seq1, vo_c2_Locus_1165_Transcript_4/5_Confidence_0.667_Length_1401, vo_c1_Locus_21378_Transcript_7/8_Confidence_0.462_Length_1323, vo_n2_Locus_22747_Transcript_1/1_Confidence_0.000_Length_2618, vo_c2_Locus_17523_Transcript_1/1_Confidence_0.667_Length_2434, vo_n2_Locus_13080_Transcript_1/2_Confidence_0.667_Length_2183, vo_c2_Locus_1219_Transcript_26/32_Confidence_0.247_Length_1220, vo_c3_Locus_4006_Transcript_5/32_Confidence_0.244_Length_1136, vo_c3_Locus_2969_Transcript_2/8_Confidence_0.600_Length_2506, vo_n1_Locus_38620_Transcript_1/1_Confidence_0.000_Length_978, vo_c3_Locus_114_Transcript_4/36_Confidence_0.138_Length_3659, vo_n2_Locus_17424_Transcript_1/1_Confidence_0.000_Length_963, vo_n2_Locus_470_Transcript_5/22_Confidence_0.257_Length_986, vo_n1_Locus_15083_Transcript_9/13_Confidence_0.571_Length_1739, vo_c2_Locus_33430_Transcript_1/1_Confidence_0.000_Length_1282, vo_n1_Locus_25627_Transcript_1/1_Confidence_0.000_Length_2707, vo_n2_Locus_1282_Transcript_4/9_Confidence_0.190_Length_1582, vo_n3_Locus_19627_Transcript_6/12_Confidence_0.600_Length_2746, vo_c2_Locus_1219_Transcript_14/32_Confidence_0.260_Length_1092, vo_n2_Locus_28392_Transcript_1/2_Confidence_0.750_Length_1804, tri_n_comp15137_c0_seq2, vo_c1_Locus_23005_Transcript_1/2_Confidence_0.667_Length_3051, vo_n2_Locus_20001_Transcript_1/1_Confidence_0.000_Length_1186, vo_c1_Locus_509_Transcript_32/34_Confidence_0.174_Length_1077, vo_c3_Locus_33563_Transcript_1/1_Confidence_0.000_Length_1595, tri_c_comp13959_c0_seq2, vo_c1_Locus_23962_Transcript_3/4_Confidence_0.500_Length_3209, tri_c_comp19627_c0_seq1, vo_c1_Locus_22781_Transcript_2/2_Confidence_0.000_Length_1365, tri_c_comp18282_c0_seq7, vo_c1_Locus_5206_Transcript_2/3_Confidence_0.400_Length_1079, tri_c_comp18292_c0_seq3, vo_c1_Locus_32369_Transcript_1/1_Confidence_0.000_Length_1831, vo_n2_Locus_28392_Transcript_2/2_Confidence_0.000_Length_1844, vo_c2_Locus_1219_Transcript_8/32_Confidence_0.274_Length_1040, vo_c3_Locus_114_Transcript_5/36_Confidence_0.008_Length_2112, tri_c_comp18282_c0_seq4, tri_c_comp37737_c0_seq1, vo_n2_Locus_8614_Transcript_11/11_Confidence_0.000_Length_593, vo_n2_Locus_931_Transcript_14/40_Confidence_0.143_Length_1853, vo_n3_Locus_270_Transcript_10/29_Confidence_0.317_Length_1136, tri_c_comp12242_c0_seq1, vo_c1_Locus_37089_Transcript_1/1_Confidence_0.000_Length_1447, vo_c3_Locus_37354_Transcript_1/1_Confidence_0.000_Length_673, vo_c1_Locus_8919_Transcript_6/7_Confidence_0.222_Length_969, tri_c_comp55340_c0_seq1, vo_n1_Locus_19864_Transcript_2/2_Confidence_0.000_Length_2707, vo_c3_Locus_21741_Transcript_5/9_Confidence_0.583_Length_1757, vo_n3_Locus_25600_Transcript_5/6_Confidence_0.375_Length_1777, tri_n_comp58061_c0_seq1, vo_c1_Locus_2657_Transcript_1/1_Confidence_0.000_Length_1513, vo_n2_Locus_23058_Transcript_1/1_Confidence_0.000_Length_1342, vo_n2_Locus_1937_Transcript_10/11_Confidence_0.615_Length_3407, vo_n1_Locus_1386_Transcript_4/9_Confidence_0.688_Length_3621, vo_n1_Locus_1386_Transcript_9/9_Confidence_0.250_Length_2403, vo_n1_Locus_1386_Transcript_5/9_Confidence_0.625_Length_3599, vo_n1_Locus_1386_Transcript_7/9_Confidence_0.438_Length_3591, vo_n3_Locus_12585_Transcript_4/4_Confidence_0.000_Length_3579, vo_n2_Locus_1937_Transcript_10/11_Confidence_0.615_Length_3407, vo_n1_Locus_1386_Transcript_4/9_Confidence_0.688_Length_3621, vo_n1_Locus_1386_Transcript_9/9_Confidence_0.250_Length_2403, vo_n1_Locus_1386_Transcript_5/9_Confidence_0.625_Length_3599, vo_n1_Locus_1386_Transcript_7/9_Confidence_0.438_Length_3591, vo_n3_Locus_12585_Transcript_4/4_Confidence_0.000_Length_3579, vo_n2_Locus_3821_Transcript_2/4_Confidence_0.750_Length_1813, tri_c_comp16609_c0_seq3, vo_c3_Locus_278_Transcript_151/322_Confidence_0.010_Length_2531, vo_c1_Locus_983_Transcript_13/109_Confidence_0.146_Length_3135, vo_n3_Locus_116_Transcript_56/74_Confidence_0.131_Length_1163, vo_n3_Locus_116_Transcript_63/74_Confidence_0.131_Length_1150, vo_c3_Locus_5_Transcript_290/353_Confidence_0.015_Length_1363, vo_n3_Locus_116_Transcript_59/74_Confidence_0.152_Length_2999, vo_n3_Locus_116_Transcript_61/74_Confidence_0.106_Length_3026, vo_c2_Locus_1312_Transcript_38/70_Confidence_0.205_Length_3683, tri_n_comp20073_c0_seq1, vo_n3_Locus_116_Transcript_62/74_Confidence_0.111_Length_2946, vo_c1_Locus_983_Transcript_106/109_Confidence_0.043_Length_1396, vo_n1_Locus_2751_Transcript_51/52_Confidence_0.033_Length_408, vo_n2_Locus_409_Transcript_4/53_Confidence_0.270_Length_2832, vo_n2_Locus_409_Transcript_31/53_Confidence_0.233_Length_3015, tri_n_comp20073_c0_seq2, vo_c3_Locus_5_Transcript_287/353_Confidence_0.027_Length_2825, vo_c3_Locus_5_Transcript_272/353_Confidence_0.003_Length_394,

					vo_c3_Locus_5_Transcript_283/353_Confidence_0.027_Length_3003, vo_c3_Locus_5_Transcript_286/353_Confidence_0.029_Length_2529, vo_n3_Locus_116_Transcript_60/74_Confidence_0.167_Length_2999, vo_n3_Locus_116_Transcript_48/74_Confidence_0.096_Length_1128, vo_c3_Locus_5_Transcript_288/353_Confidence_0.019_Length_1679, vo_c2_Locus_2261_Transcript_1/2_Confidence_1.000_Length_2564, vo_c3_Locus_5948_Transcript_1/1_Confidence_0.000_Length_1027, vo_n2_Locus_16945_Transcript_1/3_Confidence_0.500_Length_1084, vo_c3_Locus_9925_Transcript_8/11_Confidence_0.263_Length_3550, vo_n1_Locus_11137_Transcript_5/5_Confidence_0.143_Length_2829, vo_n2_Locus_16945_Transcript_3/3_Confidence_0.000_Length_746, vo_n2_Locus_30747_Transcript_1/1_Confidence_0.000_Length_951, vo_c2_Locus_2261_Transcript_1/2_Confidence_1.000_Length_2564, vo_n1_Locus_9414_Transcript_8/13_Confidence_0.167_Length_606, vo_c1_Locus_40339_Transcript_1/1_Confidence_0.000_Length_1318, vo_c3_Locus_2031_Transcript_8/8_Confidence_0.111_Length_1389, vo_n1_Locus_11137_Transcript_5/5_Confidence_0.143_Length_2829, vo_c1_Locus_4675_Transcript_3/3_Confidence_0.200_Length_1348, vo_n2_Locus_30747_Transcript_1/1_Confidence_0.000_Length_951, vo_n2_Locus_636_Transcript_13/16_Confidence_0.222_Length_768, vo_c1_Locus_2815_Transcript_3/4_Confidence_0.200_Length_2244, vo_c3_Locus_9925_Transcript_8/11_Confidence_0.263_Length_3550, tri_n_comp42565_c0_seq1, vo_c2_Locus_189_Transcript_29/60_Confidence_0.015_Length_692, vo_n2_Locus_636_Transcript_16/16_Confidence_0.139_Length_616, vo_n2_Locus_636_Transcript_14/16_Confidence_0.222_Length_622, vo_n2_Locus_636_Transcript_5/16_Confidence_0.194_Length_591, vo_n2_Locus_636_Transcript_3/16_Confidence_0.306_Length_685, vo_n2_Locus_3831_Transcript_3/5_Confidence_0.286_Length_3559, vo_c2_Locus_13441_Transcript_1/2_Confidence_0.333_Length_3397

Ascorbate and aldarate metabolism	map00053	11	ec:2.7.1.43 - glucuronokinase (phosphorylating), ec:2.7.7.44 - uridylyltransferase, ec:1.1.3.8 - oxidase, ec:1.2.1.3 - dehydrogenase (NAD+), ec:2.4.1.17 - 1-naphthol glucuronyltransferase, ec:4.1.2.20 - aldolase, ec:1.8.5.1 - dehydrogenase (ascorbate), ec:1.3.2.3 - dehydrogenase, ec:1.1.1.19 - reductase, ec:1.1.1.22 - 6-dehydrogenase, ec:1.13.99.1 - oxygenase	2, 5, 1, 5, 13, 1, 1, 1, 2, 1, 7	tri_c_comp10536_c0_seq1, vo_c2_Locus_32726_Transcript_2/2_Confidence_0.000_Length_696, vo_n2_Locus_12563_Transcript_1/1_Confidence_0.000_Length_1886, vo_c1_Locus_21075_Transcript_1/3_Confidence_0.500_Length_1034, vo_n1_Locus_3509_Transcript_2/2_Confidence_0.000_Length_1894, tri_c_comp13085_c0_seq3, tri_c_comp54583_c0_seq1, vo_n2_Locus_15834_Transcript_1/1_Confidence_0.000_Length_1997, tri_c_comp55340_c0_seq1, vo_n1_Locus_19864_Transcript_2/2_Confidence_0.000_Length_2707, vo_c3_Locus_21741_Transcript_5/9_Confidence_0.583_Length_1757, vo_n3_Locus_25600_Transcript_5/6_Confidence_0.375_Length_1777, tri_n_comp58061_c0_seq1, tri_c_comp6209_c0_seq1, vo_c1_Locus_26340_Transcript_5/9_Confidence_0.600_Length_1699, tri_n_comp65990_c0_seq1, vo_n1_Locus_13372_Transcript_1/2_Confidence_0.333_Length_1687, vo_n2_Locus_34154_Transcript_3/3_Confidence_0.000_Length_2043, vo_n2_Locus_668_Transcript_17/17_Confidence_0.070_Length_2403, tri_c_comp58135_c0_seq1, vo_c3_Locus_16093_Transcript_1/1_Confidence_0.000_Length_1750, vo_n1_Locus_13372_Transcript_2/2_Confidence_0.000_Length_1314, vo_c2_Locus_24841_Transcript_3/3_Confidence_0.167_Length_3241, vo_n2_Locus_4774_Transcript_1/1_Confidence_0.000_Length_884, vo_c1_Locus_35615_Transcript_1/1_Confidence_0.000_Length_2075, vo_n1_Locus_15639_Transcript_1/1_Confidence_0.000_Length_1086, vo_c2_Locus_24762_Transcript_1/5_Confidence_0.625_Length_3436, tri_n_comp19474_c0_seq4, vo_n2_Locus_15834_Transcript_1/1_Confidence_0.000_Length_1997, vo_c1_Locus_13556_Transcript_2/2_Confidence_0.000_Length_1297, vo_n1_Locus_10504_Transcript_1/2_Confidence_0.750_Length_1211, vo_c1_Locus_11739_Transcript_1/3_Confidence_0.929_Length_1730, vo_c2_Locus_7343_Transcript_5/6_Confidence_0.333_Length_1107, vo_c1_Locus_16134_Transcript_10/12_Confidence_0.200_Length_544, vo_c3_Locus_9233_Transcript_4/5_Confidence_0.375_Length_1432, vo_c2_Locus_7343_Transcript_6/6_Confidence_0.083_Length_1098, vo_c1_Locus_16134_Transcript_6/12_Confidence_0.250_Length_448, vo_c1_Locus_16134_Transcript_8/12_Confidence_0.150_Length_721, vo_c1_Locus_16134_Transcript_7/12_Confidence_0.200_Length_748

Steroid hormone biosynthesis	map00140	10	ec:2.1.1.6 - O-methyltransferase, ec:1.1.1.145 - dehydrogenase, ec:2.4.1.17 - 1-naphthol glucuronyltransferase, ec:1.1.1.239 - dehydrogenase (NAD+), ec:1.14.15.6 - monooxygenase (side-chain-cleaving), ec:2.8.2.15 - sulfotransferase, ec:1.1.1.51 - 17)beta-hydroxysteroid dehydrogenase, ec:1.1.1.62 - 17-dehydrogenase, ec:3.1.6.1 - sulfatase, ec:3.1.6.2 - arylsulfatase	12, 2, 13, 1, 1, 1, 9, 1, 2, 1	vo_c1_Locus_19524_Transcript_9/12_Confidence_0.615_Length_3975, vo_n1_Locus_23463_Transcript_3/4_Confidence_0.286_Length_1512, tri_c_comp32679_c0_seq1, tri_n_comp8749_c0_seq1, vo_c2_Locus_24901_Transcript_2/2_Confidence_0.000_Length_1355, vo_c3_Locus_26293_Transcript_1/4_Confidence_0.750_Length_2211, vo_n2_Locus_17803_Transcript_2/2_Confidence_0.250_Length_2793, tri_n_comp4208_c0_seq2, vo_n2_Locus_17803_Transcript_1/2_Confidence_0.500_Length_2823, vo_n3_Locus_12676_Transcript_1/1_Confidence_0.000_Length_3081, vo_c3_Locus_17106_Transcript_1/2_Confidence_1.000_Length_3041, vo_c3_Locus_26293_Transcript_2/4_Confidence_0.500_Length_2353, vo_c2_Locus_4806_Transcript_29/66_Confidence_0.103_Length_1536, vo_c3_Locus_11279_Transcript_9/10_Confidence_0.136_Length_1385, tri_c_comp6209_c0_seq1, vo_c1_Locus_26340_Transcript_5/9_Confidence_0.600_Length_1699, tri_n_comp65990_c0_seq1, vo_n1_Locus_13372_Transcript_1/2_Confidence_0.333_Length_1687, vo_n2_Locus_34154_Transcript_3/3_Confidence_0.000_Length_2043, vo_n2_Locus_668_Transcript_17/17_Confidence_0.070_Length_2403, tri_c_comp58135_c0_seq1, vo_c3_Locus_16093_Transcript_1/1_Confidence_0.000_Length_1750, vo_n1_Locus_13372_Transcript_2/2_Confidence_0.000_Length_1314, vo_c2_Locus_24841_Transcript_3/3_Confidence_0.167_Length_3241, vo_n2_Locus_4774_Transcript_1/1_Confidence_0.000_Length_884, vo_c1_Locus_35615_Transcript_1/1_Confidence_0.000_Length_2075, vo_n1_Locus_15639_Transcript_1/1_Confidence_0.000_Length_1086, vo_c1_Locus_2815_Transcript_3/4_Confidence_0.200_Length_2244, tri_c_comp28902_c0_seq1, vo_c3_Locus_1509_Transcript_1/2_Confidence_0.750_Length_1318, vo_n1_Locus_9414_Transcript_8/13_Confidence_0.167_Length_606, vo_c2_Locus_189_Transcript_29/60_Confidence_0.015_Length_692, vo_c3_Locus_2031_Transcript_8/8_Confidence_0.111_Length_1389, vo_n2_Locus_636_Transcript_16/16_Confidence_0.139_Length_616, vo_n2_Locus_636_Transcript_14/16_Confidence_0.222_Length_622, vo_n2_Locus_636_Transcript_5/16_Confidence_0.194_Length_591, vo_n2_Locus_636_Transcript_3/16_Confidence_0.306_Length_685, vo_n2_Locus_30747_Transcript_1/1_Confidence_0.000_Length_951, vo_n2_Locus_636_Transcript_13/16_Confidence_0.222_Length_768, vo_c1_Locus_2815_Transcript_3/4_Confidence_0.200_Length_2244, vo_n1_Locus_5276_Transcript_5/5_Confidence_0.000_Length_805, tri_c_comp17496_c0_seq2, vo_n1_Locus_5276_Transcript_5/5_Confidence_0.000_Length_805

Fatty acid biosynthesis	map00061	9	ec:6.4.1.2 - carboxylase, ec:1.1.1.100 - reductase, ec:6.2.1.3 - ligase, ec:4.2.1.59 - dehydratase, ec:3.1.2.14 - hydrolase, ec:1.3.1.9 - reductase (NADH), ec:2.3.1.85 - synthase, ec:2.3.1.39 - S-malonyltransferase, ec:2.3.1.41 - synthase I	16, 4, 6, 1, 1, 1, 12, 6, 3	vo_n3_Locus_484_Transcript_6/12_Confidence_0.250_Length_1533, vo_c3_Locus_17332_Transcript_1/1_Confidence_0.333_Length_6493, vo_n3_Locus_696_Transcript_20/35_Confidence_0.473_Length_5396, vo_c3_Locus_2164_Transcript_38/39_Confidence_0.292_Length_6879, tri_n_comp41552_c0_seq1, vo_c2_Locus_1509_Transcript_1/47_Confidence_0.149_Length_2731, vo_c2_Locus_2193_Transcript_1/3_Confidence_0.667_Length_1218, vo_c1_Locus_860_Transcript_10/29_Confidence_0.571_Length_6744, vo_c2_Locus_1509_Transcript_11/47_Confidence_0.164_Length_2686, vo_n2_Locus_1751_Transcript_51/59_Confidence_0.207_Length_3566, vo_n2_Locus_15097_Transcript_1/1_Confidence_0.000_Length_3935, vo_n3_Locus_484_Transcript_5/12_Confidence_0.250_Length_1451, vo_c1_Locus_860_Transcript_11/29_Confidence_0.571_Length_6741, tri_c_comp19913_c0_seq3, vo_n2_Locus_1751_Transcript_37/59_Confidence_0.195_Length_3437, vo_c2_Locus_1509_Transcript_6/47_Confidence_0.149_Length_2592, vo_n3_Locus_484_Transcript_6/12_Confidence_0.250_Length_1533, vo_c2_Locus_12502_Transcript_2/2_Confidence_0.000_Length_781, vo_n3_Locus_484_Transcript_5/12_Confidence_0.250_Length_1451, vo_c2_Locus_2193_Transcript_1/3_Confidence_0.667_Length_1218, vo_c2_Locus_5191_Transcript_21/21_Confidence_0.029_Length_509, vo_c3_Locus_1178_Transcript_1/1_Confidence_0.667_Length_2367, vo_c2_Locus_3586_Transcript_1/4_Confidence_0.667_Length_2244, vo_c2_Locus_5191_Transcript_16/21_Confidence_0.457_Length_3699, tri_c_comp29321_c0_seq1, tri_c_comp48942_c0_seq1, vo_c1_Locus_4631_Transcript_1/1_Confidence_0.000_Length_1088, vo_n2_Locus_22507_Transcript_2/2_Confidence_0.000_Length_2345, vo_c2_Locus_12009_Transcript_1/1_Confidence_0.000_Length_1400, vo_n1_Locus_6804_Transcript_2/3_Confidence_0.400_Length_3660, tri_c_comp9784_c0_seq2, tri_n_comp48567_c0_seq1, vo_c3_Locus_21966_Transcript_1/1_Confidence_0.000_Length_3835, vo_n2_Locus_7705_Transcript_1/1_Confidence_0.000_Length_3757, tri_c_comp37045_c0_seq1, tri_c_comp84696_c0_seq1, vo_c3_Locus_606_Transcript_7/16_Confidence_0.357_Length_3424, vo_n3_Locus_12727_Transcript_1/1_Confidence_0.000_Length_3602, vo_c3_Locus_30099_Transcript_6/9_Confidence_0.375_Length_2510, vo_c1_Locus_15119_Transcript_1/2_Confidence_0.333_Length_1463, vo_c3_Locus_3641_Transcript_5/5_Confidence_0.000_Length_2445, vo_c3_Locus_5147_Transcript_5/7_Confidence_0.688_Length_1975, vo_n2_Locus_6651_Transcript_2/2_Confidence_0.500_Length_2591, vo_c3_Locus_711_Transcript_3/3_Confidence_0.200_Length_1576, vo_n3_Locus_4536_Transcript_1/2_Confidence_0.333_Length_1865, vo_n1_Locus_6730_Transcript_2/6_Confidence_0.667_Length_1479, vo_c2_Locus_1667_Transcript_12/21_Confidence_0.122_Length_1452, vo_c3_Locus_422_Transcript_1/3_Confidence_0.857_Length_2914, vo_n1_Locus_8253_Transcript_1/1_Confidence_0.000_Length_1430, vo_n2_Locus_4712_Transcript_1/2_Confidence_0.333_Length_1867

Steroid biosynthesis	map00100	9	ec:1.3.1.72 - reductase, ec:2.5.1.21 - synthase, ec:2.1.1.41 - 24-C-methyltransferase, ec:5.3.3.5 - Delta-isomerase, ec:1.14.13.72 - monooxygenase, ec:3.1.1.13 - esterase, ec:2.3.1.26 - O-acyltransferase, ec:5.4.99.7 - synthase, ec:5.4.99.8 - synthase	1, 1, 2, 1, 1, 2, 1, 1, 1	tri_c_comp55318_c0_seq1, vo_n2_Locus_19315_Transcript_2/2_Confidence_0.000_Length_1802, vo_c2_Locus_11041_Transcript_1/2_Confidence_0.750_Length_1378, vo_c2_Locus_11041_Transcript_2/2_Confidence_0.000_Length_1411, vo_n1_Locus_8018_Transcript_1/1_Confidence_0.000_Length_852, vo_c1_Locus_12613_Transcript_1/1_Confidence_0.000_Length_2362, tri_c_comp14506_c0_seq1, vo_n2_Locus_16855_Transcript_8/9_Confidence_0.636_Length_2285, vo_n2_Locus_23058_Transcript_1/1_Confidence_0.000_Length_1342, vo_c2_Locus_33620_Transcript_1/1_Confidence_0.000_Length_2614, vo_c2_Locus_33620_Transcript_1/1_Confidence_0.000_Length_2614

Selenocompound metabolism	map00450	9	ec:1.8.1.9 - reductase, ec:2.1.1.13 - synthase, ec:4.4.1.8 - beta-lyase, ec:4.4.1.1 - gamma-lyase, ec:1.97.1.9 - reductase, ec:4.4.1.16 - lyase, ec:2.7.7.4 - adenylyltransferase, ec:2.7.9.3 - water dikinase, ec:6.1.1.10 - ligase	14, 4, 3, 7, 1, 4, 7, 2, 1	vo_n2_Locus_6953_Transcript_4/5_Confidence_0.444_Length_2181, vo_c2_Locus_5797_Transcript_1/2_Confidence_0.400_Length_1712, vo_c2_Locus_30373_Transcript_4/4_Confidence_0.000_Length_988, vo_c2_Locus_30373_Transcript_3/4_Confidence_0.667_Length_743, vo_n3_Locus_1380_Transcript_5/6_Confidence_0.273_Length_1217, tri_n_comp7648_c0_seq1, vo_n3_Locus_4780_Transcript_1/1_Confidence_0.000_Length_1276, vo_c1_Locus_3141_Transcript_7/36_Confidence_0.102_Length_2742, vo_n2_Locus_5095_Transcript_1/2_Confidence_0.667_Length_820, tri_n_comp63089_c0_seq1, tri_n_comp41310_c0_seq1, vo_c2_Locus_11806_Transcript_1/2_Confidence_0.333_Length_2149, vo_n2_Locus_6286_Transcript_1/1_Confidence_0.000_Length_1634, vo_c1_Locus_22833_Transcript_2/3_Confidence_0.500_Length_897, vo_n3_Locus_12898_Transcript_5/14_Confidence_0.429_Length_3637, vo_n2_Locus_4083_Transcript_7/15_Confidence_0.571_Length_4447, vo_c3_Locus_876_Transcript_5/9_Confidence_0.526_Length_3245, vo_n2_Locus_4083_Transcript_2/15_Confidence_0.619_Length_4830, vo_n2_Locus_7374_Transcript_2/3_Confidence_0.750_Length_1746, vo_c2_Locus_31468_Transcript_4/5_Confidence_0.667_Length_1579, vo_n1_Locus_3498_Transcript_1/3_Confidence_0.600_Length_1462, tri_c_comp42317_c0_seq1, vo_n2_Locus_7374_Transcript_2/3_Confidence_0.750_Length_1746, vo_n2_Locus_8711_Transcript_5/5_Confidence_0.067_Length_1558, vo_c2_Locus_11984_Transcript_2/6_Confidence_0.385_Length_1557, vo_c2_Locus_31468_Transcript_4/5_Confidence_0.667_Length_1579, vo_n1_Locus_3498_Transcript_1/3_Confidence_0.600_Length_1462, tri_c_comp30049_c0_seq1, vo_c2_Locus_5797_Transcript_1/2_Confidence_0.400_Length_1712, tri_n_comp37185_c0_seq1, vo_c1_Locus_38066_Transcript_1/1_Confidence_0.000_Length_1674, vo_c2_Locus_14495_Transcript_1/4_Confidence_0.714_Length_1830, vo_c3_Locus_9097_Transcript_2/4_Confidence_0.600_Length_3293, vo_n2_Locus_1711_Transcript_9/11_Confidence_0.320_Length_3674, vo_n2_Locus_1711_Transcript_8/11_Confidence_0.360_Length_2026, vo_c2_Locus_2008_Transcript_2/7_Confidence_0.231_Length_3495, vo_c1_Locus_18424_Transcript_1/2_Confidence_0.750_Length_906, tri_n_comp20038_c0_seq1, vo_n2_Locus_1711_Transcript_2/11_Confidence_0.280_Length_4019, vo_c1_Locus_18424_Transcript_2/2_Confidence_0.000_Length_956, vo_c3_Locus_7943_Transcript_1/2_Confidence_0.750_Length_1349, vo_n2_Locus_1308_Transcript_1/2_Confidence_1.000_Length_1311, vo_c1_Locus_27004_Transcript_1/1_Confidence_0.000_Length_2081

Sphingolipid metabolism	map00600	9	ec:3.2.1.45 - psychosine hydrolase, ec:3.5.1.23 - acylsphingosine deacylase, ec:3.2.1.22 - melibiase, ec:3.2.1.23 - lactase (ambiguous), ec:3.2.1.18 - neuraminidase, ec:2.3.1.50 - C-palmitoyltransferase, ec:2.8.2.11 - sulfotransferase, ec:3.1.6.8 - arylsulfatase A, ec:3.1.6.1 - sulfatase	4, 3, 5, 6, 16, 1, 31, 1, 2	tri_n_comp56355_c0_seq1, vo_n2_Locus_20092_Transcript_5/7_Confidence_0.636_Length_1860, vo_c3_Locus_14755_Transcript_5/5_Confidence_0.000_Length_1578, vo_n2_Locus_20092_Transcript_2/7_Confidence_0.364_Length_599, vo_n2_Locus_5568_Transcript_1/1_Confidence_0.000_Length_1060, tri_n_comp69072_c0_seq1, tri_c_comp66265_c0_seq1, vo_c2_Locus_17150_Transcript_58/102_Confidence_0.019_Length_2519, vo_n2_Locus_11259_Transcript_1/2_Confidence_0.333_Length_2880, tri_c_comp44396_c0_seq1, vo_n2_Locus_31986_Transcript_2/2_Confidence_0.333_Length_2185, vo_n2_Locus_20375_Transcript_1/2_Confidence_0.333_Length_1577, tri_c_comp12867_c0_seq1, vo_n2_Locus_1748_Transcript_2/3_Confidence_0.333_Length_4221, tri_n_comp70995_c0_seq1, vo_c2_Locus_26351_Transcript_1/2_Confidence_0.500_Length_4013, vo_c3_Locus_8047_Transcript_1/2_Confidence_0.333_Length_4021, vo_c3_Locus_32079_Transcript_2/2_Confidence_0.250_Length_2113, vo_c3_Locus_31_Transcript_21/79_Confidence_0.028_Length_1439, tri_n_comp18659_c0_seq1, vo_c3_Locus_9066_Transcript_2/6_Confidence_0.500_Length_1163, vo_c1_Locus_32359_Transcript_2/2_Confidence_0.000_Length_1561, vo_n3_Locus_1645_Transcript_4/7_Confidence_0.417_Length_2558, vo_c2_Locus_4429_Transcript_8/10_Confidence_0.385_Length_2463, tri_c_comp55333_c0_seq1, vo_n1_Locus_15621_Transcript_2/2_Confidence_0.000_Length_1596, vo_n3_Locus_21759_Transcript_2/5_Confidence_0.143_Length_653, vo_c2_Locus_4429_Transcript_7/10_Confidence_0.538_Length_2541, vo_n2_Locus_26415_Transcript_1/1_Confidence_0.000_Length_1376, vo_c2_Locus_6498_Transcript_10/10_Confidence_0.158_Length_1599, tri_n_comp15822_c0_seq1, vo_c1_Locus_27062_Transcript_5/7_Confidence_0.538_Length_1304, tri_n_comp15822_c0_seq2, vo_c3_Locus_9066_Transcript_1/6_Confidence_0.375_Length_846, vo_n1_Locus_7163_Transcript_2/5_Confidence_0.429_Length_3834, vo_c2_Locus_11068_Transcript_7/15_Confidence_0.088_Length_2478, tri_c_comp19434_c0_seq20, vo_n3_Locus_427_Transcript_152/175_Confidence_0.022_Length_1587, vo_n2_Locus_39296_Transcript_1/14_Confidence_0.615_Length_1447, vo_n1_Locus_1282_Transcript_21/28_Confidence_0.267_Length_1443, vo_c3_Locus_12992_Transcript_17/18_Confidence_0.438_Length_1753, vo_c3_Locus_10470_Transcript_2/7_Confidence_0.500_Length_1430, tri_c_comp18408_c0_seq3, vo_c3_Locus_15517_Transcript_3/3_Confidence_0.500_Length_1506, vo_c1_Locus_2146_Transcript_6/28_Confidence_0.438_Length_1427, tri_c_comp10922_c0_seq1, vo_n1_Locus_1282_Transcript_25/28_Confidence_0.250_Length_1445, vo_n1_Locus_1282_Transcript_18/28_Confidence_0.350_Length_1518, vo_c3_Locus_12992_Transcript_1/18_Confidence_0.562_Length_1707, vo_n1_Locus_1282_Transcript_20/28_Confidence_0.250_Length_1445, vo_n3_Locus_23180_Transcript_1/1_Confidence_0.000_Length_1372, vo_c3_Locus_12992_Transcript_2/18_Confidence_0.438_Length_1659, vo_n2_Locus_3_Transcript_18204/24291_Confidence_0.000_Length_1558, vo_c3_Locus_12992_Transcript_13/18_Confidence_0.562_Length_1789, vo_c3_Locus_15917_Transcript_1/2_Confidence_0.667_Length_1528, vo_c3_Locus_6108_Transcript_1/20_Confidence_0.667_Length_1501, vo_n2_Locus_1168_Transcript_12/19_Confidence_0.567_Length_1720, vo_c1_Locus_29045_Transcript_1/2_Confidence_0.600_Length_1495, vo_c3_Locus_12992_Transcript_14/18_Confidence_0.719_Length_1813, vo_c2_Locus_2351_Transcript_10/22_Confidence_0.081_Length_950, vo_c3_Locus_16912_Transcript_3/4_Confidence_0.500_Length_1461, vo_c2_Locus_557_Transcript_42/127_Confidence_0.063_Length_2107, vo_n1_Locus_1282_Transcript_28/28_Confidence_0.133_Length_1479, vo_c3_Locus_21516_Transcript_2/3_Confidence_0.333_Length_2294, vo_n2_Locus_4388_Transcript_1/1_Confidence_0.000_Length_1497, tri_n_comp20471_c0_seq2, vo_n1_Locus_5276_Transcript_5/5_Confidence_0.000_Length_805, vo_n1_Locus_5276_Transcript_5/5_Confidence_0.000_Length_805, tri_c_comp17496_c0_seq2

Riboflavin metabolism	map00740	9	ec:1.1.1.193 - reductase, ec:4.1.99.12 - synthase, ec:2.7.1.26 - kinase, ec:3.1.3.2 - phosphatase, ec:3.6.1.9 - diphosphatase, ec:2.7.7.2 - synthetase, ec:1.5.1.41 - reductase [NAD(P)H], ec:1.5.1.30 - reductase (NADPH), ec:3.5.4.25 - cyclohydrolase II	3, 7, 4, 12, 7, 4, 2, 2, 8	vo_n2_Locus_618_Transcript_2/13_Confidence_0.167_Length_2515, vo_c1_Locus_11577_Transcript_4/6_Confidence_0.222_Length_639, vo_c1_Locus_11577_Transcript_6/6_Confidence_0.000_Length_903, vo_c3_Locus_2926_Transcript_1/2_Confidence_1.000_Length_2091, vo_n3_Locus_3121_Transcript_2/3_Confidence_0.333_Length_1665, vo_n1_Locus_10359_Transcript_3/4_Confidence_0.444_Length_1674, vo_n2_Locus_5837_Transcript_4/4_Confidence_0.125_Length_2033, tri_n_comp29206_c0_seq1, vo_n1_Locus_4471_Transcript_1/4_Confidence_0.143_Length_1138, vo_n2_Locus_5837_Transcript_2/4_Confidence_0.500_Length_2218, vo_c2_Locus_1082_Transcript_2/3_Confidence_0.750_Length_1327, vo_n2_Locus_19354_Transcript_2/2_Confidence_0.000_Length_489, tri_c_comp14768_c0_seq1, vo_c1_Locus_23679_Transcript_3/3_Confidence_0.000_Length_1296, vo_n1_Locus_10075_Transcript_1/1_Confidence_0.000_Length_4386, tri_n_comp68385_c0_seq1, tri_c_comp18093_c0_seq1, vo_c1_Locus_25793_Transcript_1/2_Confidence_0.750_Length_1400, tri_n_comp42405_c0_seq1, vo_c1_Locus_11402_Transcript_1/1_Confidence_0.000_Length_1729, vo_c3_Locus_33014_Transcript_1/2_Confidence_0.750_Length_901, vo_c3_Locus_20317_Transcript_1/1_Confidence_0.000_Length_1353, vo_n1_Locus_39709_Transcript_1/1_Confidence_0.000_Length_555, vo_n2_Locus_2911_Transcript_3/13_Confidence_0.062_Length_1930, vo_n2_Locus_13424_Transcript_1/4_Confidence_0.667_Length_3514, vo_n1_Locus_10461_Transcript_1/2_Confidence_0.750_Length_1757, vo_c2_Locus_35825_Transcript_1/2_Confidence_0.500_Length_995, vo_c2_Locus_35825_Transcript_2/2_Confidence_0.000_Length_972, tri_n_comp34093_c0_seq1, vo_c2_Locus_11939_Transcript_1/4_Confidence_0.273_Length_1336, vo_c1_Locus_18906_Transcript_3/4_Confidence_0.400_Length_1298, vo_c2_Locus_26280_Transcript_1/1_Confidence_0.000_Length_1316, vo_c1_Locus_31652_Transcript_2/3_Confidence_0.429_Length_964, vo_c2_Locus_1082_Transcript_2/3_Confidence_0.750_Length_1327, vo_n2_Locus_19354_Transcript_2/2_Confidence_0.000_Length_489, tri_c_comp14768_c0_seq1, vo_c1_Locus_23679_Transcript_3/3_Confidence_0.000_Length_1296, vo_n2_Locus_14866_Transcript_1/2_Confidence_0.833_Length_1123, tri_c_comp43873_c0_seq1, vo_n2_Locus_14866_Transcript_1/2_Confidence_0.833_Length_1123, tri_c_comp43873_c0_seq1, vo_c3_Locus_2926_Transcript_1/2_Confidence_1.000_Length_2091, vo_n3_Locus_3121_Transcript_2/3_Confidence_0.333_Length_1665, vo_n2_Locus_2691_Transcript_2/12_Confidence_0.405_Length_3058, vo_n2_Locus_5837_Transcript_4/4_Confidence_0.125_Length_2033, tri_n_comp29206_c0_seq1, vo_n1_Locus_4471_Transcript_1/4_Confidence_0.143_Length_1138, vo_n2_Locus_5837_Transcript_2/4_Confidence_0.500_Length_2218, vo_n2_Locus_2691_Transcript_8/12_Confidence_0.310_Length_2836

Valine, leucine and isoleucine biosynthesis	map00290	8	ec:2.2.1.6 - synthase, ec:2.6.1.42 - transaminase, ec:4.3.1.19 - ammonia-lyase, ec:2.3.3.13 - synthase, ec:4.2.1.9 - dehydratase, ec:4.2.1.33 - dehydratase, ec:1.1.1.86 - reductoisomerase (NADP+), ec:1.1.1.85 - dehydrogenase	1, 6, 2, 1, 2, 1, 1, 2	tri_n_comp26150_c0_seq1, vo_c2_Locus_384_Transcript_2/3_Confidence_0.400_Length_1725, tri_c_comp32679_c0_seq1, vo_c1_Locus_10788_Transcript_1/1_Confidence_0.000_Length_1721, vo_c2_Locus_14969_Transcript_4/8_Confidence_0.750_Length_1231, vo_n2_Locus_3786_Transcript_2/4_Confidence_0.778_Length_1466, vo_n2_Locus_3786_Transcript_3/4_Confidence_0.667_Length_1442, vo_c2_Locus_30746_Transcript_1/1_Confidence_0.000_Length_1279, tri_c_comp34117_c0_seq1, vo_n1_Locus_2267_Transcript_1/1_Confidence_0.000_Length_2010, vo_n1_Locus_12155_Transcript_1/2_Confidence_0.500_Length_2024, vo_n2_Locus_484_Transcript_2/3_Confidence_0.333_Length_2007, vo_n2_Locus_1117_Transcript_16/36_Confidence_0.000_Length_2605, vo_n2_Locus_4139_Transcript_1/1_Confidence_0.000_Length_1669, vo_n1_Locus_6924_Transcript_4/6_Confidence_0.444_Length_1647, vo_n1_Locus_6924_Transcript_3/6_Confidence_0.222_Length_1163

N-Glycan biosynthesis	map00510	8	ec:2.4.1.260 - alpha-1,6-mannosyltransferase, ec:3.2.1.113 - 1,2-alpha-mannosidase, ec:2.4.1.38 - beta-1,4-galactosyltransferase, ec:3.2.1.106 - glucosidase, ec:2.4.1.101 - 2-beta-N-acetylglucosaminyltransferase, ec:2.4.1.144 - 4-beta-N-acetylglucosaminyltransferase, ec:2.4.1.155 - 6-beta-N-acetylglucosaminyltransferase, ec:2.4.99.1 - alpha-(2,6)-sialyltransferase	1, 8, 2, 2, 3, 3, 2, 6	tri_n_comp14001_c0_seq2, vo_n2_Locus_3852_Transcript_1/7_Confidence_0.417_Length_4358, tri_n_comp47354_c0_seq1, vo_n1_Locus_9004_Transcript_4/20_Confidence_0.171_Length_2905, vo_c1_Locus_37790_Transcript_1/1_Confidence_0.000_Length_3188, vo_c3_Locus_18796_Transcript_3/3_Confidence_0.000_Length_3826, vo_n1_Locus_24857_Transcript_1/1_Confidence_0.000_Length_2424, vo_c1_Locus_1525_Transcript_2/7_Confidence_0.167_Length_2762, vo_n2_Locus_5268_Transcript_1/2_Confidence_0.917_Length_1282, tri_c_comp20165_c0_seq7, tri_c_comp20165_c0_seq3, tri_n_comp51801_c0_seq1, vo_n3_Locus_2356_Transcript_2/3_Confidence_0.400_Length_7770, vo_c2_Locus_1239_Transcript_4/4_Confidence_0.000_Length_1343, tri_c_comp9226_c0_seq1, vo_n2_Locus_19192_Transcript_2/2_Confidence_0.250_Length_3053, vo_n2_Locus_4116_Transcript_2/13_Confidence_0.441_Length_1694, vo_c3_Locus_4339_Transcript_9/10_Confidence_0.310_Length_1598, vo_c2_Locus_18946_Transcript_11/11_Confidence_0.147_Length_1589, vo_n3_Locus_40148_Transcript_1/1_Confidence_0.000_Length_1773, vo_c3_Locus_14163_Transcript_1/2_Confidence_0.333_Length_2577, vo_n1_Locus_4002_Transcript_6/7_Confidence_0.091_Length_1369, tri_c_comp4119_c0_seq1, vo_n1_Locus_4002_Transcript_4/7_Confidence_0.636_Length_1286, vo_n2_Locus_15072_Transcript_4/7_Confidence_0.286_Length_628, tri_c_comp6583_c0_seq1, tri_n_comp6433_c0_seq1

Cyanoamino acid metabolism	map00460	8	ec:6.3.1.1 - ligase, ec:3.2.1.117 - beta-glucosidase, ec:3.2.1.118 - beta-glucosidase, ec:3.2.1.21 - gentiobiase, ec:3.5.1.1 - asparaginase II, ec:2.1.2.1 - hydroxymethyltransferase, ec:3.5.5.1 - acetonitrilase, ec:2.3.2.2 - glutamyl transpeptidase	1, 2, 2, 1, 3, 9, 1, 1	vo_n1_Locus_210_Transcript_2/3_Confidence_0.571_Length_2158, tri_c_comp12867_c0_seq1, tri_n_comp70995_c0_seq1, tri_c_comp12867_c0_seq1, tri_n_comp70995_c0_seq1, vo_c2_Locus_13105_Transcript_1/1_Confidence_0.000_Length_2797, tri_c_comp3336_c0_seq2, tri_n_comp8685_c0_seq2, tri_c_comp3336_c0_seq1, vo_c2_Locus_1465_Transcript_1/1_Confidence_0.000_Length_2684, vo_n2_Locus_668_Transcript_12/17_Confidence_0.465_Length_3343, vo_n3_Locus_1281_Transcript_13/14_Confidence_0.467_Length_2089, vo_n2_Locus_668_Transcript_10/17_Confidence_0.419_Length_1865, vo_c3_Locus_6604_Transcript_15/15_Confidence_0.590_Length_1792, vo_n3_Locus_1281_Transcript_3/14_Confidence_0.533_Length_1904, vo_c3_Locus_6604_Transcript_12/15_Confidence_0.230_Length_1729, tri_n_comp22443_c0_seq1, vo_c2_Locus_2191_Transcript_14/17_Confidence_0.464_Length_1841, vo_c2_Locus_31357_Transcript_6/7_Confidence_0.700_Length_1464, vo_n2_Locus_15602_Transcript_2/2_Confidence_0.250_Length_2162

Phenylalanine metabolism	map00360	8	ec:2.6.1.1 - transaminase, ec:1.2.1.5 - dehydrogenase [NAD(P)+], ec:1.14.16.1 - 4-monooxygenase, ec:3.5.1.4 - acylamidase, ec:2.1.1.104 - O-methyltransferase, ec:4.2.1.17 - hydratase, ec:5.3.2.1 - tautomerase, ec:1.4.3.21 - oxidase	4, 2, 8, 3, 2, 7, 3, 1	vo_n1_Locus_3423_Transcript_2/4_Confidence_0.250_Length_1347, tri_n_comp27683_c0_seq1, vo_c1_Locus_3127_Transcript_1/1_Confidence_0.000_Length_1478, vo_n1_Locus_3423_Transcript_3/4_Confidence_0.250_Length_1416, tri_c_comp55340_c0_seq1, tri_n_comp58061_c0_seq1, vo_c2_Locus_16025_Transcript_2/3_Confidence_0.400_Length_2217, vo_c3_Locus_4625_Transcript_6/11_Confidence_0.650_Length_2945, vo_c2_Locus_12196_Transcript_8/8_Confidence_0.133_Length_3092, vo_n1_Locus_3143_Transcript_9/11_Confidence_0.350_Length_2900, vo_c3_Locus_4625_Transcript_2/11_Confidence_0.350_Length_974, vo_c3_Locus_4625_Transcript_1/11_Confidence_0.350_Length_912, vo_c3_Locus_4625_Transcript_4/11_Confidence_0.650_Length_3056, vo_c3_Locus_4625_Transcript_3/11_Confidence_0.300_Length_1085, vo_c3_Locus_1581_Transcript_3/3_Confidence_0.000_Length_1736, vo_c1_Locus_1298_Transcript_3/6_Confidence_0.625_Length_1775, vo_n2_Locus_157_Transcript_2/4_Confidence_0.286_Length_2059, vo_n2_Locus_30574_Transcript_4/4_Confidence_0.000_Length_969, vo_n2_Locus_30574_Transcript_3/4_Confidence_0.500_Length_827, vo_c2_Locus_2261_Transcript_1/2_Confidence_1.000_Length_2564, vo_c3_Locus_5948_Transcript_1/1_Confidence_0.000_Length_1027, vo_n2_Locus_16945_Transcript_1/3_Confidence_0.500_Length_1084, vo_c3_Locus_9925_Transcript_8/11_Confidence_0.263_Length_3550, vo_n1_Locus_11137_Transcript_5/5_Confidence_0.143_Length_2829, vo_n2_Locus_16945_Transcript_3/3_Confidence_0.000_Length_746, vo_n2_Locus_30747_Transcript_1/1_Confidence_0.000_Length_951, vo_c2_Locus_19107_Transcript_2/4_Confidence_0.571_Length_752, vo_c1_Locus_20614_Transcript_4/6_Confidence_0.273_Length_420, vo_c2_Locus_19107_Transcript_1/4_Confidence_0.571_Length_831, tri_n_comp48625_c0_seq1

Tyrosine metabolism	map00350	8	ec:2.1.1.6 - O-methyltransferase, ec:1.2.1.16 - dehydrogenase [NAD(P)+], ec:2.6.1.1 - transaminase, ec:1.2.1.5 - dehydrogenase [NAD(P)+], ec:1.14.16.2 - 3-monooxygenase, ec:5.3.2.1 - tautomerase, ec:5.3.3.12 - isomerase, ec:1.4.3.21 - oxidase	12, 1, 4, 2, 2, 3, 3, 1	vo_c1_Locus_19524_Transcript_9/12_Confidence_0.615_Length_3975, vo_n1_Locus_23463_Transcript_3/4_Confidence_0.286_Length_1512, tri_c_comp32679_c0_seq1, tri_n_comp8749_c0_seq1, vo_c2_Locus_24901_Transcript_2/2_Confidence_0.000_Length_1355, vo_c3_Locus_26293_Transcript_1/4_Confidence_0.750_Length_2211, vo_n2_Locus_17803_Transcript_2/2_Confidence_0.250_Length_2793, tri_n_comp4208_c0_seq2, vo_n2_Locus_17803_Transcript_1/2_Confidence_0.500_Length_2823, vo_n3_Locus_12676_Transcript_1/1_Confidence_0.000_Length_3081, vo_c3_Locus_17106_Transcript_1/2_Confidence_1.000_Length_3041, vo_c3_Locus_26293_Transcript_2/4_Confidence_0.500_Length_2353, vo_c1_Locus_24159_Transcript_1/2_Confidence_0.333_Length_1573, vo_n1_Locus_3423_Transcript_2/4_Confidence_0.250_Length_1347, tri_n_comp27683_c0_seq1, vo_c1_Locus_3127_Transcript_1/1_Confidence_0.000_Length_1478, vo_n1_Locus_3423_Transcript_3/4_Confidence_0.250_Length_1416, tri_c_comp55340_c0_seq1, tri_n_comp58061_c0_seq1, vo_c2_Locus_16025_Transcript_2/3_Confidence_0.400_Length_2217, vo_n2_Locus_6286_Transcript_1/1_Confidence_0.000_Length_1634, vo_c2_Locus_19107_Transcript_2/4_Confidence_0.571_Length_752, vo_c1_Locus_20614_Transcript_4/6_Confidence_0.273_Length_420, vo_c2_Locus_19107_Transcript_1/4_Confidence_0.571_Length_831, vo_c2_Locus_19107_Transcript_2/4_Confidence_0.571_Length_752, vo_c1_Locus_20614_Transcript_4/6_Confidence_0.273_Length_420, vo_c2_Locus_19107_Transcript_1/4_Confidence_0.571_Length_831, tri_n_comp48625_c0_seq1

Sulfur metabolism	map00920	8	ec:2.7.1.25 - kinase, ec:1.8.4.8 - reductase (thioredoxin), ec:2.8.1.1 - sulfurtransferase, ec:2.7.7.4 - adenylyltransferase, ec:1.8.3.1 - oxidase, ec:2.3.1.30 - O-acetyltransferase, ec:2.3.1.46 - O-succinyltransferase, ec:2.5.1.47 - synthase	2, 3, 4, 7, 1, 4, 1, 5	vo_c1_Locus_18424_Transcript_2/2_Confidence_0.000_Length_956, vo_c1_Locus_18424_Transcript_1/2_Confidence_0.750_Length_906, tri_n_comp18266_c0_seq1, vo_c1_Locus_18284_Transcript_5/6_Confidence_0.545_Length_1691, tri_n_comp18266_c0_seq3, vo_n1_Locus_10960_Transcript_1/1_Confidence_0.000_Length_634, tri_c_comp17374_c0_seq1, vo_n2_Locus_25172_Transcript_1/1_Confidence_0.000_Length_1849, vo_n1_Locus_21114_Transcript_3/5_Confidence_0.444_Length_1255, vo_n2_Locus_1711_Transcript_9/11_Confidence_0.320_Length_3674, vo_n2_Locus_1711_Transcript_8/11_Confidence_0.360_Length_2026, vo_c2_Locus_2008_Transcript_2/7_Confidence_0.231_Length_3495, vo_c1_Locus_18424_Transcript_1/2_Confidence_0.750_Length_906, tri_n_comp20038_c0_seq1, vo_n2_Locus_1711_Transcript_2/11_Confidence_0.280_Length_4019, vo_c1_Locus_18424_Transcript_2/2_Confidence_0.000_Length_956, tri_c_comp6632_c0_seq1, vo_n1_Locus_9946_Transcript_1/3_Confidence_0.714_Length_1615, vo_c1_Locus_2886_Transcript_6/19_Confidence_0.481_Length_4535, vo_c2_Locus_4812_Transcript_2/3_Confidence_0.625_Length_1578, vo_c2_Locus_4812_Transcript_1/3_Confidence_0.625_Length_1831, vo_n1_Locus_9681_Transcript_11/11_Confidence_0.000_Length_529, vo_c2_Locus_2964_Transcript_1/2_Confidence_0.333_Length_1319, tri_c_comp42317_c0_seq1, vo_n2_Locus_211_Transcript_4/8_Confidence_0.400_Length_1517, vo_c1_Locus_1695_Transcript_1/2_Confidence_0.974_Length_1764, tri_n_comp16677_c0_seq1

Lysine biosynthesis	map00300	7	ec:2.6.1.17 - transaminase, ec:4.1.1.20 - decarboxylase, ec:2.7.2.4 - kinase, ec:1.2.1.11 - dehydrogenase, ec:1.17.1.8 - reductase, ec:1.1.1.3 - dehydrogenase, ec:4.3.3.7 - synthase	1, 2, 7, 4, 3, 1, 1	vo_c3_Locus_29526_Transcript_7/10_Confidence_0.268_Length_874, tri_c_comp30246_c0_seq1, vo_n1_Locus_454_Transcript_6/7_Confidence_0.364_Length_3522, vo_c1_Locus_1636_Transcript_120/122_Confidence_0.135_Length_3625, tri_n_comp29741_c0_seq1, vo_c3_Locus_31_Transcript_74/79_Confidence_0.124_Length_2769, vo_c2_Locus_10862_Transcript_1/1_Confidence_0.000_Length_1564, vo_c3_Locus_31_Transcript_63/79_Confidence_0.148_Length_3613, vo_c1_Locus_1636_Transcript_116/122_Confidence_0.129_Length_3526, vo_c2_Locus_52_Transcript_164/466_Confidence_0.011_Length_2810, vo_n2_Locus_3068_Transcript_2/3_Confidence_0.250_Length_1991, vo_n1_Locus_4025_Transcript_3/5_Confidence_0.286_Length_3475, vo_c1_Locus_9004_Transcript_2/4_Confidence_0.400_Length_3537, vo_c1_Locus_10440_Transcript_1/2_Confidence_0.667_Length_1569, vo_n1_Locus_32462_Transcript_1/1_Confidence_0.000_Length_1047, vo_n2_Locus_24260_Transcript_2/3_Confidence_0.250_Length_976, vo_c1_Locus_22485_Transcript_1/2_Confidence_0.500_Length_1035, vo_c2_Locus_10862_Transcript_1/1_Confidence_0.000_Length_1564, vo_c3_Locus_7185_Transcript_2/3_Confidence_0.333_Length_1661

Other glycan degradation	map00511	7	ec:3.2.1.45 - psychosine hydrolase, ec:3.2.1.52 - hexosaminidase, ec:3.5.1.26 - aspartylglucosylamine deaspartylase, ec:3.2.1.24 - alpha-D-mannosidase, ec:3.2.1.25 - mannanase, ec:3.2.1.23 - lactase (ambiguous), ec:3.2.1.18 - neuraminidase	4, 13, 1, 6, 2, 6, 16	tri_n_comp56355_c0_seq1, vo_n2_Locus_20092_Transcript_5/7_Confidence_0.636_Length_1860, vo_c3_Locus_14755_Transcript_5/5_Confidence_0.000_Length_1578, vo_n2_Locus_20092_Transcript_2/7_Confidence_0.364_Length_599, vo_c3_Locus_25279_Transcript_1/3_Confidence_0.500_Length_3460, tri_c_comp15706_c0_seq2, vo_n2_Locus_4554_Transcript_3/4_Confidence_0.444_Length_2253, vo_n2_Locus_3_Transcript_18735/24291_Confidence_0.000_Length_2121, vo_n2_Locus_4395_Transcript_3/3_Confidence_0.167_Length_2032, vo_c2_Locus_6406_Transcript_1/4_Confidence_0.333_Length_1666, vo_n1_Locus_14758_Transcript_1/2_Confidence_0.667_Length_1933, tri_n_comp35667_c0_seq1, vo_n2_Locus_4395_Transcript_1/3_Confidence_0.667_Length_1925, tri_c_comp32936_c0_seq1, tri_n_comp92955_c0_seq1, tri_n_comp30287_c0_seq1, vo_n2_Locus_4554_Transcript_4/4_Confidence_0.111_Length_2049, tri_n_comp8685_c0_seq2, vo_c1_Locus_28869_Transcript_6/6_Confidence_0.250_Length_506, vo_c1_Locus_28869_Transcript_4/6_Confidence_0.375_Length_1328, vo_c3_Locus_25165_Transcript_1/1_Confidence_0.000_Length_1760, vo_n2_Locus_19192_Transcript_2/2_Confidence_0.250_Length_3053, vo_c3_Locus_4662_Transcript_1/1_Confidence_0.000_Length_3632, tri_c_comp9226_c0_seq1, tri_c_comp12867_c0_seq1, tri_n_comp70995_c0_seq1, tri_c_comp12867_c0_seq1, vo_n2_Locus_1748_Transcript_2/3_Confidence_0.333_Length_4221, tri_n_comp70995_c0_seq1, vo_c2_Locus_26351_Transcript_1/2_Confidence_0.500_Length_4013, vo_c3_Locus_8047_Transcript_1/2_Confidence_0.333_Length_4021, vo_c3_Locus_32079_Transcript_2/2_Confidence_0.250_Length_2113, vo_c3_Locus_31_Transcript_21/79_Confidence_0.028_Length_1439, tri_n_comp18659_c0_seq1, vo_c3_Locus_9066_Transcript_2/6_Confidence_0.500_Length_1163, vo_c1_Locus_32359_Transcript_2/2_Confidence_0.000_Length_1561, vo_n3_Locus_1645_Transcript_4/7_Confidence_0.417_Length_2558, vo_c2_Locus_4429_Transcript_8/10_Confidence_0.385_Length_2463, tri_c_comp55333_c0_seq1, vo_n1_Locus_15621_Transcript_2/2_Confidence_0.000_Length_1596, vo_n3_Locus_21759_Transcript_2/5_Confidence_0.143_Length_653, vo_c2_Locus_4429_Transcript_7/10_Confidence_0.538_Length_2541, vo_n2_Locus_26415_Transcript_1/1_Confidence_0.000_Length_1376, vo_c2_Locus_6498_Transcript_10/10_Confidence_0.158_Length_1599, tri_n_comp15822_c0_seq1, vo_c1_Locus_27062_Transcript_5/7_Confidence_0.538_Length_1304, tri_n_comp15822_c0_seq2, vo_c3_Locus_9066_Transcript_1/6_Confidence_0.375_Length_846

Arachidonic acid metabolism	map00590	7	ec:3.1.1.4 - A2, ec:5.3.99.3 - synthase, ec:5.3.99.2 - synthase, ec:1.11.1.9 - peroxidase, ec:3.3.2.10 - epoxide hydrolase, ec:1.14.15.3 - 1-monooxygenase, ec:3.3.2.6 - hydrolase	59, 2, 1, 13, 3, 1, 3	vo_n2_Locus_311_Transcript_71/114_Confidence_0.150_Length_1045, vo_c3_Locus_7729_Transcript_4/5_Confidence_0.167_Length_5654, vo_n1_Locus_443_Transcript_30/53_Confidence_0.193_Length_928, vo_c1_Locus_430_Transcript_20/51_Confidence_0.291_Length_976, vo_c2_Locus_601_Transcript_31/36_Confidence_0.229_Length_1002, vo_c2_Locus_5092_Transcript_6/14_Confidence_0.476_Length_1090, vo_c3_Locus_1328_Transcript_24/26_Confidence_0.245_Length_1398, vo_n2_Locus_311_Transcript_78/114_Confidence_0.093_Length_844, vo_c3_Locus_1273_Transcript_51/57_Confidence_0.108_Length_895, vo_c1_Locus_21671_Transcript_6/49_Confidence_0.021_Length_1919, vo_c3_Locus_1273_Transcript_50/57_Confidence_0.144_Length_1471, vo_n1_Locus_443_Transcript_24/53_Confidence_0.109_Length_905, vo_n2_Locus_311_Transcript_87/114_Confidence_0.061_Length_2919, vo_n3_Locus_407_Transcript_22/43_Confidence_0.292_Length_1222, tri_n_comp10070_c0_seq2, tri_c_comp22021_c0_seq1, vo_n1_Locus_443_Transcript_32/53_Confidence_0.252_Length_1164, vo_n3_Locus_407_Transcript_32/43_Confidence_0.283_Length_1271, vo_n1_Locus_443_Transcript_10/53_Confidence_0.193_Length_1076, vo_n3_Locus_407_Transcript_7/43_Confidence_0.132_Length_1778, vo_n2_Locus_311_Transcript_109/114_Confidence_0.117_Length_1018, vo_n3_Locus_407_Transcript_39/43_Confidence_0.236_Length_1222, vo_c3_Locus_15467_Transcript_2/2_Confidence_0.000_Length_1100, vo_n1_Locus_4880_Transcript_2/6_Confidence_0.333_Length_883, vo_n2_Locus_3013_Transcript_1/2_Confidence_0.750_Length_964, vo_n2_Locus_311_Transcript_112/114_Confidence_0.146_Length_1678, vo_c2_Locus_601_Transcript_27/36_Confidence_0.337_Length_991, vo_c2_Locus_601_Transcript_2/36_Confidence_0.133_Length_1744, vo_n1_Locus_443_Transcript_39/53_Confidence_0.210_Length_1938, vo_c3_Locus_15467_Transcript_1/2_Confidence_0.750_Length_988, vo_c1_Locus_430_Transcript_11/51_Confidence_0.145_Length_955, vo_c2_Locus_5092_Transcript_10/14_Confidence_0.429_Length_1495, vo_n2_Locus_21867_Transcript_2/5_Confidence_0.200_Length_5139, tri_n_comp20432_c0_seq6, vo_n2_Locus_311_Transcript_84/114_Confidence_0.117_Length_1124, vo_n2_Locus_311_Transcript_77/114_Confidence_0.134_Length_997, vo_n2_Locus_311_Transcript_90/114_Confidence_0.061_Length_1016, vo_c2_Locus_601_Transcript_11/36_Confidence_0.289_Length_1016, tri_n_comp20432_c0_seq2, vo_c1_Locus_430_Transcript_48/51_Confidence_0.227_Length_968, vo_n1_Locus_443_Transcript_17/53_Confidence_0.168_Length_1009, tri_c_comp8925_c1_seq1, vo_c2_Locus_601_Transcript_18/36_Confidence_0.265_Length_801, vo_c3_Locus_1273_Transcript_17/57_Confidence_0.050_Length_1126, vo_n3_Locus_407_Transcript_40/43_Confidence_0.217_Length_1200, vo_c2_Locus_601_Transcript_36/36_Confidence_0.217_Length_953, vo_n3_Locus_407_Transcript_8/43_Confidence_0.189_Length_1800, vo_c3_Locus_1273_Transcript_16/57_Confidence_0.079_Length_1101, tri_n_comp20662_c0_seq7, vo_n2_Locus_23060_Transcript_2/2_Confidence_0.000_Length_889, vo_c2_Locus_601_Transcript_15/36_Confidence_0.337_Length_815, vo_n1_Locus_4880_Transcript_4/6_Confidence_0.333_Length_1109, vo_n2_Locus_311_Transcript_3/114_Confidence_0.142_Length_866, tri_c_comp20860_c0_seq2, vo_n1_Locus_443_Transcript_8/53_Confidence_0.261_Length_1031, vo_n1_Locus_443_Transcript_49/53_Confidence_0.134_Length_862, vo_n3_Locus_407_Transcript_14/43_Confidence_0.142_Length_872, vo_c2_Locus_5609_Transcript_4/4_Confidence_0.250_Length_1105, vo_c3_Locus_17900_Transcript_1/1_Confidence_0.000_Length_966, vo_c1_Locus_29164_Transcript_1/1_Confidence_0.000_Length_750, vo_c3_Locus_19248_Transcript_2/2_Confidence_0.000_Length_845, vo_c2_Locus_8227_Transcript_3/3_Confidence_0.143_Length_1106, vo_n2_Locus_1609_Transcript_3/3_Confidence_0.000_Length_489, vo_n3_Locus_21278_Transcript_2/2_Confidence_0.250_Length_899, vo_c3_Locus_7729_Transcript_4/5_Confidence_0.167_Length_5654, vo_c1_Locus_15275_Transcript_1/2_Confidence_0.333_Length_968, vo_n2_Locus_23060_Transcript_2/2_Confidence_0.000_Length_889, vo_n1_Locus_4880_Transcript_4/6_Confidence_0.333_Length_1109, vo_n1_Locus_4880_Transcript_2/6_Confidence_0.333_Length_883, vo_n2_Locus_3013_Transcript_1/2_Confidence_0.750_Length_964, vo_n2_Locus_34681_Transcript_5/6_Confidence_0.077_Length_2256, vo_c3_Locus_26080_Transcript_2/2_Confidence_0.250_Length_928, vo_c2_Locus_5609_Transcript_4/4_Confidence_0.250_Length_1105, vo_c3_Locus_17900_Transcript_1/1_Confidence_0.000_Length_966, tri_c_comp22021_c0_seq1, vo_n2_Locus_16627_Transcript_1/2_Confidence_0.333_Length_2167, vo_n2_Locus_13706_Transcript_2/3_Confidence_0.750_Length_2463, tri_n_comp17047_c0_seq1, vo_n2_Locus_2042_Transcript_5/7_Confidence_0.231_Length_2650, vo_n2_Locus_16627_Transcript_1/2_Confidence_0.333_Length_2167, vo_n2_Locus_13706_Transcript_2/3_Confidence_0.750_Length_2463, tri_n_comp17047_c0_seq1

Oxidative phosphorylation	map00190	6	ec:1.3.5.1 - dehydrogenase, ec:1.6.5.3 - reductase (H+-translocating), ec:3.6.1.1 - diphosphatase, ec:3.6.3.6 - ATPase, ec:1.9.3.1 - oxidase, ec:1.10.2.2 - reductase	11, 1, 14, 13, 46, 12	vo_n2_Locus_5500_Transcript_5/5_Confidence_0.308_Length_1268, vo_n2_Locus_5500_Transcript_1/5_Confidence_0.308_Length_1100, tri_n_comp12494_c0_seq1, vo_n2_Locus_5500_Transcript_2/5_Confidence_0.538_Length_1312, vo_n2_Locus_2182_Transcript_14/15_Confidence_0.458_Length_3129, vo_c2_Locus_642_Transcript_3/10_Confidence_0.571_Length_2141, vo_n1_Locus_1508_Transcript_4/6_Confidence_0.429_Length_1979, vo_n1_Locus_2259_Transcript_6/13_Confidence_0.429_Length_1275, vo_n1_Locus_2259_Transcript_9/13_Confidence_0.429_Length_1314, vo_c1_Locus_1462_Transcript_1/46_Confidence_0.182_Length_2129, vo_c1_Locus_1462_Transcript_8/46_Confidence_0.530_Length_9104, vo_n2_Locus_23058_Transcript_1/1_Confidence_0.000_Length_1342, vo_c2_Locus_1991_Transcript_1/9_Confidence_0.600_Length_1677, vo_c3_Locus_3320_Transcript_8/9_Confidence_0.450_Length_1918, vo_n2_Locus_3669_Transcript_6/9_Confidence_0.435_Length_642, vo_c3_Locus_3320_Transcript_6/9_Confidence_0.500_Length_1804, vo_n2_Locus_32920_Transcript_1/1_Confidence_0.000_Length_2385, vo_n1_Locus_12055_Transcript_5/12_Confidence_0.625_Length_1802, vo_c3_Locus_3661_Transcript_4/5_Confidence_0.444_Length_2325, vo_c1_Locus_5507_Transcript_1/1_Confidence_0.000_Length_1113, vo_c2_Locus_1991_Transcript_5/9_Confidence_0.600_Length_1783, vo_c2_Locus_6867_Transcript_3/4_Confidence_0.300_Length_472, vo_c2_Locus_2212_Transcript_3/4_Confidence_0.200_Length_1331, vo_n2_Locus_3669_Transcript_4/9_Confidence_0.565_Length_1692, vo_c3_Locus_7550_Transcript_2/2_Confidence_0.400_Length_1056, vo_n2_Locus_6371_Transcript_4/4_Confidence_0.000_Length_406, vo_c1_Locus_13640_Transcript_1/2_Confidence_0.972_Length_1746, vo_n2_Locus_3198_Transcript_13/13_Confidence_0.143_Length_3969, vo_c3_Locus_641_Transcript_2/3_Confidence_0.429_Length_2783, vo_n3_Locus_1906_Transcript_2/7_Confidence_0.389_Length_1663, vo_n2_Locus_7343_Transcript_2/5_Confidence_0.643_Length_1116, vo_n1_Locus_1052_Transcript_9/9_Confidence_0.150_Length_3323, vo_n2_Locus_3198_Transcript_7/13_Confidence_0.190_Length_1940, vo_c3_Locus_107_Transcript_2/2_Confidence_0.000_Length_1878, vo_n1_Locus_7271_Transcript_6/6_Confidence_0.500_Length_537, vo_n3_Locus_1906_Transcript_3/7_Confidence_0.389_Length_1640, vo_n2_Locus_3198_Transcript_4/13_Confidence_0.238_Length_2112, vo_c2_Locus_302_Transcript_1/1_Confidence_0.000_Length_1951, vo_n2_Locus_7343_Transcript_5/5_Confidence_0.286_Length_1183, vo_c1_Locus_350_Transcript_36/56_Confidence_0.031_Length_6073, vo_c3_Locus_350_Transcript_43/136_Confidence_0.013_Length_1090, vo_n3_Locus_2887_Transcript_17/89_Confidence_0.041_Length_2118, vo_n3_Locus_2887_Transcript_30/89_Confidence_0.038_Length_6215, vo_n2_Locus_2705_Transcript_77/102_Confidence_0.064_Length_11206, vo_c2_Locus_37_Transcript_240/305_Confidence_0.010_Length_5136, vo_c3_Locus_350_Transcript_53/136_Confidence_0.031_Length_2764, vo_n3_Locus_2887_Transcript_24/89_Confidence_0.014_Length_1665, tri_c_comp13429_c0_seq1, vo_c1_Locus_350_Transcript_28/56_Confidence_0.078_Length_10184, tri_c_comp20501_c0_seq96, vo_c3_Locus_350_Transcript_54/136_Confidence_0.013_Length_3833, vo_n2_Locus_2705_Transcript_52/102_Confidence_0.074_Length_11694, vo_c2_Locus_37_Transcript_265/305_Confidence_0.009_Length_4896, vo_c3_Locus_350_Transcript_72/136_Confidence_0.031_Length_2399, vo_c1_Locus_350_Transcript_41/56_Confidence_0.109_Length_7323, vo_c2_Locus_37_Transcript_244/305_Confidence_0.002_Length_3468, vo_n1_Locus_1328_Transcript_28/54_Confidence_0.026_Length_9460, vo_c2_Locus_37_Transcript_236/305_Confidence_0.014_Length_12025, vo_n3_Locus_2887_Transcript_89/89_Confidence_0.010_Length_4322, vo_c3_Locus_350_Transcript_52/136_Confidence_0.020_Length_3668, vo_n1_Locus_31781_Transcript_2/2_Confidence_0.000_Length_1482, vo_n1_Locus_6231_Transcript_1/1_Confidence_0.000_Length_808, vo_c2_Locus_37_Transcript_263/305_Confidence_0.010_Length_12853, vo_c1_Locus_350_Transcript_47/56_Confidence_0.146_Length_15341, vo_c2_Locus_7731_Transcript_1/1_Confidence_0.000_Length_894, tri_c_comp20501_c0_seq80, vo_n1_Locus_1328_Transcript_38/54_Confidence_0.020_Length_7735, vo_c1_Locus_350_Transcript_43/56_Confidence_0.109_Length_8257, tri_c_comp20501_c0_seq3, tri_n_comp20283_c1_seq60, vo_c1_Locus_20327_Transcript_1/1_Confidence_0.000_Length_657, tri_c_comp20501_c0_seq62, vo_n3_Locus_2887_Transcript_61/89_Confidence_0.097_Length_7484, tri_n_comp22502_c0_seq1, vo_n2_Locus_2705_Transcript_76/102_Confidence_0.092_Length_12161, tri_c_comp20501_c0_seq44, vo_n2_Locus_2705_Transcript_61/102_Confidence_0.131_Length_21257, vo_n3_Locus_2887_Transcript_22/89_Confidence_0.038_Length_3086, tri_c_comp20501_c0_seq26, vo_n1_Locus_1328_Transcript_44/54_Confidence_0.141_Length_7799, vo_c2_Locus_12863_Transcript_1/5_Confidence_0.500_Length_540, vo_c1_Locus_350_Transcript_34/56_Confidence_0.109_Length_14994, vo_c2_Locus_37_Transcript_268/305_Confidence_0.011_Length_8430, vo_n2_Locus_881_Transcript_1/1_Confidence_0.000_Length_815, vo_n2_Locus_6766_Transcript_4/5_Confidence_0.286_Length_587, vo_c2_Locus_5878_Transcript_5/7_Confidence_0.333_Length_1049, vo_c1_Locus_754_Transcript_8/11_Confidence_0.200_Length_971, vo_c1_Locus_754_Transcript_9/11_Confidence_0.200_Length_2339, vo_c1_Locus_8997_Transcript_2/2_Confidence_0.000_Length_877, vo_c3_Locus_6650_Transcript_4/8_Confidence_0.308_Length_1102, vo_c1_Locus_754_Transcript_5/11_Confidence_0.300_Length_1714, vo_c2_Locus_5878_Transcript_3/7_Confidence_0.444_Length_1038, vo_n1_Locus_1379_Transcript_7/9_Confidence_0.154_Length_925, vo_c2_Locus_455_Transcript_3/3_Confidence_0.000_Length_684, vo_n3_Locus_3061_Transcript_4/7_Confidence_0.417_Length_922, vo_n2_Locus_3484_Transcript_2/9_Confidence_0.333_Length_887, vo_c1_Locus_754_Transcript_7/11_Confidence_0.250_Length_1706

Streptomycin biosynthesis	map00521	6	ec:1.1.1.133 - reductase, ec:5.1.3.13 - 3,5-epimerase, ec:2.7.1.2 - glucokinase (phosphorylating), ec:3.1.3.25 - phosphatase, ec:4.2.1.46 - 4,6-dehydratase, ec:5.4.2.2 - (alpha-D-glucose-1,6-bisphosphate-dependent)	2, 1, 2, 5, 2, 7	vo_c2_Locus_1643_Transcript_1/1_Confidence_0.000_Length_1756, vo_c1_Locus_3806_Transcript_1/1_Confidence_0.000_Length_1482, vo_c2_Locus_1643_Transcript_1/1_Confidence_0.000_Length_1756, tri_c_comp10536_c0_seq1, vo_c2_Locus_32726_Transcript_2/2_Confidence_0.000_Length_696, vo_n2_Locus_24510_Transcript_1/1_Confidence_0.000_Length_1024, vo_c2_Locus_24561_Transcript_2/2_Confidence_0.000_Length_1596, vo_c2_Locus_24561_Transcript_1/2_Confidence_0.750_Length_1539, vo_c1_Locus_21354_Transcript_1/6_Confidence_0.600_Length_1404, vo_c2_Locus_13210_Transcript_4/4_Confidence_0.125_Length_1454, vo_c2_Locus_5377_Transcript_5/9_Confidence_0.150_Length_2142, vo_n1_Locus_5366_Transcript_4/4_Confidence_0.556_Length_1623, tri_n_comp18813_c0_seq13, vo_c2_Locus_23037_Transcript_2/2_Confidence_0.000_Length_2109, vo_n3_Locus_362_Transcript_3/4_Confidence_0.364_Length_3895, vo_n3_Locus_362_Transcript_1/4_Confidence_0.727_Length_3306, vo_n1_Locus_5208_Transcript_2/2_Confidence_0.000_Length_2077, vo_c2_Locus_23037_Transcript_1/2_Confidence_0.750_Length_1965, vo_n3_Locus_15336_Transcript_1/1_Confidence_0.000_Length_1810

Ether lipid metabolism	map00565	6	ec:2.7.8.2 - cholinephosphotransferase, ec:2.5.1.26 - synthase, ec:3.1.1.4 - A2, ec:3.1.1.47 - esterase, ec:2.8.2.11 - sulfotransferase, ec:3.1.4.4 - D	1, 2, 59, 4, 31, 13	vo_n2_Locus_32954_Transcript_1/2_Confidence_0.333_Length_1192, vo_c2_Locus_6733_Transcript_3/6_Confidence_0.364_Length_7025, tri_n_comp39945_c0_seq1, vo_n2_Locus_311_Transcript_71/114_Confidence_0.150_Length_1045, vo_c3_Locus_7729_Transcript_4/5_Confidence_0.167_Length_5654, vo_n1_Locus_443_Transcript_30/53_Confidence_0.193_Length_928, vo_c1_Locus_430_Transcript_20/51_Confidence_0.291_Length_976, vo_c2_Locus_601_Transcript_31/36_Confidence_0.229_Length_1002, vo_c2_Locus_5092_Transcript_6/14_Confidence_0.476_Length_1090, vo_c3_Locus_1328_Transcript_24/26_Confidence_0.245_Length_1398, vo_n2_Locus_311_Transcript_78/114_Confidence_0.093_Length_844, vo_c3_Locus_1273_Transcript_51/57_Confidence_0.108_Length_895, vo_c1_Locus_21671_Transcript_6/49_Confidence_0.021_Length_1919, vo_c3_Locus_1273_Transcript_50/57_Confidence_0.144_Length_1471, vo_n1_Locus_443_Transcript_24/53_Confidence_0.109_Length_905, vo_n2_Locus_311_Transcript_87/114_Confidence_0.061_Length_2919, vo_n3_Locus_407_Transcript_22/43_Confidence_0.292_Length_1222, tri_n_comp10070_c0_seq2, tri_c_comp22021_c0_seq1, vo_n1_Locus_443_Transcript_32/53_Confidence_0.252_Length_1164, vo_n3_Locus_407_Transcript_32/43_Confidence_0.283_Length_1271, vo_n1_Locus_443_Transcript_10/53_Confidence_0.193_Length_1076, vo_n3_Locus_407_Transcript_7/43_Confidence_0.132_Length_1778, vo_n2_Locus_311_Transcript_109/114_Confidence_0.117_Length_1018, vo_n3_Locus_407_Transcript_39/43_Confidence_0.236_Length_1222, vo_c3_Locus_15467_Transcript_2/2_Confidence_0.000_Length_1100, vo_n1_Locus_4880_Transcript_2/6_Confidence_0.333_Length_883, vo_n2_Locus_3013_Transcript_1/2_Confidence_0.750_Length_964, vo_n2_Locus_311_Transcript_112/114_Confidence_0.146_Length_1678, vo_c2_Locus_601_Transcript_27/36_Confidence_0.337_Length_991, vo_c2_Locus_601_Transcript_2/36_Confidence_0.133_Length_1744, vo_n1_Locus_443_Transcript_39/53_Confidence_0.210_Length_1938, vo_c3_Locus_15467_Transcript_1/2_Confidence_0.750_Length_988, vo_c1_Locus_430_Transcript_11/51_Confidence_0.145_Length_955, vo_c2_Locus_5092_Transcript_10/14_Confidence_0.429_Length_1495, vo_n2_Locus_21867_Transcript_2/5_Confidence_0.200_Length_5139, tri_n_comp20432_c0_seq6, vo_n2_Locus_311_Transcript_84/114_Confidence_0.117_Length_1124, vo_n2_Locus_311_Transcript_77/114_Confidence_0.134_Length_997, vo_n2_Locus_311_Transcript_90/114_Confidence_0.061_Length_1016, vo_c2_Locus_601_Transcript_11/36_Confidence_0.289_Length_1016, tri_n_comp20432_c0_seq2, vo_c1_Locus_430_Transcript_48/51_Confidence_0.227_Length_968, vo_n1_Locus_443_Transcript_17/53_Confidence_0.168_Length_1009, tri_c_comp8925_c1_seq1, vo_c2_Locus_601_Transcript_18/36_Confidence_0.265_Length_801, vo_c3_Locus_1273_Transcript_17/57_Confidence_0.050_Length_1126, vo_n3_Locus_407_Transcript_40/43_Confidence_0.217_Length_1200, vo_c2_Locus_601_Transcript_36/36_Confidence_0.217_Length_953, vo_n3_Locus_407_Transcript_8/43_Confidence_0.189_Length_1800, vo_c3_Locus_1273_Transcript_16/57_Confidence_0.079_Length_1101, tri_n_comp20662_c0_seq7, vo_n2_Locus_23060_Transcript_2/2_Confidence_0.000_Length_889, vo_c2_Locus_601_Transcript_15/36_Confidence_0.337_Length_815, vo_n1_Locus_4880_Transcript_4/6_Confidence_0.333_Length_1109, vo_n2_Locus_311_Transcript_3/114_Confidence_0.142_Length_866, tri_c_comp20860_c0_seq2, vo_n1_Locus_443_Transcript_8/53_Confidence_0.261_Length_1031, vo_n1_Locus_443_Transcript_49/53_Confidence_0.134_Length_862, vo_n3_Locus_407_Transcript_14/43_Confidence_0.142_Length_872, vo_c2_Locus_5609_Transcript_4/4_Confidence_0.250_Length_1105, vo_c3_Locus_17900_Transcript_1/1_Confidence_0.000_Length_966, tri_c_comp3336_c0_seq2, tri_n_comp26298_c0_seq1, tri_c_comp9493_c0_seq1, tri_c_comp3336_c0_seq1, vo_c2_Locus_11068_Transcript_7/15_Confidence_0.088_Length_2478, tri_c_comp19434_c0_seq20, vo_n3_Locus_427_Transcript_152/175_Confidence_0.022_Length_1587, vo_n2_Locus_39296_Transcript_1/14_Confidence_0.615_Length_1447, vo_n1_Locus_1282_Transcript_21/28_Confidence_0.267_Length_1443, vo_c3_Locus_12992_Transcript_17/18_Confidence_0.438_Length_1753, vo_c3_Locus_10470_Transcript_2/7_Confidence_0.500_Length_1430, tri_c_comp18408_c0_seq3, vo_c3_Locus_15517_Transcript_3/3_Confidence_0.500_Length_1506, vo_c1_Locus_2146_Transcript_6/28_Confidence_0.438_Length_1427, tri_c_comp10922_c0_seq1, vo_n1_Locus_1282_Transcript_25/28_Confidence_0.250_Length_1445, vo_n1_Locus_1282_Transcript_18/28_Confidence_0.350_Length_1518, vo_c3_Locus_12992_Transcript_1/18_Confidence_0.562_Length_1707, vo_n1_Locus_1282_Transcript_20/28_Confidence_0.250_Length_1445, vo_n3_Locus_23180_Transcript_1/1_Confidence_0.000_Length_1372, vo_c3_Locus_12992_Transcript_2/18_Confidence_0.438_Length_1659, vo_n2_Locus_3_Transcript_18204/24291_Confidence_0.000_Length_1558, vo_c3_Locus_12992_Transcript_13/18_Confidence_0.562_Length_1789, vo_c3_Locus_15917_Transcript_1/2_Confidence_0.667_Length_1528, vo_c3_Locus_6108_Transcript_1/20_Confidence_0.667_Length_1501, vo_n2_Locus_1168_Transcript_12/19_Confidence_0.567_Length_1720, vo_c1_Locus_29045_Transcript_1/2_Confidence_0.600_Length_1495, vo_c3_Locus_12992_Transcript_14/18_Confidence_0.719_Length_1813, vo_c2_Locus_2351_Transcript_10/22_Confidence_0.081_Length_950, vo_c3_Locus_16912_Transcript_3/4_Confidence_0.500_Length_1461, vo_c2_Locus_557_Transcript_42/127_Confidence_0.063_Length_2107, vo_n1_Locus_1282_Transcript_28/28_Confidence_0.133_Length_1479, vo_c3_Locus_21516_Transcript_2/3_Confidence_0.333_Length_2294, vo_n2_Locus_4388_Transcript_1/1_Confidence_0.000_Length_1497, tri_n_comp20471_c0_seq2, vo_c1_Locus_947_Transcript_1/7_Confidence_0.286_Length_2009, vo_c3_Locus_6042_Transcript_5/5_Confidence_0.300_Length_3385, vo_c3_Locus_4758_Transcript_1/1_Confidence_0.000_Length_1881, tri_n_comp25890_c0_seq1, tri_c_comp20356_c0_seq35, tri_c_comp20356_c0_seq10, tri_c_comp20356_c0_seq21, vo_n2_Locus_6470_Transcript_5/10_Confidence_0.333_Length_2772, tri_c_comp20356_c0_seq29, tri_c_comp20356_c0_seq27, tri_c_comp20356_c0_seq25, tri_c_comp20356_c0_seq36, tri_c_comp20356_c0_seq47

Monobactam biosynthesis	map00261	5	ec:2.7.2.4 - kinase, ec:1.2.1.11 - dehydrogenase, ec:1.17.1.8 - reductase, ec:4.3.3.7 - synthase, ec:2.7.7.4 - adenylyltransferase	7, 4, 3, 1, 7	vo_c1_Locus_1636_Transcript_120/122_Confidence_0.135_Length_3625, tri_n_comp29741_c0_seq1, vo_c3_Locus_31_Transcript_74/79_Confidence_0.124_Length_2769, vo_c2_Locus_10862_Transcript_1/1_Confidence_0.000_Length_1564, vo_c3_Locus_31_Transcript_63/79_Confidence_0.148_Length_3613, vo_c1_Locus_1636_Transcript_116/122_Confidence_0.129_Length_3526, vo_c2_Locus_52_Transcript_164/466_Confidence_0.011_Length_2810, vo_n2_Locus_3068_Transcript_2/3_Confidence_0.250_Length_1991, vo_n1_Locus_4025_Transcript_3/5_Confidence_0.286_Length_3475, vo_c1_Locus_9004_Transcript_2/4_Confidence_0.400_Length_3537, vo_c1_Locus_10440_Transcript_1/2_Confidence_0.667_Length_1569, vo_n1_Locus_32462_Transcript_1/1_Confidence_0.000_Length_1047, vo_n2_Locus_24260_Transcript_2/3_Confidence_0.250_Length_976, vo_c1_Locus_22485_Transcript_1/2_Confidence_0.500_Length_1035, vo_c3_Locus_7185_Transcript_2/3_Confidence_0.333_Length_1661, vo_n2_Locus_1711_Transcript_9/11_Confidence_0.320_Length_3674, vo_n2_Locus_1711_Transcript_8/11_Confidence_0.360_Length_2026, vo_c2_Locus_2008_Transcript_2/7_Confidence_0.231_Length_3495, vo_c1_Locus_18424_Transcript_1/2_Confidence_0.750_Length_906, tri_n_comp20038_c0_seq1, vo_n2_Locus_1711_Transcript_2/11_Confidence_0.280_Length_4019, vo_c1_Locus_18424_Transcript_2/2_Confidence_0.000_Length_956

Phosphonate and phosphinate metabolism	map00440	5	ec:2.6.1.37 - transaminase, ec:2.7.8.2 - cholinephosphotransferase, ec:3.11.1.2 - hydrolase, ec:2.7.7.14 - cytidylyltransferase, ec:2.7.7.15 - cytidylyltransferase	1, 1, 1, 3, 4	vo_c2_Locus_11195_Transcript_1/1_Confidence_0.000_Length_1464, vo_n2_Locus_32954_Transcript_1/2_Confidence_0.333_Length_1192, vo_c2_Locus_8193_Transcript_6/7_Confidence_0.091_Length_1909, vo_c3_Locus_19662_Transcript_1/2_Confidence_0.833_Length_5009, vo_c3_Locus_1617_Transcript_6/6_Confidence_0.222_Length_1645, vo_n1_Locus_16053_Transcript_1/1_Confidence_0.000_Length_1438, vo_c3_Locus_19662_Transcript_1/2_Confidence_0.833_Length_5009, vo_c3_Locus_1617_Transcript_6/6_Confidence_0.222_Length_1645, vo_n1_Locus_16053_Transcript_1/1_Confidence_0.000_Length_1438, vo_n1_Locus_29872_Transcript_1/1_Confidence_0.000_Length_1764

Drug metabolism - other enzymes	map00983	5	ec:2.7.1.48 - kinase, ec:6.3.5.2 - synthase (glutamine-hydrolysing), ec:2.4.1.17 - 1-naphthol glucuronyltransferase, ec:1.1.1.205 - dehydrogenase, ec:2.4.2.10 - phosphoribosyltransferase	10, 11, 13, 12, 1	vo_n1_Locus_27053_Transcript_1/1_Confidence_0.333_Length_1866, vo_n2_Locus_1685_Transcript_1/2_Confidence_0.667_Length_854, vo_n1_Locus_1829_Transcript_14/31_Confidence_0.635_Length_1771, vo_n1_Locus_1829_Transcript_24/31_Confidence_0.558_Length_1469, vo_c3_Locus_527_Transcript_60/87_Confidence_0.092_Length_2707, vo_n1_Locus_1829_Transcript_21/31_Confidence_0.519_Length_1458, vo_c1_Locus_12423_Transcript_2/2_Confidence_0.333_Length_980, vo_n2_Locus_26604_Transcript_1/2_Confidence_0.750_Length_1361, vo_n1_Locus_1829_Transcript_31/31_Confidence_0.269_Length_1123, vo_n2_Locus_26604_Transcript_2/2_Confidence_0.000_Length_1685, vo_n1_Locus_9681_Transcript_4/11_Confidence_0.529_Length_1748, vo_n2_Locus_215_Transcript_14/28_Confidence_0.182_Length_1973, vo_n3_Locus_2366_Transcript_13/24_Confidence_0.236_Length_1948, vo_n1_Locus_9681_Transcript_6/11_Confidence_0.353_Length_1721, vo_n2_Locus_215_Transcript_12/28_Confidence_0.212_Length_2644, vo_n1_Locus_9681_Transcript_11/11_Confidence_0.000_Length_529, vo_n1_Locus_9681_Transcript_5/11_Confidence_0.529_Length_1790, vo_n2_Locus_215_Transcript_22/28_Confidence_0.197_Length_2480, vo_n2_Locus_215_Transcript_8/28_Confidence_0.227_Length_2670, vo_c1_Locus_1789_Transcript_29/32_Confidence_0.300_Length_3220, vo_n1_Locus_6239_Transcript_3/19_Confidence_0.425_Length_1997, tri_c_comp6209_c0_seq1, vo_c1_Locus_26340_Transcript_5/9_Confidence_0.600_Length_1699, tri_n_comp65990_c0_seq1, vo_n1_Locus_13372_Transcript_1/2_Confidence_0.333_Length_1687, vo_n2_Locus_34154_Transcript_3/3_Confidence_0.000_Length_2043, vo_n2_Locus_668_Transcript_17/17_Confidence_0.070_Length_2403, tri_c_comp58135_c0_seq1, vo_c3_Locus_16093_Transcript_1/1_Confidence_0.000_Length_1750, vo_n1_Locus_13372_Transcript_2/2_Confidence_0.000_Length_1314, vo_c2_Locus_24841_Transcript_3/3_Confidence_0.167_Length_3241, vo_n2_Locus_4774_Transcript_1/1_Confidence_0.000_Length_884, vo_c1_Locus_35615_Transcript_1/1_Confidence_0.000_Length_2075, vo_n1_Locus_15639_Transcript_1/1_Confidence_0.000_Length_1086, vo_n2_Locus_21654_Transcript_1/2_Confidence_0.333_Length_1437, tri_n_comp20442_c0_seq2, vo_c1_Locus_4865_Transcript_8/17_Confidence_0.303_Length_1948, vo_n1_Locus_1854_Transcript_11/16_Confidence_0.294_Length_1888, tri_c_comp31112_c0_seq1, tri_n_comp29350_c0_seq1, vo_n1_Locus_1854_Transcript_12/16_Confidence_0.324_Length_2352, vo_n1_Locus_4507_Transcript_1/1_Confidence_0.000_Length_2067, vo_c2_Locus_673_Transcript_7/13_Confidence_0.438_Length_2095, vo_n3_Locus_24688_Transcript_1/2_Confidence_1.000_Length_1616, vo_c2_Locus_673_Transcript_6/13_Confidence_0.375_Length_2722, vo_c2_Locus_940_Transcript_2/3_Confidence_0.136_Length_2144, vo_c1_Locus_6671_Transcript_2/2_Confidence_0.000_Length_1815

Biotin metabolism	map00780	5	ec:1.1.1.100 - reductase, ec:4.2.1.59 - dehydratase, ec:6.3.4.15 - ligase, ec:2.3.1.41 - synthase I, ec:2.3.1.47 - synthase	4, 1, 2, 3, 1	vo_n3_Locus_484_Transcript_6/12_Confidence_0.250_Length_1533, vo_c2_Locus_12502_Transcript_2/2_Confidence_0.000_Length_781, vo_n3_Locus_484_Transcript_5/12_Confidence_0.250_Length_1451, vo_c2_Locus_2193_Transcript_1/3_Confidence_0.667_Length_1218, vo_c1_Locus_4631_Transcript_1/1_Confidence_0.000_Length_1088, vo_c3_Locus_9194_Transcript_2/3_Confidence_0.667_Length_1368, vo_c1_Locus_1719_Transcript_3/3_Confidence_0.000_Length_1383, vo_c3_Locus_422_Transcript_1/3_Confidence_0.857_Length_2914, vo_n1_Locus_8253_Transcript_1/1_Confidence_0.000_Length_1430, vo_n2_Locus_4712_Transcript_1/2_Confidence_0.333_Length_1867, tri_n_comp5199_c0_seq1

Fatty acid elongation	map00062	5	ec:3.1.2.22 - hydrolase, ec:4.2.1.74 - hydratase, ec:3.1.2.2 - hydrolase, ec:4.2.1.17 - hydratase, ec:1.1.1.35 - dehydrogenase	4, 11, 2, 7, 16	vo_n2_Locus_3_Transcript_9963/24291_Confidence_0.000_Length_1392, vo_c1_Locus_18266_Transcript_15/25_Confidence_0.047_Length_1151, vo_c3_Locus_13218_Transcript_2/2_Confidence_0.333_Length_1246, vo_c2_Locus_22217_Transcript_1/2_Confidence_0.667_Length_1279, vo_c2_Locus_2261_Transcript_1/2_Confidence_1.000_Length_2564, vo_n1_Locus_9414_Transcript_8/13_Confidence_0.167_Length_606, vo_c3_Locus_9925_Transcript_8/11_Confidence_0.263_Length_3550, vo_c2_Locus_189_Transcript_29/60_Confidence_0.015_Length_692, vo_c3_Locus_2031_Transcript_8/8_Confidence_0.111_Length_1389, vo_n2_Locus_636_Transcript_16/16_Confidence_0.139_Length_616, vo_n2_Locus_636_Transcript_14/16_Confidence_0.222_Length_622, vo_n2_Locus_636_Transcript_5/16_Confidence_0.194_Length_591, vo_n2_Locus_636_Transcript_3/16_Confidence_0.306_Length_685, vo_n2_Locus_30747_Transcript_1/1_Confidence_0.000_Length_951, vo_n2_Locus_636_Transcript_13/16_Confidence_0.222_Length_768, vo_n2_Locus_14051_Transcript_7/8_Confidence_0.077_Length_1546, tri_c_comp14810_c0_seq3, vo_c2_Locus_2261_Transcript_1/2_Confidence_1.000_Length_2564, vo_c3_Locus_5948_Transcript_1/1_Confidence_0.000_Length_1027, vo_n2_Locus_16945_Transcript_1/3_Confidence_0.500_Length_1084, vo_c3_Locus_9925_Transcript_8/11_Confidence_0.263_Length_3550, vo_n1_Locus_11137_Transcript_5/5_Confidence_0.143_Length_2829, vo_n2_Locus_16945_Transcript_3/3_Confidence_0.000_Length_746, vo_n2_Locus_30747_Transcript_1/1_Confidence_0.000_Length_951, vo_c2_Locus_2261_Transcript_1/2_Confidence_1.000_Length_2564, vo_n1_Locus_9414_Transcript_8/13_Confidence_0.167_Length_606, vo_c1_Locus_40339_Transcript_1/1_Confidence_0.000_Length_1318, vo_c3_Locus_2031_Transcript_8/8_Confidence_0.111_Length_1389, vo_n1_Locus_11137_Transcript_5/5_Confidence_0.143_Length_2829, vo_c1_Locus_4675_Transcript_3/3_Confidence_0.200_Length_1348, vo_n2_Locus_30747_Transcript_1/1_Confidence_0.000_Length_951, vo_n2_Locus_636_Transcript_13/16_Confidence_0.222_Length_768, vo_c1_Locus_2815_Transcript_3/4_Confidence_0.200_Length_2244, vo_c3_Locus_9925_Transcript_8/11_Confidence_0.263_Length_3550, tri_n_comp42565_c0_seq1, vo_c2_Locus_189_Transcript_29/60_Confidence_0.015_Length_692, vo_n2_Locus_636_Transcript_16/16_Confidence_0.139_Length_616, vo_n2_Locus_636_Transcript_14/16_Confidence_0.222_Length_622, vo_n2_Locus_636_Transcript_5/16_Confidence_0.194_Length_591, vo_n2_Locus_636_Transcript_3/16_Confidence_0.306_Length_685

Biosynthesis of unsaturated fatty acids	map01040	5	ec:1.1.1.100 - reductase, ec:1.3.3.6 - oxidase, ec:3.1.2.2 - hydrolase, ec:4.2.1.17 - hydratase, ec:1.14.19.1 - 9-desaturase	4, 2, 2, 7, 3	vo_n3_Locus_484_Transcript_6/12_Confidence_0.250_Length_1533, vo_c2_Locus_12502_Transcript_2/2_Confidence_0.000_Length_781, vo_n3_Locus_484_Transcript_5/12_Confidence_0.250_Length_1451, vo_c2_Locus_2193_Transcript_1/3_Confidence_0.667_Length_1218, tri_n_comp36670_c0_seq1, vo_n1_Locus_23625_Transcript_1/1_Confidence_0.000_Length_2286, vo_n2_Locus_14051_Transcript_7/8_Confidence_0.077_Length_1546, tri_c_comp14810_c0_seq3, vo_c2_Locus_2261_Transcript_1/2_Confidence_1.000_Length_2564, vo_c3_Locus_5948_Transcript_1/1_Confidence_0.000_Length_1027, vo_n2_Locus_16945_Transcript_1/3_Confidence_0.500_Length_1084, vo_c3_Locus_9925_Transcript_8/11_Confidence_0.263_Length_3550, vo_n1_Locus_11137_Transcript_5/5_Confidence_0.143_Length_2829, vo_n2_Locus_16945_Transcript_3/3_Confidence_0.000_Length_746, vo_n2_Locus_30747_Transcript_1/1_Confidence_0.000_Length_951, vo_c1_Locus_20327_Transcript_1/1_Confidence_0.000_Length_657, vo_c1_Locus_3644_Transcript_23/23_Confidence_0.148_Length_1685, vo_c1_Locus_3644_Transcript_11/23_Confidence_0.556_Length_1703

Histidine metabolism	map00340	5	ec:1.2.1.5 - dehydrogenase [NAD(P)+], ec:1.2.1.3 - dehydrogenase (NAD+), ec:3.1.3.15 - histidinol phosphate phosphatase, ec:2.4.2.17 - phosphoribosyltransferase, ec:5.3.1.16 - isomerase	2, 5, 1, 2, 1	tri_c_comp55340_c0_seq1, tri_n_comp58061_c0_seq1, tri_c_comp55340_c0_seq1, vo_n1_Locus_19864_Transcript_2/2_Confidence_0.000_Length_2707, vo_c3_Locus_21741_Transcript_5/9_Confidence_0.583_Length_1757, vo_n3_Locus_25600_Transcript_5/6_Confidence_0.375_Length_1777, tri_n_comp58061_c0_seq1, vo_n2_Locus_24510_Transcript_1/1_Confidence_0.000_Length_1024, vo_n2_Locus_19769_Transcript_1/2_Confidence_0.333_Length_1281, tri_c_comp33903_c0_seq1, vo_n1_Locus_33739_Transcript_1/1_Confidence_0.000_Length_1392

Phenylpropanoid biosynthesis	map00940	5	ec:1.2.1.44 - reductase, ec:3.2.1.126 - beta-glucosidase, ec:3.2.1.21 - gentiobiase, ec:1.11.1.7 - lactoperoxidase, ec:2.1.1.104 - O-methyltransferase	1, 2, 1, 25, 2	tri_n_comp26842_c0_seq1, tri_n_comp70995_c0_seq1, vo_c3_Locus_32079_Transcript_2/2_Confidence_0.250_Length_2113, vo_c2_Locus_13105_Transcript_1/1_Confidence_0.000_Length_2797, vo_n2_Locus_3_Transcript_23668/24291_Confidence_0.000_Length_1121, vo_c1_Locus_1107_Transcript_1/4_Confidence_0.143_Length_1311, vo_c3_Locus_15467_Transcript_1/2_Confidence_0.750_Length_988, vo_c2_Locus_437_Transcript_6/7_Confidence_0.286_Length_3893, vo_c3_Locus_7802_Transcript_5/8_Confidence_0.440_Length_1882, vo_n3_Locus_6056_Transcript_2/8_Confidence_0.200_Length_1860, vo_n2_Locus_2619_Transcript_8/9_Confidence_0.357_Length_1435, vo_n2_Locus_852_Transcript_19/20_Confidence_0.419_Length_1873, vo_n3_Locus_2602_Transcript_1/2_Confidence_0.750_Length_664, tri_c_comp52750_c0_seq1, vo_n2_Locus_3_Transcript_19366/24291_Confidence_0.000_Length_2154, vo_c1_Locus_12166_Transcript_1/1_Confidence_0.000_Length_2425, vo_n3_Locus_15275_Transcript_1/5_Confidence_0.300_Length_3763, vo_n1_Locus_2648_Transcript_4/13_Confidence_0.536_Length_2114, vo_n2_Locus_2619_Transcript_6/9_Confidence_0.286_Length_1425, vo_c3_Locus_1212_Transcript_2/3_Confidence_0.333_Length_3255, vo_n1_Locus_11008_Transcript_5/16_Confidence_0.571_Length_3078, vo_n1_Locus_21070_Transcript_5/8_Confidence_0.500_Length_2178, vo_c3_Locus_15467_Transcript_2/2_Confidence_0.000_Length_1100, vo_n1_Locus_14713_Transcript_8/13_Confidence_0.529_Length_2511, vo_c3_Locus_2692_Transcript_1/3_Confidence_0.600_Length_1240, vo_n1_Locus_9334_Transcript_2/2_Confidence_0.333_Length_768, vo_c1_Locus_1967_Transcript_9/10_Confidence_0.263_Length_3482, tri_c_comp10308_c0_seq2, vo_c3_Locus_11858_Transcript_3/3_Confidence_0.000_Length_503, vo_n2_Locus_30574_Transcript_4/4_Confidence_0.000_Length_969, vo_n2_Locus_30574_Transcript_3/4_Confidence_0.500_Length_827

Various types of N-glycan biosynthesis	map00513	5	ec:2.4.1.260 - alpha-1,6-mannosyltransferase, ec:3.2.1.52 - hexosaminidase, ec:3.2.1.113 - 1,2-alpha-mannosidase, ec:2.4.1.38 - beta-1,4-galactosyltransferase, ec:2.4.1.101 - 2-beta-N-acetylglucosaminyltransferase	1, 13, 8, 2, 3	tri_n_comp14001_c0_seq2, vo_c3_Locus_25279_Transcript_1/3_Confidence_0.500_Length_3460, tri_c_comp15706_c0_seq2, vo_n2_Locus_4554_Transcript_3/4_Confidence_0.444_Length_2253, vo_n2_Locus_3_Transcript_18735/24291_Confidence_0.000_Length_2121, vo_n2_Locus_4395_Transcript_3/3_Confidence_0.167_Length_2032, vo_c2_Locus_6406_Transcript_1/4_Confidence_0.333_Length_1666, vo_n1_Locus_14758_Transcript_1/2_Confidence_0.667_Length_1933, tri_n_comp35667_c0_seq1, vo_n2_Locus_4395_Transcript_1/3_Confidence_0.667_Length_1925, tri_c_comp32936_c0_seq1, tri_n_comp92955_c0_seq1, tri_n_comp30287_c0_seq1, vo_n2_Locus_4554_Transcript_4/4_Confidence_0.111_Length_2049, vo_n2_Locus_3852_Transcript_1/7_Confidence_0.417_Length_4358, tri_n_comp47354_c0_seq1, vo_n1_Locus_9004_Transcript_4/20_Confidence_0.171_Length_2905, vo_c1_Locus_37790_Transcript_1/1_Confidence_0.000_Length_3188, vo_c3_Locus_18796_Transcript_3/3_Confidence_0.000_Length_3826, vo_n1_Locus_24857_Transcript_1/1_Confidence_0.000_Length_2424, vo_c1_Locus_1525_Transcript_2/7_Confidence_0.167_Length_2762, vo_n2_Locus_5268_Transcript_1/2_Confidence_0.917_Length_1282, tri_c_comp20165_c0_seq7, tri_c_comp20165_c0_seq3, vo_c2_Locus_1239_Transcript_4/4_Confidence_0.000_Length_1343, tri_c_comp9226_c0_seq1, vo_n2_Locus_19192_Transcript_2/2_Confidence_0.250_Length_3053

Thiamine metabolism	map00730	5	ec:2.7.4.3 - kinase, ec:2.2.1.7 - synthase, ec:2.8.1.7 - desulfurase, ec:3.6.1.15 - phosphatase, ec:3.6.1.28 - ec:3.6.1.28 thiamine-triphosphatase	10, 1, 7, 54, 10	vo_c2_Locus_10361_Transcript_6/7_Confidence_0.250_Length_1220, tri_c_comp24673_c0_seq1, vo_n1_Locus_2451_Transcript_1/3_Confidence_0.600_Length_2348, vo_c3_Locus_4102_Transcript_1/1_Confidence_0.000_Length_1034, vo_n2_Locus_22654_Transcript_1/1_Confidence_0.000_Length_691, vo_n1_Locus_2897_Transcript_2/14_Confidence_0.160_Length_971, vo_n2_Locus_14542_Transcript_1/3_Confidence_0.200_Length_924, vo_n3_Locus_12191_Transcript_1/1_Confidence_0.000_Length_1671, vo_n2_Locus_5279_Transcript_1/1_Confidence_0.333_Length_2493, vo_n3_Locus_24634_Transcript_1/1_Confidence_0.000_Length_445, vo_c1_Locus_12331_Transcript_1/1_Confidence_0.000_Length_2548, vo_n2_Locus_8711_Transcript_5/5_Confidence_0.067_Length_1558, vo_c2_Locus_11984_Transcript_2/6_Confidence_0.385_Length_1557, vo_c3_Locus_9097_Transcript_2/4_Confidence_0.600_Length_3293, tri_n_comp37185_c0_seq1, vo_c1_Locus_38066_Transcript_1/1_Confidence_0.000_Length_1674, vo_c2_Locus_14495_Transcript_1/4_Confidence_0.714_Length_1830, tri_c_comp30049_c0_seq1, vo_c1_Locus_103_Transcript_20/42_Confidence_0.312_Length_4488, vo_n2_Locus_12749_Transcript_4/14_Confidence_0.387_Length_2426, vo_c3_Locus_9603_Transcript_2/4_Confidence_0.286_Length_3163, vo_c3_Locus_779_Transcript_1/37_Confidence_0.227_Length_6196, tri_c_comp21210_c0_seq2, vo_n3_Locus_479_Transcript_48/55_Confidence_0.124_Length_2107, vo_c3_Locus_2891_Transcript_1/3_Confidence_0.667_Length_13029, vo_c3_Locus_5178_Transcript_4/7_Confidence_0.636_Length_3142, vo_n2_Locus_5227_Transcript_2/3_Confidence_0.200_Length_602, vo_n3_Locus_9266_Transcript_10/10_Confidence_0.000_Length_869, vo_c1_Locus_103_Transcript_18/42_Confidence_0.385_Length_4716, tri_c_comp22413_c0_seq1, vo_c2_Locus_579_Transcript_49/548_Confidence_0.023_Length_681, vo_c1_Locus_3866_Transcript_5/15_Confidence_0.087_Length_4747, vo_c3_Locus_2001_Transcript_1/1_Confidence_0.000_Length_1692, vo_n2_Locus_13957_Transcript_1/1_Confidence_0.000_Length_2805, vo_n2_Locus_4636_Transcript_1/1_Confidence_0.000_Length_673, vo_n3_Locus_19594_Transcript_3/22_Confidence_0.055_Length_2072, vo_c3_Locus_5178_Transcript_7/7_Confidence_0.000_Length_4922, tri_c_comp20372_c0_seq3, vo_c3_Locus_17023_Transcript_2/5_Confidence_0.636_Length_3545, vo_n2_Locus_3_Transcript_857/24291_Confidence_0.000_Length_759, tri_n_comp20573_c1_seq4, vo_c2_Locus_1396_Transcript_1/1_Confidence_0.000_Length_1736, vo_c3_Locus_779_Transcript_28/37_Confidence_0.242_Length_6237, tri_c_comp65239_c0_seq1, vo_n2_Locus_21111_Transcript_1/2_Confidence_0.750_Length_1253, vo_n2_Locus_3_Transcript_856/24291_Confidence_0.000_Length_5013, tri_n_comp20573_c1_seq15, vo_c3_Locus_5178_Transcript_3/7_Confidence_0.455_Length_3313, tri_c_comp64746_c0_seq1, tri_n_comp20573_c1_seq19, vo_c2_Locus_15301_Transcript_1/1_Confidence_0.000_Length_8295, vo_c2_Locus_10960_Transcript_1/1_Confidence_0.000_Length_1278, vo_n1_Locus_3_Transcript_40/53_Confidence_0.223_Length_15279, vo_n2_Locus_3_Transcript_182/24291_Confidence_0.001_Length_1382, vo_n2_Locus_8329_Transcript_2/4_Confidence_0.429_Length_5800, vo_n2_Locus_10955_Transcript_1/1_Confidence_0.000_Length_3181, vo_n1_Locus_1858_Transcript_357/440_Confidence_0.022_Length_542, tri_n_comp20573_c1_seq11, vo_c3_Locus_28441_Transcript_1/1_Confidence_0.000_Length_1746, vo_c1_Locus_3866_Transcript_3/15_Confidence_0.261_Length_4727, tri_c_comp41567_c0_seq1, vo_n3_Locus_134_Transcript_12/14_Confidence_0.144_Length_2345, vo_c3_Locus_15362_Transcript_1/1_Confidence_0.000_Length_338, vo_n1_Locus_17075_Transcript_2/4_Confidence_0.400_Length_811, vo_n1_Locus_2896_Transcript_50/57_Confidence_0.030_Length_1147, vo_c1_Locus_4_Transcript_142/366_Confidence_0.014_Length_1282, vo_n1_Locus_4173_Transcript_1/1_Confidence_0.000_Length_2426, vo_c1_Locus_4_Transcript_154/366_Confidence_0.015_Length_713, vo_c1_Locus_12156_Transcript_1/2_Confidence_0.333_Length_14626, vo_c2_Locus_579_Transcript_142/548_Confidence_0.060_Length_1143, vo_n1_Locus_2914_Transcript_51/78_Confidence_0.046_Length_2290, vo_c1_Locus_24195_Transcript_1/1_Confidence_0.000_Length_4613, vo_c1_Locus_9802_Transcript_8/22_Confidence_0.545_Length_922, vo_c1_Locus_9802_Transcript_10/22_Confidence_0.591_Length_922, vo_c1_Locus_9802_Transcript_4/22_Confidence_0.364_Length_853, vo_n2_Locus_17976_Transcript_2/12_Confidence_0.600_Length_982, tri_n_comp19532_c0_seq1, vo_n3_Locus_5520_Transcript_13/13_Confidence_0.250_Length_964, vo_c1_Locus_36810_Transcript_1/1_Confidence_0.000_Length_784, vo_n2_Locus_17976_Transcript_11/12_Confidence_0.500_Length_998, vo_n3_Locus_5520_Transcript_4/13_Confidence_0.450_Length_941, vo_n2_Locus_17976_Transcript_6/12_Confidence_0.600_Length_1003

Aminobenzoate degradation	map00627	4	ec:3.5.1.4 - acylamidase, ec:3.5.5.1 - acetonitrilase, ec:2.8.3.8 - CoA-transferase, ec:4.2.1.17 - hydratase	3, 1, 1, 7	vo_c3_Locus_1581_Transcript_3/3_Confidence_0.000_Length_1736, vo_c1_Locus_1298_Transcript_3/6_Confidence_0.625_Length_1775, vo_n2_Locus_157_Transcript_2/4_Confidence_0.286_Length_2059, vo_c2_Locus_31357_Transcript_6/7_Confidence_0.700_Length_1464, vo_c2_Locus_6864_Transcript_3/5_Confidence_0.667_Length_1701, vo_c2_Locus_2261_Transcript_1/2_Confidence_1.000_Length_2564, vo_c3_Locus_5948_Transcript_1/1_Confidence_0.000_Length_1027, vo_n2_Locus_16945_Transcript_1/3_Confidence_0.500_Length_1084, vo_c3_Locus_9925_Transcript_8/11_Confidence_0.263_Length_3550, vo_n1_Locus_11137_Transcript_5/5_Confidence_0.143_Length_2829, vo_n2_Locus_16945_Transcript_3/3_Confidence_0.000_Length_746, vo_n2_Locus_30747_Transcript_1/1_Confidence_0.000_Length_951

Taurine and hypotaurine metabolism	map00430	4	ec:1.4.1.2 - dehydrogenase, ec:1.13.11.19 - dioxygenase, ec:1.13.11.20 - dioxygenase, ec:2.3.2.2 - glutamyl transpeptidase	1, 1, 2, 1	tri_c_comp28026_c0_seq1, vo_c2_Locus_13192_Transcript_5/6_Confidence_0.625_Length_1572, vo_c2_Locus_20699_Transcript_22/34_Confidence_0.180_Length_2604, vo_c2_Locus_20699_Transcript_12/34_Confidence_0.165_Length_2453, vo_n2_Locus_15602_Transcript_2/2_Confidence_0.250_Length_2162

Vitamin B6 metabolism	map00750	4	ec:1.4.3.5 - 5'-phosphate synthase, ec:2.6.1.52 - transaminase, ec:2.7.1.35 - kinase, ec:1.1.1.65 - 4-dehydrogenase	3, 4, 1, 2	vo_n2_Locus_7212_Transcript_6/7_Confidence_0.200_Length_1176, vo_c1_Locus_11209_Transcript_1/1_Confidence_0.000_Length_1909, vo_c2_Locus_1774_Transcript_2/5_Confidence_0.312_Length_1184, vo_n2_Locus_2440_Transcript_3/6_Confidence_0.182_Length_1751, tri_c_comp24934_c0_seq1, vo_c3_Locus_9067_Transcript_6/7_Confidence_0.125_Length_1757, vo_c2_Locus_1886_Transcript_3/5_Confidence_0.444_Length_2764, tri_c_comp52297_c0_seq1, vo_n3_Locus_36384_Transcript_1/1_Confidence_0.000_Length_1316, tri_n_comp17407_c0_seq1

C5-Branched dibasic acid metabolism	map00660	4	ec:2.2.1.6 - synthase, ec:4.1.3.24 - lyase, ec:6.2.1.5 - ligase (ADP-forming), ec:1.1.1.85 - dehydrogenase	1, 15, 28, 2	tri_n_comp26150_c0_seq1, vo_c1_Locus_9668_Transcript_5/11_Confidence_0.391_Length_1245, vo_c2_Locus_3988_Transcript_2/10_Confidence_0.500_Length_1233, vo_n3_Locus_4554_Transcript_5/8_Confidence_0.600_Length_1377, vo_c2_Locus_3988_Transcript_1/10_Confidence_0.607_Length_1361, vo_n1_Locus_2626_Transcript_19/26_Confidence_0.238_Length_3851, vo_c1_Locus_9668_Transcript_4/11_Confidence_0.435_Length_1360, vo_n1_Locus_2626_Transcript_15/26_Confidence_0.262_Length_1311, vo_c1_Locus_9668_Transcript_6/11_Confidence_0.348_Length_1630, vo_n2_Locus_3_Transcript_5064/24291_Confidence_0.000_Length_1233, vo_n3_Locus_4554_Transcript_6/8_Confidence_0.533_Length_1337, vo_c2_Locus_3988_Transcript_5/10_Confidence_0.536_Length_2785, vo_n1_Locus_2626_Transcript_18/26_Confidence_0.310_Length_2079, vo_c2_Locus_3988_Transcript_7/10_Confidence_0.500_Length_1273, vo_n2_Locus_3_Transcript_5069/24291_Confidence_0.000_Length_1240, vo_c2_Locus_3988_Transcript_9/10_Confidence_0.429_Length_1401, vo_c1_Locus_2207_Transcript_20/51_Confidence_0.393_Length_3556, vo_c3_Locus_2283_Transcript_28/38_Confidence_0.331_Length_3501, vo_n3_Locus_1164_Transcript_30/46_Confidence_0.400_Length_3745, tri_c_comp20184_c0_seq2, vo_c2_Locus_7270_Transcript_1/1_Confidence_0.500_Length_1488, vo_c1_Locus_31_Transcript_859/1155_Confidence_0.002_Length_1257, vo_n3_Locus_1164_Transcript_32/46_Confidence_0.456_Length_3745, vo_n1_Locus_3448_Transcript_46/64_Confidence_0.085_Length_783, vo_c2_Locus_644_Transcript_12/40_Confidence_0.459_Length_3665, vo_n3_Locus_1164_Transcript_35/46_Confidence_0.511_Length_3745, vo_c3_Locus_5_Transcript_229/353_Confidence_0.004_Length_952, vo_c2_Locus_644_Transcript_3/40_Confidence_0.071_Length_957, vo_n1_Locus_3448_Transcript_53/64_Confidence_0.225_Length_3567, vo_n2_Locus_386_Transcript_32/33_Confidence_0.208_Length_3664, vo_n1_Locus_3448_Transcript_63/64_Confidence_0.318_Length_3637, vo_n3_Locus_1164_Transcript_33/46_Confidence_0.456_Length_3748, vo_c3_Locus_2283_Transcript_26/38_Confidence_0.285_Length_3501, vo_n2_Locus_386_Transcript_22/33_Confidence_0.376_Length_3568, vo_c3_Locus_2283_Transcript_29/38_Confidence_0.292_Length_3501, vo_n2_Locus_386_Transcript_1/33_Confidence_0.139_Length_3600, vo_c2_Locus_644_Transcript_24/40_Confidence_0.398_Length_3511, vo_n3_Locus_1164_Transcript_43/46_Confidence_0.389_Length_3338, vo_c1_Locus_2207_Transcript_39/51_Confidence_0.250_Length_3586, vo_c3_Locus_79_Transcript_1/3_Confidence_0.200_Length_5222, vo_c2_Locus_52_Transcript_234/466_Confidence_0.003_Length_1462, vo_n2_Locus_386_Transcript_10/33_Confidence_0.376_Length_3676, vo_n1_Locus_1394_Transcript_2/3_Confidence_0.400_Length_2232, tri_n_comp19507_c0_seq6, vo_n1_Locus_6924_Transcript_4/6_Confidence_0.444_Length_1647, vo_n1_Locus_6924_Transcript_3/6_Confidence_0.222_Length_1163

Synthesis and degradation of ketone bodies	map00072	4	ec:2.3.1.9 - C-acetyltransferase, ec:2.3.3.10 - synthase, ec:2.8.3.5 - CoA-transferase, ec:1.1.1.30 - dehydrogenase	2, 4, 1, 6	vo_c1_Locus_2657_Transcript_1/1_Confidence_0.000_Length_1513, vo_n2_Locus_23058_Transcript_1/1_Confidence_0.000_Length_1342, vo_n2_Locus_634_Transcript_4/6_Confidence_0.357_Length_8557, tri_c_comp57035_c0_seq1, vo_c1_Locus_1809_Transcript_1/1_Confidence_0.000_Length_3087, vo_c3_Locus_18718_Transcript_1/1_Confidence_0.000_Length_2686, vo_c2_Locus_6864_Transcript_3/5_Confidence_0.667_Length_1701, vo_c3_Locus_1735_Transcript_2/3_Confidence_0.333_Length_8270, vo_c2_Locus_9268_Transcript_2/5_Confidence_0.500_Length_8293, vo_n2_Locus_10545_Transcript_1/1_Confidence_0.000_Length_1731, vo_n2_Locus_12693_Transcript_1/1_Confidence_0.000_Length_2082, vo_n2_Locus_19729_Transcript_5/6_Confidence_0.500_Length_1106, vo_c1_Locus_1033_Transcript_1/1_Confidence_0.000_Length_1983

Glycosphingolipid biosynthesis - globo and isoglobo series	map00603	4	ec:3.2.1.52 - hexosaminidase, ec:2.4.1.69 - 1 galactoside alpha-(1,2)-fucosyltransferase, ec:3.2.1.22 - melibiase, ec:2.4.99.4 - alpha-2,3-sialyltransferase	13, 6, 5, 4	vo_c3_Locus_25279_Transcript_1/3_Confidence_0.500_Length_3460, tri_c_comp15706_c0_seq2, vo_n2_Locus_4554_Transcript_3/4_Confidence_0.444_Length_2253, vo_n2_Locus_3_Transcript_18735/24291_Confidence_0.000_Length_2121, vo_n2_Locus_4395_Transcript_3/3_Confidence_0.167_Length_2032, vo_c2_Locus_6406_Transcript_1/4_Confidence_0.333_Length_1666, vo_n1_Locus_14758_Transcript_1/2_Confidence_0.667_Length_1933, tri_n_comp35667_c0_seq1, vo_n2_Locus_4395_Transcript_1/3_Confidence_0.667_Length_1925, tri_c_comp32936_c0_seq1, tri_n_comp92955_c0_seq1, tri_n_comp30287_c0_seq1, vo_n2_Locus_4554_Transcript_4/4_Confidence_0.111_Length_2049, tri_n_comp19717_c0_seq12, vo_c3_Locus_23760_Transcript_6/6_Confidence_0.000_Length_2139, vo_c2_Locus_20640_Transcript_3/3_Confidence_0.000_Length_2001, vo_n3_Locus_30096_Transcript_2/2_Confidence_0.000_Length_2064, tri_n_comp19717_c0_seq5, tri_c_comp19907_c0_seq6, vo_c2_Locus_17150_Transcript_58/102_Confidence_0.019_Length_2519, vo_n2_Locus_11259_Transcript_1/2_Confidence_0.333_Length_2880, tri_c_comp44396_c0_seq1, vo_n2_Locus_31986_Transcript_2/2_Confidence_0.333_Length_2185, vo_n2_Locus_20375_Transcript_1/2_Confidence_0.333_Length_1577, vo_c2_Locus_30916_Transcript_1/3_Confidence_0.600_Length_1243, vo_n2_Locus_15072_Transcript_3/7_Confidence_0.429_Length_1062, vo_n2_Locus_15072_Transcript_4/7_Confidence_0.286_Length_628, vo_c1_Locus_6341_Transcript_8/13_Confidence_0.611_Length_1057

Caprolactam degradation	map00930	4	ec:1.1.1.2 - dehydrogenase (NADP+), ec:1.14.15.3 - 1-monooxygenase, ec:4.2.1.17 - hydratase, ec:1.1.1.35 - dehydrogenase	10, 1, 7, 16	tri_c_comp11242_c0_seq2, tri_n_comp64914_c0_seq1, vo_c2_Locus_9062_Transcript_10/11_Confidence_0.312_Length_4945, vo_n2_Locus_22224_Transcript_1/1_Confidence_0.000_Length_1370, tri_n_comp14915_c0_seq1, vo_n3_Locus_15713_Transcript_4/7_Confidence_0.429_Length_2557, tri_c_comp37399_c0_seq1, tri_n_comp58061_c0_seq1, vo_n1_Locus_22736_Transcript_1/2_Confidence_0.333_Length_1213, vo_n2_Locus_19677_Transcript_1/1_Confidence_0.000_Length_2823, vo_n2_Locus_2042_Transcript_5/7_Confidence_0.231_Length_2650, vo_c2_Locus_2261_Transcript_1/2_Confidence_1.000_Length_2564, vo_c3_Locus_5948_Transcript_1/1_Confidence_0.000_Length_1027, vo_n2_Locus_16945_Transcript_1/3_Confidence_0.500_Length_1084, vo_c3_Locus_9925_Transcript_8/11_Confidence_0.263_Length_3550, vo_n1_Locus_11137_Transcript_5/5_Confidence_0.143_Length_2829, vo_n2_Locus_16945_Transcript_3/3_Confidence_0.000_Length_746, vo_n2_Locus_30747_Transcript_1/1_Confidence_0.000_Length_951, vo_c2_Locus_2261_Transcript_1/2_Confidence_1.000_Length_2564, vo_n1_Locus_9414_Transcript_8/13_Confidence_0.167_Length_606, vo_c1_Locus_40339_Transcript_1/1_Confidence_0.000_Length_1318, vo_c3_Locus_2031_Transcript_8/8_Confidence_0.111_Length_1389, vo_n1_Locus_11137_Transcript_5/5_Confidence_0.143_Length_2829, vo_c1_Locus_4675_Transcript_3/3_Confidence_0.200_Length_1348, vo_n2_Locus_30747_Transcript_1/1_Confidence_0.000_Length_951, vo_n2_Locus_636_Transcript_13/16_Confidence_0.222_Length_768, vo_c1_Locus_2815_Transcript_3/4_Confidence_0.200_Length_2244, vo_c3_Locus_9925_Transcript_8/11_Confidence_0.263_Length_3550, tri_n_comp42565_c0_seq1, vo_c2_Locus_189_Transcript_29/60_Confidence_0.015_Length_692, vo_n2_Locus_636_Transcript_16/16_Confidence_0.139_Length_616, vo_n2_Locus_636_Transcript_14/16_Confidence_0.222_Length_622, vo_n2_Locus_636_Transcript_5/16_Confidence_0.194_Length_591, vo_n2_Locus_636_Transcript_3/16_Confidence_0.306_Length_685

Drug metabolism - cytochrome P450	map00982	4	ec:2.5.1.18 - transferase, ec:1.2.1.5 - dehydrogenase [NAD(P)+], ec:2.4.1.17 - 1-naphthol glucuronyltransferase, ec:1.14.13.8 - monooxygenase	11, 2, 13, 7	vo_c3_Locus_7919_Transcript_7/13_Confidence_0.524_Length_1057, vo_c3_Locus_483_Transcript_11/12_Confidence_0.217_Length_759, vo_c2_Locus_8227_Transcript_3/3_Confidence_0.143_Length_1106, vo_c2_Locus_8227_Transcript_2/3_Confidence_0.571_Length_1060, vo_n2_Locus_34681_Transcript_5/6_Confidence_0.077_Length_2256, vo_c1_Locus_15275_Transcript_1/2_Confidence_0.333_Length_968, vo_n2_Locus_8358_Transcript_1/8_Confidence_0.444_Length_1513, vo_n2_Locus_2232_Transcript_6/7_Confidence_0.455_Length_2038, vo_c2_Locus_1790_Transcript_5/10_Confidence_0.474_Length_1047, vo_n2_Locus_8358_Transcript_6/8_Confidence_0.389_Length_1051, tri_n_comp38105_c0_seq1, tri_c_comp55340_c0_seq1, tri_n_comp58061_c0_seq1, tri_c_comp6209_c0_seq1, vo_c1_Locus_26340_Transcript_5/9_Confidence_0.600_Length_1699, tri_n_comp65990_c0_seq1, vo_n1_Locus_13372_Transcript_1/2_Confidence_0.333_Length_1687, vo_n2_Locus_34154_Transcript_3/3_Confidence_0.000_Length_2043, vo_n2_Locus_668_Transcript_17/17_Confidence_0.070_Length_2403, tri_c_comp58135_c0_seq1, vo_c3_Locus_16093_Transcript_1/1_Confidence_0.000_Length_1750, vo_n1_Locus_13372_Transcript_2/2_Confidence_0.000_Length_1314, vo_c2_Locus_24841_Transcript_3/3_Confidence_0.167_Length_3241, vo_n2_Locus_4774_Transcript_1/1_Confidence_0.000_Length_884, vo_c1_Locus_35615_Transcript_1/1_Confidence_0.000_Length_2075, vo_n1_Locus_15639_Transcript_1/1_Confidence_0.000_Length_1086, vo_c2_Locus_9064_Transcript_9/10_Confidence_0.125_Length_1602, vo_c2_Locus_18959_Transcript_7/18_Confidence_0.526_Length_1935, vo_c1_Locus_8021_Transcript_2/4_Confidence_0.500_Length_2019, tri_c_comp37849_c0_seq1, vo_c1_Locus_8021_Transcript_1/4_Confidence_0.625_Length_3553, vo_n1_Locus_4659_Transcript_2/2_Confidence_0.000_Length_1515, vo_n2_Locus_2921_Transcript_81/114_Confidence_0.048_Length_2190

Retinol metabolism	map00830	4	ec:1.2.1.36 - dehydrogenase, ec:1.1.1.105 - dehydrogenase (NAD+), ec:2.4.1.17 - 1-naphthol glucuronyltransferase, ec:1.14.15.3 - 1-monooxygenase	4, 1, 13, 1	vo_n1_Locus_10504_Transcript_2/2_Confidence_0.000_Length_1272, vo_c2_Locus_9062_Transcript_10/11_Confidence_0.312_Length_4945, tri_c_comp22527_c0_seq1, vo_c2_Locus_9062_Transcript_1/11_Confidence_0.312_Length_4797, vo_n2_Locus_15985_Transcript_2/3_Confidence_0.750_Length_1260, tri_c_comp6209_c0_seq1, vo_c1_Locus_26340_Transcript_5/9_Confidence_0.600_Length_1699, tri_n_comp65990_c0_seq1, vo_n1_Locus_13372_Transcript_1/2_Confidence_0.333_Length_1687, vo_n2_Locus_34154_Transcript_3/3_Confidence_0.000_Length_2043, vo_n2_Locus_668_Transcript_17/17_Confidence_0.070_Length_2403, tri_c_comp58135_c0_seq1, vo_c3_Locus_16093_Transcript_1/1_Confidence_0.000_Length_1750, vo_n1_Locus_13372_Transcript_2/2_Confidence_0.000_Length_1314, vo_c2_Locus_24841_Transcript_3/3_Confidence_0.167_Length_3241, vo_n2_Locus_4774_Transcript_1/1_Confidence_0.000_Length_884, vo_c1_Locus_35615_Transcript_1/1_Confidence_0.000_Length_2075, vo_n1_Locus_15639_Transcript_1/1_Confidence_0.000_Length_1086, vo_n2_Locus_2042_Transcript_5/7_Confidence_0.231_Length_2650

Glycosphingolipid biosynthesis - ganglio series	map00604	3	ec:3.2.1.52 - hexosaminidase, ec:3.2.1.23 - lactase (ambiguous), ec:2.4.99.4 - alpha-2,3-sialyltransferase	13, 6, 4	vo_c3_Locus_25279_Transcript_1/3_Confidence_0.500_Length_3460, tri_c_comp15706_c0_seq2, vo_n2_Locus_4554_Transcript_3/4_Confidence_0.444_Length_2253, vo_n2_Locus_3_Transcript_18735/24291_Confidence_0.000_Length_2121, vo_n2_Locus_4395_Transcript_3/3_Confidence_0.167_Length_2032, vo_c2_Locus_6406_Transcript_1/4_Confidence_0.333_Length_1666, vo_n1_Locus_14758_Transcript_1/2_Confidence_0.667_Length_1933, tri_n_comp35667_c0_seq1, vo_n2_Locus_4395_Transcript_1/3_Confidence_0.667_Length_1925, tri_c_comp32936_c0_seq1, tri_n_comp92955_c0_seq1, tri_n_comp30287_c0_seq1, vo_n2_Locus_4554_Transcript_4/4_Confidence_0.111_Length_2049, tri_c_comp12867_c0_seq1, vo_n2_Locus_1748_Transcript_2/3_Confidence_0.333_Length_4221, tri_n_comp70995_c0_seq1, vo_c2_Locus_26351_Transcript_1/2_Confidence_0.500_Length_4013, vo_c3_Locus_8047_Transcript_1/2_Confidence_0.333_Length_4021, vo_c3_Locus_32079_Transcript_2/2_Confidence_0.250_Length_2113, vo_c2_Locus_30916_Transcript_1/3_Confidence_0.600_Length_1243, vo_n2_Locus_15072_Transcript_3/7_Confidence_0.429_Length_1062, vo_n2_Locus_15072_Transcript_4/7_Confidence_0.286_Length_628, vo_c1_Locus_6341_Transcript_8/13_Confidence_0.611_Length_1057

Flavonoid biosynthesis	map00941	3	ec:1.3.1.77 - reductase [(2R,3R)-flavan-3-ol-forming], ec:5.5.1.6 - isomerase, ec:2.1.1.104 - O-methyltransferase	1, 1, 2	vo_n2_Locus_20001_Transcript_1/1_Confidence_0.000_Length_1186, vo_c1_Locus_26362_Transcript_2/4_Confidence_0.667_Length_1019, vo_n2_Locus_30574_Transcript_4/4_Confidence_0.000_Length_969, vo_n2_Locus_30574_Transcript_3/4_Confidence_0.500_Length_827

Metabolism of xenobiotics by cytochrome P450	map00980	3	ec:2.5.1.18 - transferase, ec:1.2.1.5 - dehydrogenase [NAD(P)+], ec:2.4.1.17 - 1-naphthol glucuronyltransferase	11, 2, 13	vo_c3_Locus_7919_Transcript_7/13_Confidence_0.524_Length_1057, vo_c3_Locus_483_Transcript_11/12_Confidence_0.217_Length_759, vo_c2_Locus_8227_Transcript_3/3_Confidence_0.143_Length_1106, vo_c2_Locus_8227_Transcript_2/3_Confidence_0.571_Length_1060, vo_n2_Locus_34681_Transcript_5/6_Confidence_0.077_Length_2256, vo_c1_Locus_15275_Transcript_1/2_Confidence_0.333_Length_968, vo_n2_Locus_8358_Transcript_1/8_Confidence_0.444_Length_1513, vo_n2_Locus_2232_Transcript_6/7_Confidence_0.455_Length_2038, vo_c2_Locus_1790_Transcript_5/10_Confidence_0.474_Length_1047, vo_n2_Locus_8358_Transcript_6/8_Confidence_0.389_Length_1051, tri_n_comp38105_c0_seq1, tri_c_comp55340_c0_seq1, tri_n_comp58061_c0_seq1, tri_c_comp6209_c0_seq1, vo_c1_Locus_26340_Transcript_5/9_Confidence_0.600_Length_1699, tri_n_comp65990_c0_seq1, vo_n1_Locus_13372_Transcript_1/2_Confidence_0.333_Length_1687, vo_n2_Locus_34154_Transcript_3/3_Confidence_0.000_Length_2043, vo_n2_Locus_668_Transcript_17/17_Confidence_0.070_Length_2403, tri_c_comp58135_c0_seq1, vo_c3_Locus_16093_Transcript_1/1_Confidence_0.000_Length_1750, vo_n1_Locus_13372_Transcript_2/2_Confidence_0.000_Length_1314, vo_c2_Locus_24841_Transcript_3/3_Confidence_0.167_Length_3241, vo_n2_Locus_4774_Transcript_1/1_Confidence_0.000_Length_884, vo_c1_Locus_35615_Transcript_1/1_Confidence_0.000_Length_2075, vo_n1_Locus_15639_Transcript_1/1_Confidence_0.000_Length_1086

Polyketide sugar unit biosynthesis	map00523	3	ec:1.1.1.133 - reductase, ec:5.1.3.13 - 3,5-epimerase, ec:4.2.1.46 - 4,6-dehydratase	2, 1, 2	vo_c2_Locus_1643_Transcript_1/1_Confidence_0.000_Length_1756, vo_c1_Locus_3806_Transcript_1/1_Confidence_0.000_Length_1482, vo_c2_Locus_1643_Transcript_1/1_Confidence_0.000_Length_1756, vo_c2_Locus_5377_Transcript_5/9_Confidence_0.150_Length_2142, vo_n1_Locus_5366_Transcript_4/4_Confidence_0.556_Length_1623

Benzoate degradation	map00362	3	ec:2.3.1.9 - C-acetyltransferase, ec:4.2.1.17 - hydratase, ec:1.1.1.35 - dehydrogenase	2, 7, 16	vo_c1_Locus_2657_Transcript_1/1_Confidence_0.000_Length_1513, vo_n2_Locus_23058_Transcript_1/1_Confidence_0.000_Length_1342, vo_c2_Locus_2261_Transcript_1/2_Confidence_1.000_Length_2564, vo_c3_Locus_5948_Transcript_1/1_Confidence_0.000_Length_1027, vo_n2_Locus_16945_Transcript_1/3_Confidence_0.500_Length_1084, vo_c3_Locus_9925_Transcript_8/11_Confidence_0.263_Length_3550, vo_n1_Locus_11137_Transcript_5/5_Confidence_0.143_Length_2829, vo_n2_Locus_16945_Transcript_3/3_Confidence_0.000_Length_746, vo_n2_Locus_30747_Transcript_1/1_Confidence_0.000_Length_951, vo_c2_Locus_2261_Transcript_1/2_Confidence_1.000_Length_2564, vo_n1_Locus_9414_Transcript_8/13_Confidence_0.167_Length_606, vo_c1_Locus_40339_Transcript_1/1_Confidence_0.000_Length_1318, vo_c3_Locus_2031_Transcript_8/8_Confidence_0.111_Length_1389, vo_n1_Locus_11137_Transcript_5/5_Confidence_0.143_Length_2829, vo_c1_Locus_4675_Transcript_3/3_Confidence_0.200_Length_1348, vo_n2_Locus_30747_Transcript_1/1_Confidence_0.000_Length_951, vo_n2_Locus_636_Transcript_13/16_Confidence_0.222_Length_768, vo_c1_Locus_2815_Transcript_3/4_Confidence_0.200_Length_2244, vo_c3_Locus_9925_Transcript_8/11_Confidence_0.263_Length_3550, tri_n_comp42565_c0_seq1, vo_c2_Locus_189_Transcript_29/60_Confidence_0.015_Length_692, vo_n2_Locus_636_Transcript_16/16_Confidence_0.139_Length_616, vo_n2_Locus_636_Transcript_14/16_Confidence_0.222_Length_622, vo_n2_Locus_636_Transcript_5/16_Confidence_0.194_Length_591, vo_n2_Locus_636_Transcript_3/16_Confidence_0.306_Length_685

Chloroalkane and chloroalkene degradation	map00625	3	ec:1.2.1.46 - dehydrogenase, ec:1.2.1.3 - dehydrogenase (NAD+), ec:3.3.2.10 - epoxide hydrolase	2, 5, 3	vo_c3_Locus_11405_Transcript_4/5_Confidence_0.429_Length_4324, vo_c3_Locus_15849_Transcript_1/1_Confidence_0.000_Length_1425, tri_c_comp55340_c0_seq1, vo_n1_Locus_19864_Transcript_2/2_Confidence_0.000_Length_2707, vo_c3_Locus_21741_Transcript_5/9_Confidence_0.583_Length_1757, vo_n3_Locus_25600_Transcript_5/6_Confidence_0.375_Length_1777, tri_n_comp58061_c0_seq1, vo_n2_Locus_16627_Transcript_1/2_Confidence_0.333_Length_2167, vo_n2_Locus_13706_Transcript_2/3_Confidence_0.750_Length_2463, tri_n_comp17047_c0_seq1

Glycosaminoglycan degradation	map00531	3	ec:3.1.6.14 - chondroitinsulfatase, ec:3.2.1.52 - hexosaminidase, ec:3.2.1.23 - lactase (ambiguous)	1, 13, 6	tri_c_comp17496_c0_seq2, vo_c3_Locus_25279_Transcript_1/3_Confidence_0.500_Length_3460, tri_c_comp15706_c0_seq2, vo_n2_Locus_4554_Transcript_3/4_Confidence_0.444_Length_2253, vo_n2_Locus_3_Transcript_18735/24291_Confidence_0.000_Length_2121, vo_n2_Locus_4395_Transcript_3/3_Confidence_0.167_Length_2032, vo_c2_Locus_6406_Transcript_1/4_Confidence_0.333_Length_1666, vo_n1_Locus_14758_Transcript_1/2_Confidence_0.667_Length_1933, tri_n_comp35667_c0_seq1, vo_n2_Locus_4395_Transcript_1/3_Confidence_0.667_Length_1925, tri_c_comp32936_c0_seq1, tri_n_comp92955_c0_seq1, tri_n_comp30287_c0_seq1, vo_n2_Locus_4554_Transcript_4/4_Confidence_0.111_Length_2049, tri_c_comp12867_c0_seq1, vo_n2_Locus_1748_Transcript_2/3_Confidence_0.333_Length_4221, tri_n_comp70995_c0_seq1, vo_c2_Locus_26351_Transcript_1/2_Confidence_0.500_Length_4013, vo_c3_Locus_8047_Transcript_1/2_Confidence_0.333_Length_4021, vo_c3_Locus_32079_Transcript_2/2_Confidence_0.250_Length_2113

alpha-Linolenic acid metabolism	map00592	3	ec:3.1.1.4 - A2, ec:1.3.3.6 - oxidase, ec:4.2.1.17 - hydratase	59, 2, 7	vo_n2_Locus_311_Transcript_71/114_Confidence_0.150_Length_1045, vo_c3_Locus_7729_Transcript_4/5_Confidence_0.167_Length_5654, vo_n1_Locus_443_Transcript_30/53_Confidence_0.193_Length_928, vo_c1_Locus_430_Transcript_20/51_Confidence_0.291_Length_976, vo_c2_Locus_601_Transcript_31/36_Confidence_0.229_Length_1002, vo_c2_Locus_5092_Transcript_6/14_Confidence_0.476_Length_1090, vo_c3_Locus_1328_Transcript_24/26_Confidence_0.245_Length_1398, vo_n2_Locus_311_Transcript_78/114_Confidence_0.093_Length_844, vo_c3_Locus_1273_Transcript_51/57_Confidence_0.108_Length_895, vo_c1_Locus_21671_Transcript_6/49_Confidence_0.021_Length_1919, vo_c3_Locus_1273_Transcript_50/57_Confidence_0.144_Length_1471, vo_n1_Locus_443_Transcript_24/53_Confidence_0.109_Length_905, vo_n2_Locus_311_Transcript_87/114_Confidence_0.061_Length_2919, vo_n3_Locus_407_Transcript_22/43_Confidence_0.292_Length_1222, tri_n_comp10070_c0_seq2, tri_c_comp22021_c0_seq1, vo_n1_Locus_443_Transcript_32/53_Confidence_0.252_Length_1164, vo_n3_Locus_407_Transcript_32/43_Confidence_0.283_Length_1271, vo_n1_Locus_443_Transcript_10/53_Confidence_0.193_Length_1076, vo_n3_Locus_407_Transcript_7/43_Confidence_0.132_Length_1778, vo_n2_Locus_311_Transcript_109/114_Confidence_0.117_Length_1018, vo_n3_Locus_407_Transcript_39/43_Confidence_0.236_Length_1222, vo_c3_Locus_15467_Transcript_2/2_Confidence_0.000_Length_1100, vo_n1_Locus_4880_Transcript_2/6_Confidence_0.333_Length_883, vo_n2_Locus_3013_Transcript_1/2_Confidence_0.750_Length_964, vo_n2_Locus_311_Transcript_112/114_Confidence_0.146_Length_1678, vo_c2_Locus_601_Transcript_27/36_Confidence_0.337_Length_991, vo_c2_Locus_601_Transcript_2/36_Confidence_0.133_Length_1744, vo_n1_Locus_443_Transcript_39/53_Confidence_0.210_Length_1938, vo_c3_Locus_15467_Transcript_1/2_Confidence_0.750_Length_988, vo_c1_Locus_430_Transcript_11/51_Confidence_0.145_Length_955, vo_c2_Locus_5092_Transcript_10/14_Confidence_0.429_Length_1495, vo_n2_Locus_21867_Transcript_2/5_Confidence_0.200_Length_5139, tri_n_comp20432_c0_seq6, vo_n2_Locus_311_Transcript_84/114_Confidence_0.117_Length_1124, vo_n2_Locus_311_Transcript_77/114_Confidence_0.134_Length_997, vo_n2_Locus_311_Transcript_90/114_Confidence_0.061_Length_1016, vo_c2_Locus_601_Transcript_11/36_Confidence_0.289_Length_1016, tri_n_comp20432_c0_seq2, vo_c1_Locus_430_Transcript_48/51_Confidence_0.227_Length_968, vo_n1_Locus_443_Transcript_17/53_Confidence_0.168_Length_1009, tri_c_comp8925_c1_seq1, vo_c2_Locus_601_Transcript_18/36_Confidence_0.265_Length_801, vo_c3_Locus_1273_Transcript_17/57_Confidence_0.050_Length_1126, vo_n3_Locus_407_Transcript_40/43_Confidence_0.217_Length_1200, vo_c2_Locus_601_Transcript_36/36_Confidence_0.217_Length_953, vo_n3_Locus_407_Transcript_8/43_Confidence_0.189_Length_1800, vo_c3_Locus_1273_Transcript_16/57_Confidence_0.079_Length_1101, tri_n_comp20662_c0_seq7, vo_n2_Locus_23060_Transcript_2/2_Confidence_0.000_Length_889, vo_c2_Locus_601_Transcript_15/36_Confidence_0.337_Length_815, vo_n1_Locus_4880_Transcript_4/6_Confidence_0.333_Length_1109, vo_n2_Locus_311_Transcript_3/114_Confidence_0.142_Length_866, tri_c_comp20860_c0_seq2, vo_n1_Locus_443_Transcript_8/53_Confidence_0.261_Length_1031, vo_n1_Locus_443_Transcript_49/53_Confidence_0.134_Length_862, vo_n3_Locus_407_Transcript_14/43_Confidence_0.142_Length_872, vo_c2_Locus_5609_Transcript_4/4_Confidence_0.250_Length_1105, vo_c3_Locus_17900_Transcript_1/1_Confidence_0.000_Length_966, tri_n_comp36670_c0_seq1, vo_n1_Locus_23625_Transcript_1/1_Confidence_0.000_Length_2286, vo_c2_Locus_2261_Transcript_1/2_Confidence_1.000_Length_2564, vo_c3_Locus_5948_Transcript_1/1_Confidence_0.000_Length_1027, vo_n2_Locus_16945_Transcript_1/3_Confidence_0.500_Length_1084, vo_c3_Locus_9925_Transcript_8/11_Confidence_0.263_Length_3550, vo_n1_Locus_11137_Transcript_5/5_Confidence_0.143_Length_2829, vo_n2_Locus_16945_Transcript_3/3_Confidence_0.000_Length_746, vo_n2_Locus_30747_Transcript_1/1_Confidence_0.000_Length_951

Folate biosynthesis	map00790	3	ec:1.5.1.3 - reductase, ec:6.3.2.17 - synthase, ec:1.5.1.34 - reductase	1, 1, 1	vo_c2_Locus_1969_Transcript_1/2_Confidence_0.200_Length_1807, tri_c_comp50174_c0_seq1, vo_c2_Locus_12026_Transcript_1/2_Confidence_1.000_Length_911

Isoquinoline alkaloid biosynthesis	map00950	3	ec:2.6.1.1 - transaminase, ec:1.14.16.2 - 3-monooxygenase, ec:1.4.3.21 - oxidase	4, 2, 1	vo_n1_Locus_3423_Transcript_2/4_Confidence_0.250_Length_1347, tri_n_comp27683_c0_seq1, vo_c1_Locus_3127_Transcript_1/1_Confidence_0.000_Length_1478, vo_n1_Locus_3423_Transcript_3/4_Confidence_0.250_Length_1416, vo_c2_Locus_16025_Transcript_2/3_Confidence_0.400_Length_2217, vo_n2_Locus_6286_Transcript_1/1_Confidence_0.000_Length_1634, tri_n_comp48625_c0_seq1

Indole alkaloid biosynthesis	map00901	3	ec:2.4.1.219 - glucosyltransferase, ec:3.2.1.125 - beta-glucosidase, ec:3.2.1.105 - beta-glucosidase	1, 1, 3	tri_n_comp70995_c0_seq1, tri_n_comp70995_c0_seq1, tri_c_comp12867_c0_seq1, tri_n_comp70995_c0_seq1, vo_c3_Locus_32079_Transcript_2/2_Confidence_0.250_Length_2113

Styrene degradati on	map00643	2	ec:3.5.1.4 - acylamidase, ec:3.5.5.1 - acetonitrilase	3, 1	vo_c3_Locus_1581_Transcript_3/3_Confidence_0.000_Length_1736, vo_c1_Locus_1298_Transcript_3/6_Confidence_0.625_Length_1775, vo_n2_Locus_157_Transcript_2/4_Confidence_0.286_Length_2059, vo_c2_Locus_31357_Transcript_6/7_Confidence_0.700_Length_1464

mTOR signaling pathway	map04150	2	ec:2.7.11.11 - protein kinase, ec:2.7.11.24 - protein kinase	67, 27	vo_c1_Locus_23955_Transcript_1/1_Confidence_0.000_Length_1690, vo_n2_Locus_1515_Transcript_10/24_Confidence_0.205_Length_885, tri_c_comp35466_c0_seq1, vo_c2_Locus_23610_Transcript_5/5_Confidence_0.222_Length_462, vo_n2_Locus_19015_Transcript_1/1_Confidence_0.000_Length_2784, vo_c3_Locus_12823_Transcript_7/11_Confidence_0.150_Length_1099, vo_n2_Locus_18927_Transcript_1/1_Confidence_0.000_Length_2769, vo_c1_Locus_3372_Transcript_11/12_Confidence_0.350_Length_1415, vo_c1_Locus_6709_Transcript_7/10_Confidence_0.500_Length_1389, vo_c3_Locus_1810_Transcript_8/22_Confidence_0.262_Length_1483, vo_n1_Locus_1051_Transcript_2/7_Confidence_0.556_Length_3162, vo_n2_Locus_1426_Transcript_24/28_Confidence_0.304_Length_1547, vo_c1_Locus_10052_Transcript_5/5_Confidence_0.100_Length_3554, vo_n2_Locus_10323_Transcript_2/2_Confidence_0.000_Length_2787, vo_n2_Locus_3_Transcript_17800/24291_Confidence_0.000_Length_5238, vo_n3_Locus_1253_Transcript_1/2_Confidence_0.500_Length_1626, vo_n1_Locus_8984_Transcript_1/1_Confidence_0.000_Length_2588, tri_n_comp95070_c0_seq1, vo_n1_Locus_842_Transcript_34/37_Confidence_0.203_Length_1893, vo_n3_Locus_1253_Transcript_2/2_Confidence_0.000_Length_1587, tri_n_comp17425_c0_seq2, vo_c3_Locus_4005_Transcript_5/6_Confidence_0.167_Length_1517, vo_c2_Locus_10357_Transcript_15/15_Confidence_0.045_Length_698, vo_c3_Locus_12823_Transcript_5/11_Confidence_0.200_Length_1070, vo_c2_Locus_4581_Transcript_3/8_Confidence_0.684_Length_1578, vo_c3_Locus_1810_Transcript_17/22_Confidence_0.214_Length_1421, tri_c_comp62706_c0_seq1, vo_n2_Locus_18804_Transcript_2/2_Confidence_0.000_Length_2997, vo_n1_Locus_14687_Transcript_3/3_Confidence_0.200_Length_1029, vo_c1_Locus_7903_Transcript_2/3_Confidence_0.750_Length_3162, vo_n2_Locus_3054_Transcript_2/3_Confidence_0.333_Length_3045, vo_c2_Locus_1080_Transcript_3/21_Confidence_0.207_Length_1702, vo_n2_Locus_3_Transcript_19709/24291_Confidence_0.000_Length_2745, tri_c_comp18638_c0_seq3, tri_c_comp12452_c0_seq2, vo_c1_Locus_6709_Transcript_2/10_Confidence_0.444_Length_1193, vo_c2_Locus_13626_Transcript_1/1_Confidence_0.500_Length_2755, vo_n2_Locus_3053_Transcript_1/1_Confidence_0.000_Length_2618, vo_n3_Locus_8058_Transcript_1/1_Confidence_0.000_Length_2950, tri_n_comp12148_c0_seq1, vo_c1_Locus_6477_Transcript_5/9_Confidence_0.235_Length_1167, vo_n2_Locus_1515_Transcript_15/24_Confidence_0.077_Length_1136, vo_c3_Locus_10023_Transcript_2/6_Confidence_0.125_Length_1290, vo_n2_Locus_1426_Transcript_22/28_Confidence_0.304_Length_1443, tri_n_comp11583_c0_seq1, vo_n1_Locus_1576_Transcript_8/13_Confidence_0.160_Length_2301, vo_n2_Locus_19434_Transcript_1/2_Confidence_0.333_Length_3274, vo_c1_Locus_21343_Transcript_3/9_Confidence_0.600_Length_2048, vo_n2_Locus_297_Transcript_2/5_Confidence_0.636_Length_1679, vo_c3_Locus_8311_Transcript_1/2_Confidence_0.333_Length_2973, vo_n1_Locus_14424_Transcript_4/4_Confidence_0.143_Length_7320, vo_n3_Locus_877_Transcript_3/5_Confidence_0.400_Length_1624, vo_c1_Locus_4147_Transcript_11/12_Confidence_0.278_Length_2504, vo_c2_Locus_11635_Transcript_2/4_Confidence_0.167_Length_3028, vo_c3_Locus_1810_Transcript_9/22_Confidence_0.262_Length_1447, tri_c_comp47538_c0_seq1, vo_c1_Locus_4147_Transcript_10/12_Confidence_0.222_Length_2379, vo_c1_Locus_6811_Transcript_7/8_Confidence_0.364_Length_589, vo_c1_Locus_6709_Transcript_9/10_Confidence_0.278_Length_1468, vo_c2_Locus_1080_Transcript_4/21_Confidence_0.241_Length_1741, vo_n2_Locus_5856_Transcript_8/8_Confidence_0.000_Length_3276, vo_c2_Locus_1080_Transcript_8/21_Confidence_0.138_Length_1077, vo_n1_Locus_842_Transcript_11/37_Confidence_0.188_Length_2091, vo_n2_Locus_901_Transcript_1/95_Confidence_0.023_Length_1512, vo_n2_Locus_1426_Transcript_12/28_Confidence_0.370_Length_1887, vo_n2_Locus_1426_Transcript_16/28_Confidence_0.326_Length_1443, vo_c3_Locus_1810_Transcript_5/22_Confidence_0.310_Length_1343, vo_c3_Locus_4105_Transcript_3/6_Confidence_0.417_Length_1637, vo_c1_Locus_6691_Transcript_2/2_Confidence_0.250_Length_1740, vo_c1_Locus_23003_Transcript_4/8_Confidence_0.667_Length_1396, vo_n3_Locus_8055_Transcript_2/5_Confidence_0.400_Length_1985, vo_n1_Locus_4081_Transcript_2/4_Confidence_0.500_Length_1840, tri_n_comp30548_c0_seq1, vo_c1_Locus_23003_Transcript_3/8_Confidence_0.667_Length_1323, tri_c_comp34910_c0_seq1, vo_c1_Locus_13237_Transcript_1/9_Confidence_0.176_Length_513, vo_c3_Locus_19377_Transcript_2/2_Confidence_0.000_Length_1548, tri_c_comp40129_c0_seq1, vo_n2_Locus_729_Transcript_27/52_Confidence_0.360_Length_2376, vo_n2_Locus_5122_Transcript_1/1_Confidence_0.333_Length_1529, vo_c2_Locus_15642_Transcript_1/1_Confidence_0.000_Length_1576, tri_c_comp34272_c0_seq1, vo_c2_Locus_8726_Transcript_6/6_Confidence_0.040_Length_1556, tri_c_comp31416_c0_seq1, tri_n_comp13449_c0_seq1, vo_c3_Locus_2111_Transcript_2/6_Confidence_0.625_Length_1653, vo_n1_Locus_16685_Transcript_3/3_Confidence_0.286_Length_1498, vo_n1_Locus_4081_Transcript_1/4_Confidence_0.667_Length_1798, vo_c3_Locus_4105_Transcript_4/6_Confidence_0.250_Length_1291, vo_c1_Locus_12069_Transcript_1/3_Confidence_0.667_Length_1533, vo_c1_Locus_4044_Transcript_3/3_Confidence_0.854_Length_1776, vo_c3_Locus_17929_Transcript_1/2_Confidence_0.333_Length_490, vo_n1_Locus_16685_Transcript_1/3_Confidence_0.429_Length_1465, vo_n2_Locus_729_Transcript_30/52_Confidence_0.360_Length_2337

Novobiocin biosynthesis	map00401	2	ec:2.6.1.1 - transaminase, ec:1.3.1.12 - dehydrogenase	4, 1	vo_n1_Locus_3423_Transcript_2/4_Confidence_0.250_Length_1347, tri_n_comp27683_c0_seq1, vo_c1_Locus_3127_Transcript_1/1_Confidence_0.000_Length_1478, vo_n1_Locus_3423_Transcript_3/4_Confidence_0.250_Length_1416, vo_c2_Locus_15226_Transcript_2/2_Confidence_0.500_Length_2141

Tropane, piperidine and pyridine alkaloid biosynthesis	map00960	2	ec:2.6.1.1 - transaminase, ec:1.4.3.21 - oxidase	4, 1	vo_n1_Locus_3423_Transcript_2/4_Confidence_0.250_Length_1347, tri_n_comp27683_c0_seq1, vo_c1_Locus_3127_Transcript_1/1_Confidence_0.000_Length_1478, vo_n1_Locus_3423_Transcript_3/4_Confidence_0.250_Length_1416, tri_n_comp48625_c0_seq1

Glycosaminoglycan biosynthesis - keratan sulfate	map00533	2	ec:2.4.1.38 - beta-1,4-galactosyltransferase, ec:2.4.99.4 - alpha-2,3-sialyltransferase	2, 4	tri_c_comp20165_c0_seq7, tri_c_comp20165_c0_seq3, vo_c2_Locus_30916_Transcript_1/3_Confidence_0.600_Length_1243, vo_n2_Locus_15072_Transcript_3/7_Confidence_0.429_Length_1062, vo_n2_Locus_15072_Transcript_4/7_Confidence_0.286_Length_628, vo_c1_Locus_6341_Transcript_8/13_Confidence_0.611_Length_1057
Ubiquinone and other terpenoid-quinone biosynthesis	map00130	2	ec:2.2.1.9 - synthase, ec:1.6.5.2 - dehydrogenase (quinone)	1, 1	tri_n_comp40118_c0_seq1, vo_n1_Locus_3253_Transcript_7/7_Confidence_0.000_Length_907

Primary bile acid biosynthesis	map00120	2	ec:4.2.1.107 - hydratase, ec:1.1.1.35 - dehydrogenase	8, 16	vo_n1_Locus_9414_Transcript_8/13_Confidence_0.167_Length_606, vo_c2_Locus_189_Transcript_29/60_Confidence_0.015_Length_692, vo_c3_Locus_2031_Transcript_8/8_Confidence_0.111_Length_1389, vo_n2_Locus_636_Transcript_16/16_Confidence_0.139_Length_616, vo_n2_Locus_636_Transcript_14/16_Confidence_0.222_Length_622, vo_n2_Locus_636_Transcript_5/16_Confidence_0.194_Length_591, vo_n2_Locus_30747_Transcript_1/1_Confidence_0.000_Length_951, vo_n2_Locus_636_Transcript_13/16_Confidence_0.222_Length_768, vo_c2_Locus_2261_Transcript_1/2_Confidence_1.000_Length_2564, vo_n1_Locus_9414_Transcript_8/13_Confidence_0.167_Length_606, vo_c1_Locus_40339_Transcript_1/1_Confidence_0.000_Length_1318, vo_c3_Locus_2031_Transcript_8/8_Confidence_0.111_Length_1389, vo_n1_Locus_11137_Transcript_5/5_Confidence_0.143_Length_2829, vo_c1_Locus_4675_Transcript_3/3_Confidence_0.200_Length_1348, vo_n2_Locus_30747_Transcript_1/1_Confidence_0.000_Length_951, vo_n2_Locus_636_Transcript_13/16_Confidence_0.222_Length_768, vo_c1_Locus_2815_Transcript_3/4_Confidence_0.200_Length_2244, vo_c3_Locus_9925_Transcript_8/11_Confidence_0.263_Length_3550, tri_n_comp42565_c0_seq1, vo_c2_Locus_189_Transcript_29/60_Confidence_0.015_Length_692, vo_n2_Locus_636_Transcript_16/16_Confidence_0.139_Length_616, vo_n2_Locus_636_Transcript_14/16_Confidence_0.222_Length_622, vo_n2_Locus_636_Transcript_5/16_Confidence_0.194_Length_591, vo_n2_Locus_636_Transcript_3/16_Confidence_0.306_Length_685

Mucin type O-glycan biosynthesis	map00512	2	ec:2.4.1.41 - N-acetylgalactosaminyltransferase, ec:2.4.99.4 - alpha-2,3-sialyltransferase	12, 4	tri_c_comp18925_c0_seq4, vo_c3_Locus_31796_Transcript_4/4_Confidence_0.000_Length_1398, tri_c_comp36211_c0_seq1, vo_n1_Locus_3055_Transcript_5/6_Confidence_0.083_Length_2608, vo_c2_Locus_26791_Transcript_1/1_Confidence_0.000_Length_2049, vo_c2_Locus_14086_Transcript_1/1_Confidence_0.000_Length_2122, vo_n2_Locus_10812_Transcript_5/6_Confidence_0.625_Length_2773, vo_n1_Locus_26507_Transcript_2/2_Confidence_0.000_Length_1797, tri_c_comp4176_c0_seq2, tri_c_comp65027_c0_seq1, vo_c1_Locus_3502_Transcript_2/4_Confidence_0.667_Length_1718, vo_n2_Locus_24382_Transcript_1/1_Confidence_0.000_Length_2045, vo_c2_Locus_30916_Transcript_1/3_Confidence_0.600_Length_1243, vo_n2_Locus_15072_Transcript_3/7_Confidence_0.429_Length_1062, vo_n2_Locus_15072_Transcript_4/7_Confidence_0.286_Length_628, vo_c1_Locus_6341_Transcript_8/13_Confidence_0.611_Length_1057

Geraniol degradation	map00281	2	ec:4.2.1.17 - hydratase, ec:1.1.1.35 - dehydrogenase	7, 16	vo_c2_Locus_2261_Transcript_1/2_Confidence_1.000_Length_2564, vo_c3_Locus_5948_Transcript_1/1_Confidence_0.000_Length_1027, vo_n2_Locus_16945_Transcript_1/3_Confidence_0.500_Length_1084, vo_c3_Locus_9925_Transcript_8/11_Confidence_0.263_Length_3550, vo_n1_Locus_11137_Transcript_5/5_Confidence_0.143_Length_2829, vo_n2_Locus_16945_Transcript_3/3_Confidence_0.000_Length_746, vo_n2_Locus_30747_Transcript_1/1_Confidence_0.000_Length_951, vo_c2_Locus_2261_Transcript_1/2_Confidence_1.000_Length_2564, vo_n1_Locus_9414_Transcript_8/13_Confidence_0.167_Length_606, vo_c1_Locus_40339_Transcript_1/1_Confidence_0.000_Length_1318, vo_c3_Locus_2031_Transcript_8/8_Confidence_0.111_Length_1389, vo_n1_Locus_11137_Transcript_5/5_Confidence_0.143_Length_2829, vo_c1_Locus_4675_Transcript_3/3_Confidence_0.200_Length_1348, vo_n2_Locus_30747_Transcript_1/1_Confidence_0.000_Length_951, vo_n2_Locus_636_Transcript_13/16_Confidence_0.222_Length_768, vo_c1_Locus_2815_Transcript_3/4_Confidence_0.200_Length_2244, vo_c3_Locus_9925_Transcript_8/11_Confidence_0.263_Length_3550, tri_n_comp42565_c0_seq1, vo_c2_Locus_189_Transcript_29/60_Confidence_0.015_Length_692, vo_n2_Locus_636_Transcript_16/16_Confidence_0.139_Length_616, vo_n2_Locus_636_Transcript_14/16_Confidence_0.222_Length_622, vo_n2_Locus_636_Transcript_5/16_Confidence_0.194_Length_591, vo_n2_Locus_636_Transcript_3/16_Confidence_0.306_Length_685

Limonene and pinene degradation	map00903	2	ec:1.2.1.3 - dehydrogenase (NAD+), ec:4.2.1.17 - hydratase	5, 7	tri_c_comp55340_c0_seq1, vo_n1_Locus_19864_Transcript_2/2_Confidence_0.000_Length_2707, vo_c3_Locus_21741_Transcript_5/9_Confidence_0.583_Length_1757, vo_n3_Locus_25600_Transcript_5/6_Confidence_0.375_Length_1777, tri_n_comp58061_c0_seq1, vo_c2_Locus_2261_Transcript_1/2_Confidence_1.000_Length_2564, vo_c3_Locus_5948_Transcript_1/1_Confidence_0.000_Length_1027, vo_n2_Locus_16945_Transcript_1/3_Confidence_0.500_Length_1084, vo_c3_Locus_9925_Transcript_8/11_Confidence_0.263_Length_3550, vo_n1_Locus_11137_Transcript_5/5_Confidence_0.143_Length_2829, vo_n2_Locus_16945_Transcript_3/3_Confidence_0.000_Length_746, vo_n2_Locus_30747_Transcript_1/1_Confidence_0.000_Length_951

Glycosaminoglycan biosynthesis - heparan sulfate / heparin	map00534	2	ec:2.4.1.224 - 4-alpha-N-acetylglucosaminyltransferase, ec:2.4.1.225 - 4-beta-glucuronosyltransferase	2, 2	vo_c1_Locus_23908_Transcript_1/1_Confidence_0.000_Length_2129, vo_c2_Locus_13150_Transcript_3/3_Confidence_0.000_Length_2135, vo_c1_Locus_23908_Transcript_1/1_Confidence_0.000_Length_2129, vo_c2_Locus_13150_Transcript_3/3_Confidence_0.000_Length_2135

Photosynt hesis	map00195	2	ec:1.18.1.2 - reductase, ec:1.10.9.1 - reductase	22, 11	vo_c3_Locus_6967_Transcript_1/1_Confidence_0.000_Length_1495, vo_c3_Locus_348_Transcript_22/28_Confidence_0.452_Length_1847, vo_n2_Locus_1693_Transcript_26/55_Confidence_0.182_Length_1396, vo_n2_Locus_1693_Transcript_37/55_Confidence_0.121_Length_1392, vo_n2_Locus_1693_Transcript_55/55_Confidence_0.101_Length_1537, vo_n1_Locus_1081_Transcript_31/59_Confidence_0.333_Length_2089, vo_n1_Locus_1081_Transcript_52/59_Confidence_0.126_Length_1389, vo_n1_Locus_1081_Transcript_30/59_Confidence_0.379_Length_2044, vo_n1_Locus_3201_Transcript_21/28_Confidence_0.421_Length_1518, vo_c3_Locus_348_Transcript_5/28_Confidence_0.429_Length_1341, vo_n2_Locus_1693_Transcript_27/55_Confidence_0.232_Length_1396, vo_n1_Locus_1081_Transcript_55/59_Confidence_0.241_Length_1447, vo_c1_Locus_505_Transcript_6/25_Confidence_0.667_Length_1385, vo_n1_Locus_3201_Transcript_11/28_Confidence_0.447_Length_1802, vo_c2_Locus_2807_Transcript_18/26_Confidence_0.538_Length_1367, vo_c2_Locus_2807_Transcript_6/26_Confidence_0.538_Length_1417, vo_n1_Locus_1081_Transcript_46/59_Confidence_0.299_Length_1475, vo_n1_Locus_1081_Transcript_34/59_Confidence_0.241_Length_1734, vo_n2_Locus_1693_Transcript_15/55_Confidence_0.222_Length_4326, tri_c_comp19032_c0_seq2, vo_n1_Locus_1081_Transcript_39/59_Confidence_0.333_Length_1640, vo_n2_Locus_1693_Transcript_12/55_Confidence_0.222_Length_1409, vo_c2_Locus_5878_Transcript_5/7_Confidence_0.333_Length_1049, vo_c1_Locus_754_Transcript_8/11_Confidence_0.200_Length_971, vo_c1_Locus_754_Transcript_9/11_Confidence_0.200_Length_2339, vo_c1_Locus_8997_Transcript_2/2_Confidence_0.000_Length_877, vo_c3_Locus_6650_Transcript_4/8_Confidence_0.308_Length_1102, vo_c1_Locus_754_Transcript_5/11_Confidence_0.300_Length_1714, vo_c2_Locus_5878_Transcript_3/7_Confidence_0.444_Length_1038, vo_n1_Locus_1379_Transcript_7/9_Confidence_0.154_Length_925, vo_c2_Locus_455_Transcript_3/3_Confidence_0.000_Length_684, vo_n3_Locus_3061_Transcript_4/7_Confidence_0.417_Length_922, vo_c1_Locus_754_Transcript_7/11_Confidence_0.250_Length_1706

Steroid degradation	map00984	2	ec:1.1.1.145 - dehydrogenase, ec:1.1.1.51 - 17)beta-hydroxysteroid dehydrogenase	2, 9	vo_c2_Locus_4806_Transcript_29/66_Confidence_0.103_Length_1536, vo_c3_Locus_11279_Transcript_9/10_Confidence_0.136_Length_1385, vo_n1_Locus_9414_Transcript_8/13_Confidence_0.167_Length_606, vo_c2_Locus_189_Transcript_29/60_Confidence_0.015_Length_692, vo_c3_Locus_2031_Transcript_8/8_Confidence_0.111_Length_1389, vo_n2_Locus_636_Transcript_16/16_Confidence_0.139_Length_616, vo_n2_Locus_636_Transcript_14/16_Confidence_0.222_Length_622, vo_n2_Locus_636_Transcript_5/16_Confidence_0.194_Length_591, vo_n2_Locus_636_Transcript_3/16_Confidence_0.306_Length_685, vo_n2_Locus_30747_Transcript_1/1_Confidence_0.000_Length_951, vo_n2_Locus_636_Transcript_13/16_Confidence_0.222_Length_768

Carotenoid biosynthesis	map00906	2	ec:2.5.1.32 - synthase, ec:1.13.11.51 - dioxygenase	1, 1	vo_c2_Locus_7097_Transcript_3/3_Confidence_0.000_Length_3511, vo_n3_Locus_17738_Transcript_2/2_Confidence_0.000_Length_687

T cell receptor signaling pathway	map04660	2	ec:2.7.10.2 - protein-tyrosine kinase, ec:3.1.3.16 - phosphatase	3, 98	vo_n1_Locus_304_Transcript_19/31_Confidence_0.364_Length_3278, vo_n1_Locus_304_Transcript_26/31_Confidence_0.352_Length_5345, tri_n_comp49150_c0_seq1, tri_c_comp42222_c0_seq1, tri_n_comp11728_c0_seq1, tri_n_comp11728_c0_seq2, vo_n2_Locus_618_Transcript_2/13_Confidence_0.167_Length_2515, vo_c1_Locus_1075_Transcript_27/66_Confidence_0.029_Length_2812, vo_n1_Locus_10083_Transcript_2/5_Confidence_0.200_Length_5459, vo_c3_Locus_1110_Transcript_23/23_Confidence_0.171_Length_1349, vo_c2_Locus_2829_Transcript_16/21_Confidence_0.421_Length_2869, tri_c_comp13504_c0_seq2, vo_n3_Locus_406_Transcript_4/22_Confidence_0.581_Length_1350, vo_n2_Locus_183_Transcript_41/101_Confidence_0.031_Length_3376, vo_c1_Locus_14246_Transcript_1/6_Confidence_0.538_Length_3413, vo_n2_Locus_12759_Transcript_1/1_Confidence_0.000_Length_1292, vo_n2_Locus_820_Transcript_22/27_Confidence_0.436_Length_4411, vo_c2_Locus_19330_Transcript_4/8_Confidence_0.625_Length_1112, vo_c1_Locus_3292_Transcript_10/17_Confidence_0.208_Length_1553, tri_n_comp15472_c0_seq1, vo_c1_Locus_17659_Transcript_2/5_Confidence_0.429_Length_3424, vo_n1_Locus_1557_Transcript_12/32_Confidence_0.413_Length_1400, tri_c_comp39423_c0_seq1, vo_c2_Locus_325_Transcript_1/5_Confidence_0.273_Length_3600, vo_c3_Locus_20940_Transcript_2/2_Confidence_0.000_Length_1114, vo_n1_Locus_1557_Transcript_7/32_Confidence_0.283_Length_3416, vo_n2_Locus_32740_Transcript_1/2_Confidence_0.333_Length_1824, vo_c1_Locus_11577_Transcript_6/6_Confidence_0.000_Length_903, tri_n_comp43002_c0_seq1, vo_n3_Locus_1745_Transcript_1/1_Confidence_0.333_Length_1610, tri_c_comp46798_c0_seq1, vo_c2_Locus_34481_Transcript_1/2_Confidence_0.667_Length_1801, vo_n1_Locus_23893_Transcript_1/1_Confidence_0.000_Length_1778, tri_c_comp57853_c0_seq1, vo_c1_Locus_169_Transcript_13/33_Confidence_0.257_Length_1323, tri_c_comp29751_c0_seq1, tri_c_comp16460_c0_seq2, vo_c1_Locus_6126_Transcript_1/2_Confidence_0.667_Length_1159, vo_n3_Locus_8585_Transcript_2/3_Confidence_0.400_Length_1473, vo_c3_Locus_35940_Transcript_1/1_Confidence_0.000_Length_1207, vo_n1_Locus_19163_Transcript_1/1_Confidence_0.000_Length_3417, vo_c1_Locus_7585_Transcript_12/13_Confidence_0.500_Length_1730, vo_c1_Locus_169_Transcript_10/33_Confidence_0.300_Length_1343, vo_c3_Locus_4701_Transcript_1/2_Confidence_0.833_Length_1495, vo_n1_Locus_1594_Transcript_8/12_Confidence_0.529_Length_3454, tri_n_comp13698_c0_seq2, tri_c_comp8446_c0_seq1, vo_n1_Locus_1557_Transcript_17/32_Confidence_0.304_Length_985, vo_c2_Locus_15966_Transcript_1/1_Confidence_0.000_Length_1558, vo_c1_Locus_1081_Transcript_12/28_Confidence_0.279_Length_1129, vo_n2_Locus_820_Transcript_10/27_Confidence_0.418_Length_1385, vo_c2_Locus_3834_Transcript_23/50_Confidence_0.062_Length_3121, vo_n3_Locus_406_Transcript_14/22_Confidence_0.516_Length_1465, tri_n_comp61518_c0_seq1, vo_n1_Locus_1557_Transcript_22/32_Confidence_0.239_Length_1016, vo_n2_Locus_20461_Transcript_3/4_Confidence_0.375_Length_3394, vo_n1_Locus_6468_Transcript_2/2_Confidence_0.000_Length_1048, vo_c1_Locus_29320_Transcript_1/1_Confidence_0.000_Length_2103, vo_c3_Locus_4822_Transcript_1/2_Confidence_0.714_Length_1522, vo_n1_Locus_471_Transcript_13/17_Confidence_0.355_Length_2286, tri_n_comp6659_c0_seq1, vo_c2_Locus_19330_Transcript_2/8_Confidence_0.625_Length_1071, tri_c_comp19264_c0_seq1, tri_c_comp19264_c0_seq5, tri_c_comp36977_c0_seq1, vo_n1_Locus_10488_Transcript_9/10_Confidence_0.312_Length_1034, vo_c1_Locus_4899_Transcript_11/22_Confidence_0.429_Length_2516, vo_n1_Locus_1557_Transcript_14/32_Confidence_0.413_Length_1398, vo_n2_Locus_479_Transcript_39/48_Confidence_0.029_Length_4102, tri_n_comp19137_c0_seq4, vo_c2_Locus_6204_Transcript_1/3_Confidence_0.714_Length_1299, vo_c1_Locus_7585_Transcript_13/13_Confidence_0.250_Length_1594, vo_c2_Locus_911_Transcript_5/24_Confidence_0.205_Length_2131, vo_c3_Locus_756_Transcript_2/3_Confidence_0.400_Length_2477, vo_c1_Locus_11577_Transcript_4/6_Confidence_0.222_Length_639, vo_c2_Locus_507_Transcript_12/22_Confidence_0.447_Length_1412, vo_n2_Locus_820_Transcript_14/27_Confidence_0.382_Length_1635, vo_n2_Locus_820_Transcript_5/27_Confidence_0.382_Length_1475, vo_n3_Locus_1224_Transcript_33/39_Confidence_0.173_Length_3279, vo_n3_Locus_16559_Transcript_1/4_Confidence_0.833_Length_3357, vo_n1_Locus_3668_Transcript_1/1_Confidence_0.000_Length_1350, vo_c1_Locus_7585_Transcript_10/13_Confidence_0.562_Length_1842, vo_n2_Locus_8141_Transcript_2/2_Confidence_0.250_Length_1579, vo_c1_Locus_7585_Transcript_11/13_Confidence_0.562_Length_1777, vo_c1_Locus_6537_Transcript_1/3_Confidence_0.400_Length_3118, vo_c1_Locus_1075_Transcript_28/66_Confidence_0.032_Length_3054, vo_n1_Locus_3894_Transcript_1/1_Confidence_0.000_Length_1928, vo_c1_Locus_14246_Transcript_3/6_Confidence_0.615_Length_3488, vo_c3_Locus_1110_Transcript_19/23_Confidence_0.537_Length_1687, vo_n3_Locus_1772_Transcript_1/2_Confidence_0.333_Length_1879, vo_n2_Locus_3822_Transcript_12/14_Confidence_0.562_Length_2066, vo_c1_Locus_23835_Transcript_2/2_Confidence_0.167_Length_1401, vo_n2_Locus_21630_Transcript_1/2_Confidence_1.000_Length_2263, vo_n2_Locus_13821_Transcript_1/2_Confidence_0.833_Length_1344, vo_c2_Locus_32056_Transcript_1/1_Confidence_0.000_Length_1722, vo_c1_Locus_14246_Transcript_4/6_Confidence_0.462_Length_3446, vo_n2_Locus_820_Transcript_2/27_Confidence_0.200_Length_1373, vo_c2_Locus_33927_Transcript_1/2_Confidence_0.333_Length_1530, vo_c3_Locus_21550_Transcript_4/4_Confidence_0.000_Length_602, vo_c3_Locus_12812_Transcript_5/5_Confidence_0.143_Length_6845, vo_n1_Locus_471_Transcript_17/17_Confidence_0.032_Length_2196

Glycosphingolipid biosynthesis - lacto and neolacto series	map00601	2	ec:2.4.1.69 - 1 galactoside alpha-(1,2)-fucosyltransferase, ec:2.4.99.4 - alpha-2,3-sialyltransferase	6, 4	tri_n_comp19717_c0_seq12, vo_c3_Locus_23760_Transcript_6/6_Confidence_0.000_Length_2139, vo_c2_Locus_20640_Transcript_3/3_Confidence_0.000_Length_2001, vo_n3_Locus_30096_Transcript_2/2_Confidence_0.000_Length_2064, tri_n_comp19717_c0_seq5, tri_c_comp19907_c0_seq6, vo_c2_Locus_30916_Transcript_1/3_Confidence_0.600_Length_1243, vo_n2_Locus_15072_Transcript_3/7_Confidence_0.429_Length_1062, vo_n2_Locus_15072_Transcript_4/7_Confidence_0.286_Length_628, vo_c1_Locus_6341_Transcript_8/13_Confidence_0.611_Length_1057

Lipopolysaccharide biosynthesis	map00540	1	ec:2.3.1.129 - O-acyltransferase	3	vo_n2_Locus_8711_Transcript_5/5_Confidence_0.067_Length_1558, vo_c2_Locus_11984_Transcript_2/6_Confidence_0.385_Length_1557, tri_c_comp30049_c0_seq1
Lipoic acid metabolism	map00785	1	ec:2.8.1.8 - synthase	1	tri_c_comp28689_c0_seq1

Toluene degradati on	map00623	1	ec:1.1.1.35 - dehydrogenase	16	vo_c2_Locus_2261_Transcript_1/2_Confidence_1.000_Length_2564, vo_n1_Locus_9414_Transcript_8/13_Confidence_0.167_Length_606, vo_c1_Locus_40339_Transcript_1/1_Confidence_0.000_Length_1318, vo_c3_Locus_2031_Transcript_8/8_Confidence_0.111_Length_1389, vo_n1_Locus_11137_Transcript_5/5_Confidence_0.143_Length_2829, vo_c1_Locus_4675_Transcript_3/3_Confidence_0.200_Length_1348, vo_n2_Locus_30747_Transcript_1/1_Confidence_0.000_Length_951, vo_n2_Locus_636_Transcript_13/16_Confidence_0.222_Length_768, vo_c1_Locus_2815_Transcript_3/4_Confidence_0.200_Length_2244, vo_c3_Locus_9925_Transcript_8/11_Confidence_0.263_Length_3550, tri_n_comp42565_c0_seq1, vo_c2_Locus_189_Transcript_29/60_Confidence_0.015_Length_692, vo_n2_Locus_636_Transcript_16/16_Confidence_0.139_Length_616, vo_n2_Locus_636_Transcript_14/16_Confidence_0.222_Length_622, vo_n2_Locus_636_Transcript_5/16_Confidence_0.194_Length_591, vo_n2_Locus_636_Transcript_3/16_Confidence_0.306_Length_685

Other types of O-glycan biosynthesis	map00514	1	ec:2.4.99.1 - alpha-(2,6)-sialyltransferase	6	vo_n1_Locus_4002_Transcript_6/7_Confidence_0.091_Length_1369, tri_c_comp4119_c0_seq1, vo_n1_Locus_4002_Transcript_4/7_Confidence_0.636_Length_1286, vo_n2_Locus_15072_Transcript_4/7_Confidence_0.286_Length_628, tri_c_comp6583_c0_seq1, tri_n_comp6433_c0_seq1

Linoleic acid metabolism	map00591	1	ec:3.1.1.4 - A2	59	vo_n2_Locus_311_Transcript_71/114_Confidence_0.150_Length_1045, vo_c3_Locus_7729_Transcript_4/5_Confidence_0.167_Length_5654, vo_n1_Locus_443_Transcript_30/53_Confidence_0.193_Length_928, vo_c1_Locus_430_Transcript_20/51_Confidence_0.291_Length_976, vo_c2_Locus_601_Transcript_31/36_Confidence_0.229_Length_1002, vo_c2_Locus_5092_Transcript_6/14_Confidence_0.476_Length_1090, vo_c3_Locus_1328_Transcript_24/26_Confidence_0.245_Length_1398, vo_n2_Locus_311_Transcript_78/114_Confidence_0.093_Length_844, vo_c3_Locus_1273_Transcript_51/57_Confidence_0.108_Length_895, vo_c1_Locus_21671_Transcript_6/49_Confidence_0.021_Length_1919, vo_c3_Locus_1273_Transcript_50/57_Confidence_0.144_Length_1471, vo_n1_Locus_443_Transcript_24/53_Confidence_0.109_Length_905, vo_n2_Locus_311_Transcript_87/114_Confidence_0.061_Length_2919, vo_n3_Locus_407_Transcript_22/43_Confidence_0.292_Length_1222, tri_n_comp10070_c0_seq2, tri_c_comp22021_c0_seq1, vo_n1_Locus_443_Transcript_32/53_Confidence_0.252_Length_1164, vo_n3_Locus_407_Transcript_32/43_Confidence_0.283_Length_1271, vo_n1_Locus_443_Transcript_10/53_Confidence_0.193_Length_1076, vo_n3_Locus_407_Transcript_7/43_Confidence_0.132_Length_1778, vo_n2_Locus_311_Transcript_109/114_Confidence_0.117_Length_1018, vo_n3_Locus_407_Transcript_39/43_Confidence_0.236_Length_1222, vo_c3_Locus_15467_Transcript_2/2_Confidence_0.000_Length_1100, vo_n1_Locus_4880_Transcript_2/6_Confidence_0.333_Length_883, vo_n2_Locus_3013_Transcript_1/2_Confidence_0.750_Length_964, vo_n2_Locus_311_Transcript_112/114_Confidence_0.146_Length_1678, vo_c2_Locus_601_Transcript_27/36_Confidence_0.337_Length_991, vo_c2_Locus_601_Transcript_2/36_Confidence_0.133_Length_1744, vo_n1_Locus_443_Transcript_39/53_Confidence_0.210_Length_1938, vo_c3_Locus_15467_Transcript_1/2_Confidence_0.750_Length_988, vo_c1_Locus_430_Transcript_11/51_Confidence_0.145_Length_955, vo_c2_Locus_5092_Transcript_10/14_Confidence_0.429_Length_1495, vo_n2_Locus_21867_Transcript_2/5_Confidence_0.200_Length_5139, tri_n_comp20432_c0_seq6, vo_n2_Locus_311_Transcript_84/114_Confidence_0.117_Length_1124, vo_n2_Locus_311_Transcript_77/114_Confidence_0.134_Length_997, vo_n2_Locus_311_Transcript_90/114_Confidence_0.061_Length_1016, vo_c2_Locus_601_Transcript_11/36_Confidence_0.289_Length_1016, tri_n_comp20432_c0_seq2, vo_c1_Locus_430_Transcript_48/51_Confidence_0.227_Length_968, vo_n1_Locus_443_Transcript_17/53_Confidence_0.168_Length_1009, tri_c_comp8925_c1_seq1, vo_c2_Locus_601_Transcript_18/36_Confidence_0.265_Length_801, vo_c3_Locus_1273_Transcript_17/57_Confidence_0.050_Length_1126, vo_n3_Locus_407_Transcript_40/43_Confidence_0.217_Length_1200, vo_c2_Locus_601_Transcript_36/36_Confidence_0.217_Length_953, vo_n3_Locus_407_Transcript_8/43_Confidence_0.189_Length_1800, vo_c3_Locus_1273_Transcript_16/57_Confidence_0.079_Length_1101, tri_n_comp20662_c0_seq7, vo_n2_Locus_23060_Transcript_2/2_Confidence_0.000_Length_889, vo_c2_Locus_601_Transcript_15/36_Confidence_0.337_Length_815, vo_n1_Locus_4880_Transcript_4/6_Confidence_0.333_Length_1109, vo_n2_Locus_311_Transcript_3/114_Confidence_0.142_Length_866, tri_c_comp20860_c0_seq2, vo_n1_Locus_443_Transcript_8/53_Confidence_0.261_Length_1031, vo_n1_Locus_443_Transcript_49/53_Confidence_0.134_Length_862, vo_n3_Locus_407_Transcript_14/43_Confidence_0.142_Length_872, vo_c2_Locus_5609_Transcript_4/4_Confidence_0.250_Length_1105, vo_c3_Locus_17900_Transcript_1/1_Confidence_0.000_Length_966

Flavone and flavonol biosynthesis	map00944	1	ec:2.8.2.25 - 3-sulfotransferase	1	vo_c3_Locus_1509_Transcript_1/2_Confidence_0.750_Length_1318

Th1 and Th2 cell differentiation	map04658	1	ec:3.1.3.16 - phosphatase	98	tri_c_comp42222_c0_seq1, tri_n_comp11728_c0_seq1, tri_n_comp11728_c0_seq2, vo_n2_Locus_618_Transcript_2/13_Confidence_0.167_Length_2515, vo_c1_Locus_1075_Transcript_27/66_Confidence_0.029_Length_2812, vo_n1_Locus_10083_Transcript_2/5_Confidence_0.200_Length_5459, vo_c3_Locus_1110_Transcript_23/23_Confidence_0.171_Length_1349, vo_c2_Locus_2829_Transcript_16/21_Confidence_0.421_Length_2869, tri_c_comp13504_c0_seq2, vo_n3_Locus_406_Transcript_4/22_Confidence_0.581_Length_1350, vo_n2_Locus_183_Transcript_41/101_Confidence_0.031_Length_3376, vo_c1_Locus_14246_Transcript_1/6_Confidence_0.538_Length_3413, vo_n2_Locus_12759_Transcript_1/1_Confidence_0.000_Length_1292, vo_n2_Locus_820_Transcript_22/27_Confidence_0.436_Length_4411, vo_c2_Locus_19330_Transcript_4/8_Confidence_0.625_Length_1112, vo_c1_Locus_3292_Transcript_10/17_Confidence_0.208_Length_1553, tri_n_comp15472_c0_seq1, vo_c1_Locus_17659_Transcript_2/5_Confidence_0.429_Length_3424, vo_n1_Locus_1557_Transcript_12/32_Confidence_0.413_Length_1400, tri_c_comp39423_c0_seq1, vo_c2_Locus_325_Transcript_1/5_Confidence_0.273_Length_3600, vo_c3_Locus_20940_Transcript_2/2_Confidence_0.000_Length_1114, vo_n1_Locus_1557_Transcript_7/32_Confidence_0.283_Length_3416, vo_n2_Locus_32740_Transcript_1/2_Confidence_0.333_Length_1824, vo_c1_Locus_11577_Transcript_6/6_Confidence_0.000_Length_903, tri_n_comp43002_c0_seq1, vo_n3_Locus_1745_Transcript_1/1_Confidence_0.333_Length_1610, tri_c_comp46798_c0_seq1, vo_c2_Locus_34481_Transcript_1/2_Confidence_0.667_Length_1801, vo_n1_Locus_23893_Transcript_1/1_Confidence_0.000_Length_1778, tri_c_comp57853_c0_seq1, vo_c1_Locus_169_Transcript_13/33_Confidence_0.257_Length_1323, tri_c_comp29751_c0_seq1, tri_c_comp16460_c0_seq2, vo_c1_Locus_6126_Transcript_1/2_Confidence_0.667_Length_1159, vo_n3_Locus_8585_Transcript_2/3_Confidence_0.400_Length_1473, vo_c3_Locus_35940_Transcript_1/1_Confidence_0.000_Length_1207, vo_n1_Locus_19163_Transcript_1/1_Confidence_0.000_Length_3417, vo_c1_Locus_7585_Transcript_12/13_Confidence_0.500_Length_1730, vo_c1_Locus_169_Transcript_10/33_Confidence_0.300_Length_1343, vo_c3_Locus_4701_Transcript_1/2_Confidence_0.833_Length_1495, vo_n1_Locus_1594_Transcript_8/12_Confidence_0.529_Length_3454, tri_n_comp13698_c0_seq2, tri_c_comp8446_c0_seq1, vo_n1_Locus_1557_Transcript_17/32_Confidence_0.304_Length_985, vo_c2_Locus_15966_Transcript_1/1_Confidence_0.000_Length_1558, vo_c1_Locus_1081_Transcript_12/28_Confidence_0.279_Length_1129, vo_n2_Locus_820_Transcript_10/27_Confidence_0.418_Length_1385, vo_c2_Locus_3834_Transcript_23/50_Confidence_0.062_Length_3121, vo_n3_Locus_406_Transcript_14/22_Confidence_0.516_Length_1465, tri_n_comp61518_c0_seq1, vo_n1_Locus_1557_Transcript_22/32_Confidence_0.239_Length_1016, vo_n2_Locus_20461_Transcript_3/4_Confidence_0.375_Length_3394, vo_n1_Locus_6468_Transcript_2/2_Confidence_0.000_Length_1048, vo_c1_Locus_29320_Transcript_1/1_Confidence_0.000_Length_2103, vo_c3_Locus_4822_Transcript_1/2_Confidence_0.714_Length_1522, vo_n1_Locus_471_Transcript_13/17_Confidence_0.355_Length_2286, tri_n_comp6659_c0_seq1, vo_c2_Locus_19330_Transcript_2/8_Confidence_0.625_Length_1071, tri_c_comp19264_c0_seq1, tri_c_comp19264_c0_seq5, tri_c_comp36977_c0_seq1, vo_n1_Locus_10488_Transcript_9/10_Confidence_0.312_Length_1034, vo_c1_Locus_4899_Transcript_11/22_Confidence_0.429_Length_2516, vo_n1_Locus_1557_Transcript_14/32_Confidence_0.413_Length_1398, vo_n2_Locus_479_Transcript_39/48_Confidence_0.029_Length_4102, tri_n_comp19137_c0_seq4, vo_c2_Locus_6204_Transcript_1/3_Confidence_0.714_Length_1299, vo_c1_Locus_7585_Transcript_13/13_Confidence_0.250_Length_1594, vo_c2_Locus_911_Transcript_5/24_Confidence_0.205_Length_2131, vo_c3_Locus_756_Transcript_2/3_Confidence_0.400_Length_2477, vo_c1_Locus_11577_Transcript_4/6_Confidence_0.222_Length_639, vo_c2_Locus_507_Transcript_12/22_Confidence_0.447_Length_1412, vo_n2_Locus_820_Transcript_14/27_Confidence_0.382_Length_1635, vo_n2_Locus_820_Transcript_5/27_Confidence_0.382_Length_1475, vo_n3_Locus_1224_Transcript_33/39_Confidence_0.173_Length_3279, vo_n3_Locus_16559_Transcript_1/4_Confidence_0.833_Length_3357, vo_n1_Locus_3668_Transcript_1/1_Confidence_0.000_Length_1350, vo_c1_Locus_7585_Transcript_10/13_Confidence_0.562_Length_1842, vo_n2_Locus_8141_Transcript_2/2_Confidence_0.250_Length_1579, vo_c1_Locus_7585_Transcript_11/13_Confidence_0.562_Length_1777, vo_c1_Locus_6537_Transcript_1/3_Confidence_0.400_Length_3118, vo_c1_Locus_1075_Transcript_28/66_Confidence_0.032_Length_3054, vo_n1_Locus_3894_Transcript_1/1_Confidence_0.000_Length_1928, vo_c1_Locus_14246_Transcript_3/6_Confidence_0.615_Length_3488, vo_c3_Locus_1110_Transcript_19/23_Confidence_0.537_Length_1687, vo_n3_Locus_1772_Transcript_1/2_Confidence_0.333_Length_1879, vo_n2_Locus_3822_Transcript_12/14_Confidence_0.562_Length_2066, vo_c1_Locus_23835_Transcript_2/2_Confidence_0.167_Length_1401, vo_n2_Locus_21630_Transcript_1/2_Confidence_1.000_Length_2263, vo_n2_Locus_13821_Transcript_1/2_Confidence_0.833_Length_1344, vo_c2_Locus_32056_Transcript_1/1_Confidence_0.000_Length_1722, vo_c1_Locus_14246_Transcript_4/6_Confidence_0.462_Length_3446, vo_n2_Locus_820_Transcript_2/27_Confidence_0.200_Length_1373, vo_c2_Locus_33927_Transcript_1/2_Confidence_0.333_Length_1530, vo_c3_Locus_21550_Transcript_4/4_Confidence_0.000_Length_602, vo_c3_Locus_12812_Transcript_5/5_Confidence_0.143_Length_6845, vo_n1_Locus_471_Transcript_17/17_Confidence_0.032_Length_2196

Sesquiterpenoid and triterpenoid biosynthesis	map00909	1	ec:2.5.1.21 - synthase	1	vo_n2_Locus_19315_Transcript_2/2_Confidence_0.000_Length_1802
D-Arginine and D-ornithine metabolism	map00472	1	ec:1.4.3.3 - oxidase	1	vo_n2_Locus_26350_Transcript_7/9_Confidence_0.231_Length_1309
Biosynthesis of terpenoids and steroids	map01062	1	ec:2.5.1.32 - synthase	1	vo_c2_Locus_7097_Transcript_3/3_Confidence_0.000_Length_3511
Biosynthesis of vancomycin group antibiotics	map01055	1	ec:4.2.1.46 - 4,6-dehydratase	2	vo_c2_Locus_5377_Transcript_5/9_Confidence_0.150_Length_2142, vo_n1_Locus_5366_Transcript_4/4_Confidence_0.556_Length_1623
Anthocyanin biosynthesis	map00942	1	ec:2.4.1.115 - 3-O-glucosyltransferase	1	tri_c_comp17063_c0_seq3

Insect hormone biosynthesis	map00981	1	ec:1.2.1.3 - dehydrogenase (NAD+)	5	tri_c_comp55340_c0_seq1, vo_n1_Locus_19864_Transcript_2/2_Confidence_0.000_Length_2707, vo_c3_Locus_21741_Transcript_5/9_Confidence_0.583_Length_1757, vo_n3_Locus_25600_Transcript_5/6_Confidence_0.375_Length_1777, tri_n_comp58061_c0_seq1
Acarbose and validamycin biosynthesis	map00525	1	ec:4.2.1.46 - 4,6-dehydratase	2	vo_c2_Locus_5377_Transcript_5/9_Confidence_0.150_Length_2142, vo_n1_Locus_5366_Transcript_4/4_Confidence_0.556_Length_1623

Betalain biosynthe sis	map00965	1	ec:2.1.1.6 - O-methyltransferase	12	vo_c1_Locus_19524_Transcript_9/12_Confidence_0.615_Length_3975, vo_n1_Locus_23463_Transcript_3/4_Confidence_0.286_Length_1512, tri_c_comp32679_c0_seq1, tri_n_comp8749_c0_seq1, vo_c2_Locus_24901_Transcript_2/2_Confidence_0.000_Length_1355, vo_c3_Locus_26293_Transcript_1/4_Confidence_0.750_Length_2211, vo_n2_Locus_17803_Transcript_2/2_Confidence_0.250_Length_2793, tri_n_comp4208_c0_seq2, vo_n2_Locus_17803_Transcript_1/2_Confidence_0.500_Length_2823, vo_n3_Locus_12676_Transcript_1/1_Confidence_0.000_Length_3081, vo_c3_Locus_17106_Transcript_1/2_Confidence_1.000_Length_3041, vo_c3_Locus_26293_Transcript_2/4_Confidence_0.500_Length_2353

Glycosylphosphatidylinositol (GPI)-anchor biosynthesis	map00563	1	ec:2.4.1.198 - N-acetylglucosaminyltransferase	2	vo_c3_Locus_15749_Transcript_2/2_Confidence_0.000_Length_1608, vo_n1_Locus_27865_Transcript_1/1_Confidence_0.000_Length_1578
Neomycin, kanamycin and gentamicin biosynthesis	map00524	1	ec:2.7.1.2 - glucokinase (phosphorylating)	2	tri_c_comp10536_c0_seq1, vo_c2_Locus_32726_Transcript_2/2_Confidence_0.000_Length_696
Stilbenoid, diarylheptanoid and gingerol biosynthesis	map00945	1	ec:2.1.1.104 - O-methyltransferase	2	vo_n2_Locus_30574_Transcript_4/4_Confidence_0.000_Length_969, vo_n2_Locus_30574_Transcript_3/4_Confidence_0.500_Length_827
Atrazine degradation	map00791	1	ec:3.5.1.5 - ec:3.5.1.5 urease	1	tri_c_comp36137_c0_seq1

Penicillin and cephalosporin biosynthesis	map00311	1	ec:1.4.3.3 - oxidase	1	vo_n2_Locus_26350_Transcript_7/9_Confidence_0.231_Length_1309
Bisphenol degradation	map00363	1	ec:3.1.1.2 - A-esterase	1	vo_c1_Locus_5656_Transcript_1/2_Confidence_0.333_Length_1233

Aflatoxin biosynthesis	map00254	1	ec:6.4.1.2 - carboxylase	16	vo_n3_Locus_484_Transcript_6/12_Confidence_0.250_Length_1533, vo_c3_Locus_17332_Transcript_1/1_Confidence_0.333_Length_6493, vo_n3_Locus_696_Transcript_20/35_Confidence_0.473_Length_5396, vo_c3_Locus_2164_Transcript_38/39_Confidence_0.292_Length_6879, tri_n_comp41552_c0_seq1, vo_c2_Locus_1509_Transcript_1/47_Confidence_0.149_Length_2731, vo_c2_Locus_2193_Transcript_1/3_Confidence_0.667_Length_1218, vo_c1_Locus_860_Transcript_10/29_Confidence_0.571_Length_6744, vo_c2_Locus_1509_Transcript_11/47_Confidence_0.164_Length_2686, vo_n2_Locus_1751_Transcript_51/59_Confidence_0.207_Length_3566, vo_n2_Locus_15097_Transcript_1/1_Confidence_0.000_Length_3935, vo_n3_Locus_484_Transcript_5/12_Confidence_0.250_Length_1451, vo_c1_Locus_860_Transcript_11/29_Confidence_0.571_Length_6741, tri_c_comp19913_c0_seq3, vo_n2_Locus_1751_Transcript_37/59_Confidence_0.195_Length_3437, vo_c2_Locus_1509_Transcript_6/47_Confidence_0.149_Length_2592

Secondary bile acid biosynthesis	map00121	1	ec:1.1.1.201 - dehydrogenase (NADP+)	1	vo_c1_Locus_2815_Transcript_3/4_Confidence_0.200_Length_2244
Diterpenoid biosynthesis	map00904	1	ec:1.14.11.13 - 2beta-dioxygenase	2	vo_n1_Locus_5731_Transcript_4/4_Confidence_0.000_Length_912, vo_c2_Locus_31803_Transcript_1/2_Confidence_0.750_Length_1130
Melanogenesis	map04916	1	ec:5.3.3.12 - isomerase	3	vo_c2_Locus_19107_Transcript_2/4_Confidence_0.571_Length_752, vo_c1_Locus_20614_Transcript_4/6_Confidence_0.273_Length_420, vo_c2_Locus_19107_Transcript_1/4_Confidence_0.571_Length_831

Glucosinolate biosynthesis	map00966	1	ec:2.6.1.42 - transaminase	6	vo_c2_Locus_384_Transcript_2/3_Confidence_0.400_Length_1725, tri_c_comp32679_c0_seq1, vo_c1_Locus_10788_Transcript_1/1_Confidence_0.000_Length_1721, vo_c2_Locus_14969_Transcript_4/8_Confidence_0.750_Length_1231, vo_n2_Locus_3786_Transcript_2/4_Confidence_0.778_Length_1466, vo_n2_Locus_3786_Transcript_3/4_Confidence_0.667_Length_1442

Cutin, suberine and wax biosynthesis	map00073	1	ec:2.3.1.20 - O-acyltransferase	8	vo_c2_Locus_1239_Transcript_4/4_Confidence_0.000_Length_1343, vo_n1_Locus_12743_Transcript_2/2_Confidence_0.000_Length_633, tri_c_comp9226_c0_seq1, tri_n_comp38992_c0_seq1, vo_n3_Locus_16690_Transcript_3/4_Confidence_0.667_Length_408, vo_n2_Locus_19192_Transcript_2/2_Confidence_0.250_Length_3053, vo_c2_Locus_16114_Transcript_1/1_Confidence_0.000_Length_1890, vo_n1_Locus_1454_Transcript_3/3_Confidence_0.000_Length_612

Biosynthesis of ansamycins	map01051	1	ec:2.2.1.1 - glycolaldehydetransferase	24	vo_c3_Locus_1314_Transcript_17/46_Confidence_0.365_Length_2530, vo_n3_Locus_387_Transcript_34/39_Confidence_0.386_Length_2514, vo_c2_Locus_924_Transcript_20/40_Confidence_0.452_Length_2520, vo_c2_Locus_924_Transcript_13/40_Confidence_0.310_Length_2559, vo_c3_Locus_1314_Transcript_33/46_Confidence_0.292_Length_2524, vo_c1_Locus_1204_Transcript_14/17_Confidence_0.394_Length_4476, vo_c2_Locus_924_Transcript_19/40_Confidence_0.333_Length_2562, vo_n1_Locus_327_Transcript_13/41_Confidence_0.275_Length_2480, vo_c3_Locus_1314_Transcript_6/46_Confidence_0.208_Length_1518, vo_n1_Locus_327_Transcript_40/41_Confidence_0.176_Length_2442, vo_c3_Locus_1314_Transcript_38/46_Confidence_0.302_Length_2522, vo_c2_Locus_924_Transcript_34/40_Confidence_0.369_Length_2618, vo_c3_Locus_1314_Transcript_14/46_Confidence_0.229_Length_3828, vo_c2_Locus_924_Transcript_16/40_Confidence_0.321_Length_2559, vo_c2_Locus_924_Transcript_23/40_Confidence_0.488_Length_2850, vo_c3_Locus_1314_Transcript_21/46_Confidence_0.365_Length_2524, vo_c1_Locus_1204_Transcript_13/17_Confidence_0.364_Length_4497, vo_n1_Locus_327_Transcript_24/41_Confidence_0.385_Length_2547, vo_c2_Locus_924_Transcript_17/40_Confidence_0.333_Length_2453, tri_c_comp20608_c0_seq4, tri_c_comp20608_c0_seq5, vo_c3_Locus_1314_Transcript_43/46_Confidence_0.198_Length_2519, vo_n2_Locus_2054_Transcript_26/37_Confidence_0.448_Length_2522, tri_c_comp20608_c0_seq7

Carbapenem biosynthesis	map00332	1	ec:2.7.2.11 - 5-kinase	1	vo_c2_Locus_12865_Transcript_1/3_Confidence_0.667_Length_1839

Supplemental Table S8. BUSCO analysis results. Lineage dataset eukariota_odb9 was used. Out of 303 BUSCOs 68.4% complete entries were identified (44.6% single and 23.8% duplicated) and 4.0% were detected as fragments.

# Busco id	Status	Sequence	Score	Length
EOG0937004M	Complete	tri_n_comp28287_c0_seq1	3561.9	1998
EOG09370082	Complete	vo_n2_Locus_1536_Transcript_2_3_Confidence_0.400_Length_9999	1442.8	1309
EOG093700N7	Missing			
EOG0937017X	Duplicated	vo_c2_Locus_12179_Transcript_1_3_Confidence_0.750_Length_3821	731.4	1141
EOG0937017X	Duplicated	vo_n1_Locus_32615_Transcript_1_1_Confidence_0.000_Length_3822	672.9	1130
EOG0937017X	Duplicated	vo_n1_Locus_6757_Transcript_2_2_Confidence_0.000_Length_3945	722.2	1152
EOG0937018Z	Complete	vo_c3_Locus_15719_Transcript_1_2_Confidence_0.333_Length_3591	876.6	886
EOG0937019B	Duplicated	tri_c_comp19738_c0_seq1	1395.3	834
EOG0937019B	Duplicated	vo_c2_Locus_12608_Transcript_10_13_Confidence_0.472_Length_3456	1395.2	830
EOG0937019B	Duplicated	vo_c3_Locus_22919_Transcript_2_3_Confidence_0.471_Length_3607	1388.3	834
EOG093701EE	Duplicated	tri_c_comp51602_c0_seq1	723.9	788
EOG093701EE	Duplicated	vo_n2_Locus_13218_Transcript_3_4_Confidence_0.375_Length_5213	672.1	800
EOG093701IQ	Complete	tri_c_comp17782_c0_seq3	512.8	380
EOG093701S0	Complete	vo_n2_Locus_3662_Transcript_1_1_Confidence_0.667_Length_3532	1033.7	692
EOG093701SQ	Complete	vo_n2_Locus_4121_Transcript_4_4_Confidence_0.167_Length_4300	1212.2	877
EOG093701V7	Complete	vo_n2_Locus_8676_Transcript_1_2_Confidence_0.750_Length_3354	885.7	688
EOG0937029K	Complete	vo_c3_Locus_19859_Transcript_1_1_Confidence_0.000_Length_3251	215.3	425
EOG093702AT	Complete	tri_n_comp34787_c0_seq1	475.8	477
EOG093702DP	Duplicated	vo_c2_Locus_1147_Transcript_1_2_Confidence_0.750_Length_1828	163.4	361
EOG093702DP	Duplicated	vo_c3_Locus_11479_Transcript_1_2_Confidence_1.000_Length_1915	181.7	361
EOG093702MJ	Duplicated	vo_c2_Locus_1219_Transcript_31_32_Confidence_0.123_Length_3127	114.8	433
EOG093702MJ	Duplicated	vo_n2_Locus_553_Transcript_3_18_Confidence_0.077_Length_3029	114.9	433
EOG093702PE	Complete	vo_n1_Locus_3390_Transcript_2_3_Confidence_0.400_Length_4401	175.0	407
EOG0937033T	Complete	tri_c_comp47897_c0_seq1	307.7	461
EOG0937036L	Complete	tri_c_comp27527_c0_seq1	365.7	974
EOG093703C8	Missing			
EOG093703DY	Complete	vo_c1_Locus_26649_Transcript_1_2_Confidence_0.333_Length_2985	159.2	391

EOG093703N5	Complete	tri_n_comp57560_c0_seq1	187.5	248
EOG093703UX	Missing			
EOG093703W1	Missing			
EOG093703WD	Complete	tri_n_comp40166_c0_seq1	469.4	553
EOG093703YA	Duplicated	vo_n2_Locus_14475_Transcript_1_5_Confidence_0.700_Length_2590	632.1	519
EOG093703YA	Duplicated	vo_n2_Locus_4619_Transcript_1_1_Confidence_0.000_Length_2377	685.6	603
EOG0937049Y	Missing			
EOG093704GD	Complete	vo_c2_Locus_17606_Transcript_1_1_Confidence_0.000_Length_2178	298.8	455
EOG093704HC	Duplicated	vo_c2_Locus_344_Transcript_2_3_Confidence_0.333_Length_2699	672.9	396
EOG093704HC	Duplicated	vo_n2_Locus_4514_Transcript_3_3_Confidence_0.167_Length_5219	670.8	396
EOG093704J7	Missing			
EOG093704Q0	Complete	tri_c_comp34928_c0_seq1	406.6	430
EOG093704UY	Missing			
EOG0937050C	Complete	vo_c2_Locus_21699_Transcript_1_2_Confidence_1.000_Length_3034	409.6	649
EOG093705DM	Complete	vo_n1_Locus_10149_Transcript_1_1_Confidence_0.000_Length_1299	317.6	293
EOG093705E5	Complete	vo_c1_Locus_3242_Transcript_1_1_Confidence_0.000_Length_2341	255.5	439
EOG093705EY	Complete	vo_n2_Locus_14099_Transcript_1_1_Confidence_0.000_Length_2060	521.6	450
EOG093705KN	Complete	vo_c3_Locus_2223_Transcript_7_8_Confidence_0.231_Length_3519	371.4	386
EOG093705U4	Missing			
EOG093705VV	Complete	tri_c_comp43285_c0_seq1	536.0	433
EOG093705YA	Duplicated	vo_c3_Locus_19664_Transcript_2_6_Confidence_0.583_Length_2143	564.9	466
EOG093705YA	Duplicated	vo_c3_Locus_8275_Transcript_2_3_Confidence_0.750_Length_2028	659.6	536
EOG093705YE	Duplicated	vo_c2_Locus_14_Transcript_9_23_Confidence_0.077_Length_3444	62.6	228
EOG093705YE	Duplicated	vo_n1_Locus_949_Transcript_2_4_Confidence_0.286_Length_2270	55.2	286
EOG093705Z6	Missing			
EOG0937060I	Duplicated	vo_c2_Locus_18064_Transcript_1_1_Confidence_0.333_Length_2385	514.1	617
EOG0937060I	Duplicated	vo_n2_Locus_7639_Transcript_1_1_Confidence_0.000_Length_3449	582.6	618
EOG0937060I	Duplicated	vo_n3_Locus_16518_Transcript_1_1_Confidence_0.000_Length_2956	548.7	676
EOG0937066G	Duplicated	tri_n_comp27405_c0_seq1	530.7	411
EOG0937066G	Duplicated	vo_c1_Locus_5594_Transcript_1_2_Confidence_0.500_Length_3744	526.8	411
EOG0937066G	Duplicated	vo_c1_Locus_5594_Transcript_1_2_Confidence_0.500_Length_3744	527.0	411
EOG0937066G	Duplicated	vo_c2_Locus_17209_Transcript_1_2_Confidence_0.750_Length_2003	532.1	431

EOG0937068H	Duplicated	tri_c_comp25244_c0_seq1	257.3	404
EOG0937068H	Duplicated	tri_c_comp33097_c0_seq1	231.7	405
EOG093706PM	Complete	vo_c1_Locus_13782_Transcript_1_2_Confidence_0.333_Length_2472	333.0	298
EOG093706RN	Complete	vo_n3_Locus_14997_Transcript_1_1_Confidence_0.000_Length_2574	217.7	416
EOG093706X0	Duplicated	vo_c3_Locus_7772_Transcript_3_6_Confidence_0.600_Length_1899	122.6	295
EOG093706X0	Duplicated	vo_c3_Locus_7772_Transcript_4_6_Confidence_0.500_Length_1937	122.5	295
EOG093706X0	Duplicated	vo_n2_Locus_4706_Transcript_4_7_Confidence_0.308_Length_2684	136.4	354
EOG09370762	Complete	vo_c3_Locus_107_Transcript_2_2_Confidence_0.000_Length_1878	698.5	486
EOG093707B7	Complete	tri_n_comp29647_c0_seq1	182.0	418
EOG093707HK	Complete	vo_n2_Locus_2003_Transcript_2_2_Confidence_0.250_Length_2177	301.1	483
EOG093707LO	Complete	tri_c_comp17539_c0_seq5	225.8	400
EOG093707RF	Complete	vo_c1_Locus_21157_Transcript_2_3_Confidence_0.600_Length_3136	280.9	439
EOG093707VN	Complete	vo_c3_Locus_9511_Transcript_1_1_Confidence_0.000_Length_2619	348.5	407
EOG093707VQ	Complete	tri_n_comp31257_c0_seq1	281.6	537
EOG0937084T	Complete	vo_c2_Locus_3047_Transcript_2_4_Confidence_0.286_Length_3515	164.6	351
EOG0937085E	Complete	vo_n1_Locus_24686_Transcript_1_1_Confidence_0.000_Length_1774	399.6	412
EOG093708IM	Complete	vo_c1_Locus_1424_Transcript_1_1_Confidence_0.000_Length_2064	372.3	387
EOG093708OP	Missing			
EOG093708TP	Duplicated	vo_n1_Locus_6239_Transcript_19_19_Confidence_0.150_Length_1856	386.1	376
EOG093708TP	Duplicated	vo_n1_Locus_6239_Transcript_3_19_Confidence_0.425_Length_1997	387.3	378
EOG093708TP	Duplicated	vo_n3_Locus_2366_Transcript_13_24_Confidence_0.236_Length_1948	387.9	380
EOG0937091Y	Complete	vo_c1_Locus_1184_Transcript_9_10_Confidence_0.357_Length_2595	580.4	518
EOG0937092T	Missing			
EOG0937092W	Complete	vo_c2_Locus_3336_Transcript_1_1_Confidence_0.333_Length_2657	288.6	463
EOG093709BZ	Duplicated	tri_c_comp30439_c0_seq1	126.8	400
EOG093709BZ	Duplicated	vo_c1_Locus_27004_Transcript_1_1_Confidence_0.000_Length_2081	128.7	381
EOG093709HP	Complete	tri_c_comp52375_c0_seq1	144.7	331
EOG093709JG	Complete	vo_n2_Locus_28120_Transcript_1_1_Confidence_0.000_Length_2388	80.7	256
EOG09370A22	Duplicated	vo_c3_Locus_15749_Transcript_2_2_Confidence_0.000_Length_1608	451.2	350
EOG09370A22	Duplicated	vo_n1_Locus_27865_Transcript_1_1_Confidence_0.000_Length_1578	451.8	350

EOG09370ADO	Duplicated	vo_c2_Locus_1983_Transcript_1_4_Confidence_0.714_Length_1572	579.3	381
EOG09370ADO	Duplicated	vo_n3_Locus_2382_Transcript_1_2_Confidence_1.000_Length_1689	671.8	422
EOG09370AI8	Complete	vo_c3_Locus_12888_Transcript_1_1_Confidence_0.000_Length_1609	250.3	246
EOG09370AJP	Complete	vo_n1_Locus_6460_Transcript_1_1_Confidence_0.000_Length_1912	540.7	398
EOG09370AL3	Duplicated	vo_c1_Locus_25304_Transcript_1_1_Confidence_0.000_Length_1848	332.1	366
EOG09370AL3	Duplicated	vo_n2_Locus_4701_Transcript_2_2_Confidence_0.000_Length_1858	308.1	366
EOG09370ARO	Missing			
EOG09370AS9	Complete	vo_c1_Locus_1681_Transcript_2_6_Confidence_0.500_Length_2477	167.9	294
EOG09370AV1	Complete	vo_n1_Locus_964_Transcript_1_1_Confidence_0.000_Length_1596	134.4	296
EOG09370AWB	Missing			
EOG09370B1L	Complete	vo_n3_Locus_15336_Transcript_1_1_Confidence_0.000_Length_1810	402.0	408
EOG09370B65	Missing			
EOG09370B7D	Duplicated	vo_c2_Locus_18469_Transcript_7_11_Confidence_0.429_Length_1816	127.8	146
EOG09370B7D	Duplicated	vo_c2_Locus_18469_Transcript_9_11_Confidence_0.214_Length_1549	141.0	165
EOG09370BBR	Complete	tri_c_comp27551_c0_seq1	550.1	340
EOG09370BGO	Duplicated	vo_c1_Locus_10425_Transcript_2_3_Confidence_0.667_Length_2502	48.3	191
EOG09370BGO	Duplicated	vo_c2_Locus_1193_Transcript_3_9_Confidence_0.588_Length_5761	44.8	191
EOG09370BGO	Duplicated	vo_c2_Locus_1193_Transcript_4_9_Confidence_0.588_Length_5737	44.8	191
EOG09370BJE	Duplicated	tri_n_comp27390_c0_seq1	343.5	296
EOG09370BJE	Duplicated	vo_n1_Locus_11298_Transcript_1_3_Confidence_0.833_Length_2343	343.6	296
EOG09370BJE	Duplicated	vo_n3_Locus_1440_Transcript_3_5_Confidence_0.231_Length_3420	345.5	255
EOG09370BK5	Missing			
EOG09370BQO	Missing			
EOG09370BYX	Duplicated	tri_n_comp12418_c0_seq1	25.6	334
EOG09370BYX	Duplicated	tri_n_comp12418_c0_seq2	25.3	333
EOG09370C5A	Complete	vo_n2_Locus_12673_Transcript_1_1_Confidence_0.000_Length_1679	140.2	433
EOG09370CAN	Missing			
EOG09370CAV	Complete	vo_c2_Locus_15048_Transcript_2_3_Confidence_0.333_Length_3467	337.9	511
EOG09370CF1	Missing			
EOG09370CIV	Duplicated	tri_n_comp9789_c0_seq1	145.8	241
EOG09370CIV	Duplicated	tri_n_comp9789_c0_seq2	145.9	241
EOG09370CIV	Duplicated	vo_n2_Locus_1288_Transcript_4_8_Confidence_0.265_Length_1902	146.1	241
EOG09370CK6	Missing			

EOG09370CKL	Missing			
EOG09370CRF	Complete	vo_n2_Locus_17117_Transcript_1_1_Confidence_0.000_Length_1585	322.5	352
EOG09370CTU	Missing			
EOG09370CZ0	Complete	vo_c2_Locus_25768_Transcript_1_1_Confidence_0.000_Length_1551	200.6	214
EOG09370CZT	Complete	vo_c3_Locus_18882_Transcript_1_1_Confidence_0.000_Length_3621	119.3	205
EOG09370D13	Duplicated	vo_n2_Locus_7930_Transcript_11_23_Confidence_0.455_Length_3963	208.0	377
EOG09370D13	Duplicated	vo_n2_Locus_7930_Transcript_12_23_Confidence_0.364_Length_3996	208.0	377
EOG09370D13	Duplicated	vo_n2_Locus_7930_Transcript_9_23_Confidence_0.485_Length_4050	207.9	377
EOG09370DO5	Complete	vo_c1_Locus_5647_Transcript_1_3_Confidence_0.600_Length_3208	150.5	284
EOG09370DP4	Fragmented	vo_n1_Locus_7772_Transcript_1_1_Confidence_0.000_Length_6986	23.7	98
EOG09370DS4	Missing			
EOG09370DTG	Fragmented	vo_c1_Locus_24563_Transcript_5_7_Confidence_0.127_Length_2200	74.7	207
EOG09370DV4	Duplicated	vo_n2_Locus_6078_Transcript_6_7_Confidence_0.600_Length_2112	345.3	324
EOG09370DV4	Duplicated	vo_n2_Locus_6078_Transcript_7_7_Confidence_0.300_Length_2132	345.3	324
EOG09370DX4	Missing			
EOG09370DXT	Missing			
EOG09370DYK	Duplicated	tri_n_comp6202_c0_seq1	152.0	209
EOG09370DYK	Duplicated	tri_n_comp6202_c0_seq2	137.2	209
EOG09370E0Y	Duplicated	vo_n1_Locus_3026_Transcript_2_3_Confidence_0.400_Length_2292	424.0	339
EOG09370E0Y	Duplicated	vo_n2_Locus_13581_Transcript_1_1_Confidence_0.000_Length_1796	425.5	339
EOG09370E36	Missing			
EOG09370E4U	Complete	vo_n1_Locus_690_Transcript_69_127_Confidence_0.011_Length_1601	302.3	239
EOG09370E4W	Complete	tri_n_comp23997_c0_seq1	259.7	281
EOG09370E8S	Complete	tri_n_comp31892_c0_seq1	211.2	267
EOG09370EHZ	Duplicated	vo_n1_Locus_24951_Transcript_1_2_Confidence_0.750_Length_1980	349.0	395
EOG09370EHZ	Duplicated	vo_n1_Locus_24951_Transcript_2_2_Confidence_0.000_Length_2025	348.9	395
EOG09370EL3	Missing			
EOG09370ER5	Complete	vo_n2_Locus_35831_Transcript_1_1_Confidence_0.000_Length_2496	237.2	323
EOG09370F1Q	Complete	vo_n1_Locus_18911_Transcript_2_2_Confidence_0.333_Length_1516	70.1	123
EOG09370F3T	Missing			
EOG09370F47	Complete	vo_n3_Locus_26739_Transcript_1_1_Confidence_0.000_Length_1382	413.4	265
EOG09370FKI	Missing			
EOG09370FLD	Missing			

EOG09370FN1	Duplicated	vo_c1_Locus_799_Transcript_1_12_Confidence_0.423_Length_1663	390.7	312
EOG09370FN1	Duplicated	vo_c3_Locus_3850_Transcript_7_16_Confidence_0.250_Length_1369	390.8	279
EOG09370FN1	Duplicated	vo_n1_Locus_1510_Transcript_4_24_Confidence_0.208_Length_1370	392.5	320
EOG09370FSS	Complete	vo_c3_Locus_26616_Transcript_1_1_Confidence_0.000_Length_1783	122.6	247
EOG09370FVX	Complete	tri_n_comp38078_c0_seq1	238.7	298
EOG09370FWQ	Duplicated	tri_n_comp41542_c0_seq1	373.4	311
EOG09370FWQ	Duplicated	vo_c2_Locus_1588_Transcript_1_1_Confidence_0.000_Length_1316	375.5	311
EOG09370FXE	Missing			
EOG09370GC2	Complete	vo_n1_Locus_23038_Transcript_2_3_Confidence_0.400_Length_2942	78.6	152
EOG09370GEO	Complete	vo_c3_Locus_20979_Transcript_2_3_Confidence_0.400_Length_1639	79.0	158
EOG09370GHM	Duplicated	vo_c1_Locus_1120_Transcript_3_11_Confidence_0.500_Length_3021	164.8	201
EOG09370GHM	Duplicated	vo_c1_Locus_1120_Transcript_6_11_Confidence_0.333_Length_2994	164.8	201
EOG09370GHM	Duplicated	vo_c1_Locus_3540_Transcript_1_1_Confidence_0.000_Length_2284	186.7	223
EOG09370GHP	Missing			
EOG09370GIK	Duplicated	vo_c1_Locus_20849_Transcript_1_1_Confidence_0.000_Length_1120	303.4	215
EOG09370GIK	Duplicated	vo_c2_Locus_30999_Transcript_2_2_Confidence_0.000_Length_1165	283.8	218
EOG09370GJ7	Complete	tri_n_comp29754_c0_seq1	328.0	342
EOG09370GK0	Missing			
EOG09370H3E	Complete	vo_c2_Locus_703_Transcript_1_2_Confidence_0.333_Length_2334	306.4	461
EOG09370H68	Complete	tri_n_comp4523_c0_seq1	68.0	215
EOG09370HBN	Fragmented	vo_c2_Locus_16306_Transcript_1_1_Confidence_0.000_Length_1112	109.7	153
EOG09370HD7	Missing			
EOG09370HKV	Duplicated	vo_c2_Locus_19754_Transcript_2_3_Confidence_0.500_Length_1997	141.1	318
EOG09370HKV	Duplicated	vo_n2_Locus_4167_Transcript_1_6_Confidence_0.667_Length_2035	150.6	328
EOG09370HVI	Complete	vo_n2_Locus_7479_Transcript_1_1_Confidence_0.000_Length_1358	115.3	190
EOG09370I3J	Duplicated	tri_c_comp16401_c0_seq2	191.8	213
EOG09370I3J	Duplicated	tri_c_comp16401_c0_seq4	190.0	208
EOG09370I3J	Duplicated	tri_c_comp16401_c0_seq5	191.6	213
EOG09370I7R	Missing			
EOG09370IF5	Complete	tri_c_comp25586_c0_seq1	277.7	274
EOG09370IH5	Fragmented	vo_c3_Locus_7546_Transcript_3_14_Confidence_0.529_Length_1389	35.5	103
EOG09370IMU	Complete	vo_c2_Locus_10633_Transcript_7_19_Confidence_0.448_Length_1346	59.8	164
EOG09370INZ	Complete	tri_c_comp10311_c0_seq1	198.5	280

EOG09370IUA	Complete	vo_c3_Locus_1134_Transcript_1_1_Confidence_0.000_Length_1398	300.9	205
EOG09370J9L	Complete	vo_n2_Locus_27499_Transcript_1_1_Confidence_0.000_Length_4654	107.0	182
EOG09370JIK	Missing			
EOG09370JW6	Missing			
EOG09370JZ3	Missing			
EOG09370K15	Missing			
EOG09370K1R	Complete	vo_c3_Locus_1766_Transcript_1_1_Confidence_0.000_Length_1152	146.1	205
EOG09370KD4	Complete	vo_n1_Locus_20507_Transcript_1_2_Confidence_0.667_Length_1235	359.2	236
EOG09370KGV	Complete	tri_n_comp28286_c0_seq1	113.5	169
EOG09370KJ3	Complete	vo_c3_Locus_15650_Transcript_1_1_Confidence_0.000_Length_1033	180.6	168
EOG09370KM9	Complete	vo_n3_Locus_17035_Transcript_1_1_Confidence_0.000_Length_2586	117.3	232
EOG09370KOV	Fragmented	vo_n1_Locus_26419_Transcript_1_1_Confidence_0.000_Length_1111	90.8	118
EOG09370KWF	Missing			
EOG09370KYZ	Missing			
EOG09370L23	Duplicated	vo_c3_Locus_18494_Transcript_5_9_Confidence_0.667_Length_1652	272.0	213
EOG09370L23	Duplicated	vo_n1_Locus_21388_Transcript_1_4_Confidence_0.667_Length_1107	281.3	218
EOG09370L23	Duplicated	vo_n1_Locus_21388_Transcript_2_4_Confidence_0.500_Length_1146	264.1	218
EOG09370L77	Missing			
EOG09370LFI	Duplicated	vo_c1_Locus_9447_Transcript_1_1_Confidence_0.000_Length_1221	304.2	205
EOG09370LFI	Duplicated	vo_n3_Locus_17513_Transcript_2_2_Confidence_0.000_Length_884	295.9	200
EOG09370LGH	Complete	tri_n_comp77429_c0_seq1	247.9	280
EOG09370LMP	Complete	vo_n1_Locus_30418_Transcript_3_3_Confidence_0.000_Length_1559	339.9	305
EOG09370LQ2	Duplicated	vo_c1_Locus_25845_Transcript_1_1_Confidence_0.000_Length_1224	90.7	178
EOG09370LQ2	Duplicated	vo_n2_Locus_22805_Transcript_5_6_Confidence_0.273_Length_3128	85.3	178
EOG09370LYY	Complete	vo_n2_Locus_305_Transcript_1_3_Confidence_0.714_Length_1537	128.8	430
EOG09370M9E	Complete	vo_c2_Locus_11139_Transcript_1_1_Confidence_0.000_Length_1057	128.1	202
EOG09370MAO	Complete	vo_c2_Locus_1532_Transcript_2_2_Confidence_0.000_Length_1203	244.5	232
EOG09370MDU	Duplicated	vo_c3_Locus_6904_Transcript_2_2_Confidence_0.250_Length_1012	213.0	179
EOG09370MDU	Duplicated	vo_n2_Locus_2814_Transcript_6_6_Confidence_0.444_Length_1020	212.9	179
EOG09370MGF	Complete	tri_c_comp32838_c0_seq1	139.7	181
EOG09370MHI	Fragmented	vo_c3_Locus_2992_Transcript_5_11_Confidence_0.213_Length_1574	169.4	169

EOG09370MMK	Missing			
EOG09370MQ0	Complete	vo_n2_Locus_16404_Transcript_1_2_Confidence_1.000_Length_1226	231.6	213
EOG09370MZ4	Complete	vo_n3_Locus_9972_Transcript_1_1_Confidence_0.000_Length_991	372.4	226
EOG09370N7W	Complete	vo_c2_Locus_30736_Transcript_1_1_Confidence_0.000_Length_1713	31.0	165
EOG09370NBT	Complete	vo_n1_Locus_17235_Transcript_1_2_Confidence_0.333_Length_1228	215.2	242
EOG09370NBW	Missing			
EOG09370NHX	Complete	vo_c2_Locus_23277_Transcript_1_1_Confidence_0.000_Length_1058	108.9	134
EOG09370NIG	Complete	tri_c_comp52297_c0_seq1	132.5	134
EOG09370O06	Complete	vo_n2_Locus_3339_Transcript_1_1_Confidence_0.000_Length_1083	127.3	195
EOG09370O51	Complete	vo_c1_Locus_10206_Transcript_1_2_Confidence_0.667_Length_876	234.3	196
EOG09370OFR	Complete	tri_n_comp56132_c0_seq1	67.5	179
EOG09370OGU	Complete	vo_n2_Locus_6796_Transcript_1_1_Confidence_0.000_Length_1236	100.9	146
EOG09370OGW	Missing			
EOG09370OHP	Complete	vo_c3_Locus_32304_Transcript_1_1_Confidence_0.000_Length_1078	199.3	204
EOG09370OLY	Complete	vo_n2_Locus_12823_Transcript_1_1_Confidence_0.000_Length_1341	172.5	162
EOG09370OO3	Missing			
EOG09370OS5	Missing			
EOG09370OUS	Missing			
EOG09370P2W	Complete	tri_c_comp38699_c0_seq1	77.4	97
EOG09370P5Q	Complete	tri_n_comp62251_c0_seq1	133.9	199
EOG09370P7I	Complete	vo_c3_Locus_21398_Transcript_1_1_Confidence_0.000_Length_891	79.5	159
EOG09370P9T	Missing			
EOG09370PBD	Duplicated	vo_c3_Locus_4396_Transcript_5_18_Confidence_0.345_Length_977	334.1	221
EOG09370PBD	Duplicated	vo_n1_Locus_1534_Transcript_14_27_Confidence_0.224_Length_935	334.7	221
EOG09370PBD	Duplicated	vo_n1_Locus_1534_Transcript_16_27_Confidence_0.241_Length_938	335.5	221
EOG09370PLC	Complete	tri_c_comp49730_c0_seq1	146.7	222
EOG09370PN3	Missing			
EOG09370PYJ	Complete	tri_c_comp29883_c0_seq1	289.1	217
EOG09370QBK	Complete	vo_n1_Locus_22998_Transcript_13_14_Confidence_0.533_Length_983	41.3	128
EOG09370QBR	Missing			
EOG09370QCX	Complete	tri_c_comp26027_c0_seq1	86.4	122
EOG09370QFD	Fragmented	tri_n_comp6551_c0_seq1	40.7	113
EOG09370QI4	Complete	vo_c1_Locus_8464_Transcript_1_1_Confidence_0.000_Length_1137	124.7	189
EOG09370QJ1	Missing			
EOG09370QKS	Missing			

EOG09370QMM	Missing			
EOG09370QT3	Missing			
EOG09370QVI	Missing			
EOG09370R3M	Complete	tri_n_comp22296_c0_seq1	258.2	183
EOG09370R67	Duplicated	vo_c2_Locus_31589_Transcript_2_2_Confidence_0.000_Length_1480	120.6	136
EOG09370R67	Duplicated	vo_n2_Locus_21119_Transcript_1_4_Confidence_0.800_Length_1427	120.9	136
EOG09370RCD	Missing			
EOG09370RCQ	Complete	vo_n2_Locus_21967_Transcript_1_1_Confidence_0.000_Length_1233	174.3	181
EOG09370RCS	Complete	vo_c3_Locus_3261_Transcript_1_3_Confidence_0.667_Length_3934	174.6	172
EOG09370RIT	Missing			
EOG09370RJN	Fragmented	vo_c1_Locus_2791_Transcript_1_1_Confidence_0.000_Length_1592	48.6	96
EOG09370RRJ	Missing			
EOG09370S0F	Complete	vo_c2_Locus_25315_Transcript_1_3_Confidence_0.400_Length_1033	79.9	114
EOG09370S1Z	Missing			
EOG09370S3E	Missing			
EOG09370S4T	Missing			
EOG09370SC9	Fragmented	vo_c1_Locus_12548_Transcript_1_3_Confidence_0.571_Length_692	54.8	89
EOG09370SO4	Missing			
EOG09370SON	Complete	vo_n1_Locus_33361_Transcript_1_3_Confidence_0.400_Length_1225	88.8	137
EOG09370SPQ	Missing			
EOG09370SV2	Duplicated	vo_c1_Locus_9056_Transcript_2_2_Confidence_0.250_Length_907	91.5	127
EOG09370SV2	Duplicated	vo_c2_Locus_2560_Transcript_3_3_Confidence_0.143_Length_850	80.8	114
EOG09370SYK	Complete	vo_c2_Locus_6435_Transcript_5_6_Confidence_0.032_Length_7385	345.7	434
EOG09370T6W	Complete	vo_c2_Locus_313_Transcript_5_7_Confidence_0.250_Length_1589	162.0	166
EOG09370T9R	Complete	vo_c1_Locus_5185_Transcript_6_21_Confidence_0.118_Length_1734	204.8	156
EOG09370TBD	Duplicated	vo_n2_Locus_14153_Transcript_1_4_Confidence_0.833_Length_970	218.1	135
EOG09370TBD	Duplicated	vo_n2_Locus_14153_Transcript_2_4_Confidence_0.833_Length_1009	218.0	135
EOG09370TBG	Duplicated	tri_c_comp29751_c0_seq1	66.3	139
EOG09370TBG	Duplicated	vo_c1_Locus_22175_Transcript_1_1_Confidence_0.000_Length_1063	72.4	119
EOG09370TCX	Duplicated	vo_c2_Locus_20815_Transcript_5_6_Confidence_0.263_Length_1314	48.2	170
EOG09370TCX	Duplicated	vo_c2_Locus_20815_Transcript_6_6_Confidence_0.158_Length_1305	49.7	164
EOG09370TCX	Duplicated	vo_c3_Locus_2547_Transcript_8_8_Confidence_0.130_Length_1319	51.6	170

EOG09370TFO	Complete	vo_n1_Locus_19361_Transcript_1_2_Confidence_0.333_Length_1454	116.2	278
EOG09370TG2	Duplicated	vo_c1_Locus_33182_Transcript_2_3_Confidence_0.250_Length_838	163.8	149
EOG09370TG2	Duplicated	vo_n2_Locus_28077_Transcript_1_2_Confidence_0.333_Length_835	163.8	149
EOG09370TG6	Complete	vo_n2_Locus_5069_Transcript_1_1_Confidence_0.000_Length_852	152.4	130
EOG09370TH4	Duplicated	tri_n_comp29271_c0_seq1	129.2	117
EOG09370TH4	Duplicated	vo_n1_Locus_18221_Transcript_2_3_Confidence_0.333_Length_2330	127.1	117
EOG09370TH4	Duplicated	vo_n2_Locus_7391_Transcript_1_1_Confidence_0.000_Length_1253	129.4	117
EOG09370TP2	Complete	vo_n2_Locus_10965_Transcript_1_1_Confidence_0.000_Length_1384	134.8	211
EOG09370TS7	Duplicated	tri_c_comp13669_c0_seq1	53.6	130
EOG09370TS7	Duplicated	tri_c_comp13669_c0_seq2	54.1	130
EOG09370TS7	Duplicated	vo_n3_Locus_14626_Transcript_1_1_Confidence_0.000_Length_1061	53.7	130
EOG09370TTI	Missing			
EOG09370U0A	Duplicated	tri_c_comp25165_c0_seq1	221.2	214
EOG09370U0A	Duplicated	vo_c1_Locus_7619_Transcript_3_5_Confidence_0.222_Length_2303	218.5	216
EOG09370U0A	Duplicated	vo_n3_Locus_3123_Transcript_2_16_Confidence_0.046_Length_1409	221.4	214
EOG09370U71	Duplicated	vo_c1_Locus_18378_Transcript_4_13_Confidence_0.556_Length_1230	132.8	195
EOG09370U71	Duplicated	vo_n2_Locus_7202_Transcript_2_10_Confidence_0.524_Length_1121	116.6	205
EOG09370U9P	Complete	vo_n2_Locus_8097_Transcript_1_6_Confidence_0.500_Length_1196	133.8	170
EOG09370UEC	Missing			
EOG09370UG9	Duplicated	vo_c2_Locus_17369_Transcript_1_1_Confidence_0.000_Length_872	138.4	155
EOG09370UG9	Duplicated	vo_n2_Locus_7335_Transcript_1_3_Confidence_0.625_Length_757	130.0	139
EOG09370UID	Duplicated	vo_c1_Locus_5485_Transcript_1_3_Confidence_0.714_Length_983	94.8	116
EOG09370UID	Duplicated	vo_c3_Locus_19272_Transcript_2_5_Confidence_0.667_Length_1078	94.6	116
EOG09370UID	Duplicated	vo_n3_Locus_4239_Transcript_1_2_Confidence_0.667_Length_790	86.6	116
EOG09370UO4	Duplicated	vo_c2_Locus_1995_Transcript_5_8_Confidence_0.283_Length_1191	196.7	166
EOG09370UO4	Duplicated	vo_c3_Locus_24967_Transcript_5_7_Confidence_0.278_Length_1263	196.4	166
EOG09370UO4	Duplicated	vo_n2_Locus_3_Transcript_21809_24291_Confidence_0.000_Length_1296	196.3	166
EOG09370UR1	Complete	vo_c3_Locus_24633_Transcript_1_1_Confidence_0.000_Length_761	94.9	136
EOG09370UR3	Missing			
EOG09370USH	Complete	tri_c_comp25256_c0_seq1	139.3	141

EOG09370USJ	Fragmented	vo_c2_Locus_20278_Transcript_2_2_Confidence_0.000_Length_1134	160.3	101
EOG09370UV4	Fragmented	vo_n1_Locus_24077_Transcript_2_2_Confidence_0.000_Length_636	104.2	145
EOG09370V9R	Complete	vo_c2_Locus_20718_Transcript_1_5_Confidence_0.333_Length_1663	53.3	113
EOG09370VFL	Duplicated	vo_c1_Locus_27431_Transcript_1_1_Confidence_0.333_Length_1120	128.3	241
EOG09370VFL	Duplicated	vo_n1_Locus_13323_Transcript_2_2_Confidence_0.400_Length_1076	134.0	242
EOG09370VLM	Complete	vo_c2_Locus_6942_Transcript_1_2_Confidence_1.000_Length_758	55.0	115
EOG09370VTP	Complete	vo_c1_Locus_28178_Transcript_1_1_Confidence_0.000_Length_1349	285.9	353
EOG09370VUX	Missing			
EOG09370W44	Complete	vo_n1_Locus_30711_Transcript_1_1_Confidence_0.000_Length_904	133.4	158
EOG09370W6N	Missing			
EOG09370W86	Duplicated	vo_n3_Locus_25439_Transcript_2_4_Confidence_0.625_Length_948	166.3	162
EOG09370W86	Duplicated	vo_n3_Locus_25439_Transcript_4_4_Confidence_0.125_Length_991	168.1	150
EOG09370WT1	Duplicated	vo_c1_Locus_10096_Transcript_1_3_Confidence_0.200_Length_717	151.6	122
EOG09370WT1	Duplicated	vo_c2_Locus_8914_Transcript_3_3_Confidence_0.167_Length_1003	151.0	122
EOG09370WT1	Duplicated	vo_n2_Locus_5522_Transcript_1_3_Confidence_0.750_Length_958	151.0	122
EOG09370WWI	Missing			
EOG09370WYG	Duplicated	vo_c3_Locus_2501_Transcript_10_10_Confidence_0.067_Length_716	77.4	135
EOG09370WYG	Duplicated	vo_n2_Locus_11647_Transcript_1_1_Confidence_0.000_Length_740	76.0	133
EOG09370WYG	Duplicated	vo_n3_Locus_501_Transcript_56_67_Confidence_0.061_Length_1078	76.2	135
EOG09370WZX	Duplicated	vo_c1_Locus_17920_Transcript_4_4_Confidence_0.250_Length_826	57.8	116
EOG09370WZX	Duplicated	vo_n3_Locus_24308_Transcript_2_3_Confidence_0.667_Length_968	57.8	116
EOG09370WZX	Duplicated	vo_n3_Locus_24308_Transcript_3_3_Confidence_0.000_Length_1053	54.6	112
EOG09370X1T	Missing			
EOG09370XFG	Complete	vo_c1_Locus_17033_Transcript_1_1_Confidence_0.000_Length_781	41.4	100
EOG09370XFJ	Complete	vo_c2_Locus_331_Transcript_2_4_Confidence_0.667_Length_1647	322.6	356
EOG09370XO6	Duplicated	vo_c2_Locus_4326_Transcript_1_2_Confidence_0.333_Length_603	135.5	121
EOG09370XO6	Duplicated	vo_n3_Locus_6524_Transcript_2_3_Confidence_0.400_Length_1865	134.2	130
EOG09370Y63	Duplicated	tri_c_comp17048_c0_seq4	205.3	133
EOG09370Y63	Duplicated	vo_c3_Locus_2228_Transcript_1_8_Confidence_0.792_Length_644	203.0	132

EOG09370Y63	Duplicated	vo_n1_Locus_3700_Transcript_5_13_Confidence_0.235_Length_627	205.5	133
EOG09370YC4	Duplicated	tri_n_comp25535_c0_seq1	83.5	153
EOG09370YC4	Duplicated	vo_n2_Locus_2906_Transcript_1_1_Confidence_0.000_Length_895	83.8	153
EOG09370YCN	Complete	vo_c3_Locus_8549_Transcript_1_1_Confidence_0.000_Length_686	86.1	115
EOG09370YSI	Missing			
EOG09370ZI7	Complete	vo_n1_Locus_1223_Transcript_4_4_Confidence_0.200_Length_4281	52.6	106
EOG09370ZOK	Missing			
EOG0937106D	Missing			
EOG0937106Y	Duplicated	tri_n_comp40576_c0_seq1	96.6	120
EOG0937106Y	Duplicated	vo_c1_Locus_17884_Transcript_1_4_Confidence_0.571_Length_1084	100.4	120
EOG09371088	Complete	vo_n2_Locus_22654_Transcript_1_1_Confidence_0.000_Length_691	141.8	138
EOG093710A7	Complete	vo_c1_Locus_800_Transcript_1_1_Confidence_0.000_Length_1094	53.1	94
EOG093710JH	Duplicated	tri_n_comp45206_c0_seq1	30.9	85
EOG093710JH	Duplicated	vo_c1_Locus_144_Transcript_15_17_Confidence_0.097_Length_2228	30.2	69
EOG093710SY	Duplicated	vo_c1_Locus_10992_Transcript_1_1_Confidence_0.000_Length_882	151.2	106
EOG093710SY	Duplicated	vo_c2_Locus_14944_Transcript_1_2_Confidence_1.000_Length_1236	148.5	122
EOG093710SY	Duplicated	vo_c2_Locus_14944_Transcript_1_2_Confidence_1.000_Length_1236	149.5	106
EOG093710SY	Duplicated	vo_n2_Locus_3635_Transcript_1_2_Confidence_1.000_Length_748	128.9	97
EOG093710T1	Duplicated	tri_c_comp21721_c0_seq1	200.6	118
EOG093710T1	Duplicated	vo_c1_Locus_1693_Transcript_6_51_Confidence_0.053_Length_551	201.0	118
EOG093710T1	Duplicated	vo_c2_Locus_245_Transcript_7_11_Confidence_0.241_Length_591	200.8	118
EOG0937112Y	Fragmented	vo_n2_Locus_20951_Transcript_3_5_Confidence_0.000_Length_595	95.3	89
EOG093711E9	Complete	vo_c1_Locus_8125_Transcript_1_2_Confidence_0.667_Length_569	156.0	114
EOG093711LC	Duplicated	vo_n2_Locus_5115_Transcript_1_1_Confidence_0.000_Length_1108	59.3	79
EOG093711LC	Duplicated	vo_n2_Locus_5115_Transcript_1_1_Confidence_0.000_Length_1108	59.3	79
EOG093711QY	Complete	vo_n1_Locus_2982_Transcript_4_17_Confidence_0.192_Length_2025	85.0	97
EOG093711ZO	Duplicated	tri_n_comp29947_c0_seq1	85.4	158
EOG093711ZO	Duplicated	vo_c2_Locus_12685_Transcript_1_1_Confidence_0.000_Length_980	79.9	187
EOG0937122Q	Missing			
EOG0937128O	Complete	vo_n1_Locus_25878_Transcript_2_2_Confidence_0.000_Length_719	81.9	86
EOG0937129K	Missing			

EOG093712G8	Missing			
EOG093712Q6	Missing			
EOG09371330	Missing			
EOG0937133J	Duplicated	vo_c2_Locus_4120_Transcript_11_21_Confidence_0.244_Length_613	182.0	111
EOG0937133J	Duplicated	vo_n1_Locus_1030_Transcript_11_27_Confidence_0.345_Length_567	182.6	111
EOG0937133J	Duplicated	vo_n3_Locus_5371_Transcript_5_16_Confidence_0.281_Length_590	182.2	111
EOG093713HJ	Missing			
EOG093713LY	Duplicated	vo_c3_Locus_3728_Transcript_4_10_Confidence_0.103_Length_989	118.2	90
EOG093713LY	Duplicated	vo_c3_Locus_3728_Transcript_5_10_Confidence_0.000_Length_633	119.4	90
EOG093713LY	Duplicated	vo_n2_Locus_6571_Transcript_8_8_Confidence_0.038_Length_515	120.0	90
EOG09371431	Duplicated	vo_c1_Locus_4662_Transcript_17_20_Confidence_0.182_Length_666	166.0	97
EOG09371431	Duplicated	vo_c2_Locus_3455_Transcript_1_4_Confidence_0.833_Length_457	167.2	97
EOG093714JU	Missing			
EOG093714Q2	Complete	vo_c1_Locus_21978_Transcript_1_2_Confidence_0.333_Length_2296	67.0	69
EOG093715EC	Complete	tri_n_comp8120_c0_seq1	53.7	79
EOG09371675	Complete	tri_n_comp21879_c0_seq1	108.2	118
EOG0937167R	Complete	vo_c1_Locus_6297_Transcript_2_2_Confidence_0.500_Length_985	47.0	95
EOG093716T7	Duplicated	vo_c1_Locus_7043_Transcript_1_2_Confidence_0.750_Length_2755	140.4	226
EOG093716T7	Duplicated	vo_c1_Locus_7043_Transcript_2_2_Confidence_0.000_Length_2853	140.4	226
EOG093717LU	Missing			
EOG0937183G	Complete	vo_n1_Locus_17289_Transcript_2_2_Confidence_0.000_Length_445	73.6	67
EOG0937186Q	Missing			
EOG093718E9	Missing			
EOG093718EG	Complete	tri_c_comp13746_c0_seq2	71.1	58
EOG0937192A	Complete	vo_c3_Locus_32806_Transcript_1_1_Confidence_0.000_Length_1003	79.8	130
EOG093719IK	Duplicated	vo_c3_Locus_8674_Transcript_5_9_Confidence_0.182_Length_1753	98.3	102
EOG093719IK	Duplicated	vo_n2_Locus_5831_Transcript_1_1_Confidence_0.000_Length_575	101.9	103
EOG093719M8	Duplicated	tri_c_comp27616_c0_seq1	37.8	63
EOG093719M8	Duplicated	vo_n2_Locus_29712_Transcript_1_1_Confidence_0.000_Length_670	39.1	64
EOG09371BIR	Missing			

Supplementary Table S9. DEG id, log2Fold Change, adjusted P value (padj), Sequence description and enzyme codes

id	log2 fold change	padj	Seq. Description	Enzyme Codes
vo_n2_Locus_5179_Transcript_5/19_Confidence_0.522_Length_3870	6,73798017	9,90E-13	tl15a_arath ame: full=thylakoid lumenal 15 kda protein chloroplastic ame: full=p15 flags: precursor	-
vo_c1_Locus_7371_Transcript_1/4_Confidence_0.500_Length_1352	6,49714632	7,24E-26	mdhm_fraan ame: full=malate mitochondrial flags: precursor	EC:1.1.1.37; EC:1.1.1.82
vo_c1_Locus_31255_Transcript_2/4_Confidence_0.444_Length_1598	6,47008907	0,00478469	oml6_orysj ame: full=protein mei2-like 6 short=oml6 ame: full=mei2-like protein 6	
vo_n2_Locus_636_Transcript_3/16_Confidence_0.306_Length_685	6,46253683	2,14E-11	dhb4_human ame: full=peroxisomal multifunctional enzyme type 2 short=mfe-2 ame: full=17-beta-hydroxysteroid dehydrogenase 4 short=17-beta-hsd 4 ame: full=d-bifunctional protein short=dbp ame: full=multifunctional protein 2 short=mpf-2 contains: ame: full= -hydroxyacyl- dehydrogenase contains: ame: full=enoyl- hydratase 2 ame: full=3- - -alpha-trihydroxy-5-beta-cholest-24-enoyl- hydratase	EC:1.1.1.35; EC:1.1.1.51; EC:4.2.1.74
vo_c1_Locus_1287_Transcript_24/49_Confidence_0.341_Length_2746	6,22766076	1,22E-09	y326_metja ame: full= permease mj0326	-
vo_n1_Locus_10677_Transcript_1/2_Confidence_0.333_Length_6802	6,22350109	2,05E-09	dpol_hbvc4 ame: full=protein p includes: ame: full=dna-directed dna polymerase includes: ame: full=rna-directed dna polymerase includes: ame: full=ribonuclease h	
vo_c3_Locus_3418_Transcript_17/28_Confidence_0.286_Length_802	6,16444896	7,73E-09	---NA---	
tri_n_comp33784_c0_seq1	6,03562084	9,94E-10	---NA---	
vo_c2_Locus_3191_Transcript_18/23_Confidence_0.300_Length_775	5,94168976	9,36E-08	dptor_mouse ame: full=dep domain-containing mtor-interacting protein ame: full=dep domain-containing protein 6	
vo_n3_Locus_16240_Transcript_2/3_Confidence_0.400_Length_3532	5,80187888	6,09E-07	star_drome ame: full=protein star	-

vo_n1_Locus_33218_Transcript_1/1_Confidence_0.000_Length_1206	5,72059668	8,87E-07	p4ha2_caeel ame: full=prolyl 4-hydroxylase subunit alpha-2 short=4-ph alpha-2 ame: full=procollagen- -oxoglutarate-4-dioxygenase subunit alpha-2 flags: precursor	EC:1.14.11
tri_c_comp21232_c1_seq44	5,66290247	2,47E-06	jmjd6_hydat ame: full=bifunctional arginine demethylase and lysyl-hydroxylase psr ame: full=phosphatidylserine receptor	-
vo_c2_Locus_19599_Transcript_2/5_Confidence_0.455_Length_2709	5,65334974	1,64E-06	galt1_mouse ame: full=polypeptide n-acetylgalactosaminyltransferase 1 ame: full=polypeptide c transferase 1 short= c-t1 short=pp- ase 1 ame: full=protein-udp acetylgalactosaminyltransferase 1 ame: full=udp-c:polypeptide n-acetylgalactosaminyltransferase 1 contains: ame: full=polypeptide n-acetylgalactosaminyltransferase 1 soluble form	-
vo_n2_Locus_15818_Transcript_1/10_Confidence_0.429_Length_2380	5,63220175	1,35E-06	polc2_junox ame: full=polcalcin jun o 2 ame: full=calcium-binding pollen allergen jun o 2 ame: allergen=jun o 2	-
vo_n2_Locus_5145_Transcript_8/9_Confidence_0.294_Length_1998	5,60392769	1,41E-06	deda_borbu ame: full=inner membrane protein bb_0250	
vo_n3_Locus_21257_Transcript_2/4_Confidence_0.375_Length_1948	5,52407512	8,80E-06	f90a2_human ame: full= protein fam90a2p	-
vo_n1_Locus_5727_Transcript_43/46_Confidence_0.134_Length_9569	5,46336031	1,43E-05	dhx57_human ame: full= atp-dependent rna helicase dhx57 ame: full=deah box protein 57	-
vo_c2_Locus_10763_Transcript_5/20_Confidence_0.388_Length_2919	5,44168069	2,93E-06	huth_rhosk ame: full=histidine ammonia-lyase short=histidase	
vo_c1_Locus_8663_Transcript_1/7_Confidence_0.300_Length_2289	5,31726267	1,88E-05	---NA---	
vo_n3_Locus_18699_Transcript_1/2_Confidence_0.500_Length_1893	5,31620674	1,88E-05	unc89_caeel ame: full=muscle m-line assembly protein unc-89 ame: full=uncoordinated protein 89	
vo_n1_Locus_25409_Transcript_1/1_Confidence_0.000_Length_1010	5,29910355	1,76E-05	---NA---	
vo_n3_Locus_724_Transcript_2/7_Confidence_0.500_Length_2248	5,25325823	8,44E-05	lpxa_sulnb ame: full=acyl-	

vo_n1_Locus_43261_Transcript_1/1_Confidence_0.000_Length_260	5,17941234	8,75E-05	polg_hcvjl ame: full=genome polyprotein contains: ame: full=core protein p21 ame: full=capsid protein c ame: full=p21 contains: ame: full=core protein p19 contains: ame: full=envelope glycoprotein e1 ame: full=gp32 ame: full=gp35 contains: ame: full=envelope glycoprotein e2 ame: full=ns1 ame: full=gp68 ame: full=gp70 contains: ame: full=p7 contains: ame: full=protease ns2-3 short=p23 contains: ame: full=serine protease ns3 ame: full=hepacivirin ame: full=ns3p ame: full=p70 contains: ame: full=non-structural protein 4a short=ns4a ame: full=p8 contains: ame: full=non-structural protein 4b short=ns4b ame: full=p27 contains: ame: full=non-structural protein 5a short=ns5a ame: full=p56 contains: ame: full=rna-directed rna polymerase ame: full=ns5b ame: full=p68	
vo_n2_Locus_7614_Transcript_1/1_Confidence_0.000_Length_1532	5,16685186	6,43E-05	lac4_arath ame: full=laccase-4 ame: full=benzenediol:oxygen oxidoreductase 4 ame: full=diphenol oxidase 4 ame: full=protein irregular xylem 12 ame: full=urishiol oxidase 4 flags: precursor	
tri_n_comp20382_c0_seq1	5,16139525	0,000171901	cyb_plach ame: full=cytochrome b ame: full=complex iii subunit 3 ame: full=complex iii subunit iii ame: full=cytochrome b-c1 complex subunit 3 ame: full=ubiquinol-cytochrome-c reductase complex cytochrome b subunit	-
vo_c2_Locus_3577_Transcript_1/5_Confidence_0.091_Length_1993	5,11790638	0,000233272	pepa_calja ame: full=pepsin a flags: precursor	EC:3.4.23
tri_n_comp20072_c0_seq9	5,11191746	3,33E-05	rcd1_macfa ame: full=cell differentiation protein rcd1 homolog short=rcd-1 ame: full=ccr4-not transcription complex subunit 9gi	-
vo_c3_Locus_24067_Transcript_1/2_Confidence_0.667_Length_217	5,09015053	6,85E-05	---NA---	
tri_n_comp50355_c0_seq1	5,03654577	7,23E-05	sec24_picpa ame: full=protein transport protein sec24	
vo_n3_Locus_22_Transcript_23/31_Confidence_0.030_Length_1941	5,02791281	0,00030066	---NA---	
vo_n2_Locus_34437_Transcript_1/1_Confidence_0.000_Length_791	4,95984028	0,000420263	gcs2_chlad ame: full= glutamate--cysteine ligase 2 ame: full=gamma-glutamylcysteine synthetase 2 short=gcs 2 short=gamma-gcs 2	

vo_n3_Locus_31555_Transcript_2/3_Confidence_0.333_Length_2001	4,9429584	0,000737771	mco_stahj ame: full=multicopper oxidase mco	-
vo_n2_Locus_9140_Transcript_22/26_Confidence_0.233_Length_3641	4,93440842	0,000171901	you1_caeel ame: full= transporter	-
vo_n1_Locus_1282_Transcript_9/28_Confidence_0.233_Length_1398	4,89968537	0,000434799	g3st1_human ame: full=galactosylceramide sulfotransferase short= er sulfotransferase ame: full=3 -phosphoadenosine-5 -phosphosulfate: er sulfotransferase ame: full=3 -phosphoadenylylsulfate:galactosylceramide 3 -sulfotransferase ame: full=cerebroside sulfotransferase	
tri_n_comp73516_c0_seq1	4,88781499	0,000592196	---NA---	
vo_n2_Locus_3134_Transcript_3/6_Confidence_0.615_Length_2515	4,86400047	0,000477135	---NA---	
vo_n1_Locus_7615_Transcript_12/21_Confidence_0.494_Length_2036	4,85959438	0,00125422	vncs_aedev ame: full=non-capsid protein ns-1 ame: full=ncvp1 ame: full=non-structural protein ns1	
vo_c3_Locus_2065_Transcript_17/73_Confidence_0.277_Length_1776	4,84803676	0,001379322	kv3a3_mouse ame: full=ig kappa chain v-iii region mopc 70	
vo_c1_Locus_12555_Transcript_1/7_Confidence_0.455_Length_294	4,83714528	0,000502091	pkho1_danre ame: full=pleckstrin homology domain-containing family o member 1-a short=ph domain-containing family o member 1-a ame: full=casein kinase 2-interacting protein 1 short=ck2-interacting protein 1 short=ckip-1	
vo_c2_Locus_5926_Transcript_10/12_Confidence_0.333_Length_2827	4,75591326	0,001346055	gpma_thetn ame: full= -bisphosphoglycerate-dependent phosphoglycerate mutase short=bpg-dependent pgam short=pgam short=phosphoglyceromutase short=dpgm	-
tri_n_comp7767_c0_seq2	4,74907704	0,000680253	nrps7_aspfu ame: full=nonribosomal peptide synthetase 7	-
tri_n_comp20382_c0_seq94	4,71818322	0,001166407	gt251_bovin ame: full=procollagen galactosyltransferase 1 ame: full=collagen beta(1-o)galactosyltransferase 1 ame: full=glycosyltransferase 25 family member 1 ame: full=hydroxylysine galactosyltransferase 1 flags: precursor	
vo_n3_Locus_36529_Transcript_2/3_Confidence_0.333_Length_2968	4,70131398	0,003453619	ppsa_mycto ame: full=phthiocerol synthesis polyketide synthase type i ame: full=beta-ketoacyl-acyl-carrier-protein synthase i	-
vo_c1_Locus_1107_Transcript_1/4_Confidence_0.143_Length_1311	4,68357786	0,003778206	apx7_orysj ame: full=probable l-ascorbate peroxidase chloroplastic ame: full= x07 flags: precursor	EC:1.11.1.7

tri_c_comp21232_c1_seq89	4,67902213	0,003585906	jmjd6_hydat ame: full=bifunctional arginine demethylase and lysyl-hydroxylase psr ame: full=phosphatidylserine receptor	-
vo_n1_Locus_16253_Transcript_1/1_Confidence_0.000_Length_673	4,64947086	0,000787869	chm4c_human ame: full=charged multivesicular body protein 4c ame: full=chromatin-modifying protein 4c short=chmp4c ame: full=snf7 homolog associated with alix 3 ame: full=snf7-3 short=hsnf7-3 ame: full=vacuolar protein sorting-associated protein 32-3 short=vps32-3 short=hvps32-3	
vo_n1_Locus_25036_Transcript_1/1_Confidence_0.000_Length_1116	4,63192454	0,002772061	mtip_metka ame: full=probable s-methyl-5 -thioinosine phosphorylase ame: full=5 -methylthioinosine phosphorylase short=mti phosphorylase short=mtip	-
vo_c1_Locus_2394_Transcript_16/42_Confidence_0.323_Length_1978	4,61343379	0,005165988	amt12_arath ame: full=ammonium transporter 1 member 2 short= 1 2	-
vo_c2_Locus_8806_Transcript_6/17_Confidence_0.306_Length_875	4,61343379	0,005165988	---NA---	
tri_n_comp14749_c0_seq1	4,58418222	0,002252194	tc159_arath ame: full=translocase of chloroplast chloroplastic short= oc159 ame: full=159 kda chloroplast outer envelope protein ame: full=plastid protein import 2 ame: full=translocase of chloroplast chloroplastic short= oc160 ame: full=translocase of chloroplast chloroplastic short= oc86	
tri_n_comp19741_c0_seq16	4,54491663	0,003403828	---NA---	
vo_n1_Locus_6568_Transcript_1/1_Confidence_0.667_Length_239	4,53783714	0,003453619	dapd_rhort ame: full= -tetrahydropyridine- -dicarboxylate n-succinyltransferase ame: full=tetrahydrodipicolinate n-succinyltransferase short=thdp succinyltransferase short=thp succinyltransferase short=tetrahydropicolinate succinylase	
vo_c1_Locus_31_Transcript_162/1155_Confidence_0.005_Length_446	4,53634688	0,004543773	vglm_tswv ame: full=envelope glycoprotein short=gp ame: full=m polyprotein contains: ame: full=glycoprotein g1 contains: ame: full=glycoprotein g2 flags: precursor	
vo_c3_Locus_1097_Transcript_26/47_Confidence_0.267_Length_2975	4,53547651	0,003046928	pof9_schpo ame: full=f-box protein pof9	
vo_n1_Locus_12248_Transcript_11/19_Confidence_0.462_Length_6817	4,49626944	0,00553337	ans1b_danre ame: full=ankyrin repeat and sterile alpha motif domain-containing protein 1b	EC:3.4.22
vo_n3_Locus_31357_Transcript_7/9_Confidence_0.538_Length_1281	4,4775918	0,004756059	huti_flaj1 ame: full=imidazolonepropionase ame: full=imidazolone-5-propionate hydrolase	
tri_c_comp20467_c0_seq3	4,38884661	0,015870763	---NA---	

tri_c_comp21232_c1_seq99	4,37324182	0,009895515	jmjd6_hydat ame: full=bifunctional arginine demethylase and lysyl-hydroxylase psr ame: full=phosphatidylserine receptor	-
vo_n1_Locus_4757_Transcript_1/2_Confidence_0.750_Length_684	4,35181787	0,016134458	oaf1_yeast ame: full=oleate-activated transcription factor 1	
vo_c1_Locus_7414_Transcript_11/26_Confidence_0.551_Length_5434	4,31268593	0,02515293	svep1_human ame: full= von willebrand factor type egf and pentraxin domain-containing protein 1 ame: full=ccp module-containing protein 22 ame: full=polydom ame: full=selectin-like osteoblast-derived protein short=sel-ob ame: full=serologically defined breast cancer antigen ny-br-38 flags: precursor	-
vo_c1_Locus_8135_Transcript_68/78_Confidence_0.238_Length_6119	4,27075484	0,030686812	grm4_human ame: full=metabotropic glutamate receptor 4 short=m 4 flags: precursor	-
vo_n2_Locus_3666_Transcript_11/13_Confidence_0.343_Length_3404	4,26632661	0,016405138	clpc_guith ame: full=atp-dependent clp protease atp-binding subunit clpa homolog	-
vo_n3_Locus_24788_Transcript_1/1_Confidence_0.000_Length_902	4,2554962	0,018233577	gatc_opitp ame: full=aspartyl glutamyl-trna(asn gln) amidotransferase subunit c short=asp glu-adt subunit c	
vo_c2_Locus_9395_Transcript_1/2_Confidence_0.920_Length_1509	4,25341292	0,018233577	rng1l_arath ame: full=e3 ubiquitin-protein ligase ring1-like ame: full=ring finger protein 1	-
vo_n2_Locus_206_Transcript_94/103_Confidence_0.005_Length_4232	4,25046648	0,018233577	cubn_mouse ame: full=cubilin ame: full=intrinsic factor-cobalamin receptor flags: precursor	
tri_n_comp19462_c0_seq11	4,23999512	0,022012691	kdm6a_human ame: full=lysine-specific demethylase 6a ame: full=histone demethylase utx ame: full=ubiquitously-transcribed tpr protein on the x chromosome ame: full=ubiquitously-transcribed x chromosome tetratricopeptide repeat protein	
vo_n2_Locus_3_Transcript_20543/24291_Confidence_0.000_Length_3890	4,22284199	0,031240268	hs7c1_dicdi ame: full=heat shock cognate 70 kda protein 1 short=hsc70-1	-
vo_c3_Locus_10183_Transcript_2/22_Confidence_0.500_Length_2470	4,21547909	0,034315221	r1b17_solde ame: full= late blight resistance protein homolog r1b-17	
vo_n1_Locus_7190_Transcript_5/7_Confidence_0.667_Length_3010	4,20450332	0,022327152	kcnae_drome ame: full=potassium voltage-gated channel protein eag ame: full=ether-a-go-go protein	-

vo_n1_Locus_7034_Transcript_4/6_Confidence_0.692_Length_1788	4,20369243	0,023444485	kpcd2_rat ame: full=serine threonine-protein kinase d2 ame: full=npkc-d2	
vo_n2_Locus_1445_Transcript_12/24_Confidence_0.250_Length_1016	4,19402335	0,016856025	pnta_ecoli ame: full=nad transhydrogenase subunit alpha ame: full=nicotinamide nucleotide transhydrogenase subunit alpha ame: full=pyridine nucleotide transhydrogenase subunit alpha	
tri_c_comp19578_c0_seq4	4,13532323	0,014067346	---NA---	
vo_n1_Locus_17075_Transcript_2/4_Confidence_0.400_Length_811	4,09414661	0,038417345	ab23g_arath ame: full=abc transporter g family member 23 short=abc transporter short= 23 ame: full=probable white-brown complex homolog protein 24 short= 24	EC:3.6.1.15
vo_n2_Locus_19192_Transcript_2/2_Confidence_0.250_Length_3053	4,08609721	0,040683605	manea_rat ame: full=glycoprotein endo-alpha- -mannosidase short=endo-alpha mannosidase short=endomannosidase short=rendo ame: full=endo-alpha-d-mannosidase short=enman	EC:2.3.1.22; EC:3.2.1.24; EC:2.4.1.101; EC:2.4.1.94; EC:2.3.1.20
tri_n_comp16805_c0_seq2	4,08247364	0,039106281	ung_vzvo ame: full=uracil-dna glycosylase short=udg ame: full=ung	
vo_c2_Locus_10226_Transcript_61/67_Confidence_0.410_Length_3306	4,07449335	0,034315221	pmp19_chlpn ame: full=probable outer membrane protein pmp19 ame: full=polymorphic membrane protein 19 flags: precursor	
tri_n_comp16502_c0_seq1	4,07195068	0,040614214	prta_dicch ame: full=serralysin a ame: full=secreted protease a short= flags: precursor	
tri_n_comp54988_c0_seq1	4,04696204	0,040614214	rr3_chavu ame: full=30s ribosomal protein chloroplastic	
vo_c2_Locus_19266_Transcript_5/9_Confidence_0.632_Length_1844	4,04696204	0,040614214	cac1d_human ame: full=voltage-dependent l-type calcium channel subunit alpha-1d ame: full=calcium l alpha-1 isoform 2 ame: full=voltage-gated calcium channel subunit alpha	
vo_c1_Locus_4958_Transcript_8/15_Confidence_0.615_Length_2367	4,04442198	0,0447359	dnjh_cucsa ame: full= protein homolog ame: full=dnaj-1 flags: precursor	
vo_n1_Locus_19358_Transcript_1/2_Confidence_0.333_Length_3813	4,01894767	0,0447359	gso2_arath ame: full=lrr receptor-like serine threonine-protein kinase gso2 ame: full=protein embryo sac development arrest 23 ame: full=protein gassho 2 flags: precursor	-
vo_c3_Locus_6066_Transcript_21/22_Confidence_0.202_Length_2076	3,9964015	0,0447359	---NA---	
vo_n2_Locus_492_Transcript_3/4_Confidence_0.429_Length_2376	3,98825969	0,028427964	---NA---	

vo_n2_Locus_4317_Transcript_3/3_Confidence_0.200_Length_590	3,98523429	0,024565634	---NA---	
vo_c3_Locus_438_Transcript_13/50_Confidence_0.139_Length_756	3,96301415	0,023444485	---NA---	-
tri_n_comp72938_c0_seq1	3,95104806	0,044567259	murc_rhiec ame: full=udp-n-acetylmuramate--l-alanine ligase ame: full=udp-n-acetylmuramoyl-l-alanine synthetase	
tri_n_comp46573_c0_seq1	3,93687959	0,029892879	kn12b_arath ame: full=kinesin-like protein kin12b ame: full=phragmoplast-associated kinesin-related protein 1-like protein short= 1l	
vo_n2_Locus_3470_Transcript_2/6_Confidence_0.727_Length_879	3,88639277	0,044468428	mob1a_mouse ame: full=mob kinase activator 1a ame: full=mob1 homolog 1b ame: full=mps one binder kinase activator-like 1b	-
tri_c_comp20671_c0_seq32	3,87152266	0,040073493	lorf2_human ame: full=line-1 retrotransposable element orf2 protein short=orf2p includes: ame: full=reverse transcriptase includes: ame: full=endonuclease	
vo_n1_Locus_39093_Transcript_3/5_Confidence_0.300_Length_1114	3,85835501	0,040073493	met13_mouse ame: full=methyltransferase-like protein 13	-
vo_n2_Locus_20353_Transcript_1/1_Confidence_0.000_Length_259	3,78577984	0,044970833	btbba_danre ame: full=ankyrin repeat and btb poz domain-containing protein btbd11-a ame: full=btb poz domain-containing protein 11-a	
tri_c_comp20609_c0_seq8	3,56290867	0,0267341	thii_metth ame: full=probable trna sulfurtransferase ame: full=sulfur carrier protein sulfurtransferase ame: full=thiamine biosynthesis protein ame: full=trna 4-thiouridine synthase	
vo_n1_Locus_1043_Transcript_4/19_Confidence_0.581_Length_1867	2,23226077	0,028563022	epn2_arath ame: full=clathrin interactor epsin 2 ame: full=epsin-related 2	
vo_c2_Locus_189_Transcript_40/60_Confidence_0.152_Length_1470	1,84683527	0,024565634	clip3_ponab ame: full=cap-gly domain-containing linker protein 3 ame: full=cytoplasmic linker protein 170-related 59 kda protein short=clip-170-related 59 kda protein short=clipr-59	-
vo_n1_Locus_9015_Transcript_1/5_Confidence_0.667_Length_2154	1,82966715	0,040592726	tempt_aplca ame: full=temptin flags: precursor	
vo_n3_Locus_427_Transcript_37/175_Confidence_0.071_Length_2114	0,95705745	0,003736617	nrt25_arath ame: full=high affinity nitrate transporter short= 2:5	-

vo_n3_Locus_138_Transcript_26/29_Confidence_0.190_Length_1802	0,76750219	0,000967552	fcp_isoga ame: full=fucoxanthin-chlorophyll a-c binding chloroplastic short=fcp flags: precursor	-
vo_n1_Locus_16_Transcript_169/225_Confidence_0.061_Length_1117	0,70880589	8,93E-05	hsp90_eimte ame: full=heat shock protein 90	-
vo_c2_Locus_446_Transcript_3/9_Confidence_0.353_Length_489	0,66605701	0,01863807	gata4_chick ame: full=transcription factor gata-4 ame: full=gata-binding factor partial	
vo_c1_Locus_31_Transcript_932/1155_Confidence_0.008_Length_1081	0,62868879	0,041264197	trub2_rat ame: full=probable trna pseudouridine synthase 2	
vo_n2_Locus_561_Transcript_15/51_Confidence_0.279_Length_2571	0,60774172	0,000434799	nrt25_arath ame: full=high affinity nitrate transporter short= 2:5	-
tri_n_comp46903_c0_seq1	0,60614156	0,036082433	fmt_kosot ame: full=methionyl-trna formyltransferase	
vo_c3_Locus_25322_Transcript_1/1_Confidence_0.000_Length_1606	0,5924054	0,034315221	ltv1_xenla ame: full=protein ltv1 homolog	
tri_n_comp24956_c0_seq1	0,58955164	3,33E-05	nia1_orysj ame: full=nitrate reductase	EC:1.7.1.1
vo_n2_Locus_128_Transcript_8/33_Confidence_0.085_Length_4154	0,51890657	0,000592196	nirb_kleox ame: full=nitrite reductase	EC:1.7.1.4
vo_c1_Locus_5705_Transcript_16/19_Confidence_0.489_Length_4193	-0,5239369	0,037710321	idh_azovi ame: full=isocitrate dehydrogenase	EC:1.1.1.42
vo_c1_Locus_11109_Transcript_8/9_Confidence_0.600_Length_4491	-0,5732894	0,034315221	kcnh2_human ame: full=potassium voltage-gated channel subfamily h member 2 ame: full=eag homolog ame: full=ether-a-go-go-related gene potassium channel 1 short=erg-1 short=eag-related protein 1 short=ether-a-go-go-related protein 1 short=h-erg short=herg-1 short=herg1 ame: full=voltage-gated potassium channel subunit	-
vo_n2_Locus_1515_Transcript_20/24_Confidence_0.103_Length_4140	-0,5813126	0,000455968	fks1_crynh ame: full= -beta-glucan synthase component fks1	EC:2.7.11; EC:2.4.1.34
vo_c2_Locus_4642_Transcript_1/2_Confidence_0.333_Length_1565	-0,6032733	0,000175679	aml1_arath ame: full=protein mei2-like 1 short=aml1 ame: full=mei2-like protein 1	-

tri_c_comp10077_c0_seq1	-0,6138635	0,034627737	kcnh6_human ame: full=potassium voltage-gated channel subfamily h member 6 ame: full=ether-a-go-go-related gene potassium channel 2 short=erg-2 short=eag-related protein 2 short=ether-a-go-go-related protein 2 short=herg-2 short=herg2 ame: full=voltage-gated potassium channel subunit	-
vo_n2_Locus_2148_Transcript_1/1_Confidence_0.000_Length_3586	-0,6187105	0,005166346	kcnae_drome ame: full=potassium voltage-gated channel protein eag ame: full=ether-a-go-go protein	-
tri_n_comp57238_c0_seq1	-0,6780797	0,022189548	cnga1_human ame: full=cgmp-gated cation channel alpha-1 ame: full=cyclic nucleotide-gated cation channel 1 ame: full=cyclic nucleotide-gated channel alpha-1 short=cng channel alpha-1 short=cng-1 short=cng1 ame: full=cyclic nucleotide-gated photoreceptor ame: full=rod photoreceptor cgmp-gated channel subunit alpha	-
vo_n2_Locus_713_Transcript_13/28_Confidence_0.321_Length_2527	-0,7421177	0,010964152	y326_metja ame: full= permease mj0326	-
tri_n_comp58611_c0_seq1	-0,7691416	0,000898441	pk2_dicdi ame: full=protein kinase 2 short=pk2	EC:2.7.11
tri_c_comp19120_c0_seq2	-0,9386599	0,00200196	y326_metja ame: full= permease mj0326	-
tri_c_comp14057_c0_seq1	-3,9637236	0,041264197	m3k7l_drome ame: full= mitogen-activated protein kinase kinase kinase 7-like	-
tri_c_comp16850_c0_seq7	-4,0023428	0,044970833	tydp1_human ame: full=tyrosyl-dna phosphodiesterase 1 short=tyr-dna phosphodiesterase 1	
vo_c1_Locus_26724_Transcript_2/11_Confidence_0.571_Length_1308	-4,016307	0,038417345	eri1_rat ame: full=3 -5 exoribonuclease 1 ame: full=histone mrna 3 -exonuclease 1	EC:3.1.13
vo_c3_Locus_17701_Transcript_5/6_Confidence_0.222_Length_1749	-4,0377185	0,040614214	ilvi_salty ame: full=acetolactate synthase isozyme 3 large subunit ame: full=ahas-iii ame: full=als-iii ame: full=acetohydroxy-acid synthase iii large subunit	
tri_c_comp19658_c1_seq3	-4,0703976	0,048356611	arap3_mouse ame: full=arf-gap with rho-gap ank repeat and ph domain-containing protein 3 ame: full=centaurin-delta-3 short=cnt-d3 ame: full=dual specificity rho- and arf-gtpase-activating protein 1	
vo_c3_Locus_3763_Transcript_1/4_Confidence_0.375_Length_285	-4,0847487	0,016861725	---NA---	

vo_c3_Locus_2504_Transcript_24/34_Confidence_0.330_Length_1270	-4,0866014	0,049309357	mst4_human ame: full=serine threonine-protein kinase mst4 ame: full=mammalian ste20-like protein kinase 4 short=mst-4 ame: full=mst3 and sok1-related kinase ame: full=ste20-like kinase mst4 ame: full=serine threonine-protein kinase mask	EC:2.7.11
vo_c1_Locus_8568_Transcript_1/2_Confidence_0.750_Length_1624	-4,1084381	0,013639185	bh013_arath ame: full=transcription factor bhlh13 ame: full=basic helix-loop-helix protein 13 short= 13 short=bhlh 13 ame: full=transcription factor en 39 ame: full=bhlh transcription factor bhlh013	
vo_c3_Locus_2570_Transcript_8/49_Confidence_0.062_Length_4245	-4,1268874	0,022012691	y6689_dicdi ame: full=protein ddb_g0276689	-
vo_n1_Locus_1154_Transcript_68/131_Confidence_0.027_Length_2639	-4,1347009	0,032439123	rp25l_mouse ame: full=ribonuclease p protein subunit p25-like protein short=rnase p protein subunit-like p25 ame: full=rpp25-like protein	-
tri_c_comp20063_c0_seq11	-4,1383802	0,024961718	spo22_yeast ame: full=sporulation-specific protein 22	
vo_c1_Locus_13264_Transcript_4/7_Confidence_0.467_Length_2394	-4,1441967	0,037284653	fmnl3_mouse ame: full=formin-like protein 3	
tri_c_comp44583_c0_seq1	-4,171994	0,035016479	coca1_chick ame: full=collagen alpha-1 chain ame: full=fibrochimerin flags: precursor	
vo_c1_Locus_31_Transcript_272/1155_Confidence_0.009_Length_1491	-4,2020786	0,031240268	fcpa_macpy ame: full=fucoxanthin-chlorophyll a-c binding protein chloroplastic flags: precursor	-
tri_c_comp20609_c0_seq12	-4,2542164	0,016861725	lac8_arath ame: full=laccase-8 ame: full=benzenediol:oxygen oxidoreductase 8 ame: full=diphenol oxidase 8 ame: full=urishiol oxidase 8 flags: precursor	
vo_c1_Locus_103_Transcript_38/42_Confidence_0.156_Length_1066	-4,2565264	0,024445611	rgpa1_rat ame: full=ral gtpase-activating protein subunit alpha-1 ame: full=gap-related-interacting partner to e12 short=gripe ame: full=gtpase-activating domain-like 1 ame: full=tuberin-like protein 1 ame: full=p240	
vo_c3_Locus_2096_Transcript_2/10_Confidence_0.125_Length_2147	-4,2604135	0,01511993	---NA---	
tri_n_comp20796_c0_seq13	-4,2682556	0,0154194	if2_cloph ame: full=translation initiation factor if-2	
vo_c3_Locus_291_Transcript_10/90_Confidence_0.022_Length_2582	-4,2711539	0,015422492	sossb_dicdi ame: full=soss complex subunit b homolog	-

vo_n1_Locus_734_Transcript_24/29_Confidence_0.236_Length_1766	-4,2829986	0,021847292	df142_arath ame: full= defensin-like protein 142 ame: full= low-molecular-weight cysteine-rich protein 34 short=protein lcr34 flags: precursor	
vo_c1_Locus_2693_Transcript_1/1_Confidence_0.000_Length_706	-4,2998795	0,018233577	nog2_schpo ame: full=nucleolar gtp-binding protein 2	
vo_c2_Locus_19170_Transcript_2/2_Confidence_0.000_Length_742	-4,3275444	0,008892159	dod_drome ame: full= peptidyl-prolyl cis-trans isomerase dodo short=ppiase dodo ame: full=rotamase dodo	EC:5.2.1.8
tri_n_comp20618_c0_seq20	-4,3322507	0,016861725	upp_chrsd ame: full=uracil phosphoribosyltransferase ame: full=ump pyrophosphorylase ame: full=uprtase	
vo_n1_Locus_216_Transcript_40/55_Confidence_0.104_Length_2361	-4,3345289	0,016861725	muty_mycto ame: full=a g-specific adenine glycosylase	
vo_n2_Locus_3_Transcript_4226/24291_Confidence_0.000_Length_427	-4,3416996	0,015432585	murd_causk ame: full=udp-n-acetylmuramoylalanine--d-glutamate ligase ame: full=d-glutamic acid-adding enzyme ame: full=udp-n-acetylmuramoyl-l-alanyl-d-glutamate synthetase	
vo_c1_Locus_18312_Transcript_1/1_Confidence_0.000_Length_295	-4,4002792	0,006382506	opgg_niteu ame: full=glucans biosynthesis protein g flags: precursor	
vo_c3_Locus_950_Transcript_16/59_Confidence_0.031_Length_783	-4,4077084	0,009884449	---NA---	
vo_c1_Locus_7775_Transcript_2/3_Confidence_0.667_Length_915	-4,42187	0,009895515	glmm_psef5 ame: full=phosphoglucosamine mutase	
tri_c_comp23668_c0_seq1	-4,4264219	0,005547491	f10al_arath ame: full=fam10 family protein at4g22670	-
vo_c3_Locus_31_Transcript_32/79_Confidence_0.035_Length_3193	-4,5580505	0,002890162	yg35_yeast ame: full=uncharacterized vacuolar membrane protein ygr125w	
vo_c1_Locus_11474_Transcript_18/25_Confidence_0.222_Length_1308	-4,5593642	0,002225433	te1_maize ame: full=protein terminal ear1	-
tri_c_comp21200_c0_seq4	-4,5677281	0,004520248	tsad_cuptr ame: full=trna n6-adenosine threonylcarbamoyltransferase ame: full=n6-l-threonylcarbamoyladenine synthase short=t a synthase ame: full=t a37 threonylcarbamoyladenosine biosynthesis protein ame: full=trna threonylcarbamoyladenosine biosynthesis protein	

vo_c1_Locus_15341_Transcript_4/4_Confidence_0.333_Length_950	-4,5717868	0,002644891	ftsh2_therp ame: full=atp-dependent zinc metalloprotease 2	
tri_c_comp19736_c0_seq15	-4,5744917	0,001755161	nrt_drome ame: full=neurotactin	-
tri_c_comp2038_c0_seq1	-4,604734	0,003403828	idhp_pig ame: full=isocitrate dehydrogenase	
vo_n1_Locus_8670_Transcript_15/23_Confidence_0.500_Length_5737	-4,6101634	0,003453619	hs3s4_human ame: full=heparan sulfate glucosamine 3-o-sulfotransferase 4 ame: full=heparan sulfate d-glucosaminyl 3-o-sulfotransferase 4 short=3-ost-4 short=heparan sulfate 3-o-sulfotransferase 4 short=h3-ost-4	
vo_c1_Locus_13749_Transcript_3/5_Confidence_0.500_Length_2621	-4,6218076	0,003062513	dnaj_trow8 ame: full=chaperone protein	-
tri_c_comp5651_c0_seq1	-4,6439651	0,001381403	y187_brebn ame: full= atp:guanido phosphotransferase bbr47_01870	
tri_c_comp19424_c0_seq3	-4,6505002	0,001137135	goga4_human ame: full=golgin subfamily a member 4 ame: full=256 kda golgin ame: full=golgin-245 ame: full=protein ame: full=trans-golgi p230	
vo_c2_Locus_9868_Transcript_4/8_Confidence_0.667_Length_2885	-4,665172	0,001207847	ano7_rat ame: full=anoctamin-7 ame: full=new gene expressed in prostate homolog ame: full=transmembrane protein 16g	-
vo_n1_Locus_678_Transcript_14/19_Confidence_0.340_Length_1076	-4,6718482	0,002234295	rl9_orysj ame: full=60s ribosomal protein l9	-
vo_n1_Locus_5719_Transcript_37/41_Confidence_0.500_Length_5524	-4,6772437	0,00179619	pi5k6_arath ame: full=phosphatidylinositol 4-phosphate 5-kinase 6 short=5k6 ame: full=1-phosphatidylinositol 4-phosphate kinase 6 ame: full=diphosphoinositide kinase 6 ame: full= ns p-5-kinase 6	EC:2.7.1.68
vo_n1_Locus_10454_Transcript_5/5_Confidence_0.143_Length_3714	-4,687121	0,001926888	ure1_rhie6 ame: full=urease subunit alpha ame: full=urea amidohydrolase subunit alpha	
vo_n2_Locus_3_Transcript_21438/24291_Confidence_0.000_Length_1004	-4,7356165	0,00125422	foxn4_xenla ame: full=forkhead box protein n4	
vo_n3_Locus_3273_Transcript_19/31_Confidence_0.157_Length_6208	-4,7483112	0,001297948	pag_bacan ame: full=protective antigen short=pa ame: full=anthrax toxins translocating protein ame: full=pa-83 short=pa83 contains: ame: full=protective antigen pa-20 short=pa20 contains: ame: full=protective antigen pa-63 short=pa63 flags: precursor	

vo_c2_Locus_6903_Transcript_12/49_Confidence_0.355_Length_6039	-4,7576215	0,001014085	vat1_danre ame: full=synaptic vesicle membrane protein vat-1 homolog	-
vo_c1_Locus_5025_Transcript_2/9_Confidence_0.690_Length_478	-4,7684255	0,00125422	catr_schdu ame: full=caltractin ame: full=centrin	-
vo_c1_Locus_1697_Transcript_1/6_Confidence_0.273_Length_11684	-4,7822722	0,00110171	dek1_orysj ame: full=calpain-type cysteine protease adl1 ame: full=phytocalpain adl1 ame: full=protein adaxialized leaf1 ame: full=protein defective kernel 1 short= 1 ame: full=protein shootless 3 flags: precursor	
vo_c3_Locus_33886_Transcript_1/1_Confidence_0.000_Length_922	-4,841865	0,000513901	syr_ralso ame: full=arginine--trna ligase ame: full=arginyl-trna synthetase short=	
tri_n_comp9316_c0_seq2	-4,8703084	0,000469136	---NA---	
vo_c1_Locus_21325_Transcript_1/3_Confidence_0.250_Length_371	-4,8784872	0,000469136	mura_olico ame: full=udp-n-acetylglucosamine 1-carboxyvinyltransferase ame: full=enoylpyruvate transferase ame: full=udp-n-acetylglucosamine enolpyruvyl transferase short=ept	
vo_c2_Locus_23160_Transcript_16/16_Confidence_0.238_Length_3292	-4,8785246	0,000470656	pcsk5_human ame: full=proprotein convertase subtilisin kexin type 5 ame: full=proprotein convertase 5 short=pc5 ame: full=proprotein convertase 6 short=pc6 short=hpc6 ame: full=subtilisin kexin-like protease pc5 flags: precursor	EC:3.4.21
tri_c_comp17100_c0_seq5	-4,8940477	0,000420263	y3475_arath ame: full=probable lrr receptor-like serine threonine-protein kinase at3g47570 flags: precursor	-
vo_c3_Locus_14253_Transcript_15/18_Confidence_0.184_Length_1257	-4,8955307	0,000502091	yhfe_bacsu ame: full= aminopeptidase	
vo_c2_Locus_15438_Transcript_6/11_Confidence_0.357_Length_2407	-4,9310049	0,000219152	chmp3_rat ame: full=charged multivesicular body protein 3 ame: full=chromatin-modifying protein 3 ame: full=vacuolar protein sorting-associated protein 24 short=rvps24p	-
vo_n1_Locus_187_Transcript_69/100_Confidence_0.030_Length_569	-4,9538836	0,000322732	rl27b_yeast ame: full=60s ribosomal protein l27-b	-
vo_c2_Locus_2195_Transcript_2/4_Confidence_0.816_Length_13071	-4,9619934	0,000305504	dyh7_rat ame: full=dynein heavy chain axonemal ame: full=axonemal beta dynein heavy chain 7 ame: full=axonemal dynein heavy chain b ame: full=ciliary dynein heavy chain 7 ame: full=dynein-like protein 7	-
vo_c1_Locus_3318_Transcript_22/22_Confidence_0.027_Length_480	-4,9658062	0,000289634	gbpa_vibpa ame: full= c-binding protein a flags: precursor	

vo_n1_Locus_7785_Transcript_6/10_Confidence_0.562_Length_1374	-4,9742689	0,000209806	xerc_ross1 ame: full=tyrosine recombinase	
vo_c2_Locus_9557_Transcript_1/3_Confidence_0.750_Length_3758	-4,9751331	9,11E-05	tpm_boomi ame: full=tropomyosin	
tri_c_comp20609_c0_seq10	-4,993492	0,000250167	---NA---	
vo_n1_Locus_913_Transcript_37/48_Confidence_0.315_Length_914	-5,0014804	0,000192425	bud4_ashgo ame: full=bud site selection protein 4 homolog	
tri_c_comp21276_c0_seq15	-5,0316109	7,25E-05	wbs22_dicdi ame: full=probable methyltransferase wbscr22 homolog	
tri_c_comp30303_c0_seq1	-5,0535461	0,00010133	---NA---	
vo_c3_Locus_6126_Transcript_5/9_Confidence_0.667_Length_2660	-5,0605608	0,000125979	cul3_rat ame: full=cullin-3	-
tri_c_comp17992_c0_seq9	-5,0662003	6,85E-05	cb043_human ame: full=upf0554 protein c2orf43	
vo_c2_Locus_9414_Transcript_9/10_Confidence_0.200_Length_798	-5,1008826	6,54E-05	gt253_bovin ame: full=probable inactive glycosyltransferase 25 family member 3 ame: full=cerebral endothelial cell adhesion molecule flags: precursor	
vo_c1_Locus_3347_Transcript_32/55_Confidence_0.134_Length_3787	-5,1258629	5,27E-05	m3k7_ponab ame: full=mitogen-activated protein kinase kinase kinase 7	EC:2.7.11; EC:3.4
vo_c1_Locus_19495_Transcript_1/3_Confidence_0.200_Length_1414	-5,133821	5,02E-05	prax_mouse ame: full=periaxin	
tri_c_comp7251_c0_seq2	-5,1550758	1,88E-05	ube4b_human ame: full=ubiquitin conjugation factor e4 b ame: full=homozygously deleted in neuroblastoma 1 ame: full=ubiquitin fusion degradation protein 2	
vo_c3_Locus_6426_Transcript_2/2_Confidence_0.250_Length_1098	-5,1793439	3,33E-05	fl2d_danre ame: full=pre-mrna-splicing regulator wtap ame: full=female-lethal d homolog ame: full=wt1-associated protein ame: full=wilms tumor 1-associating protein	-
vo_c1_Locus_31_Transcript_1137/1155_Confidence_0.029_Length_4296	-5,2158844	3,93E-05	fcpb_macpy ame: full=fucoxanthin-chlorophyll a-c binding protein chloroplastic flags: precursor	-

vo_c3_Locus_4574_Transcript_2/2_Confidence_0.000_Length_358	-5,2158844	3,93E-05	dpnp4_arath ame: full=probable sal4 phosphatase ame: full=3 (2) -bisphosphate nucleotidase 4 ame: full=3 (2) -bisphosphonucleoside 3 (2)-phosphohydrolase 4 ame: full=dpnpase 4 ame: full=inositol polyphosphate 1-phosphatase 4 short=ippase 4 ame: full=inositol- -bisphosphate 1-phosphatase 4	
vo_c1_Locus_18404_Transcript_3/6_Confidence_0.156_Length_7696	-5,2295594	3,53E-05	vp13e_dicdi ame: full= vacuolar protein sorting-associated protein 13e	-
vo_c2_Locus_2249_Transcript_4/4_Confidence_0.200_Length_632	-5,2347844	3,33E-05	star3_danre ame: full= -related lipid transfer protein 3 ame: full=mln64-like protein ame: full=start domain-containing protein 3 short= 3	
vo_n1_Locus_29_Transcript_412/1145_Confidence_0.012_Length_3945	-5,2632852	1,47E-05	otogl_human ame: full=otogelin-like protein flags: precursor	
vo_n1_Locus_153_Transcript_6/12_Confidence_0.238_Length_1610	-5,2965121	1,20E-05	huwe1_mouse ame: full=e3 ubiquitin-protein ligase huwe1 ame: full=e3histone ame: full= uba and wwe domain-containing protein 1 ame: full=upstream regulatory element-binding protein 1 short=ure-b1 short=ure-binding protein 1	
vo_c1_Locus_35331_Transcript_1/4_Confidence_0.938_Length_822	-5,3264624	6,76E-06	---NA---	
vo_n2_Locus_1294_Transcript_19/21_Confidence_0.238_Length_1084	-5,3744602	6,76E-06	fxyd4_human ame: full=fxyd domain-containing ion transport regulator 4 flags: precursor	
vo_c3_Locus_1194_Transcript_16/22_Confidence_0.235_Length_1025	-5,4441534	4,46E-06	chll_zygcr ame: full=light-independent protochlorophyllide reductase iron-sulfur atp-binding protein short=dpor subunit l short=li-por subunit l	-
vo_c2_Locus_2645_Transcript_1/2_Confidence_0.333_Length_2635	-5,4493412	3,08E-06	---NA---	
tri_c_comp19146_c0_seq2	-5,4689976	2,58E-06	rasl2_human ame: full=ras gtpase-activating protein 4 ame: full=calcium-promoted ras inactivator ame: full=ras p21 protein activator 4 ame: full= -activating-like protein 2	-
vo_c1_Locus_20303_Transcript_1/3_Confidence_0.200_Length_1013	-5,4986546	1,94E-06	ccm1_yeast ame: full=mitochondrial group i intron splicing factor ccm1 ame: full=cob and cox1 mrna maturation protein 1 ame: full=required for respiratory growth protein 2 flags: precursor	

vo_n2_Locus_3_Transcript_18921/24291_Confidence_0.000_Length_847	-5,5016389	2,49E-06	eutc_xanc8 ame: full=ethanolamine ammonia-lyase light chain ame: full=ethanolamine ammonia-lyase small subunit	
vo_c1_Locus_10050_Transcript_2/2_Confidence_0.000_Length_733	-5,5961209	8,93E-07	mok11_schpo ame: full=cell wall alpha- -glucan synthase mok11	
tri_c_comp19834_c0_seq2	-5,6652781	2,66E-07	y3030_ralso ame: full=upf0192 protein rsc3030 flags: precursor	-
vo_n1_Locus_3505_Transcript_1/9_Confidence_0.250_Length_854	-5,6965116	2,66E-07	roc1_arath ame: full=ribonucleoprotein chloroplastic flags: precursor	-
vo_c1_Locus_788_Transcript_5/34_Confidence_0.340_Length_1423	-5,7096872	1,74E-07	pgky_wheat ame: full=phosphoglycerate cytosolic	EC:2.7.2.3
vo_c1_Locus_28880_Transcript_1/1_Confidence_0.000_Length_259	-5,7101271	1,52E-07	ispe_brebn ame: full=4-diphosphocytidyl-2-c-methyl-d-erythritol kinase short=cmk ame: full=4-(cytidine-5 -diphospho)-2-c-methyl-d-erythritol kinase	
vo_c1_Locus_754_Transcript_7/11_Confidence_0.250_Length_1706	-5,7777633	1,04E-07	ucri_synsc ame: full=cytochrome b6-f complex iron-sulfur subunit ame: full=plastohydroquinone:plastocyanin oxidoreductase iron-sulfur protein short=isp short=risp ame: full=rieske iron-sulfur protein	EC:1.10.9.1; EC:1.10.2.2
vo_c2_Locus_8643_Transcript_13/30_Confidence_0.208_Length_6402	-5,8271265	5,53E-08	pag_bacan ame: full=protective antigen short=pa ame: full=anthrax toxins translocating protein ame: full=pa-83 short=pa83 contains: ame: full=protective antigen pa-20 short=pa20 contains: ame: full=protective antigen pa-63 short=pa63 flags: precursor	
tri_c_comp18645_c0_seq15	-5,8754594	2,34E-08	miaa_ruepo ame: full=trna dimethylallyltransferase ame: full=dimethylallyl diphosphate:trna dimethylallyltransferase short=dmapp:trna dimethylallyltransferase short=dmatase ame: full=isopentenyl-diphosphate:trna isopentenyltransferase short=ipp transferase short=ippt short=iptase	-
vo_c1_Locus_1583_Transcript_36/50_Confidence_0.307_Length_1248	-5,9197652	1,55E-08	hair_rat ame: full=lysine-specific demethylase hairless	
vo_n2_Locus_3_Transcript_4251/24291_Confidence_0.000_Length_957	-6,0237006	2,45E-09	fcpb_macpy ame: full=fucoxanthin-chlorophyll a-c binding protein chloroplastic flags: precursor	-

vo_c1_Locus_5520_Transcript_11/15_Confidence_0.316_Length_1184	-6,1684164	2,06E-10	---NA---	
vo_n1_Locus_888_Transcript_30/39_Confidence_0.387_Length_1527	-6,302068	0,006226802	dtd_bdeba ame: full=d-tyrosyl-trna deacylase	
tri_c_comp31150_c0_seq1	-6,3585216	3,25E-12	---NA---	
vo_c1_Locus_23313_Transcript_1/1_Confidence_0.000_Length_1456	-6,4198624	2,64E-12	no66_drope ame: full=bifunctional lysine-specific demethylase and histidyl-hydroxylase no66 ame: full=histone lysine demethylase no66	
tri_c_comp18645_c0_seq21	-6,4882054	2,18E-13	miaa_ruepo ame: full=trna dimethylallyltransferase ame: full=dimethylallyl diphosphate:trna dimethylallyltransferase short=dmapp:trna dimethylallyltransferase short=dmatase ame: full=isopentenyl-diphosphate:trna isopentenyltransferase short=ipp transferase short=ippt short=iptase	-
vo_c1_Locus_7662_Transcript_13/16_Confidence_0.149_Length_4207	-6,8054197	2,65E-15	if2p_human ame: full=eukaryotic translation initiation factor 5b short=eif-5b ame: full=translation initiation factor if-2	-
vo_c3_Locus_1421_Transcript_4/4_Confidence_0.125_Length_941	-6,8546	8,49E-15	rsmg_agrvs ame: full=ribosomal rna small subunit methyltransferase g ame: full=16s rrna 7-methylguanosine methyltransferase short=16s rrna m7g methyltransferase	
vo_c2_Locus_17796_Transcript_2/4_Confidence_0.429_Length_3172	-6,9393897	9,34E-17	sr140_mouse ame: full=u2 snrnp-associated surp motif-containing protein ame: full=140 kda ser arg-rich domain protein ame: full=u2-associated protein sr140	-
vo_c1_Locus_242_Transcript_37/44_Confidence_0.372_Length_935	-7,0330204	1,29E-17	arf_vigun ame: full=adp-ribosylation factor	-
vo_c1_Locus_947_Transcript_1/7_Confidence_0.286_Length_2009	-7,2300493	4,02E-20	hmox2_macfa ame: full=heme oxygenase 2 short=ho-2	EC:1.14.99.3; EC:3.1.4.4
tri_c_comp40688_c0_seq1	-7,3404701	3,60E-23	chln_proma ame: full=light-independent protochlorophyllide reductase subunit n short=dpor subunit n short=li-por subunit n	

Supplementary Table S10. List of KEGG pathways associated to contigs with assigned enzyme codes in up-regulated DEGs. The table contains pathway names, pathway ID, number of enzymes found for each pathway, enzyme codes and sequence names.

Pathway	Pathway ID	#Enzs in Pathway	Enzyme	#Seqs of Enzyme	Seqs
Nitrogen metabolism	map00910	2	ec:1.7.1.1 - reductase (NADH), ec:1.7.1.4 - reductase [NAD(P)H]	1, 1	tri_n_comp24956_c0_seq1, vo_n2_Locus_128_Transcript_8/33_Confidence_0.085_Length_4154
Pyruvate metabolism	map00620	2	ec:1.1.1.37 - dehydrogenase, ec:1.1.1.82 - dehydrogenase (NADP+)	1, 1	vo_c1_Locus_7371_Transcript_1/4_Confidence_0.500_Length_1352, vo_c1_Locus_7371_Transcript_1/4_Confidence_0.500_Length_1352
Biosynthesis of antibiotics	map01130	2	ec:1.1.1.37 - dehydrogenase, ec:1.1.1.35 - dehydrogenase	1, 1	vo_c1_Locus_7371_Transcript_1/4_Confidence_0.500_Length_1352, vo_n2_Locus_636_Transcript_3/16_Confidence_0.306_Length_685
Carbon fixation pathways in prokaryotes	map00720	2	ec:1.1.1.37 - dehydrogenase, ec:1.1.1.35 - dehydrogenase	1, 1	vo_c1_Locus_7371_Transcript_1/4_Confidence_0.500_Length_1352, vo_n2_Locus_636_Transcript_3/16_Confidence_0.306_Length_685
Fatty acid degradation	map00071	2	ec:1.1.1.35 - dehydrogenase, ec:4.2.1.74 - hydratase	1, 1	vo_n2_Locus_636_Transcript_3/16_Confidence_0.306_Length_685, vo_n2_Locus_636_Transcript_3/16_Confidence_0.306_Length_685
Glycerolipid metabolism	map00561	2	ec:2.3.1.20 - O-acyltransferase, ec:2.3.1.22 - O-acyltransferase	1, 1	vo_n2_Locus_19192_Transcript_2/2_Confidence_0.250_Length_3053, vo_n2_Locus_19192_Transcript_2/2_Confidence_0.250_Length_3053
Fatty acid elongation	map00062	2	ec:1.1.1.35 - dehydrogenase, ec:4.2.1.74 - hydratase	1, 1	vo_n2_Locus_636_Transcript_3/16_Confidence_0.306_Length_685, vo_n2_Locus_636_Transcript_3/16_Confidence_0.306_Length_685
Carbon fixation in photosynthetic organisms	map00710	2	ec:1.1.1.37 - dehydrogenase, ec:1.1.1.82 - dehydrogenase (NADP+)	1, 1	vo_c1_Locus_7371_Transcript_1/4_Confidence_0.500_Length_1352, vo_c1_Locus_7371_Transcript_1/4_Confidence_0.500_Length_1352
Phenylpropanoid biosynthesis	map00940	1	ec:1.11.1.7 - lactoperoxidase	1	vo_c1_Locus_1107_Transcript_1/4_Confidence_0.143_Length_1311
Glyoxylate and dicarboxylate metabolism	map00630	1	ec:1.1.1.37 - dehydrogenase	1	vo_c1_Locus_7371_Transcript_1/4_Confidence_0.500_Length_1352
Tryptophan metabolism	map00380	1	ec:1.1.1.35 - dehydrogenase	1	vo_n2_Locus_636_Transcript_3/16_Confidence_0.306_Length_685
Lysine degradation	map00310	1	ec:1.1.1.35 - dehydrogenase	1	vo_n2_Locus_636_Transcript_3/16_Confidence_0.306_Length_685
Caprolactam degradation	map00930	1	ec:1.1.1.35 - dehydrogenase	1	vo_n2_Locus_636_Transcript_3/16_Confidence_0.306_Length_685
Butanoate metabolism	map00650	1	ec:1.1.1.35 - dehydrogenase	1	vo_n2_Locus_636_Transcript_3/16_Confidence_0.306_Length_685
Various types of N-glycan biosynthesis	map00513	1	ec:2.4.1.101 - 2-beta-N-acetylglucosaminyltransferase	1	vo_n2_Locus_19192_Transcript_2/2_Confidence_0.250_Length_3053
Primary bile acid biosynthesis	map00120	1	ec:1.1.1.35 - dehydrogenase	1	vo_n2_Locus_636_Transcript_3/16_Confidence_0.306_Length_685

Cutin, suberine and wax biosynthesis	map00073	1	ec:2.3.1.20 - O-acyltransferase	1	vo_n2_Locus_19192_Transcript_2/2_Confidence_0.250_Length_3053
Thiamine metabolism	map00730	1	ec:3.6.1.15 - phosphatase	1	vo_n1_Locus_17075_Transcript_2/4_Confidence_0.400_Length_811
Steroid hormone biosynthesis	map00140	1	ec:1.1.1.51 - 17)beta-hydroxysteroid dehydrogenase	1	vo_n2_Locus_636_Transcript_3/16_Confidence_0.306_Length_685
Cysteine and methionine metabolism	map00270	1	ec:1.1.1.37 - dehydrogenase	1	vo_c1_Locus_7371_Transcript_1/4_Confidence_0.500_Length_1352
Purine metabolism	map00230	1	ec:3.6.1.15 - phosphatase	1	vo_n1_Locus_17075_Transcript_2/4_Confidence_0.400_Length_811
Steroid degradation	map00984	1	ec:1.1.1.51 - 17)beta-hydroxysteroid dehydrogenase	1	vo_n2_Locus_636_Transcript_3/16_Confidence_0.306_Length_685
Geraniol degradation	map00281	1	ec:1.1.1.35 - dehydrogenase	1	vo_n2_Locus_636_Transcript_3/16_Confidence_0.306_Length_685
Valine, leucine and isoleucine degradation	map00280	1	ec:1.1.1.35 - dehydrogenase	1	vo_n2_Locus_636_Transcript_3/16_Confidence_0.306_Length_685
Benzoate degradation	map00362	1	ec:1.1.1.35 - dehydrogenase	1	vo_n2_Locus_636_Transcript_3/16_Confidence_0.306_Length_685
Other glycan degradation	map00511	1	ec:3.2.1.24 - alpha-D-mannosidase	1	vo_n2_Locus_19192_Transcript_2/2_Confidence_0.250_Length_3053
Methane metabolism	map00680	1	ec:1.1.1.37 - dehydrogenase	1	vo_c1_Locus_7371_Transcript_1/4_Confidence_0.500_Length_1352
Toluene degradation	map00623	1	ec:1.1.1.35 - dehydrogenase	1	vo_n2_Locus_636_Transcript_3/16_Confidence_0.306_Length_685
N-Glycan biosynthesis	map00510	1	ec:2.4.1.101 - 2-beta-N-acetylglucosaminyltransferase	1	vo_n2_Locus_19192_Transcript_2/2_Confidence_0.250_Length_3053
Citrate cycle (TCA cycle)	map00020	1	ec:1.1.1.37 - dehydrogenase	1	vo_c1_Locus_7371_Transcript_1/4_Confidence_0.500_Length_1352

Supplementary Table S11. List of KEGG pathways associated to contigs with assigned enzyme codes in down-regulated DEGs. The table contains pathway names, pathway ID, number of enzymes found for each pathway, enzyme codes and sequence names.

Pathway	Pathway ID	#Enzs in Pathway	Enzyme	#Seqs of Enzyme	Seqs
Biosynthesis of antibiotics	map01130	2	ec:1.1.1.42 - dehydrogenase (NADP+), ec:2.7.2.3 - kinase	1, 1	vo_c1_Locus_5705_Transcript_16/19_Confidence_0.489_Length_4193, vo_c1_Locus_788_Transcript_5/34_Confidence_0.340_Length_1423
Inositol phosphate metabolism	map00562	1	ec:2.7.1.68 - 5-kinase	1	vo_n1_Locus_5719_Transcript_37/41_Confidence_0.500_Length_5524
Carbon fixation pathways in prokaryotes	map00720	1	ec:1.1.1.42 - dehydrogenase (NADP+)	1	vo_c1_Locus_5705_Transcript_16/19_Confidence_0.489_Length_4193
Glycolysis / Gluconeogenesis	map00010	1	ec:2.7.2.3 - kinase	1	vo_c1_Locus_788_Transcript_5/34_Confidence_0.340_Length_1423
Glutathione metabolism	map00480	1	ec:1.1.1.42 - dehydrogenase (NADP+)	1	vo_c1_Locus_5705_Transcript_16/19_Confidence_0.489_Length_4193
Starch and sucrose metabolism	map00500	1	ec:2.4.1.34 - synthase	1	vo_n2_Locus_1515_Transcript_20/24_Confidence_0.103_Length_4140
Carbon fixation in photosynthetic organisms	map00710	1	ec:2.7.2.3 - kinase	1	vo_c1_Locus_788_Transcript_5/34_Confidence_0.340_Length_1423
Oxidative phosphorylation	map00190	1	ec:1.10.2.2 - reductase	1	vo_c1_Locus_754_Transcript_7/11_Confidence_0.250_Length_1706
Ether lipid metabolism	map00565	1	ec:3.1.4.4 - D	1	vo_c1_Locus_947_Transcript_1/7_Confidence_0.286_Length_2009
Glycerophospholipid metabolism	map00564	1	ec:3.1.4.4 - D	1	vo_c1_Locus_947_Transcript_1/7_Confidence_0.286_Length_2009
Phosphatidylinositol signaling system	map04070	1	ec:2.7.1.68 - 5-kinase	1	vo_n1_Locus_5719_Transcript_37/41_Confidence_0.500_Length_5524
Photosynthesis	map00195	1	ec:1.10.9.1 - reductase	1	vo_c1_Locus_754_Transcript_7/11_Confidence_0.250_Length_1706
Citrate cycle (TCA cycle)	map00020	1	ec:1.1.1.42 - dehydrogenase (NADP+)	1	vo_c1_Locus_5705_Transcript_16/19_Confidence_0.489_Length_4193
